# A general catalytic synthetic strategy for highly strained methylenecyclobutanes and spiromethylenecyclobutanes†

**DOI:** 10.1039/d3sc01103h

**Published:** 2023-06-09

**Authors:** Haotian Zhao, Yu Lin, Mingyu Jiang, Bo Su

**Affiliations:** a State Key Laboratory of Medical Chemical Biology, College of Pharmacy, Nankai University 38 Tongyan Road, Jinnan District Tianjin 300350 P. R. China subo@nankai.edu.cn

## Abstract

Highly strained methylenecyclobutanes (MCBs) are intriguing scaffolds in synthetic chemistry and drug discovery, but there is no such strategy that enables the synthesis of structurally diverse MCBs with defined stereochemistry. We report a general synthetic strategy for (boromethylene)cyclobutanes (BMCBs) and spiro-BMCBs by a challenging Cu-catalyzed highly chemo-, stereo-, and regioselective borylative cyclization of aliphatic alkynes. This strategy not only enables the installation of various functionalities at each site on the MCB skeleton with unambiguous stereochemistry but also introduces a versatile boromethylene unit that is readily transformable to a wide range of new functional groups; these features significantly expand the structural diversity of MCBs and are particularly valuable in drug discovery. The concise and divergent total syntheses of four cyclobutane-containing natural products were achieved from one common BMCB obtained by this strategy. The origin of the high regioselectivity in the borylcupration of alkynes and the high efficiency of the strained ring cyclization was also studied.

## Abstract 2

We developed a copper-catalyzed stereoselective strategy for the unified synthesis of borylated methylenecyclobutanes. The versatile boromethylene unit greatly expands their structural diversity and potential in future drug discovery.

## Graphical abstract

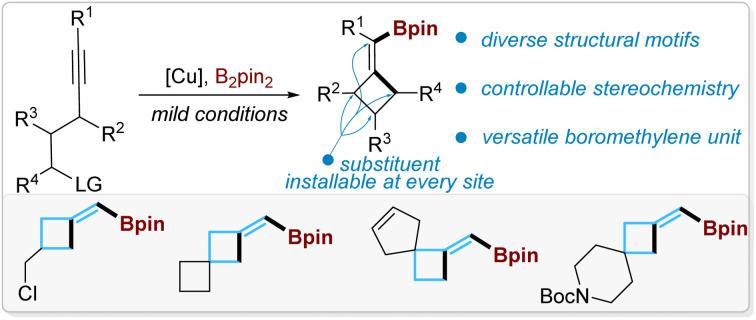

## Introduction

**Fig. 1:**
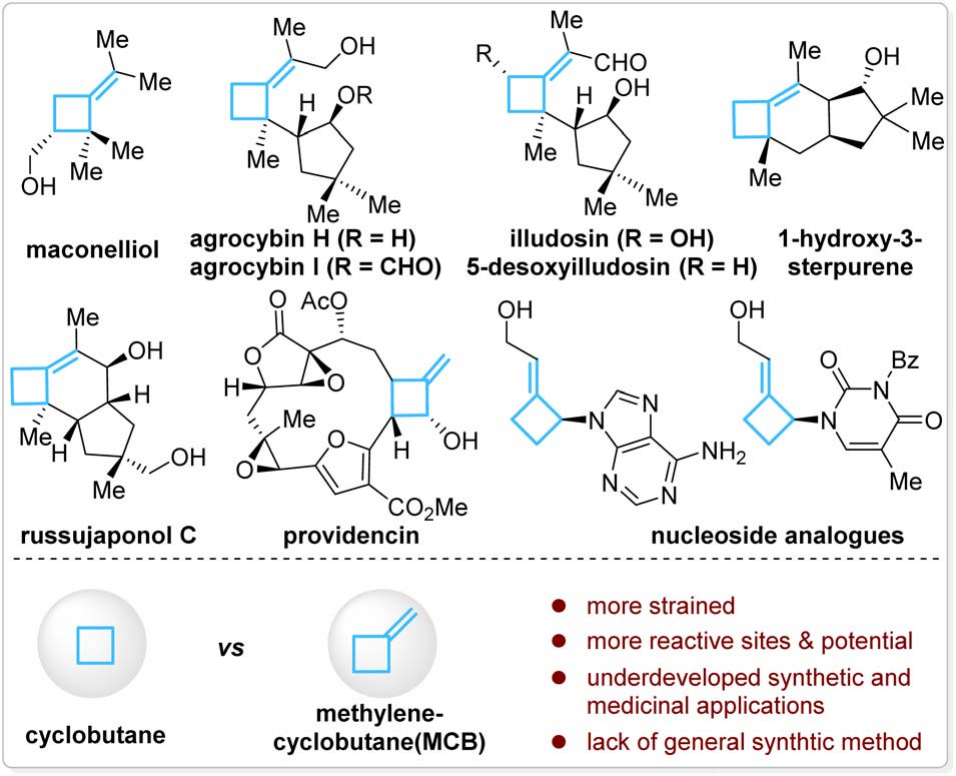
MCB and MCB-containing natural products and drug candidates.

Methylenecyclobutanes (MCBs) are a subclass of structurally special cyclobutanes and are found in a few natural products and drug candidates (Fig. 1, top).^1^ Compared with cyclobutanes, the presence of an *exo*-methylene unit makes MCBs more strained and provides them with more reactive sites in such a small ring system—the allylic C–H bonds, the C–C double bond, and the bent C–C single bonds (Fig. 1, bottom).^2^ With these unique properties, MCBs should have found more extensive applications in synthetic chemistry, but in fact, they are much less explored than other strained carbocycles, such as cyclobutanes, methylenecyclopropanes, and other cyclopropane derivatives.^3^ The lack of general synthetic strategies for MCBs is one of the major reasons for their underdevelopment in organic synthesis and medicinal chemistry.^3*a*^

**Scheme 1:**
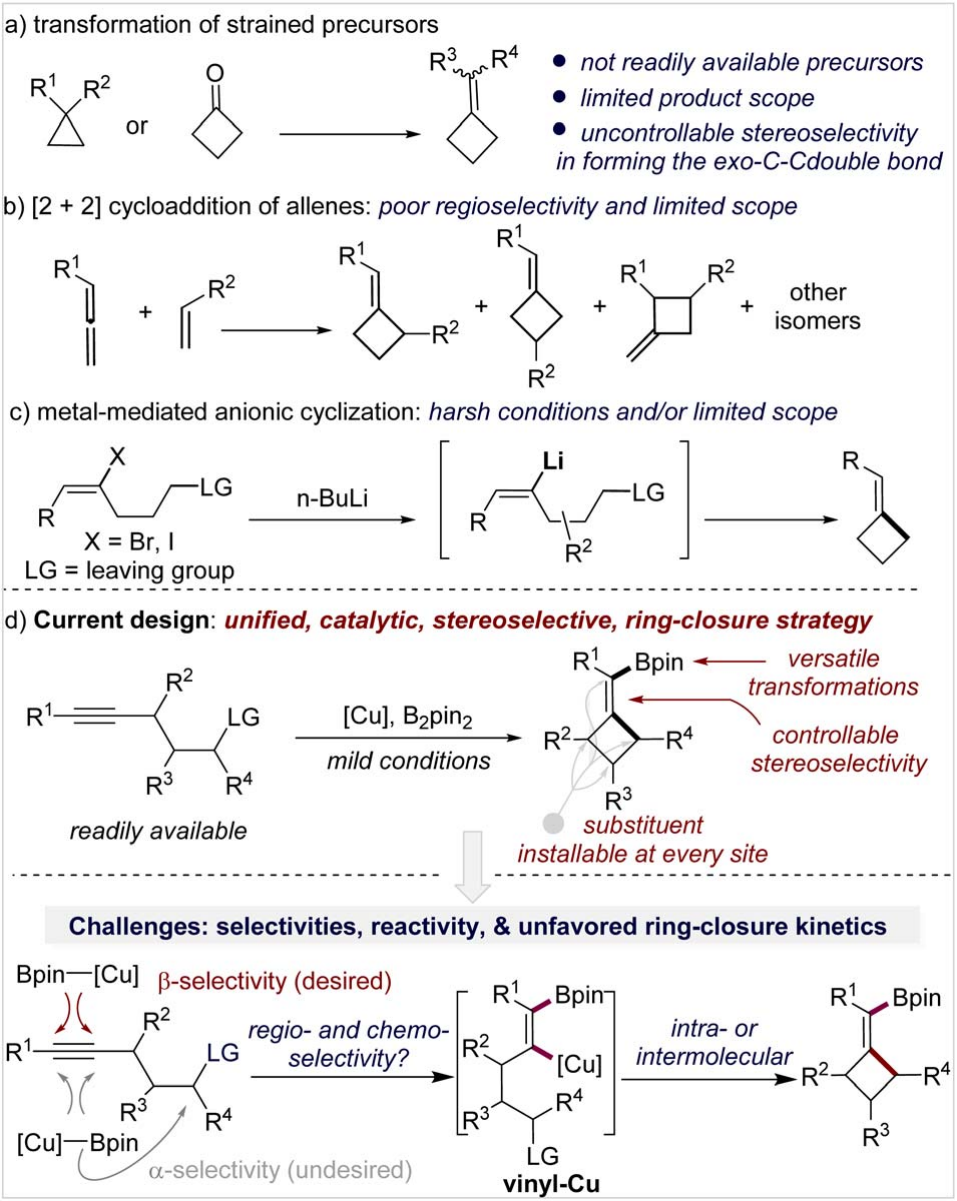
Strategies for the construction of methylenecyclobutanes.

Generally, there are three strategies to access MCBs, but each has limitations in terms of availability of starting materials, stereo- and regioselectivity control, functionality tolerance, and substitution pattern of the product. Transformation of small-ring carbocycles is one approach (Scheme 1a)^4^ but is limited by the poor availability of precursors and no stereocontrol in forming the exocyclic double bond. [2 + 2]-Cycloaddition of an allene and alkene is the second approach (Scheme 1b),^5^ in which the control of regio- and stereoselectivity is a longstanding challenge, particularly when it occurs in an intermolecular fashion. Anionic cyclization of an alkenyl halide with a pendant leaving group is the third approach (Scheme 1c); this approach is straightforward and forms products with defined stereochemistry but only tolerates simple functional groups due to the use of stoichiometric reactive organolithium reagents.^6^ Although other miscellaneous methods have also been developed,^7^ the aforementioned limitations still exist.

Therefore, the development of a new synthetic approach that can avoid or largely reduce these limitations is required, and it will be particularly attractive if the downstream requirements in drug discovery can be taken into consideration. The replacement of flat aromatic rings with non-classical, C(sp^3^)-rich bioisosteres (*e.g.*, cyclobutanes, spirocyclobutanes, bicyclo[1,1,1]pentanes, *etc.*) has emerged as a popular strategy in medicinal chemistry.^1*a*,8,9^ This strategy can dramatically accelerate the speed of discovering new lead molecules by dexterously circumventing the existing intellectual property regions and provides a valuable approach for tuning the structural rigidity and modifying pharmacophores in three-dimensional space.^8*c*,10^ In this context, the past several years have witnessed an increasing development of new synthetic methodology for cyclobutanes and spirocyclobutanes;^11^ however, MCBs seem to have been ignored again in this renaissance. The coincidental underdevelopment of MCBs in synthetic chemistry and drug discovery prompted us to design a general synthetic approach for MCBs.

Inspired by the anionic alkenyl halide cyclization which occurs through a vinyl metal intermediate (Scheme 1c) and as part of our continuous interest in organoboron chemistry,^12^ we envisioned that a copper-catalyzed borylative cyclization of alkynes to form (boromethylene)cyclobutanes (BMCB) should be an attractive approach (Scheme 1d, top). This catalytic strategy, if successfully implemented, will enable the installation of various substituents at each site of the core skeleton with defined stereochemistry and introduce a versatile boromethylene unit that is a precursor to a broad range of functionalities. These features can greatly expand the chemical space of MCBs and are particularly valuable in drug discovery.

Copper-catalyzed borylative cyclization of alkenes to synthesize carbocycles has been developed.^13^ For example, Ito and co-workers reported seminal work on the cyclization of vinylsilanes.^13*a*,*b*^ However, the implementation of such a cyclization on aliphatic alkynes to construct carbocycles is challenging. Several hurdles associated with the regio- and chemoselectivity, reactivity, and unfavorable kinetics in the ring-closure process need to be overcome (Scheme 1d, bottom).^14^ First, the borylcupration of alkynes must occur β-selectively to form the vinyl copper intermediate vinyl-Cu, locating the copper and the pendant alkyl electrophile at the geminal carbon; the challenge associated with this process is reflected by the fact that although the borylative cyclization of electronically biased alkynes, containing a heteroatom at the acetylenic or (homo)propargylic position, proceeds efficiently to form 5- and 6-membered heterocycles,^15^ attempts to synthesize the corresponding carbocycles from alkynes failed or gave very low yield.^15*d*^ Second, multiple chemoselectivities need to be controlled: the borylcopper species [Cu]-Bpin must chemoselectively react with the alkyne instead of the alkyl electrophiles, and the resulting vinyl-Cu must undergo the alkylation in an intramolecular fashion to form the strained MCB scaffold rather than in an intermolecular fashion to form an alkylated alkene. Third, the reactivity of vinyl-Cu and the alkyl electrophile must be well controlled; because the vinyl copper species is less nucleophilic than the corresponding alkyl copper intermediate (generated from borylcupration of an alkene), reactive alkylating reagents (*e.g.*, methyl-, benzyl-, and allyl halides) are often required for the alkylation of vinyl copper, but the use of reactive electrophiles would make the aforementioned chemoselectivities associated with the [Cu]-Bpin (with the alkyne or the alkyl electrophile) and vinyl-Cu (intra- *vs.* inter-) more complicated.^16^ Moreover, strained four-membered ring closure is kinetically unfavored, and such a catalytic process will be more difficult.

Herein we report the methodology development of the borylative cyclization of aliphatic alkynes to form structurally diverse (spiro-)BMCBs. The versatile transformations of the newly introduced boromethylene unit greatly expand the structural diversity of (spiro-)MCBs. The application of this strategy is further showcased by the concise and divergent total synthesis of cyclobutane-containing natural products. The origin of the high regioselectivity in the borylcupration of alkynes and the high efficiency of the ring-closure process is also disclosed.

## Results and discussion

### Conditions optimization

To identify the conditions for the borylative cyclization, we first examined terminal alkynes tethered with various leaving groups (Table 1, entries 1–4). Under conditions with B_2_pin_2_ and copper complexes derived from CuCl_2_, BINAP, and *t*BuOK in THF at 50 °C, the reaction with 4-pentyn-1-ol tosylate formed BMCB 2a in a higher yield (entry 4, 42%) than those with substrates containing other leaving groups (entries 1–3; Br, OP(O)(OEt)_2_, and OMs). Reactions at higher (80 °C) or lower temperatures formed product 2a in slightly decreased yields (entries 5 and 6). Evaluation of ligands showed that the reaction with an N-heterocyclic carbene (IMes) generated *in situ* from 1,3-bis(2,4,6-trimethylphenyl)imidazolium chloride (L3) gave a higher 71% yield than those with other ligands, such as PPh_3_, xantphos, and other NHC ligands (entries 4 and 7–11). Structurally similar L2 showed lower efficiency than L3 in the reaction, probably due to the increased steric bulkiness caused by the bulky isopropyl group on it. Various alkoxide bases were also tested; the yield of 2a decreased significantly when *t*BuOK was replaced with other bases (entries 11–14). Further examination of copper salts and reaction time revealed that the reaction conducted with CuCl for a prolonged time (20 h) formed BMCB 2a in a synthetically practical yield (83%). It is worth noting that by-products derived from borylative protonation of the alkyne were observed under some conditions (detailed information is presented in the section of Mechanistic studies).

**Table 1:** Conditions optimization^a^

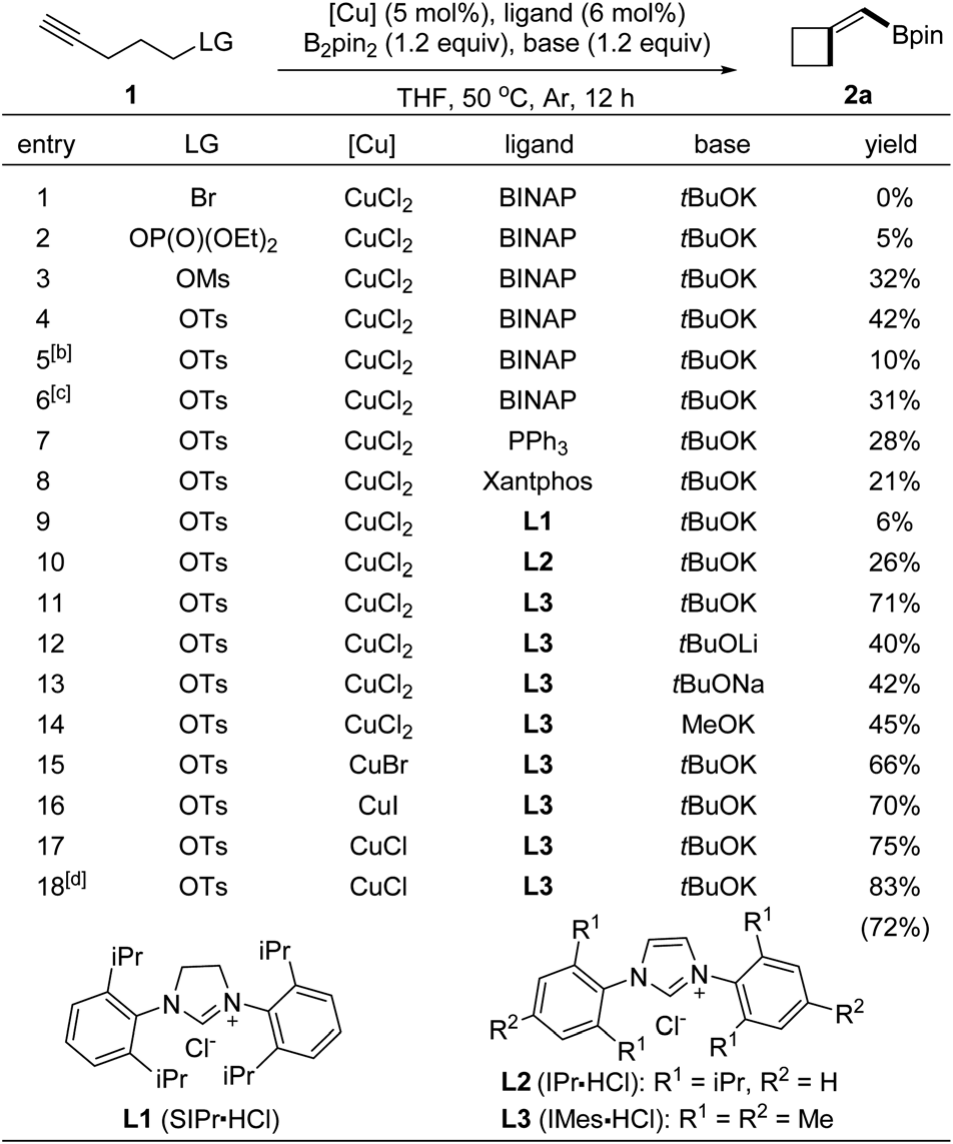

a Reaction was conducted with 1a (0.1 mmol) in THF (1.0 mL), and the yield refers to ^1^H NMR yield with 1,3,5-trimethoxybenzene as internal standard.

b Reaction temperature: 25 °C.

c Reaction temperature: 80 °C.

d Reaction was conducted for 24 h, and the yield in parentheses refers to isolated yield.

### Scope of the reaction

**Fig. 2:**
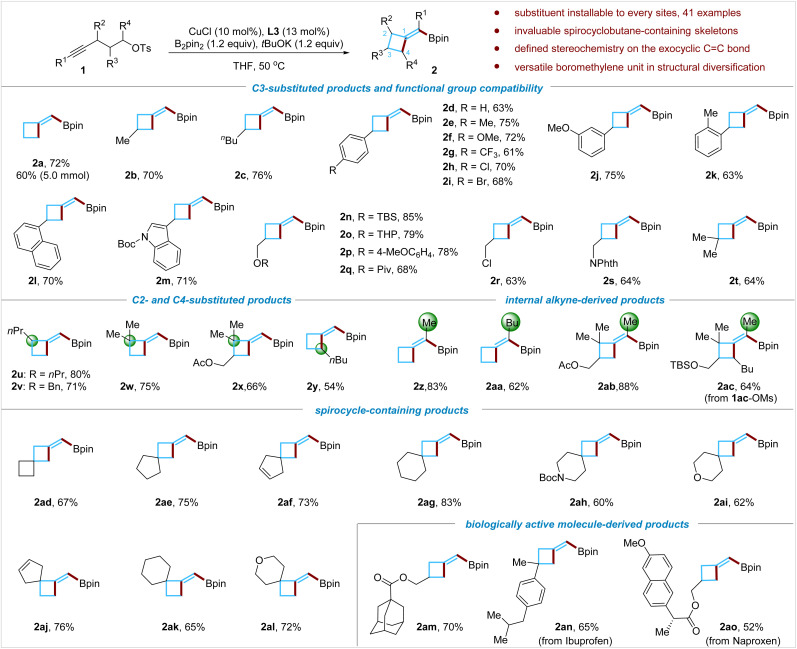
Scope of the borylative cyclization of aliphatic alkynes.

Having established the conditions to obtain BMCB 2a in high yield, we examined the scope of the reaction (Fig. 2). A wide range of functional groups, such as alkyl groups (1b and 1c), various substituted aryl groups (1d–1k), a naphthyl group (1l), and an indolyl group (1m), were compatible with the reaction conditions; the reaction also tolerates heteroatom-based functionalities, including a silyl ether (1n), a ketal group (1o), an aryl ether (1p), an ester (1q), an alkyl chloride (1r), and an imide (1s), affording products 2n–2s in 63–85% yields. The reaction was easily scalable: when the reaction of 1a was conducted on a larger scale (5 mmol), a yield (60%) similar to that on a smaller scale (0.2 mmol) was obtained.

Methylenecyclobutanes bearing an allylic quaternary all-carbon center are common motifs in natural products but are difficult to access by existing methods.^1*c*,3*a*,3*b*^ Reactions of sterically congested alkynes and an alkyne tethered with a secondary alkyl tosylate were tested; these substrates enable the installation of substituents at both allylic positions of BMCB products. The borylative cyclization of alkynes bearing tertiary (1u and 1v) or quaternary (1w and 1x) propargyl carbon centers proceeded smoothly, affording products 2u–2x in 66–80% yields. When an alkyne tethered with a secondary alkyl tosylate (1y) was subjected to the standard conditions, product 2y formed successfully, albeit in moderate yield (54%). The utilization of unactivated secondary alkyl electrophiles in the copper-catalyzed borylative alkylation of any unsaturated C–C bonds has been challenging due to their low reactivity; to the best of our knowledge, this is the first report on the Cu-catalyzed borylative alkylation of an alkyne with a secondary alkylating reagent, which might inspire more studies in this direction in the future.

Internal aliphatic alkynes that could form tetrasubstituted MCBs were further evaluated. Reactions with internal alkynes 1z to 1ac afforded the corresponding products 2z to 2ac in 62% to 83% yields, respectively, and no obvious by-products derived from the undesired α-regioselective borylcupration process were detected by the ^1^H NMR analysis of the crude reaction mixture. Regioselectivity control has been particularly difficult for copper-catalyzed borylative functionalization of internal aliphatic alkynes,^15*c*^ and the high regioselectivity observed in this transformation is noteworthy.

Cyclobutane-containing spirocyclic scaffolds are of great interest in medicinal chemistry and have recently attracted increasing attention.^8*b*,9*b*,9*c*,11*h*^ By this borylative cyclization strategy, structurally diverse spiro-BMCBs were readily prepared; spiro[3.3]heptane (2ad), spiro[3.4]octane (2ae), spiro[3.4]oct-6-enes (2af and 2aj), spiro[3.5]nonanes (2ag and 2ak), azaspiro[3.5]nonane (2ah), and oxaspiro[3.5]nonanes (2ai and 2al) were obtained in good to excellent yields under standard conditions. These spirocycles are difficult to synthesize by traditional methods, and the newly installed boromethylene unit is flexible for further structural modifications (for versatile transformations of the boromethylene unit, *vide infra*).

The borylative cyclization reaction was also assessed for introducing the BMCB scaffold in biologically active molecules. For example, the reaction with the adamantane-1-carboxylic acid derivative afforded product 2am in 70% yield, and the substrates (1an and 1ao) derived from nonsteroidal anti-inflammatory drugs, ibuprofen and naproxen, formed the BMCB-containing products 2an and 2ao in 65% and 52% yields, respectively.

### Transformations of the boromethylene unit

**Fig. 3:**
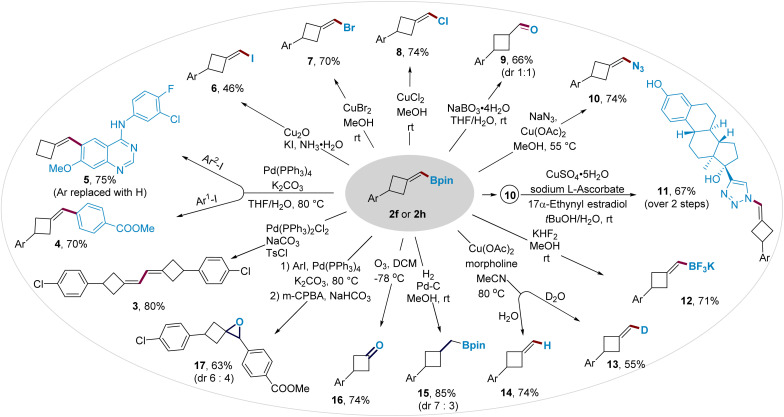
Versatile transformations of the boromethylene unit in BMCBs.

The boromethylene unit in BMCB 2 is poised for conversion to a wide range of new functionalities (Fig. 3). Boronate 2h was converted to diene 3 by a palladium-catalyzed homo-coupling reaction and to biaryls 4 and 5 by palladium-catalyzed cross-coupling reactions. The C–B bond in BMCB 2f was converted to a C–X bond (X = I, Br, or Cl) by copper-mediated halogenation, affording (halomethylene)cyclobutanes 6–8 in moderate to high yields. Oxidation of the C–B bond in 2f, followed by the enol–keto tautomerization, led to the formation of aldehyde 9 with a 1 : 1 diastereoselective ratio. An azide group was introduced on the methylene unit by a copper-mediated azidation of the C–B bond, affording MCB-containing azide 10 in 74% yield. The resulting alkenyl azide provides a useful handle to integrate the MCB scaffold into biologically active molecules through a copper-catalyzed click reaction; for example, azide 10 readily reacted with an estradiol derivative that bears an alkyne moiety to give compound 11 smoothly. Borate 2f underwent cleavage of the pinacolate by fluoride to form a more reactive trifluoroborate 12 and deborylative deuteriation/protonation catalyzed by copper to form labeled and unlabeled products 13 and 14, respectively.

Transformations of the exocyclic methylene moiety in BMCB 2 were also explored, affording other functionalized four-membered ring-containing products. The methylene unit underwent a palladium-catalyzed hydrogenation to form boromethyl cyclobutane 15 (d.r. = 7 : 3) and an ozonolysis to produce cyclobutanone 16. Epoxide-containing cyclobutane 17 was obtained in 63% yield and 6 : 4 d.r. from 2h by a sequential palladium-catalyzed cross-coupling and alkene epoxidation.

### Applications in total synthesis

**Fig. 4:**
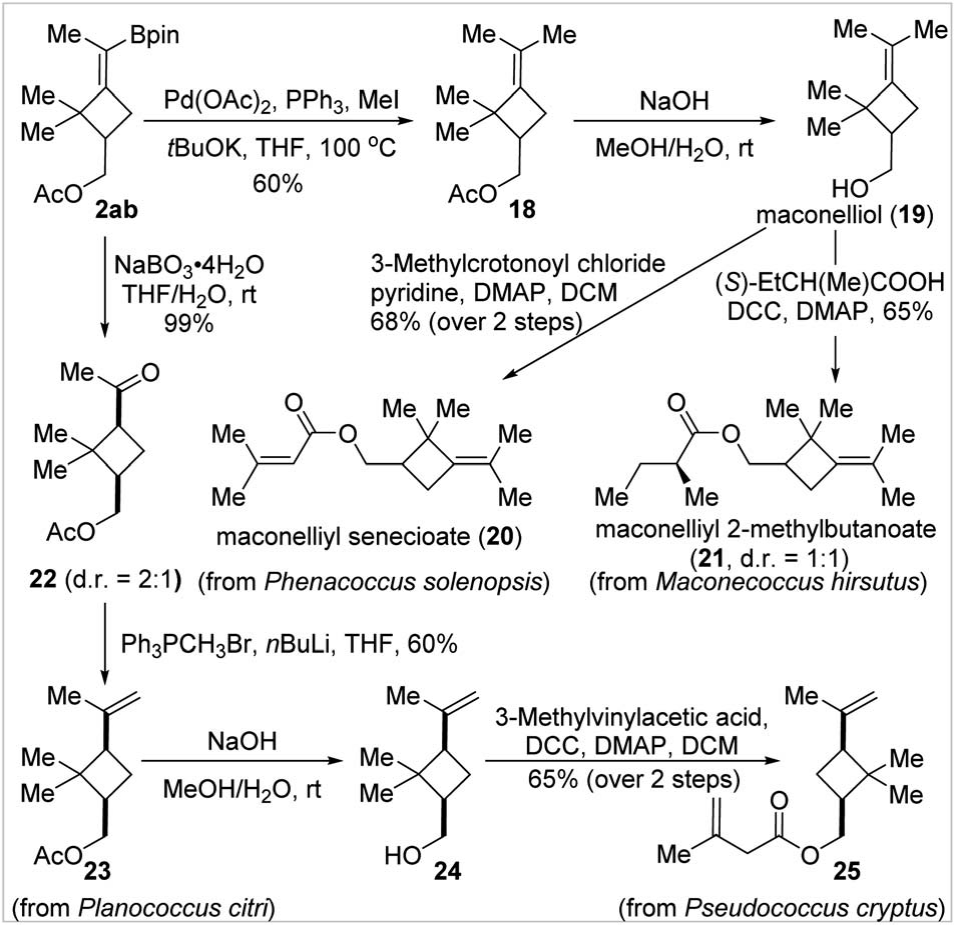
Concise and divergent total synthesis of cyclobutane-containing natural products.

To further showcase the usefulness of the BMCB products, we established the concise and divergent syntheses of four cyclobutane-containing natural products, 20, 21, 23, and 25, from a common BMCB 2ab in two to four steps (Fig. 4). These compounds are sex pheromones of several worldwide species of mealybugs—serious pests to a wide range of plants, including vegetables, fruits, and ornamental crops—and have found wide applications in the detection, monitoring, and attract-and-kill of pests in agriculture.^17^ Compounds 20 and 21, two MCB-containing sex pheromones, can be prepared by esterification of a common precursor, maconelliol 19, the synthesis of which required six steps from α-pinene as the starting material by a known method.^17*b*^ Using BMCB 2ab as the starting material, maconelliol 19 was readily obtained in 2 steps: a palladium-catalyzed methylation of the C–B bond in 2ab and removal of the acetyl group in compound 18 under basic conditions. Cyclobutane-containing sex pheromones 23 and 25 were synthesized from BMCB 2ab in two to four steps. Oxidation of the C–B bond in 2ab quantitatively afforded ketone 22 (*cis*/*trans* = 2 : 1), and the major *cis*-isomer was transformed to pheromone 23 in 60% yield by the Wittig olefination reaction. The acetyl group in 23 was replaced by a 3-methylbut-3-enoyl group by aqueous hydrolysis and subsequent esterification, affording 25 in 65% yield in 2 steps.

### Mechanistic studies

**Fig. 5:**
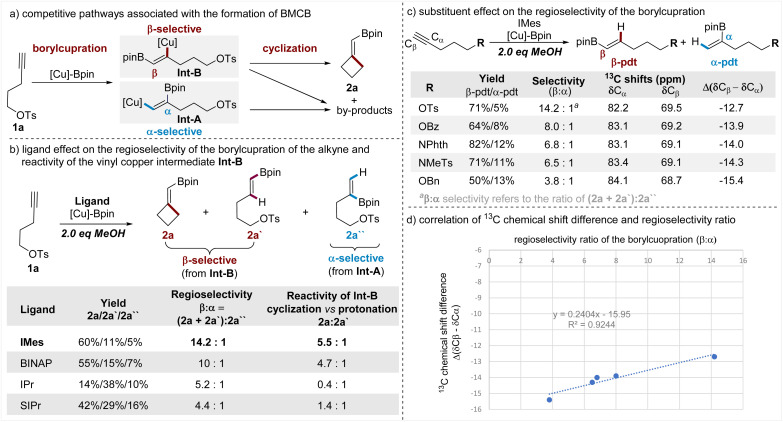
Mechanistic studies on the regioselectivity of the borylcupration and reactivity of the vinyl copper intermediate.

Having demonstrated the wide scope of this borylative cyclization, the versatile transformations of the BMCB products, and the concise total syntheses of cyclobutane-containing natural products, we attempted to disclose the key factors that are responsible for the observed high β-regioselectivity of the borylcupration of the alkyne and high efficiency of the subsequent strained four-membered ring-closure. To illustrate the key steps in the formation of BMCB 2a, a simplified reaction mechanism is depicted in Fig. 5a. Borylcupration of alkyne 1a occurs in a β-selective fashion to give the vinyl copper intermediate Int-B and in an α-selective way to form Int-A; cyclization of Int-B leads to the highly strained BMCB 2a, and other pathways associated with both Int-A and Int-B form by-products.

To probe the ligand effect on the regioselectivity of the borylcupration, we conducted reactions of 1a with varying ligands (Fig. 5b). It is worth noting that for the convenience of obtaining good mass balances (>90%) for these reactions and analyzing the distribution of products associated with both borylcupration processes, the standard conditions were modified to include 2 equiv. of MeOH. As the results show (Fig. 5b, column 3), IMes, the NHC ligand used under the optimized conditions, displays the highest β-selectivity (*β* : *α* ratio = 14.2 : 1) and BINAP shows slightly decreased selectivity (*β* : *α* ratio = 10 : 1), whereas more sterically demanding NHC ligands (IPr and SIPr) are dramatically less selective (5.2 : 1 and 4.4 : 1, respectively). These results indicate that the ligand (IMes) is crucial for the achieved high β-selectivity.

Protonation of vinyl copper intermediates is usually one of the major unproductive pathways in the borylative functionalization of alkynes due to the presence of trace amounts of water in the solvent and/or the reactants. The addition of a proton source (MeOH) in the reactions of 1a allowed us to directly compare the relative reactivities of the vinyl copper Int-B with the pendent alkyl electrophile and the proton, thus providing insights into the ligand influence on the efficiency of the strained ring-cyclization. The results (Fig. 5b, column 4) showed that the IMes ligand is also the most efficient ligand in promoting the cyclization (*vs.* the protonation) of Int-B. Even in the presence of 2 equiv. of MeOH, Int-B containing the IMes ligand largely favors the cyclization to form the strained BMCB 2a rather than the protonation to form protoborylation product 2a′ (2a/2a′ = 5.5 : 1), whereas Int-B containing other ligands is less favorable for the cyclization or even preferable for the protonation.

Besides the ligand effect, the pendent substituent (OTs) can also contribute to the observed high β-selectivity through steric, chelating, and/or electronic effects.^15*a*,*b*,*d*,18^ To distinguish between these possible effects, we synthesized alkynes tethered with various oxygen- and nitrogen-based substituents featuring similar steric and chelating but different electronic properties. These alkynes were subjected to otherwise the same conditions as the reaction of 1a, and the results are summarized in a sequentially decreasing β-selectivity (Fig. 5c, column 3). Generally, the alkyne with the strongest electronegative substituent (OTs) shows the highest selectivity and the one with the least electronegative substituent (OBn) displays the lowest selectivity; it seems the decreasing trend of the regioselectivity (*β* : *α* ratio) is aligned with the decreasing electron-withdrawing nature of the pendent substituent.

^13^C NMR analysis has been used as a convenient and effective tool to define the electronic nature of different substituents and their respective inductive effects on alkynes.^19^ To gain more insights into the electronic effect of these substituents, we collected the ^13^C NMR data of these alkynes, and the chemical shifts of both C_α_ and C_β_ atoms of the C–C triple bonds are summarized (Fig. 5c, columns 4 and 5). From the variations of the chemical shifts, we can see the pendent substituent has a reversed influence on the electron density at the C_α_ and C_β_ sites; it causes electron accumulation at the C_α_ site and electron attenuation at the C_β_ site. The nucleophilic boryl unit in the [Cu]-Bpin species prefers to attach the terminal C_β_ site of the alkyne, and electron attenuation at this site has a favorable influence for achieving a high β-selective borylcupration; meanwhile, an effect causing electron accumulation at the C_α_ site is deleterious for the α-selectivity, thus also being beneficial for the β-selectivity. Among these substituents examined, OTs caused both the strongest electron attenuation at the C_β_ site and the strongest electron accumulation at the C_α_ site, thus showing the highest β-selectivity. Moreover, the ^13^C NMR chemical shift difference between the C_β_ and C_α_ sites (Δ(*δ*C_β_ − *δ*C_α_)) was used to estimate the electronic inductive effect of a substituent on the C–C triple bond in the literature;^19*a*^ in this work, we also observed a good correlation between the chemical differences (Δ(*δ*C_β_ − *δ*C_α_)) and the regioselectivity (*β* : *α* ratio). These results together support the view that the OTs induces β-selectivity through its electronic properties.

## Conclusion

In summary, we developed a unified catalytic strategy for the synthesis of highly strained MCBs and spiro-MCBs, which relies on a copper-catalyzed highly regioselective borylative cyclization of aliphatic alkynes and versatile transformations of the newly installed boromethylene unit. This strategy enables the installation of functionalities at each site on the MCB skeleton with defined stereochemistry and greatly expands the existing chemical space of MCBs and spiro-MCBs. The concise and divergent total syntheses of four cyclobutane-containing natural products were achieved in 2–4 steps from a common BMCB precursor obtained by this strategy. Preliminary mechanistic studies revealed that both the NHC ligand (IMes) for the copper catalyst and the tethered leaving group (–OTs) are crucial for the observed high β-regioselectivity in the borylcupration process of alkynes and that the ligand also plays a key role in promoting the strained ring-closure of the vinyl copper intermediate.

## Data availability

Data for all of the new compounds in this manuscript are available in the ESI,† which includes experimental details, characterisation, copies of ^1^H and ^13^C NMR spectra.

## Author contributions

H. Z. performed the experiments with the aid of Y. L. and M. J. B. S. and H. Z. conceived the project. B. S. wrote the manuscript with the aid of H. Z., oversaw the research, and supervised the co-authors. All authors have given approval to the final version of the manuscript.

## Conflicts of interest

There are no conflicts to declare.

## Supplementary Material

SC-014-D3SC01103H-s001
