# Supplementary material for: A general catalytic synthetic strategy for highly strained methylenecyclobutanes and spiromethylenecyclobutanes
Source: Chem Sci. 2023 Jun 9;14(29):7897–904. doi: 10.1039/d3sc01103h (PMC10370550; doi:10.1039/d3sc01103h)
Supplement: SC-014-D3SC01103H-s001 [file SC-014-D3SC01103H-s001.pdf]

Electronic Supplementary Material (ESI) for Chemical Science.
This journal is © The Royal Society of Chemistry 2023

A General Catalytic Synthetic Strategy for Highly Strained Methylenecyclobutanes and Spiromethylenecyclobutanes

Haotian Zhao, Yu Lin, Mingyu Jiang, and Bo Su*

State Key Laboratory of Medical Chemical Biology, College of Pharmacy, Nankai University, Tianjin, 300350 (P. R. China)

E-mail: subo@nankai.edu.cn

Supporting Information

Table of Contents

1. General information 1
2. Preparation of the alkyne substrates 2
3. Condition Optimization 24
4. General procedure for the borylative cyclization of alkynes 24
5. Transformations of the BMCBs 45
6. Concise total synthesis of cyclobutane-containing natural products 53
7. Mechanism Studies 57
8. References 58
9. NMR Spectra 59

1. General information

Chemicals were purchased and used as received unless otherwise noted. Solvents were purchased from commercial suppliers, dried using standard methods, and further stored over activated 4A molecular sieves in an N_2-filled glovebox. The borylative cyclization reactions were set up in an N_2-filled glovebox using oven-dried glassware and were stirred with Teflon-coated magnetic stirring bars at specific temperature outside the glovebox. Reaction temperatures above room temperature refer to temperatures of an aluminum heating block, which was controlled by an electronic temperature modulator. Flash chromatography was performed using 200-300 mesh silica gel with the indicated eluent according to standard techniques. Analytical thin-layer chromatography (TLC) was performed on pre-coated, glass-backed silica gel plates. Visualization of the developed chromatogram was performed by UV absorbance (254 nm) or commonly used spray reagents ($KMnO_4$ solution, phosphomolybdic acid solution or iodine vapor). ^1H and ^{13}C NMR spectra were recorded on Bruker 400 MHz spectrometers with ^{13}C operating frequencies of 100 MHz, and in the ^{13}C NMR spectra of the borylated products, the carbon connected with boron was not observed. ^{11}B NMR spectra were recorded on Bruker 400 MHz spectrometers with a ^{11}B operating frequency of 126 MHz. ^{19}F NMR spectra were recorded on Bruker 400 MHz spectrometers with a ^{19}F operating frequency of 376 MHz. Chemical shifts are reported in ppm relative to the residual solvent signal [$CDCl_3$: δ 7.26, $(CD_3)_2SO$: δ 2.50 for ^1H-NMR, and $CDCl_3$: δ 77.16, $(CD_3)_2SO$: δ 39.52 for ^{13}C-NMR]. HRMS were recorded on an Ion Spec FT-ICR mass spectrometer with ESI resource and Ion trap mass analyzer.

2. Preparation of the alkyne substrates

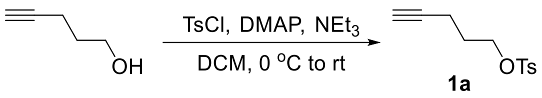

Pent-4-yn-1-yl 4-methylbenzenesulfonate (1a):

Procedure A: To a solution of 4-pentyn-1-ol (0.841 g, 10.0 mmol), NEt_3 (2.5 mL, 18 mmol), and DMAP (0.12 g, 1.0 mmol) in DCM (15 mL) was added TsCl (2.80 g, 15.0 mmol) in three portions at 0 °C. The reaction mixture was warmed to room temperature, and the reaction was stirred for 6 h (the reaction was generally monitored by TLC till the alcohol was fully consumed). The reaction was quenched with saturated aqueous NH_4Cl solution (15 mL), and the resulting mixture was extracted with DCM (10 mL * 3). The combined organic layers were washed with saturated aqueous NH_4Cl solution (5 mL * 2) and brine, dried over Na_2SO_4, filtered, and concentrated *in vacuo*. The residue was purified by flash column chromatography on silica gel (hexane : EtOAc 100:0 to 70:30) to give compound **1a** (75%, 1.78 g) as a colorless oil. ^1H NMR (400 MHz, $CDCl_3$) δ 7.79 (d, J = 8.0 Hz, 2H), 7.34 (d, J = 8.0 Hz, 2H), 4.14 (t, J = 6.0 Hz, 2H), 2.44 (s, 3H), 2.25 (td, J = 6.8, 2.4 Hz, 2H), 1.91–1.76 (m, 3H). The ^1H NMR data matched with those reported in the literature.[1]

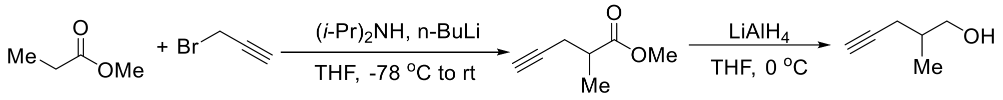

Synthesis of the 2-substituted pent-4-yn-1-ol.

Procedure B: To a solution of diisopropylamine (1.8 mL, 13 mmol) in THF (15 mL) was added *n*-BuLi (2.5 M in hexane, 4.8 mL, 12 mmol) dropwise at 0 °C under Ar. The reaction mixture was stirred at 0 °C for 30 min and was cooled to -78 °C, followed by the addition of a solution of methyl propionate (1.02 g, 10.0 mmol). The resulting solution was stirred for 1 h at -78 °C, and 3-bromopropyne (1.2 mL, 15 mmol) was added dropwise via a syringe. The reaction mixture was allowed to warm to room temperature till the ester was fully consumed, which was monitored by TLC. The reaction was quenched with saturated aqueous NH_4Cl solution (30 mL), and the resulting mixture was evaporated to remove the volatiles under reduced pressure. The residue was extracted with ethyl acetate (20 mL * 3). The combined organic layers were washed with saturated aqueous

NH_4Cl solution (10 mL * 2) and brine, dried over Na_2SO_4, filtered, and concentrated *in vacuo*. The residue was purified by flash column chromatography on silica gel (hexane : EtOAc 100:0 to 50:50) to give the methyl 2-methylpent-4-ynoate (42%, 0.53 g) as a colorless oil. To a solution of lithium aluminum hydride (0.24 g, 6.3 mmol) in THF (15 mL) was added a solution of 2-methylpent-4-ynoate (0.53 g, 4.2 mmol) in THF dropwise at 0 °C. The reaction mixture was stirred at room temperature and monitored by TLC. The reaction was quenched with water dropwise, followed by the addition of $MgSO_4$ (5 g). The mixture was filtered, and the volatiles were removed by evaporation under reduced pressure to give the 2-methylpent-4-yn-ol (98%, 0.40 g) as a colorless oil, which was used directly in the next step without further purification.

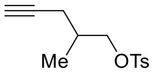

1b

2-Methylpent-4-yn-1-yl 4-methylbenzenesulfonate (1b):

The general procedure A was followed with the corresponding alcohol (0.40 g, 4.1 mmol) and TsCl (1.18 g, 6.20 mmol). The crude product was purified by flash column chromatography on silica gel to give compound **1b** (74%, 0.76 g) as a colorless oil. ^1H NMR (400 MHz, $CDCl_3$) δ 7.79 (d, J = 7.6 Hz, 2H), 7.35 (d, J = 7.6 Hz, 2H), 3.95 (d, J = 5.6 Hz, 2H), 2.45 (s, 3H), 2.25–2,14 (m, 2H), 2.10–1.95 (m, 1H), 1.89 (s, 1H), 0.99 (d, J = 6.4 Hz, 3H). The ^1H NMR data matched with those reported in the literature.[1]

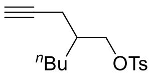

1c

2-(Prop-2-yn-1-yl)hexyl 4-methylbenzenesulfonate (1c):

The general procedure A was followed with the corresponding alcohol (0.83 g, 5.9 mmol) and TsCl (1.68 g, 8.85 mmol). The crude product was purified by flash column chromatography on silica gel (hexane : EtOAc 100:0 to 70:30) to give compound **1c** (61%, 1.06 g) as a colorless oil. ^1H NMR (400 MHz, $CDCl_3$) δ 7.79 (d, J = 7.6 Hz, 2H), 7.34 (d, J = 7.6 Hz, 2H), 4.01 (dd, J = 15.6, 5.4 Hz, 2H), 2.45 (s, 3H), 2.33–2.12 (m, 2H), 1.92–1.75 (m, 2H), 1.46–1.06 (m, 6H), 0.85 (t, J = 6.4 Hz, 3H). ^{13}C NMR (100 MHz, $CDCl_3$) δ 144.8, 132.9, 129.8, 128.0, 80.9, 71.6, 70.1, 36.9, 29.3, 28.7, 22.6, 21.6, 19.9, 13.9. HRMS (ESI+) calcd for $C_{16}H_{23}O_3S$ $[M+H]^+$ 295.1362, found 295.1357.

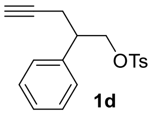

1d

2-Phenylpent-4-yn-1-yl 4-methylbenzenesulfonate (1d):

The general procedure A was followed with the corresponding alcohol (0.8 g, 5 mmol) and TsCl (1.4 g, 7.5 mmol). The crude product was purified by flash column chromatography on silica gel (hexane : EtOAc 100:0 to 70:30) to give compound **1d** (86%, 1.35g) as a white solid. ^{1}H NMR (400 MHz, $CDCl_3$) δ 7.69 (d, J = 7.6 Hz, 2H), 7.39–7.24 (m, 5H), 7.15 (d, J = 7.2 Hz, 2H), 4.21–4.30 (m, 2H), 3.30–3.04 (m, 1H), 2.52–2.65 (m, 2H), 2.44 (s, 3H), 1.90 (s, 1H). ^{13}C NMR (100 MHz, $CDCl_3$) δ 144.9, 139.0, 132.7, 129.9, 128.7, 128.0, 127.7, 127.6, 80.9, 72.0, 70.8, 43.5, 21.8, 21.8. HRMS (ESI+) calcd for $C_{18}H_{19}O_3S$ $[M+H]^+$ 315.1049, found 315.1047.

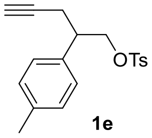

1e

2-(P-tolyl)pent-4-yn-1-yl 4-methylbenzenesulfonate (1e):

The general procedure A was followed with the corresponding alcohol (0.64 g, 3.7 mmol) and TsCl (1.05 g, 5.50 mmol). The crude product was purified by flash column chromatography on silica gel (hexane : EtOAc 100:0 to 70:30) to give compound **1e** (66%, 0.8 g) as a white solid. ^{1}H NMR (400 MHz, $CDCl_3$) δ 7.69 (d, J = 8.0 Hz, 2H), 7.28 (d, J = 8.0 Hz, 2H), 7.08 (d, J = 8.0 Hz, 2H), 7.03 (d, J = 8.0 Hz, 2H), 4.30–4.17 (m, 2H), 3.12 (p, J = 6.8 Hz, 1H), 2.58 (dd, J = 6.4, 2.2 Hz, 1H), 2.54 (dd, J = 7.1, 2.0 Hz, 1H), 2.44 (s, 3H), 2.32 (s, 3H), 1.89 (s, 1H). ^{13}C NMR (100 MHz, $CDCl_3$) δ 144.8, 137.3, 135.9, 132.8, 129.9, 129.4, 128.0, 127.6, 81.1, 72.2, 70.7, 43.1, 21.9, 21.8, 21.2. HRMS (ESI+) calcd for $C_{19}H_{21}O_3S$ $[M+H]^+$ 329.1206, found 329.1201.

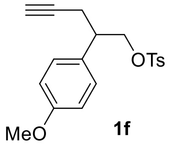

1f

2-(4-Methoxyphenyl)pent-4-yn-1-yl 4-methylbenzenesulfonate (1f):

The general procedure A was followed with the corresponding alcohol (1.3 g, 6.7 mmol) and TsCl (1.91 g, 10.1 mmol). The crude product was purified by flash column chromatography on silica gel

(hexane : EtOAc 100:0 to 70:30) to give compound **1f** (51%, 1.16 g) as a white solid. ^{1}H NMR (400 MHz, $CDCl_3$) δ 7.69 (d, J = 8.0 Hz, 2H), 7.29 (d, J = 8.0 Hz, 2H), 7.07 (d, J = 8.2 Hz, 2H), 6.80 (d, J = 8.2 Hz, 2H), 4.28–4.15 (m, 2H), 3.78 (s, 3H), 3.11 (p, J = 6.4 Hz, 1H), 2.59 (dd, J = 16.8, 6.4 Hz, 1H), 2.51 (dd, J = 16.8, 7.2 Hz, 1H), 2.43 (s, 3H), 1.90 (s, 1H). ^{13}C NMR (100 MHz, $CDCl_3$) δ 159.0, 144.8, 132.8, 131.0, 130.0, 128.7, 128.0, 114.1, 81.1, 72.2, 70.8, 55.3, 42.7, 21.9, 21.7. HRMS (ESI+) calcd for $C_{19}H_{20}NaO_4S$ [M+Na]$^+$ 367.0975, found 367.0969.

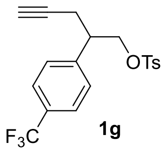

1g

2-(4-(Trifluoromethyl)phenyl)pent-4-yn-1-yl 4-methylbenzenesulfonate (1g):

The general procedure A was followed with the corresponding alcohol (0.60 g, 2.6 mmol) and TsCl (0.75 g, 4.0 mmol). The crude product was purified by flash column chromatography on silica gel (hexane : EtOAc 100:0 to 70:30) to give compound **1g** (54%, 547 mg) as a white solid. ^{1}H NMR (400 MHz, $CDCl_3$) δ 7.64 (d, J = 7.6 Hz, 2H), 7.51 (d, J = 7.6 Hz, 2H), 4.43–4.14 (m, 2H), 3.23 (t, J = 6.4 Hz, 1H), 2.59 (t, J = 6.8 Hz, 2H), 1.94 (s, 1H). ^{19}F NMR (376 MHz, $CDCl_3$) δ -62.6. HRMS (ESI+) calcd for $C_{19}H_{18}F_3O_3S$ [M+H]$^+$ 383.0923, found 383.0919.

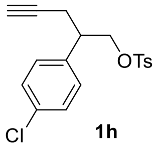

1h

2-(4-Chlorophenyl)pent-4-yn-1-yl 4-methylbenzenesulfonate (1h):

The general procedure A was followed with the corresponding alcohol (1.7 g, 8.3 mmol) and TsCl (2.50 g, 13.1 mmol). The crude product was purified by flash column chromatography on silica gel (hexane : EtOAc 100:0 to 70:30) to give compound **1h** (62%, 1.89 g) as a white solid. ^{1}H NMR (400 MHz, $CDCl_3$) δ 7.65 (d, J = 8.4 Hz, 2H), 7.28 (d, J = 8.0 Hz, 2H), 7.22 (d, J = 8.4 Hz, 2H), 7.06 (d, J = 8.4 Hz, 2H), 4.28–4.17 (m, 2H), 3.14 (p, J = 6.4 Hz, 1H), 2.61–2.47 (m, 2H), 2.44 (s, 3H), 1.92 (t, J = 2.4 Hz, 1H). ^{13}C NMR (100 MHz, $CDCl_3$) δ 145.0, 137.5, 133.4, 132.6, 129.9, 129.1, 128.8, 128.0, 80.5, 71.7, 71.1, 43.0, 21.7, 21.7. HRMS (ESI+) calcd for $C_{18}H_{18}ClO_3S$ [M+H]$^+$ 349.0660, found 349.0656.

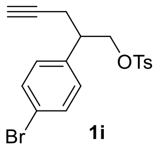

1i

2-(4-Bromophenyl)pent-4-yn-1-yl 4-methylbenzenesulfonate (1i):

The general procedure A was followed with the corresponding alcohol (0.71g g, 3.0 mmol) and TsCl (0.86 g, 4.5 mmol). The crude product was purified by flash column chromatography on silica gel (hexane : EtOAc 100:0 to 70:30) to give compound **1i** (67%, 786 mg) as a white solid. ^{1}H NMR (400 MHz, $CDCl_3$) δ 7.64 (d, J = 8.0 Hz, 2H), 7.37 (d, J = 8.4 Hz, 2H), 7.28 (d, J = 8.0 Hz, 2H), 7.00 (d, J = 8.0 Hz, 2H), 4.31–4.15 (m, 2H), 3.12 (p, J = 6.4 Hz, 1H), 2.63–2.46 (m, 2H), 2.45 (s, 3H), 1.92 (s, 1H). ^{13}C NMR (100 MHz, $CDCl_3$) δ 145.0, 138.0, 132.6, 131.8, 129.9, 129.4, 128.0, 121.6, 80.5, 71.7, 71.1, 43.0, 21.8, 21.6. HRMS (ESI+) calcd for $C_{18}H_{18}BrO_3S$ $[M+H]^+$ 393.0155, found 393.0150.

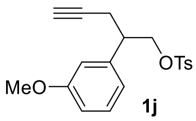

1j

2-(3-Methoxyphenyl)pent-4-yn-1-yl 4-methylbenzenesulfonate (1j):

The general procedure A was followed with the corresponding alcohol (2.40 g, 12.6 mmol) and TsCl (3.59 g, 18.9 mmol). The crude product was purified by flash column chromatography on silica gel (hexane : EtOAc 100:0 to 70:30) to give compound **1j** (59%, 2.54 g) as a white solid. ^{1}H NMR (400 MHz, $CDCl_3$) δ 7.68 (d, J = 7.6 Hz, 2H), 7.28 (d, J = 7.6 Hz, 2H), 7.19 (t, J = 8.0 Hz, 1H), 6.78 (d, J = 8.4 Hz, 1H), 6.73 (d, J = 7.6 Hz, 1H), 6.68 (s, 1H), 4.31–4.19 (m, 2H), 3.76 (s, 3H), 3.13 (p, J = 6.4 Hz, 1H), 2.50–2.63 (m, 7.2 Hz, 2H), 2.43 (s, 3H), 1.92 (s, 1H). ^{13}C NMR (100 MHz, $CDCl_3$) δ 159.8, 144.9, 140.6, 132.7, 129.9, 129.7, 128.0, 120.0, 113.6, 112.8, 80.9, 72.0, 70.9, 55.2, 43.5, 21.8, 21.7. HRMS (ESI+) calcd for $C_{19}H_{20}NaO_4S$ $[M+Na]^+$ 367.0975, found 367.0970.

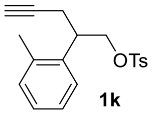

1k

2-(O-tolyl)pent-4-yn-1-yl 4-methylbenzenesulfonate (1k):

The general procedure A was followed with the corresponding alcohol (0.89 g, 5.1 mmol) and TsCl (1.5 g, 7.6 mmol). The crude product was purified by flash column chromatography on silica gel

(hexane : EtOAc 100:0 to 70:30) to give compound **1k** (74%, 1.23 g) as a white solid. ^{1}H NMR (400 MHz, CDCl$_3$) δ 7.69 (d, J = 7.2 Hz, 2H), 7.29 (d, J = 7.6 Hz, 2H), 7.17–7.05 (m, 4H), 4.30–4.13 (m, 2H), 3.48 (p, J = 6.4 Hz, 1H), 2.61 (dd, J = 16.8, 6.3 Hz, 1H), 2.52 (dd, J = 16.8, 7.2 Hz, 1H), 2.44 (s, 3H), 2.28 (s, 3H), 1.88 (s, 1H). ^{13}C NMR (100 MHz, CDCl$_3$) δ 144.9, 137.1, 136.3, 132.7, 130.7, 129.9, 128.0, 127.3, 126.3, 126.0, 81.0, 71.7, 70.5, 38.6, 21.7, 21.4, 19.5. HRMS (ESI+) calcd for $C_{19}H_{20}NaO_3S$ [M+Na]$^+$ 351.1025, found 351.1021.

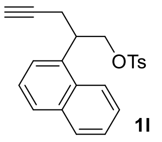

1l

2-(Naphthalen-1-yl)pent-4-yn-1-yl 4-methylbenzenesulfonate (1l):

The general procedure A was followed with the corresponding alcohol (1.8 g, 8.6 mmol) and TsCl (2.45 g, 12.9 mmol). The crude product was purified by flash column chromatography on silica gel (hexane : EtOAc 100:0 to 70:30) to give compound **1l** (59%, 1.85 g) as a white solid. ^{1}H NMR (400 MHz, CDCl$_3$) δ 7.95–7.82 (m, 2H), 7.76 (d, J = 8.0 Hz, 1H), 7.63 (d, J = 7.6 Hz, 2H), 7.52–7.46 (m, 2H), 7.40 (t, J = 7.6 Hz, 1H), 7.33 (d, J = 7.2 Hz, 1H), 7.20 (d, J = 7.6 Hz, 2H), 4.49–4.29 (m, 2H), 4.06 (p, J = 6.4 Hz, 1H), 2.77 (q, J = 10.4, 8.4 Hz, 2H), 2.39 (s, 3H), 1.91 (s, 1H). ^{13}C NMR (100 MHz, CDCl$_3$) δ 144.8, 134.3, 134.0, 132.6, 131.4, 129.8, 129.2, 128.2, 127.9, 126.6, 125.77, 125.4, 124.2, 122.3, 81.0, 71.5, 71.0, 37.8, 21.7, 21.3. HRMS (ESI+) calcd for $C_{22}H_{20}NaO_3S$ [M+Na]$^+$ 387.1025, found 387.1022.

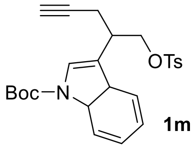

1m

Tert-butyl 3-(1-(tosyloxy)pent-4-yn-2-yl)-3a,7a-dihydro-1H-indole-1-carboxylate (1m):

The general procedure A was followed with the corresponding alcohol (1.06 g, 3.60 mmol) and TsCl (1.01 g, 5.33 mmol). The crude product was purified by flash column chromatography on silica gel (hexane : EtOAc 100:0 to 70:30) to give the product **1m** (53%, 0.66 g) as a colorless oil. ^{1}H NMR (400 MHz, CDCl$_3$) δ 8.08 (d, J = 7.6 Hz, 1H), 7.65 (d, J = 8.4 Hz, 2H), 7.46 (s, 1H), 7.36 (d, J = 8.0 Hz, 1H), 7.30 (t, J = 7.2 Hz, 1H), 7.18 (dt, J = 7.2, 3.2 Hz, 3H), 4.38 (dd, J = 9.6, 5.2 Hz, 1H),

4.30 (dd, J = 9.6, 7.2 Hz, 1H), 3.43 (p, J = 6.4 Hz, 1H), 2.69 (dd, J = 6.4, 2.4 Hz, 2H), 2.39 (s, 3H), 1.95 (t, J = 2.4 Hz, 1H), 1.68 (s, 9H).

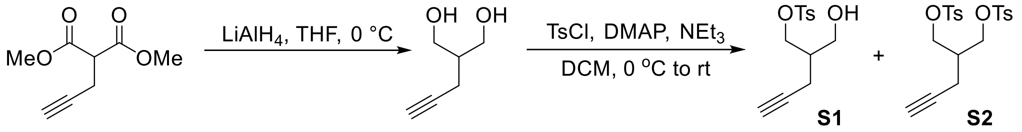

To a solution of lithium aluminum hydride (3.00 g, 79.4 mmol) in THF (80 mL) was added a solution of dimethyl 2-(prop-2-yn-1-yl)malonate (4.50 g, 26.5 mmol) in THF dropwise at 0 °C. After stirred for 3 h, the reaction mixture was quenched with water dropwise, followed by the addition of $MgSO_4$ (20 g). The resulting mixture was filtered, and the volatiles were removed by evaporation under reduced pressure to give the 2-(prop-2-yn-1-yl)propane-1,3-diol (64%, 1.93 g) as a colorless oil, which was used directly in the next step without further purification. To a solution of 2-(prop-2-yn-1-yl)propane-1,3-diol (1.93 g, 17.0 mmol), NEt_3 (2.30 mL, 17.0 mmol), and DMAP (0.21 g, 1.7 mmol) in DCM (15 mL) was added p-toluenesulfonyl chloride (2.58 g, 13.6 mmol) in three portions at 0 °C. The reaction mixture was allowed to warm to room temperature and stirred for 5 h. The reaction was quenched with saturated aqueous NH_4Cl solution (20 mL), and the reaction mixture was extracted with DCM (10 mL * 3). The combined organic layers were washed with saturated aqueous NH_4Cl solution (5 mL * 2) and brine, dried over Na_2SO_4, filtered, and concentrated *in vacuo*. The residue was purified by flash column chromatography on silica gel (hexane : EtOAc 100:0 to 70:30) to give compounds **S1** (25%, 1.15 g) and **S2** (25%, 1.79 g).

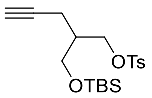

1n

2-(((Tert-butyldimethylsilyl)oxy)methyl)pent-4-yn-1-yl 4-methylbenzenesulfonate (1n):

To a solution of the alcohol **S1** (0.77 g, 2.8 mmol), DMAP (37 mg, 0.30 mmol), and NEt_3 (0.50 mL, 3.4 mmol) in DCM (10 mL) was added TBSCl (0.51 g, 3.4 mmol) at 0 °C. The reaction mixture was allowed to warm to room temperature and stirred for 4 h. The reaction was quenched with saturated aqueous NH_4Cl solution (5 mL), and the reaction mixture was extracted with DCM (5 mL * 3). The combined organic layers were washed with saturated aqueous NH_4Cl solution (5 mL * 2)

and brine, dried over Na_2SO_4, filtered, and concentrated *in vacuo*. The residue was purified by flash column chromatography on silica gel (hexane : EtOAc 100:0 to 70:30) to give compound **1n** (80%, 855 mg) as a colorless oil. ^{1}H NMR (400 MHz, $CDCl_3$) δ 7.79 (d, J = 8.0Hz, 2H), 7.34 (d, J = 8.0 Hz, 2H), 4.08 (p, J = 5.6 Hz, 2H), 3.67–3.52 (m, 2H), 2.44 (s, 3H), 2.26–2.20 (m, 2H), 2.04 (dt, J = 12.0, 6.0 Hz, 1H), 1.89 (s, 1H), 0.82 (s, 9H), 0.00 (s, 6H). ^{13}C NMR (100 MHz, $CDCl_3$) δ 144.9, 132.8, 130.0, 128.1, 81.1, 70.2, 69.6, 61.3, 40.1, 25.9, 21.8, 18.3, 17.2, -5.5. HRMS (ESI+) calcd for $C_{19}H_{31}O_4SSi$ $[M+H]^+$ 383.1707, found 383.1702.

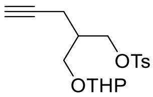

1o

2-(((Tetrahydro-2H-pyran-2-yl)oxy)methyl)pent-4-yn-1-yl 4-methylbenzenesulfonate (1o):

To a solution of the alcohol **S1** (0.40 g, 1.5 mmol), and p-toluenesulfonic acid (3 mg, 1 mol%) in DCM (5 mL) was added 3,4-dihydro-2H-pyran (378 mg, 4.5 mmol) at 0 °C. The reaction mixture was allowed to warm to room temperature and stirred for 3 h. The reaction was quenched with saturated aqueous NH_4Cl solution (10 mL), and the reaction mixture was extracted with DCM (5 mL * 3). The combined organic layers were washed with saturated aqueous NH_4Cl solution (5 mL * 2) and brine, dried over Na_2SO_4, filtered, and concentrated *in vacuo*. The residue was purified by flash column chromatography on silica gel (hexane : EtOAc 100:0 to 70:30) to give compound **1o** (76%, 400 mg) as a colorless oil. ^{1}H NMR (400 MHz, $CDCl_3$) δ 7.80 (d, J = 8.0 Hz, 2H), 7.34 (d, J = 8.0 Hz, 2H), 4.49 (s, 1H), 4.24–4.04 (m, 2H), 3.79–3.69 (m, 2H), 3.52–3.27 (m, 2H), 2.44 (s, 3H), 2.29 (s, 2H), 2.18 (s, 1H), 1.90 (s, 1H), 1.74–1.45 (m, 6H). ^{13}C NMR (100 MHz, $CDCl_3$) δ144.9, 132.9, 130.0, 128.1, 99.1, 98.8, 80.9, 80.8, 70.4, 69.6, 65.9, 65.7, 62.2, 62.1, 38.1, 38.1, 30.5, 30.5, 25.5, 21.8, 19.4, 19.3, 17.7, 17.6. HRMS (ESI+) calcd for $C_{18}H_{24}NaO_5S$ $[M+Na]^+$ 375.1237, found 375.1234.

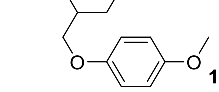

1p

2-((4-Methoxyphenoxy)methyl)pent-4-yn-1-yl 4-methylbenzenesulfonate (1p):

To a solution of **S2** (1.0 g, 2.4 mmol) and K_2CO_3 (331 mg, 2.40 mmol) in acetonitrile (5 mL) was added 4-methoxyphenol (238 mg, 1.90 mmol) at room temperature. The reaction mixture was stirred

at 70 °C for 4 h. The reaction was quenched with saturated aqueous NH_4Cl solution (10 mL), and the mixture was extracted with EtOAc (5 mL * 3). The combined organic layers were washed with saturated aqueous NH_4Cl solution (5 mL * 2) and brine, dried over Na_2SO_4, filtered, and concentrated *in vacuo*. The residue was purified by flash column chromatography on silica gel (hexane : EtOAc 100:0 to 70:30) to give compound **1p** (30%, 215 mg) as a white solid. ^{1}H NMR (400 MHz, $CDCl_3$) δ 7.76 (d, J = 8.4 Hz, 2H), 7.27 (d, J = 8.4 Hz, 2H), 6.80 (d, J = 9.2 Hz, 2H), 6.71 (d, J = 9.2 Hz, 2H), 4.30–4.14 (m, 2H), 3.94–3.82 (m, 2H), 3.77 (s, 3H), 2.40 (s, 3H), 2.39–2.30 (m, 3H), 1.94 (t, J = 2.4 Hz, 1H). ^{13}C NMR (100 MHz, $CDCl_3$) δ 154.2, 152.6, 145.0, 132.6, 130.0, 128.0, 115.5, 114.7, 80.5, 70.7, 69.2, 66.7, 55.8, 37.9, 21.7, 17.6. HRMS (ESI+) calcd for $C_{20}H_{22}NaO_5S$ $[M+Na]^+$ 397.1080, found 397.1077.

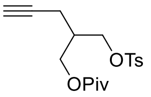

1q

2-((Tosyloxy)methyl)pent-4-yn-1-yl pivalate (1q):

To a solution of the alcohol **S1** (536 mg, 2.00 mmol), DMAP (25 mg, 0.20 mmol), and NEt_3 (0.60 mL, 3.0 mmol) in DCM (5 mL) was added pivaloyl chloride (361 mg, 3.00 mmol) at 0 °C. The reaction mixture was allowed to warm to room temperature and stirred for 3 h. The reaction was quenched with saturated aqueous NH_4Cl solution (10 mL), and the reaction mixture was extracted with DCM (5 mL * 3). The combined organic layers were washed with saturated aqueous NH_4Cl solution (5 mL * 2) and brine, dried over Na_2SO_4, filtered, and concentrated *in vacuo*. The residue was purified by flash column chromatography on silica gel (hexane : EtOAc 100:0 to 70:30) to give compound **1q** (60%, 422 mg) as a colorless oil. ^{1}H NMR (400 MHz, $CDCl_3$) δ 7.79 (d, J = 8.0 Hz, 2H), 7.35 (d, J = 8.0 Hz, 2H), 4.17–3.99 (m, 4H), 2.45 (s, 3H), 2.34–2.23 (m, 3H), 1.93 (s, 1H), 1.14 (s, 9H). ^{13}C NMR (100 MHz, $CDCl_3$) δ 178.1, 145.1, 132.7, 130.1, 128.1, 79.9, 71.0, 68.8, 62.7, 38.9, 37.2, 27.2, 21.8, 17.7. HRMS (ESI+) calcd for $C_{18}H_{25}O_5S$ $[M+H]^+$ 353.1417, found 353.1413.

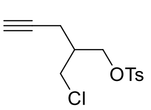

1r

2-(Chloromethyl)pent-4-yn-1-yl 4-methylbenzenesulfonate (1r):

To a solution of the alcohol **S1** (536 mg, 2.00 mmol), and PPh_3 (787 mg, 3.00 mmol) in DCM (5 mL) was added carbon tetrachloride (461 mg, 3.00 mmol) at 0 °C. The reaction mixture was allowed to warm to room temperature and stirred overnight. The reaction was quenched with saturated aqueous NH_4Cl solution (10 mL), and the reaction mixture was extracted with DCM (5 mL * 3). The combined organic layers were washed with saturated aqueous NH_4Cl solution (5 mL * 2) and brine, dried over Na_2SO_4, filtered, and concentrated *in vacuo*. The residue was purified by flash column chromatography on silica gel (hexane : EtOAc 100:0 to 70:30) to give compound **1r** (52%, 300 mg) as a white solid. ^{1}H NMR (400 MHz, $CDCl_3$) δ 7.80 (d, J = 8.0 Hz, 2H), 7.36 (d, J = 8.0 Hz, 2H), 4.13 (dt, J = 9.6, 5.6 Hz, 2H), 3.61 (d, J = 5.2 Hz, 2H), 2.46 (s, 3H), 2.37–2.25 (m, 3H), 1.97 (s, 1H). ^{13}C NMR (100 MHz, $CDCl_3$) δ 145.2, 132.6, 130.1, 128.1, 79.7, 71.1, 69.0, 43.6, 39.7, 21.8, 18.4. HRMS (ESI+) calcd for $C_{13}H_{16}ClO_3S$ $[M+H]^+$ 287.0503, found 287.0500.

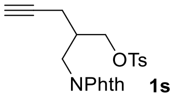

1s

2-((1,3-dioxoisoindolin-2-yl)methyl)pent-4-yn-1-yl 4-methylbenzenesulfonate (1s):

To a solution of **S2** (1.0 g, 2.4 mmol) in DMF (5 mL) was added phthalimide potassium salt (351 mg, 1.90 mmol) at room temperature. The reaction mixture was allowed to warm to 80 °C and stirred for 4 h. The reaction was quenched with saturated aqueous NH_4Cl solution (15 mL), and the reaction mixture was extracted with EtOAc (5 mL * 3), the combined organic layers were washed with saturated aqueous NH_4Cl solution (5 mL * 2) and brine, dried over Na_2SO_4, filtered, and concentrated *in vacuo*. The residue was purified by flash column chromatography on silica gel (hexane : EtOAc 100:0 to 70:30) to give compound **1s** (29%, 272 mg) as a white solid. ^{1}H NMR (400 MHz, $CDCl_3$) δ 7.83 (dd, J = 5.2, 2.8 Hz, 2H), 7.76 (d, J = 8.0 Hz, 2H), 7.72 (dd, J = 5.2, 2.8 Hz, 2H), 7.31 (d, J = 8.0 Hz, 2H), 4.12 (d, J = 4.8 Hz, 2H), 3.71 (d, J = 7.2 Hz, 2H), 2.47–2.38 (m, 4H), 2.34–2.29 (m, 2H), 1.85 (t, J = 2.4 Hz, 1H). ^{13}C NMR (100 MHz, $CDCl_3$) δ 168.3, 145.0, 134.3, 132.6, 132.0, 130.0, 128.2, 123.5, 79.9, 71.0, 69.7, 38.7, 36.8, 21.8, 18.8. HRMS (ESI+) calcd for $C_{21}H_{20}NO_5S$ $[M+H]^+$ 398.1057, found 398.1052.

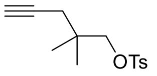

1t

2,2-Dimethylpent-4-yn-1-yl 4-methylbenzenesulfonate (1t):

The general procedure A was followed with the corresponding alcohol (0.31 g, 2.8 mmol) and TsCl (0.80 g, 4.2 mmol). The crude product was purified by flash column chromatography on silica gel (hexane : EtOAc 100:0 to 70:30) to give compound **1t** (60%, 452 mg) as a colorless oil. ^{1}H NMR (400 MHz, $CDCl_3$) δ 7.79 (d, J = 8.0 Hz, 2H), 7.34 (d, J = 8.0 Hz, 2H), 3.79 (s, 2H), 2.45 (s, 3H), 2.12 (d, J = 2.5 Hz, 2H), 1.88 (s, 1H), 0.97 (s, 6H). The ^{1}H NMR data matched with those reported in the literature.[2]

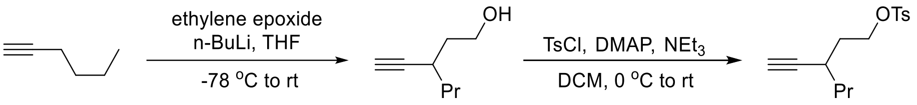

1u

3-Ethynylhexyl 4-methylbenzenesulfonate (1u):

Procedure C: To a solution of 1-hexyne (0.82 g, 10 mmol) in THF (15 mL) was added *n*-BuLi (2.5M in hexane, 9.6 mL, 24 mmol) dropwise over 5 min at -40 °C. After addition, the mixture was stirred at room temperature for 1.5 h and then at 40 °C for 2h. The reaction mixture was cooled to -40 °C, followed by the addition of a solution of ethylene epoxide in THF (1.0 M/L, 8 mL, 8 mmol). The resulting reaction mixture was allowed to warm to room temperature gradually and stirred overnight. The reaction was quenched with saturated aqueous NH_4Cl solution (30 mL), and the volatiles were evaporated under reduced pressure. The residue was extracted with ethyl acetate (20 mL * 3). The combined organic layers were washed with saturated aqueous NH_4Cl solution (10 mL * 2) and brine, dried over Na_2SO_4, filtered, and concentrated *in vacuo*. The residue was purified by flash column chromatography on silica gel (hexane : EtOAc 100:0 to 50:50) to give the 3-ethynylhexan-1-ol (48%, 0.54 g) as a colorless oil. To a solution of the alcohol (480 mg, 3.80 mmol), NEt_3 (0.95 mL, 6.8 mmol), and DMAP (49 mg, 0.40 mmol) in DCM (10 mL) was added p-toluenesulfonyl chloride (1.1 g, 5.1 mmol) in three portions at 0 °C. The reaction mixture was allowed to warm to room temperature and stirred for 5 h. The reaction was quenched with saturated aqueous NH_4Cl solution (10 mL), and the resulting mixture was extracted with DCM (5 mL * 3). The combined organic layers were washed with saturated aqueous NH_4Cl solution (5 mL * 2) and

brine, dried over Na_2SO_4, filtered, and concentrated *in vacuo*. The residue was purified by flash column chromatography on silica gel (hexane : EtOAc 100:0 to 70:30) to give compound **1u** (74%, 787 mg) as a colorless oil. ^{1}H NMR (400 MHz, $CDCl_3$) δ 7.80 (d, J = 8.4 Hz, 2H), 7.34 (d, J = 8.4 Hz, 2H), 4.26–4.14(m, 2H), 2.68–2.29 (m, 4H), 1.96 (d, J = 2.4 Hz, 1H), 1.89–1.78 (m, 1H), 1.73–1.62 (m, 1H), 1.54–1.44 (m, 1H), 1.42–1.30 (m, 3H), 0.88 (t, J = 6.8 Hz, 3H). ^{13}C NMR (100 MHz, $CDCl_3$) δ 144.8, 133.2, 129.9, 128.0, 85.7, 70.4, 68.6, 36.9, 34.1, 27.7, 21.7, 20.3, 13.8. HRMS (ESI+) calcd for $C_{15}H_{21}O_3S$ $[M+H]^+$ 281.1206, found 281.1202.

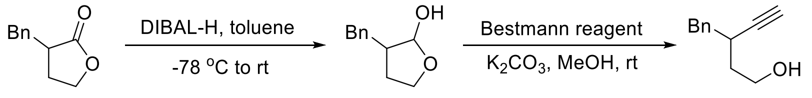

To a solution of 3-benzyldihydrofuran-2(3*H*)-one (0.94 g, 5.3 mmol) in toluene (10 mL) was added diisobutylaluminium hydride (1.0 M in toluene, 10.6 mL, 10.6 mmol) dropwise at -78 °C under Ar. After stirred for 2 h, the reaction was quenched with saturated aqueous potassium sodium tartrate solution (15 mL) slowly at -78 °C and stirred for 1 h at room temperature. The reaction mixture was extracted with ethyl acetate (10 mL * 3), and the combined organic layers were washed with saturated aqueous NH_4Cl solution (5 mL * 2) and brine, dried over Na_2SO_4, filtered, and concentrated *in vacuo*. The residue was purified by flash column chromatography on silica gel (hexane : EtOAc 100:0 to 70:30) to give the 3-benzyltetrahydrofuran-2-ol (26%, 250 mg) as a colorless oil. To a solution of the 3-benzyltetrahydrofuran-2-ol (250 mg, 1.40 mmol) and potassium carbonate (483 mg, 3.50 mmol) in MeOH (5 mL) was added the Bestmann reagent (403 mg, 2.10 mmol) at room temperature under Ar, and the resulting mixture was stirred for 6 h. The reaction was quenched with saturated aqueous NH_4Cl solution (5 mL), and the volatiles were evaporated under reduced pressure. The residue was extracted with ethyl acetate (10 mL * 3). The combined organic layers were washed with saturated aqueous NH_4Cl solution (5 mL * 2) and brine, dried over Na_2SO_4, filtered, and concentrated *in vacuo*. The residue was purified by flash column chromatography on silica gel (hexane : EtOAc 100:0 to 70:30) to give the 3-benzylpent-4-yn-1-ol (78%, 180 mg) as a colorless oil.

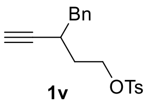

1v

3-Benzylpent-4-yn-1-yl 4-methylbenzenesulfonate (1v):

The general procedure A was followed with the corresponding alcohol (0.18 g, 1.0 mmol) and TsCl (0.29g, 1.5 mmol). The crude product was purified by flash column chromatography on silica gel (hexane : EtOAc 100:0 to 70:30) to give compound **1v** (54%, 177 mg) as a colorless oil. ^{1}H NMR (400 MHz, $CDCl_3$) δ 7.78 (d, J = 8.4 Hz, 2H), 7.33 (d, J = 8.4 Hz, 2H), 7.31–7.27 (m, 2H), 7.23 (t, J = 7.2 Hz, 1H), 7.17 (d, J = 6.8 Hz, 2H), 4.28–4.13 (m, 2H), 2.82–2.69 (m, 3H), 2.44 (s, 3H), 1.99 (d, J = 1.6 Hz, 1H), 1.94–1.83 (m, 1H), 1.74–1.60 (m, 1H). ^{13}C NMR (100 MHz, $CDCl_3$) δ 144.9, 138.4, 133.0, 129.9, 129.3, 128.4, 128.0, 126.7, 84.9, 71.5, 68.4, 40.9, 33.3, 29.8, 21.7. HRMS (ESI+) calcd for $C_{19}H_{20}NaO_3S$ $[M+Na]^+$ 351.1025, found 351.1024.

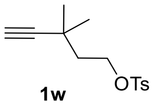

1w

3,3-Dimethylpent-4-yn-1-yl 4-methylbenzenesulfonate (1w):

The general procedure A was followed with the corresponding alcohol (36 mg, 0.30 mmol) and TsCl (86 mg, 0.45 mmol). The crude product was purified by flash column chromatography on silica gel (hexane : EtOAc 100:0 to 70:30) to give compound **1w** (45%, 36 mg) as a colorless oil. ^{1}H NMR (400 MHz, $CDCl_3$) δ 7.80 (d, J = 8.4 Hz, 2H), 7.35 (d, J = 8.0 Hz, 2H), 4.24 (t, J = 7.2 Hz, 2H), 2.45 (s, 3H), 2.05 (s, 1H), 1.79 (t, J = 7.2 Hz, 2H), 1.20 (s, 6H). ^{13}C NMR (100 MHz, $CDCl_3$) δ 144.9, 133.3, 130.0, 128.1, 89.7, 69.2, 68.3, 41.3, 29.7, 29.5, 21.8. HRMS (ESI+) calcd for $C_{14}H_{19}O_3S$ $[M+H]^+$ 267.1049, found 267.1051.

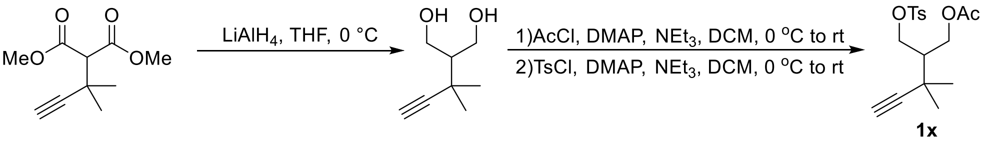

3,3-Dimethyl-2-((tosyloxy)methyl)pent-4-yn-1-yl acetate (1x):

The general procedure A was followed with the corresponding alcohol (0.14 g, 0.73 mmol) and TsCl (0.21 g, 1.1 mmol). The crude product was purified by flash column chromatography on silica gel (hexane : EtOAc 100:0 to 70:30) to give compound **1x** (71%, 0.18 g) as a colorless oil. ^{1}H NMR

(400 MHz, $CDCl_3$) δ 7.79 (d, J = 8.0 Hz, 2H), 7.34 (d, J = 8.0 Hz, 2H), 4.28 (td, J = 9.6, 4.4 Hz, 2H), 4.20 (dd, J = 10.0, 6.0 Hz, 1H), 4.11 (dd, J = 11.6, 6.8 Hz, 1H), 2.44 (s, 3H), 2.09 (s, 1H), 1.97–1.88 (m, 4H), 1.23 (s, 6H). ^{13}C NMR (100 MHz, $CDCl_3$) δ 170.7, 145.0, 133.0, 130.0, 128.1, 88.8, 70.0, 68.2, 61.7, 46.2, 32.3, 28.1, 27.7, 21.8, 20.9. HRMS (ESI+) calcd for $C_{17}H_{23}O_5S$ $[M+H]^+$ 339.1261, found 339.1265.

Synthesis of the 1-substituted pent-4-yn-1-ol and the corresponding tosylate.

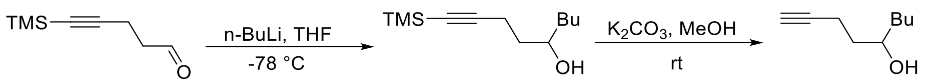

Procedure D: To a solution of the aldehyde (1.02 g, 6.70 mmol) in THF (15 mL) was added *n*-BuLi (2.5 M/L in hexane, 3.2 mL, 8.0 mmol) dropwise over 5 min at -78 °C. The reaction mixture was allowed to warm to room temperature gradually and stirred for 2 h. The reaction was quenched with saturated aqueous NH_4Cl solution (20 mL), and volatiles were evaporated under reduced pressure. The residue was extracted with ethyl acetate (10 mL * 3). The combined organic layers were washed saturated aqueous NH_4Cl solution (5 mL * 2) and with brine, dried over Na_2SO_4, filtered, and concentrated *in vacuo*. The residue was purified by flash column chromatography on silica gel (hexane : EtOAc 100:0 to 70:30) to give the 1-(trimethylsilyl)non-1-yn-5-ol (30%, 424 mg) as a colorless oil. To a solution of the 1-(trimethylsilyl)non-1-yn-5-ol (0.42 g, 2.0 mmol) in methanol (5 mL) was added K_2CO_3 (0.41 g, 3.0 mmol) at room temperature. The reaction was stirred for 3 h and then quenched with saturated aqueous NH_4Cl solution (10 mL). The volatiles were evaporated under reduced pressure, and the residue was extracted with ethyl acetate (5 mL * 3). The combined organic layers were washed with saturated aqueous NH_4Cl solution (5 mL * 2) and brine, dried over Na_2SO_4, filtered, and concentrated *in vacuo*. The residue was purified by flash column chromatography on silica gel (hexane : EtOAc 100:0 to 70:30) to give the non-1-yn-5-ol (60%, 168 mg) as a colorless oil.

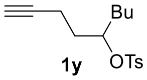

1y

Non-1-yn-5-yl 4-methylbenzenesulfonate (1y):

The general procedure A was followed with the corresponding alcohol (0.19 g, 1.4 mmol) and TsCl (0.38 g, 2.0mmol). The crude product was purified by flash column chromatography on silica gel (hexane : EtOAc 100:0 to 70:30) to give compound **1y** (44%, 175 mg) as a colorless oil. ^{1}H NMR (400 MHz, $CDCl_3$) δ 7.80 (d, J = 8.4 Hz, 2H), 7.33 (d, J = 8.0 Hz, 2H), 4.66 (p, J = 6.0 Hz, 1H), 2.44 (s, 3H), 2.27–2.00 (m, 2H), 1.91 (t, J = 2.4 Hz, 1H), 1.86–1.75 (m, 2H), 1.65–1.55 (m, 2H), 1.30–1.13 (m, 4H), 0.87–0.77 (m, 3H). ^{13}C NMR (100 MHz, $CDCl_3$) δ 144.7, 134.5, 129.9, 127.9, 83.0, 82.8, 69.1, 34.0, 33.1, 26.8, 22.4, 21.8, 14.6, 13.9. HRMS (ESI+) calcd for $C_{16}H_{22}NaO_3S$ $[M+Na]^+$ 317.1182, found 317.1178.

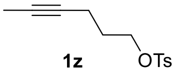

1z

Hex-4-yn-1-yl 4-methylbenzenesulfonate (1z):

The general procedure A was followed with the corresponding alcohol (0.10 g, 1.0 mmol) and TsCl (0.29 g, 1.5 mmol). The crude product was purified by flash column chromatography on silica gel (hexane : EtOAc 100:0 to 70:30) to give compound **1z** (87%, 0.22 g) as a colorless oil. ^{1}H NMR (400 MHz, $CDCl_3$) δ 7.79 (d, J = 8.4 Hz, 2H), 7.34 (d, J = 8.0 Hz, 2H), 4.13 (t, J = 6.0 Hz, 2H), 2.44 (s, 3H), 2.22–2.13 (m, 2H), 1.79 (p, J = 6.4 Hz, 2H), 1.68 (t, J = 2.4 Hz, 3H). ^{13}C NMR (100 MHz, $CDCl_3$) δ 144.8, 133.2, 129.9, 128.1, 76.9, 76.8, 69.2, 28.3, 21.8, 15.1, 3.5. HRMS (ESI+) calcd for $C_{13}H_{16}NaO_3S$ $[M+Na]^+$ 275.0712, found 275.0714.

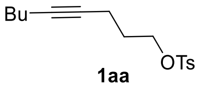

1aa

Non-4-yn-1-yl 4-methylbenzenesulfonate (1aa):

The general procedure A was followed with the corresponding alcohol (0.36 g, 2.5 mmol) and TsCl (0.73 g, 3.8 mmol). The crude product was purified by flash column chromatography on silica gel (hexane : EtOAc 100:0 to 70:30) to give compound **1aa** (67%, 0.49 g) as a colorless oil. ^{1}H NMR (400 MHz, $CDCl_3$) δ 7.79 (d, J = 8.4 Hz, 2H), 7.34 (d, J = 8.0 Hz, 2H), 4.13 (t, J = 6.4 Hz, 2H), 2.44 (s, 3H), 2.24–2.14 (m, 2H), 2.10–1.99 (m, 2H), 1.79 (p, J = 6.4 Hz, 2H), 1.45–1.26 (m, 4H), 0.87 (t, J = 7.2 Hz, 3H). ^{13}C NMR (100 MHz, $CDCl_3$) δ 144.8, 133.2, 129.9, 128.0, 81.6, 77.7, 69.3,

31.1, 28.4, 22.0, 21.7, 18.4, 15.1, 13.7. HRMS (ESI+) calcd for $C_{16}H_{22}NaO_3S$ $[M+Na]^+$ 317.1182, found 317.1183.

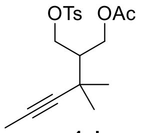

1ab

3,3-Dimethyl-2-((tosyloxy)methyl)hex-4-yn-1-yl acetate (1ab):

The general procedure A was followed with the corresponding alcohol (74 mg, 0.37 mmol) and TsCl (0.11 g, 0.56 mmol). The crude product was purified by flash column chromatography on silica gel (hexane : EtOAc 100:0 to 70:30) to give compound **1ab** (73%, 95 mg) as a colorless oil. ^1H NMR (400 MHz, $CDCl_3$) δ 7.80 (d, J = 8.0 Hz, 2H), 7.35 (d, J = 8.0 Hz, 2H), 4.34–4.21 (m, 2H), 4.18 (dd, J = 10.0, 6.4 Hz, 1H), 4.09 (dd, J = 11.2, 7.2 Hz, 1H), 2.45 (s, 3H), 1.95 (s, 3H), 1.89 (p, J = 5.6 Hz, 2H), 1.70 (s, 3H), 1.18 (s, 6H). ^{13}C NMR (100 MHz, $CDCl_3$) δ 170.8, 144.9, 133.0, 129.9, 128.0, 83.7, 77.3, 68.6, 62.0, 46.5, 32.4, 28.4, 27.9, 21.7, 20.9, 3.4. HRMS (ESI+) calcd for $C_{18}H_{25}O_5S$ $[M+H]^+$ 353.1417, found 353.1420.

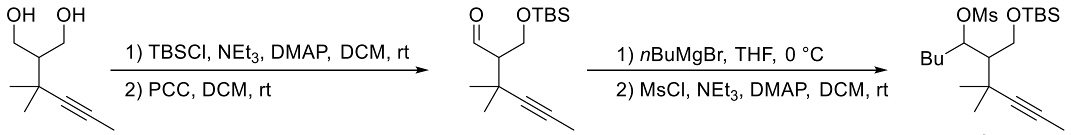

1ac

To a solution of the diol (1.1 g, 7.0 mmol), DMAP (86 mg, 0.70 mmol), and NEt_3 (1.2 mL, 8.4 mmol) in DCM (10 mL) was added TBSCl (1.0 g, 7.0 mmol) at 0 °C. The reaction mixture was allowed to warm to room temperature and was stirred for 4 h. The reaction was quenched with saturated aqueous NH_4Cl solution (10 mL), and the reaction mixture was extracted with DCM (10 mL * 3). The combined organic layers were washed with saturated aqueous NH_4Cl solution (10 mL * 2) and brine, dried over Na_2SO_4, filtered, and concentrated *in vacuo*. The residue was dissolved in DCM (10 mL), to which was added PCC (1.9 g, 9.0 mmol) in portions at 0 °C. The reaction mixture was allowed to warm to room temperature and stirred for 2 h. The reaction was filtered, and concentrated *in vacuo*. The residue was purified by flash column chromatography on silica gel (hexane : EtOAc 100:1 to 30:1) to give the product of aldehyde (68%, 1.1 g) as a colorless oil.

The obtained aldehyde (1.1 g, 4.0 mmol) was dissolved in THF (8 mL), followed by the dropwise addition of *n*-BuMgBr (1.0 M in THF, 6.0 mL, 6.0 mmol) at 0 °C under Ar. The reaction mixture was allowed to warm to room temperature and was stirred for 2 h. The reaction was quenched with saturated aqueous NH_4Cl solution (10 mL), and the reaction mixture was extracted with EtOAc (10 mL * 3). The combined organic layers were washed with saturated aqueous NH_4Cl solution (10 mL * 2) and brine, dried over Na_2SO_4, filtered, and concentrated *in vacuo*. The residue was purified by flash column chromatography on silica gel (hexane : EtOAc 100:1 to 30:1) to give the product of alcohol (71%, 0.9 g) as a colorless oil. To a solution of the alcohol (0.3 g, 1.0 mmol), DMAP (12 mg, 0.10 mmol), and NEt_3 (0.20 mL, 1.5 mmol) in DCM (2 mL) was added MsCl (0.17 g, 1.5 mmol) at 0 °C. The reaction mixture was allowed to warm to room temperature and stirred for 4 h. The reaction was quenched with saturated aqueous NH_4Cl solution (5 mL), and the reaction mixture was extracted with DCM (5 mL * 3). The combined organic layers were washed with saturated aqueous NH_4Cl solution (5 mL * 2) and brine, dried over Na_2SO_4, filtered, and concentrated *in vacuo*. The residue was purified by flash column chromatography on silica gel (hexane : EtOAc 100:1 to 50:1) to give the product **1ac** (50%, 0.2 g) as a colorless oil. ^1H NMR (400 MHz, $CDCl_3$) δ 5.35–5.06 (m, 1H), 3.83 (d, J = 5.6 Hz, 2H), 3.00 (s, 3H), 2.09–2.00 (m, 1H), 1.97–1.89 (m, 1H), 1.82–1.76 (m, 1H), 1.76 (s, 3H), 1.42–1.32 (m, 4H), 1.27 (s, 3H), 1.25 (s, 3H), 0.94–0.84 (m, 12H), 0.07 (s, 6H). ^{13}C NMR (100 MHz, $CDCl_3$) δ 85.8, 84.2, 60.5, 52.4, 39.6, 33.8, 33.1, 28.8, 28.6, 28.2, 26.0, 22.6, 18.2, 14.1, 3.7, -5.3. HRMS (ESI+) calcd for $C_{20}H_{40}NaO_4SSi$ $[M+Na]^+$ 427.2309, found 427.2308.

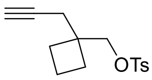

1ad

(1-(Prop-2-yn-1-yl)cyclobutyl)methyl 4-methylbenzenesulfonate (1ad):

The general procedure A was followed with the corresponding alcohol (0.50 g, 4.0 mmol) and TsCl (1.1 g, 6.0 mmol). The crude product was purified by flash column chromatography on silica gel (hexane : EtOAc 100:0 to 70:30) to give compound **1ad** (50%, 0.55 g) as a colorless oil. ^1H NMR (400 MHz, $CDCl_3$) δ 7.80 (d, J = 8.0 Hz, 2H), 7.35 (d, J = 7.6 Hz, 2H), 4.03 (s, 2H), 2.45 (s, 3H), 2.33 (s, 2H), 1.95–1.71 (m, 7H). ^{13}C NMR (100 MHz, $CDCl_3$) δ 144.8, 132.9, 129.9, 128.1, 80.6, 74.3, 70.1, 40.48, 27.6, 26.2, 21.7, 14.8. HRMS (ESI+) calcd for $C_{15}H_{18}NaO_3S$ $[M+Na]^+$ 301.0869, found 301.0865.

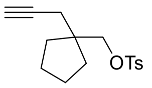

1ae

(1-(Prop-2-yn-1-yl)cyclopentyl)methyl 4-methylbenzenesulfonate (1ae):

The general procedure A was followed with the corresponding alcohol (0.39 g, 2.8 mmol) and TsCl (0.80 g, 4.2 mmol). The crude product was purified by flash column chromatography on silica gel (hexane : EtOAc 100:0 to 70:30) to give compound **1ae** (60%, 0.49 g) as a colorless oil. ^{1}H NMR (400 MHz, $CDCl_3$) δ 7.80 (d, J = 7.6 Hz, 2H), 7.34 (d, J = 7.6 Hz, 2H), 3.89 (s, 2H), 2.45 (s, 3H), 2.22 (s, 2H), 1.78 (s, 1H), 1.65–1.45 (m, 8H). ^{13}C NMR (100 MHz, $CDCl_3$) δ 144.8, 132.9, 129.9, 128.1, 81.3, 74.7, 69.8, 45.5, 34.1, 26.4, 25.0, 21.7. HRMS (ESI+) calcd for $C_{16}H_{20}NaO_3S$ $[M+Na]^+$ 315.1025, found 315.1021.

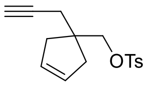

1af

(1-(Prop-2-yn-1-yl)cyclopent-3-en-1-yl)methyl 4-methylbenzenesulfonate (1af):

The general procedure A was followed with the corresponding alcohol (0.17 g, 1.3 mmol) and TsCl (0.36 g, 1.9 mmol). The crude product was purified by flash column chromatography on silica gel (hexane : EtOAc 100:0 to 70:30) to give compound **1af** (44%, 166 mg) as a colorless oil. ^{1}H NMR (400 MHz, $CDCl_3$) δ 7.80 (d, J = 8.0 Hz, 2H), 7.34 (d, J = 8.0 Hz, 2H), 5.55 (s, 2H), 3.97 (s, 2H), 2.45 (s, 3H), 2.33–2.30 (m, 2H), 2.29– 2.18(m, 4H), 1.81 (s, 1H). ^{13}C NMR (100 MHz, $CDCl_3$) δ 144.9, 132.9, 130.0, 128.7, 128.2, 81.1, 74.4, 70.3, 44.7, 40.9, 26.5, 21.8. HRMS (ESI+) calcd for $C_{16}H_{18}NaO_3S$ $[M+Na]^+$ 313.0869, found 313.0866.

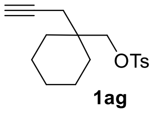

1ag

(1-(Prop-2-yn-1-yl)cyclohexyl)methyl 4-methylbenzenesulfonate (1ag):

The general procedure A was followed with the corresponding alcohol (0.58 g, 3.8 mmol) and TsCl (1.1 g, 5.8 mmol). The crude product was purified by flash column chromatography on silica gel (hexane : EtOAc 100:0 to 70:30) to give compound **1ag** (65%, 759 mg) as a colorless oil. ^{1}H NMR (400 MHz, $CDCl_3$) δ 7.80 (d, J = 8.0 Hz, 2H), 7.34 (d, J = 8.0 Hz, 2H), 3.91 (s, 2H), 2.45 (s, 3H), 2.21 (s, 2H), 1.81 (s, 1H), 1.38 (s, 10H). The ^{1}H NMR data matched with those reported in the literature.[3]

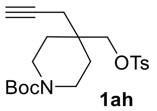

1ah

Tert-butyl 4-(prop-2-yn-1-yl)-4-((tosyloxy)methyl)piperidine-1-carboxylate (1ah):

The general procedure A was followed with the corresponding alcohol (0.30 g, 1.1 mmol) and TsCl (314 mg, 1.65 mmol). The crude product was purified by flash column chromatography on silica gel (hexane : EtOAc 100:0 to 60:40) to give compound **1ah** (74%, 346 mg) as a colorless oil. ^{1}H NMR (400 MHz, $CDCl_3$) δ 7.79 (d, J = 7.6 Hz, 2H), 7.35 (d, J = 7.6 Hz, 2H), 3.94 (s, 2H), 3.37 (dt, J = 11.2, 5.2 Hz, 1H), 3.26 (dt, J = 13.2, 5.6 Hz, 1H), 2.45 (s, 3H), 2.27 (s, 2H), 1.86 (s, 1H), 1.55–1.45 (m, 4H), 1.43 (s, 9H). ^{13}C NMR (100 MHz, $CDCl_3$) δ154.9, 145.1, 132.7, 130.0, 128.2, 79.9, 79.4, 73.2, 71.6, 39.1, 35.7, 30.8, 28.5, 24.3, 21.8. HRMS (ESI+) calcd for $C_{21}H_{29}NNaO_3S$ $[M+Na]^+$ 430.1659, found 430.1656.

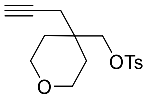

1ai

(4-(Prop-2-yn-1-yl)tetrahydro-2H-pyran-4-yl)methyl 4-methylbenzenesulfonate (1ai):

The general procedure A was followed with the corresponding alcohol (0.42 g, 2.7 mmol) and TsCl (0.77 g, 4.1 mmol). The crude product was purified by flash column chromatography on silica gel (hexane : EtOAc 100:0 to 70:30) to give compound **1ai** (74%, 616 mg) as a colorless oil. ^{1}H NMR (400 MHz, $CDCl_3$) δ 7.78 (d, J = 8.0 Hz, 2H), 7.34 (d, J = 8.0 Hz, 2H), 3.96 (s, 2H), 3.56 (t, J = 5.2 Hz, 4H), 2.43 (s, 3H), 2.31 (d, J = 2.4 Hz, 2H), 1.85 (t, J = 2.8 Hz, 1H), 1.59–1.41 (m, 4H). ^{13}C NMR (100 MHz, $CDCl_3$) δ 145.0, 132.7, 130.0, 128.1, 79.4, 73.3, 71.5, 63.1, 34.7, 31.4, 24.7, 21.7. HRMS (ESI+) calcd for $C_{16}H_{21}O_4S$ $[M+H]^+$ 309.1155, found 309.1151.

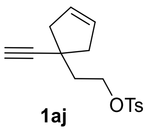

1aj

2-(1-Ethynylcyclopent-3-en-1-yl)ethyl 4-methylbenzenesulfonate (1aj):

The general procedure A was followed with the corresponding alcohol (35 mg, 0.26 mmol) and TsCl (74 mg, 0.39 mmol). The crude product was purified by flash column chromatography on silica gel (hexane : EtOAc 100:0 to 70:30) to give compound **1aj** (90%, 68 mg) as a colorless oil.

^{1}H NMR (400 MHz, $CDCl_3$) δ 7.80 (d, J = 7.6 Hz, 2H), 7.35 (d, J = 8.0 Hz, 2H), 5.62 (s, 2H), 4.25 (t, J = 6.8 Hz, 2H), 2.66 (d, J = 15.2 Hz, 2H), 2.45 (s, 3H), 2.40 (d, J = 15.2 Hz, 2H), 2.06 (s, 1H), 1.95 (t, J = 7.0 Hz, 2H). ^{13}C NMR (100 MHz, $CDCl_3$) δ 144.9, 133.2, 130.0, 128.6, 128.1, 89.9, 69.1, 68.6, 47.0, 39.2, 38.7, 21.8. HRMS (ESI+) calcd for $C_{16}H_{19}O_3S$ [M+H]$^+$ 291.1049, found 291.1050.

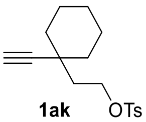

1ak

2-(1-Ethynylcyclohexyl)ethyl 4-methylbenzenesulfonate (1ak):

The general procedure A was followed with the corresponding alcohol (70 mg, 0.46 mmol) and TsCl (0.13 g, 0.69 mmol). The crude product was purified by flash column chromatography on silica gel (hexane : EtOAc 100:0 to 70:30) to give compound **1ak** (65%, 90 mg) as a colorless oil. ^{1}H NMR (400 MHz, $CDCl_3$) δ 7.79 (d, J = 8.4 Hz, 2H), 7.34 (d, J = 8.0 Hz, 2H), 4.27 (t, J = 7.2 Hz, 2H), 2.45 (s, 3H), 2.10 (s, 1H), 1.77 (t, J = 7.2 Hz, 2H), 1.73–1.53 (m, 7H), 1.24–1.07 (m, 3H). ^{13}C NMR (100 MHz, $CDCl_3$) δ 144.8, 133.4, 129.9, 128.1, 87.9, 71.6, 68.1, 41.2, 37.8, 35.3, 25.9, 22.8, 21.8. HRMS (ESI+) calcd for $C_{17}H_{23}O_3S$ [M+H]$^+$ 307.1362, found 307.1361.

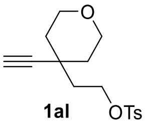

1al

2-(4-Ethynyltetrahydro-2H-pyran-4-yl)ethyl 4-methylbenzenesulfonate (1al):

The general procedure A was followed with the corresponding alcohol (27 mg, 0.18 mmol) and TsCl (50 mg, 0.26 mmol). The crude product was purified by flash column chromatography on silica gel (hexane : EtOAc 100:0 to 70:30) to give compound **1al** (72%, 40 mg) as a yellow oil. ^{1}H NMR (400 MHz, $CDCl_3$) δ 7.73 (d, J = 7.6 Hz, 2H), 7.28 (d, J = 7.6 Hz, 2H), 4.23 (t, J = 6.8 Hz, 2H), 3.73 (d, J = 10.8 Hz, 2H), 3.64 (t, J = 11.6 Hz, 2H), 2.39 (s, 3H), 2.14 (s, 1H), 1.75 (t, J = 6.8 Hz, 2H), 1.51 (t, J = 16.0 Hz, 4H). ^{13}C NMR (100 MHz, $CDCl_3$) δ 144.9, 133.3, 130.0, 128.1, 86.0, 73.1, 67.4, 64.6, 41.2, 37.7, 33.4, 21.8. HRMS (ESI+) calcd for $C_{16}H_{21}O_4S$ [M+H]$^+$ 309.1155, found 309.1157.

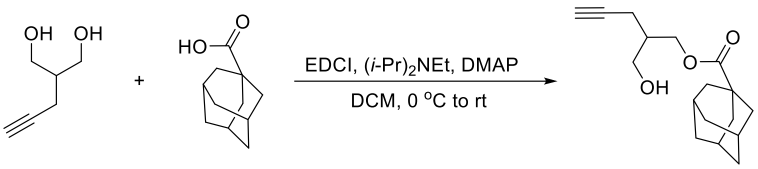

To a solution of the diol (137 mg, 1.20 mmol), EDCI (288 mg, 1.50 mmol), N,N-diisopropylethylamine (0.30 mL, 1.5 mmol) and DMAP (37 mg, 0.30 mmol) in DCM (5 mL) was added 1-adamantanecarboxylic acid (194 mg, 1.00 mmol) at 0 °C under Ar. The reaction mixture was allowed to warm to room temperature and stirred overnight. The reaction was quenched with saturated aqueous NH_4Cl solution (10 mL), and the reaction mixture was extracted with DCM (5 mL * 3). The combined organic layers were washed with saturated aqueous NH_4Cl solution (5 mL * 2) and brine, dried over Na_2SO_4, filtered, and concentrated *in vacuo*. The residue was purified by flash column chromatography on silica gel (hexane : EtOAc 100:0 to 70:30) to give the alcohol (59%, 170 mg) as a white solid.

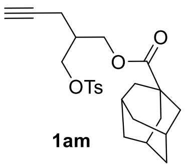

1am

2-((Tosyloxy)methyl)pent-4-yn-1-yl (1s,3s)-adamantane-1-carboxylate (1am):

The general procedure A was followed with the corresponding alcohol (0.17 g, 0.59 mmol) and TsCl (0.17g, 0.90 mmol). The crude product was purified by flash column chromatography on silica gel (hexane : EtOAc 100:0 to 70:30) to give compound **1am** (61%, 155 mg) as a white solid. ^{1}H NMR (400 MHz, $CDCl_3$) δ 7.73 (d, J = 8.4 Hz, 2H), 7.28 (d, J = 8.0 Hz, 2H), 4.06–3.91 (m, 4H), 2.38 (s, 3H), 2.27–2.13 (m, 3H), 1.92 (s, 3H), 1.87 (t, J = 2.4 Hz, 1H), 1.72 (d, J = 2.4 Hz, 6H), 1.62 (q, J = 12.0 Hz, 6H). ^{13}C NMR (100 MHz, $CDCl_3$) δ 177.1, 145.1, 132.7, 130.0, 128.1, 80.0, 70.9, 68.8, 62.4, 40.9, 38.8, 37.2, 36.5, 27.9, 21.8, 17.7. HRMS (ESI+) calcd for $C_{24}H_{30}NaO_5S$ $[M+Na]^+$ 453.1706, found 453.1700.

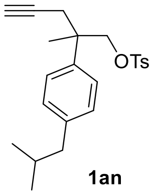

1an

2-(4-Isobutylphenyl)-2-methylpent-4-yn-1-yl 4-methylbenzenesulfonate (1an):

The general procedure A was followed with the corresponding alcohol (1.2 g, 5.0 mmol) and TsCl (1.4 g, 7.5 mmol). The crude product was purified by flash column chromatography on silica gel (hexane : EtOAc 100:0 to 70:30) to give compound **1an** (70%, 1.34 g) as a white solid. ^{1}H NMR (400 MHz, $CDCl_3$) δ 7.68 (d, J = 8.0 Hz, 2H), 7.28 (d, J = 8.0 Hz, 2H), 7.15 (d, J = 8.4 Hz, 2H), 7.05 (d, J = 8.4 Hz, 2H), 4.13 (s, 2H), 2.67–2.52 (m, 2H), 2.48–2.39 (m, 5H), 1.89–1.80 (m, 2H), 1.40 (s, 3H), 0.90 (d, J = 6.4 Hz, 6H). ^{13}C NMR (100 MHz, $CDCl_3$) δ 144.7, 140.5, 139.6, 132.9, 129.8, 129.2, 128.1, 125.9, 80.5, 75.9, 71.4, 45.0, 41.1, 30.2, 28.1, 22.9, 22.5, 21.7. HRMS (ESI+) calcd for $C_{23}H_{28}NaO_3S$ $[M+Na]^+$ 407.1651, found 407.1648.

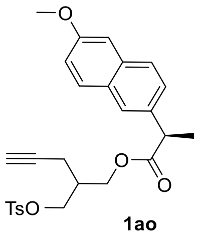

1ao

2-((Tosyloxy)methyl)pent-4-yn-1-yl (2R)-2-(6-methoxynaphthalen-2-yl)propanoate (1ao):

To a solution of the alcohol **S1** (536 mg, 0.700 mmol), EDCI (192 mg, 1.00 mmol), N,N-diisopropylethylamine (129 mg, 1.00 mmol) and DMAP (25 mg, 0.20 mmol) in DCM (10 mL) was added Naproxen (184 mg, 0.800 mmol) at 0 °C under Ar. The reaction mixture was allowed to warm to room temperature and stirred overnight. The reaction was quenched with saturated aqueous NH_4Cl solution (10 mL), and the resulting mixture was extracted with DCM (5 mL * 3). The combined organic layers were washed with saturated aqueous NH_4Cl solution (5 mL * 2) and brine, dried over Na_2SO_4, filtered, and concentrated *in vacuo*. The residue was purified by flash column chromatography on silica gel (hexane : EtOAc 100:0 to 70:30) to give compound **1ao** (60%, 201 mg) as a white solid. ^{1}H NMR (400 MHz, $CDCl_3$) δ 7.73–7.68 (m, 4H), 7.62 (s, 1H), 7.33 (dd, J = 8.4, 1.6 Hz, 1H), 7.28 (dd, J = 8.4, 2.4 Hz, 2H), 7.15 (dd, J = 8.8, 2.4 Hz, 1H), 7.12 (d, J = 2.4 Hz, 1H), 4.14–3.94 (m, 5H), 3.92 (s, 3H), 3.80 (q, J = 7.2 Hz, 1H), 2.43 (s, 3H), 2.21–2.12 (m, 3H), 1.89–1.85 (m, 1H), 1.54 (d, J = 7.2 Hz, 3H). ^{13}C NMR (100 MHz, $CDCl_3$) δ 174.3, 157.8, 145.1, 135.4, 133.8, 132.7, 130.0, 129.4, 129.0, 128.1, 127.4, 126.2, 126.0, 119.2, 105.7, 79.8, 70.9, 68.74, 68.68, 63.0, 55.4, 45.4, 37.1, 21.8, 18.4, 17.52, 17.49. HRMS (ESI+) calcd for $C_{27}H_{29}O_6S$ $[M+H]^+$ 481.1679, found 481.1678.

3. Condition Optimization

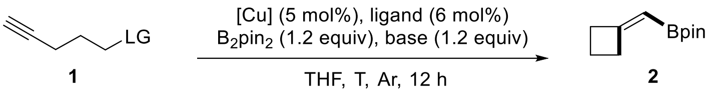

Procedure E: In an argon-filled glovebox, copper precatalyst (5 μmol, 5 mol%), base (0.12 mmol, 1.2 equiv), and THF (0.5 mL) were added to a vial (5 mL) equipped with a stirring bar, followed by the addition of the ligand (6 μmol, 6 mol%). The reaction mixture was stirred for 5 minutes, followed by the addition of B_2pin_2 (30.5 mg, 0.120 mmol, 1.2 equiv), the alkyne **1** (0.10 mmol), and THF (0.5 mL). The vial was sealed, and the reaction mixture was stirred at T °C for 12 h. The yield was determined by ^{1}H NMR analysis with 1,3,5-trimethoxybenzene used as internal standard.

4. General procedure for the borylative cyclization of alkynes

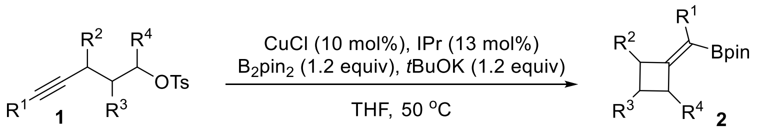

Procedure F: In an argon-filled glovebox, CuCl (2.0 mg, 20 μmol, 10 mol%), potassium tert-butoxide (26.9 mg, 0.240 mmol, 1.20 equiv), and THF (0.5 mL) were added to a 4 mL vial charged with a stirring bar. After the mixture was stirred for 5 minutes, 1,3-bis(2,4,6-trimethylphenyl) imidazolium chloride (8.9 mg, 26 μmol, 13 mol%) was added, followed by the addition of B_2pin_2 (61 mg, 0.24 mmol, 1.2 equiv), the alkyne **1** (0.20 mmol), and THF (0.5 mL). The vial was sealed with a Teflon-lined cap, and the reaction mixture was stirred at 50 °C for 24 h. After cooled to room temperature, the volatiles in the reaction mixture were evaporated under reduced pressure, and the residue was purified by column chromatography on silica gel.

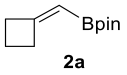

2a

2-(Cyclobutylidenemethyl)-4,4,5,5-tetramethyl-1,3,2-dioxaborolane (2a):

The borylative cyclization of alkyne was conducted by procedure F, using **1a** (47.6 mg, 0.200 mmol), B_2Pin_2 (61 mg, 0.24 mmol, 1.2 equiv), 1,3-bis(2,4,6-trimethylphenyl) imidazolium chloride (8.9 mg, 26 μmol, 13 mol%), potassium tert-butoxide (26.9 mg, 0.24 mmol, 1.20 equiv), and THF (1.0 mL). The crude product was purified by column chromatography on silica gel (hexane : EtOAc 100:0 to 80:20) (hexane:EA 100:1 to 30:1) to give the product **2a** (27.9 mg, 72%) as a colorless oil. ^{1}H NMR (400 MHz, $CDCl_3$) δ 5.08 (t, J = 2.4 Hz, 1H), 2.96–2.86 (m, 2H), 2.81–2.72 (m, 2H), 1.95 (p, J = 8.0 Hz, 2H), 1.24 (s, 12H). The ^{1}H NMR data matched with those reported in the literature.[4]

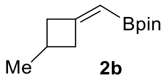

2b

4,4,5,5-Tetramethyl-2-((3-methylcyclobutylidene)methyl)-1,3,2-dioxaborolane (2b):

The borylative cyclization of alkyne was conducted by procedure F, using **1b** (50.4 mg, 0.200 mmol), B_2Pin_2 (61.0 mg, 0.240 mmol, 1.2 equiv), 1,3-bis(2,4,6-trimethylphenyl) imidazolium chloride s (8.9 mg, 26 μmol, 13 mol%), potassium tert-butoxide (26.9 mg, 0.240 mmol, 1.2 equiv) and THF (1.0 mL). The crude product was purified by column chromatography on silica gel (hexane : EtOAc 100:0 to 80:20) (hexane:EA 100:1 to 30:1) to give the product **2b** (41.6 mg, 70%) as a colorless oil. ^{1}H NMR (400 MHz, $CDCl_3$) δ 5.11 (t, J = 2.4 Hz, 1H), 3.17–2.97 (m, 1H), 2.94–2.79 (m, 1H), 2.56–2.20 (m, 3H), 1.23 (s, 12H), 1.13 (d, J = 6.4 Hz, 3H). ^{13}C NMR (100 MHz, $CDCl_3$) δ 167.2, 82.6, 42.2, 41.7, 25.2, 25.04, 24.97, 22.0 (the carbon bound to boron was not observed). ^{11}B NMR (128 MHz, $CDCl_3$) δ 29.8.

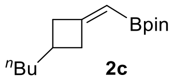

2c

2-((3-Butylcyclobutylidene)methyl)-4,4,5,5-tetramethyl-1,3,2-dioxaborolane (2c):

The borylative cyclization of alkyne was conducted by procedure F, using **1c** (58.8 mg, 0.200 mmol), B_2Pin_2 (61.0 mg, 0.240 mmol, 1.2 equiv), 1,3-bis(2,4,6-trimethylphenyl) imidazolium chloride (8.9 mg, 26 μmol, 13 mol%), potassium tert-butoxide (26.9 mg, 0.240 mmol, 1.2 equiv) and THF (1.0 mL). The crude product was purified by column chromatography on silica gel (hexane : EtOAc 100:0 to 80:20) (hexane:EA 100:1 to 30:1) to give the product **2c** (38.0 mg, 76%) as a colorless oil. ^{1}H NMR (400 MHz, $CDCl_3$) δ 5.13–5.07 (m, 1H), 3.07–2.96 (m, 1H), 2.86–2.76 (m, 1H), 2.50–

2.42 (m, 1H), 2.39–2.32 (m, 1H), 2.23 (dq, J = 15.2, 7.6 Hz, 1H), 1.45 (q, J = 7.6 Hz, 2H), 1.33–1.22 (m, 16H), 0.88 (t, J = 7.2 Hz, 3H). ^{13}C NMR (100 MHz, $CDCl_3$) δ 167.5, 82.6, 40.7, 40.1, 36.6, 30.4, 30.0, 25.0, 24.9, 22.8, 14.3 (the carbon bound to boron was not observed). ^{11}B NMR (128 MHz, $CDCl_3$) δ 29.4. HRMS (ESI+) calcd for $C_{15}H_{28}BO_2$ $[M+H]^+$ 251.2177, found 251.2178.

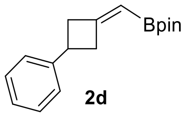

2d

4,4,5,5-Tetramethyl-2-((3-phenylcyclobutylidene)methyl)-1,3,2-dioxaborolane (2d):

The borylative cyclization of alkyne was conducted by procedure F, using **1d** (62.8 mg, 0.200 mmol), B_2Pin_2 (61.0 mg, 0.240 mmol, 1.2 equiv), 1,3-bis(2,4,6-trimethylphenyl) imidazolium chloride (8.9 mg, 26 μmol, 13 mol%), potassium tert-butoxide (26.9 mg, 0.240 mmol, 1.2 equiv) and THF (1.0 mL). The crude product was purified by column chromatography on silica gel (hexane : EtOAc 100:0 to 80:20) (hexane:EA 100:1 to 30:1) to give the product **2d** (34.0 mg, 63%) as a yellow oil. ^{1}H NMR (400 MHz, $CDCl_3$) δ 7.35–7.24 (m, 4H), 7.19 (t, J = 6.8 Hz, 1H), 5.22 (s, 1H), 3.55 (p, J = 8.4 Hz, 1H), 3.45–3.35 (m, 1H), 3.22–3.12 (m, 1H), 3.08–2.99 (m, 1H), 2.98–2.90 (m, 1H), 1.25 (s, 12H). ^{13}C NMR (100 MHz, $CDCl_3$) δ 165.1, 145.7, 128.4, 126.6, 126.0, 82.8, 42.5, 42.0, 34.9, 25.1, 25.0 (the carbon bound to boron was not observed). ^{11}B NMR (128 MHz, $CDCl_3$) δ 29.5. HRMS (ESI+) calcd for $C_{17}H_{24}BO_2$ $[M+H]^+$ 271.1864, found 271.1863.

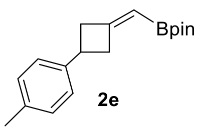

2e

4,4,5,5-Tetramethyl-2-((3-(p-tolyl)cyclobutylidene)methyl)-1,3,2-dioxaborolane (2e):

The borylative cyclization of alkyne was conducted by procedure F, using **1e** (65.6 mg, 0.200 mmol), B_2Pin_2 (61.0 mg, 0.240 mmol, 1.2 equiv), 1,3-bis(2,4,6-trimethylphenyl) imidazolium chloride (8.9 mg, 26 μmol, 13 mol%), potassium tert-butoxide (26.9 mg, 0.240 mmol, 1.2 equiv) and THF (1.0 mL). The crude product was purified by column chromatography on silica gel (hexane : EtOAc 100:0 to 80:20) (hexane:EA 100:1 to 30:1) to give the product **2e** (42.6 mg, 63%) as a yellow oil. ^{1}H NMR (400 MHz, $CDCl_3$) δ 7.17 (d, J = 8.0 Hz, 2H), 7.12 (d, J = 8.0 Hz, 2H), 5.22 (p, J = 2.0 Hz, 1H), 3.52 (p, J = 8.0 Hz, 1H), 3.43–3.34 (m, 1H), 3.20–3.11 (m, 1H), 3.06–2.97 (m, 1H), 2.96–

2.88 (m, 1H), 2.33 (s, 3H), 1.26 (s, 12H). ^{13}C NMR (100 MHz, $CDCl_3$) δ 165.3, 142.7, 135.5, 129.1, 126.5, 82.7, 42.7, 42.0, 34.6, 25.0, 24.9, 21.1 (the carbon bound to boron was not observed). ^{11}B NMR (128 MHz, $CDCl_3$) δ 29.4. HRMS (ESI+) calcd for $C_{18}H_{26}BO_2$ $[M+H]^+$ 285.2020, found 285.2018.

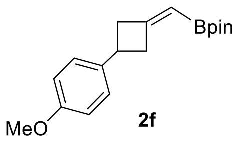

2f

2-((3-(4-Methoxyphenyl)cyclobutylidene)methyl)-4,4,5,5-tetramethyl-1,3,2-dioxaborolane (2f):

The borylative cyclization of alkyne was conducted by procedure F, using **1f** (68.8 mg, 0.200 mmol), B_2Pin_2 (61.0 mg, 0.240 mmol, 1.2 equiv), 1,3-bis(2,4,6-trimethylphenyl) imidazolium chloride (8.9 mg, 26 μmol, 13 mol%), potassium tert-butoxide (26.9 mg, 0.240 mmol, 1.2 equiv) and THF (1.0 mL). The crude product was purified by column chromatography on silica gel (hexane:EA 100:1 to 30:1) to give the product **2f** (43.2 mg, 72%) as a yellow oil. ^1H NMR (400 MHz, $CDCl_3$) δ 7.19 (d, J = 8.4 Hz, 2H), 6.85 (d, J = 8.8 Hz, 2H), 5.22 (p, J = 2.0 Hz, 1H), 3.79 (s, 3H), 3.49 (p, J = 8.4 Hz, 1H), 3.42–3.33 (m, 1H), 3.19–3.10 (m, 1H), 3.03–2.95 (m, 1H), 2.93–2.85 (m, 1H), 1.26 (s, 12H). ^{13}C NMR (100 MHz, $CDCl_3$) δ 165.3, 158.0, 137.9, 127.6, 113.9, 82.8, 55.4, 42.8, 42.2, 34.3, 25.1, 25.0 (the carbon bound to boron was not observed). ^{11}B NMR (128 MHz, $CDCl_3$) δ 29.7. HRMS (ESI+) calcd for $C_{18}H_{26}BO_3$ $[M+H]^+$ 301.1970, found 301.1967.

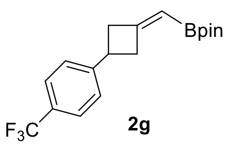

2g

4,4,5,5-Tetramethyl-2-((3-(4-(trifluoromethyl)phenyl)cyclobutylidene)methyl)-1,3,2-dioxaborolane (2g):

The borylative cyclization of alkyne was conducted by procedure F, using **1g** (76.4 mg, 0.200 mmol), B_2Pin_2 (61.0 mg, 0.240 mmol, 1.2 equiv), 1,3-bis(2,4,6-trimethylphenyl) imidazolium chloride (8.9 mg, 26 μmol, 13 mol%), potassium tert-butoxide (26.9 mg, 0.240 mmol, 1.2 equiv) and THF (1.0 mL). The crude product was purified by column chromatography on silica gel (hexane:EA 100:1 to

30:1) to give the product **2g** (41.2 mg, 61%) as a yellow oil. ^{1}H NMR (400 MHz, $CDCl_3$) δ 7.56 (d, J = 8.0 Hz, 2H), 7.37 (d, J = 8.0 Hz, 2H), 5.25 (s, 1H), 3.60 (p, J = 8.0 Hz, 1H), 3.49–3.39 (m, 1H), 3.26–3.16 (m, 1H), 3.08–2.99 (m, 1H), 2.98–2.89 (m, 1H), 1.26 (s, 12H). ^{13}C NMR (100 MHz, $CDCl_3$) δ 163.9, 149.8, 127.0, 125.4 (q, J = 3.7 Hz), 128.4 (q, J = 32.1 Hz), 124.5 (q, J = 270.0 Hz), 82.9, 42.3, 41.8, 34.8, 25.1, 25.0 (the carbon bound to boron was not observed). ^{11}B NMR (128 MHz, $CDCl_3$) δ 28.8. ^{19}F NMR (376 MHz, $CDCl_3$) δ -62.3. HRMS (ESI+) calcd for $C_{18}H_{23}BF_3O_2$ $[M+H]^+$ 339.1738, found 339.1735.

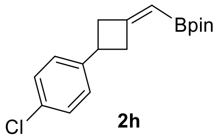

2h

2-((3-(4-Chlorophenyl)cyclobutylidene)methyl)-4,4,5,5-tetramethyl-1,3,2-dioxaborolane (2h): The borylative cyclization of alkyne was conducted by procedure F, using **1h** (69.6 mg, 0.200 mmol), B_2Pin_2 (61.0 mg, 0.240 mmol, 1.2 equiv), 1,3-bis(2,4,6-trimethylphenyl) imidazolium chloride (8.9 mg, 26 µmol, 13 mol%), potassium tert-butoxide (26.9 mg, 0.240 mmol, 1.2 equiv) and THF (1.0 mL). The crude product was purified by column chromatography on silica gel (hexane:EA 100:1 to 30:1) to give the product **2h** (42.5 mg, 70%) as a yellow oil. ^{1}H NMR (400 MHz, $CDCl_3$) δ 7.28–7.25 (m, 2H), 7.19 (d, J = 8.4 Hz, 2H), 5.23 (p, J = 2.4 Hz, 1H), 3.58–3.45 (m, 1H), 3.46–3.32 (m, 1H), 3.23–3.10 (m, 1H), 3.04–2.92 (m, 1H), 2.93–2.83 (m, 1H), 1.25 (s, 12H). ^{13}C NMR (100 MHz, $CDCl_3$) δ 164.3, 144.2, 131.7, 128.5, 128.0, 82.8, 42.4, 42.0, 34.4, 25.1, 25.0 (the carbon bound to boron was not observed). ^{11}B NMR (128 MHz, $CDCl_3$) δ 29.1. HRMS (ESI+) calcd for $C_{17}H_{23}BClO_2$ $[M+H]^+$ 305.1474, found 305.1473.

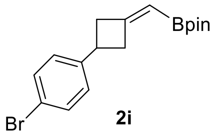

2i

2-((3-(4-Bromophenyl)cyclobutylidene)methyl)-4,4,5,5-tetramethyl-1,3,2-dioxaborolane (2i): The borylative cyclization of alkyne was conducted by procedure F, using **1i** (78.4 mg, 0.200 mmol), B_2Pin_2 (61.0 mg, 0.240 mmol, 1.2 equiv), 1,3-bis(2,4,6-trimethylphenyl) imidazolium chloride (8.9 mg, 26 µmol, 13 mol%), potassium tert-butoxide (26.9 mg, 0.240 mmol, 1.2 equiv) and THF (1.0

mL). The crude product was purified by column chromatography on silica gel (hexane:EA 100:1 to 30:1) to give the product **2i** (47.3 mg, 68%) as a yellow oil. ^{1}H NMR (400 MHz, $CDCl_3$) δ 7.42 (d, J = 8.4 Hz, 2H), 7.14 (d, J = 8.4 Hz, 2H), 5.23 (p, J = 2.4 Hz, 1H), 3.50 (p, J = 8.4 Hz, 1H), 3.44–3.34 (m, 1H), 3.21–3.12 (m, 1H), 3.02–2.94 (m, 1H), 2.92–2.84 (m, 1H), 1.25 (s, 12H). ^{13}C NMR (100 MHz, $CDCl_3$) δ 164.3, 144.7, 131.5, 128.4, 119.7, 82.8, 42.4, 41.9, 34.4, 25.1, 24.9 (the carbon bound to boron was not observed). ^{11}B NMR (128 MHz, $CDCl_3$) δ 29.9. HRMS (ESI+) calcd for $C_{17}H_{23}BBrO_2$ $[M+H]^+$ 349.0969, found 349.0966.

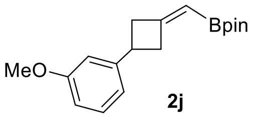

2j

2-((3-(3-Methoxyphenyl)cyclobutylidene)methyl)-4,4,5,5-tetramethyl-1,3,2-dioxaborolane (2j):

The borylative cyclization of alkyne was conducted by procedure F, using **1j** (68.8 mg, 0.200 mmol), B_2Pin_2 (61.0 mg, 0.240 mmol, 1.2 equiv), 1,3-bis(2,4,6-trimethylphenyl) imidazolium chloride (8.9 mg, 26 μmol, 13 mol%), potassium tert-butoxide (26.9 mg, 0.240 mmol, 1.2 equiv) and THF (1.0 mL). The crude product was purified by column chromatography on silica gel (hexane:EA 100:1 to 30:1) to give the product **2j** (45.0 mg, 75%) as a yellow oil. ^{1}H NMR (400 MHz, $CDCl_3$) δ 7.22 (t, J = 8.0 Hz, 1H), 6.86 (d, J = 7.6 Hz, 1H), 6.82 (d, J = 2.0 Hz, 1H), 6.74 (dd, J = 8.0, 2.0 Hz, 1H), 5.22 (p, J = 2.4 Hz, 1H), 3.80 (s, 3H), 3.53 (p, J = 8.4 Hz, 1H), 3.43–3.33 (m, 1H), 3.20–3.10 (m, 1H), 3.07–2.99 (m, 1H), 2.98–2.90 (m, 1H), 1.25 (s, 12H). ^{13}C NMR (100 MHz, $CDCl_3$) δ 165.0, 159.8, 147.4, 129.4, 119.0, 112.4, 111.4, 82.8, 55.3, 42.4, 41.9, 35.0, 25.0, 24.9 (the carbon bound to boron was not observed). ^{11}B NMR (128 MHz, $CDCl_3$) δ 29.6. HRMS (ESI+) calcd for $C_{18}H_{26}BO_3$ $[M+H]^+$ 301.1970, found 301.1967.

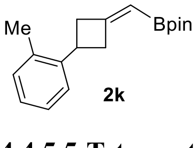

2k

4,4,5,5-Tetramethyl-2-((3-(o-tolyl)cyclobutylidene)methyl)-1,3,2-dioxaborolane (2k):

The borylative cyclization of alkyne was conducted by procedure F, using **1k** (65.6 mg, 0.200 mmol), B_2Pin_2 (61.0 mg, 0.240 mmol, 1.2 equiv), 1,3-bis(2,4,6-trimethylphenyl) imidazolium chloride (8.9 mg, 26 μmol, 13 mol%), potassium tert-butoxide (26.9 mg, 0.240 mmol, 1.2 equiv) and THF (1.0

mL). The crude product was purified by column chromatography on silica gel (hexane:EA 100:1 to 30:1) to give the product **2k** (35.8 mg, 63%) as a yellow oil. ^{1}H NMR (400 MHz, $CDCl_3$) δ 7.31 (d, J = 7.6 Hz, 1H), 7.20 (t, J = 6.0 Hz, 1H), 7.18–7.11 (m, 2H), 5.23 (s, 1H), 3.66 (p, J = 8.4 Hz, 1H), 3.45–3.32 (m, 1H), 3.21–3.10 (m, 1H), 3.08–3.00 (m, 1H), 2.99–2.90 (m, 1H), 2.28 (s, 3H), 1.27 (s, 12H). ^{13}C NMR (100 MHz, $CDCl_3$) δ 164.9, 142.8, 136.2, 130.1, 126.1, 126.0, 125.2, 82.7, 68.1, 41.2, 40.4, 32.7, 25.0, 25.0. (the carbon bound to boron was not observed). ^{11}B NMR (128 MHz, $CDCl_3$) δ 29.0. HRMS (ESI+) calcd for $C_{18}H_{26}BO_2$ [M+H]$^+$ 285.2020, found 285.2019.

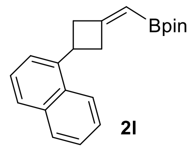

2l

4,4,5,5-Tetramethyl-2-((3-(naphthalen-1-yl)cyclobutylidene)methyl)-1,3,2-dioxaborolane (2l): The borylative cyclization of alkyne was conducted by procedure F, using **1l** (72.8 mg, 0.200 mmol), B_2Pin_2 (61.0 mg, 0.240 mmol, 1.2 equiv), 1,3-bis(2,4,6-trimethylphenyl) imidazolium chloride (8.9 mg, 26 μmol, 13 mol%), potassium tert-butoxide (26.9 mg, 0.240 mmol, 1.2 equiv) and THF (1.0 mL). The crude product was purified by column chromatography on silica gel (hexane:EA 100:1 to 30:1) to give the product **2l** (44.8 mg, 70%) as a white solid. ^{1}H NMR (400 MHz, $CDCl_3$) δ 7.96 (d, J = 7.6 Hz, 1H), 7.92–7.83 (m, 1H), 7.73 (d, J = 7.2 Hz, 1H), 7.58–7.40 (m, 4H), 5.28 (s, 1H), 4.18 (p, J = 8.4 Hz, 1H), 3.63–3.52 (m, 1H), 3.40–3.28 (m, 1H), 3.27–3.07 (m, 2H), 1.28 (d, J = 3.2 Hz, 12H). ^{13}C NMR (100 MHz, $CDCl_3$) δ 164.9, 140.6, 133.9, 131.8, 128.8, 126.8, 125.9, 125.6, 125.6, 124.3, 122.5, 82.8, 41.3, 40.7, 32.5, 25.1, 25.0 (the carbon bound to boron was not observed). ^{11}B NMR (128 MHz, $CDCl_3$) δ 29.7. HRMS (ESI+) calcd for $C_{21}H_{26}BO_2$ [M+H]$^+$ 321.2020, found 321.2016.

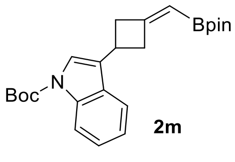

2m

Tert-butyl 3-(3-((4,4,5,5-tetramethyl-1,3,2-dioxaborolan-2-yl)methylene)cyclobutyl)-3a,7a-dihydro-1H-indole-1-carboxylate (2m):

The borylative cyclization of alkyne was conducted by procedure F, using **1m** (91 mg, 0.20 mmol), B_2Pin_2 (61.0 mg, 0.240 mmol, 1.2 equiv), 1,3-bis(2,4,6-trimethylphenyl) imidazolium chloride (8.9 mg, 26 μmol, 13 mol%), potassium tert-butoxide (26.9 mg, 0.240 mmol, 1.2 equiv) and THF (1.0 mL). The crude product was purified by column chromatography on silica gel (hexane:EA 100:1 to 30:1) to give product **2m** (28.3 mg, 71%) as a white solid. ^{1}H NMR (400 MHz, $CDCl_3$) δ 8.12 (s, 1H), 7.49 (d, J = 7.6 Hz, 1H), 7.39 (s, 1H), 7.36–7.27 (m, 1H), 7.27–7.18 (m, 1H), 5.28–5.22 (m, 1H), 3.68 (p, J = 7.6 Hz, 1H), 3.51–3.41 (m, 1H), 3.33–3.20 (m, 1H), 3.17–3.06 (m, 1H), 3.05–2.95 (m, 1H), 1.67 (s, 9H), 1.26 (s, 12H). ^{13}C NMR (100 MHz, $CDCl_3$) δ 165.3, 150.0, 136.0, 130.1, 125.1, 124.5, 122.4, 121.4, 119.5, 115.5, 83.5, 82.8, 41.1, 40.6, 28.4, 26.5, 25.04, 24.97 (the carbon bound to boron was not observed). ^{11}B NMR (128 MHz, $CDCl_3$) δ 30.1. HRMS (ESI+) calcd for $C_{24}H_{33}BNO_4$ $[M-H]^-$ 410.2497, found 410.2490.

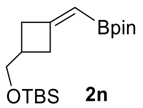

2n

Tert-butyldimethyl((3-((4,4,5,5-tetramethyl-1,3,2-dioxaborolan-2-yl)methylene)cyclobutyl)-methoxy)silane (2n):

The borylative cyclization of alkyne was conducted by procedure F, using **1n** (76.4 mg, 0.200 mmol), B_2Pin_2 (61.0 mg, 0.240 mmol, 1.2 equiv), 1,3-bis(2,4,6-trimethylphenyl) imidazolium chloride (8.9 mg, 26 μmol, 13 mol%), potassium tert-butoxide (26.9 mg, 0.240 mmol, 1.2 equiv) and THF (1.0 mL). The crude product was purified by column chromatography on silica gel (hexane:EA 100:1 to 30:1) to give the product **2n** (57.4 mg, 85%) as a colorless oil. ^{1}H NMR (400 MHz, $CDCl_3$) δ 5.15–5.10 (m, 1H), 3.62 (dd, J = 6.4, 2.0 Hz, 2H), 3.01–2.89 (m, 1H), 2.84–2.72 (m, 1H), 2.67–2.56 (m, 1H), 2.54–2.42 (m, 2H), 1.24 (s, 12H), 0.89 (s, 9H), 0.05 (s, 6H). ^{13}C NMR (100 MHz, $CDCl_3$) δ 166.8, 82.6, 67.0, 37.2, 36.6, 32.1, 26.1, 24.98, 24.97, 18.5, -5.1 (the carbon bound to boron was not observed). ^{11}B NMR (128 MHz, $CDCl_3$) δ 29.4. HRMS (ESI+) calcd for $C_{18}H_{35}BNaO_3Si$ $[M+Na]^+$ 361.2341, found 361.2335.

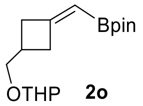

2o

4,4,5,5-Tetramethyl-2-((3-(((tetrahydro-2H-pyran-2-yl)oxy)methyl)cyclobutylidene)methyl)-1,3,2-dioxaborolane (2o):

The borylative cyclization of alkyne was conducted by procedure F, using **1o** (70.4 mg, 0.200 mmol), B_2Pin_2 (61.0 mg, 0.240 mmol, 1.2 equiv), 1,3-bis(2,4,6-trimethylphenyl) imidazolium chloride (8.9 mg, 26 μmol, 13 mol%), potassium tert-butoxide (26.9 mg, 0.240 mmol, 1.2 equiv) and THF (1.0 mL). The crude product was purified by column chromatography on silica gel (hexane:EA 50:1 to 20:1) to give the product **2o** (55.8 mg, 79%) as a colorless oil. ^{1}H NMR (400 MHz, $CDCl_3$) δ 5.14 (s, 1H), 4.60 (q, J = 3.2 Hz, 1H), 3.91–3.81 (m, 1H), 3.82–3.72 (m, 1H), 3.54–3.45 (m, 1H), 3.48–3.37 (m, 1H), 3.09–2.96 (m, 1H), 2.90–2.79 (m, 1H), 2.70–2.47 (m, 3H), 1.88–1.77 (m, 1H), 1.75–1.66 (m, 1H), 1.63–1.48 (m, 4H), 1.23 (s, 12H). ^{13}C NMR (100 MHz, $CDCl_3$) δ 166.4, 99.1, 99.0, 82.6, 71.73, 71.70, 62.4, 62.3, 37.7, 37.3, 30.8, 29.8, 25.6, 25.0, 24.9, 19.7, 19.6 (the carbon bound to boron was not observed). ^{11}B NMR (128 MHz, $CDCl_3$) δ 29.4. HRMS (ESI+) calcd for $C_{17}H_{29}BNaO_4$ $[M+Na]^+$ 331.2051, found 331.2051.

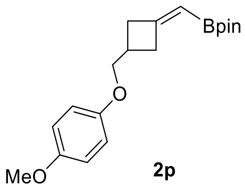

2p

2-((3-((4-Methoxyphenoxy)methyl)cyclobutylidene)methyl)-4,4,5,5-tetramethyl-1,3,2-dioxa-borolane (2p):

The borylative cyclization of alkyne was conducted by procedure F, using **1p** (74.8 mg, 0.200 mmol), B_2Pin_2 (61.0 mg, 0.240 mmol, 1.2 equiv), 1,3-bis(2,4,6-trimethylphenyl) imidazolium chloride (8.9 mg, 26 μmol, 13 mol%), potassium tert-butoxide (26.9 mg, 0.240 mmol, 1.2 equiv) and THF (1.0 mL). The crude product was purified by column chromatography on silica gel (hexane:EA 100:1 to 30:1) to give the product **2p** (51.5 mg, 78%) as a white solid. ^{1}H NMR (400 MHz, $CDCl_3$) δ 6.86–6.80 (m, 4H), 5.19 (p, J = 2.4 Hz, 1H), 3.94 (d, J = 6.4 Hz, 2H), 3.77 (s, 3H), 3.18–3.03 (m, 1H), 2.97–2.87 (m, 1H), 2.81–2.70 (m, 2H), 2.68–2.58 (m, 1H), 1.24 (s, 12H). ^{13}C NMR (100 MHz, $CDCl_3$) δ 165.7, 153.9, 153.4, 115.6, 114.7, 82.7, 72.5, 55.8, 37.5, 37.1, 29.5, 25.0, 24.9 (the carbon bound to boron was not observed). ^{11}B NMR (128 MHz, $CDCl_3$) δ 29.4. HRMS (ESI+) calcd for $C_{19}H_{27}BNaO_4$ $[M+Na]^+$ 353.1895, found 353.1888.

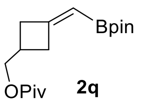

OPiv **2q**

(3-((4,4,5,5-Tetramethyl-1,3,2-dioxaborolan-2-yl)methylene)cyclobutyl)methyl pivalate (2q): The borylative cyclization of alkyne was conducted by procedure F, using **1q** (70.4 mg, 0.200 mmol), B_2Pin_2 (61.0 mg, 0.240 mmol, 1.2 equiv), 1,3-bis(2,4,6-trimethylphenyl) imidazolium chloride (8.9 mg, 26 μmol, 13 mol%), potassium tert-butoxide (26.9 mg, 0.240 mmol, 1.2 equiv) and THF (1.0 mL). The crude product was purified by column chromatography on silica gel (hexane:EA 100:1 to 30:1) to give the product **2q** (41.3 mg, 68%) as a colorless oil. ^1H NMR (400 MHz, $CDCl_3$) δ 5.16 (s, 1H), 4.10 (d, J = 5.6 Hz, 2H), 3.10–2.97 (m, 1H), 2.89–2.81 (m, 1H), 2.72–2.50 (m, 3H), 1.24 (s, 12H), 1.20 (s, 9H). ^{13}C NMR (100 MHz, $CDCl_3$) δ 178.8, 165.1, 82.7, 67.7, 39.0, 37.3, 36.9, 29.0, 27.3, 24.99, 24.97 (the carbon bound to boron was not observed). ^{11}B NMR (128 MHz, $CDCl_3$) δ 29.4. HRMS (ESI+) calcd for $C_{17}H_{29}BNaO_4$ $[M+Na]^+$ 331.2051, found 331.2048.

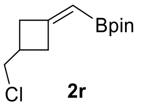

Cl **2r**

2-((3-(Chloromethyl)cyclobutylidene)methyl)-4,4,5,5-tetramethyl-1,3,2-dioxaborolane (2r): The borylative cyclization of alkyne was conducted by procedure F, using **1r** (57.2 mg, 0.200 mmol), B_2Pin_2 (61.0 mg, 0.240 mmol, 1.2 equiv), 1,3-bis(2,4,6-trimethylphenyl) imidazolium chloride (8.9 mg, 26 μmol, 13 mol%), potassium tert-butoxide (26.9 mg, 0.240 mmol, 1.2 equiv) and THF (1.0 mL). The crude product was purified by column chromatography on silica gel (hexane:EA 100:1 to 30:1) to give the product **2r** (30.5 mg, 63%) as a colorless oil.^1H NMR (400 MHz, $CDCl_3$) δ 5.21–5.17 (m, 1H), 3.60 (d, J = 6.8 Hz, 2H), 3.14–3.04 (m, 1H), 2.94–2.86 (m, 1H), 2.72–2.63 (m, 2H), 2.59–2.51 (m, 1H), 1.24 (s, 12H). ^{13}C NMR (100 MHz, $CDCl_3$) δ 163.7, 82.8, 49.5, 38.5, 38.2, 32.4, 25.0, 24.9 (the carbon bound to boron was not observed). ^{11}B NMR (128 MHz, $CDCl_3$) δ 29.7. HRMS (ESI+) calcd for $C_{12}H_{20}BClNaO_2$ $[M+Na]^+$ 267.1108, found 267.1107.

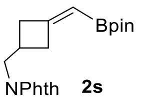

NPhth **2s**

2-((3-((4,4,5,5-Tetramethyl-1,3,2-dioxaborolan-2-yl)methylene)cyclobutyl)methyl)isoindoline-1,3-dione (2s):

The borylative cyclization of alkyne was conducted by procedure F, using **1s** (57.2 mg, 0.200 mmol), B_2Pin_2 (61.0 mg, 0.240 mmol, 1.2 equiv), 1,3-bis(2,4,6-trimethylphenyl) imidazolium chloride (8.9 mg, 26 μmol, 13 mol%), potassium tert-butoxide (26.9 mg, 0.240 mmol, 1.2 equiv) and THF (1.0 mL). The crude product was purified by column chromatography on silica gel (hexane:EA 30:1 to 20:1) to give the product **2s** (45.2 mg, 64%) as a white solid. ^{1}H NMR (400 MHz, $CDCl_3$) δ 7.87–7.80 (m, 2H), 7.76–7.67 (m, 2H), 5.15 (s, 1H), 3.80 (d, J = 6.4 Hz, 2H), 3.10–2.96 (m, 1H), 2.87–2.59 (m, 4H), 1.22 (s, 12H). ^{13}C NMR (100 MHz, $CDCl_3$) δ 168.7, 164.3, 134.0, 132.2, 123.4, 82.7, 43.1, 38.5, 38.1, 29.5, 24.99, 24.95 (the carbon bound to boron was not observed). ^{11}B NMR (128 MHz, $CDCl_3$) δ 29.8. HRMS (ESI+) calcd for $C_{20}H_{25}BNO_4$ $[M+H]^+$ 354.1871, found 354.1867.

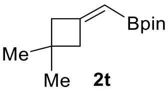

2t

2-((3,3-Dimethylcyclobutylidene)methyl)-4,4,5,5-tetramethyl-1,3,2-dioxaborolane (2t):

The borylative cyclization of alkyne was conducted by procedure F, using **1t** (53.2 mg, 0.200 mmol), B_2Pin_2 (61.0 mg, 0.240 mmol, 1.2 equiv), 1,3-bis(2,4,6-trimethylphenyl) imidazolium chloride (8.9 mg, 26 μmol, 13 mol%), potassium tert-butoxide (26.9 mg, 0.240 mmol, 1.2 equiv) and THF (1.0 mL). The crude product was purified by column chromatography on silica gel (hexane:EA 100:1 to 30:1) to give the product **2t** (28.4 mg, 64%) as a colorless oil. ^{1}H NMR (400 MHz, $CDCl_3$) δ 5.18 (s, 1H), 2.64 (s, 2H), 2.48 (s, 2H), 1.24 (s, 12H), 1.16 (s, 6H). ^{13}C NMR (100 MHz, $CDCl_3$) δ 165.2, 82.6, 47.7, 47.1, 31.1, 28.9, 25.0 (the carbon bound to boron was not observed). ^{11}B NMR (128 MHz, $CDCl_3$) δ 29.5. HRMS (ESI+) calcd for $C_{13}H_{24}BO_2$ $[M+H]^+$ 223.1864., found 223.1863.

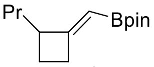

2u

(*E*)-4,4,5,5-Tetramethyl-2-((2-propylcyclobutylidene)methyl)-1,3,2-dioxaborolane (2u):

The borylative cyclization of alkyne was conducted by procedure F, using **1u** (56 mg, 0.20 mmol), B_2Pin_2 (61.0 mg, 0.240 mmol, 1.2 equiv), 1,3-bis(2,4,6-trimethylphenyl) imidazolium chloride (8.9 mg, 26 μmol, 13 mol%), potassium tert-butoxide (26.9 mg, 0.240 mmol, 1.2 equiv) and THF (1.0

mL). The crude product was purified by column chromatography on silica gel (hexane:EA 100:1 to 30:1) to give the product **2u** (37.7 mg, 80%) as a colorless oil. ^{1}H NMR (400 MHz, $CDCl_3$) δ 5.11 (q, J = 2.4 Hz, 1H), 3.02–2.85 (m, 1H), 2.89–2.70 (m, 2H), 2.15–2.02 (m, 1H), 1.67–1.51 (m, 2H), 1.41–1.29 (m, 3H), 1.24 (s, 12H), 0.88 (t, J = 7.2 Hz, 3H). ^{13}C NMR (100 MHz, $CDCl_3$) δ 174.5, 82.6, 47.0, 36.0, 31.6, 25.1, 25.0, 23.8, 20.3, 14.3 (the carbon bound to boron was not observed). ^{11}B NMR (128 MHz, $CDCl_3$) δ 29.3. HRMS (ESI+) calcd for $C_{14}H_{26}BO_2$ $[M+H]^+$ 237.2020, found 237.2018.

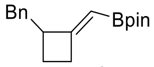

2v

(*E*)-2-((2-Benzylcyclobutylidene)methyl)-4,4,5,5-tetramethyl-1,3,2-dioxaborolane (2v):

The borylative cyclization of alkyne was conducted by procedure F, using **1v** (32.8 mg, 0.100 mmol), B_2Pin_2 (30.5 mg, 0.120 mmol, 1.2 equiv), 1,3-bis(2,4,6-trimethylphenyl) imidazolium chloride (4.4 mg, 13 μmol, 13 mol%), potassium tert-butoxide (13.5 mg, 0.120 mmol, 1.2 equiv) and THF (1.0 mL). The crude product was purified by column chromatography on silica gel (hexane:EA 100:1 to 30:1) to give the product **2v** (20.1 mg, 71%) as a colorless oil. ^{1}H NMR (400 MHz, $CDCl_3$) δ 7.23 (d, J = 5.6 Hz, 2H), 7.19–7.12 (m, 3H), 5.12 (q, J = 2.4 Hz, 1H), 3.32–3.22 (m, 1H), 2.95 (dd, J = 14.0, 6.0 Hz, 1H), 2.90–2.72 (m, 2H), 2.67 (dd, J = 14.0, 9.2 Hz, 1H), 2.11–2.00 (m, 1H), 1.76–1.64 (m, 1H), 1.23 (s, 12H). ^{13}C NMR (100 MHz, $CDCl_3$) δ 173.0, 140.5, 128.8, 128.4, 126.0, 82.7, 47.8, 39.8, 31.5, 25.1, 24.9, 23.9 (the carbon bound to boron was not observed). ^{11}B NMR (128 MHz, $CDCl_3$) δ 30.0. HRMS (ESI+) calcd for $C_{18}H_{26}BO_2$ $[M+H]^+$ 285.2020, found 285.2017.

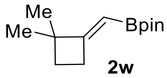

2w

(*E*)-2-((2,2-dimethylcyclobutylidene)methyl)-4,4,5,5-tetramethyl-1,3,2-dioxaborolane (2w):

The borylative cyclization of alkyne was conducted by procedure F, using **1w** (53.2 mg, 0.200 mmol), B_2Pin_2 (61.0 mg, 0.240 mmol, 1.2 equiv), 1,3-bis(2,4,6-trimethylphenyl) imidazolium chloride (8.9 mg, 26 μmol, 13 mol%), potassium tert-butoxide (27.0 mg, 0.240 mmol, 1.2 equiv) and THF (1.0 mL). The crude product was purified by column chromatography on silica gel (hexane:EA 100:1 to 50:1) to give the product **2w** (33.0 mg, 75%) as a colorless oil. ^{1}H NMR (400

MHz, $CDCl_3$) δ 5.08 (t, *J* = 2.4 Hz, 1H), 2.85 (td, *J* = 8.0, 2.4 Hz, 2H), 1.76 (t, *J* = 8.0 Hz, 2H), 1.24 (s, 12H), 1.13 (s, 6H). ^{13}C NMR (100 MHz, $CDCl_3$) δ 179.2, 82.7, 46.3, 32.0, 29.3, 27.1, 25.0 (the carbon bound to boron was not observed). ^{11}B NMR (128 MHz, $CDCl_3$) δ 29.8. HRMS (ESI+) calcd for $C_{13}H_{14}BO_2$ $[M+H]^+$ 223.1864, found 223.1869.

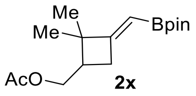

(*E*)-(2,2-dimethyl-3-((4,4,5,5-tetramethyl-1,3,2-dioxaborolan-2yl)methylene)cyclobutyl) methyl acetate (2x):

The borylative cyclization of alkyne was conducted by procedure F, using **1x** (33.8 mg, 0.100 mmol), B_2Pin_2 (30.5 mg, 0.120 mmol, 1.2 equiv), 1,3-bis(2,4,6-trimethylphenyl) imidazolium chloride (4.4 mg, 13 μmol, 13 mol%), potassium tert-butoxide (13.5 mg, 0.120 mmol, 1.2 equiv) and THF (1.0 mL). The crude product was purified by column chromatography on silica gel (hexane:EA 100:1 to 30:1) to give the product **2x** (19.0 mg, 66%) as a yellow oil. ^1H NMR (400 MHz, $CDCl_3$) δ 5.13 (s, 1H), 4.16–4.10 (m, 2H), 3.00 (dd, *J* = 16.8, 8.8 Hz, 1H), 2.56 (dd, *J* = 16.8, 6.4 Hz, 1H), 2.31 (p, *J* = 8.0 Hz, 1H), 2.03 (s, 3H), 1.24 (s, 12H), 1.17 (s, 3H), 1.07 (s, 3H). ^{13}C NMR (100 MHz, $CDCl_3$) δ 175.1, 171.3, 82.8, 65.6, 47.6, 39.8, 33.1, 27.8, 25.1, 25.0, 21.3, 21.1 (the carbon bound to boron was not observed). ^{11}B NMR (128 MHz, $CDCl_3$) δ 30.1. HRMS (ESI+) calcd for $C_{16}H_{27}BNaO_4$ $[M+Na]^+$ 317.1895, found 317.1898.

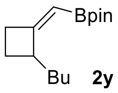

(*Z*)-2-((2-Butylcyclobutylidene)methyl)-4,4,5,5-tetramethyl-1,3,2-dioxaborolane (2y):

The borylative cyclization of alkyne was conducted by procedure F, using **1y** (58.8 mg, 0.200 mmol), B_2Pin_2 (61.0 mg, 0.240 mmol, 1.2 equiv), 1,3-bis(2,4,6-trimethylphenyl) imidazolium chloride (8.9 mg, 26 μmol, 13 mol%), potassium tert-butoxide (26.9 mg, 0.240 mmol, 1.2 equiv) and THF (1.0 mL). The crude product was purified by column chromatography on silica gel (hexane:EA 100:1 to 30:1) to give the product **2y** (27.0 mg, 54%) as a colorless oil. ^1H NMR (400 MHz, $CDCl_3$) δ 5.07 (d, *J* = 2.0 Hz, 1H), 3.11 (s, 1H), 2.85–2.70 (m, 1H), 2.66–2.54 (m, 1H), 2.08 (dt, *J* = 18.0, 9.2 Hz, 1H), 1.94–1.79 (m, 1H), 1.65–1.53 (m, 1H), 1.52–1.38 (m, 1H), 1.36–1.27 (m, 4H), 1.24 (d, *J* = 3.6

Hz, 12H), 0.90 (t, J = 6.8 Hz, 3H). ^{13}C NMR (100 MHz, $CDCl_3$) δ 174.1, 82.7, 46.4, 34.0, 32.1, 29.1, 25.1, 24.8, 22.7, 22.4, 14.2 (the carbon bound to boron was not observed). ^{11}B NMR (128 MHz, $CDCl_3$) δ 29.7. HRMS (ESI+) calcd for $C_{15}H_{28}BO_2$ $[M+H]^+$ 251.2177, found 251.2176.

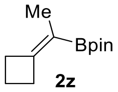

2z

2-(1-cyclobutylideneethyl)-4,4,5,5-tetramethyl-1,3,2-dioxaborolane (2z):

The borylative cyclization of alkyne was conducted by procedure F, using **1z** (50.4 mg, 0.200 mmol), B_2Pin_2 (61.0 mg, 0.240 mmol, 1.2 equiv), 1,3-bis(2,4,6-trimethylphenyl) imidazolium chloride (8.9 mg, 26 μmol, 13 mol%), potassium tert-butoxide (26.9 mg, 0.240 mmol, 1.2 equiv) and THF (1.0 mL). The crude product was purified by column chromatography on silica gel (hexane:EA 100:1 to 30:1) to give the product **2z** (34.0 mg, 83%) as a colorless oil. ^{1}H NMR (400 MHz, $CDCl_3$) δ 2.87 (t, J = 7.2 Hz, 2H), 2.70 (t, J = 7.6 Hz, 2H), 1.93 (p, J = 8.0 Hz, 2H), 1.54 (s, 3H), 1.23 (s, 12H). ^{13}C NMR (100 MHz, $CDCl_3$) δ 160.2, 82.7, 33.7, 31.5, 25.0, 16.6, 14.6 (the carbon bound to boron was not observed). ^{11}B NMR (128 MHz, $CDCl_3$) δ 29.8. HRMS (ESI+) calcd for $C_{12}H_{21}BNaO_2$ $[M+Na]^+$ 231.1527, found 231.1528.

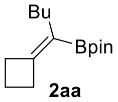

2aa

2-(1-cyclobutylidenepentyl)-4,4,5,5-tetramethyl-1,3,2-dioxaborolane (2aa):

The borylative cyclization of alkyne was conducted by procedure F, using **1aa** (58.8 mg, 0.200 mmol), B_2Pin_2 (61.0 mg, 0.240 mmol, 1.2 equiv), 1,3-bis(2,4,6-trimethylphenyl) imidazolium chloride (8.9 mg, 26 μmol, 13 mol%), potassium tert-butoxide (26.9 mg, 0.240 mmol, 1.2 equiv) and THF (1.0 mL). The crude product was purified by column chromatography on silica gel (hexane:EA 100:1 to 30:1) to give the product **2aa** (31.0 mg, 62%) as a colorless oil. ^{1}H NMR (400 MHz, $CDCl_3$) δ 2.88 (t, J = 7.6 Hz, 2H), 2.71 (t, J = 7.6 Hz, 2H), 2.00–1.86 (m, 4H), 1.33–1.25 (m, 4H), 1.23 (s, 12H), 0.91–0.83 (m, 3H). ^{13}C NMR (100 MHz, $CDCl_3$) δ 159.4, 82.6, 33.7, 32.5, 31.5, 29.5, 25.0, 22.7, 16.8, 14.3 (the carbon bound to boron was not observed). ^{11}B NMR (128 MHz, $CDCl_3$) δ 30.5. HRMS (ESI+) calcd for $C_{15}H_{28}BO_2$ $[M+H]^+$ 251.2177, found 251.2175.

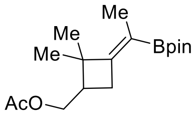

2ab

(*Z*)-(2,2-dimethyl-3-(1-(4,4,5,5-tetramethyl-1,3,2-dioxaborolan-2-yl)ethylidene)cyclobutyl) methyl acetate (2ab):

The borylative cyclization of alkyne was conducted by procedure F, using **1ab** (70.4 mg, 0.200 mmol), B_2Pin_2 (61.0 mg, 0.240 mmol, 1.2 equiv), 1,3-bis(2,4,6-trimethylphenyl) imidazolium chloride (8.8 mg, 26 μmol, 13 mol%), potassium tert-butoxide (27.0 mg, 0.240 mmol, 1.2 equiv) and THF (1.0 mL). The crude product was purified by column chromatography on silica gel (hexane:EA 100:1 to 30:1) to give the product **2ab** (70.0 mg, 88%) as a yellow oil. ^{1}H NMR (400 MHz, $CDCl_3$) δ 4.21–4.01 (m, 2H), 2.90 (dd, J = 17.2, 9.2 Hz, 1H), 2.42 (dd, J = 16.0, 6.8 Hz, 1H), 2.23 (p, J = 8.0 Hz, 1H), 2.04 (s, 3H), 1.65 (s, 3H), 1.30 (s, 3H), 1.23 (s, 12H), 1.19 (s, 3H). ^{13}C NMR (100 MHz, $CDCl_3$) δ 171.3, 163.1, 82.8, 65.8, 47.2, 39.3, 31.9, 27.8, 25.1, 25.0, 21.2, 20.4, 14.4 (the carbon bound to boron was not observed). ^{11}B NMR (128 MHz, $CDCl_3$) δ 30.1. HRMS (ESI+) calcd for $C_{17}H_{29}BNaO_4$ $[M+Na]^+$ 331.2051, found 331.2055.

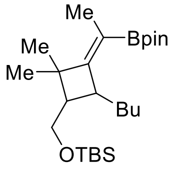

2ac

(*Z*)-tert-butyl((4-butyl-2,2-dimethyl-3-(1-(4,4,5,5-tetramethyl-1,3,2-dioxaborolan-2-yl)ethylidene)cyclobutyl)methoxy)dimethylsilane (2ac):

The borylative cyclization of alkyne was conducted by procedure F, using **1ac** (40 mg, 0.10 mmol), B_2Pin_2 (30.5 mg, 0.120 mmol, 1.20 equiv), 1,3-bis(2,4,6-trimethylphenyl) imidazolium chloride (4.4 mg, 13 µmol, 13 mol%), potassium tert-butoxide (13.5 mg, 0.120 mmol, 1.20 equiv) and THF (1.0 mL). The crude product was purified by column chromatography on silica gel (hexane:EA 100:1 to 30:1) to give the product **2ac** (28 mg, 64%) as a colorless oil. ^{1}H NMR (400 MHz, $CDCl_3$) δ 3.78–3.65 (m, 1H), 3.64–3.53 (m, 1H), 2.55–2.44 (m, 1H), 2.00–1.87 (m, 1H), 1.81–1.71 (m, 1H), 1.67 (s, 3H), 1.43–1.29 (m, 3H), 1.30 (s, 3H), 1.28–1.22 (m, 2H), 1.24 (d, J = 2.8 Hz, 12H), 1.20 (s, 3H), 0.92 – 0.87 (m, 3H), 0.89 (s, 3H), 0.04 (s, 6H). ^{13}C NMR (100 MHz, $CDCl_3$) δ 169.0, 82.8,

64.4, 49.9, 44.2, 43.6, 35.5, 29.6, 29.0, 26.1, 25.2, 24.9, 22.9, 20.8, 18.3, 15.1, 14.3, -5.2, -5.3. ^{11}B NMR (128 MHz, $CDCl_3$) δ 30.5. HRMS (ESI+) calcd for $C_{25}H_{49}BNaO_3Si$ $[M+Na]^+$ 459.3436, found 459.3437.

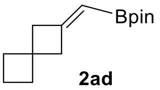

2ad

4,4,5,5-Tetramethyl-2-(spiro[3.3]heptan-2-ylidenemethyl)-1,3,2-dioxaborolane (2ad):

The borylative cyclization of alkyne was conducted by procedure F, using **1ad** (55.6 mg, 0.200 mmol), B_2Pin_2 (61.0 mg, 0.240 mmol, 1.2 equiv), 1,3-bis(2,4,6-trimethylphenyl) imidazolium chloride (8.9 mg, 26 μmol, 13 mol%), potassium tert-butoxide (26.9 mg, 0.240 mmol, 1.2 equiv) and THF (1.0 mL). The crude product was purified by column chromatography on silica gel (hexane:EA 100:1 to 30:1) to give the product **2ad** (31.3 mg, 67%) as a yellow oil. ^{1}H NMR (400 MHz, $CDCl_3$) δ 5.12 (p, J = 2.4 Hz, 1H), 2.89 (d, J = 2.4 Hz, 2H), 2.74–2.71 (m, 2H), 2.04–1.97 (m, 4H), 1.86–1.78 (m, 2H), 1.24 (s, 12H). ^{13}C NMR (100 MHz, $CDCl_3$) δ 165.5, 82.6, 47.3, 46.9, 38.9, 34.7, 25.0, 16.5 (the carbon bound to boron was not observed). ^{11}B NMR (128 MHz, $CDCl_3$) δ 29.5. HRMS (ESI+) calcd for $C_{14}H_{24}BO_2$ $[M+H]^+$ 235.1864, found 235.1862.

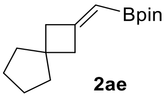

2ae

4,4,5,5-Tetramethyl-2-(spiro[3.4]octan-2-ylidenemethyl)-1,3,2-dioxaborolane (2ae):

The borylative cyclization of alkyne was conducted by procedure F, using **1ae** (58.4 mg, 0.200 mmol), B_2Pin_2 (61.0 mg, 0.240 mmol, 1.2 equiv), 1,3-bis(2,4,6-trimethylphenyl) imidazolium chloride (8.9 mg, 26 μmol, 13 mol%), potassium tert-butoxide (26.9 mg, 0.240 mmol, 1.2 equiv) and THF (1.0 mL). The crude product was purified by column chromatography on silica gel (hexane:EA 100:1 to 30:1) to give the product **2ae** (37.2 mg, 75%) as a colorless oil. ^{1}H NMR (400 MHz, $CDCl_3$) δ 5.15 (p, J = 2.4 Hz, 1H), 2.75 (d, J = 2.0 Hz, 2H), 2.64–2.58 (m, 2H), 1.63–1.57 (m, 8H), 1.23 (s, 12H). ^{13}C NMR (100 MHz, $CDCl_3$) δ 165.9, 82.6, 45.9, 45.5, 42.1, 39.3, 25.0, 24.1 (the carbon bound to boron was not observed). ^{11}B NMR (128 MHz, $CDCl_3$) δ 29.3. HRMS (ESI+) calcd for $C_{15}H_{26}BO_2$ $[M+H]^+$ 249.2020, found 249.2017.

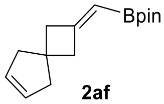

2af

4,4,5,5-Tetramethyl-2-(spiro[3.4]oct-6-en-2-ylidenemethyl)-1,3,2-dioxaborolane (2af):

The borylative cyclization of alkyne was conducted by procedure F, using **1af** (58.0 mg, 0.200 mmol), B_2Pin_2 (61.0 mg, 0.240 mmol, 1.20 equiv), 1,3-bis(2,4,6-trimethylphenyl) imidazolium chloride (8.9 mg, 26 µmol, 13 mol%), potassium tert-butoxide (26.9 mg, 0.240 mmol, 1.20 equiv) and THF (1.0 mL). The crude product was purified by column chromatography on silica gel (hexane:EA 100:1 to 30:1) to give the product **2af** (35.9 mg, 73%)as a colorless oil. ^{1}H NMR (400 MHz, $CDCl_3$) δ 5.67 (s, 2H), 5.20–5.16 (m, 1H), 2.87 (d, J = 2.4 Hz, 2H), 2.72 (s, 2H), 2.45 (s, 4H), 1.24 (s, 12H). ^{13}C NMR (100 MHz, $CDCl_3$) δ 165.4, 130.0, 82.7, 47.8, 47.3, 46.8, 40.5, 25.0 (the carbon bound to boron was not observed). ^{11}B NMR (128 MHz, $CDCl_3$) δ 29.4. HRMS (ESI+) calcd for $C_{15}H_{22}BO_2$ $[M-H]^-$ 245.1707, found 245.1706.

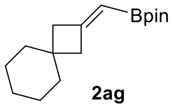

2ag

4,4,5,5-Tetramethyl-2-(spiro[3.5]nonan-2-ylidenemethyl)-1,3,2-dioxaborolane (2ag):

The borylative cyclization of alkyne was conducted by procedure F, using **1ag** (61.2 mg, 0.200 mmol), B_2Pin_2 (61.0 mg, 0.240 mmol, 1.20 equiv), 1,3-bis(2,4,6-trimethylphenyl) imidazolium chloride (8.9 mg, 26 µmol, 13 mol%), potassium tert-butoxide (26.9 mg, 0.240 mmol, 1.20 equiv) and THF (1.0 mL). The crude product was purified by column chromatography on silica gel (hexane:EA 100:1 to 30:1) to give the product **2ag** (43.5 mg, 83%) as a colorless oil. ^{1}H NMR (400 MHz, $CDCl_3$) δ 5.17 (p, J = 2.4 Hz, 1H), 2.60–2.54 (m, 2H), 2.42 (s, 2H), 1.47 (d, J = 5.6 Hz, 4H), 1.43–1.35 (m, 6H), 1.24 (s, 12H). ^{13}C NMR (100 MHz, $CDCl_3$) δ 166.1, 82.6, 45.6, 45.1, 37.9, 35.4, 26.0, 25.0, 23.5 (the carbon bound to boron was not observed). ^{11}B NMR (128 MHz, $CDCl_3$) δ 29.5. HRMS (ESI+) calcd for $C_{16}H_{28}BO_2$ $[M+H]^+$ 263.2177, found 263.2175.

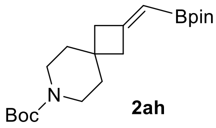

2ah

Tert-butyl 2-((4,4,5,5-tetramethyl-1,3,2-dioxaborolan-2-yl)methylene)-7-azaspiro[3.5] nonane-7-carboxylate (2ah):

The borylative cyclization of alkyne was conducted by procedure F, using **1ah** (81.4 mg, 0.200 mmol), B_2Pin_2 (61.0 mg, 0.240 mmol, 1.20 equiv), 1,3-bis(2,4,6-trimethylphenyl) imidazolium chloride (8.9 mg, 26 μmol, 13 mol%), potassium tert-butoxide (26.9 mg, 0.240 mmol, 1.20 equiv) and THF (1.0 mL). The crude product was purified by column chromatography on silica gel (hexane:EA 100:1 to 30:1) to give the product **2ah** (43.6 mg, 60%) as a colorless oil. ^{1}H NMR (400 MHz, $CDCl_3$) δ 5.58–4.91 (m, 1H), 3.39–3.26 (m, 4H), 2.65 (s, 2H), 2.50 (s, 2H), 1.55 (t, J = 5.2 Hz, 4H), 1.45 (s, 9H), 1.24 (s, 12H). ^{13}C NMR (100 MHz, $CDCl_3$) δ 163.8, 155.1, 82.7, 79.4, 44.6, 44.0, 36.8, 33.8, 28.6, 25.0 (the carbon bound to boron was not observed). ^{11}B NMR (128 MHz, $CDCl_3$) δ 29.8. HRMS (ESI+) calcd for $C_{20}H_{34}BNNaO_4$ [M+Na]$^+$ 386.2473, found 386.2469.

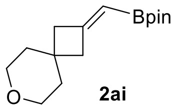

2ai

2-((7-Oxaspiro[3.5]nonan-2-ylidene)methyl)-4,4,5,5-tetramethyl-1,3,2-dioxaborolane (2ai): The borylative cyclization of alkyne was conducted by procedure F, using **1ai** (61.6 mg, 0.200 mmol), B_2Pin_2 (61.0 mg, 0.240 mmol, 1.20 equiv), 1,3-bis(2,4,6-trimethylphenyl) imidazolium chloride (8.9 mg, 26 μmol, 13 mol%), potassium tert-butoxide (26.9 mg, 0.240 mmol, 1.20 equiv) and THF (1.0 mL). The crude product was purified by column chromatography on silica gel (hexane:EA 100:1 to 30:1) to give the product **2ai** (32.7 mg, 62%) as a yellow oil. ^{1}H NMR (400 MHz, $CDCl_3$) δ 5.22 (s, 1H), 3.71–3.49 (m, 4H), 2.68 (s, 2H), 2.53 (s, 2H), 1.66–1.58 (m, 4H), 1.24 (s, 12H). ^{13}C NMR (100 MHz, $CDCl_3$) δ 163.9, 82.8, 65.3, 45.2, 44.7, 37.8, 32.9, 29.8, 25.0 (the carbon bound to boron was not observed). ^{11}B NMR (128 MHz, $CDCl_3$) δ 29.7. HRMS (ESI+) calcd for $C_{15}H_{26}BO_3$ [M+H]$^+$ 265.1970, found 265.1968.

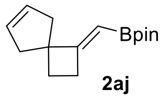

2aj

(*E*)-4,4,5,5-tetramethyl-2-(spiro[3.4]oct-6-en-1-ylidenemethyl)-1,3,2-dioxaborolane (2aj): The borylative cyclization of alkyne was conducted by procedure F, using **1aj** (29.0 mg, 0.100 mmol), B_2Pin_2 (30.5 mg, 0.120 mmol, 1.20 equiv), 1,3-bis(2,4,6-trimethylphenyl) imidazolium chloride (4.4 mg, 13 μmol, 13 mol%), potassium tert-butoxide (13.5 mg, 0.120 mmol, 1.20 equiv) and THF (1.0 mL). The crude product was purified by column chromatography on silica gel

(hexane:EA 100:1 to 30:1) to give the product **2aj** (18.0 mg, 73%) as a yellow oil. ^{1}H NMR (400 MHz, $CDCl_3$) δ 5.60 (s, 2H), 5.15 (s, 1H), 2.84 (t, J = 8.0 Hz, 2H), 2.62 (d, J = 15.2 Hz, 2H), 2.44 (d, J = 15.2 Hz, 2H), 1.98 (t, J = 8.0 Hz, 2H), 1.24 (s, 12H). ^{13}C NMR (100 MHz, $CDCl_3$) δ 178.2, 129.1, 82.7, 55.2, 46.2, 33.7, 30.3, 25.0 (the carbon bound to boron was not observed). ^{11}B NMR (128 MHz, $CDCl_3$) δ 29.8. HRMS (ESI+) calcd for $C_{15}H_{24}BO_2$ $[M+H]^+$ 247.1864, found 247.1870.

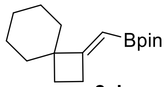

2ak

(*E*)-4,4,5,5-tetramethyl-2-(spiro[3.5]nonan-1-ylidenemethyl)-1,3,2-dioxaborolane (2ak):

The borylative cyclization of alkyne was conducted by procedure F, using **1ak** (30.6 mg, 0.100 mmol), B_2Pin_2 (30.5 mg, 0.120 mmol, 1.20 equiv), 1,3-bis(2,4,6-trimethylphenyl) imidazolium chloride (4.4 mg, 13 μmol, 13 mol%), potassium tert-butoxide (13.5 mg, 0.120 mmol, 1.20 equiv) and THF (1.0 mL). The crude product was purified by column chromatography on silica gel (hexane:EA 100:1 to 30:1) to give the product **2ak** (17.0 mg, 65%) as a yellow oil. ^{1}H NMR (400 MHz, $CDCl_3$) δ 5.15 (s, 1H), 2.80 (t, J = 8.0 Hz, 2H), 1.75 (t, J = 8.0 Hz, 2H), 1.61–1.51 (m, 4H), 1.50–1.38 (m, 3H), 1.36–1.27 (m, 3H), 1.24 (s, 12H). ^{13}C NMR (100 MHz, $CDCl_3$) δ 179.2, 82.6, 51.1, 36.1, 29.4, 28.8, 26.0, 25.0, 22.7 (the carbon bound to boron was not observed). ^{11}B NMR (128 MHz, $CDCl_3$) δ 29.8. HRMS (ESI+) calcd for $C_{16}H_{28}BO_2$ $[M+H]^+$ 263.2177, found 263.2182.

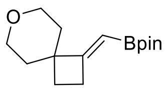

2al

(*E*)-2-((7-oxaspiro[3.5]nonan-1-ylidene)methyl)-4,4,5,5-tetramethyl-1,3,2-dioxaborolane (2al):

The borylative cyclization of alkyne was conducted by procedure F, using **1al** (30.8 mg, 0.100 mmol), B_2Pin_2 (30.5 mg, 0.120 mmol, 1.20 equiv), 1,3-bis(2,4,6-trimethylphenyl) imidazolium chloride (4.4 mg, 13 μmol, 13 mol%), potassium tert-butoxide (13.5 mg, 0.120 mmol, 1.20 equiv) and THF (1.0 mL). The crude product was purified by column chromatography on silica gel (hexane:EA 100:1 to 30:1) to give the product **2al** (19.0 mg, 72%) as a yellow oil. ^{1}H NMR (400 MHz, $CDCl_3$) δ 5.22 (t, J = 2.4 Hz, 1H), 3.79 (dt, J = 11.6, 4.0 Hz, 2H), 3.53–3.43 (m, 2H), 2.85 (td, J = 8.0, 2.4 Hz, 2H), 1.87 (t, J = 8.0 Hz, 2H), 1.80–1.71 (m, 2H), 1.55 (d, J = 13.6 Hz, 2H), 1.24

(s, 12H). ^{13}C NMR (100 MHz, CDCl$_3$) δ 176.4, 82.8, 64.7, 48.0, 36.2, 29.2, 28.7, 25.0 (the carbon bound to boron was not observed). ^{11}B NMR (128 MHz, CDCl$_3$) δ 30.0. HRMS (ESI+) calcd for $C_{15}H_{26}BO_3$ [M+H]$^+$ 265.1970, found 265.1973.

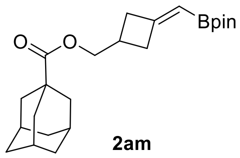

2am

(3-((4,4,5,5-Tetramethyl-1,3,2-dioxaborolan-2-yl)methylene)cyclobutyl)methyl (3r,5r,7r)-adamantane-1-carboxylate (2am):

The borylative cyclization of alkyne was conducted by procedure F, using **1am** (43 mg, 0.10 mmol), B_2Pin_2 (30.5 mg, 0.120 mmol, 1.20 equiv), 1,3-bis(2,4,6-trimethylphenyl) imidazolium chloride (4.4 mg, 13 μmol, 13 mol%), potassium tert-butoxide (13.5 mg, 0.120 mmol, 1.20 equiv) and THF (1.0 mL). The crude product was purified by column chromatography on silica gel (hexane:EA 100:1 to 30:1) to give the product **2am** (27.0 mg, 70%) as a white solid. ^{1}H NMR (400 MHz, CDCl$_3$) δ 5.15 (t, J = 2.4 Hz, 1H), 4.08 (d, J = 6.0 Hz, 2H), 3.09–2.95 (m, 1H), 2.89–2.79 (m, 1H), 2.71–2.49 (m, 3H), 2.00 (s, 3H), 1.88 (d, J = 2.8 Hz, 6H), 1.75–1.65 (m, 6H), 1.24 (s, 12H). ^{13}C NMR (100 MHz, CDCl$_3$) δ 177.9, 165.3, 82.7, 67.4, 41.0, 39.0, 37.3, 36.9, 36.7, 29.1, 28.1, 25.0 (the carbon bound to boron was not observed). ^{11}B NMR (128 MHz, CDCl$_3$) δ 30.5. HRMS (ESI+) calcd for $C_{23}H_{36}BO_4$ [M+H]$^+$ 387.2701, found 387.2702.

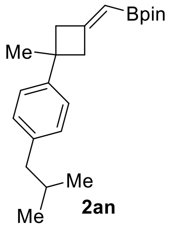

2an

2-((3-(4-Isobutylphenyl)-3-methylcyclobutylidene)methyl)-4,4,5,5-tetramethyl-1,3,2-dioxaborolane (2an):

The borylative cyclization of alkyne was conducted by procedure F, using **1an** (76.8 mg, 0.200 mmol), B_2Pin_2 (61.0 mg, 0.240 mmol, 1.20 equiv), 1,3-bis(2,4,6-trimethylphenyl) imidazolium chloride (8.9 mg, 26 μmol, 13 mol%), potassium tert-butoxide (26.9 mg, 0.240 mmol, 1.20 equiv) and THF (1.0 mL). The crude product was purified by column chromatography on silica gel

(hexane:EA 100:1 to 30:1) to give the product **2an** (44.2 mg, 65%) as a colorless oil. ^{1}H NMR (400 MHz, CDCl$_3$) δ 7.16 (d, J = 8.0 Hz, 2H), 7.09 (d, J = 8.0 Hz, 2H), 5.36–5.13 (m, 1H), 3.24 (dt, J = 16.4, 2.8 Hz, 1H), 3.15 (dt, J = 16.0, 2.4 Hz, 1H), 3.01 (dq, J = 16.4, 3.2 Hz, 1H), 2.85–2.74 (m, 1H), 2.45 (d, J = 7.2 Hz, 2H), 1.86 (dp, J = 13.6, 6.8 Hz, 1H), 1.46 (s, 3H), 1.26 (d, J = 1.6 Hz, 12H), 0.91 (d, J = 6.4 Hz, 6H). ^{13}C NMR (100 MHz, CDCl$_3$) δ 164.3, 148.1, 138.8, 129.0, 125.1, 82.7, 47.7, 47.2, 45.2, 38.4, 30.8, 30.4, 25.03, 25.00, 22.6 (the carbon bound to boron was not observed). ^{11}B NMR (128 MHz, CDCl$_3$) δ 29.4. HRMS (ESI+) calcd for $C_{22}H_{34}BO_2$ [M+H]$^+$ 341.2646, found 341.2646.

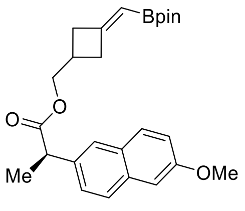

2ao

(3-((4,4,5,5-Tetramethyl-1,3,2-dioxaborolan-2-yl)methylene)cyclobutyl)methyl (2R)-2-(6-methoxynaphthalen-2-yl)propanoate (2ao):

The borylative cyclization of alkyne was conducted by procedure F, using **1ao** (48 mg, 0.10 mmol), B$_2$Pin$_2$ (30.5 mg, 0.120 mmol, 1.20 equiv), 1,3-bis(2,4,6-trimethylphenyl) imidazolium chloride (4.4 mg, 13 μmol, 13 mol%), potassium tert-butoxide (13.5 mg, 0.120 mmol, 1.20 equiv) and THF (1.0 mL). The crude product was purified by column chromatography on silica gel (hexane:EA 100:1 to 30:1) to give the product **2ao** (22.7 mg, 52%) as a white solid. ^{1}H NMR (400 MHz, CDCl$_3$) δ 7.71 (d, J = 3.2 Hz, 1H), 7.68 (d, J = 2.8 Hz, 1H), 7.66 (s, 1H), 7.40 (dd, J = 8.4, 1.6 Hz, 1H), 7.18–7.07 (m, 2H), 5.16–5.07 (m, 1H), 4.16–4.07 (m, 2H), 3.91 (s, 3H), 3.86 (q, J = 7.2 Hz, 1H), 3.01–2.91 (m, 1H), 2.81–2.71 (m, 1H), 2.65–2.53 (m, 2H), 2.50–2.42 (m, 1H), 1.58 (d, J = 7.2 Hz, 3H), 1.22 (s, 12H). ^{13}C NMR (100 MHz, CDCl$_3$) δ 174.9, 165.0, 157.7, 135.9, 133.8, 129.4, 129.1, 127.2, 126.4, 126.0, 119.1, 105.7, 82.7, 68.3, 68.2, 55.4, 45.6, 37.3, 37.0, 36.9, 28.9, 25.00, 24.95, 18.6 (the carbon bound to boron was not observed). ^{11}B NMR (128 MHz, CDCl$_3$) δ 30.1. HRMS (ESI+) calcd for $C_{26}H_{34}BO_5$ [M+H]$^+$ 437.2494, found 437.2489.

5. Transformations of the BMCBs

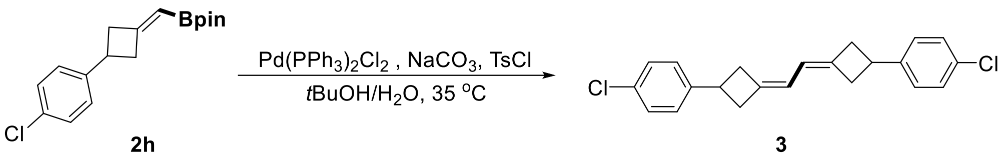

Compound 3:

To a 5-mL screw-up vial were added **2h** (60.8 mg, 0.200 mmol), tosyl chloride (19 mg, 0.10 mmol), Na_2CO_3 (42.4 mg, 0.400 mmol), $Pd(PPh_3)_2Cl_2$ (14 mg, 0.020 mmol), and *t*-BuOH (1.0 mL) under Ar, followed by the addition of H_2O (1.0 mL) through a syringe, and the mixture was stirred at 35 °C for 12 h under Ar. After the reaction mixture was cooled to room temperature, the volatiles were evaporated under reduced pressure, and the residue was purified by column chromatography on silica gel (hexane:EA 100:1) to give the product **3** (28.3 mg, 80%) as a white solid. ^1H NMR (400 MHz, $CDCl_3$) δ 7.28 (d, J = 8.4 Hz, 4H), 7.20 (d, J = 8.0 Hz, 4H), 5.82 (s, 2H), 3.54 (q, J = 8.4 Hz, 2H), 3.30–3.08 (m, 4H), 2.92–2.70 (m, 4H). ^{13}C NMR (100 MHz, $CDCl_3$) δ 144.4, 136.9, 131.8, 128.6, 128.0, 118.1, 39.3, 37.7, 34.98, 34.95.

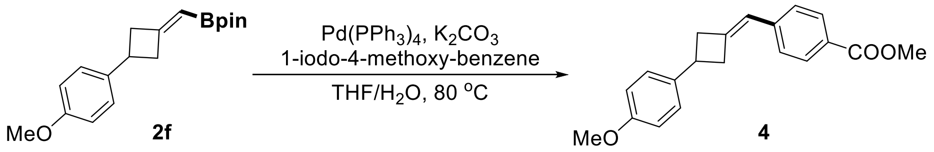

Compound 4:

To a 5-mL screw-up vial were added **2f** (60 mg, 0.20 mmol), 1-iodo-4-methoxy-benzene (0.14 g, 0.60 mmol), K_2CO_3 (84 mg, 0.60 mmol), $Pd(PPh_3)_4$ (22 mg, 20 μmol), and THF (1.8 mL) under Ar, followed by the addition of H_2O (0.2 mL) through a syringe. The mixture was stirred at 80 °C for 20 h under Ar. After the reaction mixture was cooled to room temperature, the volatiles were evaporated under reduced pressure, and the residue was purified by column chromatography on silica gel (hexane:EA 50:1) to give the product **4** (43.1 mg, 70%) as a yellow solid. ^1H NMR (400 MHz, $CDCl_3$) δ 7.97 (d, J = 8.4 Hz, 2H), 7.28 (d, J = 8.4 Hz, 2H), 7.23 (d, J = 8.4 Hz, 2H), 6.88 (d, J = 8.8 Hz, 2H), 6.36–6.15 (m, 1H), 3.90 (s, 3H), 3.80 (s, 3H), 3.66 (p, J = 8.0 Hz, 1H), 3.54–3.40

(m, 1H), 3.35–3.24 (m, 1H), 3.24–3.11 (m, 1H), 3.09–2.97 (m, 1H). ^{13}C NMR (100 MHz, $CDCl_3$) δ 167.1, 158.2, 144.1, 142.6, 137.4, 129.9, 127.5, 127.0, 121.2, 114.0, 55.4, 52.1, 41.0, 35.8. HRMS (ESI+) calcd for $C_{20}H_{21}O_3$ $[M+H]^+$ 309.1485, found 309.1481.

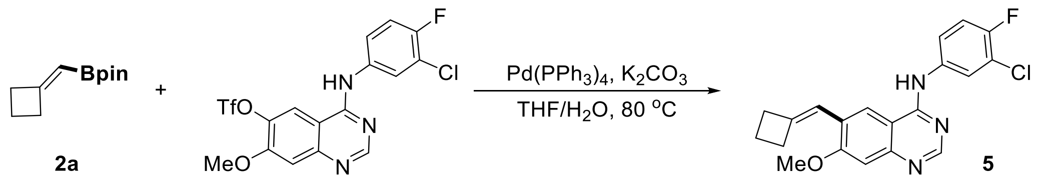

Compound 5:

To a 5-mL screw-up vial were added **2a** (19.4 mg, 100 µmol), Ar-OTf (31.6 mg, 70.0 µmol), K_2CO_3 (29 mg, 0.21 mmol), $Pd(PPh_3)_4$ (8.1 mg, 7.0 µmol), and dioxane (0.8 mL) under Ar, followed by the addition of H_2O (0.2 mL) through a syringe. The mixture was stirred at 120 °C for 20 h under Ar. After the reaction mixture was cooled to room temperature, the volatiles were evaporated under reduced pressure, and the residue was purified by column chromatography on silica gel (hexane:EA 2:1) to give the product **5** (19.4 mg, 75%) as a white solid. ^{1}H NMR (400 MHz, $CDCl_3$) δ 8.64 (s, 1H), 7.87 (dd, J = 6.4, 2.4 Hz, 1H), 7.58–7.45 (m, 2H), 7.23–7.10 (m, 3H), 6.53–6.45 (m, 1H), 3.95 (s, 3H), 3.05 (t, J = 7.6 Hz, 2H), 2.94 (t, J = 7.6 Hz, 2H), 2.15 (p, J = 7.6 Hz, 2H). ^{13}C NMR (100 MHz, $CDCl_3$) δ 160.8, 156.8, 156.2 (d, J = 244.0 Hz), 154.6, 150.9, 147.7, 135.2, 128.9, 124.2, 121.8 (d, J = 6.8 Hz), 121.3 (d, J = 18.6 Hz), 118.2, 116.8 (d, J = 22.0 Hz), 114.4, 109.1, 106.8, 56.0, 33.1, 33.0, 18.6. ^{19}F NMR (376 MHz, $CDCl_3$) δ -120.6. HRMS (ESI+) calcd for $C_{20}H_{18}ClFN_3O$ $[M+H]^+$ 370.1117, found 370.1115.

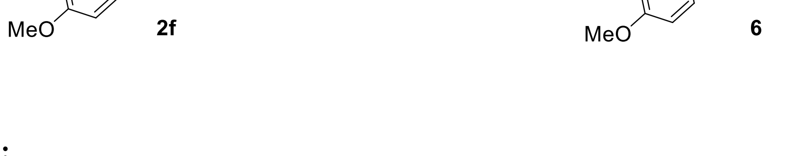

Compound 6:

To a 5-mL screw-up vial were added **2f** (60 mg, 0.20 mmol), Cu_2O (2.8 mg, 20 µmol), KI (166 mg, 1.00 mmol), 25% NH_3•H_2O (38 µL, 0.50 mmol of NH_3) and H_2O (1.0 mL). The mixture was stirred at rt for 24 h under air. After the reaction was completed, all volatiles were evaporated and the

residue was purified by column chromatography on silica gel (hexane:EA 50:1) to give the product **6** (27.6 mg, 46%) as a colorless oil. ^{1}H NMR (400 MHz, $CDCl_3$) δ 7.19 (t, J = 8.0 Hz, 2H), 6.91–6.84 (m, 2H), 5.84 (p, J = 2.4 Hz, 0.66H), 4.84 (p, J = 2.4 Hz, 0.34H), 3.81 (s, 3H), 3.48 (p, J = 8.0 Hz, 1H), 3.18–3.04 (m, 1.32H), 3.03–2.91 (m, 0.68H), 2.87–2.59 (m, 2H). ^{13}C NMR (100 MHz, $CDCl_3$) δ 158.3 and 158.0, 149.5 and 146.3, 138.0 and 136.9, 127.6 and 127.5, 114.0 and 113.9, 105.7, 69.0 and 55.4, 43.3, 40.2, 40.1, 34.3 and 32.2. HRMS (ESI+) calcd for $C_{12}H_{14}IO$ [M+H]$^+$ 301.0084, found 301.0084.

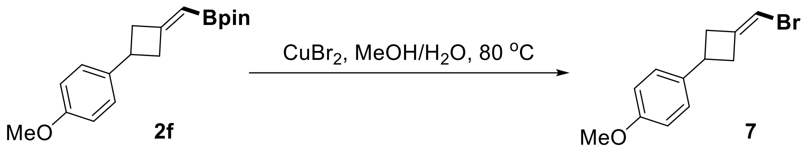

Compound 7:

To a solution of $CuBr_2$ (66 mg, 0.30 mmol) in MeOH/H_2O (1:1) (1.0 mL) was added **2f** (30 mg, 0.10 mmol), and the resulting reaction mixture was stirred for 24 h at 80 °C. After the mixture was cooled to room temperature, the volatiles were evaporated under reduced pressure, and the residue was purified by column chromatography on silica gel (hexane:EA 50:1) to give the product **7** (18.6 mg, 74%) as a colorless oil. ^{1}H NMR (400 MHz, $CDCl_3$) δ 7.19 (d, J = 8.4 Hz, 2H), 6.87 (d, J = 8.8 Hz, 2H), 5.92–5.86 (m, 1H), 3.81 (s, 3H), 3.52 (p, J = 8.4 Hz, 1H), 3.16–3.05 (m, 2H), 2.84–2.63 (m, 2H). ^{13}C NMR (100 MHz, $CDCl_3$) δ 158.3, 142.4, 137.0, 127.6, 114.0, 97.0, 55.5, 39.5, 39.1, 33.5. HRMS (ESI+) calcd for $C_{12}H_{14}BrO$ [M+H]$^+$ 253.0223, found 253.0221.

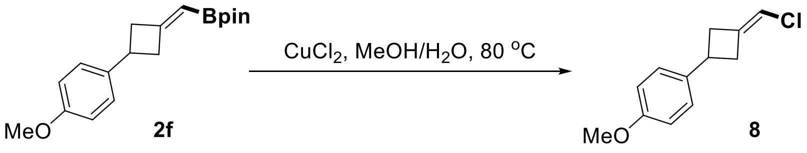

Compound 8:

To a solution of $CuCl_2$ (41 mg, 0.30 mmol) in MeOH/H_2O (1:1) (1.0 mL) was added **2f** (30 mg, 0.10 mmol), and the resulting reaction mixture was stirred for 24 h at 80 °C. After the mixture was cooled to room temperature, the volatiles were evaporated under reduced pressure, and the residue

was purified by column chromatography on silica gel (hexane:EA 50:1) to give the product **8** (14.6 mg, 70%) as a yellow oil. ^{1}H NMR (400 MHz, $CDCl_3$) δ 7.19 (d, J = 8.4 Hz, 2H), 6.88 (d, J = 8.4 Hz, 2H), 5.83 (s, 1H), 3.81 (s, 3H), 3.53 (p, J = 8.0 Hz, 1H), 3.24–3.03 (m, 2H), 2.88–2.72 (m, 2H). ^{13}C NMR (100 MHz, $CDCl_3$) δ 158.3, 139.2, 137.1, 127.5, 114.0, 108.9, 55.5, 37.9, 37.5, 34.2. HRMS (ESI+) calcd for $C_{12}H_{14}ClO$ $[M+H]^+$ 209.0728, found 209.0727.

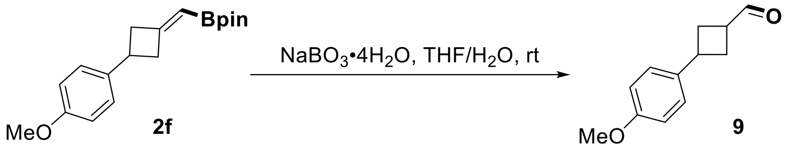

Compound 9:

To a solution of sodium perborate tetrahydrate (154 mg, 1.00 mmol) in THF/H_2O (1:1) (2.0 mL) was added **2f** (60 mg, 0.20 mmol), and the resulting reaction mixture was stirred for 3h at room temperature. After the mixture was completed, the volatiles were evaporated under reduced pressure, and the residue was purified by column chromatography on silica gel (hexane:EA 20:1) to give product **9** (25.1 mg, 66%) as a mixture of two inseparable diastereoisomers (d.r. = 1:1). ^{1}H NMR (400 MHz, $CDCl_3$) δ 9.95 (d, J = 1.6 Hz, 0.5H), 9.73 (d, J = 2.0 Hz, 0.5H), 7.15 (t, J = 8.0 Hz, 2H), 7.00–6.71 (m, 2H), 3.80 (s, 1.5H), 3.79 (s, 1.5H), 3.59–3.45 (m, 1H), 3.23–3.08 (m, 1H), 2.75–2.64 (m, 1H), 2.59–2.48 (m, 1H), 2.41–2.26 (m, 2H). ^{13}C NMR (100 MHz, $CDCl_3$) δ 202.8 and 202.5, 158.3 and 158.2, 137.1 and 136.7, 127.5 and 127.4, 114.0 and 113.9, 55.4, 42.3 and 41.5, 36.3 and 35.5, 30.4 and 29.5. HRMS (ESI+) calcd for $C_{12}H_{15}O_2$ $[M+H]^+$ 191.1067, found 191.1071.

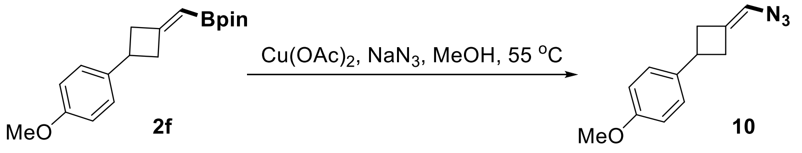

Compound 10:

To a 5-mL vial were added **2f** (60 mg, 0.20 mmol), NaN_3 (20 mg, 0.30 mmol), $Cu(OAc)_2$ (3.6 mg, 0.020 mmol), and MeOH (1.0 mL), and the resulting reaction mixture was stirred at 55 °C under air for 3 h. After the reaction mixture was cooled to room temperature, the volatiles were evaporated

under reduced pressure, and the residue was purified by column chromatography on silica gel (hexane:EA 100:1) to give the product **10** (31.8 mg, 74%) as a yellow oil. ^{1}H NMR (400 MHz, $CDCl_3$) δ 7.18 (d, J = 8.4 Hz, 2H), 6.86 (d, J = 8.8 Hz, 2H), 5.85 (p, J = 2.4 Hz, 1H), 3.80 (s, 3H), 3.54 (p, J = 8.4 Hz, 1H), 3.20–3.01 (m, 2H), 2.83–2.74 (m, 2H). ^{13}C NMR (100 MHz, $CDCl_3$) δ 158.2, 137.3, 128.5, 127.5, 117.6, 114.0, 55.4, 37.1, 36.4, 35.2. HRMS (ESI-) calcd for $C_{12}H_{14}NO$ $[M-N_2+H]^-$ 188.1081, found 188.1070.

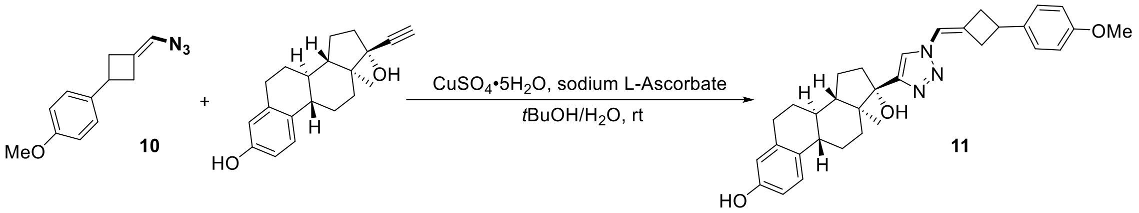

Compound 11:

To a solution of the compound **10** (33 mg, 0.15 mmol) and 17a-Ethynyl estradiol (30 mg, 0.10 mmol) in ^{t}BuOH/H_2O (1:1) (1.0 mL) were added $CuSO_4$•$5H_2O$ (5 mg, 0.02 mmol) and sodium ascorbate (4 mg, 0.02 mmol). The resulting reaction mixture was stirred at room temperature for 20 h The volatiles were evaporated under reduced pressure, and the residue was purified by column chromatography on silica gel (hexane:EA 3:1) to give the product **11** (39.8 mg, 78%) as a white solid. ^{1}H NMR (400 MHz, $CDCl_3$) δ 7.49 (s, 1H), 7.22 (d, J = 8.8 Hz, 2H), 7.09–7.00 (m, 2H), 6.88 (d, J = 8.4 Hz, 2H), 6.59 (dd, J = 8.4, 2.5 Hz, 1H), 6.56–6.54 (m, 1H), 3.81 (s, 3H), 3.75–3.64 (m, 1H), 3.51–3.40 (m, 1H), 3.35–3.26 (m, 1H), 3.23–3.12 (m, 1H), 3.09–2.98 (m, 1H), 2.85–2.73 (m, 2H), 2.50–2.40 (m, 1H), 2.18–2.07 (m, 2H), 1.99–1.83 (m, 3H), 1.69–1.55 (m, 2H), 1.50–1.36 (m, 2H), 1.36–1.23 (m, 2H), 1.04 (s, 3H), 0.79–0.66 (m, 1H). ^{13}C NMR (100 MHz, $CDCl_3$) δ 153.6, 153.5, 138.4, 136.6, 132.90, 132.87, 132.7, 127.5, 126.5, 119.8, 116.9, 115.4, 114.1, 112.8, 82.6, 55.5, 48.6, 47.5, 43.4, 39.6, 39.2, 38.2, 37.5, 35.5, 33.1, 29.8, 27.4, 26.4, 23.6, 14.4. HRMS (ESI+) calcd for $C_{32}H_{38}N_3O_3$ $[M+H]^+$ 512.2908, found 512.2903.

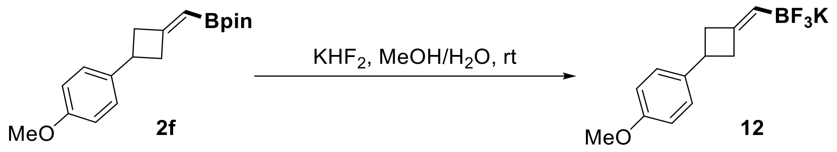

Compound 12:

To a solution of the compound **2f** (60 mg, 0.20 mmol) in MeOH (1.0 mL) was added a solution of KHF_2 (0.44 mL, 4.5 M in H_2O). The resulting reaction mixture was stirred at room temperature for 24 h, and the volatiles were evaporated under reduced pressure. To the residue was added hot acetone (3 mL), and the resulting suspension was filtered through a sintered disc filter. The combined filtrate was concentrated under reduced pressure, and the residue was washed with pentane and Et_2O (2:1, 5 mL). All volatiles were removed *in vacuo*, giving the corresponding potassium trifluoroborate **12** (39.8 mg, 71%) as a white solid. ^{1}H NMR (400 MHz, DMSO-d$_6$) δ 6.15 (d, J = 8.4 Hz, 2H), 5.83 (d, J = 8.4 Hz, 2H), 3.91 (s, 1H), 2.70 (s, 3H), 2.28 (p, J = 8.0 Hz, 1H), 2.06–1.96 (m, 1H), 1.92–1.80 (m, 1H), 1.64–1.32 (m, 2H). ^{13}C NMR (100 MHz, DMSO-d_6) δ 157.9 and 157.6, 140.4 (q, J = 4.4 Hz), 139.2 and 138.0, 127.82 and 127.78, 114.2 and 114.0, 55.5 and 55.4, 42.6 and 42.1, 42.0 and 41.1, 34.5and 34.0. ^{19}F NMR (376 MHz, DMSO-d_6) δ -141.2 (m). ^{11}B NMR (128 MHz, DMSO-d_6) δ 0.6 (q, J = 18.4 Hz).

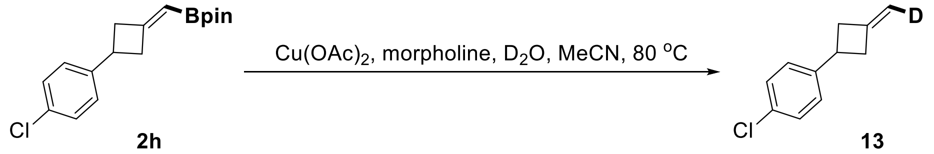

Compound 13:

To a 5-mL screw-up vial were added **2h** (30.4 mg, 0.100 mmol), $Cu(OAc)_2$ (22 mg, 0.11 mmol), morpholine (11 μL, 0.10 mmol), acetonitrile (0.5 mL) and D_2O (30 μL). The mixture was allowed to stir at 80 °C for 16 h under Ar. After the reaction mixture was cooled to room temperature, the volatiles were evaporated under reduced pressure, and the residue was purified by column chromatography on silica gel (hexane:EA 100:1) to give the product **13** (9.8 mg, 55%) as a colorless oil. ^{1}H NMR (400 MHz, $CDCl_3$) δ 7.30 (d, J = 8.4 Hz, 2H), 7.22 (d, J = 8.4 Hz, 2H), 5.00–4.70 (m, 1H), 3.51 (q, J = 8.4 Hz, 1H), 3.23–3.03 (m, 2H), 2.90–2.73 (m, 2H). ^{13}C NMR (100 MHz, $CDCl_3$)

δ 145.5, 144.3, 131.8, 128.6, 128.0, 106.2, 39.83, 39.79, 34.5. HRMS (ESI+) calcd for $C_{11}H_{11}DCl$ $[M+H]^+$ 180.0685, found 180.0685.

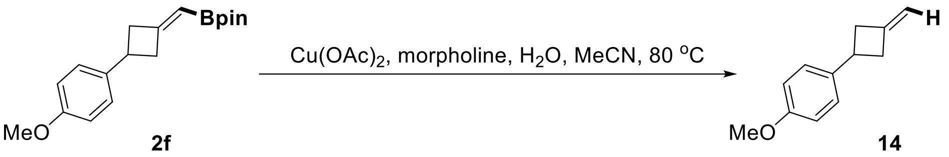

Compound 14:

To a 5-mL screw-up vial were added **2f** (60 mg, 0.20 mmol), $Cu(OAc)_2$ (44 mg, 0.22 mmol), morpholine (22 μL, 0.20 mmol), acetonitrile (1.0 mL) and H_2O (30 μL). The mixture was allowed to stir at 80 °C for 16 h under Ar. After the reaction mixture was cooled to room temperature, the volatiles were evaporated under reduced pressure, and the residue was purified by column chromatography on silica gel (hexane:EA 100:1) to give the product **14** (25.7 mg, 74%) as a colorless oil. ^1H NMR (400 MHz, $CDCl_3$) δ 7.20 (d, J = 8.4 Hz, 2H), 6.86 (d, J = 8.4 Hz, 2H), 4.83 (s, 2H), 3.80 (s, 3H), 3.48 (p, J = 8.4 Hz, 1H), 3.15–3.02 (m, 2H), 2.86–2.77 (m, 2H). ^{13}C NMR (100 MHz, $CDCl_3$) δ 158.1, 146.3, 138.0, 127.5, 113.9, 105.7, 55.4, 40.1, 34.4. HRMS (ESI+) calcd for $C_{12}H_{15}O$ $[M+H]^+$ 175.1117, found 175.1118.

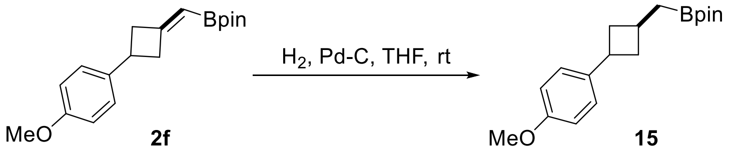

Compound 15:

To a solution of the compound **2f** (60 mg, 0.20 mmol) in THF (1.0 mL) was added 10% Pd-C (3 mg). The flask was evacuated and backfilled with H_2 from a balloon for three times, and the resulting reaction mixture was stirred overnight at room temperature. The mixture was filtered, and the filtrate was evaporated under reduced pressure. The residue was purified by column chromatography on silica gel (hexane:EA 50:1) to give the product **15** (51.3 mg, 85%, d.r. = 7:3) as a mixture of two inseparable diastereoisomers. ^1H NMR (400 MHz, $CDCl_3$) δ 7.16 (d, 8.4 Hz, 0.6H), 7.13 (d, 8.4 Hz, 1.4H), 6.88–6.80 (m, 2H), 3.79 (s, 0.9H), 3.78 (s, 2.1H), 3.62–3.51 (m, 0.3H), 3.29–3.17 (m, 0.7H),

2.58–2.46 (m, 1.7H), 2.45–2.35 (m, 0.7H), 2.34–2.25 (m, 0.6H), 2.11–1.99 (m, 0.6H), 1.76–1.64 (m, 1.4H), 1.243 (s, 3.6H), 1.240 (s, 8.4H), 1.14 (d, *J* = 8.0 Hz, 0.6H), 0.97 (d, *J* = 7.6 Hz, 1.4H). ^{13}C NMR (100 MHz, $CDCl_3$) δ 157.7, 139.0 and 138.5, 127.5, 113.74 and 113.66, 83.0, 55.4, 38.9, 36.9, 36.0 and 35.4, 28.1 and 27.5, 24.9 (the carbon bound to boron was not observed). ^{11}B NMR (128 MHz, $CDCl_3$) δ 34.0. HRMS (ESI+) calcd for $C_{18}H_{28}BO_3$ $[M+H]^+$ 303.2126, found 303.2122.

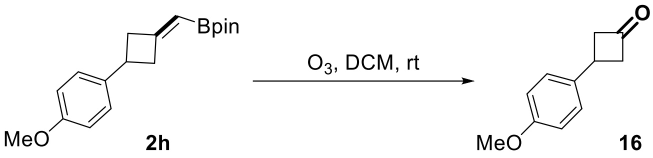

Compound 16:

To a 5-mL screw-up vial were added **2f** (30 mg, 0.10 mmol) and DCM (1.0 mL). The flask was evacuated and backfilled with O_3 from a balloon for three times, and the resulting reaction mixture was stirred 0.5 h at -78 °C. After the reaction was quenched with PPh_3, the volatiles were evaporated under reduced pressure. The residue was purified by column chromatography on silica gel (hexane:EA 30:1) to give the product **16** (13.0 mg, 74%). ^{1}H NMR (400 MHz, $CDCl_3$) δ 7.15 (d, *J* = 8.4 Hz, 2H), 6.82 (d, *J* = 8.8 Hz, 2H), 3.74 (s, 3H), 3.57 (p, *J* = 7.6 Hz, 1H), 3.46–3.34 (m, 2H), 3.19–3.07 (m, 2H). ^{13}C NMR (100 MHz, $CDCl_3$) δ 207.2, 158.5, 135.8, 127.7, 114.2, 55.5, 55.0, 27.9. HRMS (ESI+) calcd for $C_{11}H_{13}O_2$ $[M+H]^+$ 177.0910, found 177.0911.

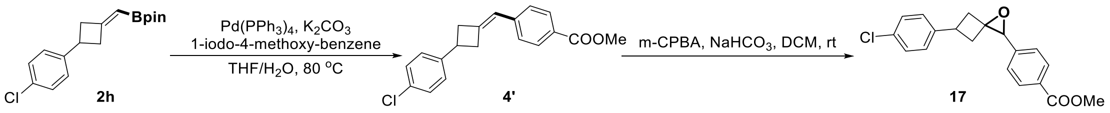

Compound 17:

To a 5-mL screw-up vial were added **2h** (25 mg, 0.080 mmol), 1-iodo-4-methoxy-benzene (62.9 mg, 0.240 mmol), K_2CO_3 (33 mg, 0.24 mmol), $Pd(PPh_3)_4$ (9.2 mg, 8.0 μmol), and THF (0.9 mL) under Ar, followed by the addition of H_2O (0.1 mL) through a syringe. The resulting reaction mixture was stirred at 80 °C for 20 h under Ar. After the reaction mixture was cooled to room temperature, the volatiles were evaporated, and the residue was purified by column chromatography on silica (hexane:EA 50:1) to give the product **4′** (20.0 mg, 76%). To a 5-mL screw-up vial were added **4′** (20 mg, 0.060 mmol), 3-chloroperoxybenzoic acid (20.7 mg, 0.120 mmol), $NaHCO_3$(15.1

mg, 0.180 mmol), and DCM (1.0 mL) under Ar. The resulting mixture was stirred at room temperature for 12 h. The volatiles were evaporated under reduced pressure, and the residue was purified by column chromatography on silica gel (hexane:EA 20:1) to give the product **17** (17.7 mg, 90%, 6:4 dr) as a mixture of two inseparable diastereoisomers. ^{1}H NMR (400 MHz, $CDCl_3$) δ 8.13–7.91 (m, 2H), 7.33–7.24 (m, 3H), 7.26–7.15 (m, 2H), 7.05 (d, J = 8.4 Hz, 1H), 4.03 (s, 0.4H), 3.96 (s, 0.6H), 3.93 (s, 1.2H), 3.92 (s, 1.8H), 3.68–3.55 (m, 0.6H), 3.24 (p, J = 8.8 Hz, 0.4H), 3.13–3.03 (m, 0.6H), 2.98–2.84 (m, 1H), 2.80–2.69 (m, 0.4H), 2.63–2.50 (m, 1H), 2.43–2.29 (m, 0.4H), 2.08 (dt, J = 13.6, 3.2 Hz, 0.6H). ^{13}C NMR (100 MHz, $CDCl_3$) δ 166.94 and 166.91, 143.5 and 142.8, 141.7 and 141.4, 132.2 and 132.0, 129.93 and 129.91, 129.8 and 129.7, 128.73 and 128.72, 128.0 and 127.9, 126.2 and 126.1, 65.8 and 64.1, 61.9 and 61.8, 52.3, 39.1 and 38.9, 36.2 and 35.4, 31.2 and 30.6. HRMS (ESI+) calcd for $C_{19}H_{18}ClO_3$ $[M+H]^+$ 329.0939, found 329.0937.

6. Concise total synthesis of cyclobutane-containing natural products

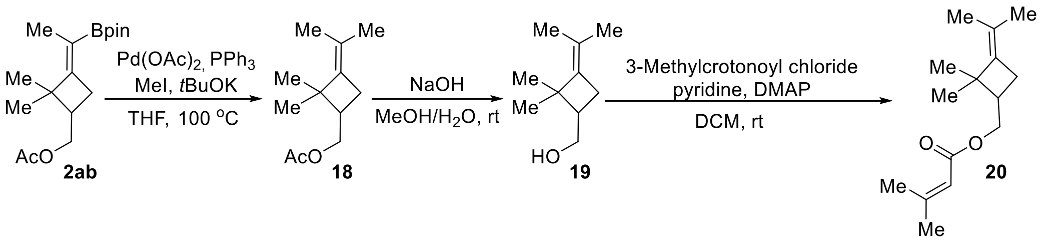

To a solution of **2ab** (0.13 g, 0.40 mmol), $Pd(OAc)_2$ (9.0 mg, 0.040 mmol), potassium tert-butoxide (54 mg, 0.48 mmol) and PPh_3 (21 mg, 0.080 mmol) in THF (2 mL) was added MeI (0.28 g, 2.0 mmol) at room temperature under Ar. The resulting reaction mixture was stirred at 100 °C for 20 h. After the reaction was completed, the volatiles were evaporated under reduced pressure, and the residue was purified by column chromatography on silica gel (hexane:EA 100:1) to give product **18** (47 mg, 60%). ^{1}H NMR (400 MHz, $CDCl_3$) δ 4.23–3.92 (m, 2H), 2.60 (dd, J = 13.2, 7.6 Hz, 1H), 2.25–2.08 (m, 2H), 2.04 (s, 3H), 1.58 (s, 3H), 1.45 (s, 3H), 1.25 (s, 3H), 1.14 (s, 3H).

Compound **18** (47 mg, 0.24 mmol) was dissolved in MeOH/H_2O (1:1) (2.0 mL), to which NaOH (48 mg, 1.2 mmol) was added. The reaction was stirred for 3 h at room temperature and quenched

with saturated aqueous NH_4Cl solution (10 mL). The volatiles were evaporated under reduced pressure, and the residue was extracted with ethyl acetate (5 mL * 3). The combined organic layers were washed with brine, dried over Na_2SO_4, filtered, and concentrated *in vacuo*. The residue was purified by flash column chromatography on silica gel to give the alcohol **19**.

To a solution of the alcohol **19** (37 mg, 0.24 mmol), pyridine (29 mg, 0.36 mmol), and DMAP (6 mg, 0.05 mmol) in DCM (2 mL) was added 3-methylcrotonoyl chloride (43 mg, 0.36 mmol) at room temperature. After the reaction was completed, the volatiles were evaporated under reduced pressure, and the residue was purified by column chromatography on silica gel (hexane:EA 100:1) to give product **20** (32 mg, 68%). ^{1}H NMR (400 MHz, $CDCl_3$) δ 5.66 (s, 1H), 4.29–3.96 (m, 2H), 2.65–2.53 (m, 1H), 2.26–2.10 (m, 5H), 1.88 (s, 3H), 1.57 (s, 3H), 1.45 (s, 3H), 1.25 (s, 3H), 1.14 (s, 3H). ^{13}C NMR (100 MHz, $CDCl_3$) δ 167.0, 156.4, 137.4, 122.7, 116.3, 64.9, 44.4, 39.3, 28.6, 28.0, 27.5, 21.2, 20.3, 19.7, 18.7. HRMS (ESI+) calcd for $C_{15}H_{25}O_2$ $[M+H]^+$ 237.1849, found 237.1854.

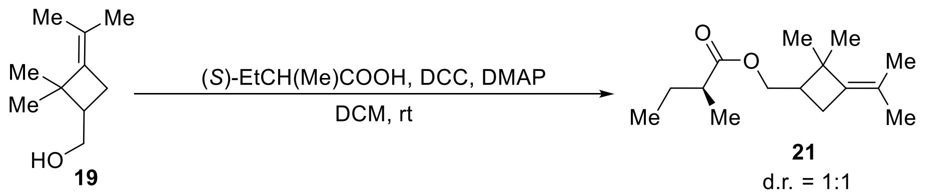

To a solution of the alcohol **19** (30 mg, 0.20 mmol), (*S*)-(+)-2-Methylbutyric acid (31 mg, 0.30 mmol), and DMAP (5 mg, 0.04 mmol) in DCM (2 mL) was added DCC (61 mg, 0.30 mmol) at room temperature. After the reaction was completed, the volatiles were evaporated under reduced pressure, and the residue was purified by column chromatography on silica gel (hexane:EA 100:1) to give product **21** (15 mg, 65%) as a mixture of two inseparable diastereoisomers (d.r. = 1:1). ^{1}H NMR (400 MHz, C_6D_6) δ 4.26–4.05 (m, 2H), 2.55–2.43 (m, 1H), 2.33–2.23 (m, 1H), 2.22–2.12 (m, 1H), 2.11–2.01 (m, 1H), 1.76–1.62 (m, 1H), 1.52 (s, 3H), 1.41 (s, 3H), 1.39–1.32 (m, 1H), 1.24 (s, 3H), 1.13 (s, 3H), 1.09 (d, J = 7.2 Hz, 3H), 0.84 (t, J = 7.6 Hz, 3H). ^{13}C NMR (100 MHz, C_6D_6) δ 175.8, 137.5, 122.6, 65.3, 44.4, 41.4, 39.8 and 39.7, 28.7, 27.8, 27.2 and 27.1, 21.2, 19.6, 18.7, 16.93 and 19.89, 11.84.

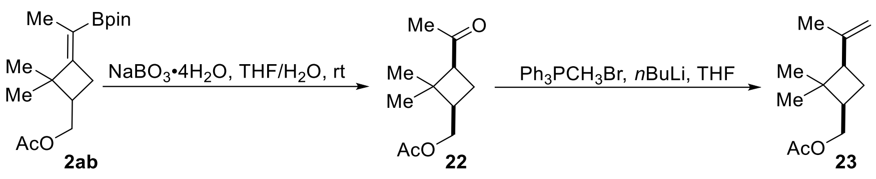

To a solution of sodium perborate tetrahydrate (289 mg, 1.88 mmol) in THF/H_2O (2:1) (1.5 mL) was added **2ab** (0.12 g, 0.38 mmol). The resulting reaction mixture was stirred for 1.5 h at room temperature. After the reaction was completed, the volatiles were evaporated under reduced pressure, and the residue was purified by column chromatography on silica gel (hexane:EA 5:1) to give product **22** (75.0 mg, 99%) as a mixture of two inseparable diastereoisomers (d.r. = 2:1). ^{1}H NMR (400 MHz, $CDCl_3$) δ 4.19–4.11 (m, 0.33H), 4.10–3.98 (m, 1H), 3.96–3.87 (m, 0.67H), 2.96 (t, J = 7.6 Hz, 0.33H), 2.85 (dd, J = 10.0, 7.6 Hz, 0.67H), 2.48–2.37 (m, 0.33H), 2.31-2.19 (m, 0.67H), 2.19–2.09 (m, 0.33H), 2.07–1.91 (m, 6.67H), 1.85–1.74 (m, 0.67H), 1.57–1.47 (m, 0.33H), 1.32 (s, 2H), 1.22 (s, 1H), 1.02 (s, 1H), 0.89 (s, 2H). ^{13}C NMR (100 MHz, $CDCl_3$) δ 208.4 and 207.5, 171.2 and171.0, 65.1 and 64.5, 53.8 and 53.6, 42.7 and 41.2, 40.0 and 39.7, 30.9 and 30.7, 30.3, 21.1 and 21.0, 19.8 and 19.6, 17.2. HRMS (ESI+) calcd for $C_{11}H_{18}NaO_3$ $[M+Na]^+$ 221.1148, found 221.1151.

To a solution of methyltriphenylphosphonium bromide (0.20 g, 0.57 mmol) in THF (2 mL) was added n-BuLi (1.6 M in hexane, 0.36 mL) dropwise at 0 °C under Ar. The mixture was stirred for 0.5 h at this temperature and then cooled to -78 °C. A solution of ketone **22** (75 mg, 0.38 mmol) in THF was added dropwise. After the reaction was completed, the volatiles were evaporated under reduced pressure, and the residue was purified by column chromatography on silica gel (hexane:EA 150:1) to give product **23** (44.7 mg, 60%) as a mixture of two inseparable diastereoisomers (d.r. = 2:1). ^{1}H NMR (400 MHz, $CDCl_3$) δ 4.84 (s, 0.33H), 4.80 (s, 0.67H), 4.62 (s, 0.33H), 4.56 (s, 0.67H), 4.30–4.21 (m, 0.33H), 4.19–4.09 (m, 0.33H), 4.08–4.00 (m, 0.67H), 3.99–3.86 (m, 0.67H), 2.58 (t, J = 8.8 Hz, 0.33H), 2.39 (t, J = 9.2 Hz, 0.67H), 2.23–2.10 (m, 1H), 2.07–1.94 (m, 3.33H), 1.93–1.84 (m, 0.67H), 1.68–1.54 (m, 4.33H), 1.25 (s, 0.66H), 1.19 (s, 2H), 1.10 (s, 1H), 0.96 (s, 1H), 0.81 (s, 2H). ^{13}C NMR (100 MHz, $CDCl_3$) δ 171.5 and 171.3, 145.6 and 145.1, 109.6 and 109.5, 65.8 and 65.1, 49.0 and 48.3, 41.3 and 40.0, 39.8 and 39.5, 31.1, 23.3 and 23.2, 23.1 and 22.4, 21.2 and 21.1, 16.3. HRMS (ESI+) calcd for $C_{12}H_{21}O_2$ $[M+H]^+$ 197.1536, found 197.1541.

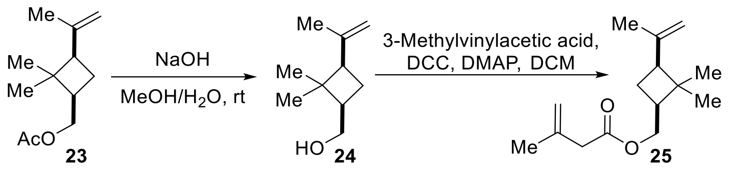

To a solution of **23** (20 mg, 0.10 mmol) in MeOH/H_2O (1:1) (2.0 mL) was added NaOH (20 mg, 0.50 mmol). The resulting reaction mixture was stirred for 1 h at room temperature. The reaction was quenched with saturated aqueous NH_4Cl solution (10 mL), and the resulting mixture was evaporated under reduced pressure. The residue was extracted with ethyl acetate (5 mL * 3), and the combined organic layers were washed with brine, dried over Na_2SO_4, filtered, and concentrated *in vacuo* to give alcohol **24**, which was used directly in the next step without further purification. To a solution of alcohol **24** (15 mg, 0.10 mmol), 3-butenoic acid (15 mg, 0.15 mmol), and DMAP (3 mg, 0.02 mmol) in DCM (2 mL) was added DCC (31 mg, 0.15 mmol) at room temperature. After the reaction was completed, the volatiles were evaporated under reduced pressure, and the residue was purified by column chromatography on silica gel (hexane:EA 100:1) to give product **25** (15 mg, 65%) as a mixture of two diastereoisomers (d.r. = 2:1). ^{1}H NMR (400 MHz, $CDCl_3$) δ 4.90 (s, 1H), 4.85–4.80 (m, 2H), 4.63 (s, 0.3H), 4.56 (s, 0.7H), 4.27 (dd, J = 11.2, 8.0 Hz, 0.3H), 4.18 (dd, J = 11.2, 7.2 Hz, 0.3H), 4.07 (dd, J = 11.2, 6.4 Hz, 0.7H), 3.96 (dd, J = 11.2, 8.8 Hz, 0.7H), 3.03 (s, 0.6H), 3.01 (s, 1.4H), 2.59 (t, J = 8.8 Hz, 0.3H), 2.44–2.33 (m, 0.7H), 2.24–2.08 (m, 1H), 1.93–1.84 (m, 1H), 1.81 (s, 3H), 1.65 (s, 3H), 1.63–1.58 (m, 1H), 1.19 (s, 2H), 1.10 (s, 1H), 0.96 (s, 1H), 0.81 (s, 2H). ^{13}C NMR (100 MHz, $CDCl_3$) δ 171.5, 145.6 and 145.2, 138.7, 114.8, 109.6 and 109.5, 66.0 and 65.3, 49.0 and 48.3, 43.80 and 43.76, 41.3, 40.0 and 39.6, 31.1, 23.4 and 23.11, 23.06, 22.6 and 22.3, 16.3. HRMS (ESI+) calcd for $C_{15}H_{25}O_2$ $[M+H]^+$ 237.1849, found 237.1852.

7. Mechanism Studies

Ligand effect on the regioselectivity of the borylcupration of the alkyne and reactivity of the vinyl copper intermediate Int-B:

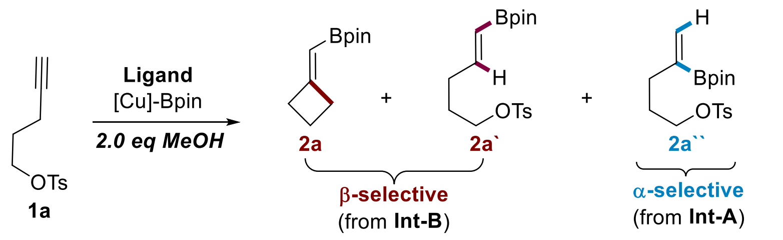

Ligand	Yield 2a/2a`/2a``	Regioselectivity β:α = (2a + 2a`):2a``	Reactivity of Int-B cyclization *vs* protonation 2a:2a`
**IMes**	60%/11%/5%	**14.2 : 1**	**5.5 : 1**
BINAP	55%/15%/7%	10 : 1	4.7 : 1
IPr	14%/38%/10%	5.2 : 1	0.4 : 1
SIPr	42%/29%/16%	4.4 : 1	1.4 : 1

Procedure G: To a 5 mL-vial were added CuCl (5 µmol, 5 mol%), *t*BuOK (0.12 mmol,1.2 equiv), and THF (0.5 mL) in an argon-filled glovebox. The mixture was stirred for 5 minutes, followed by the addition of the ligand (6 µmol, 6 mol%), B_2pin_2 (30.5 mg, 0.120 mmol, 1.20 equiv), **1a** (0.10 mmol), MeOH (2.0 equiv), and THF (0.5 mL). The vial was sealed with a Teflon-lined cap, and the reaction mixture was heated at 50 °C till compound **1a** was fully consumed, which was monitored by TLC. The reaction mixture was cooled to room temperature, and the volatiles were removed under reduced pressure. The yields of **2a**, **2a`**, and **2a``** were determined by ^1H NMR analysis with 1,3,5-trimethoxybenzene used as the internal standard.

Substituent effect on the regioselectivity of the borylcupration:

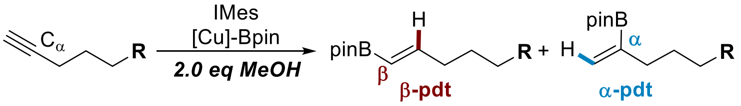

R	Yield β-pdt/α-pdt	Selectivity (β:α)	^{13}C shifts (ppm) δC_α	δC_β	$\Delta(\delta C_\beta - \delta C_\alpha)$
OTs	71%/5%	14.2 : 1^a	82.2	69.5	-12.7
OBz	64%/8%	8.0 : 1	83.1	69.2	-13.9
NPhth	82%/12%	6.8 : 1	83.1	69.1	-14.0
NMeTs	71%/11%	6.5 : 1	83.4	69.1	-14.3
OBn	50%/13%	3.8 : 1	84.1	68.7	-15.4

^{a}β:α selectivity refers to the ratio of **(2a + 2a`):2a``**

Procedure H: To a 5 mL-vial were added CuCl (5 µmol, 5 mol%), *t*BuOK (0.12 mmol,1.2 equiv), and THF (0.5 mL) in an argon-filled glovebox. The mixture was stirred for 5 minutes, followed by the addition of the ligand (6 µmol, 6 mol%), B_2pin_2 (30.5 mg, 0.120 mmol, 1.20 equiv), alkyne substrate (0.10 mmol), MeOH (2.0 equiv), and THF (0.5 mL). The vial was sealed with a Teflon-lined cap, and the reaction mixture was heated at 50 °C till alkyne was fully consumed, which was monitored by TLC. The reaction mixture was cooled to room temperature, and the volatiles were removed under reduced pressure. The yields of the protoborylated products were determined by ^{1}H NMR analysis with 1,3,5-trimethoxybenzene used as the internal standard. The ^{13}C NMR data were obtained from a sample in $CDCl_3$, and the signal of the residue $CHCl_3$ (77.2 ppm) was used as the standard.

8. References

1. James E. M. Lewis, Florian Modicom, and Stephen M. Goldup, *J. Am. Chem. Soc.* **2018**, *140*, 4787-4791.
2. Sumeng Liu, Zhejun Zhang, John R. Abelson, and Gregory S. Girolami, *Organometallics*, **2020**, *39*, 3817-3829.
3. Masahito Ochiai, and Hermann J. Frohn, *J. Am. Chem. Soc.* **2005**, *127,* 10460-10461.
4. Maksym Kovalenko, Dmytro V. Tolmachev, and Oleksandr O. Grygorenko, *Eur. J. Org. Chem*. **2019**, *33*, 5624-5635.
5. Yue Hu, Wei Sun, Tao Zhang, Nuo Xu, Jianeng Xu, Yu Lan, Chao Liu, *Angew. Chem. Int. Ed.* **2019**, *58*, 15813-15818.

9. NMR Spectra

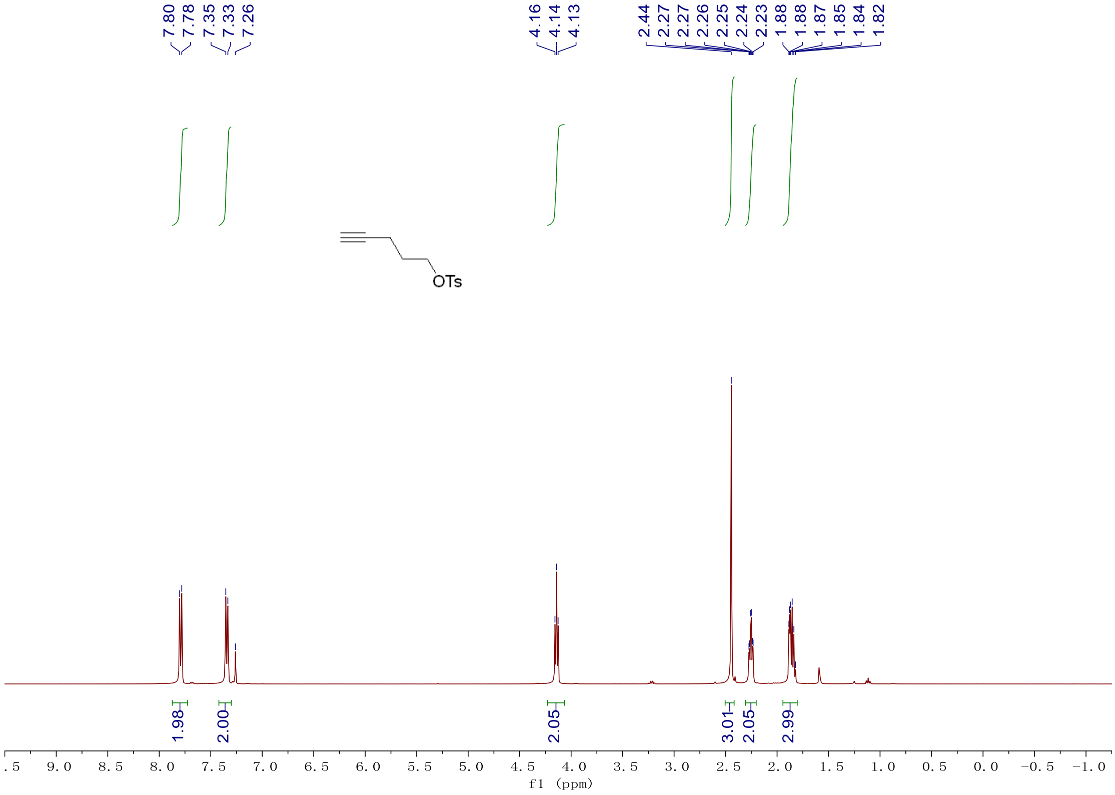

^{1}H NMR of compound 1a

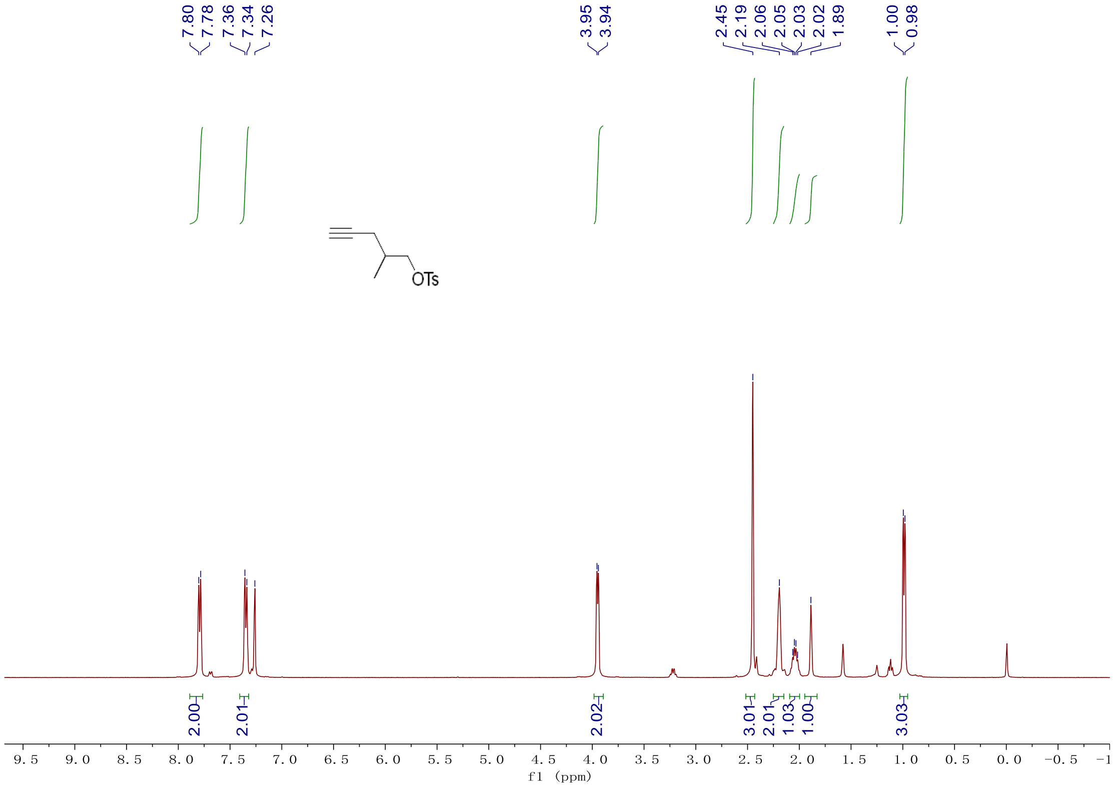

^{1}H NMR of compound 1b

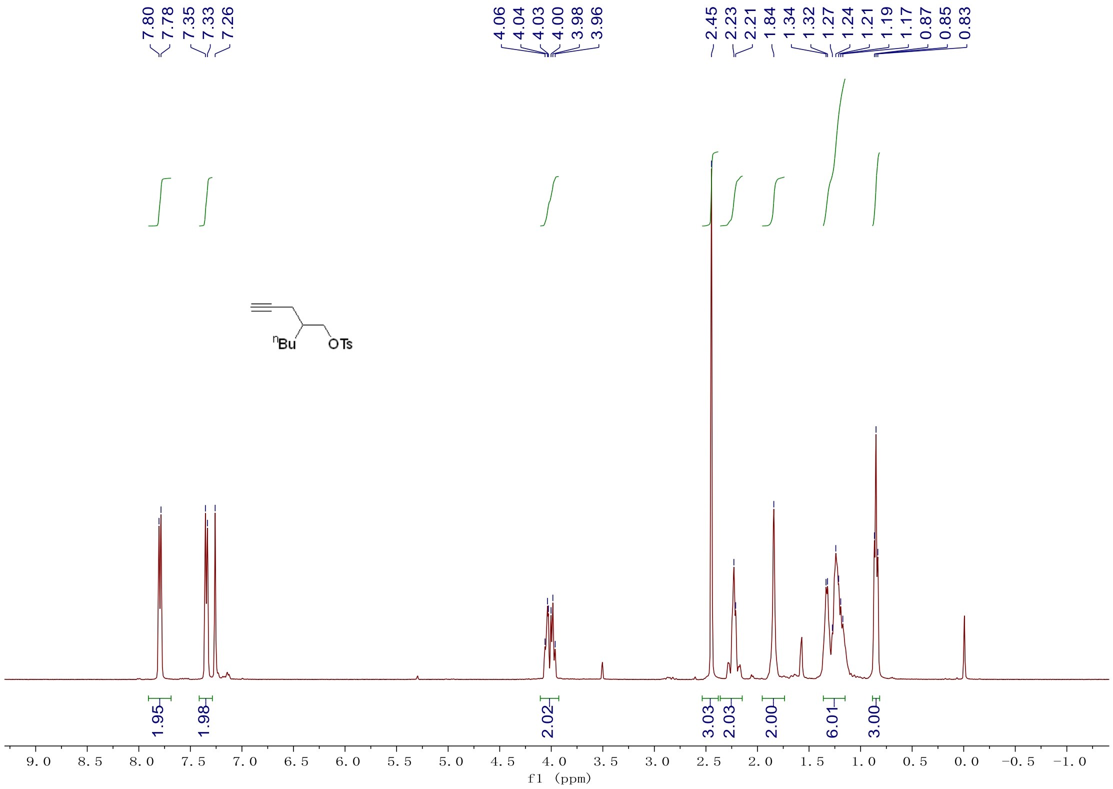

^{1}H NMR of compound 1c

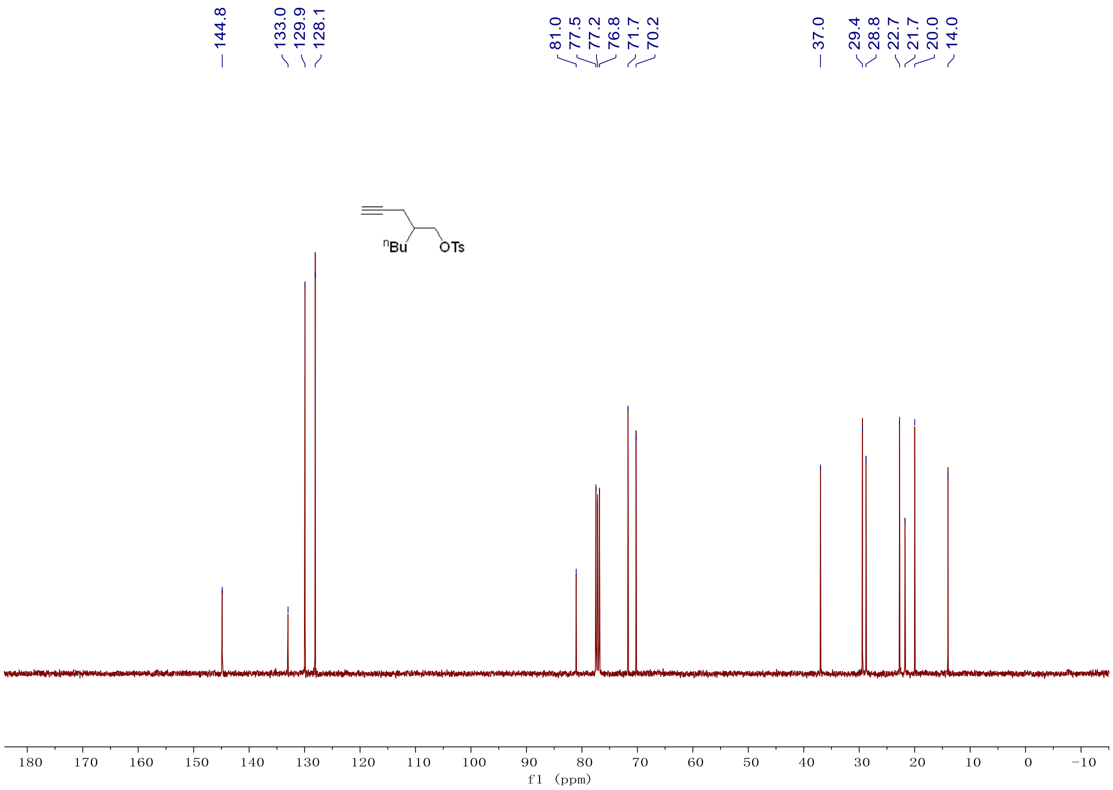

^{13}C NMR of compound 1c

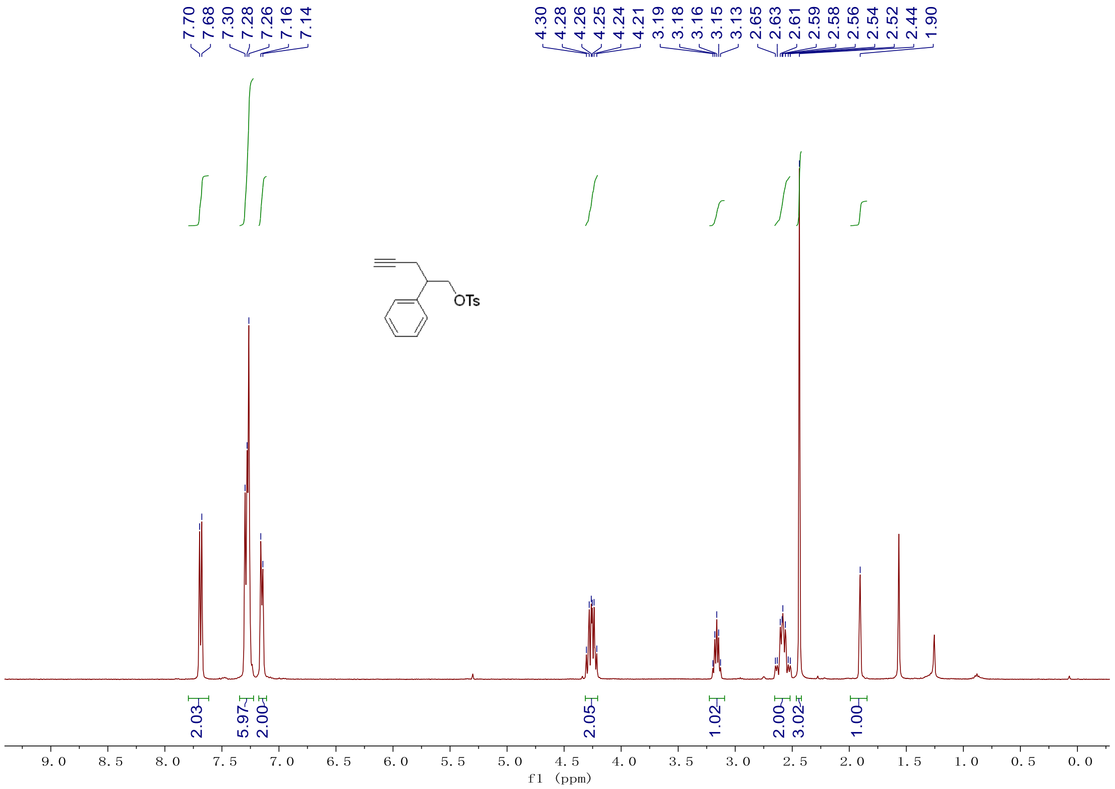

^{1}H NMR of compound 1d

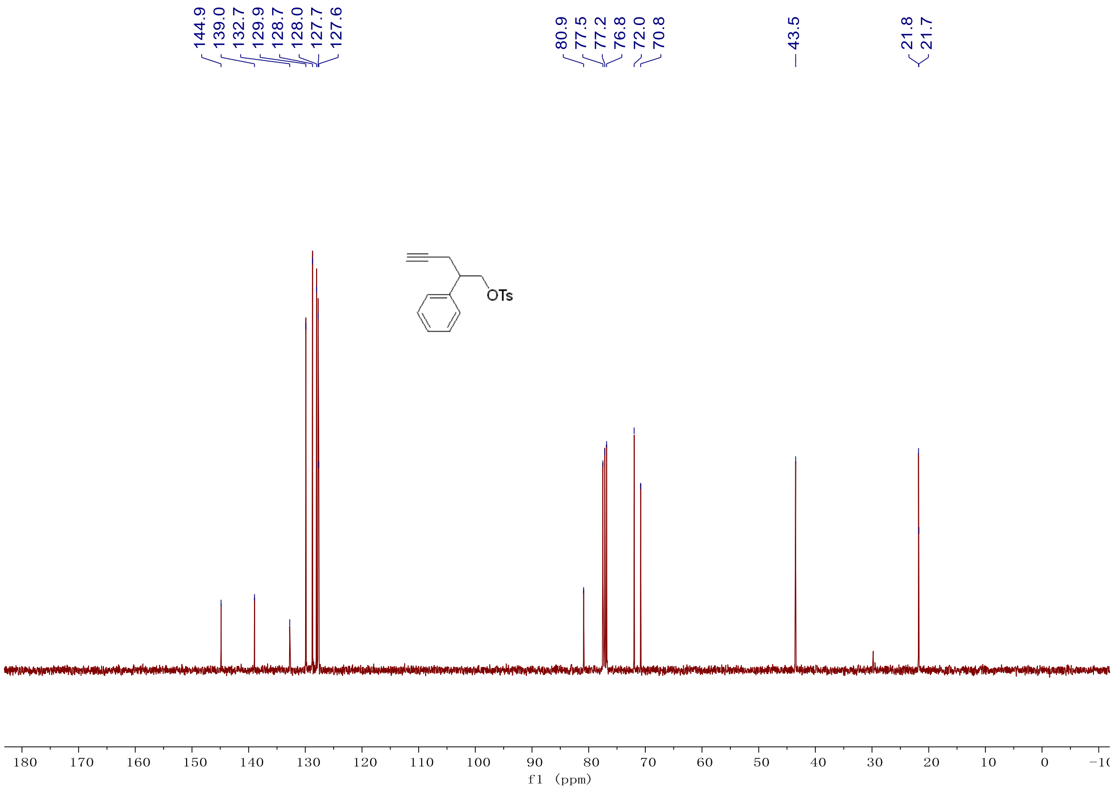

^{13}C NMR of compound 1d

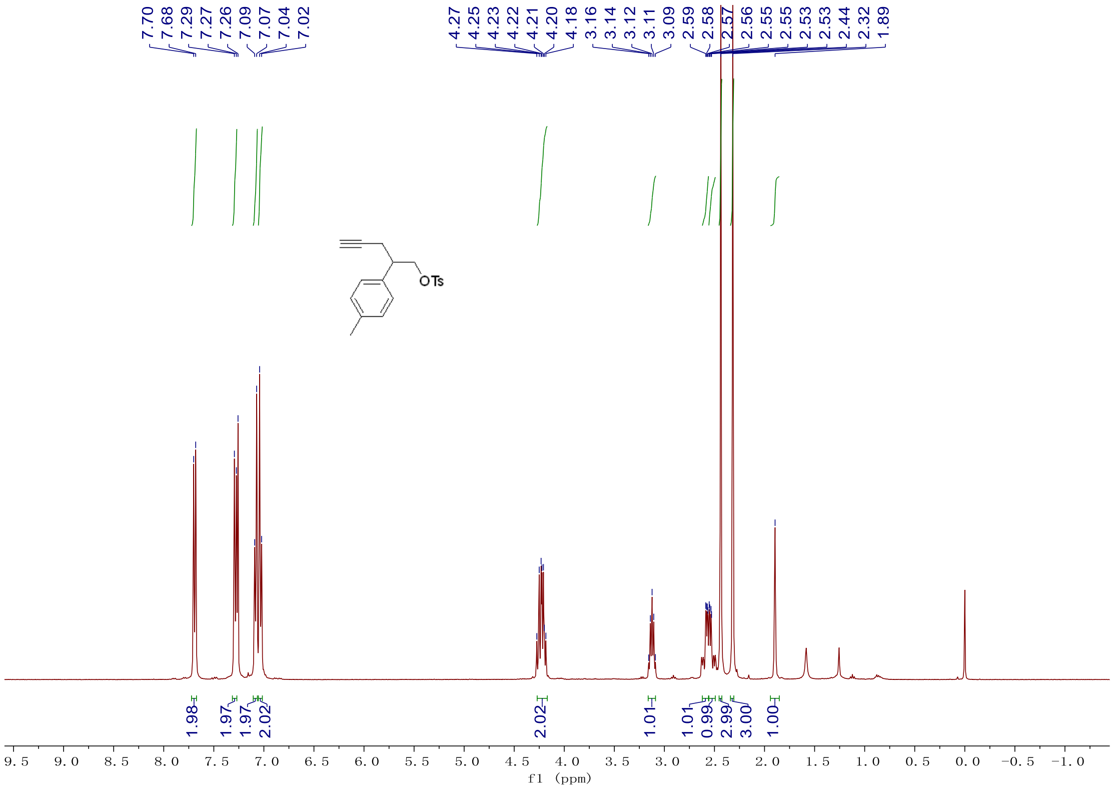

^{1}H NMR of compound 1e

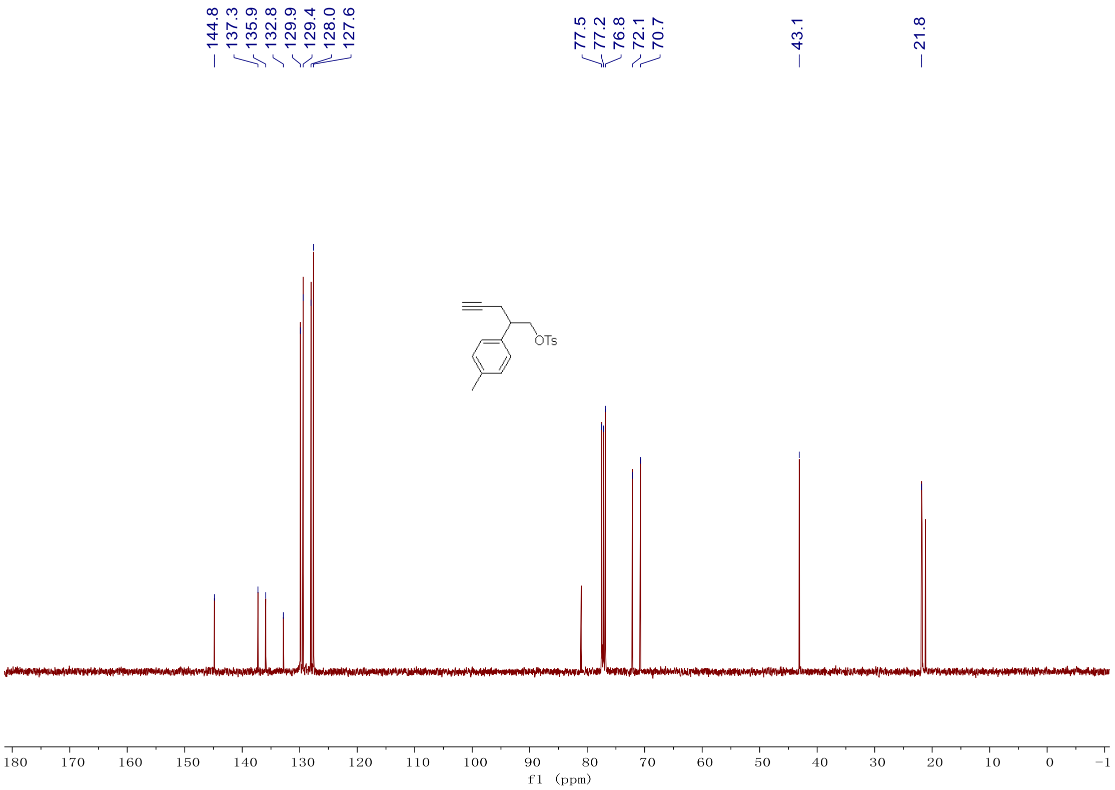

^{13}C NMR of compound 1e

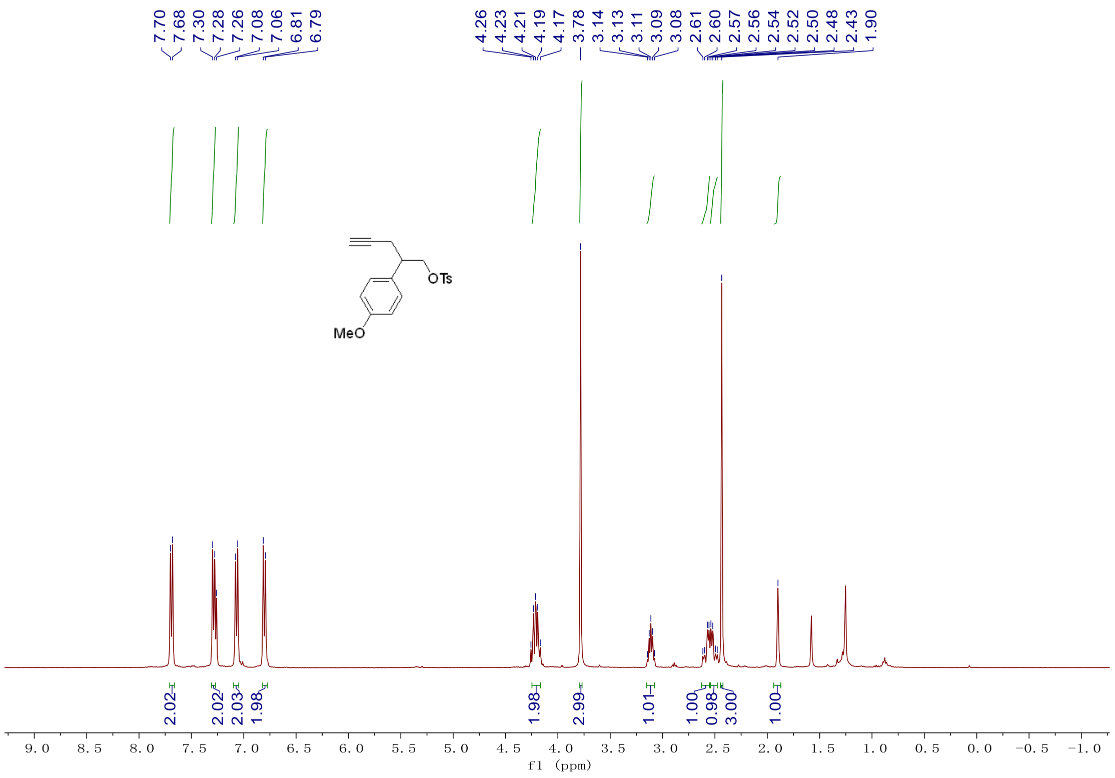

^{1}H NMR of compound 1f

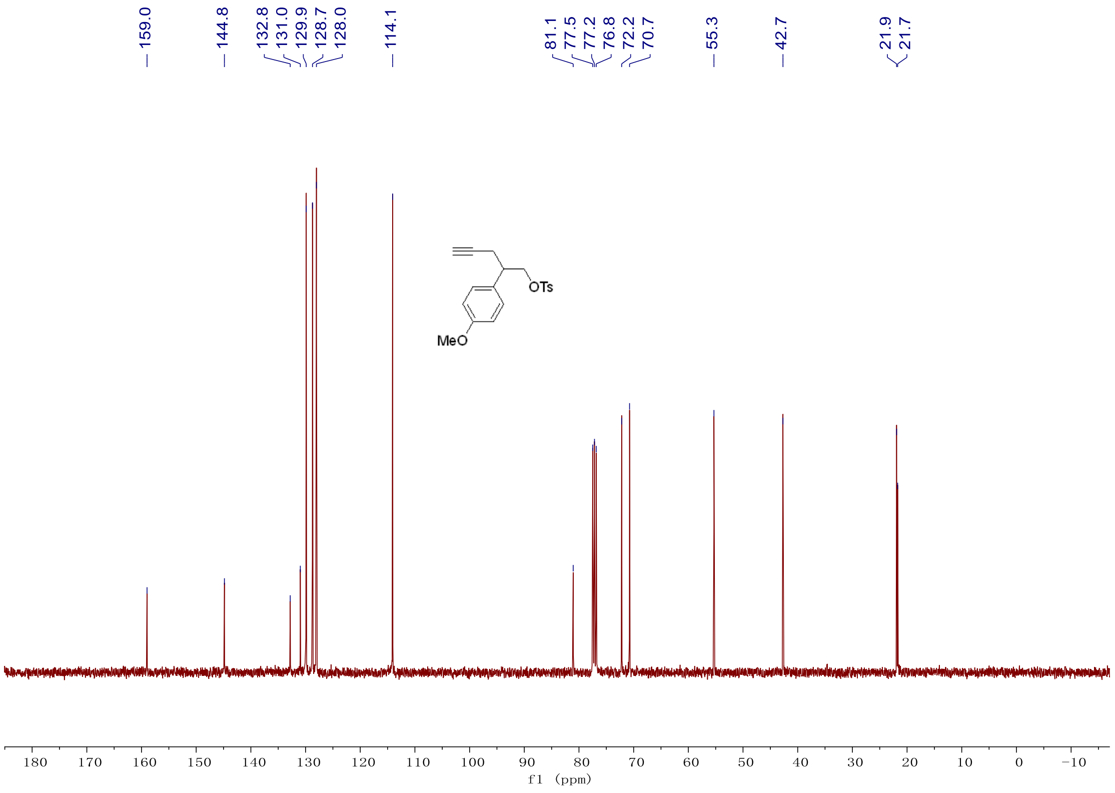

^{13}C NMR of compound 1f

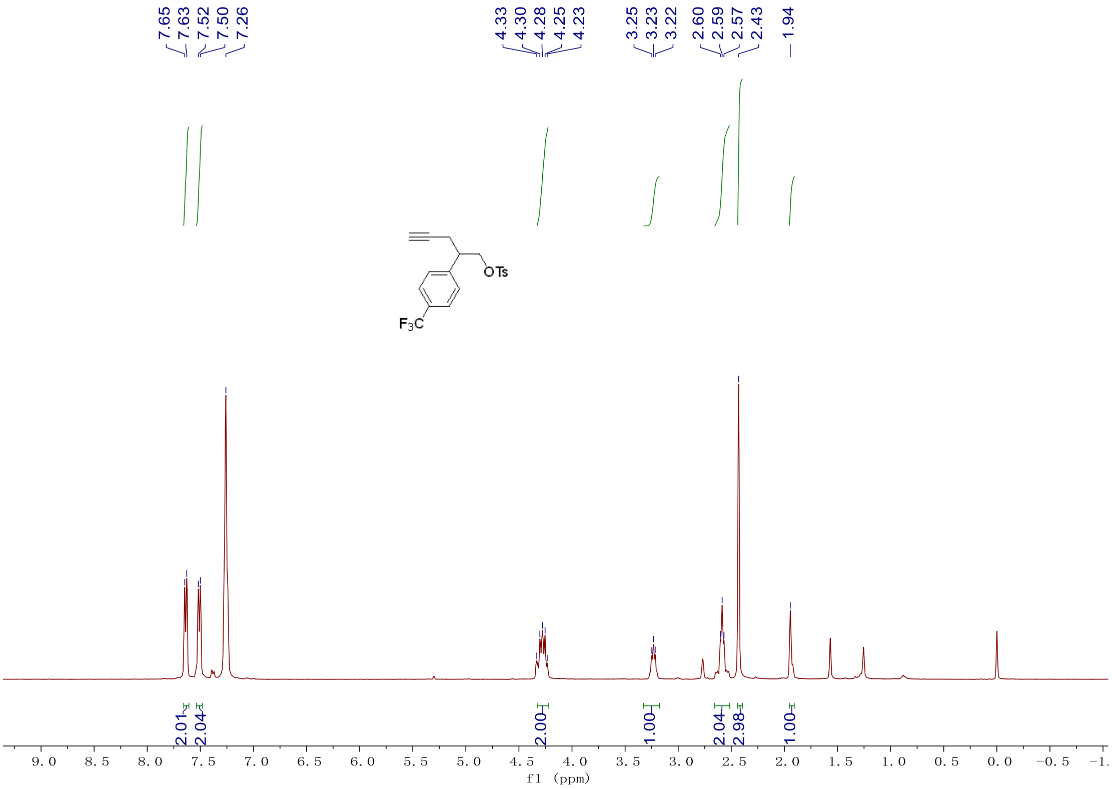

^{1}H NMR of compound 1g

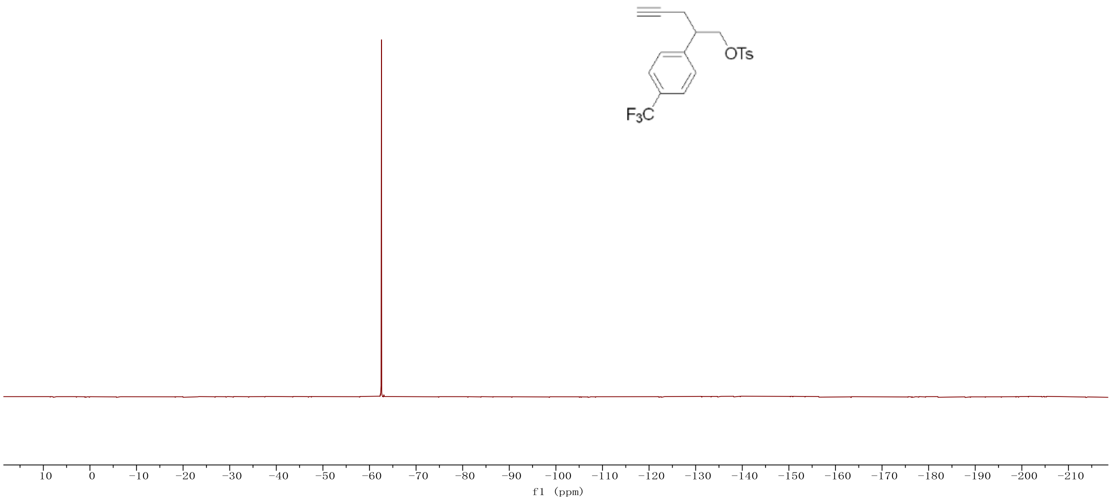

^{19}F NMR of compound 1g

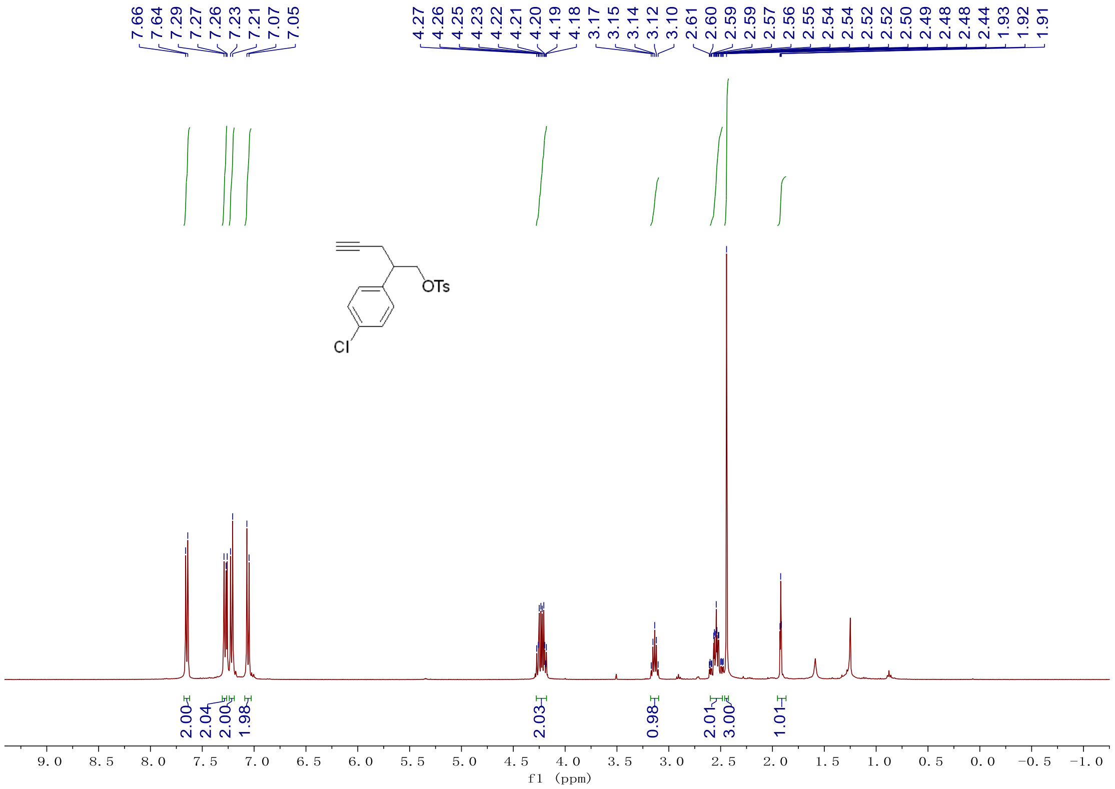

^{1}H NMR of compound 1h

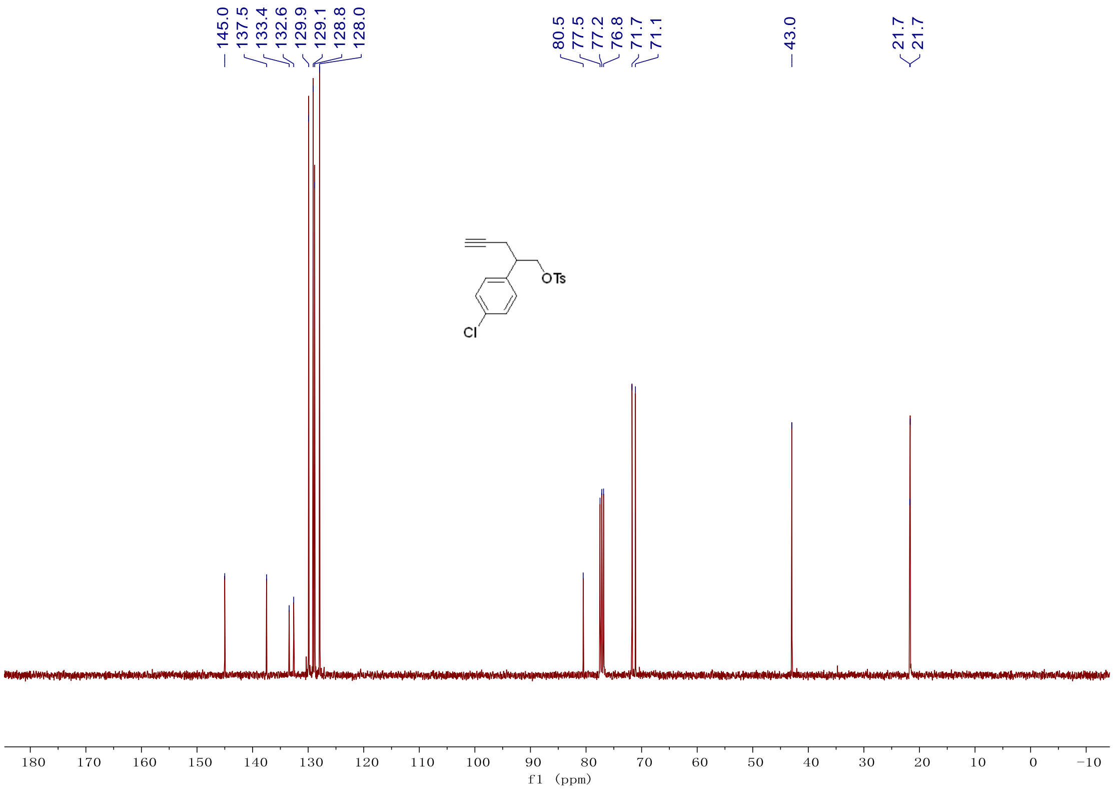

^{13}C NMR of compound 1h

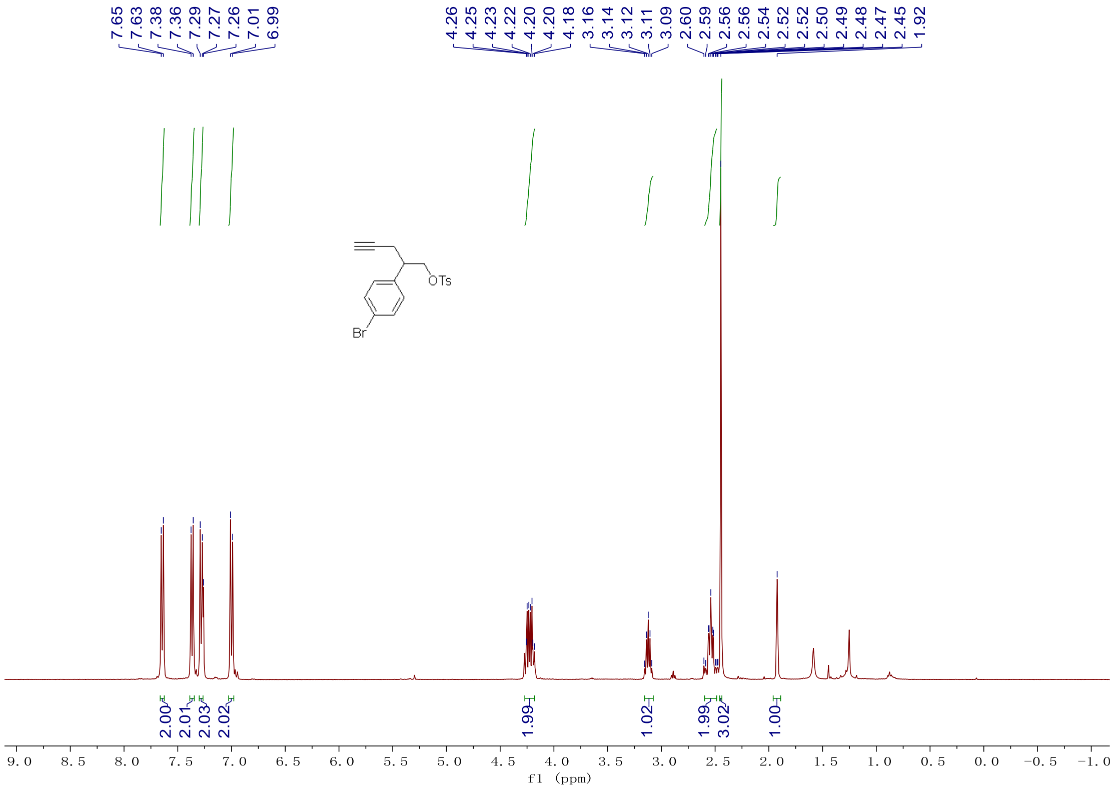

^{1}H NMR of compound 1i

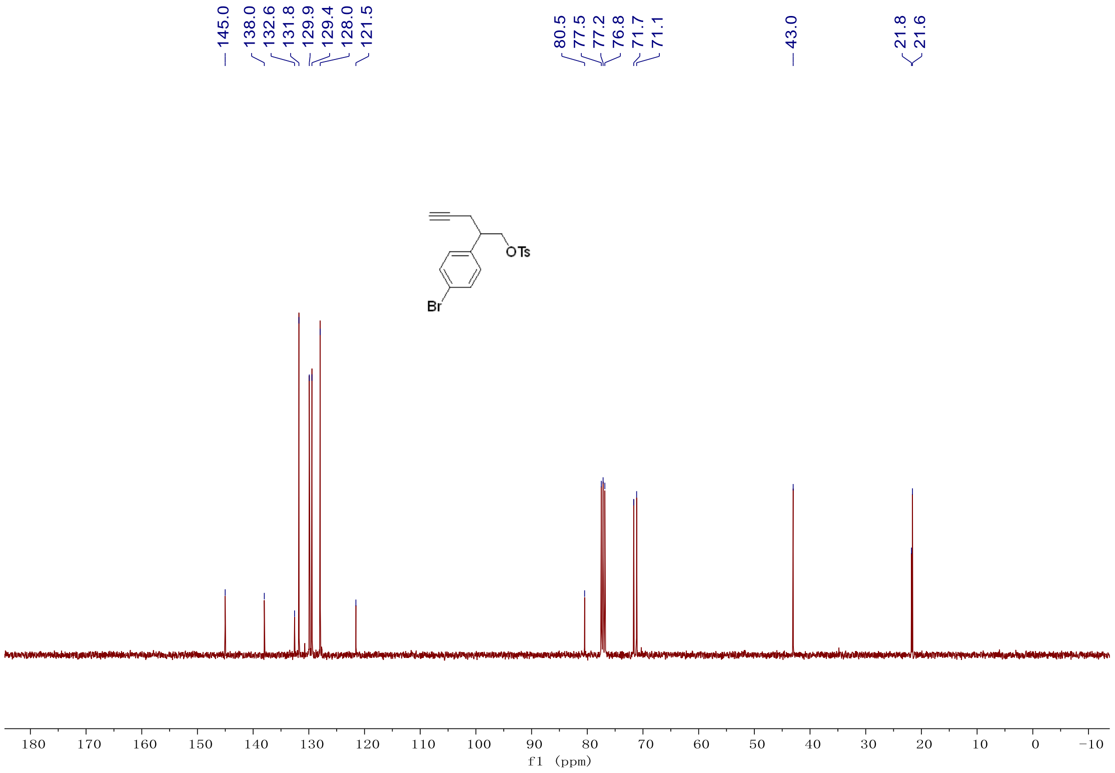

^{13}C NMR of compound 1i

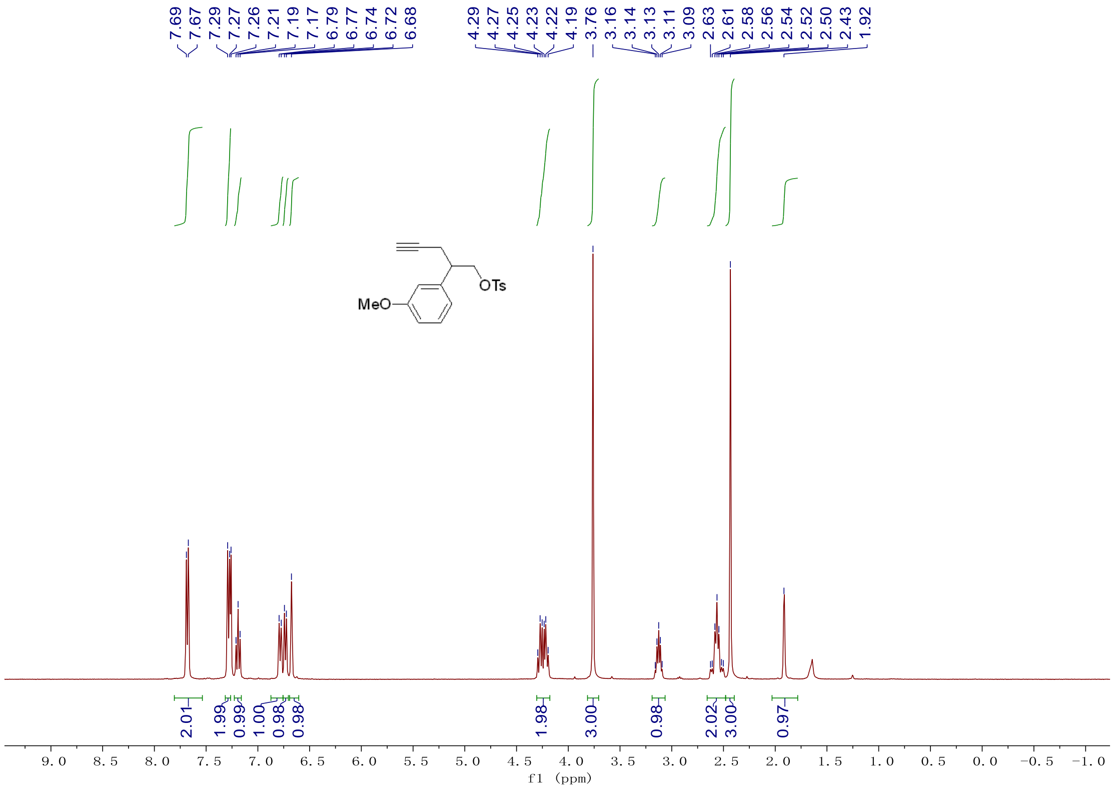

^{1}H NMR of compound 1j

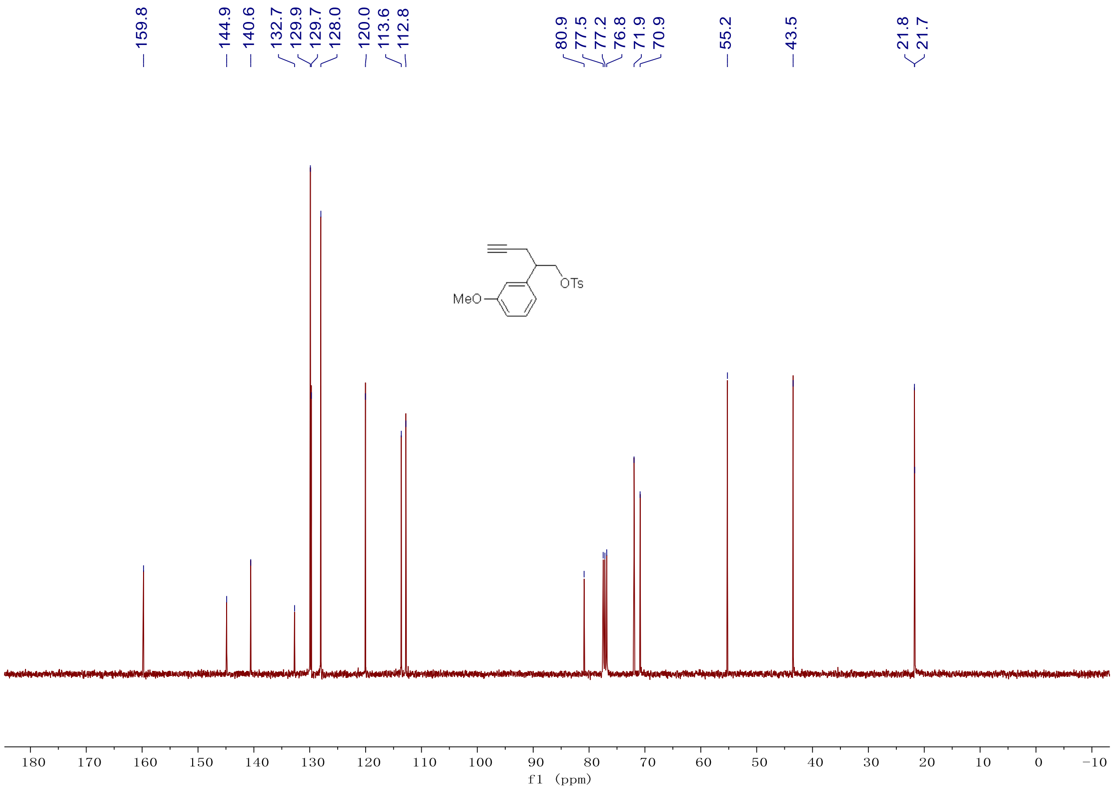

^{13}C NMR of compound 1j

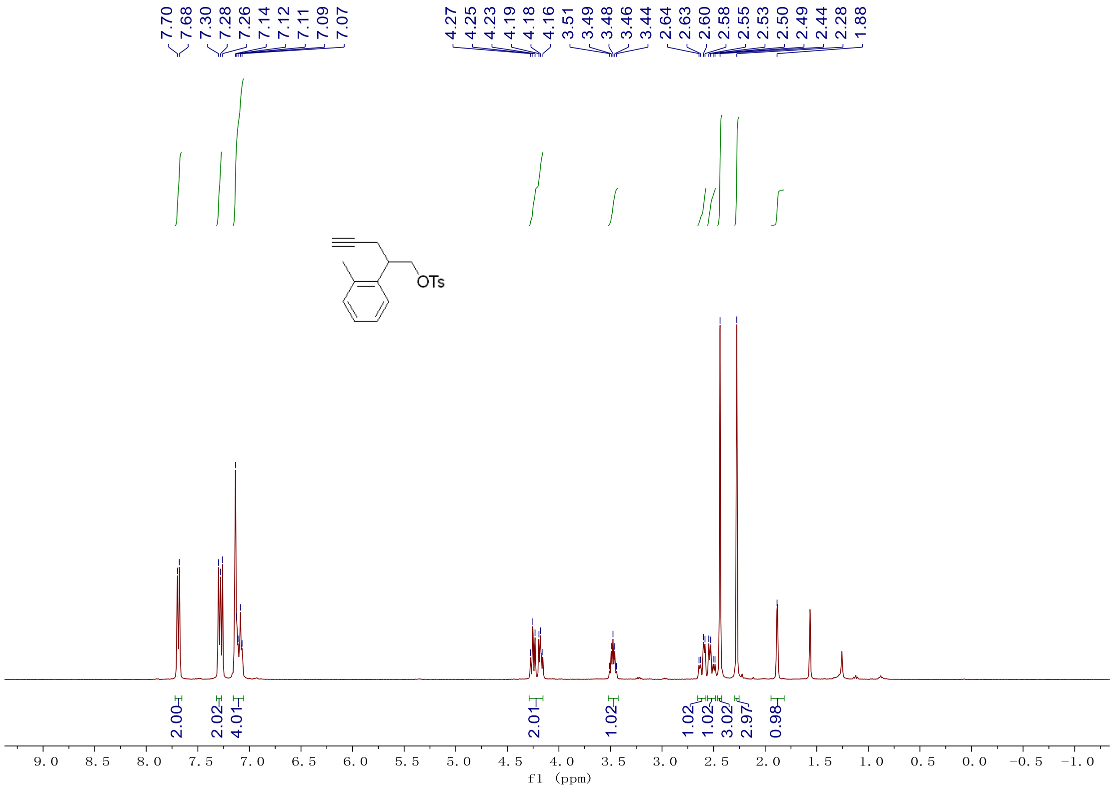

^{1}H NMR of compound 1k

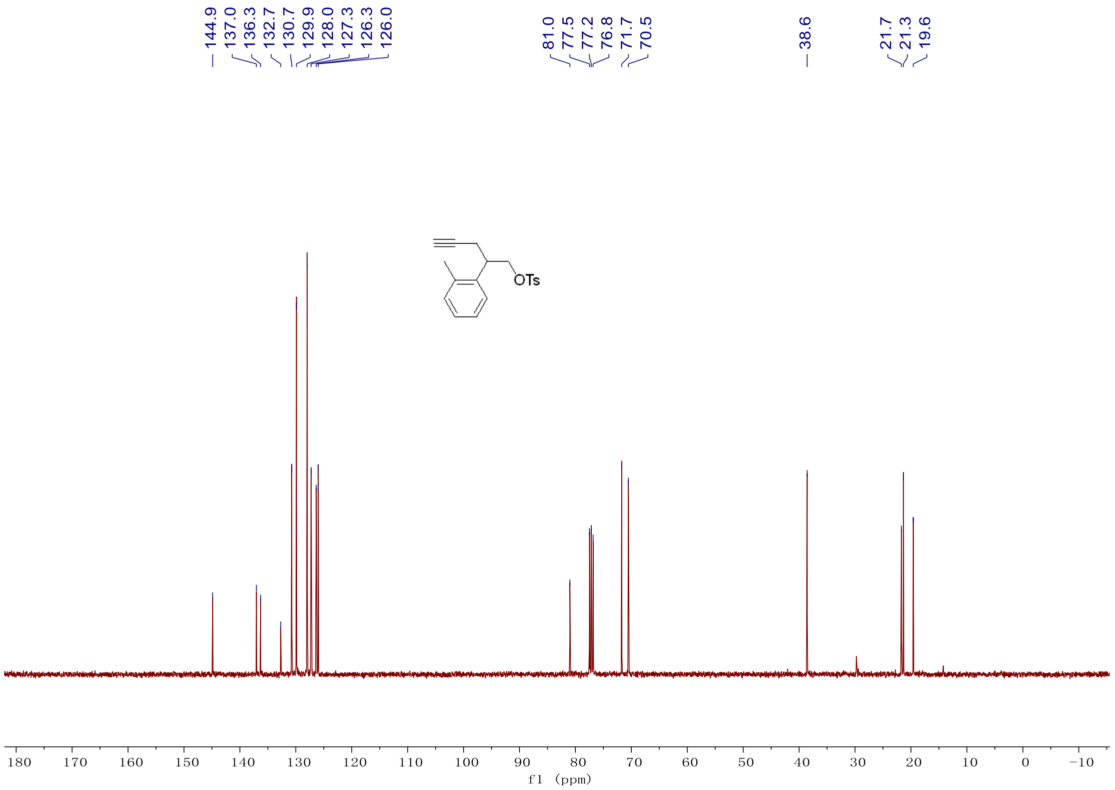

^{13}C NMR of compound 1k

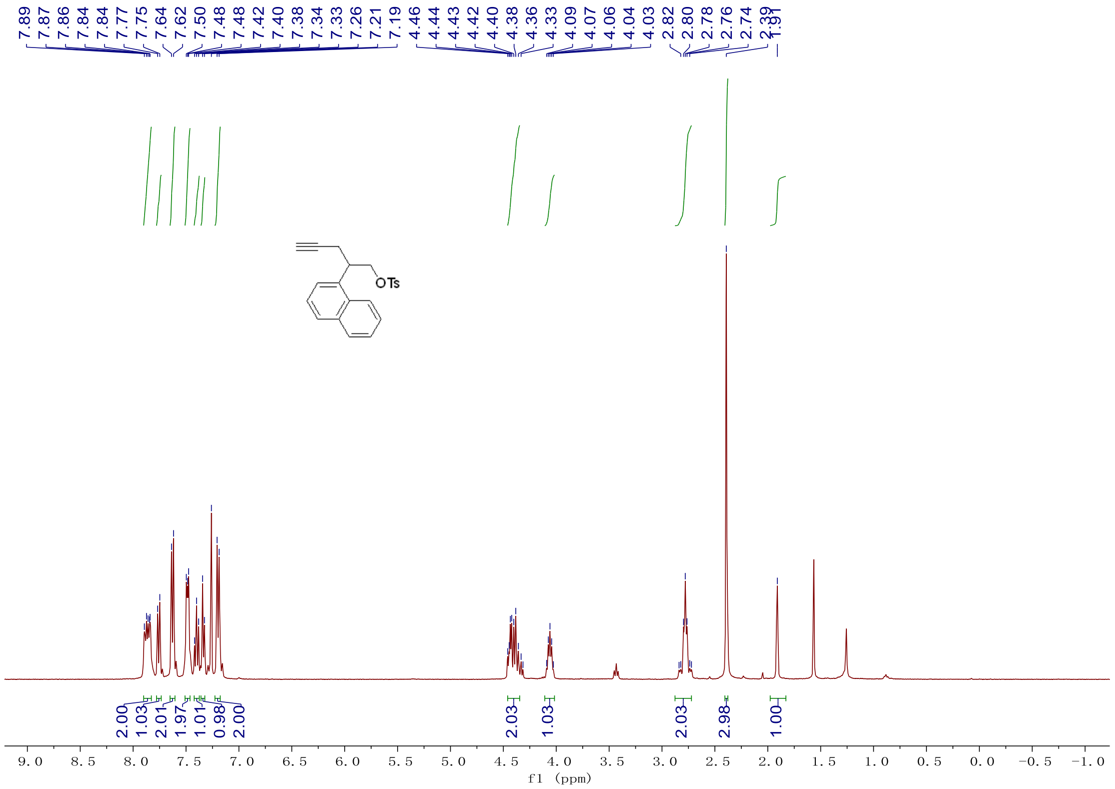

^{1}H NMR of compound 1l

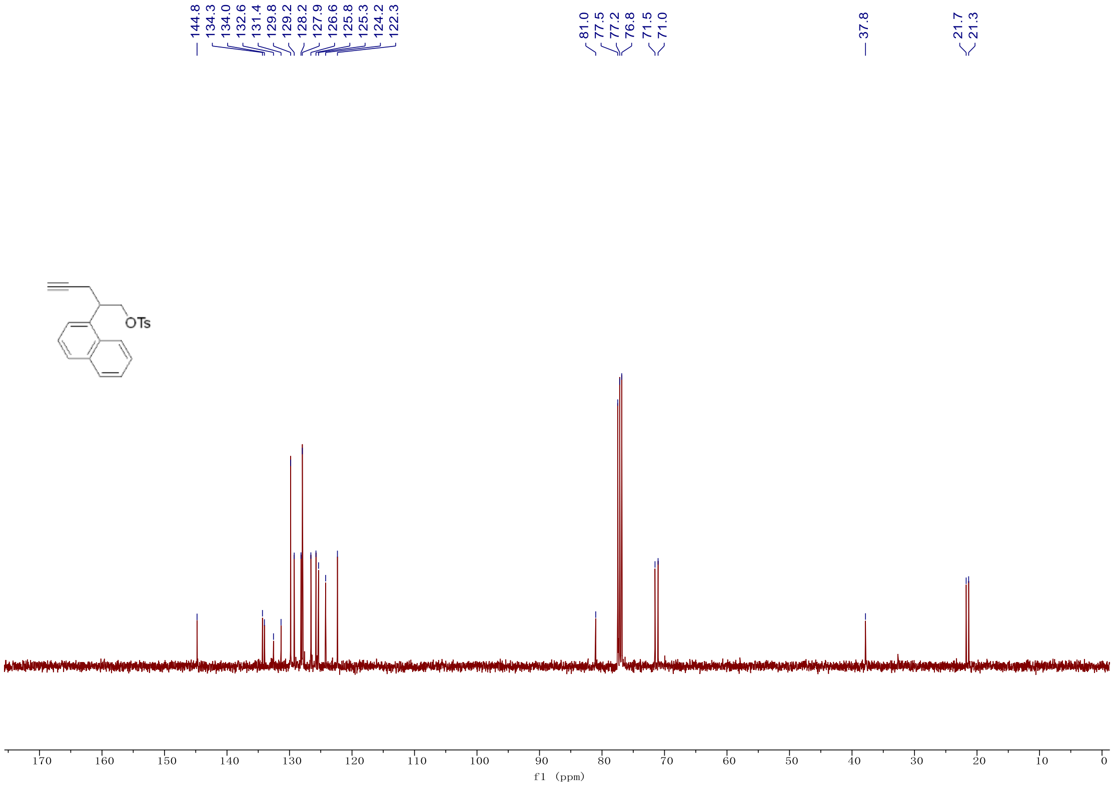

^{13}C NMR of compound 1l

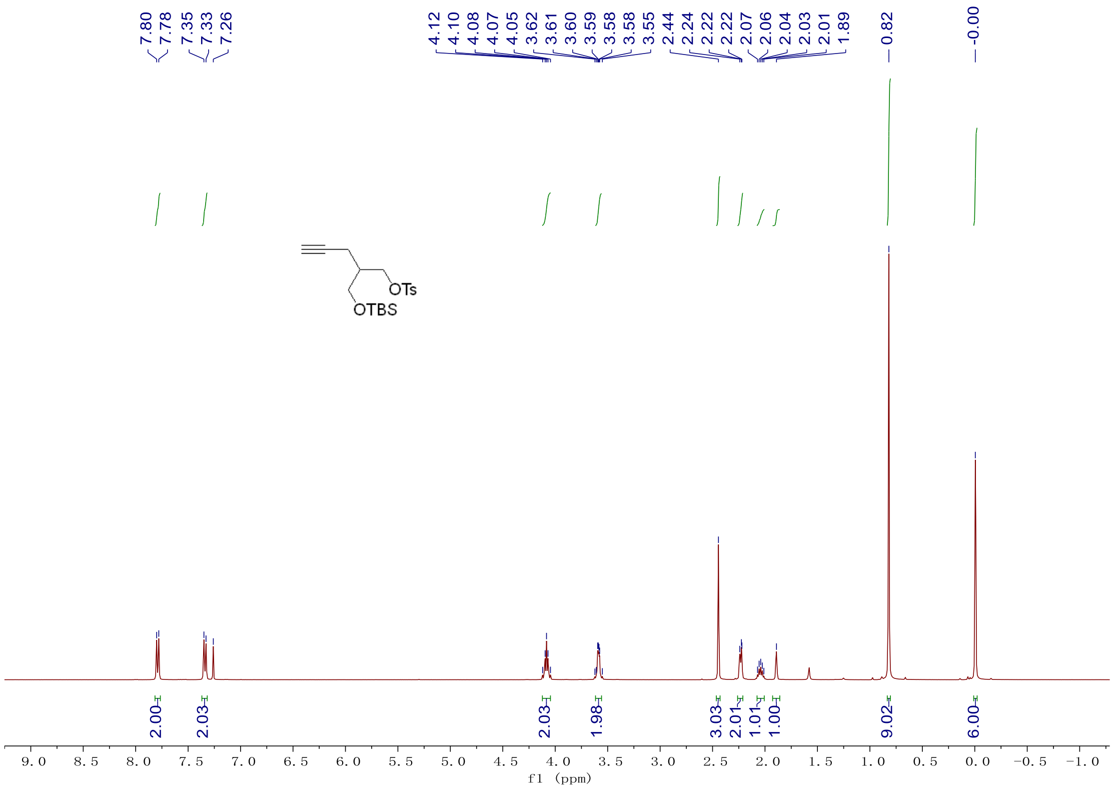

^{1}H NMR of compound 1n

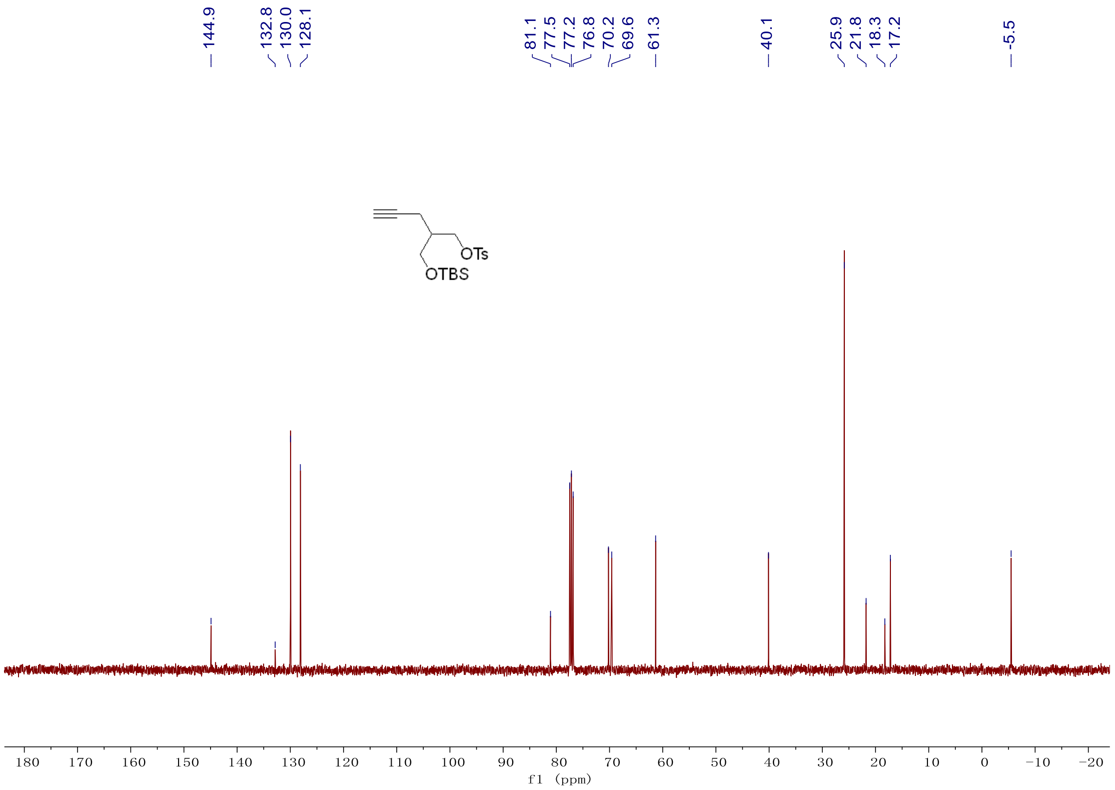

^{13}C NMR of compound 1n

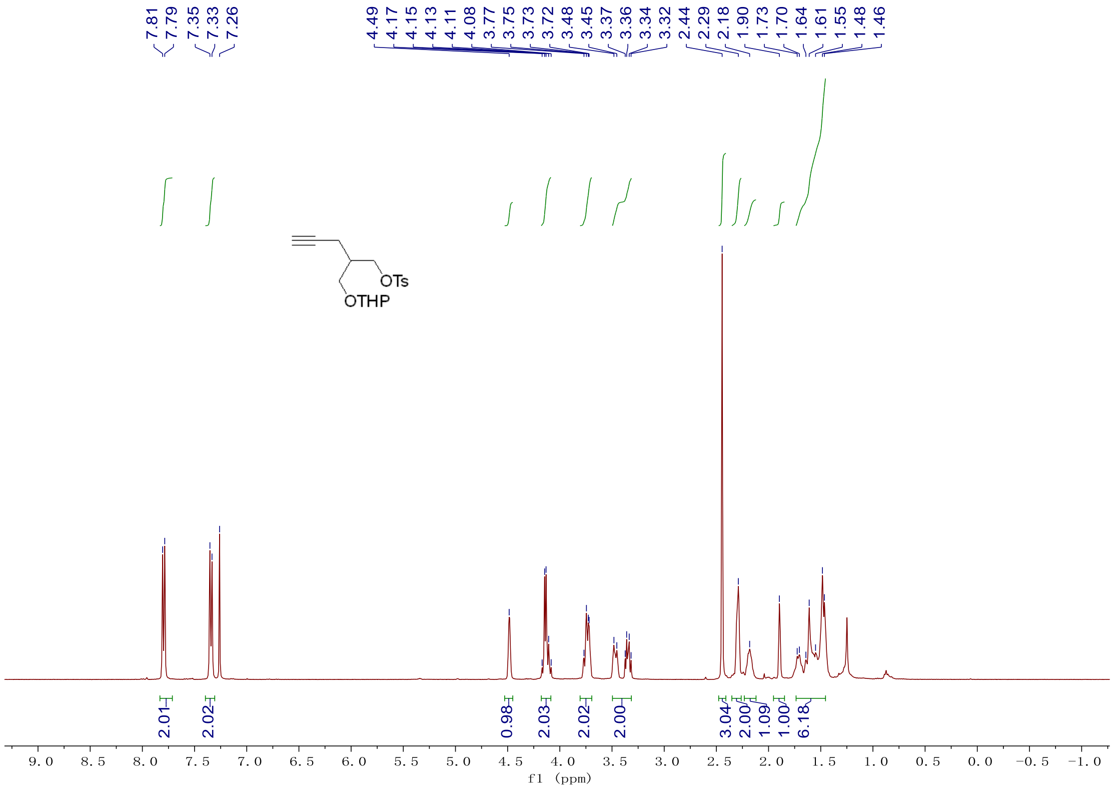

^{1}H NMR of compound 1o

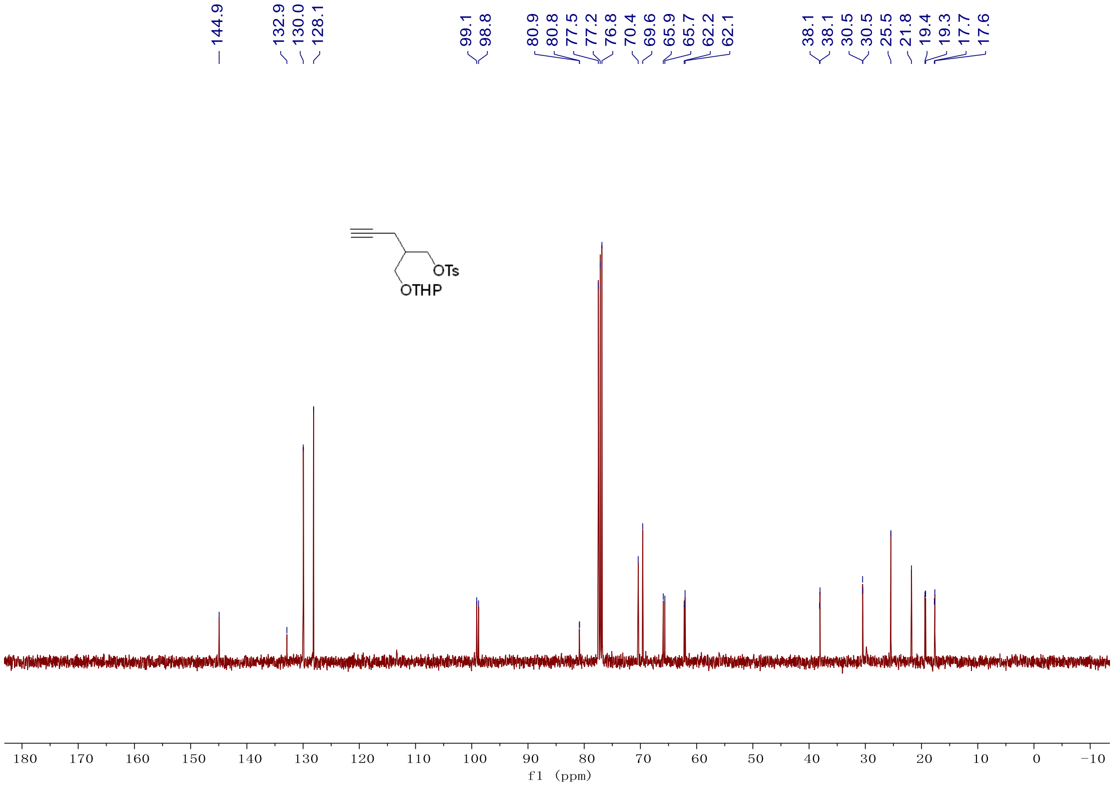

^{13}C NMR of compound 1o

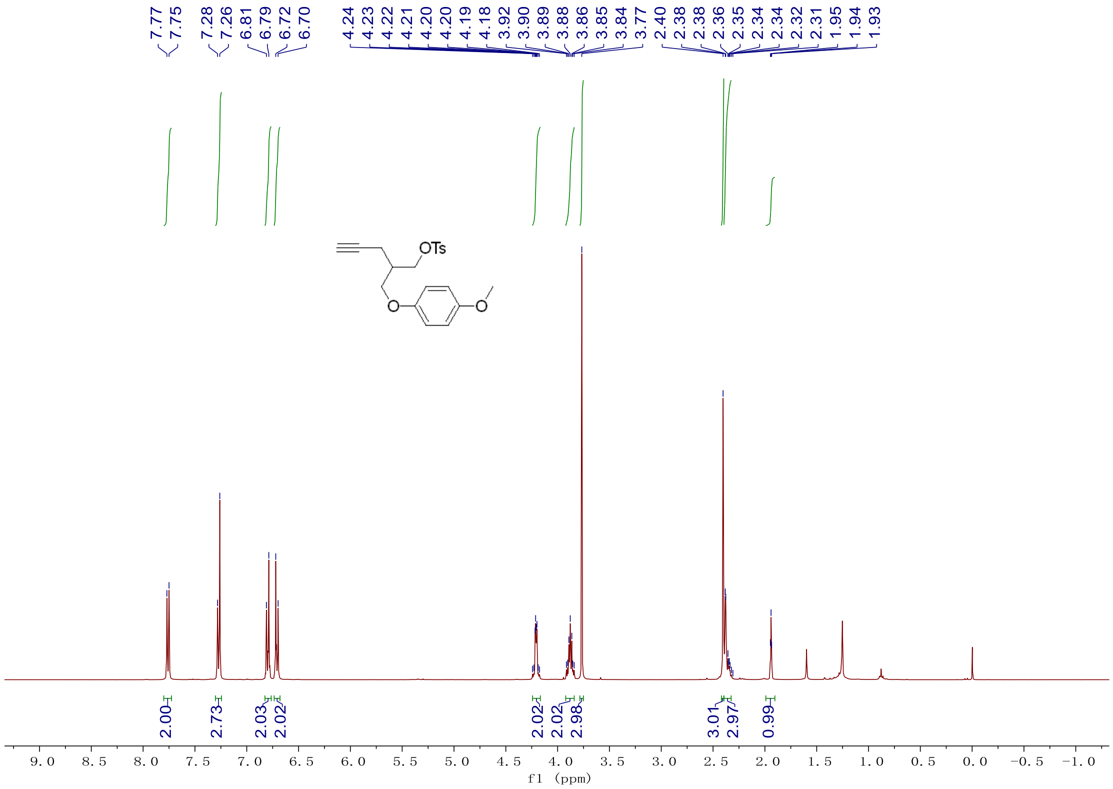

¹H NMR of compound 1p

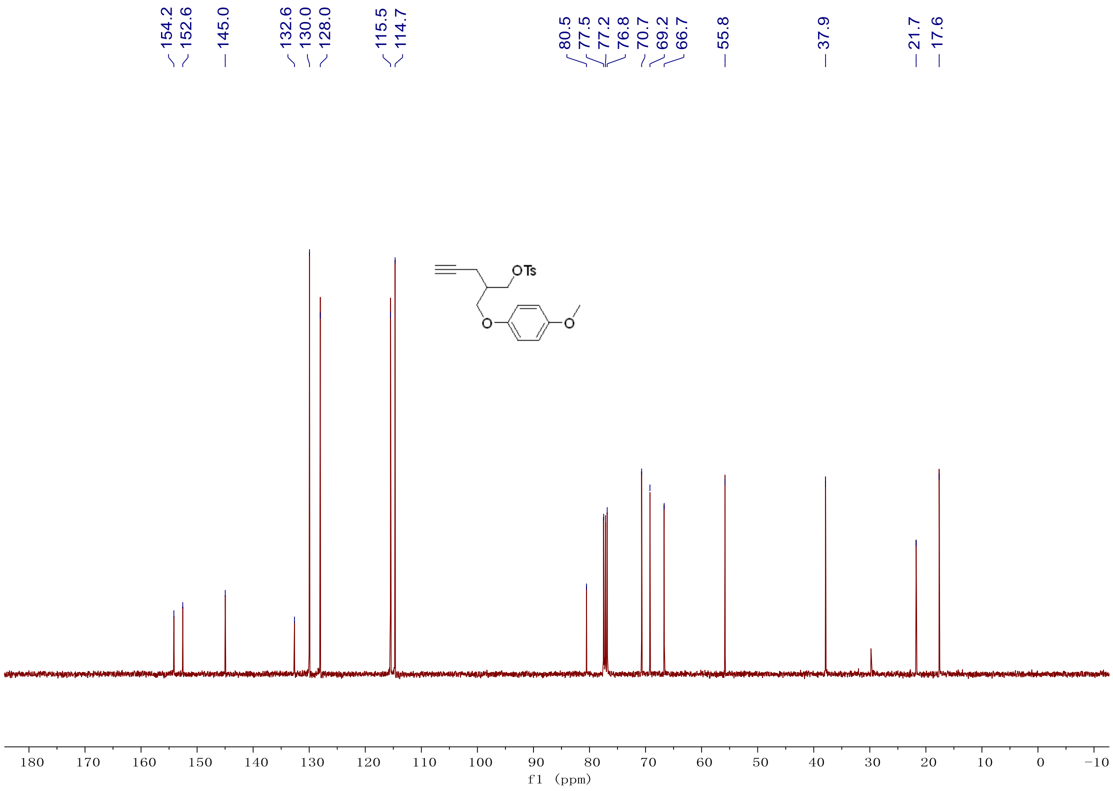

¹³C NMR of compound 1p

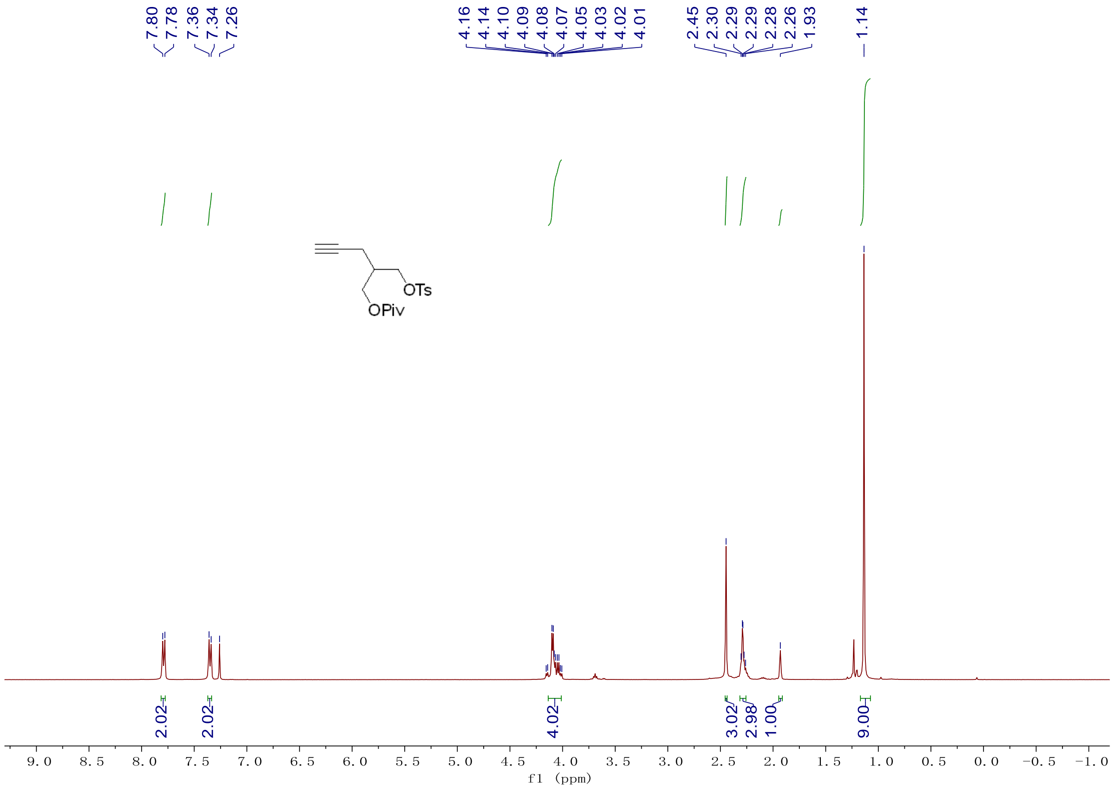

^{1}H NMR of compound 1q

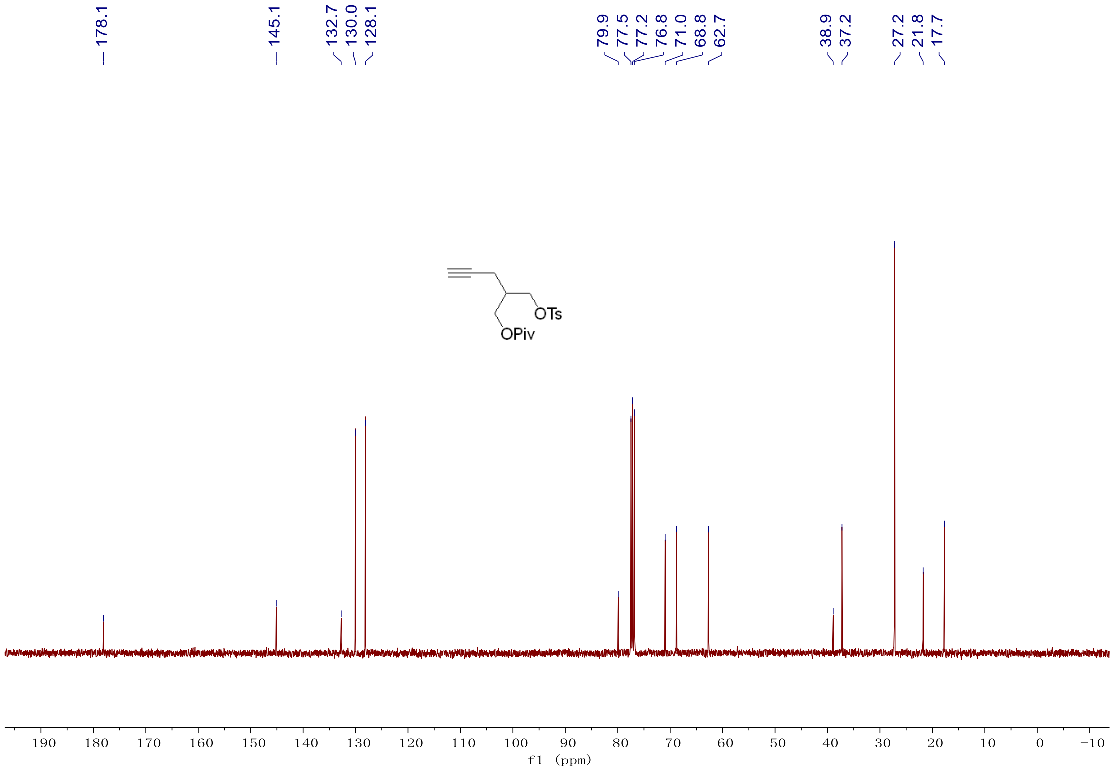

^{13}C NMR of compound 1q

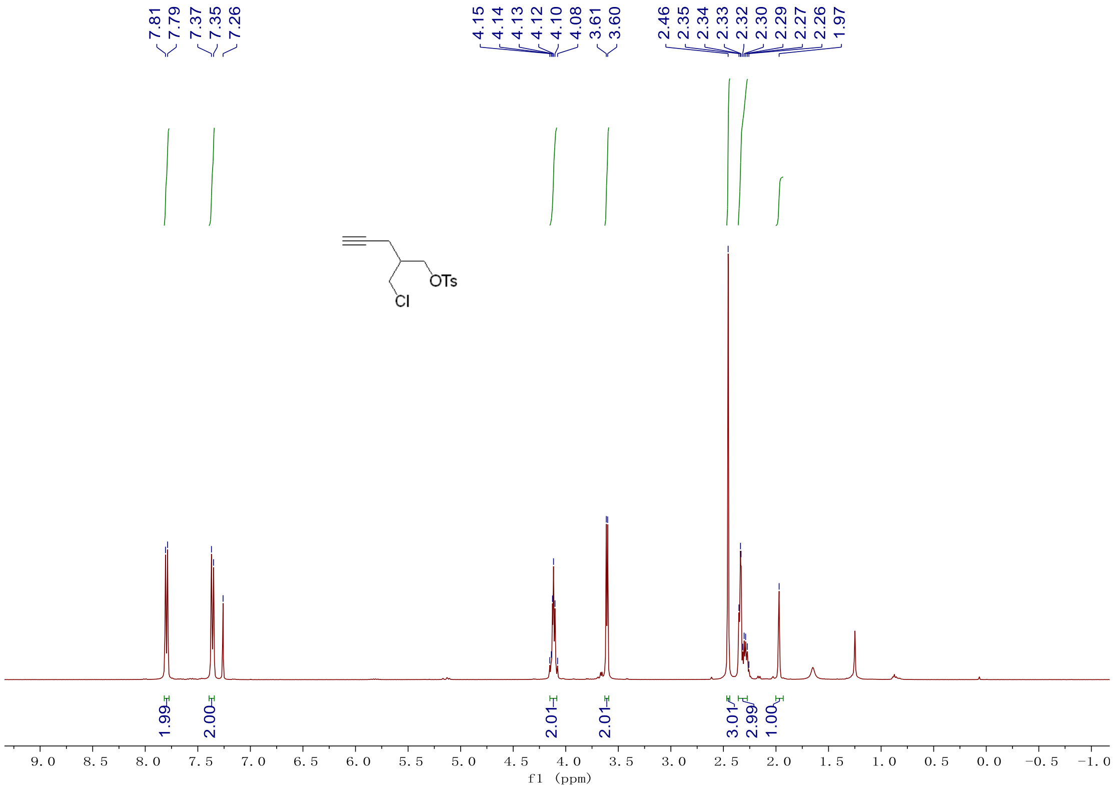

^{1}H NMR of compound 1r

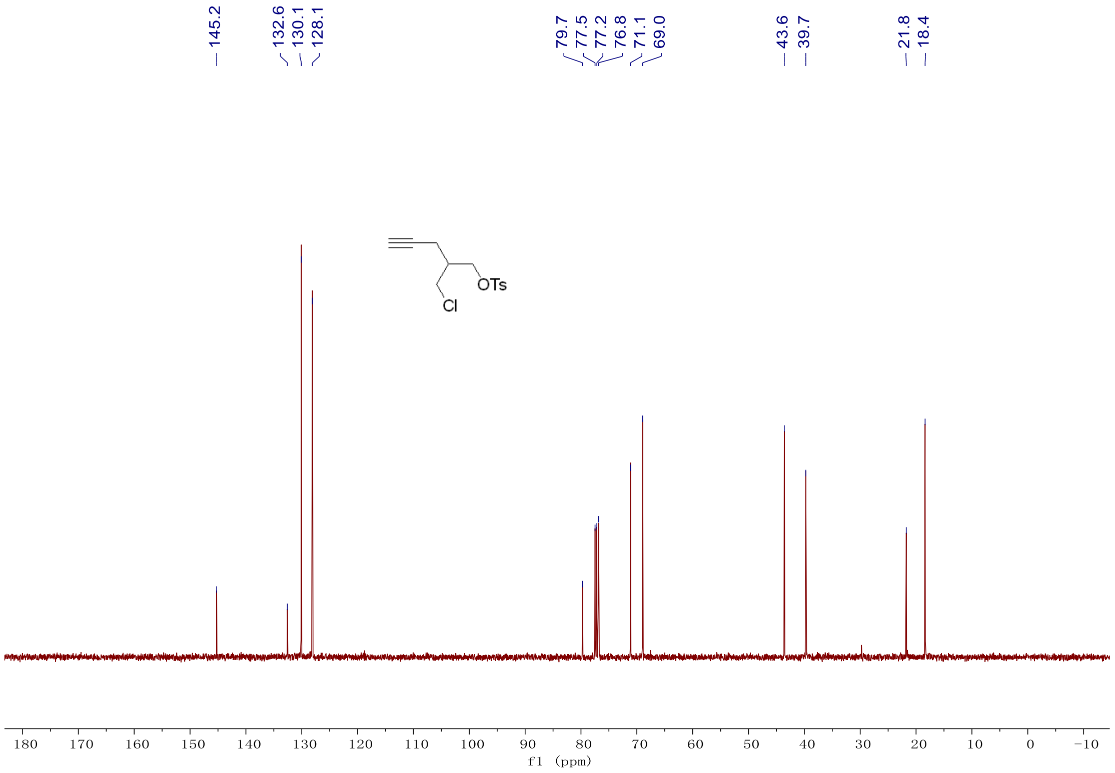

^{13}C NMR of compound 1r

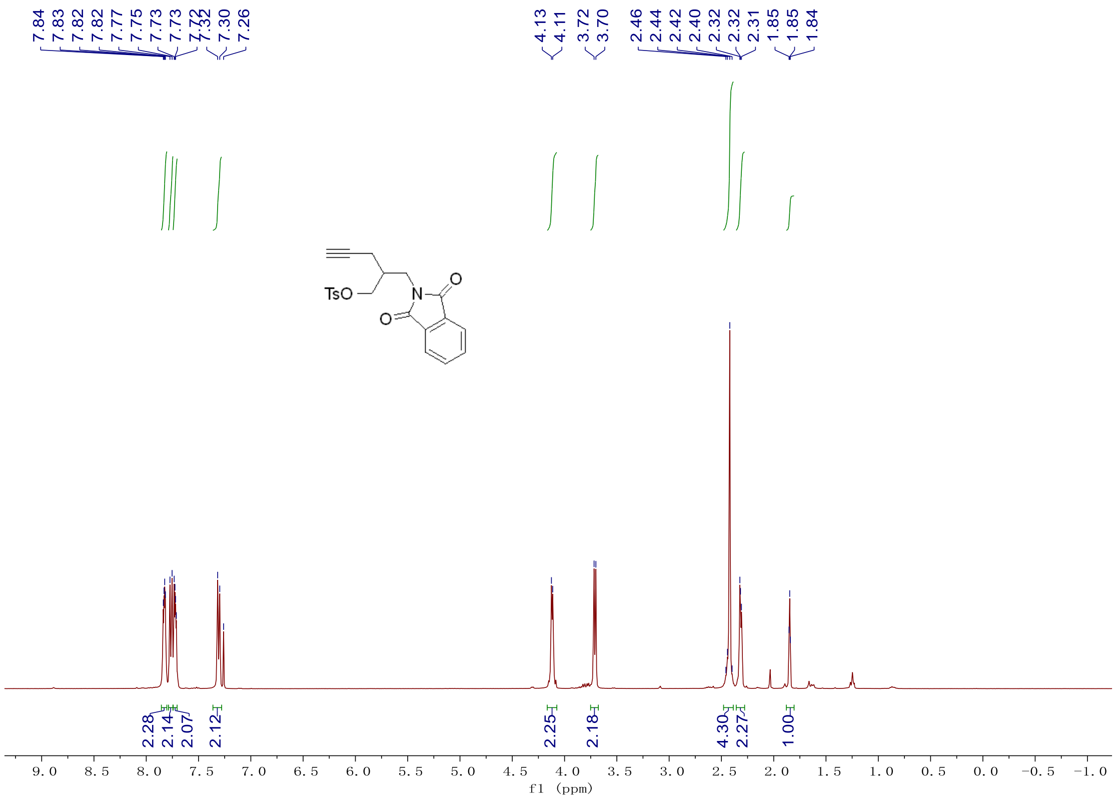

^{1}H NMR of compound 1s

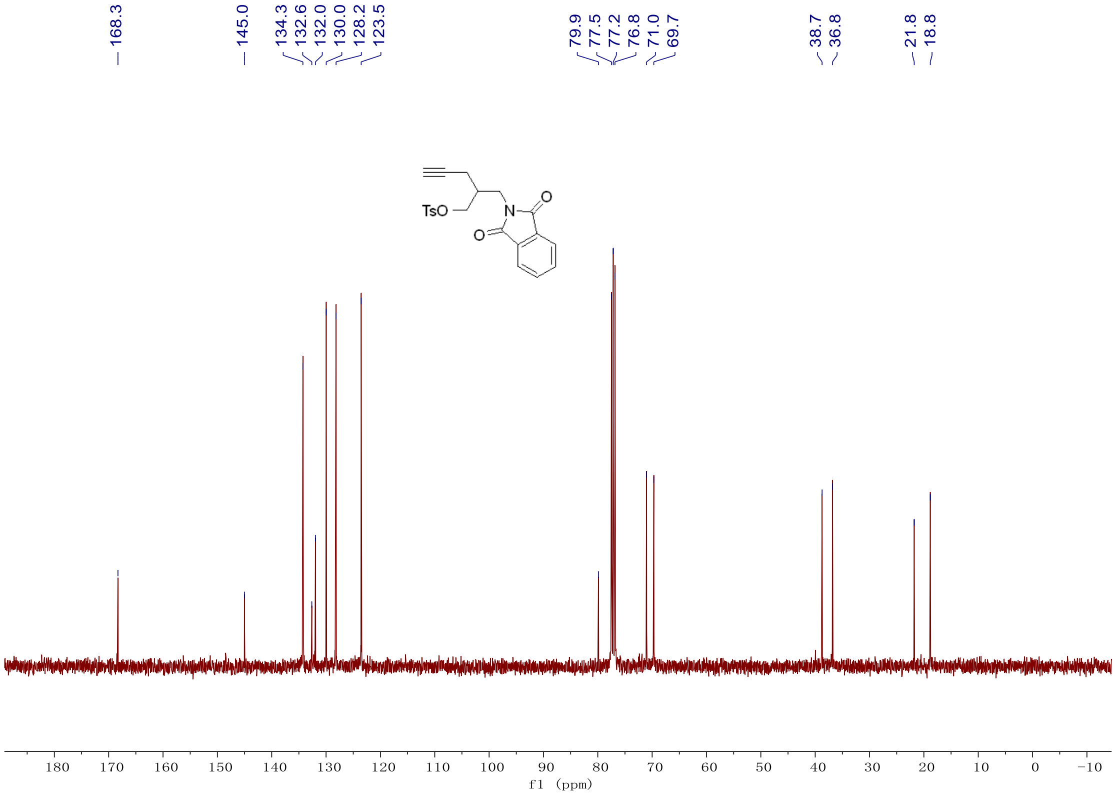

^{13}C NMR of compound 1s

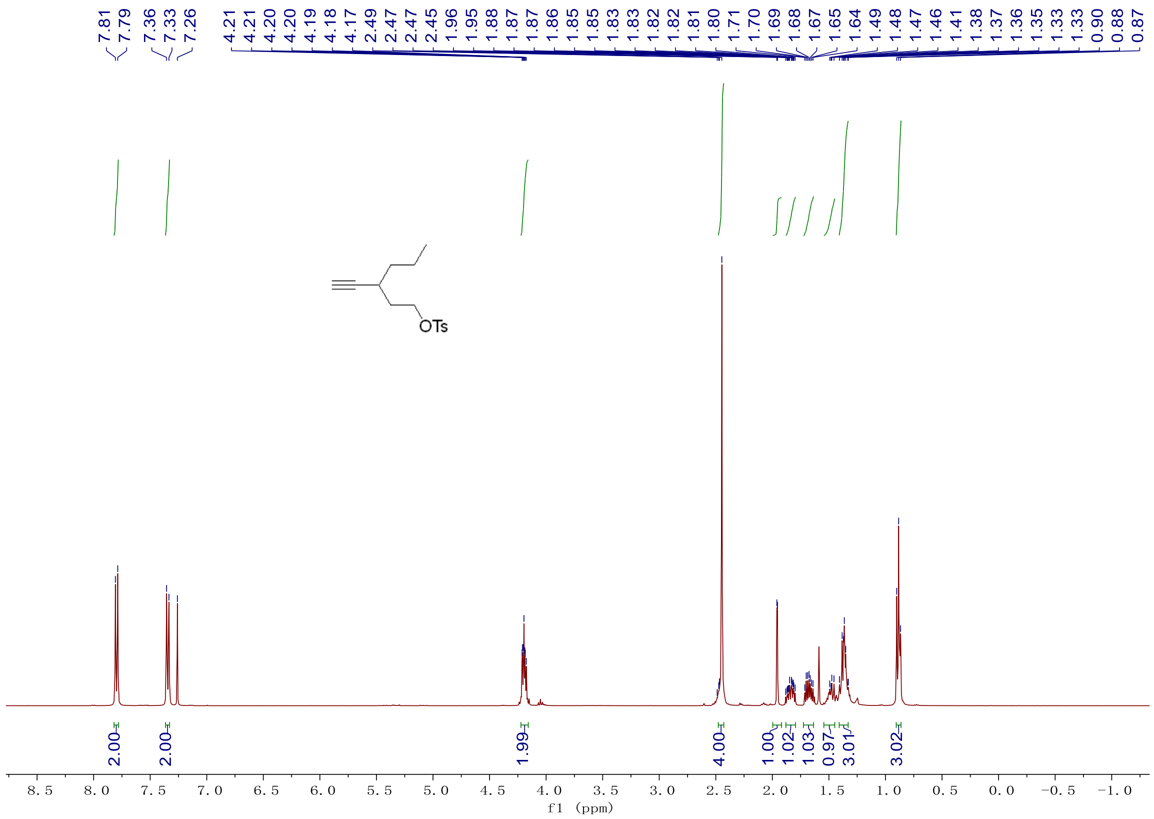

^{1}H NMR of compound 1u

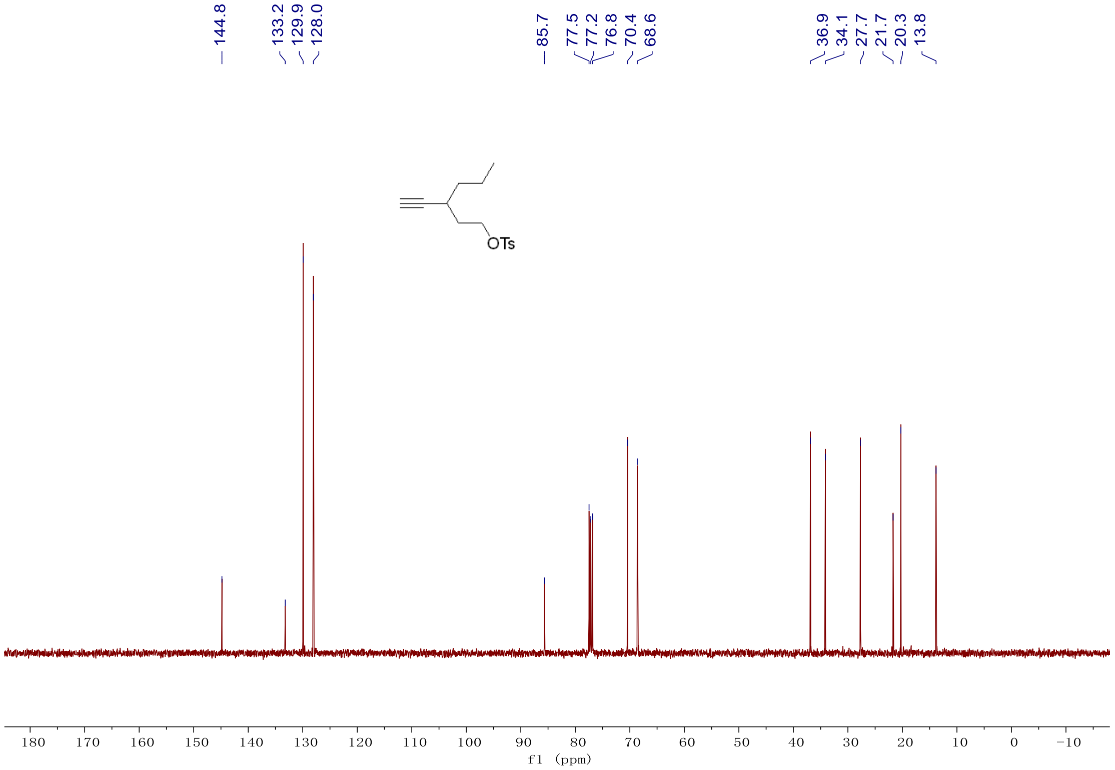

^{13}C NMR of compound 1u

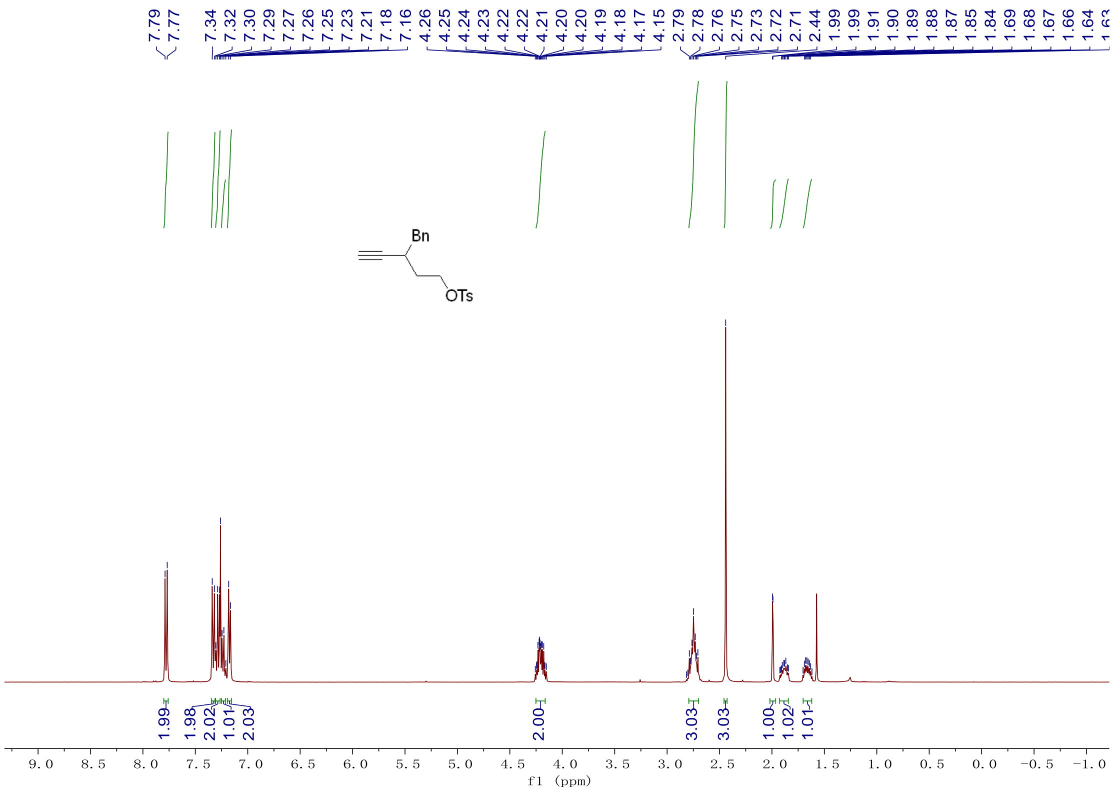

^{1}H NMR of compound 1v

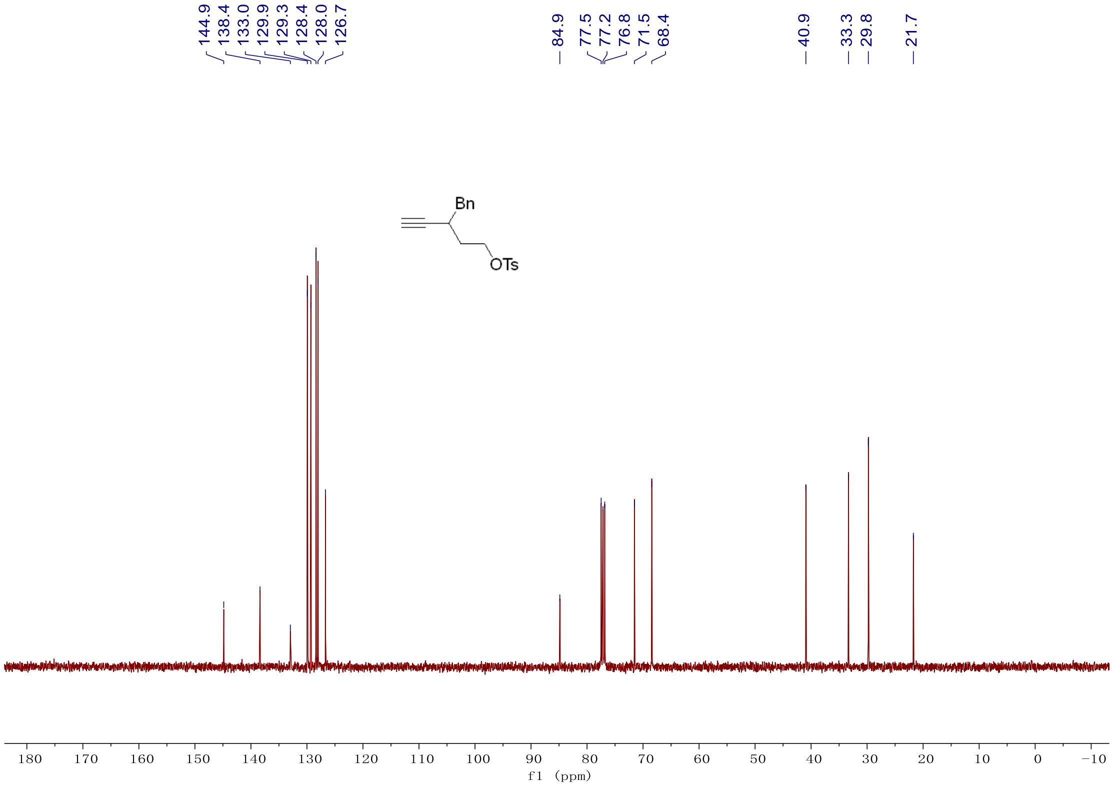

^{13}C NMR of compound 1v

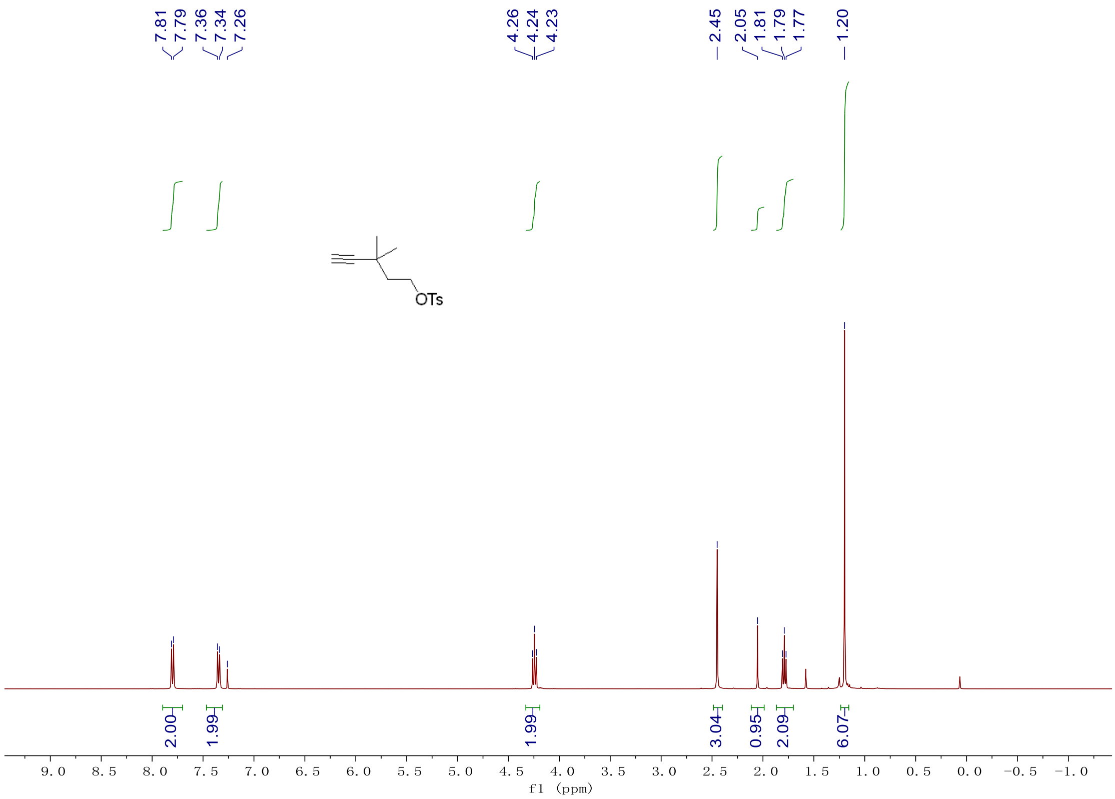

^{1}H NMR of compound 1w

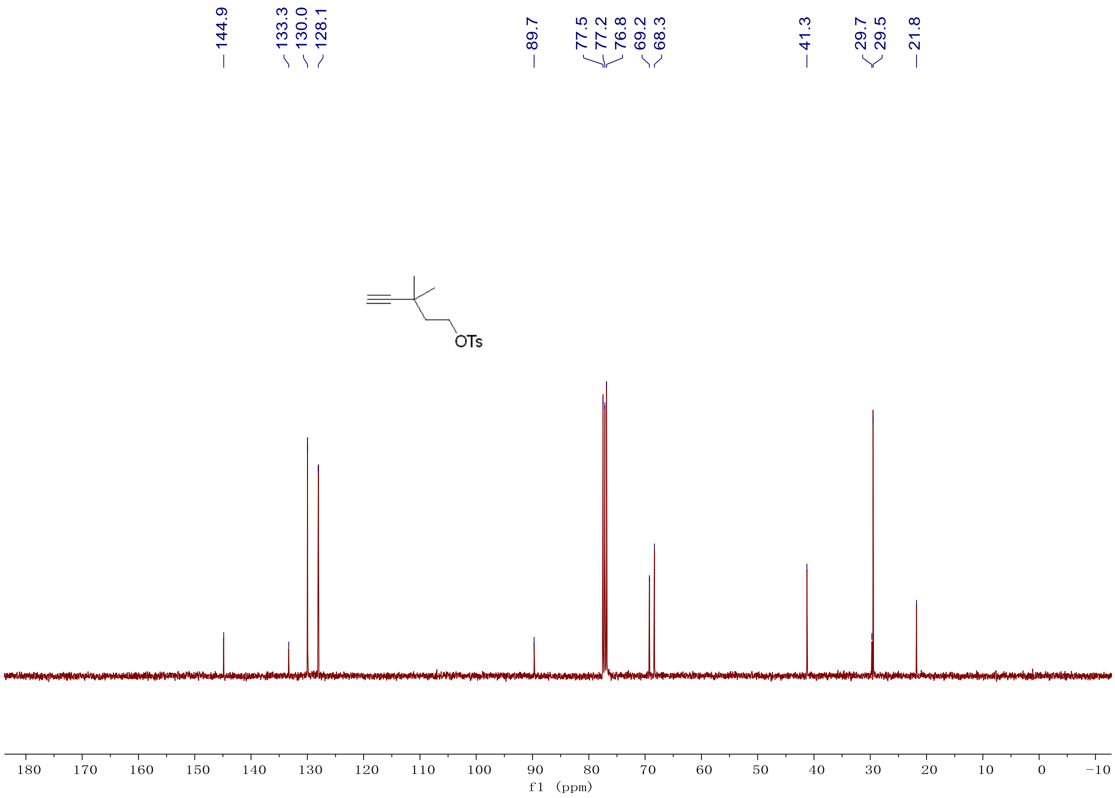

^{13}C NMR of compound 1w

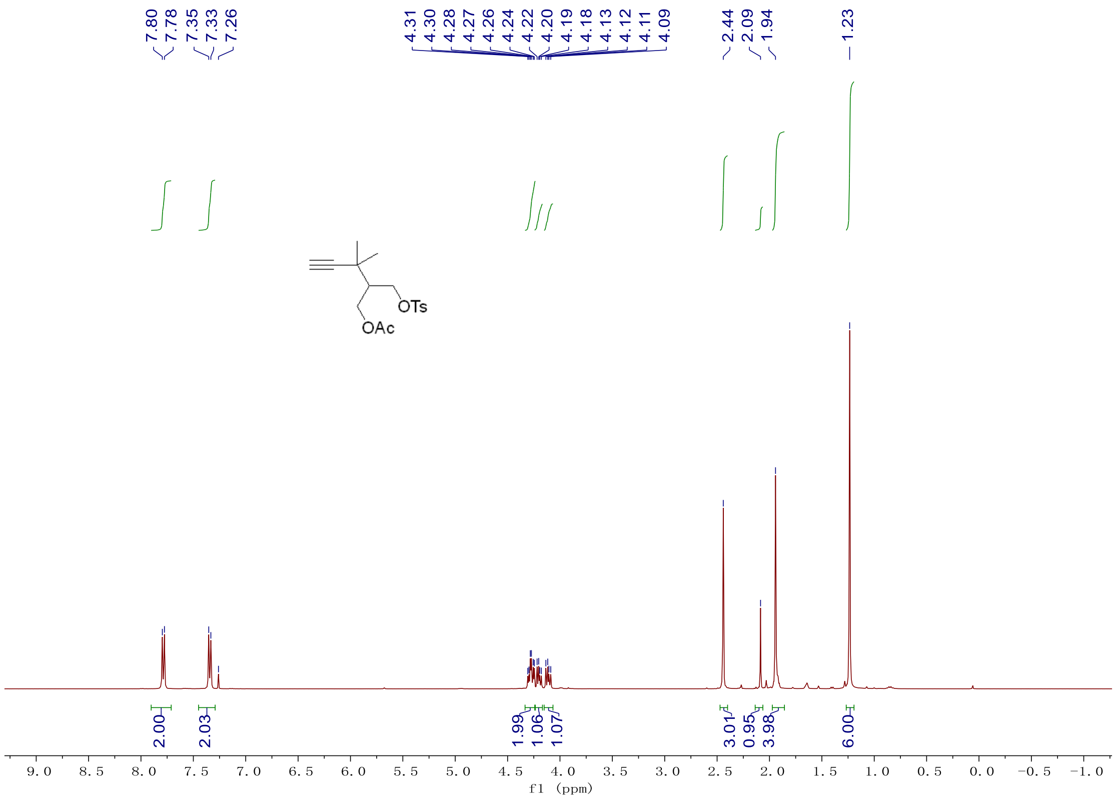

^{1}H NMR of compound 1x

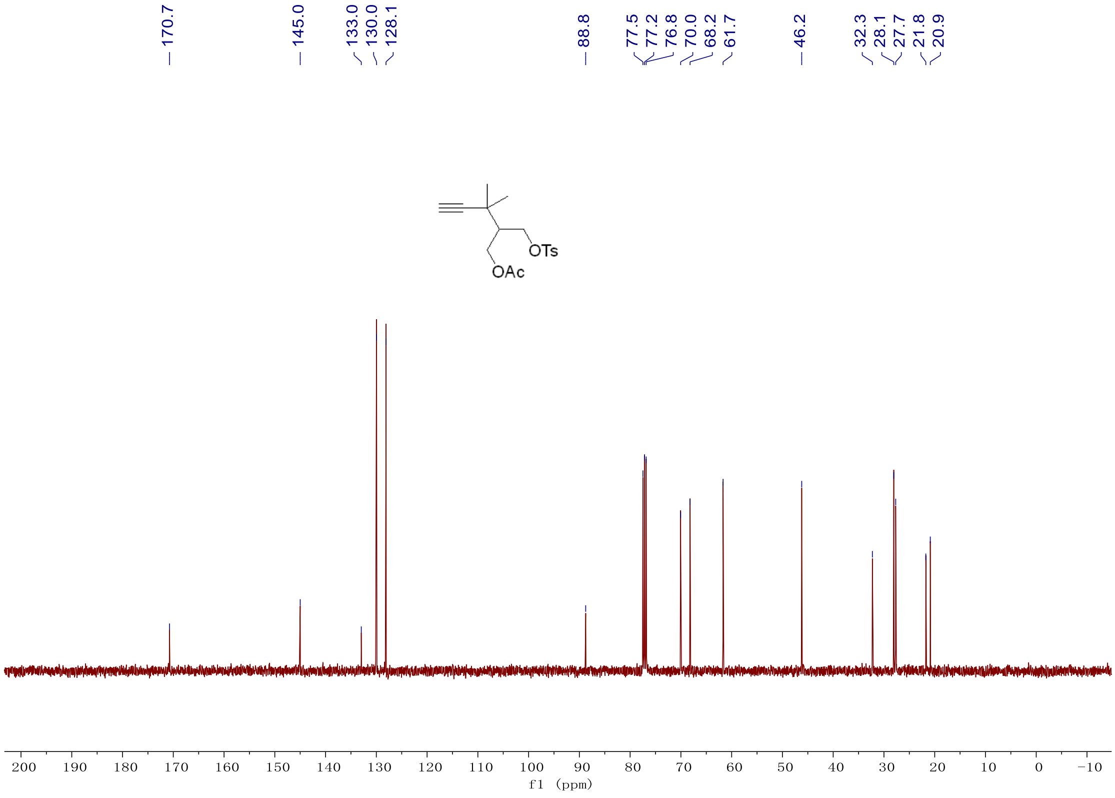

^{13}C NMR of compound 1x

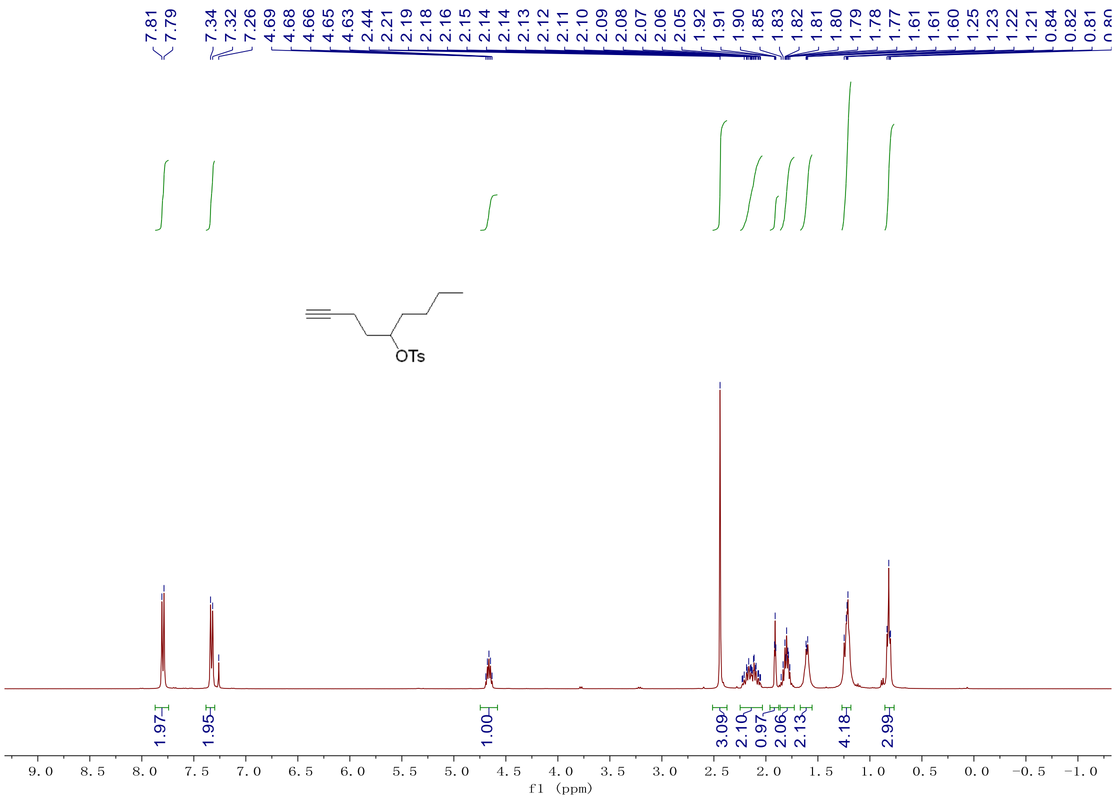

^{1}H NMR of compound 1y

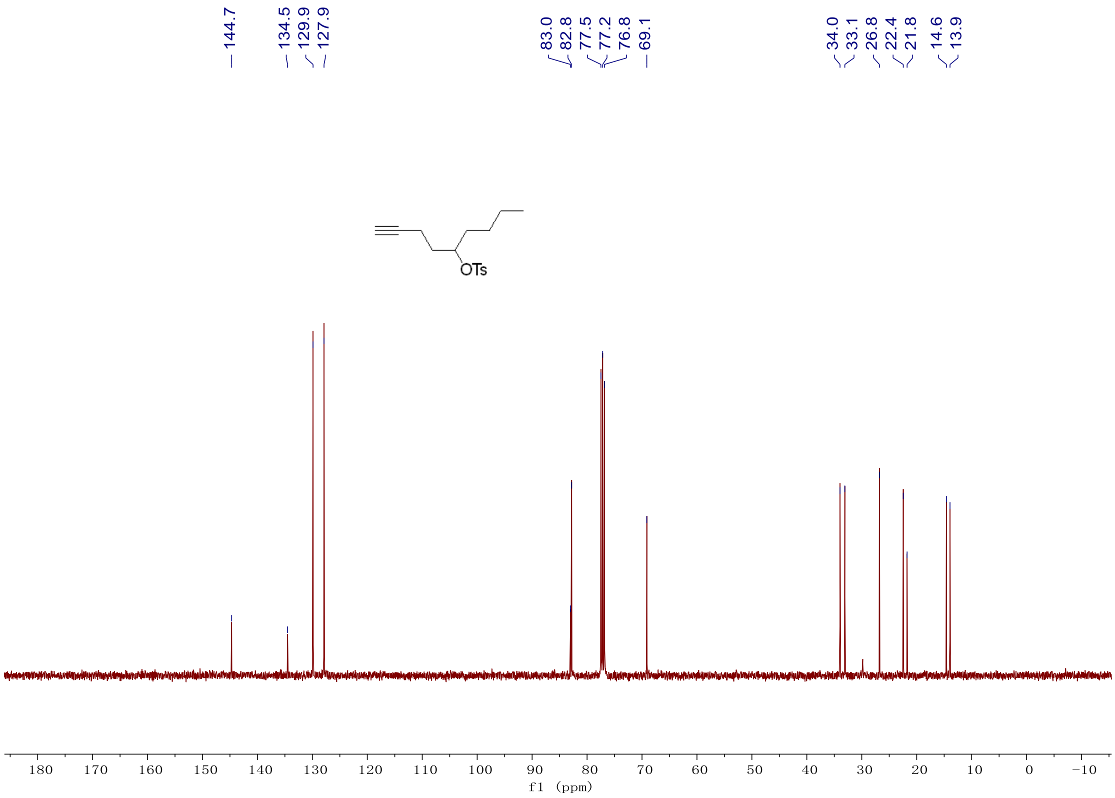

^{13}C NMR of compound 1y

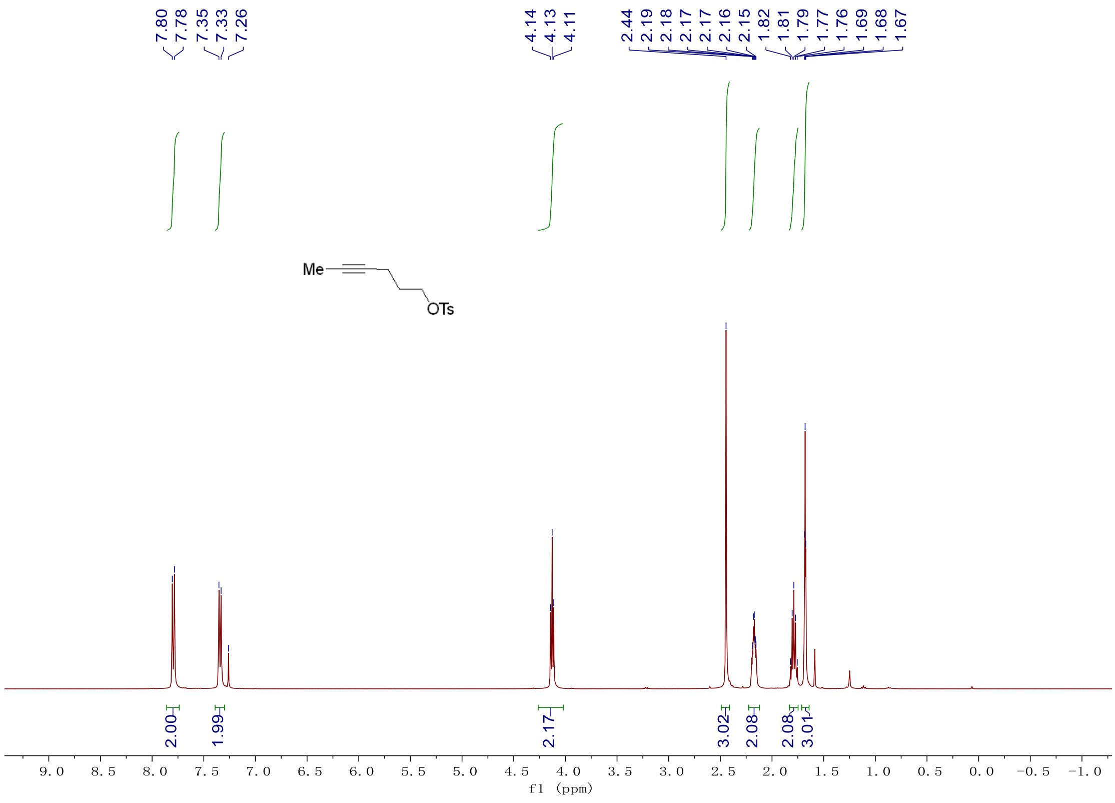

^{1}H NMR of compound 1z

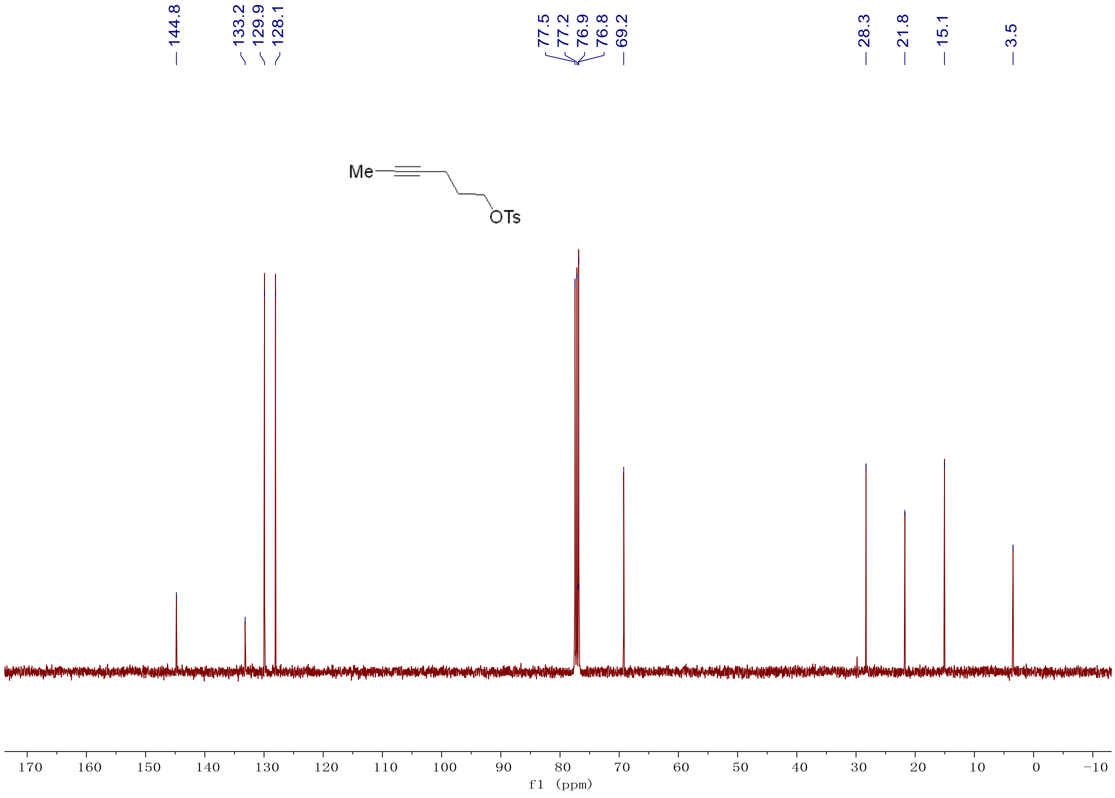

^{13}C NMR of compound 1z

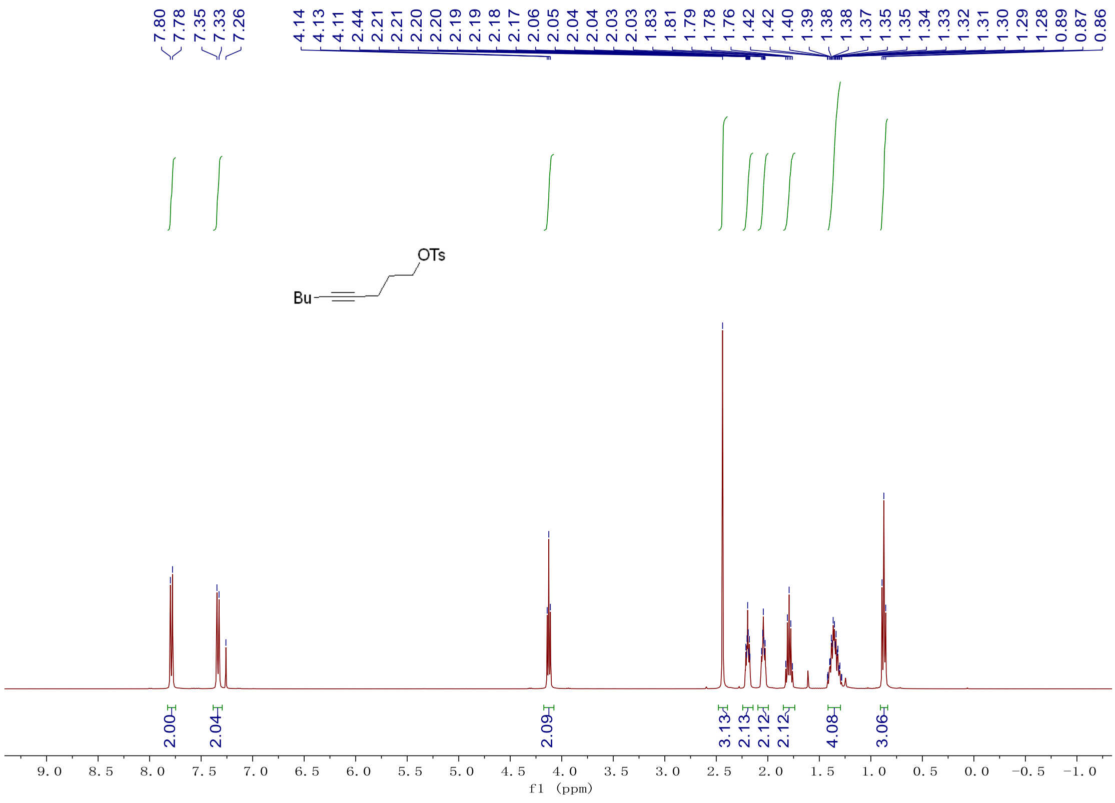

^{1}H NMR of compound 1aa

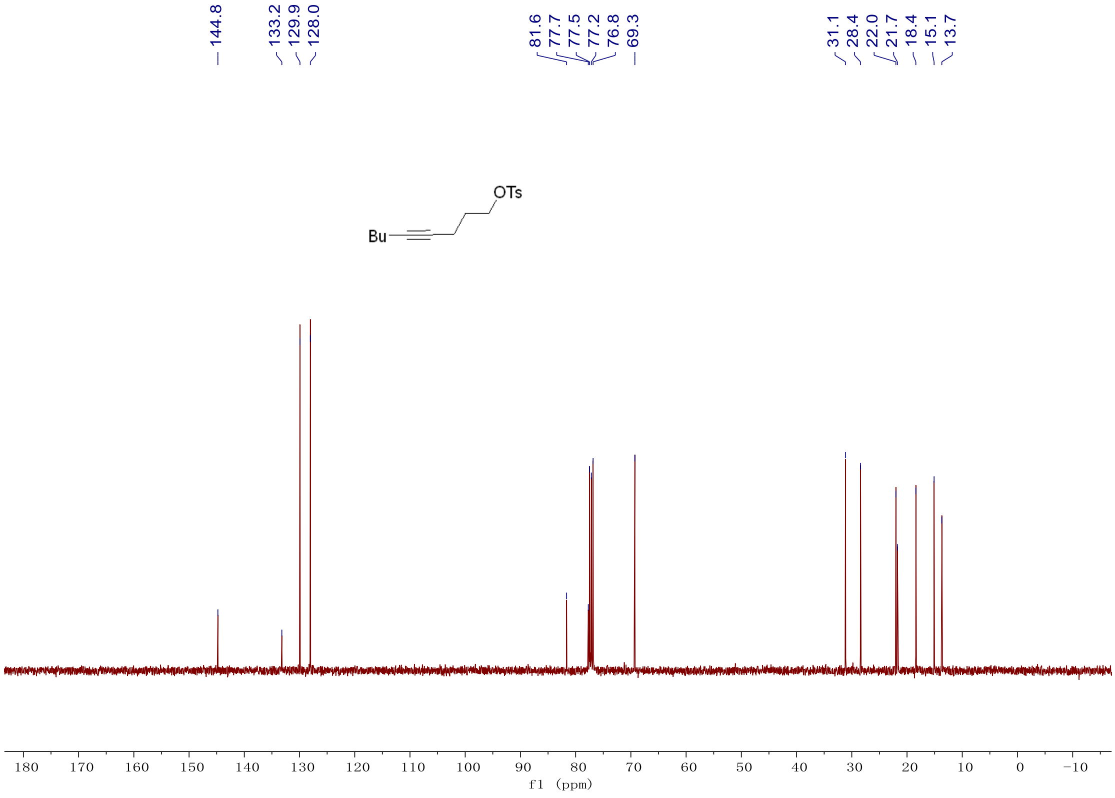

^{13}C NMR of compound 1aa

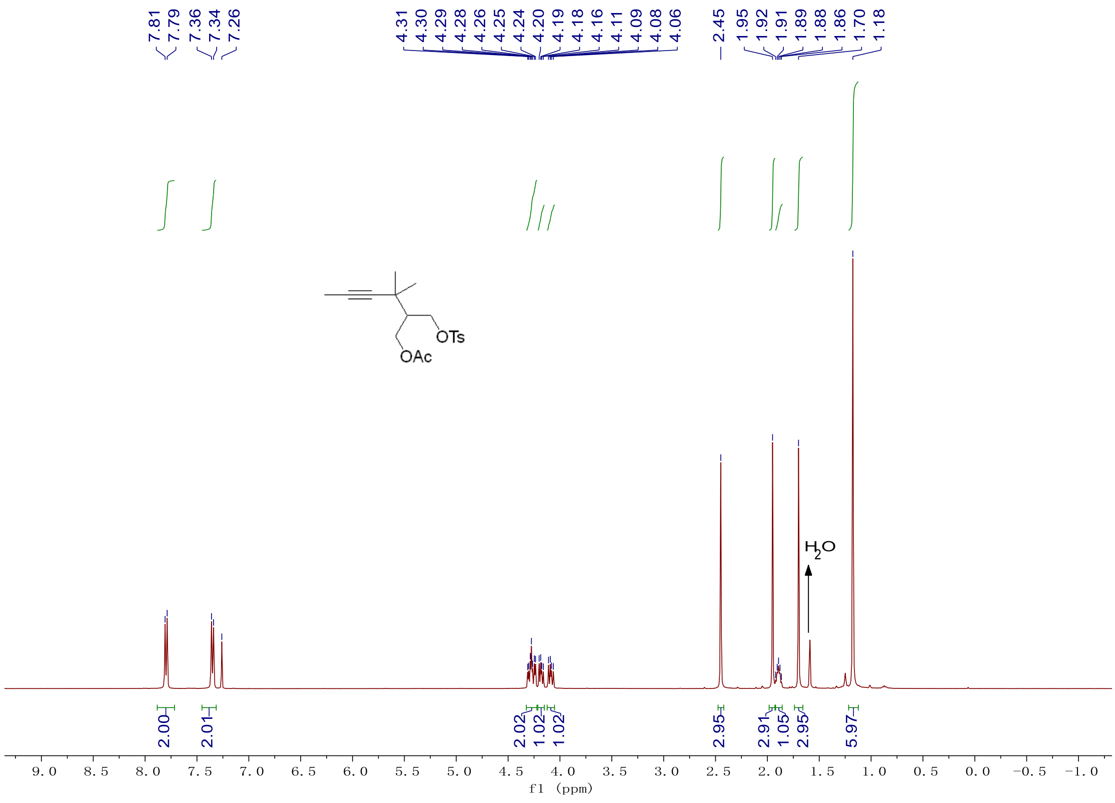

^{1}H NMR of compound 1ab

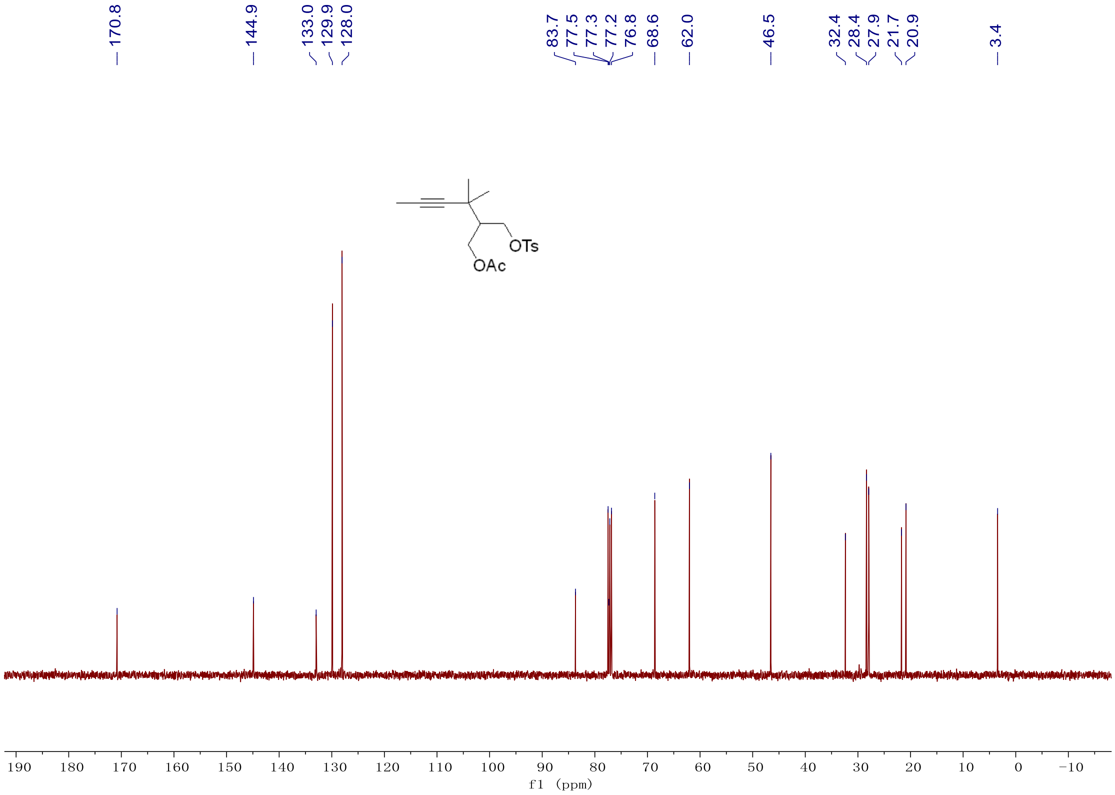

^{13}C NMR of compound 1ab

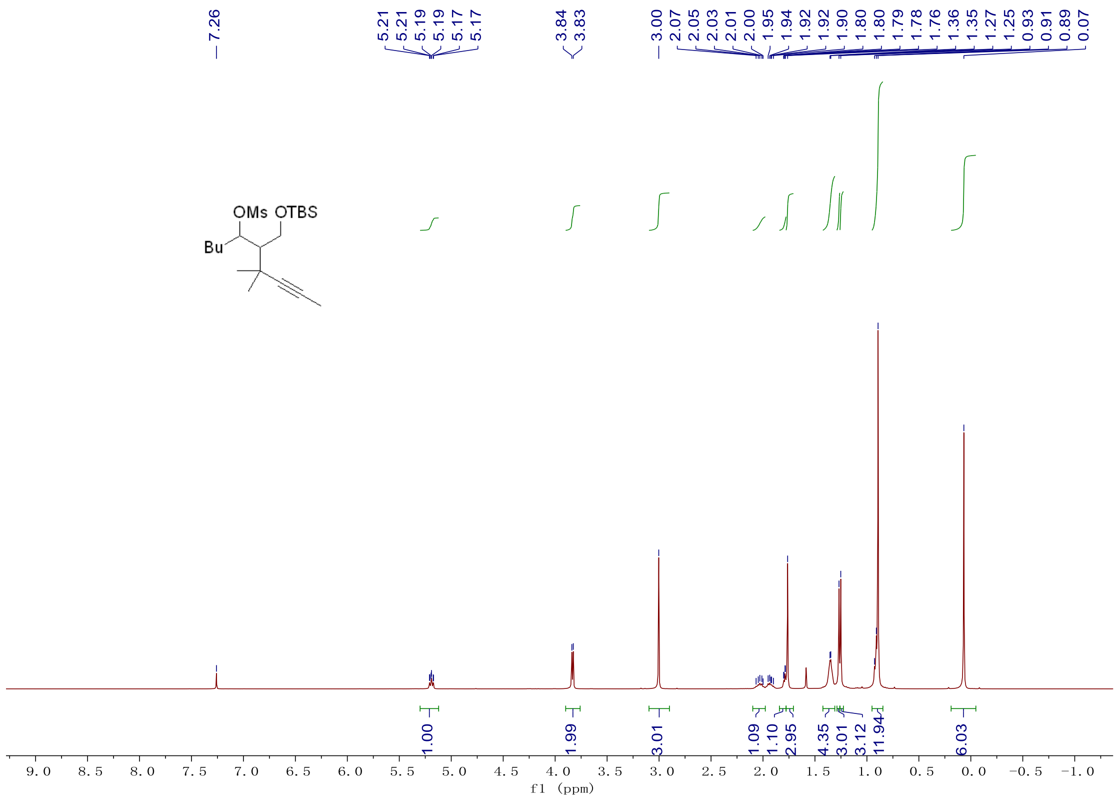

^{1}H NMR of compound 1ac

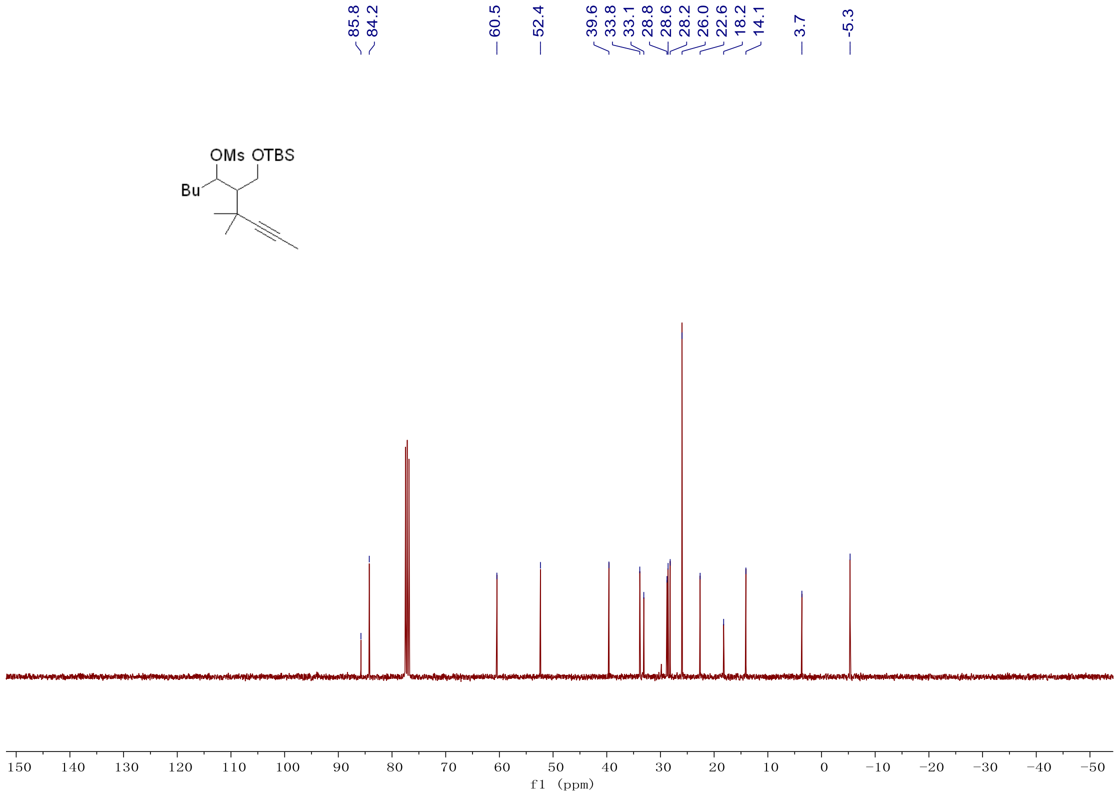

^{13}C NMR of compound 1ac

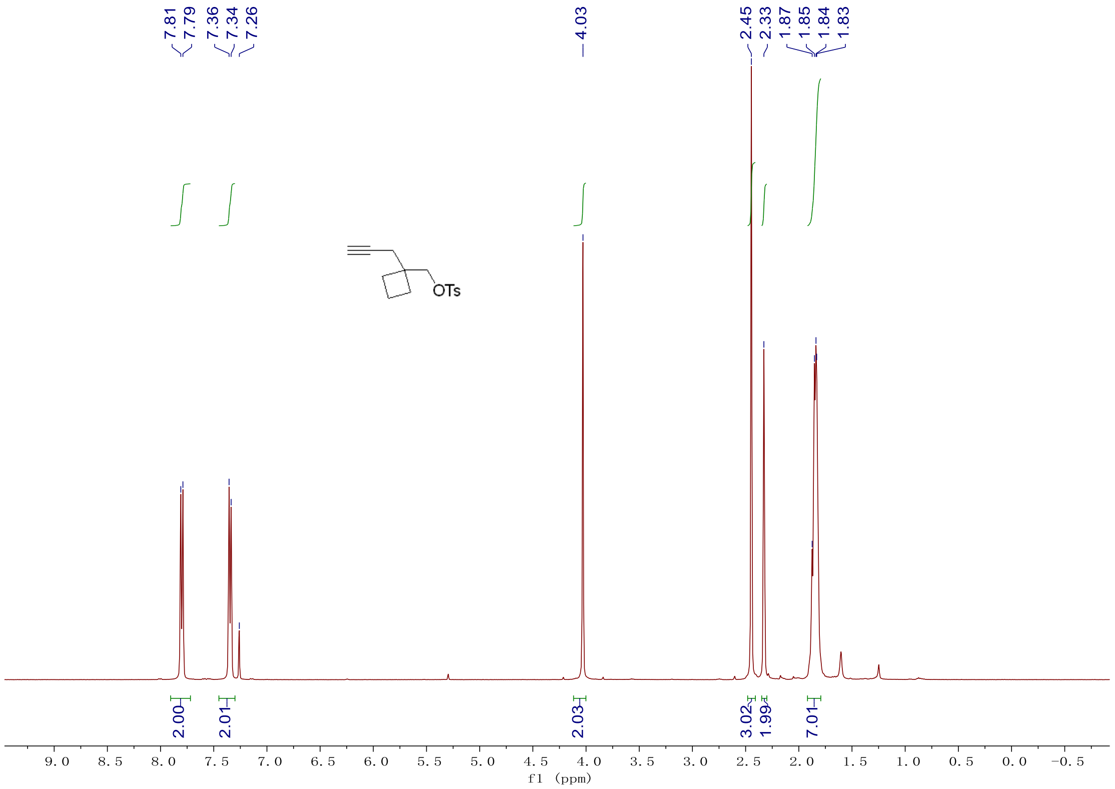

^{1}H NMR of compound 1ad

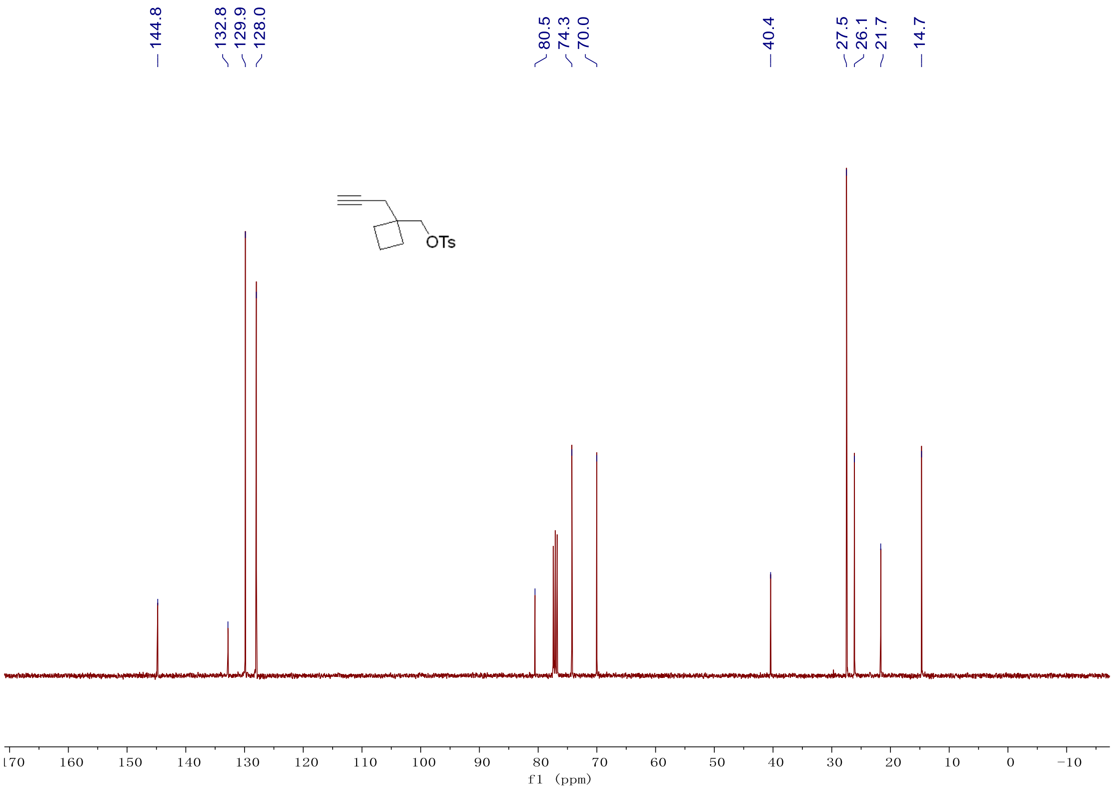

^{13}C NMR of compound 1ad

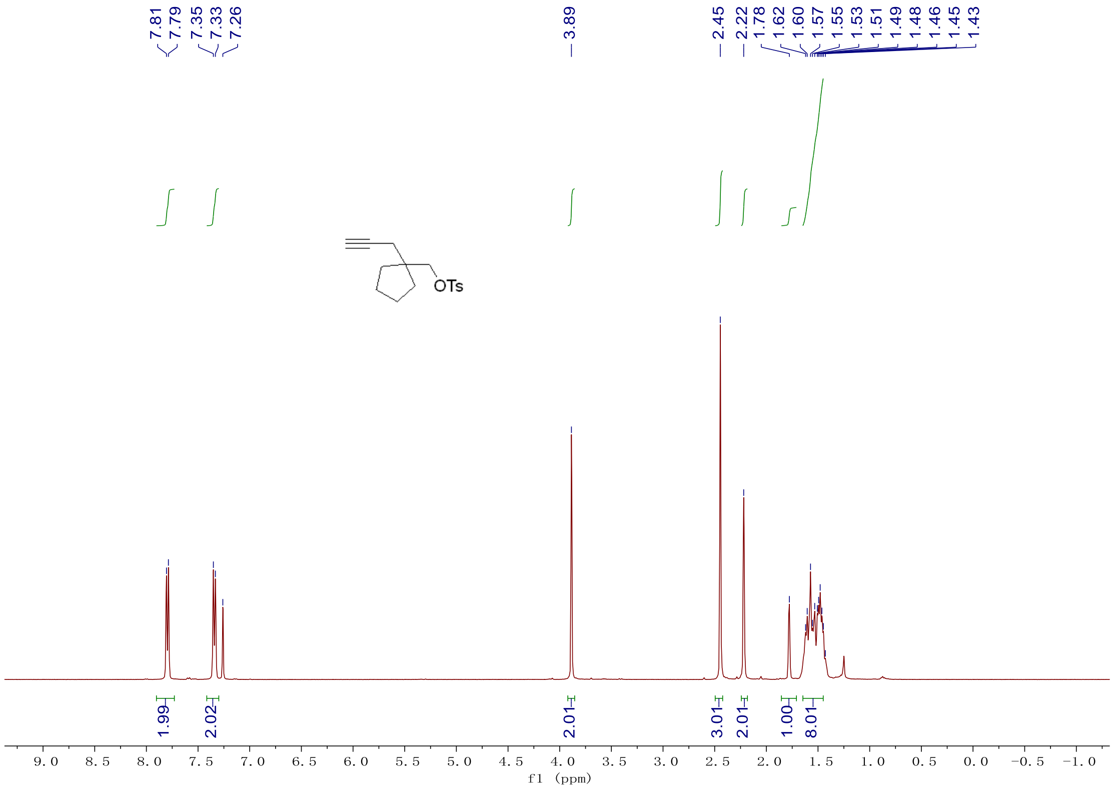

^{1}H NMR of compound 1ae

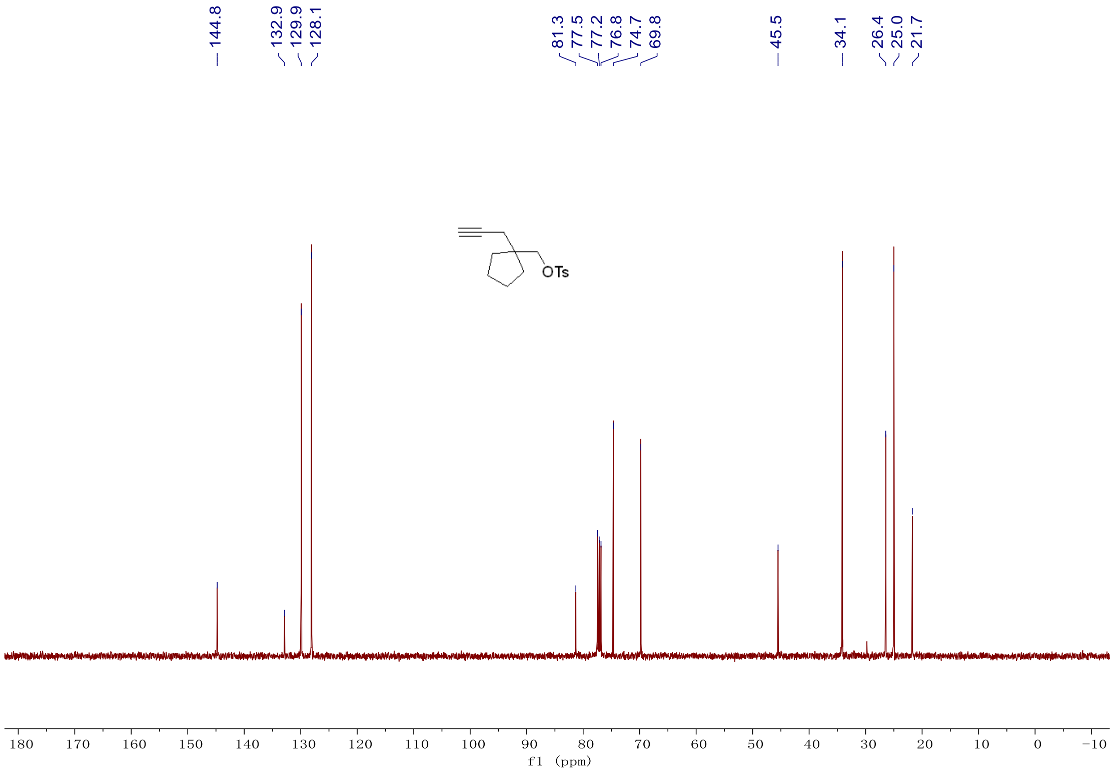

^{13}C NMR of compound 1ae

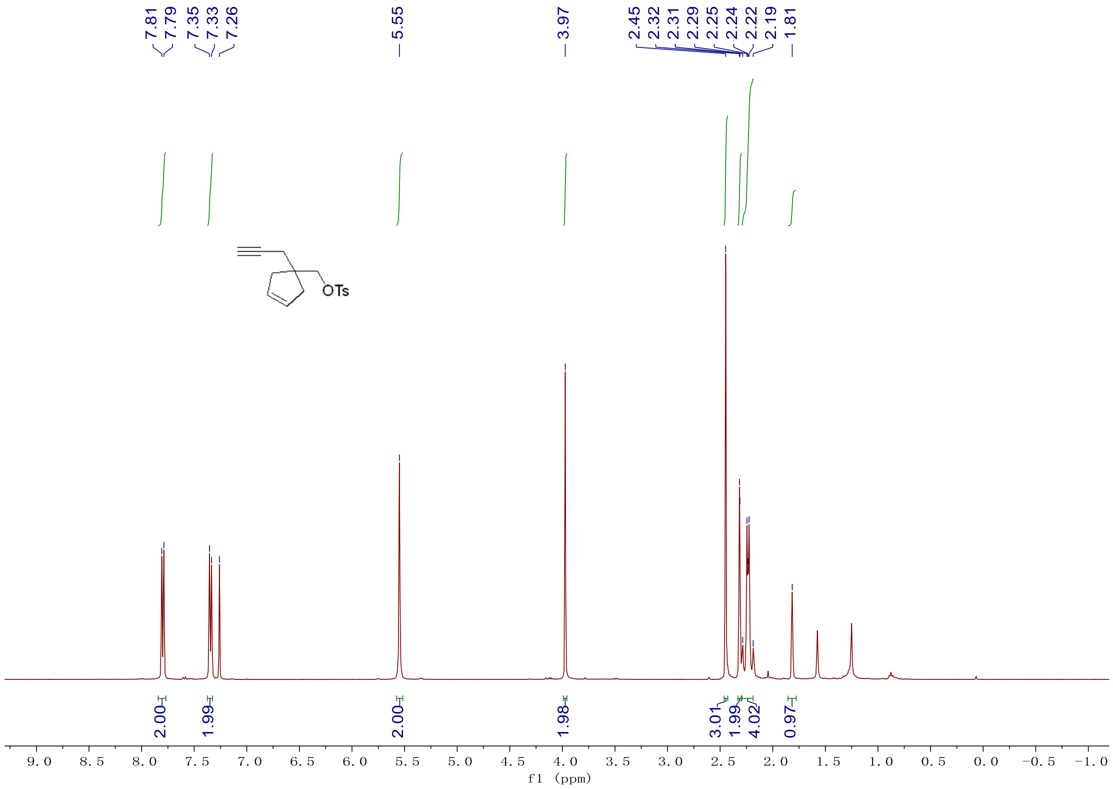

^{1}H NMR of compound 1af

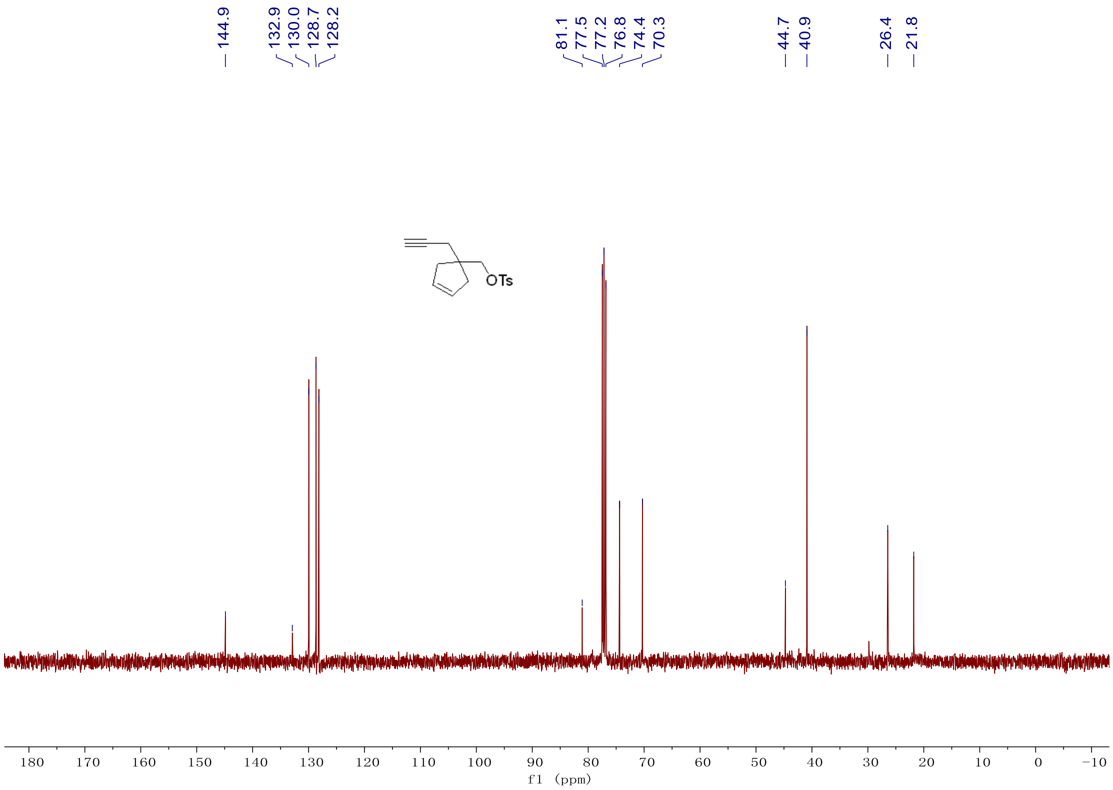

^{13}C NMR of compound 1af

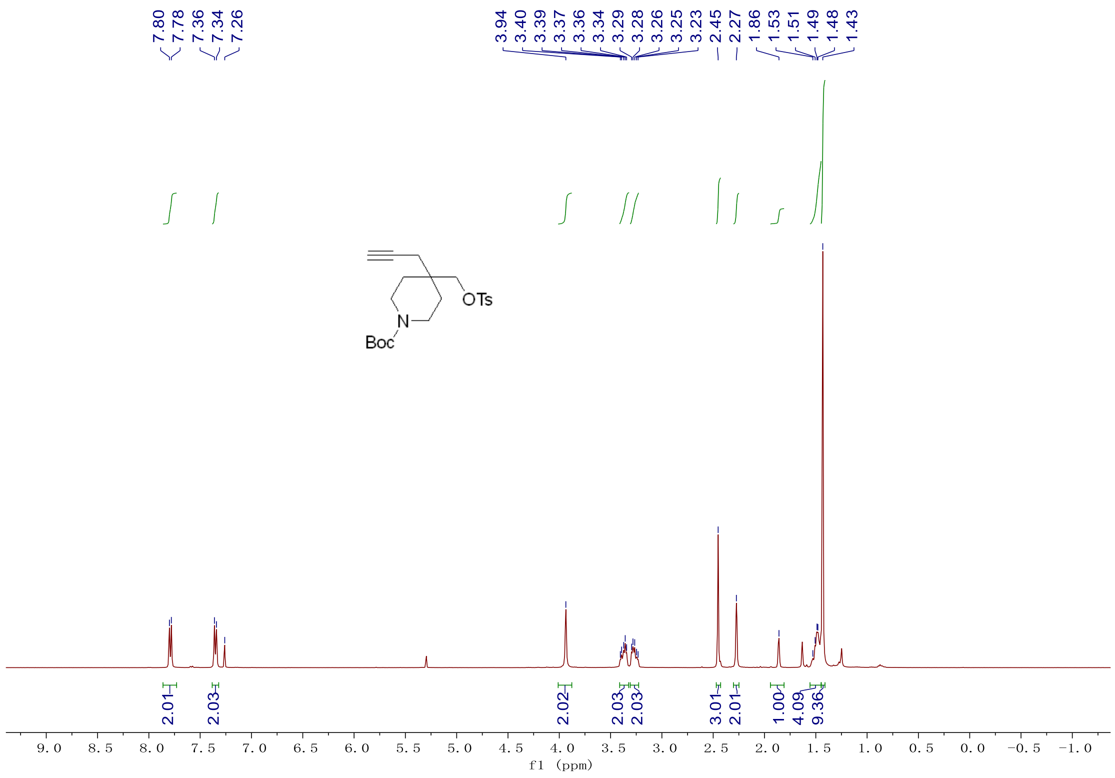

^{1}H NMR of compound 1ah

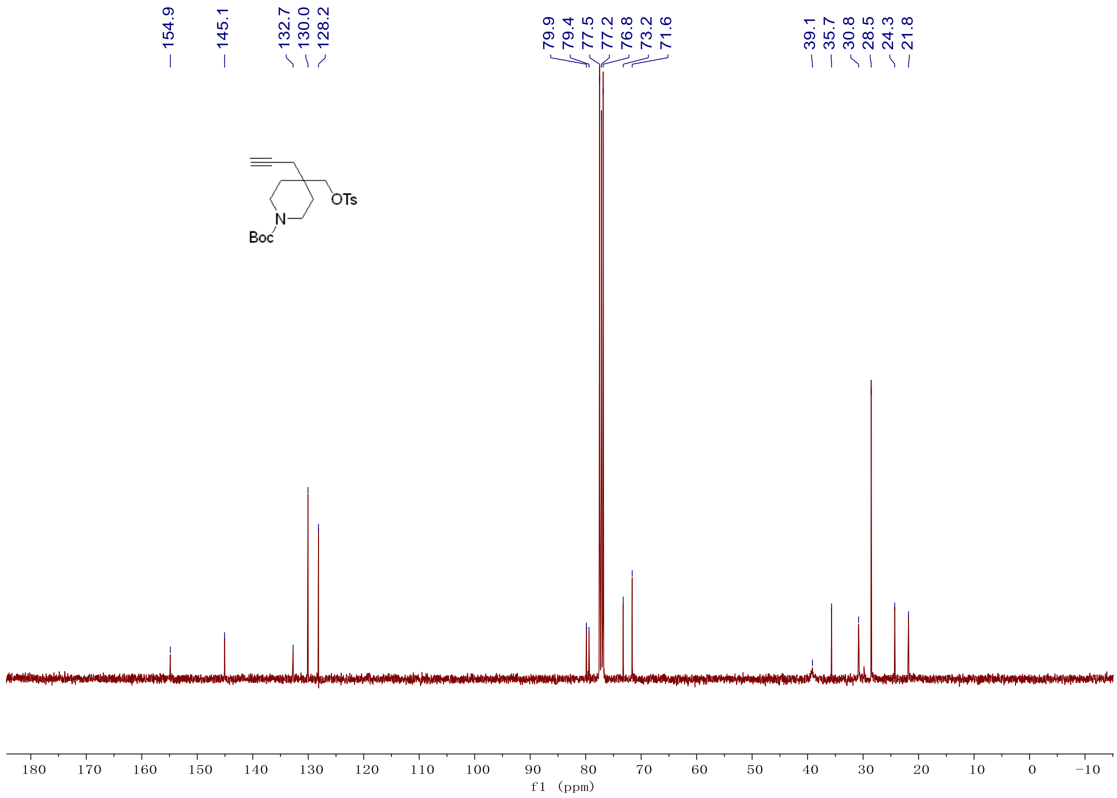

^{13}C NMR of compound 1ah

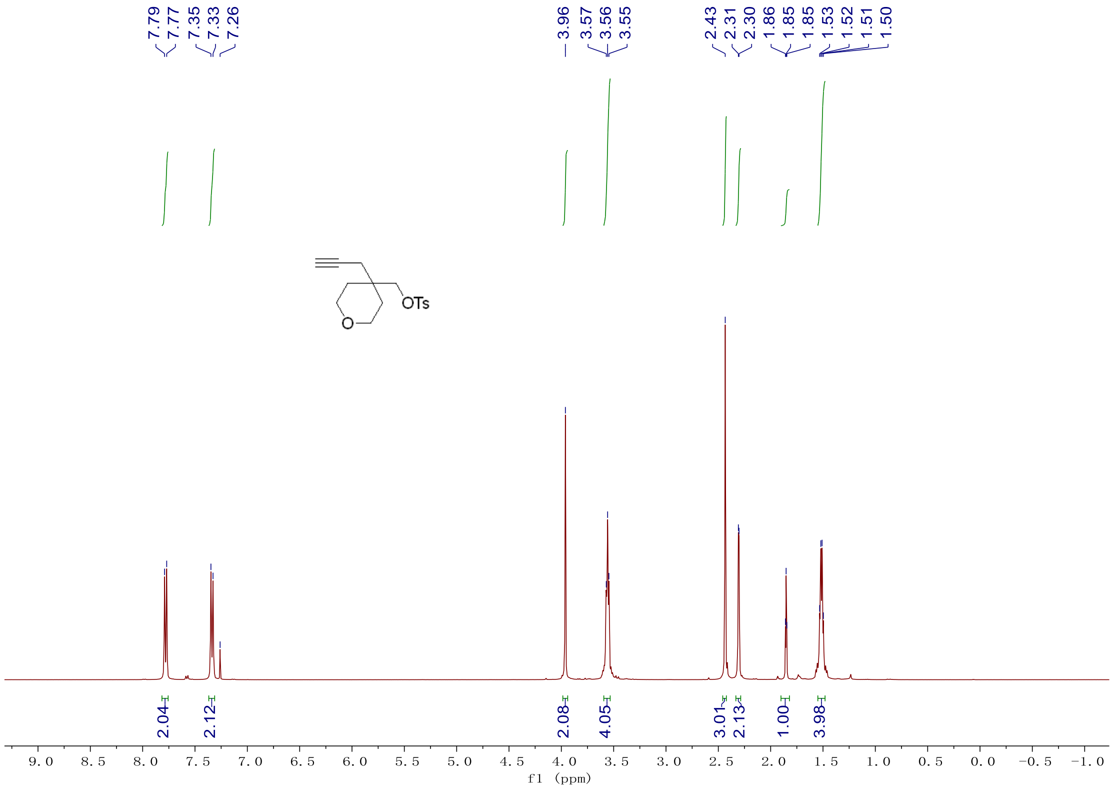

^{1}H NMR of compound 1ai

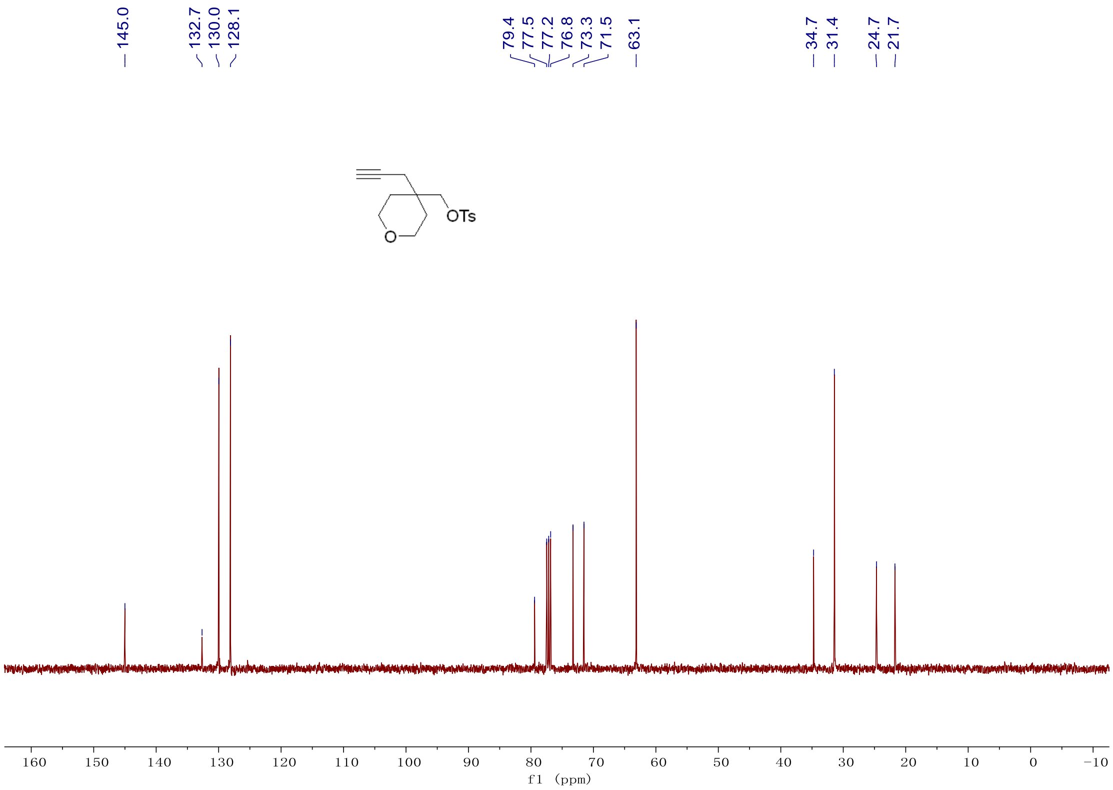

^{13}C NMR of compound 1ai

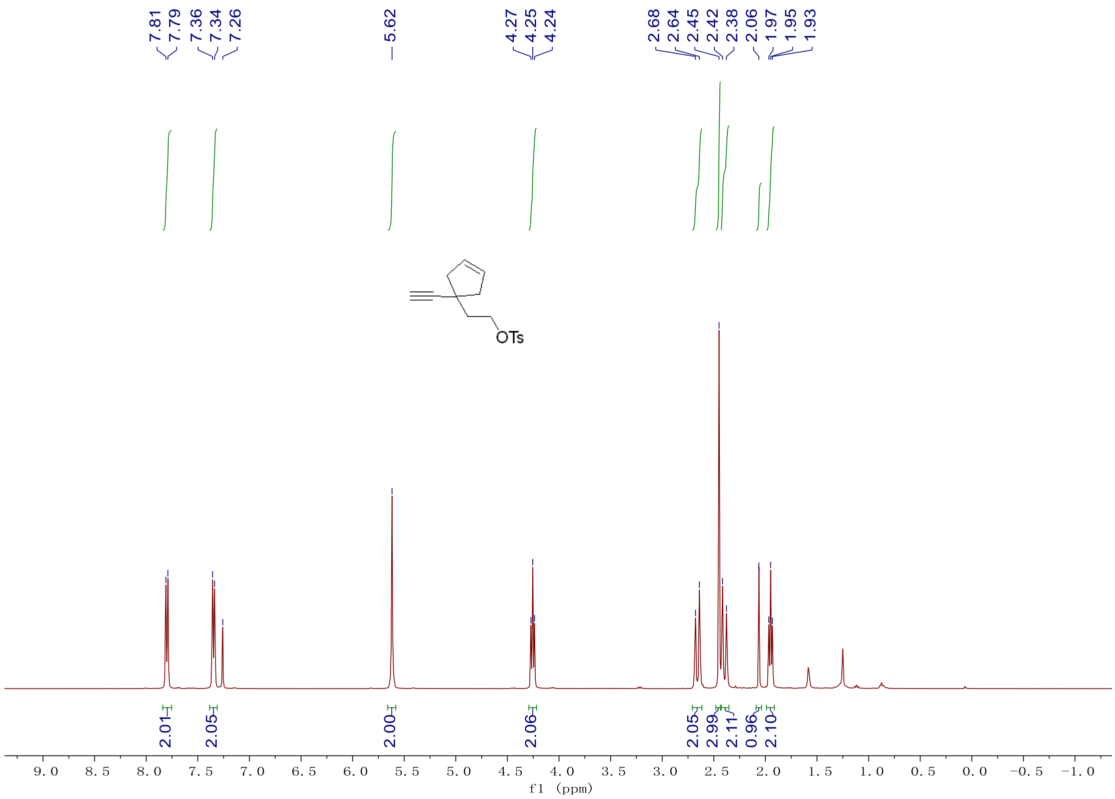

^{1}H NMR of compound 1aj

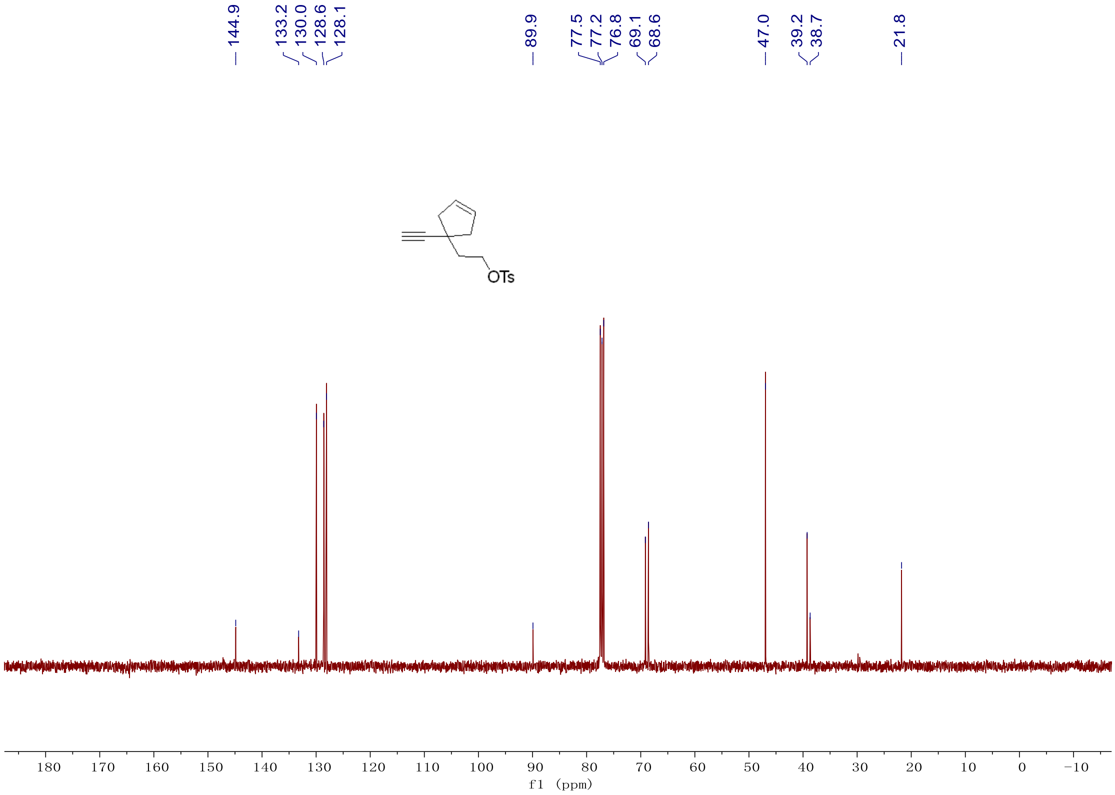

^{13}C NMR of compound 1aj

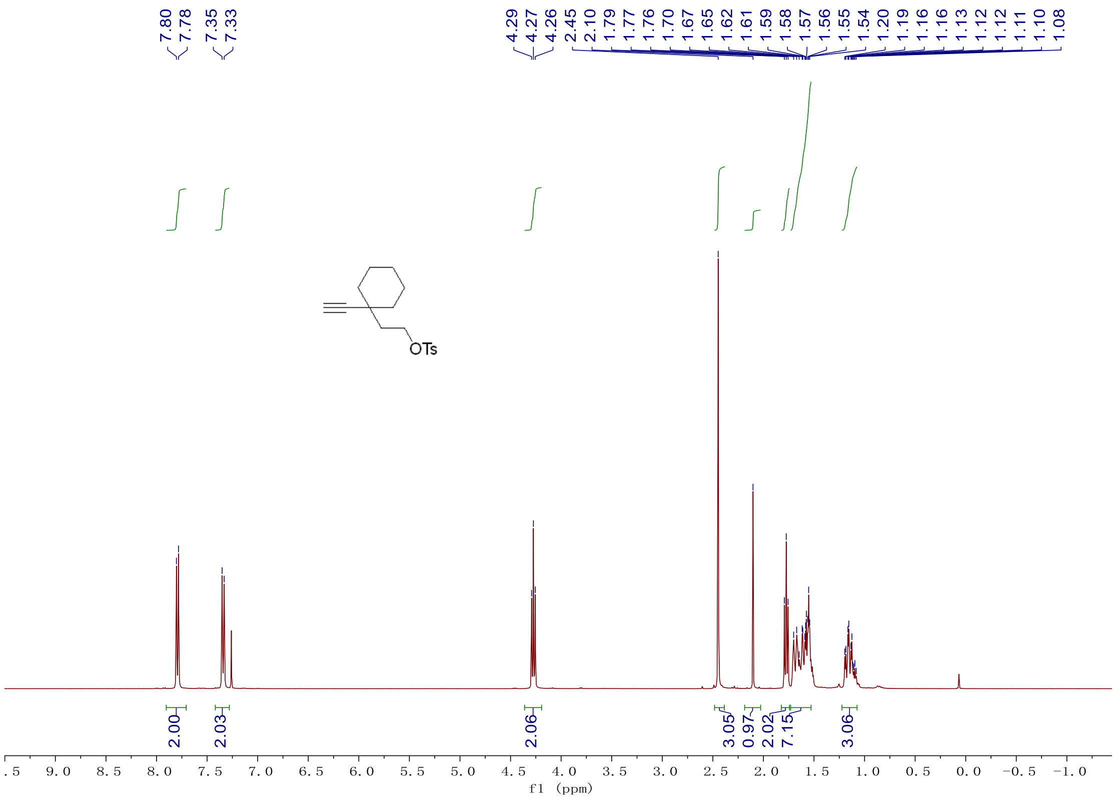

^{1}H NMR of compound 1ak

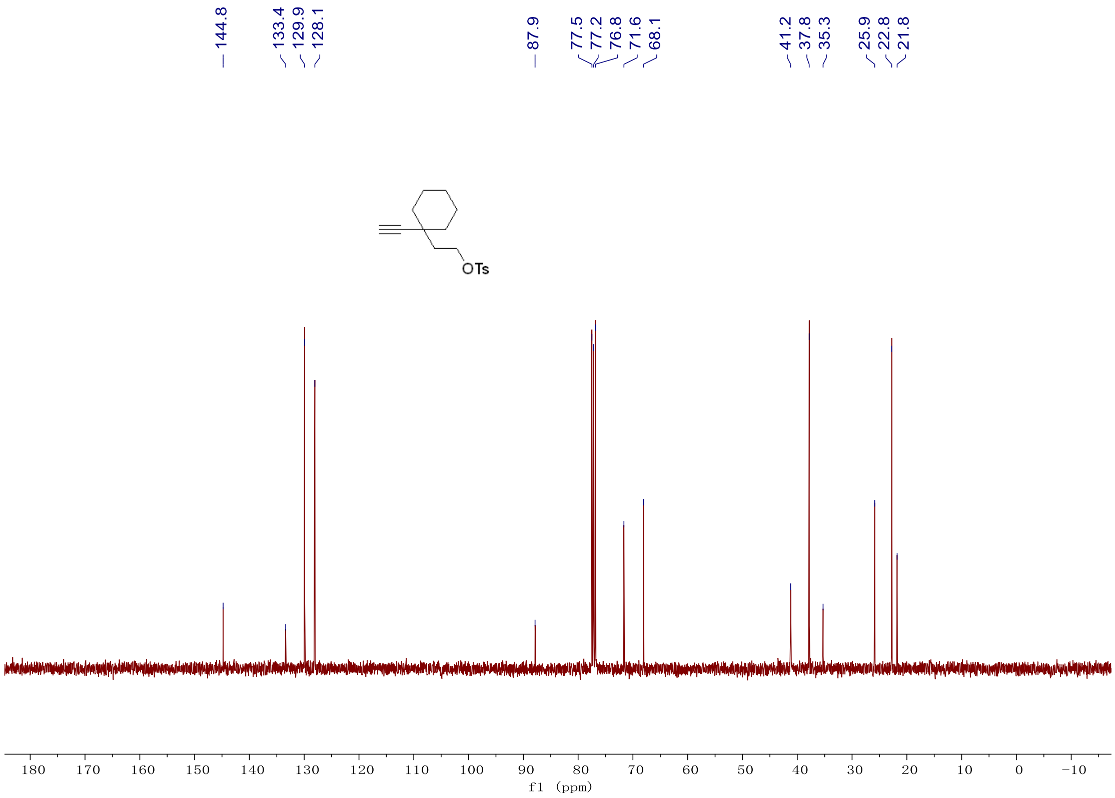

^{13}C NMR of compound 1ak

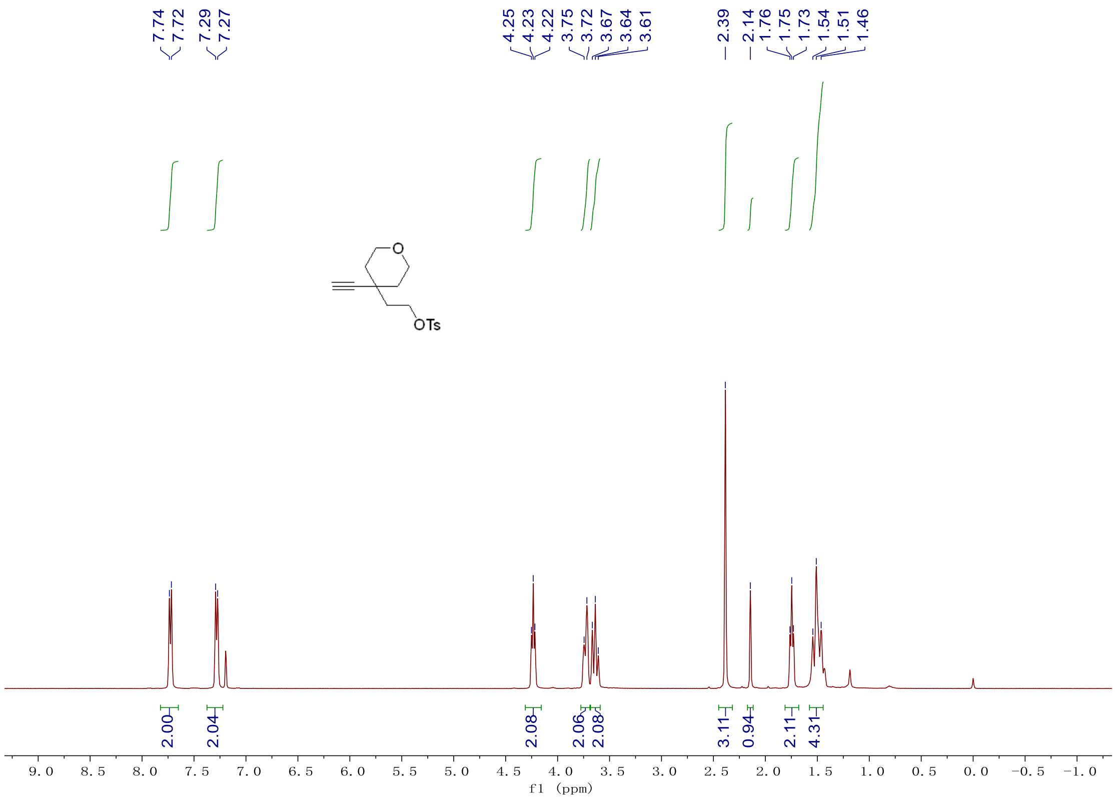

^{1}H NMR of compound 1al

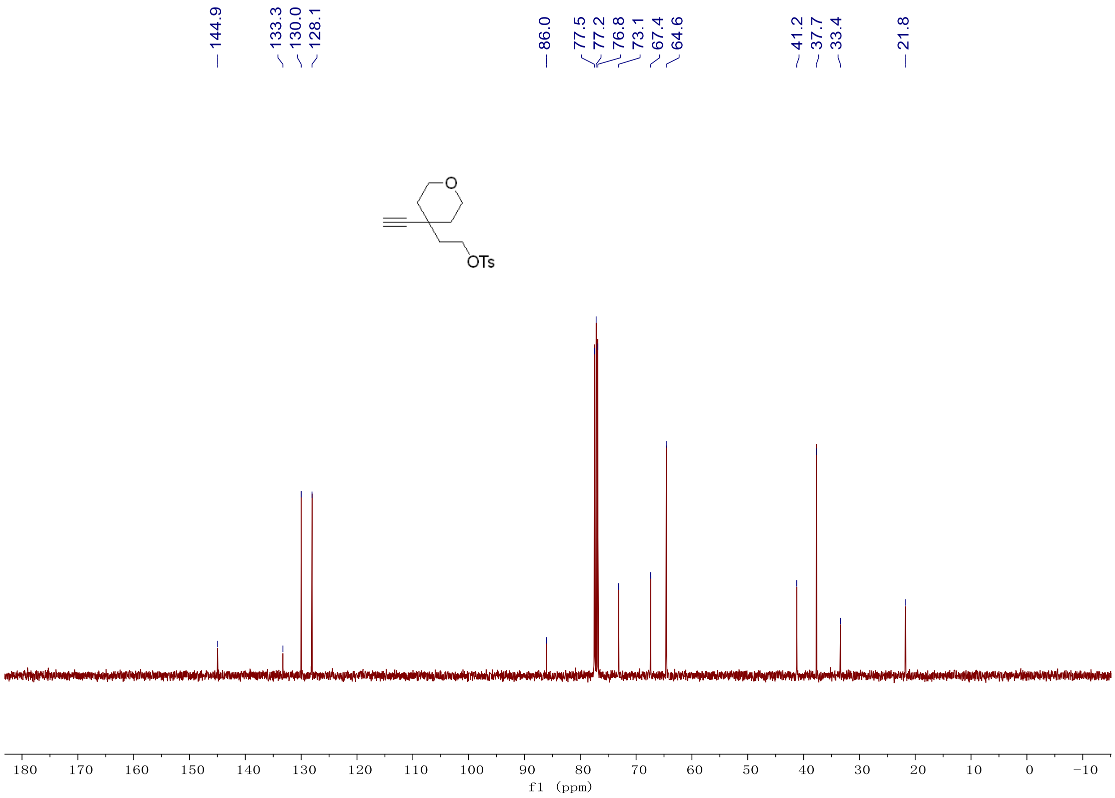

^{13}C NMR of compound 1al

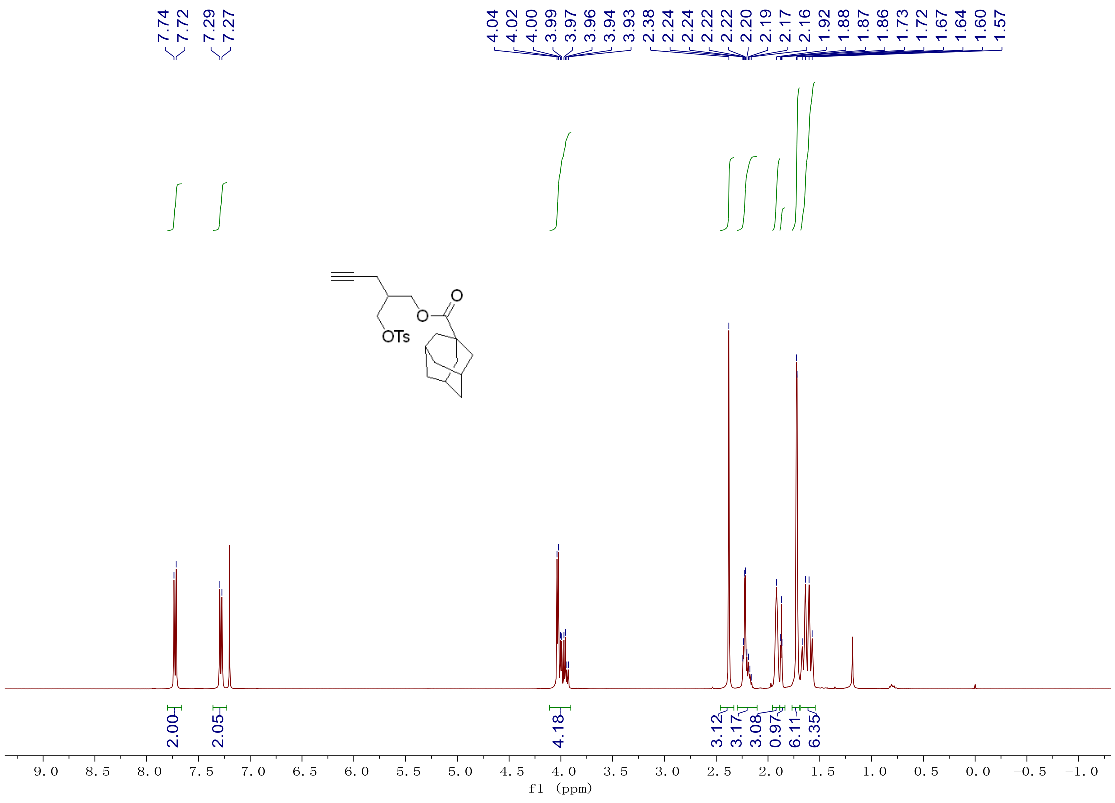

^{1}H NMR of compound 1am

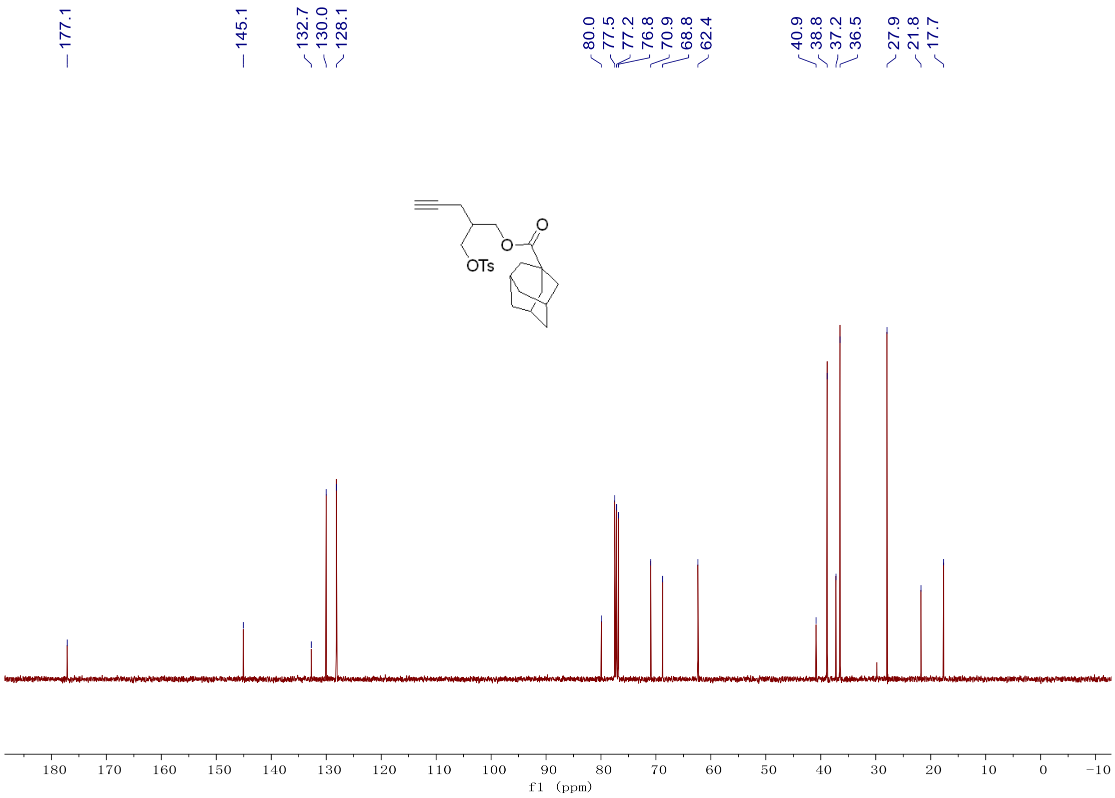

^{13}C NMR of compound 1am

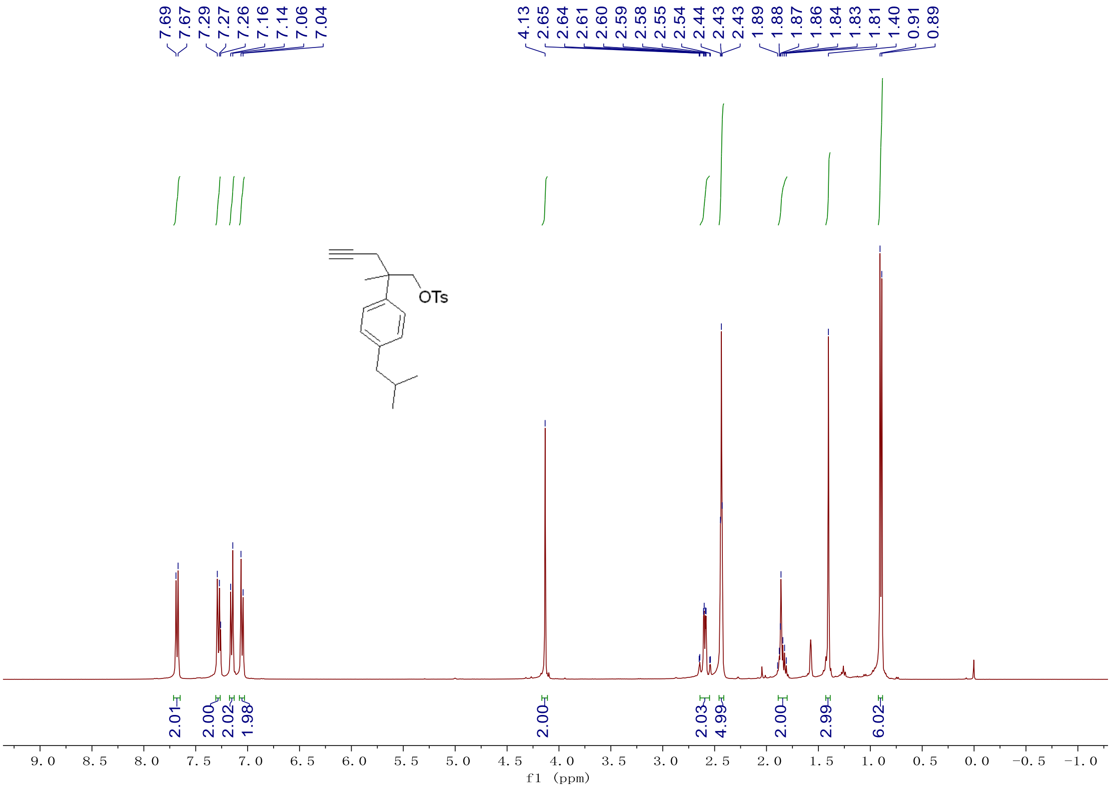

^{1}H NMR of compound 1an

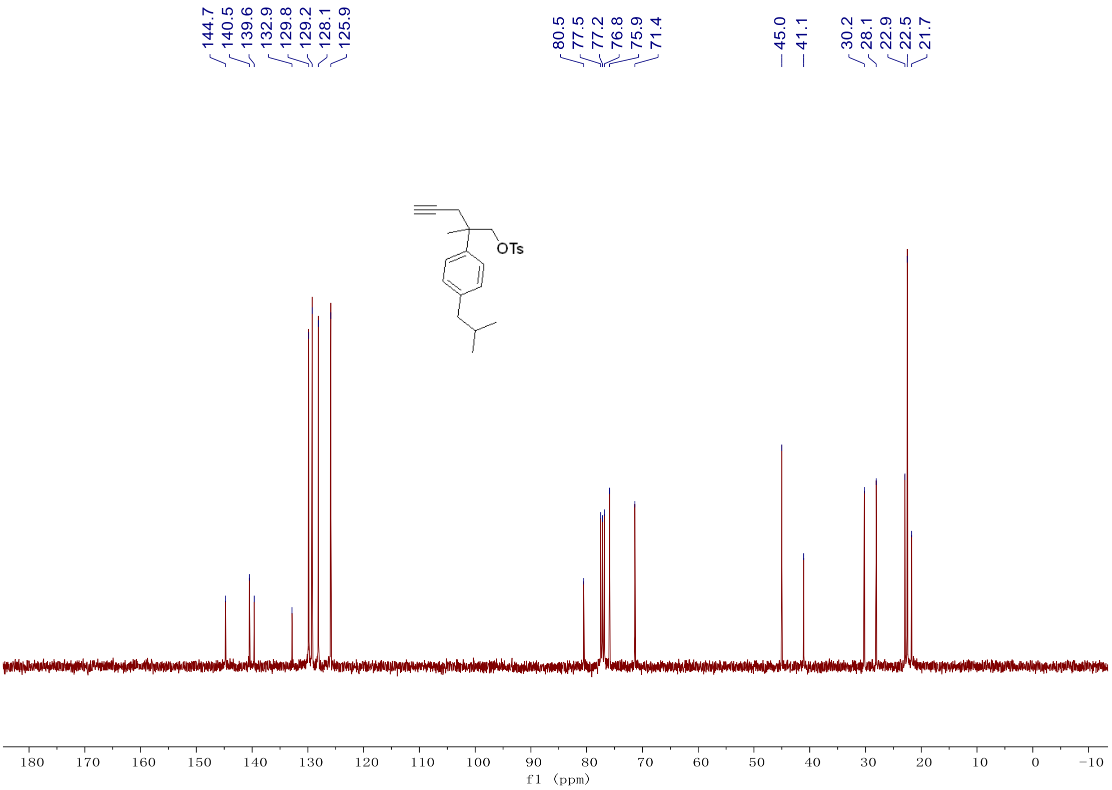

^{13}C NMR of compound 1an

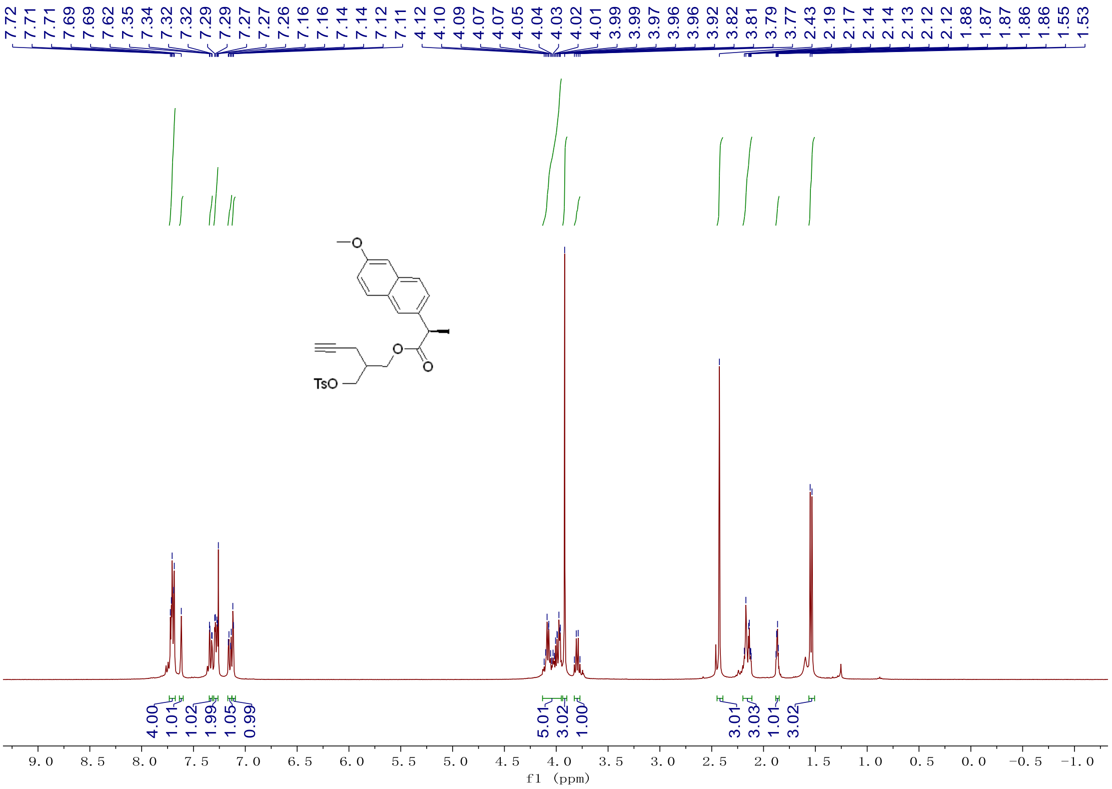

^{1}H NMR of compound 1ao

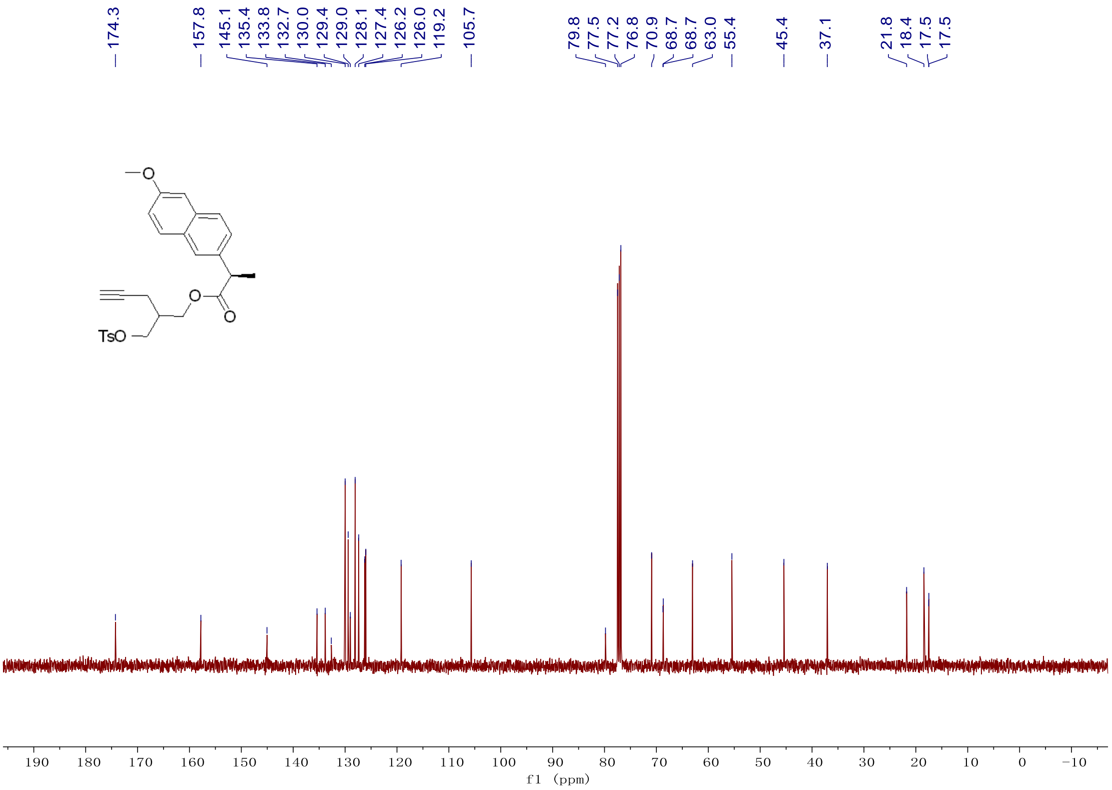

^{13}C NMR of compound 1ao

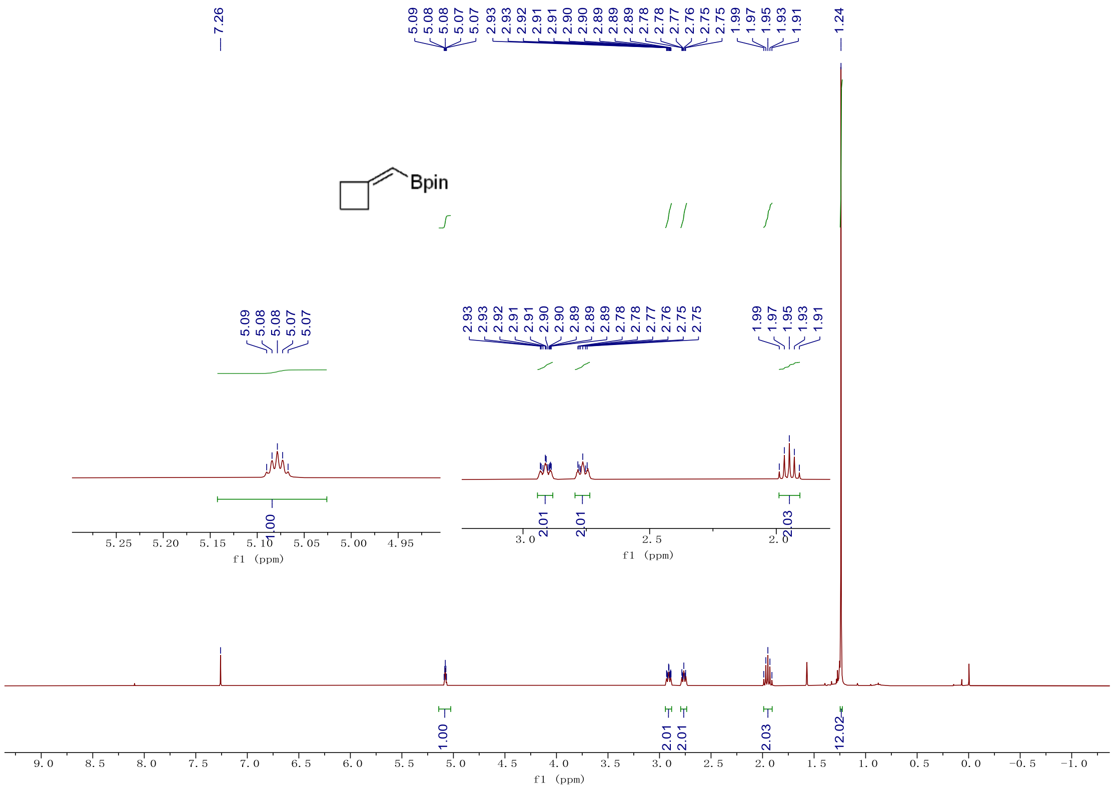

^{1}H NMR of compound 2a

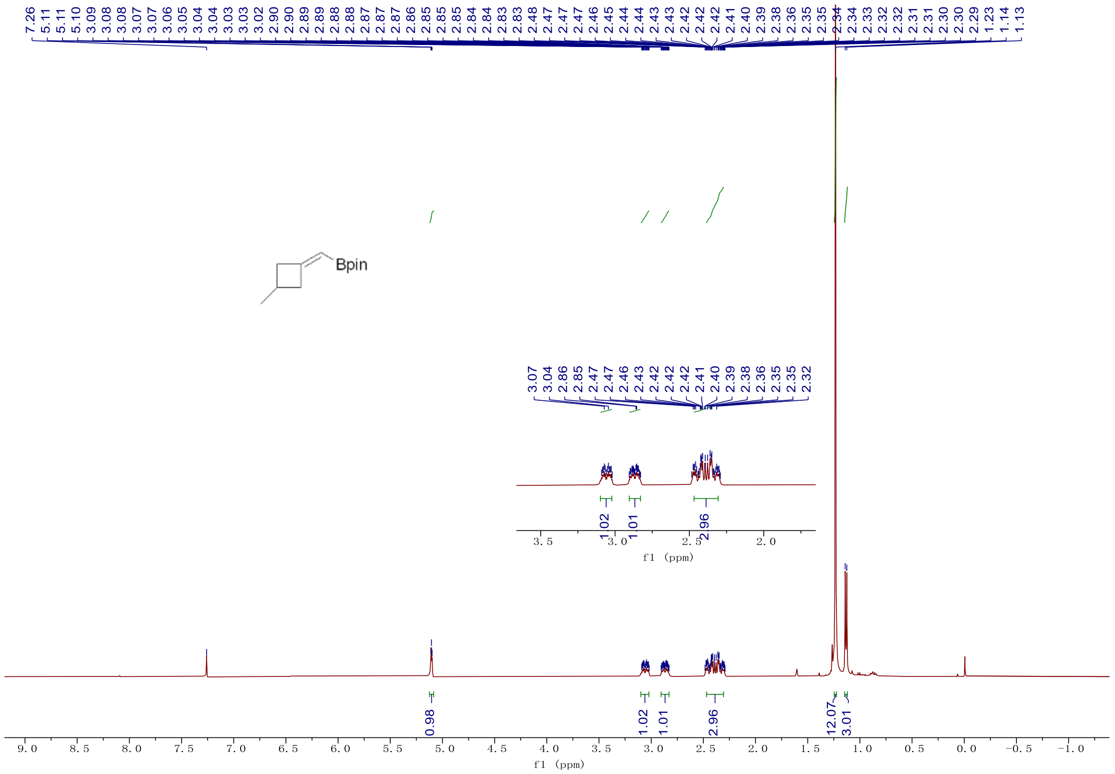

^{1}H NMR of compound 2b

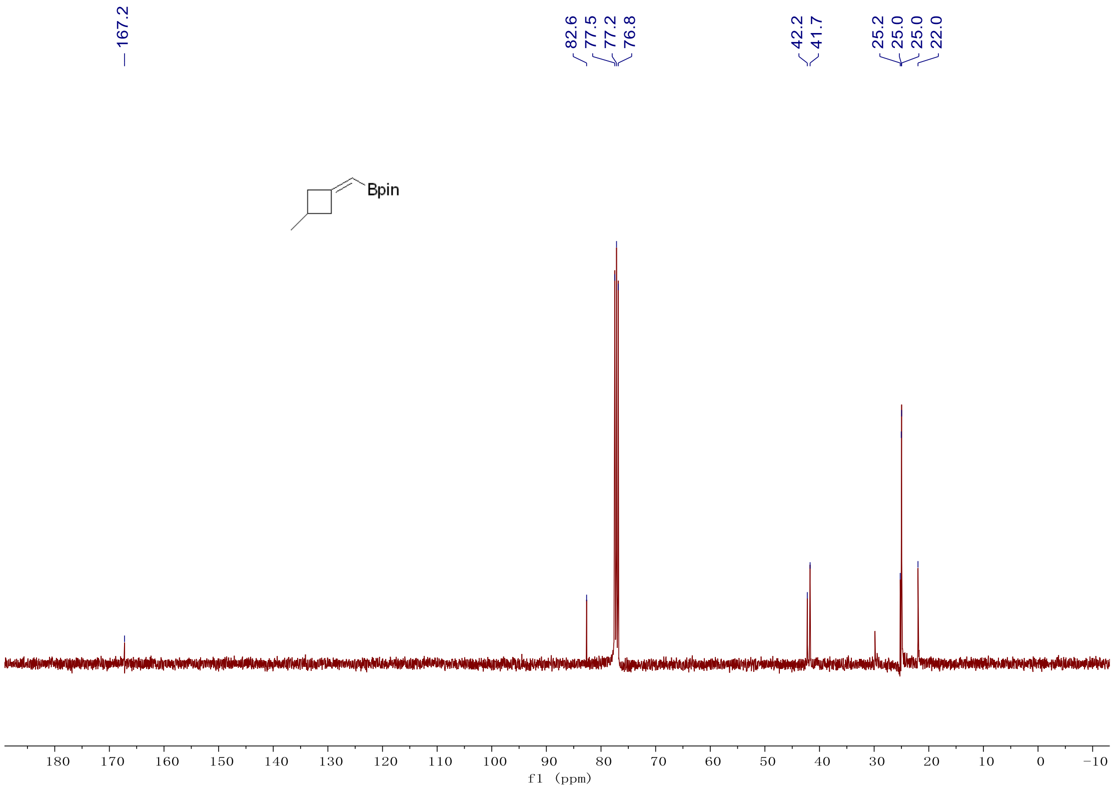

^{13}C NMR of compound 2b

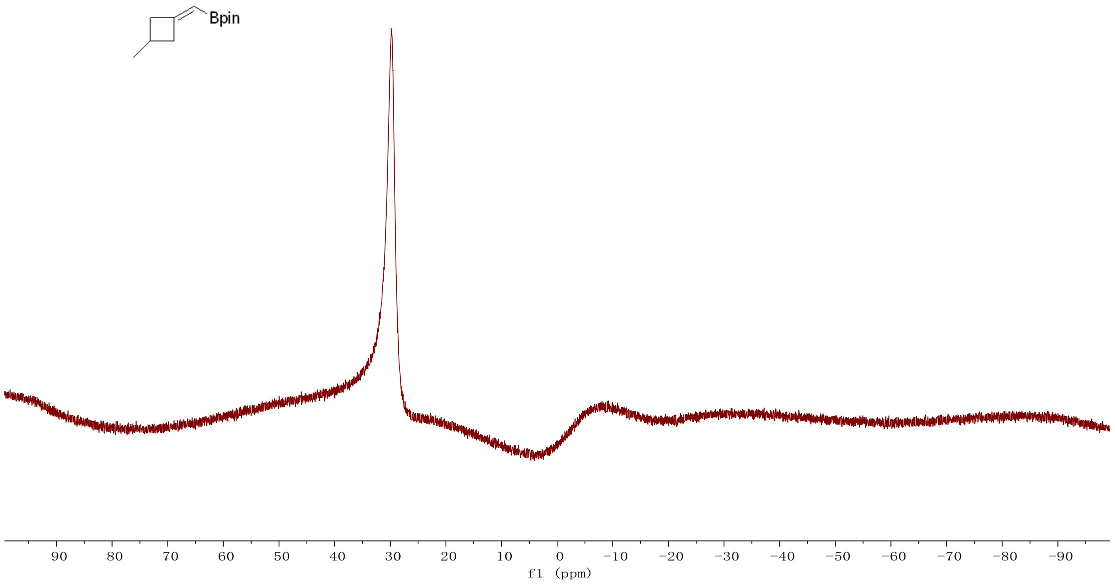

^{11}B NMR of compound 2b

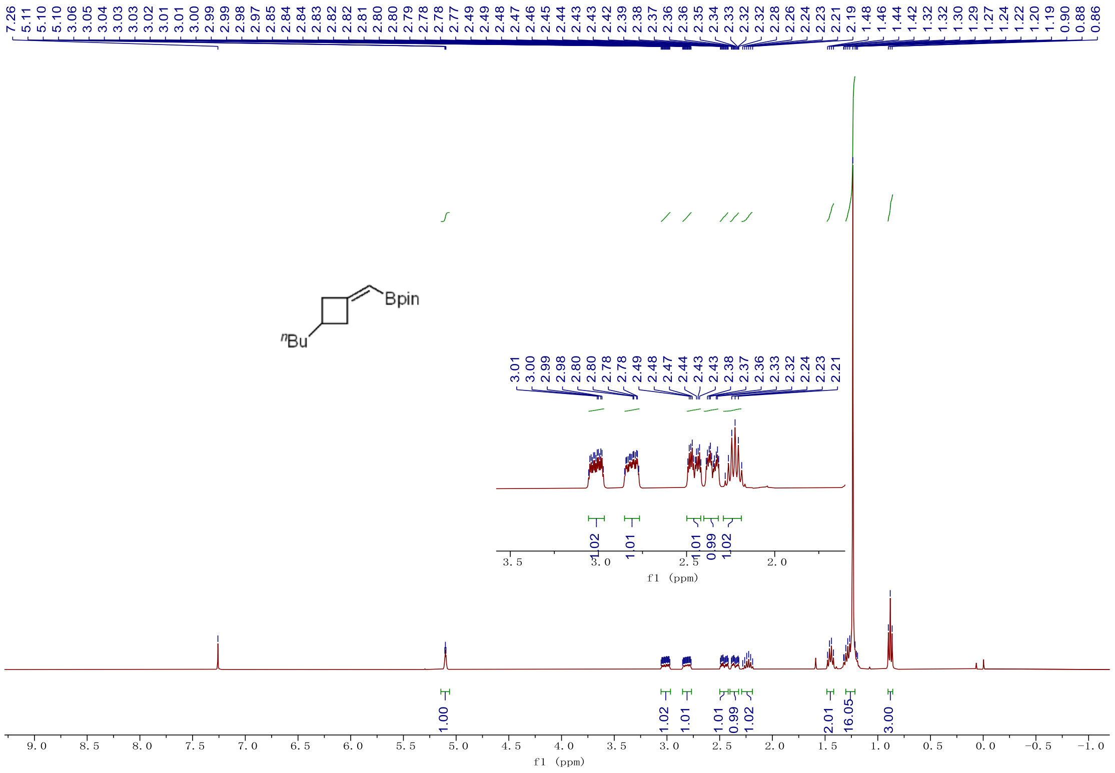

^{1}H NMR of compound 2c

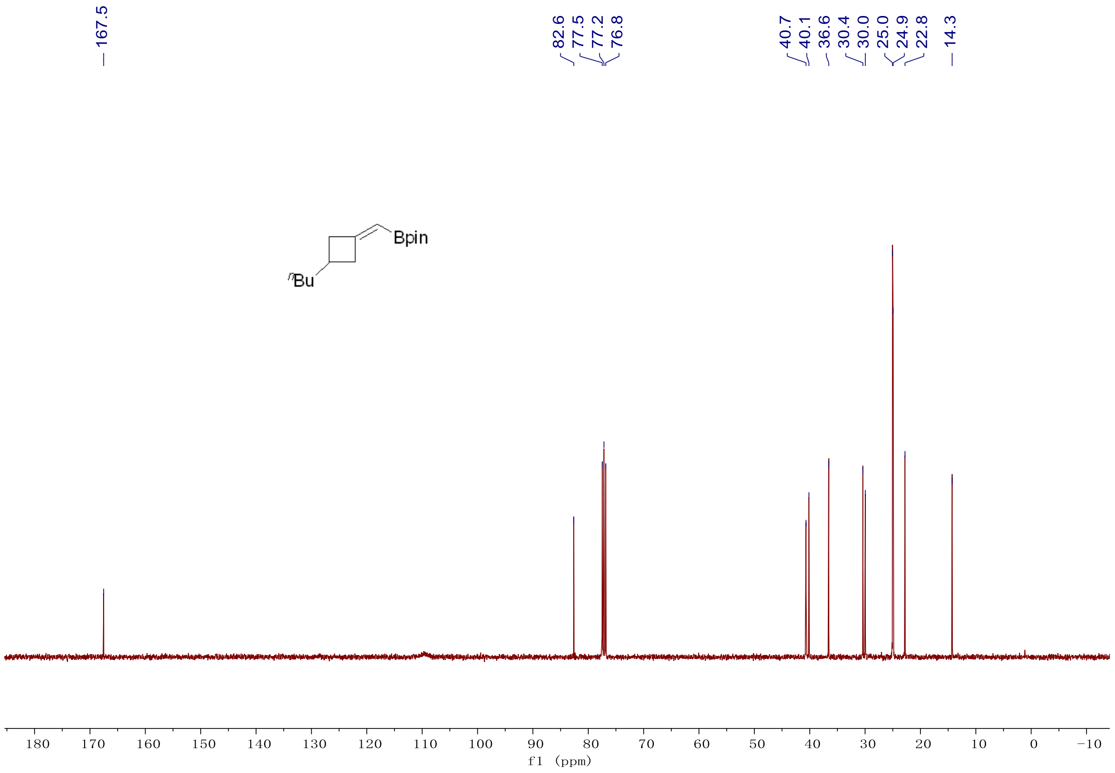

^{13}C NMR of compound 2c

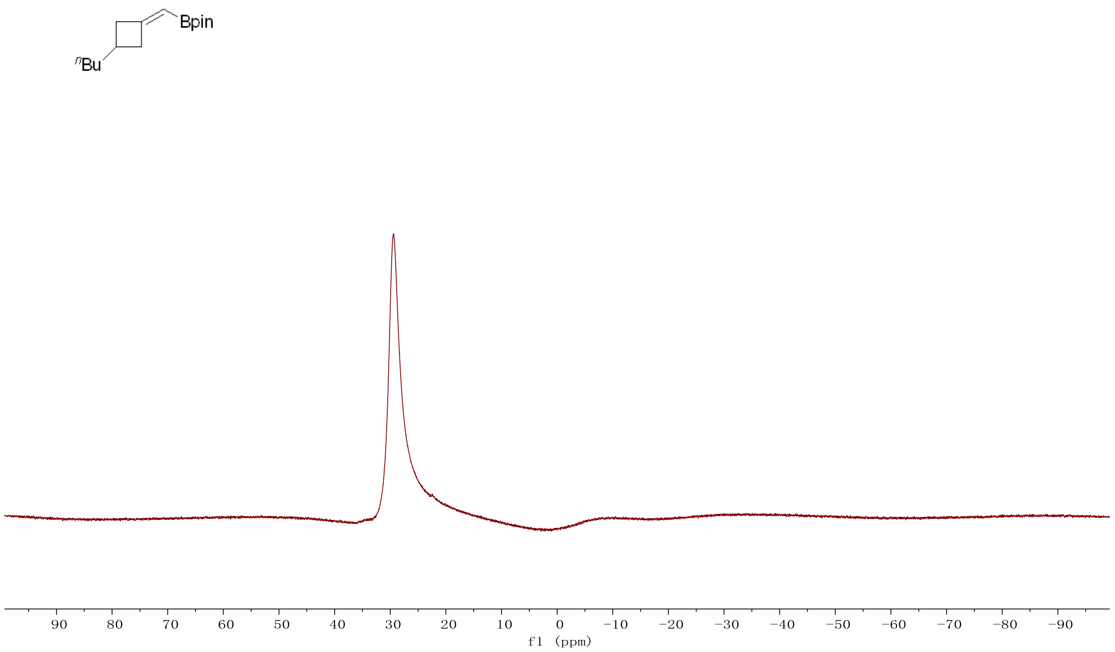

^{11}B NMR of compound 2c

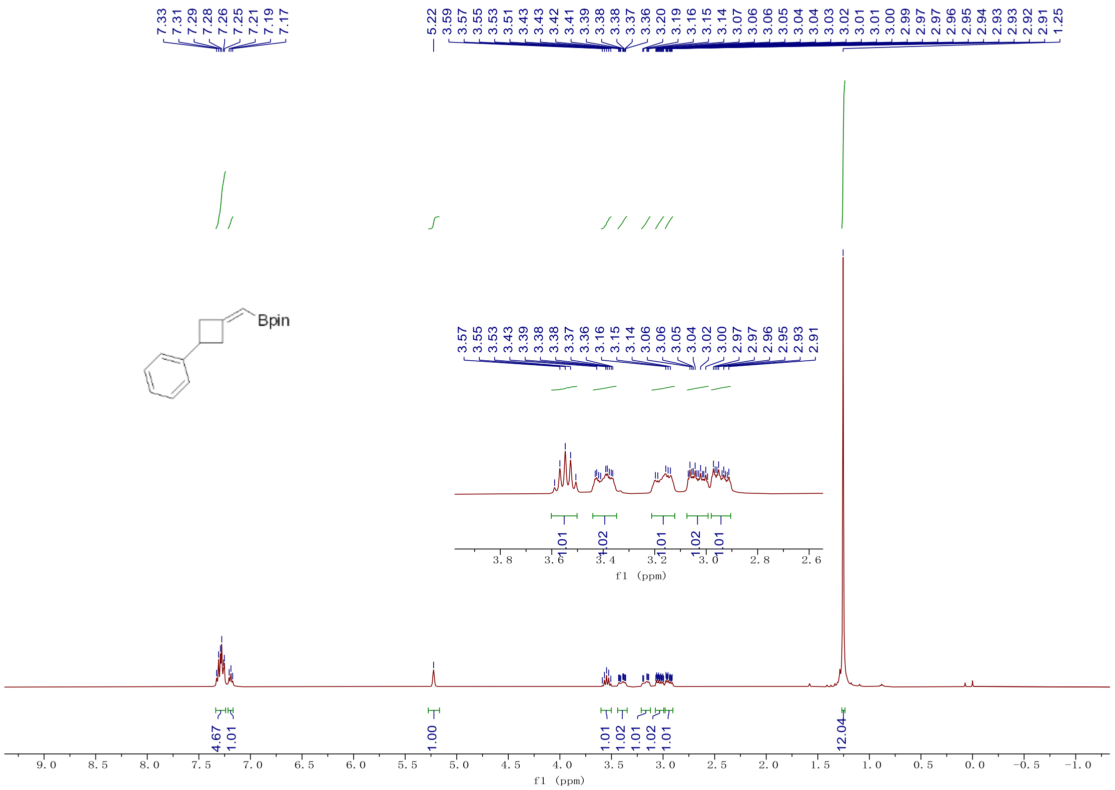

^{1}H NMR of compound 2d

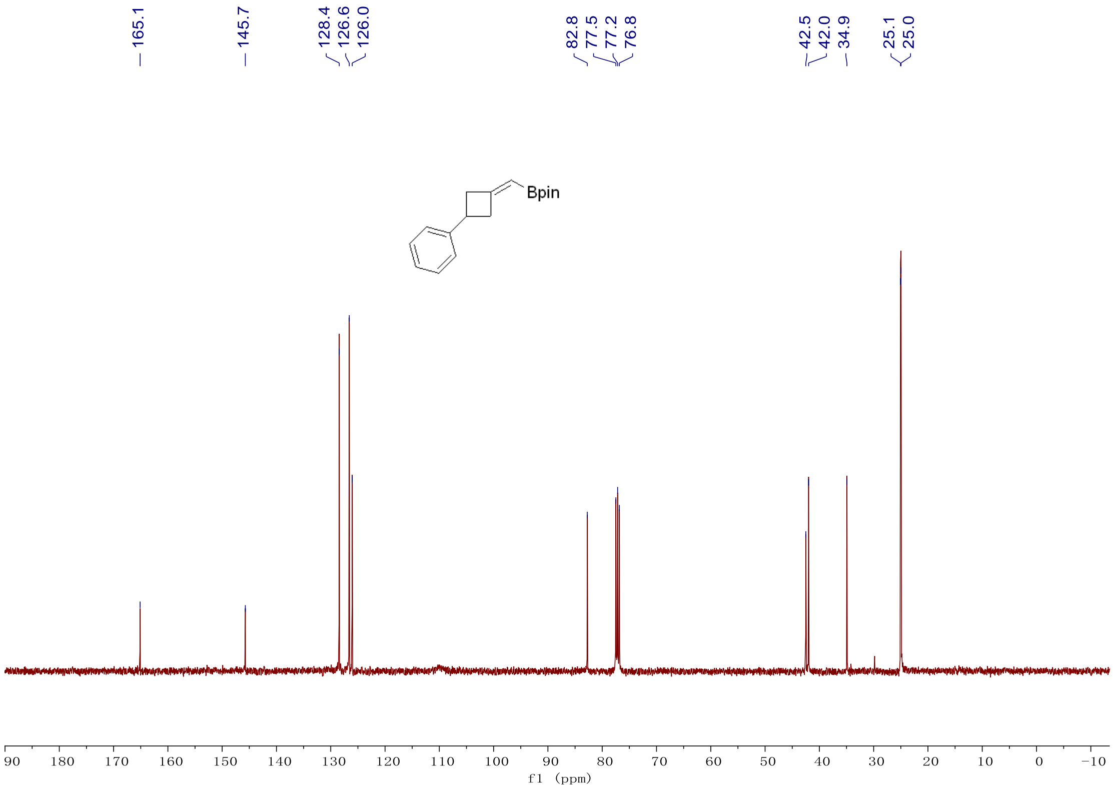

^{13}C NMR of compound 2d

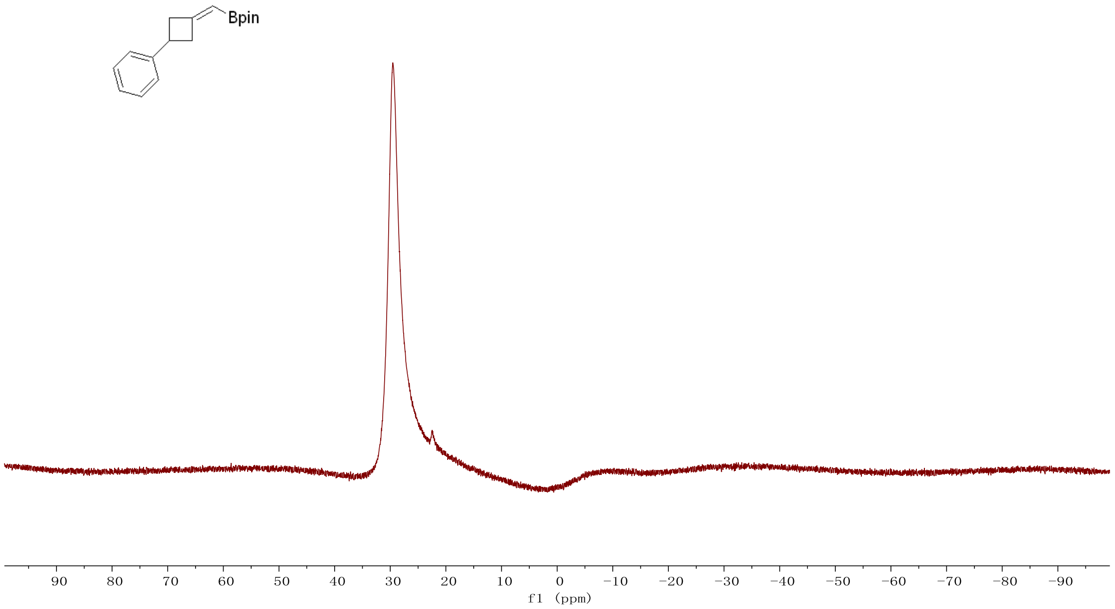

^{11}B NMR of compound 2d

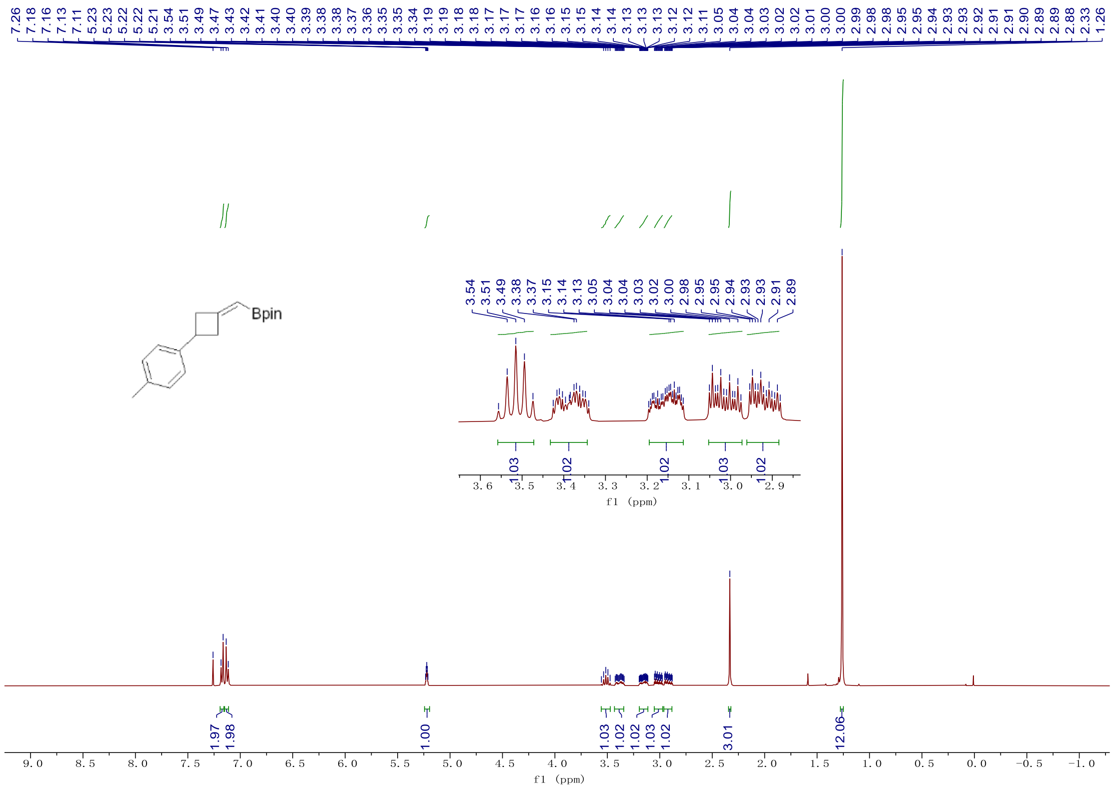

^{1}H NMR of compound 2e

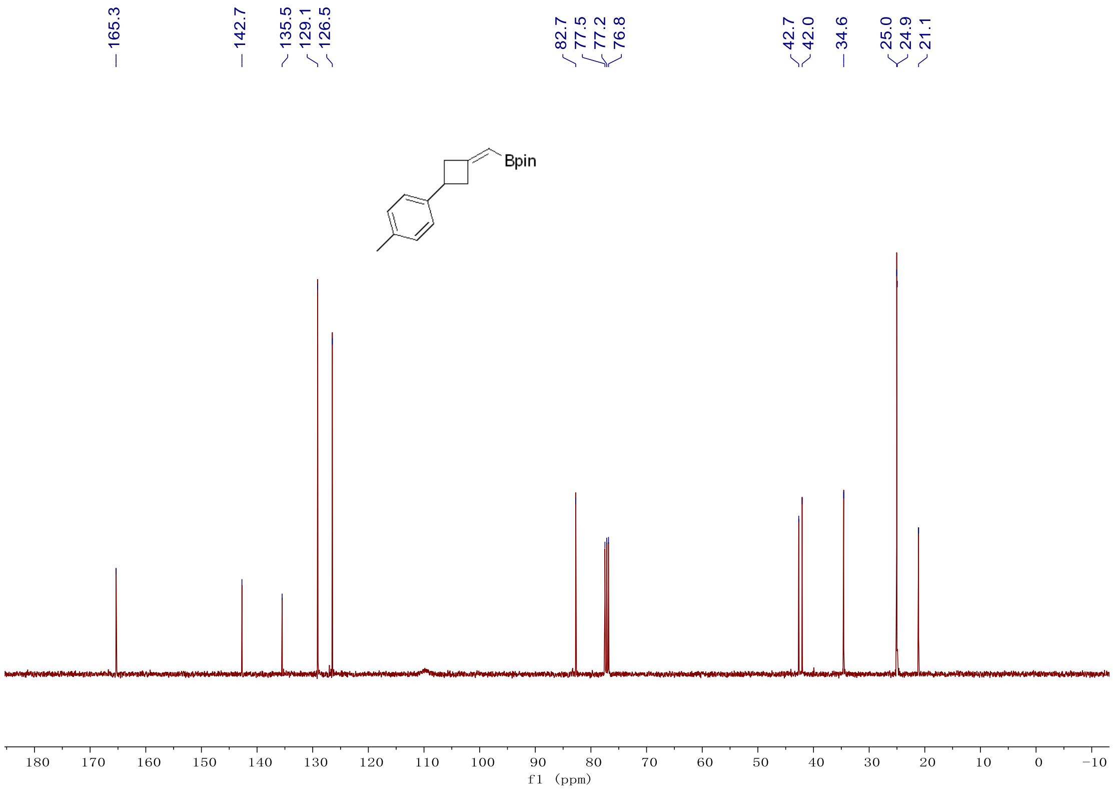

^{13}C NMR of compound 2e

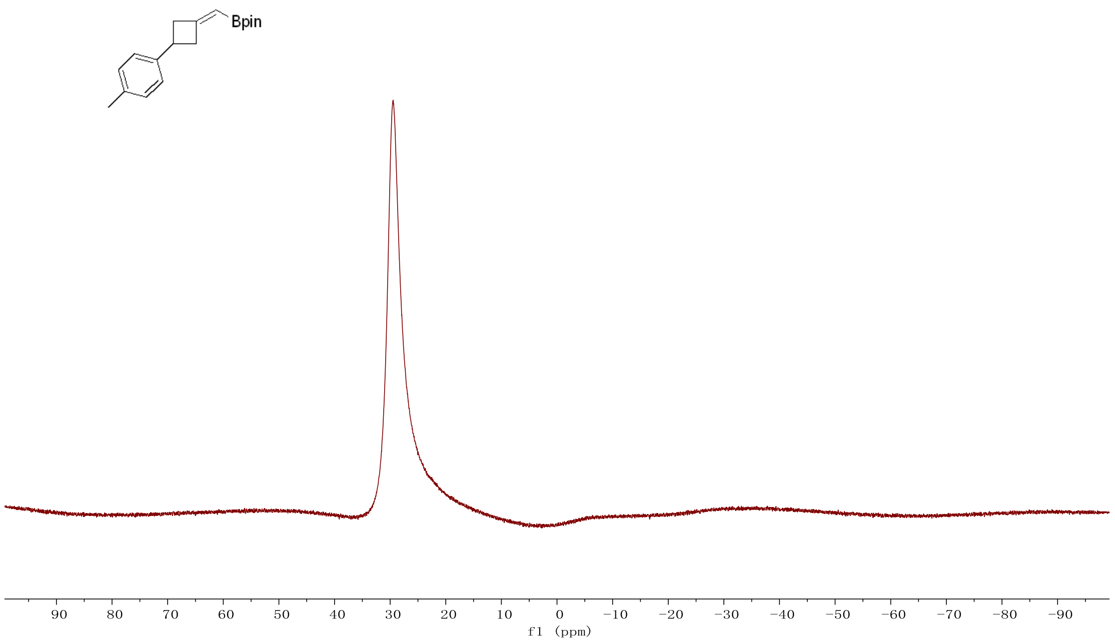

^{11}B NMR of compound 2e

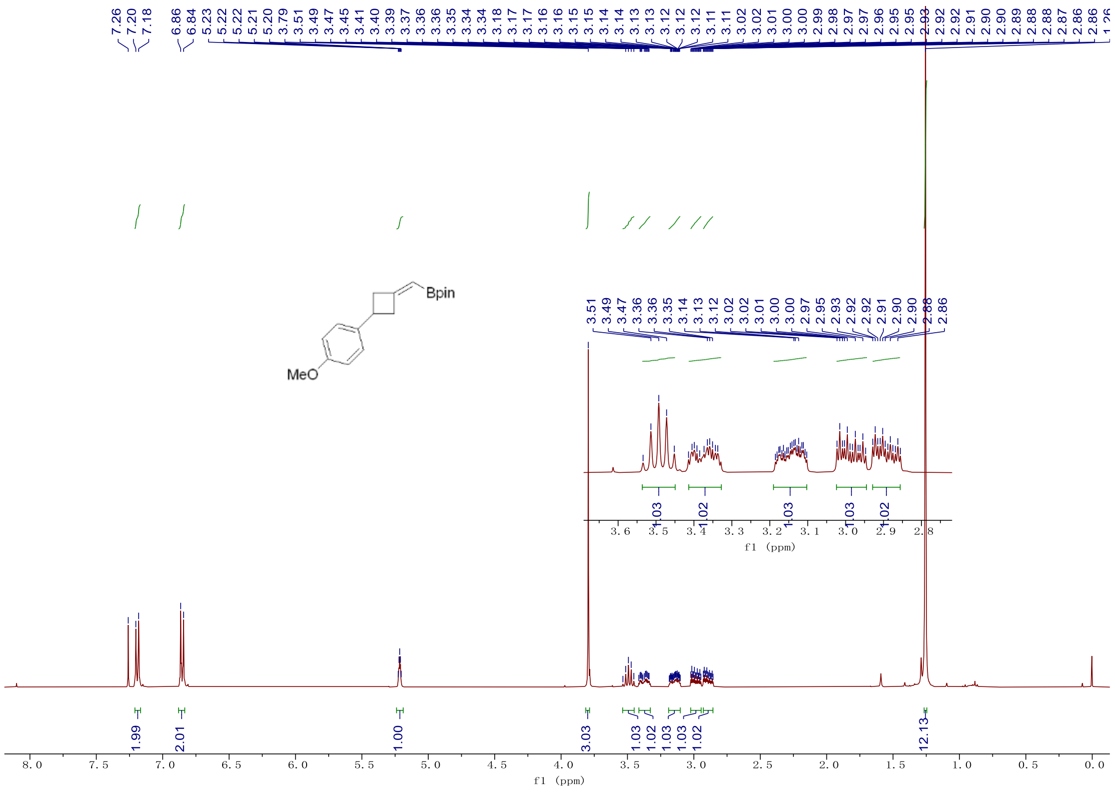

^{1}H NMR of compound 2f

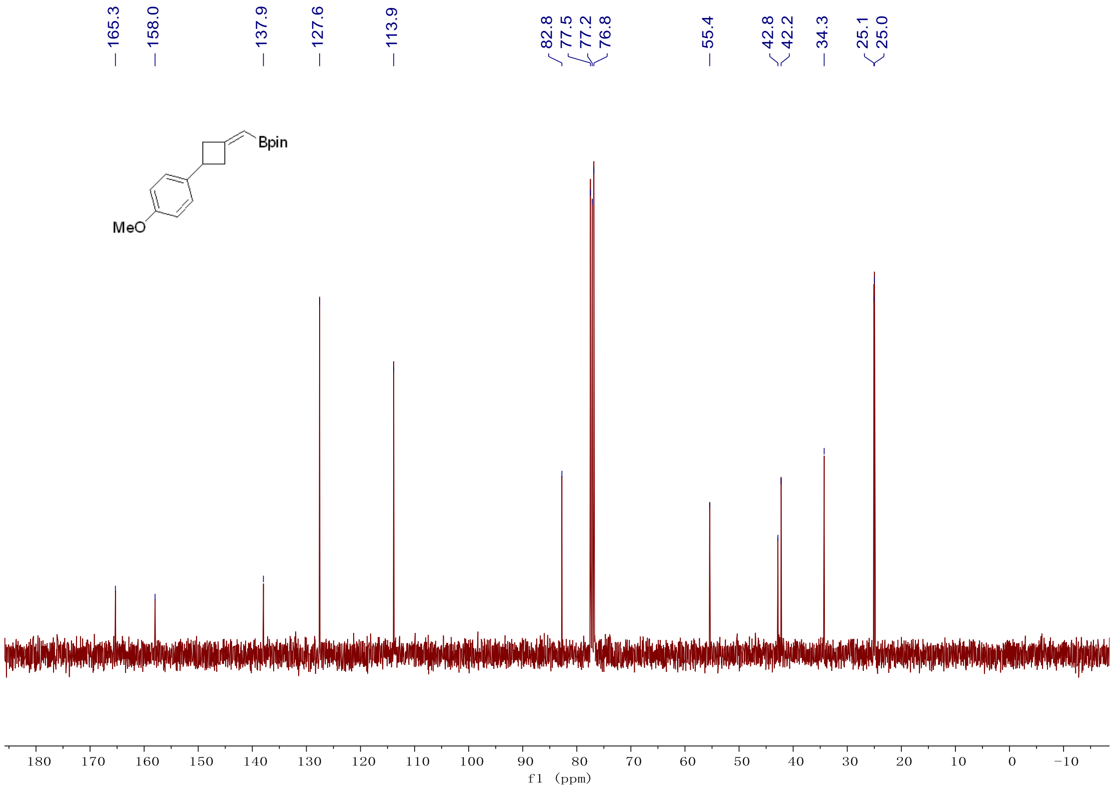

^{13}C NMR of compound 2f

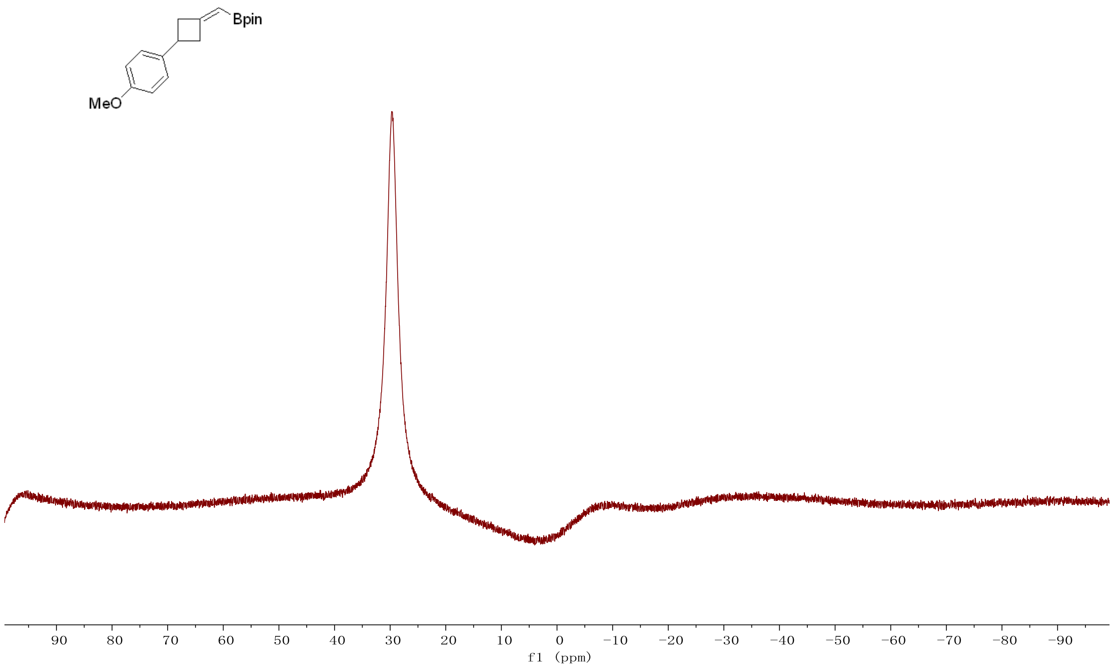

^{11}B NMR of compound 2f

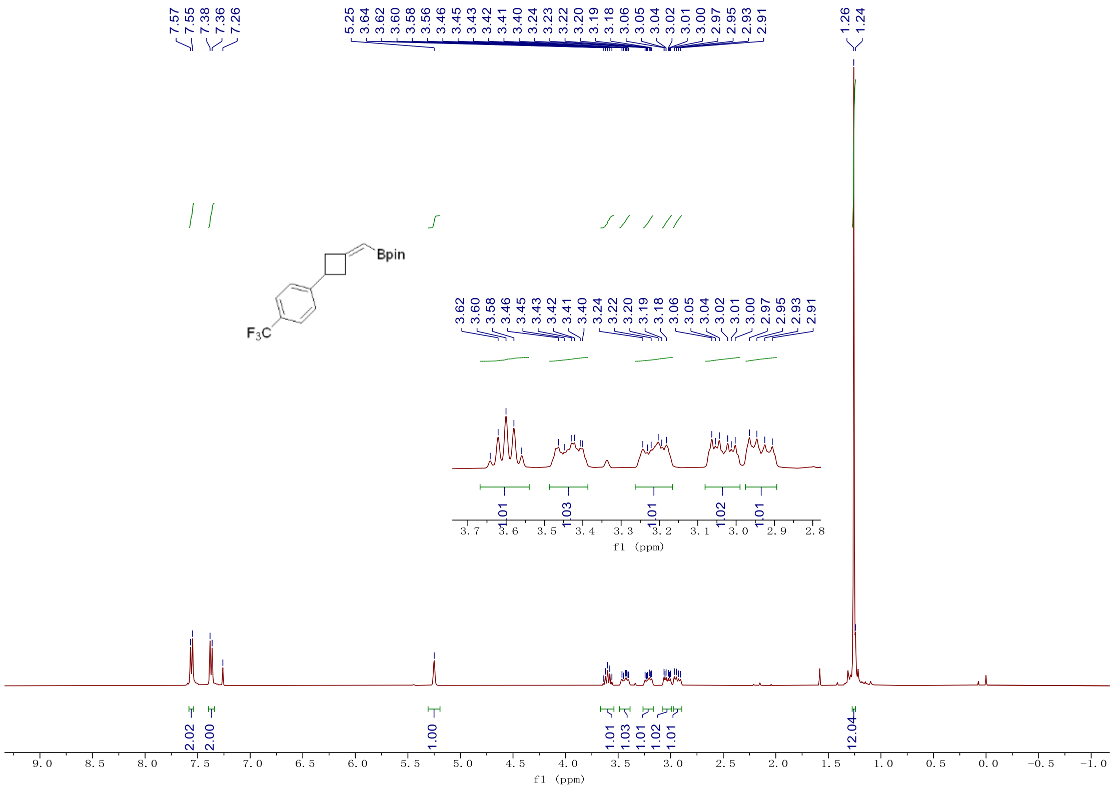

^{1}H NMR of compound 2g

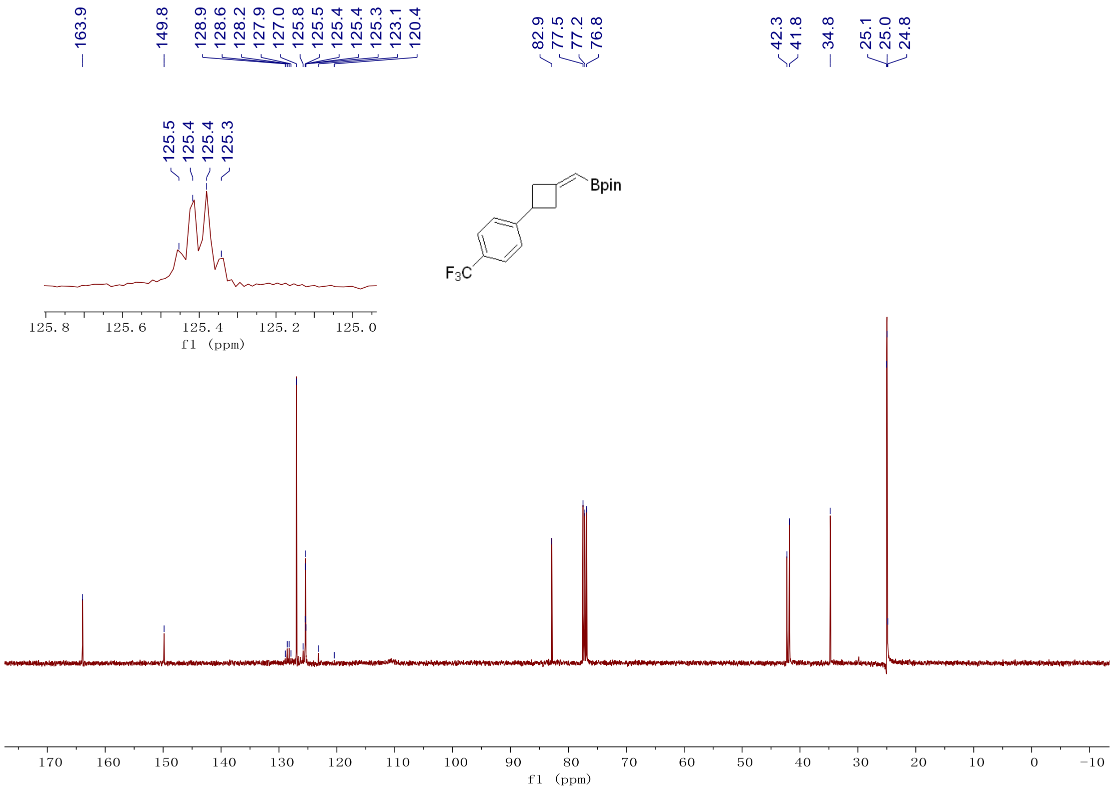

^{13}C NMR of compound 2g

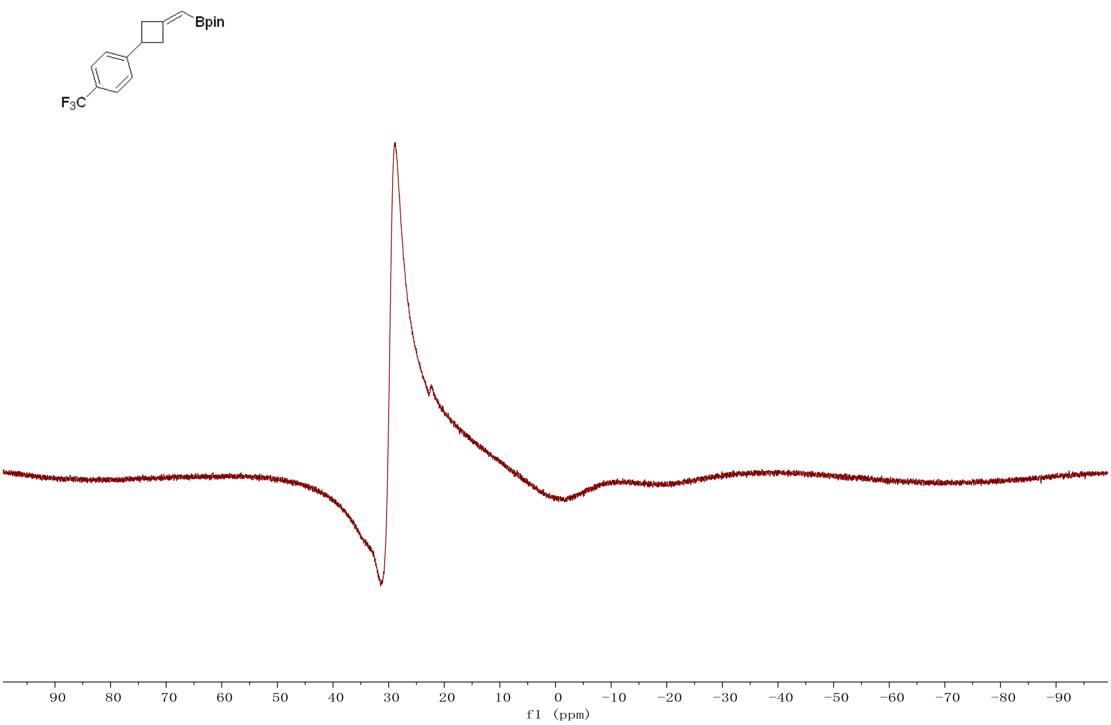

^{11}B NMR of compound 2g

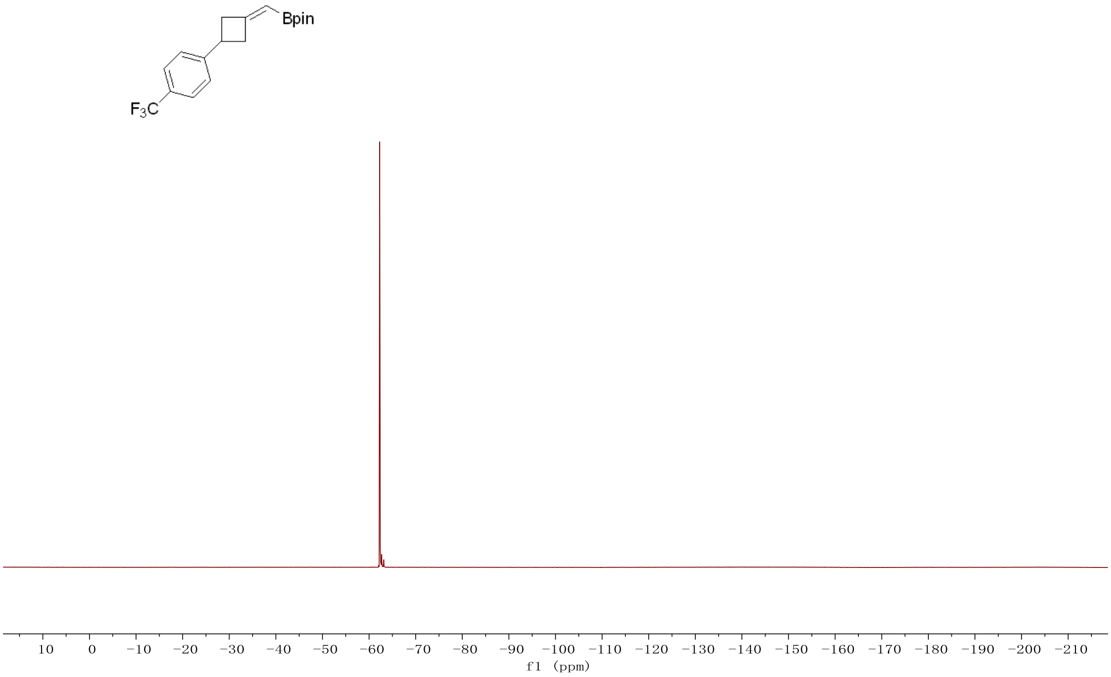

^{19}F NMR of compound 2g

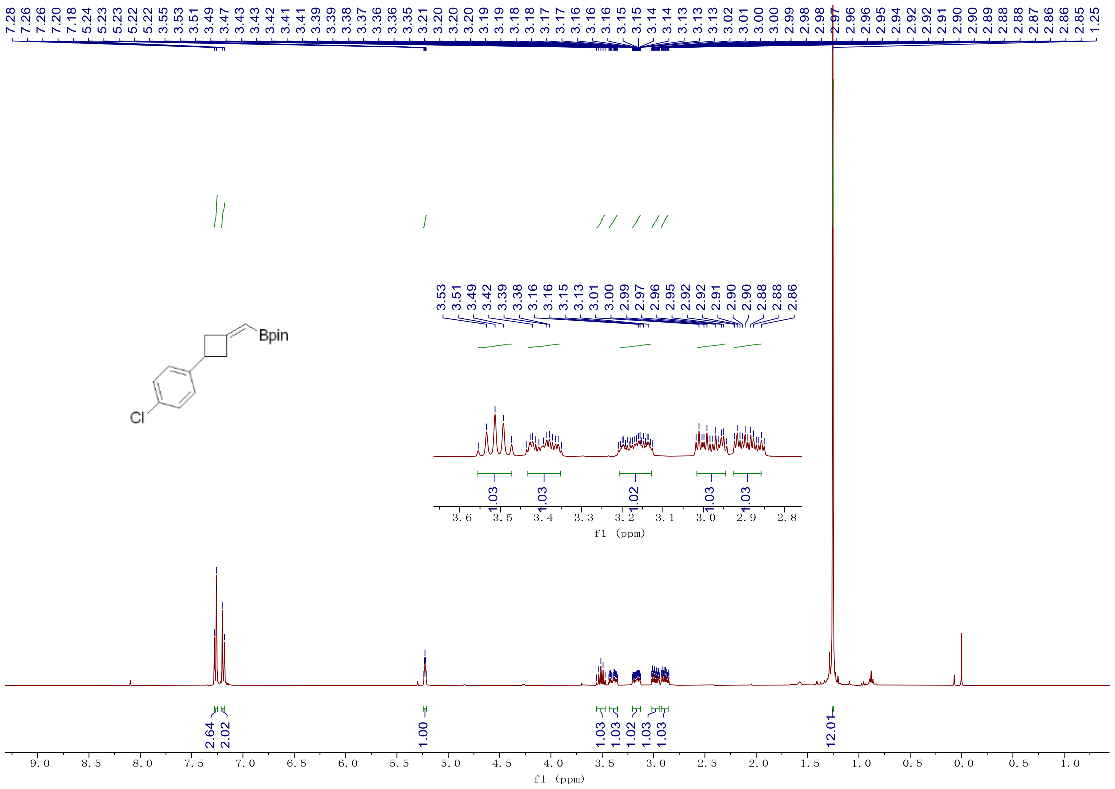

^{1}H NMR of compound 2h

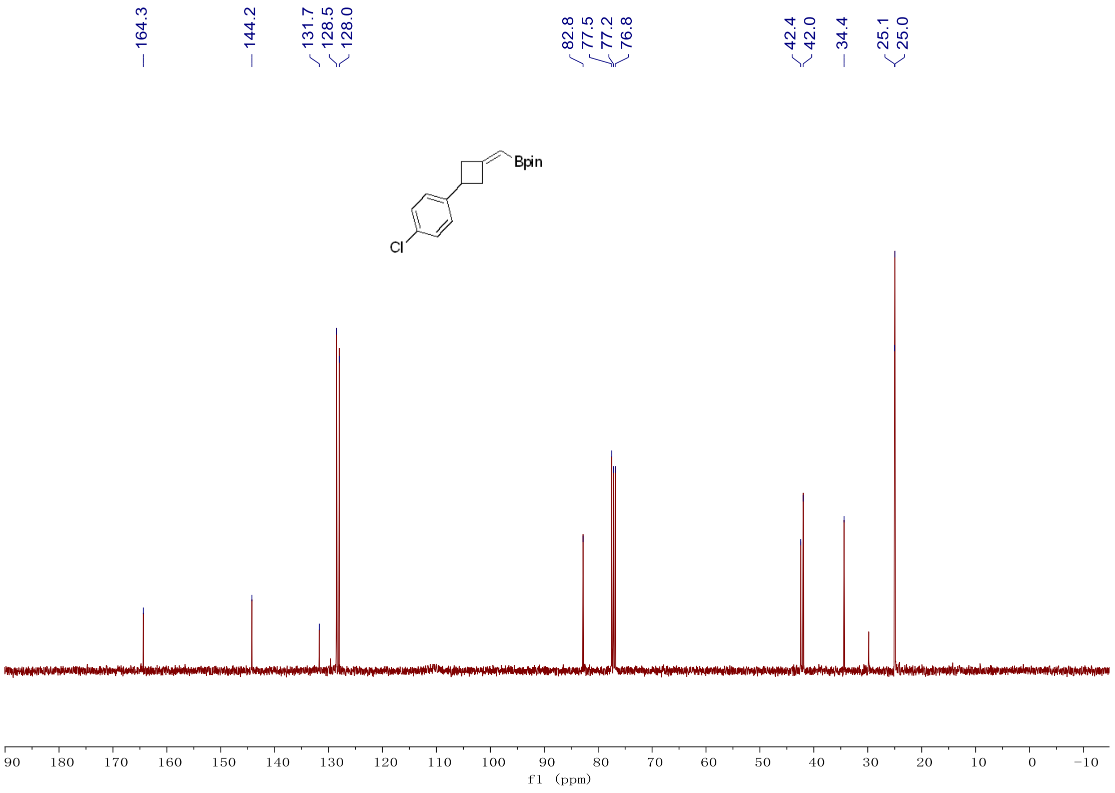

^{13}C NMR of compound 2h

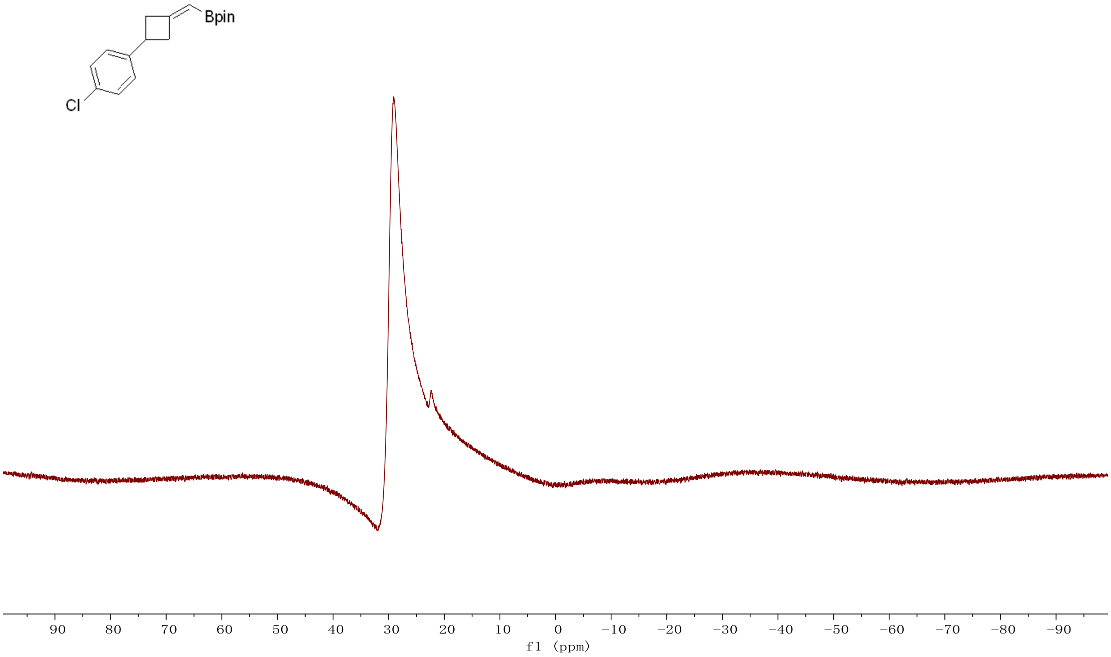

^{11}B NMR of compound 2h

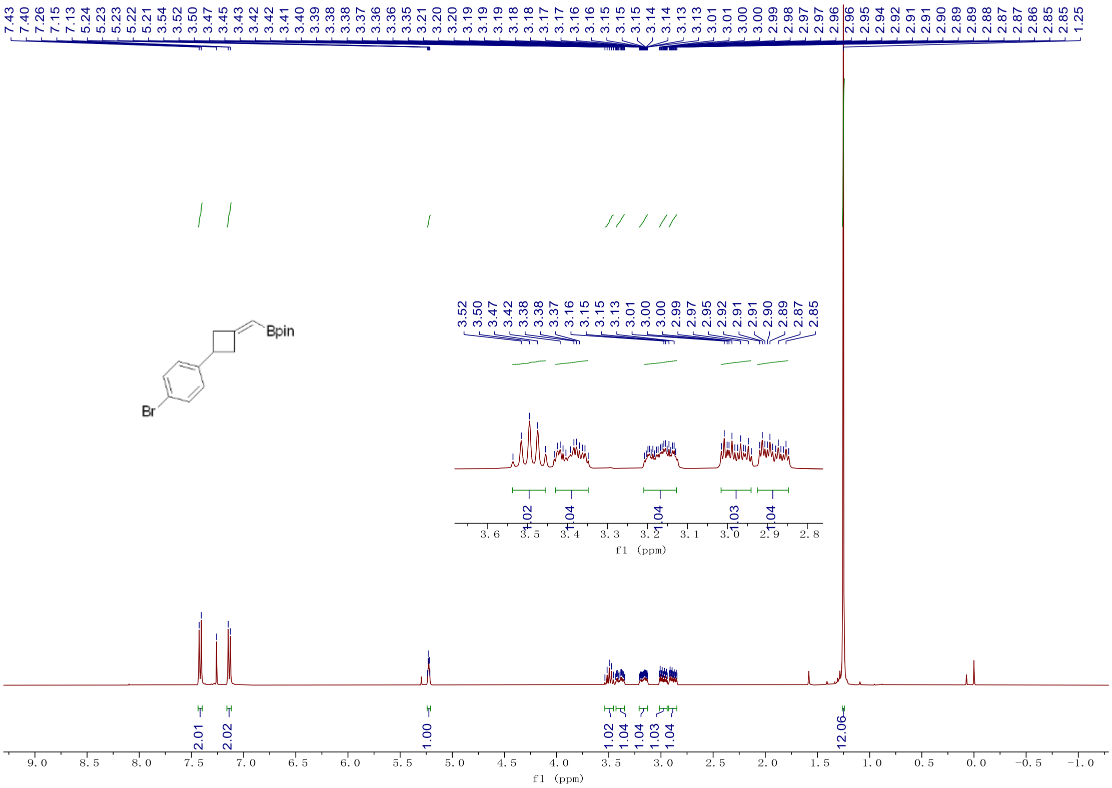

^{1}H NMR of compound 2i

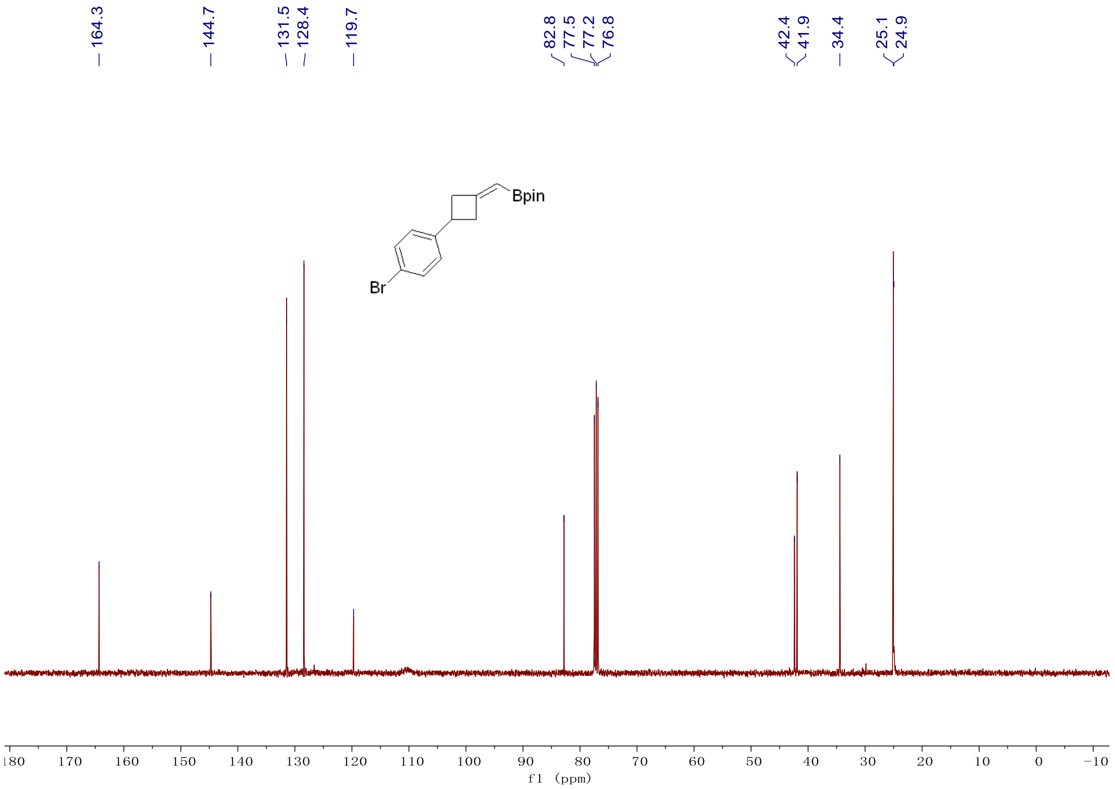

^{13}C NMR of compound 2i

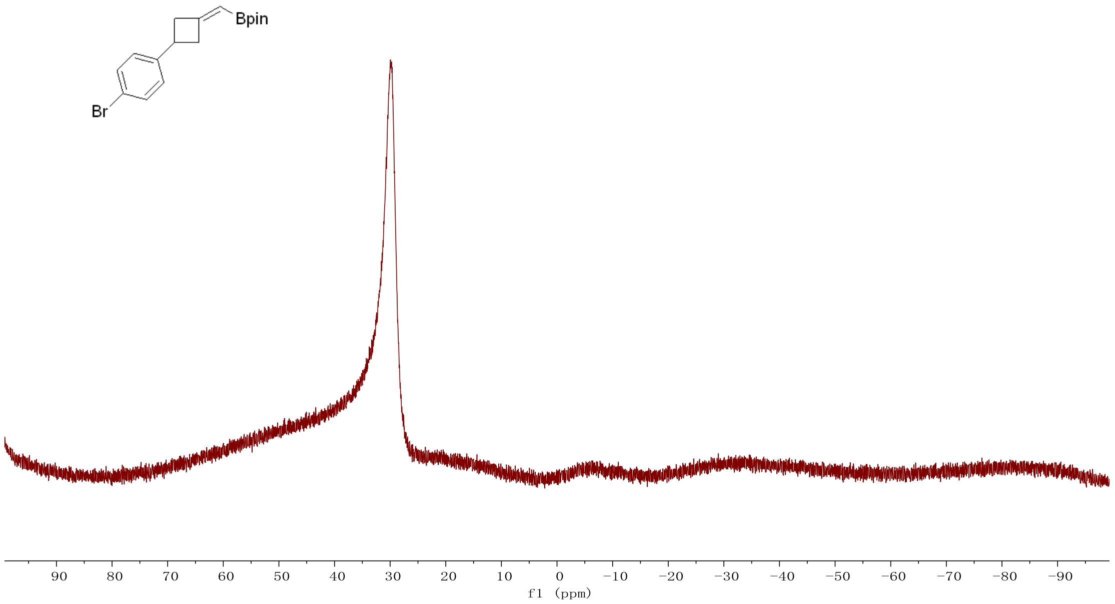

^{11}B NMR of compound 2i

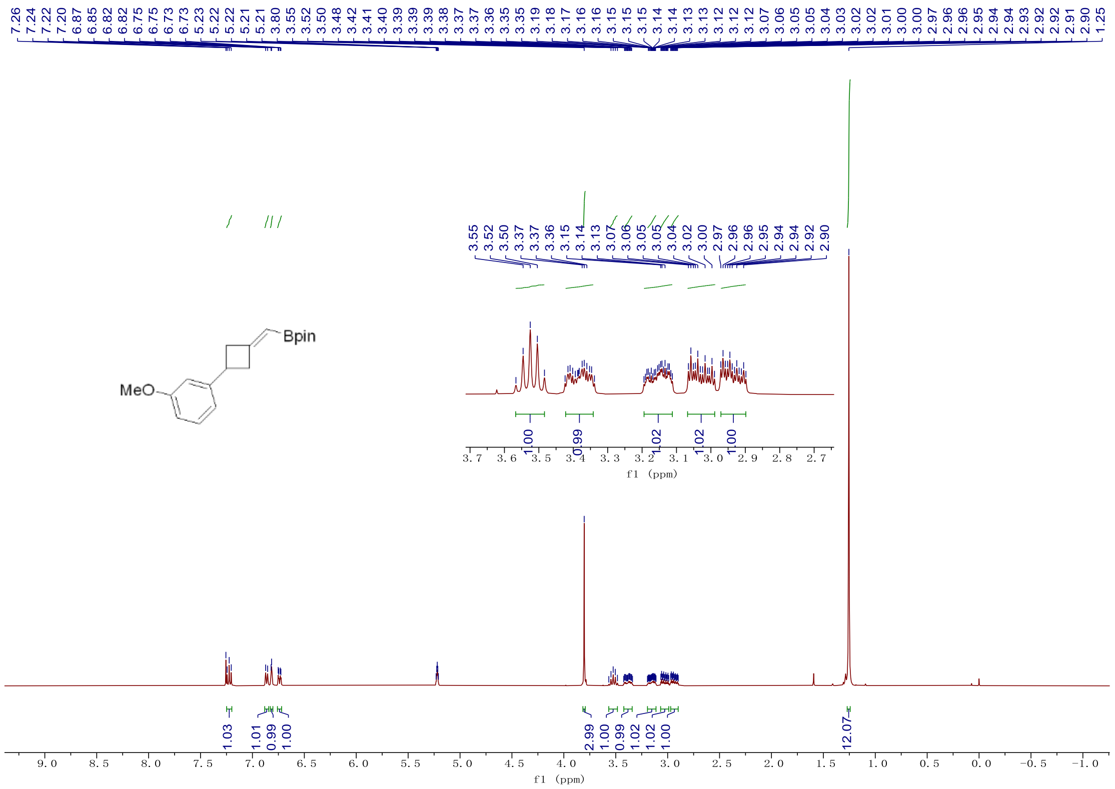

^{1}H NMR of compound 2j

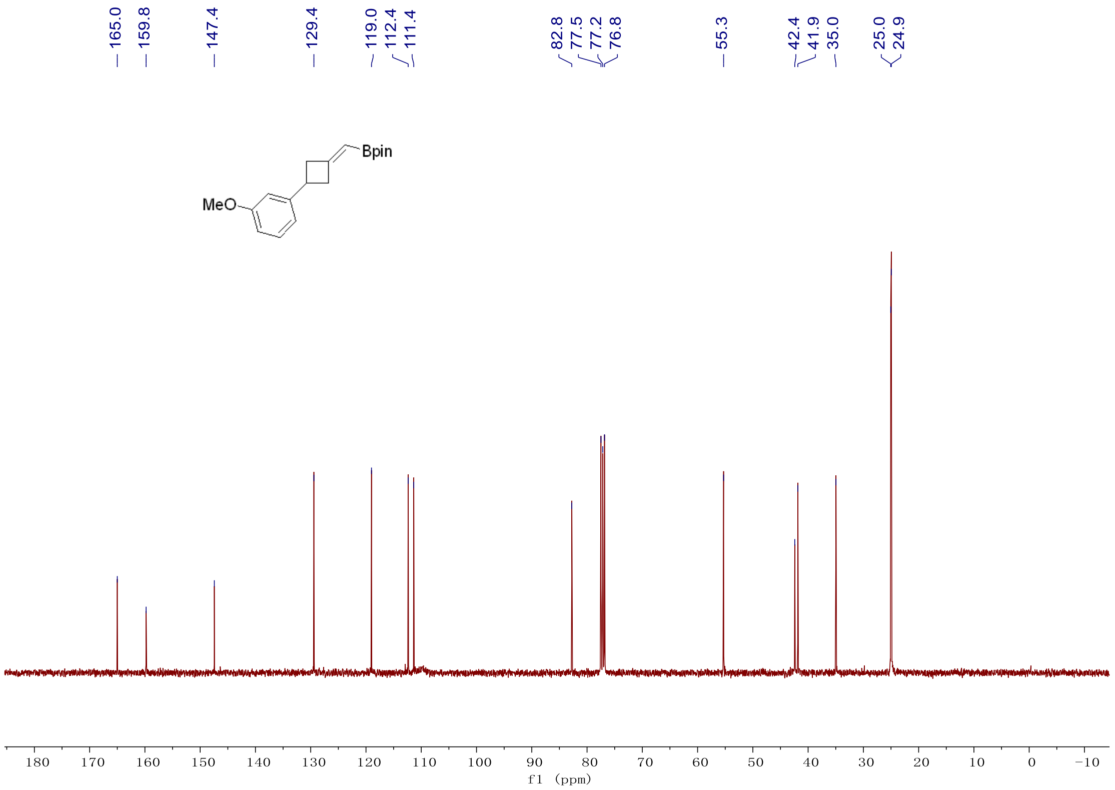

^{13}C NMR of compound 2j

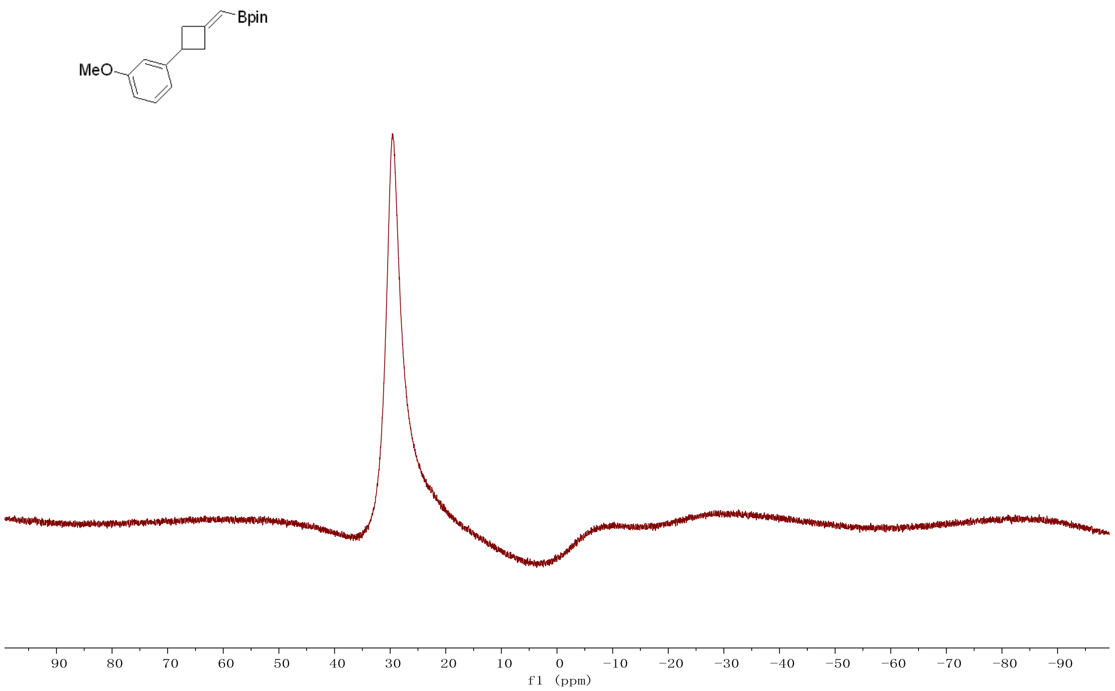

^{11}B NMR of compound 2j

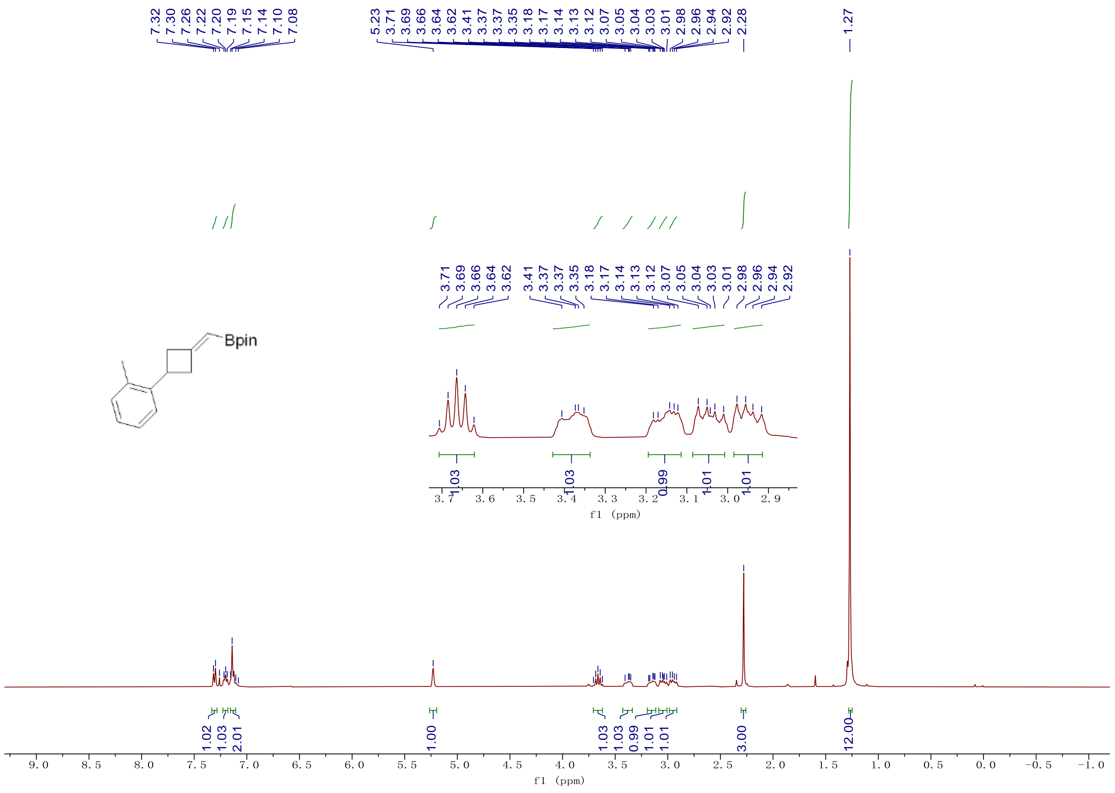

^{1}H NMR of compound 2k

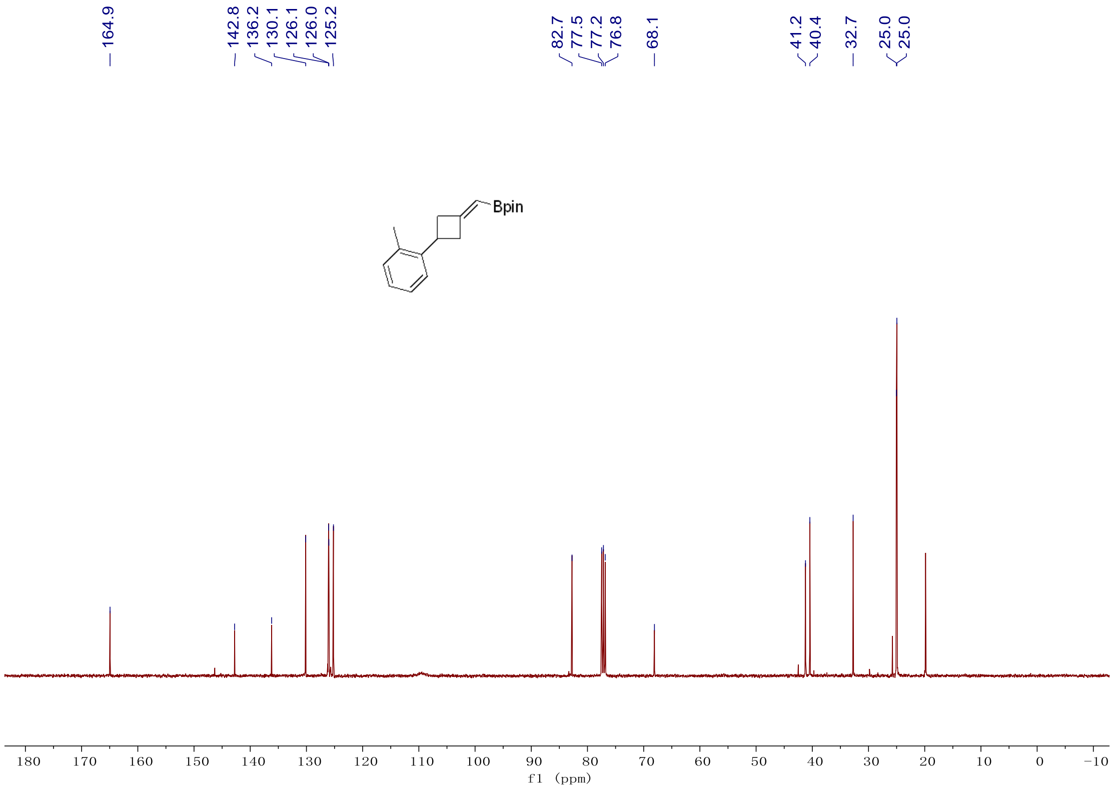

^{13}C NMR of compound 2k

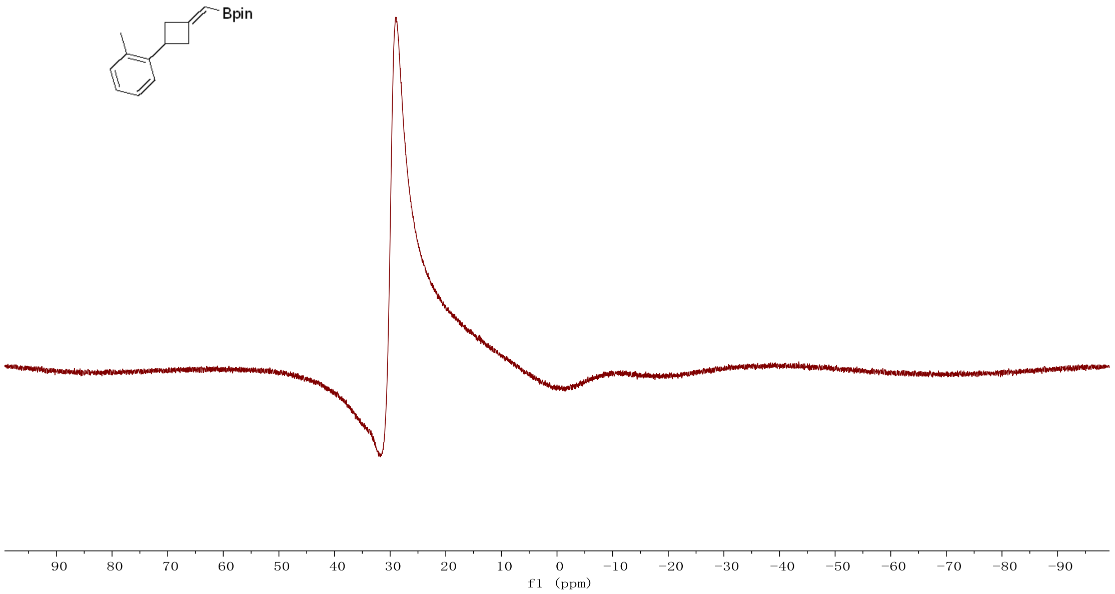

^{11}B NMR of compound 2k

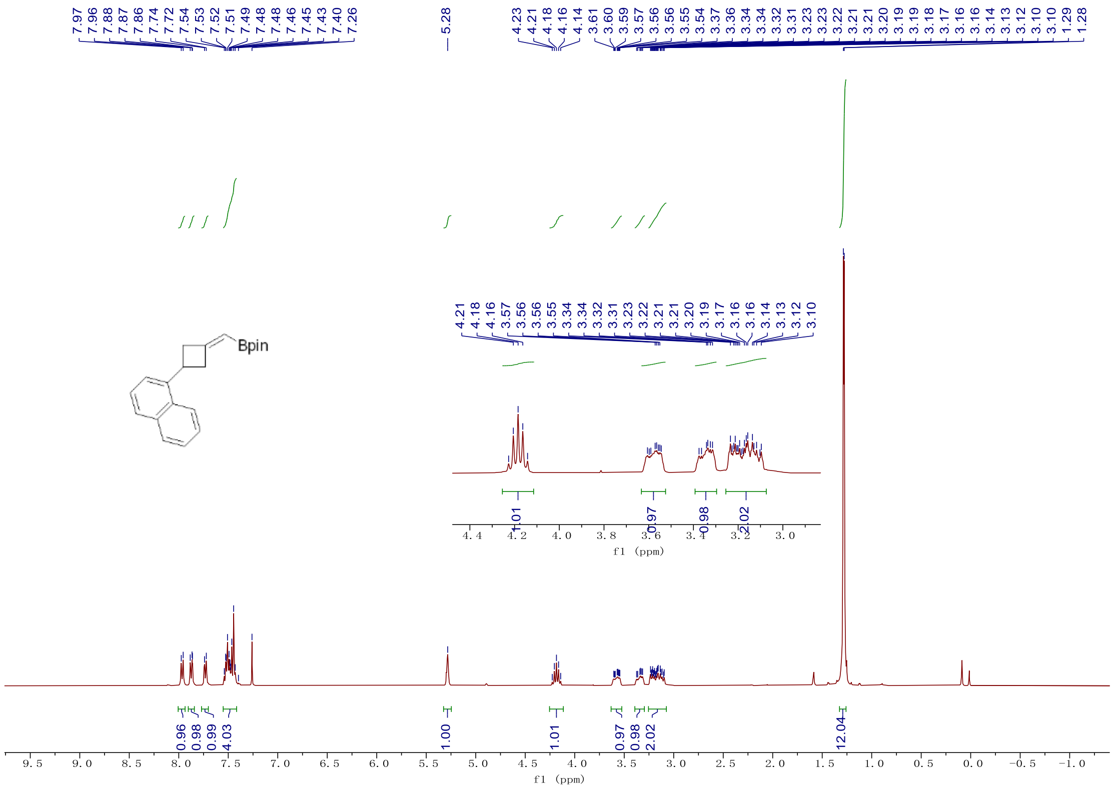

^{1}H NMR of compound 2l

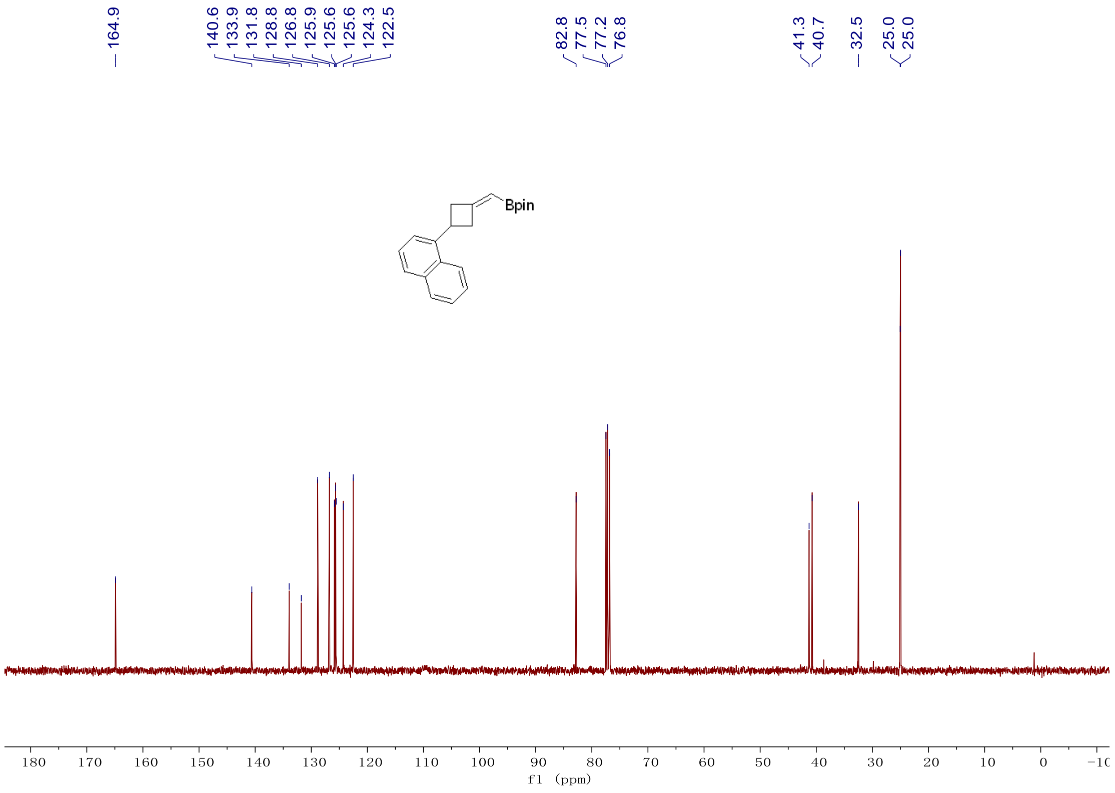

^{13}C NMR of compound 2l

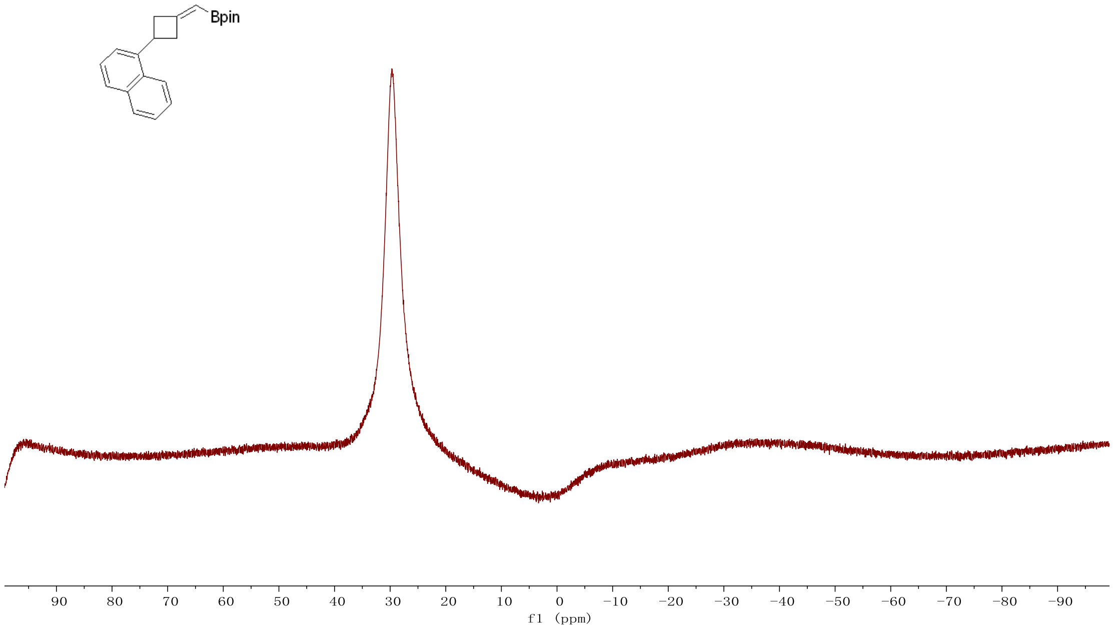

^{11}B NMR of compound 2l

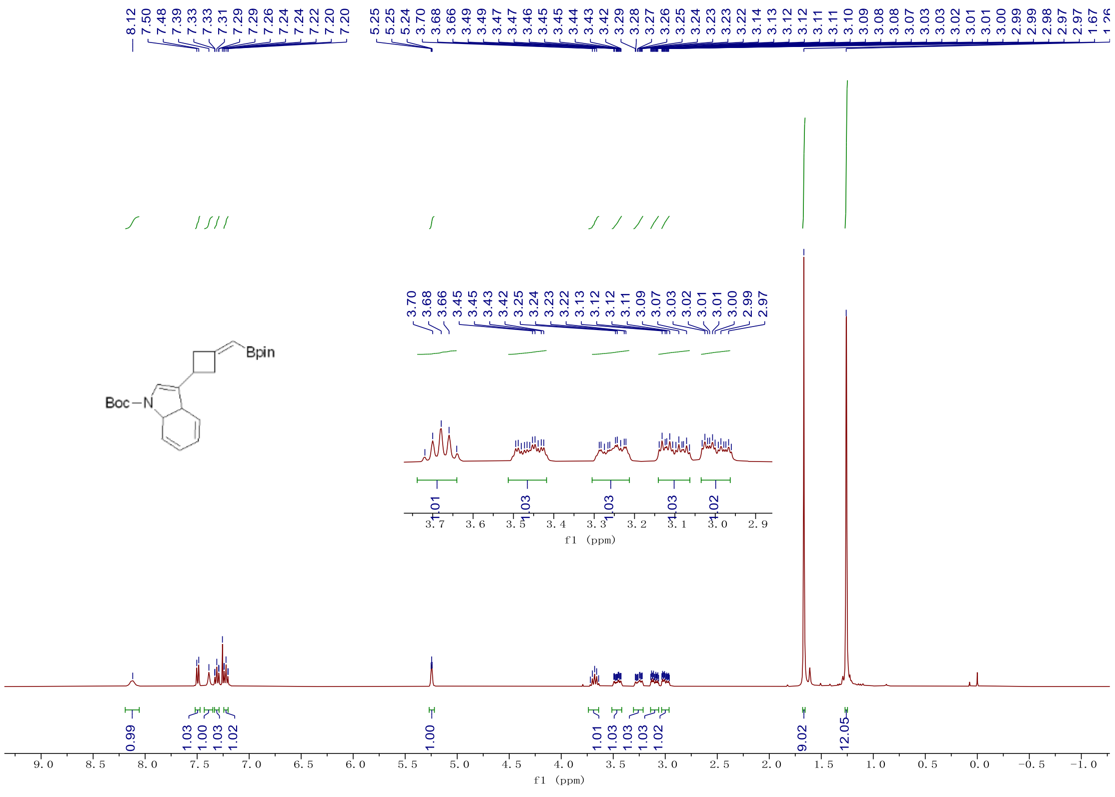

^{1}H NMR of compound 2m

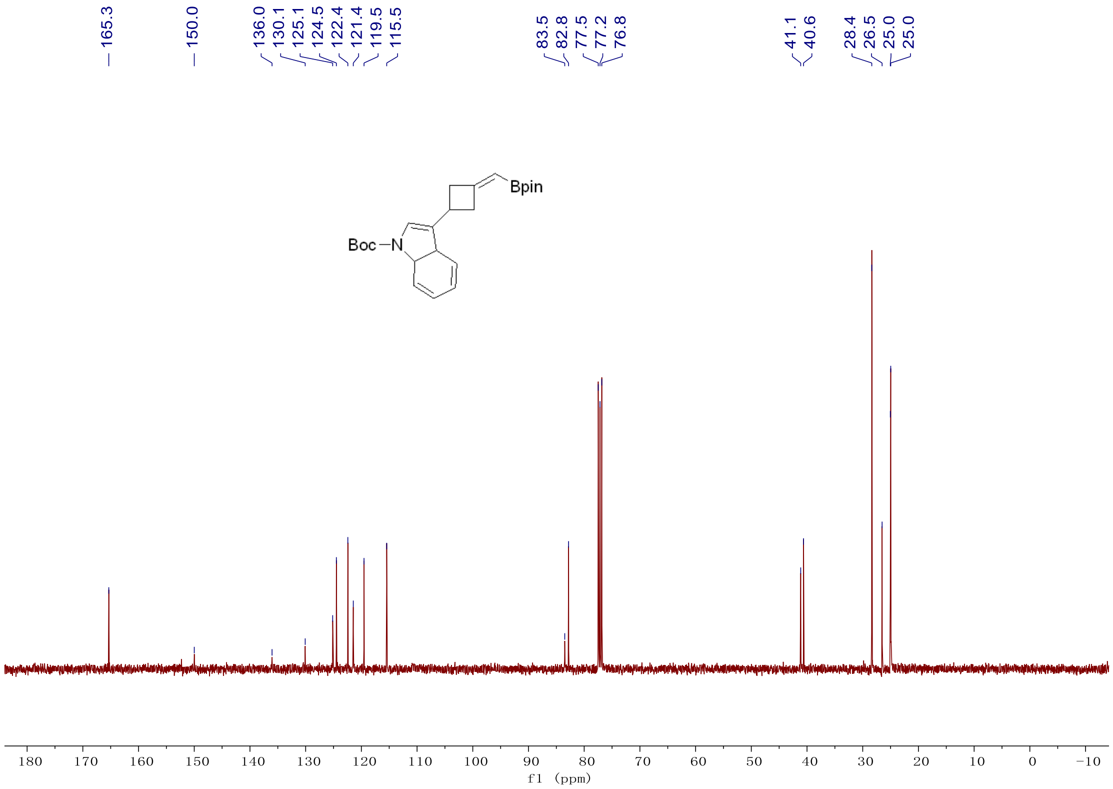

^{13}C NMR of compound 2m

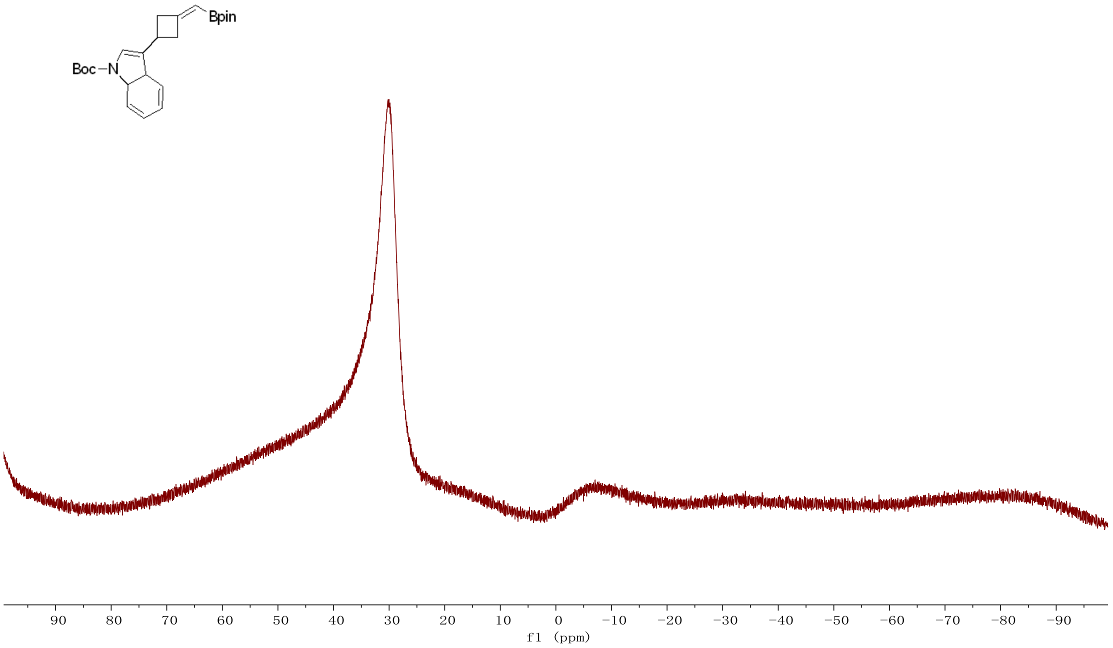

^{11}B NMR of compound 2m

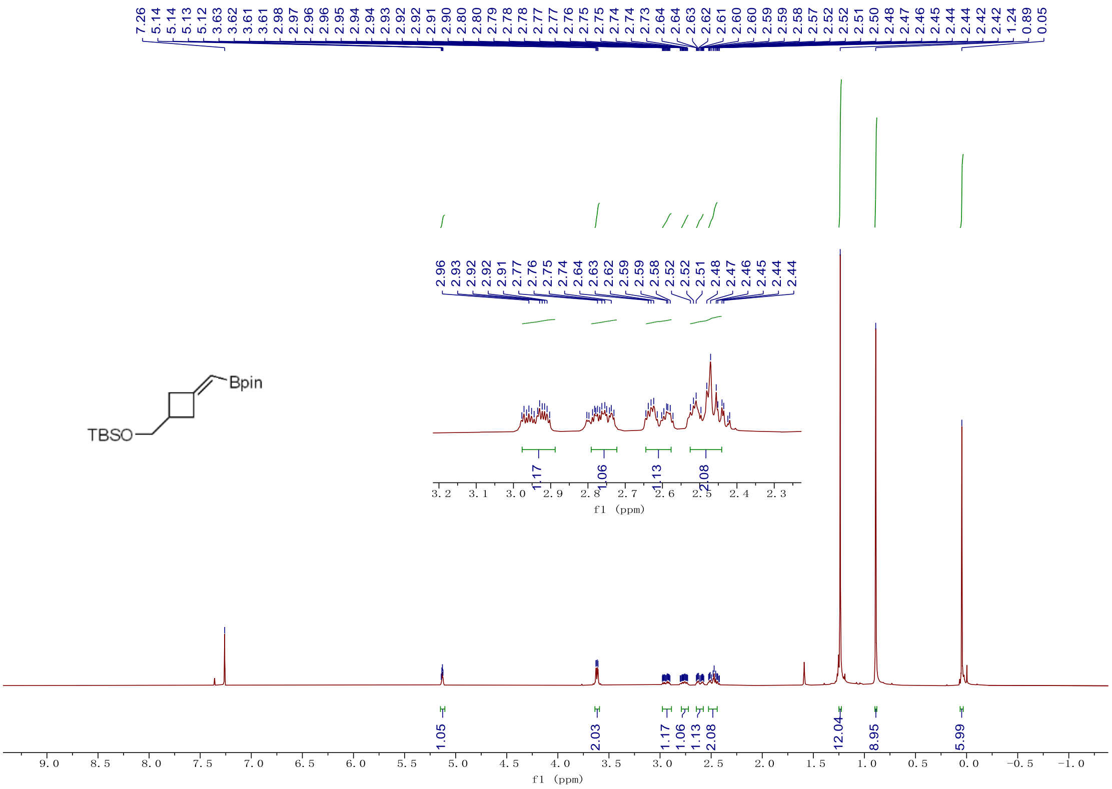

^{1}H NMR of compound 2n

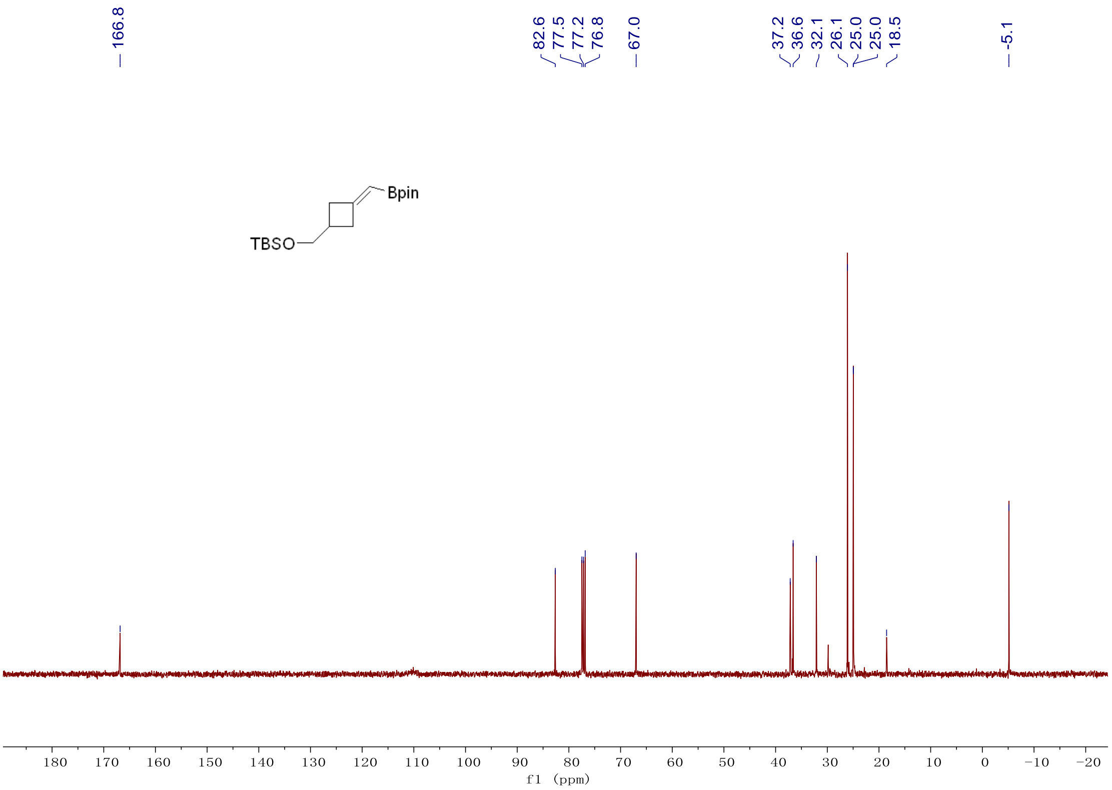

^{13}C NMR of compound 2n

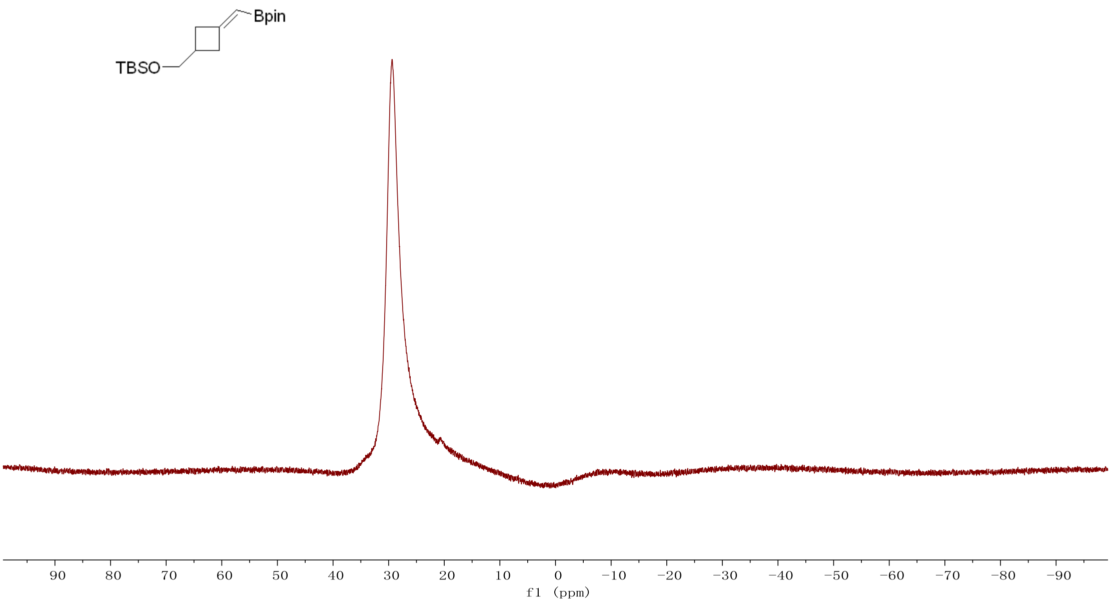

^{11}B NMR of compound 2n

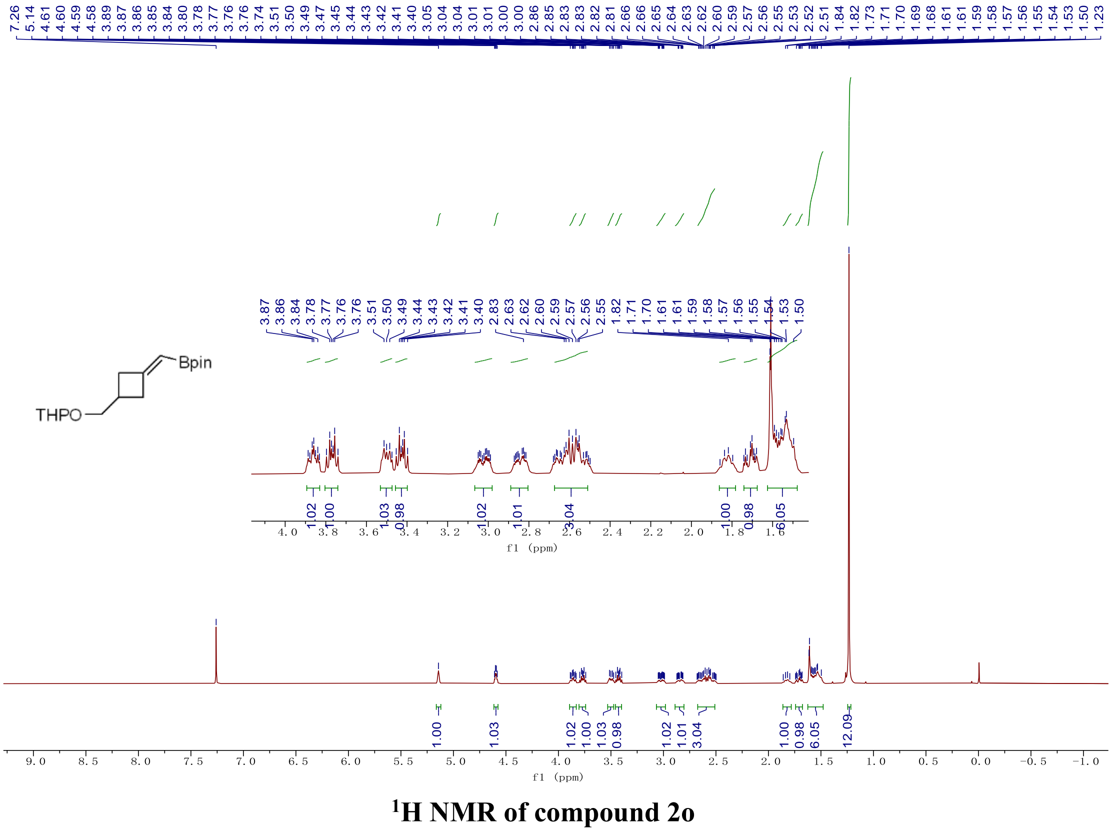

^{1}H NMR of compound 2o

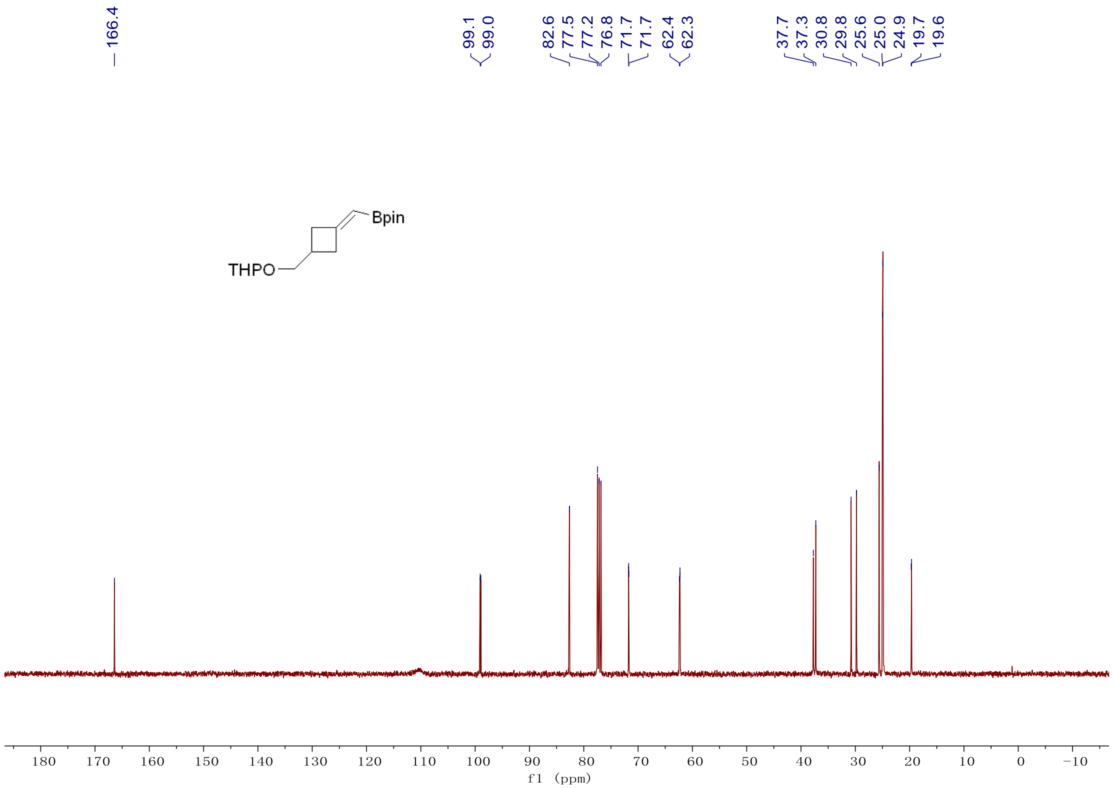

^{13}C NMR of compound 2o

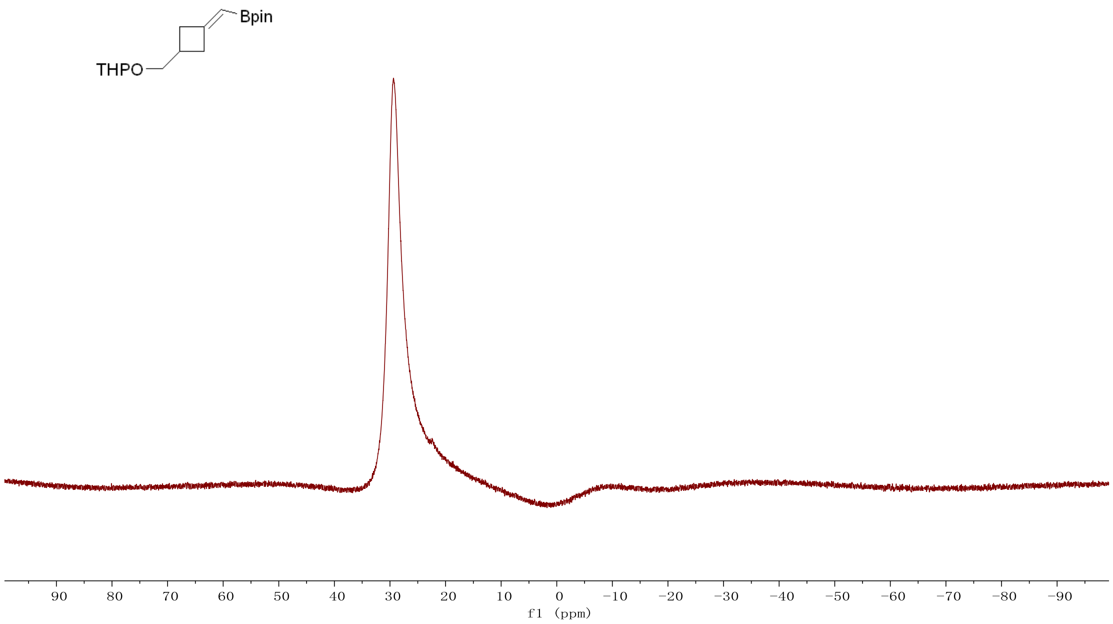

^{11}B NMR of compound 2o

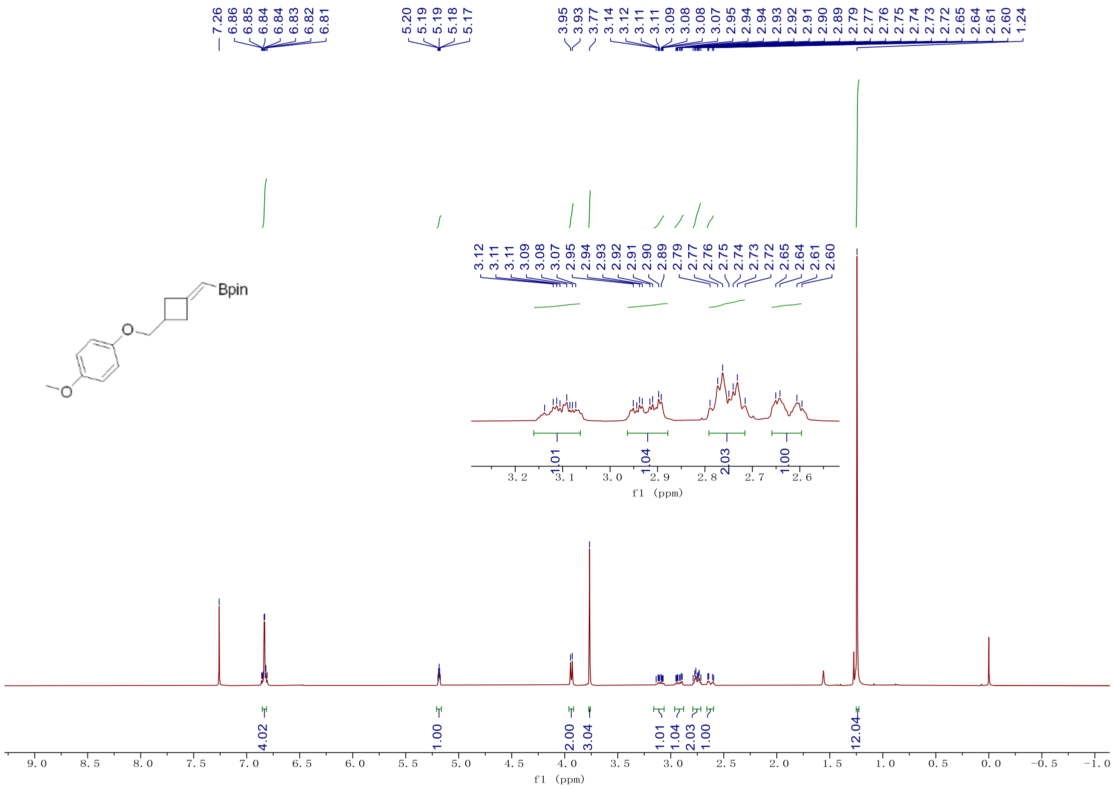

^{1}H NMR of compound 2p

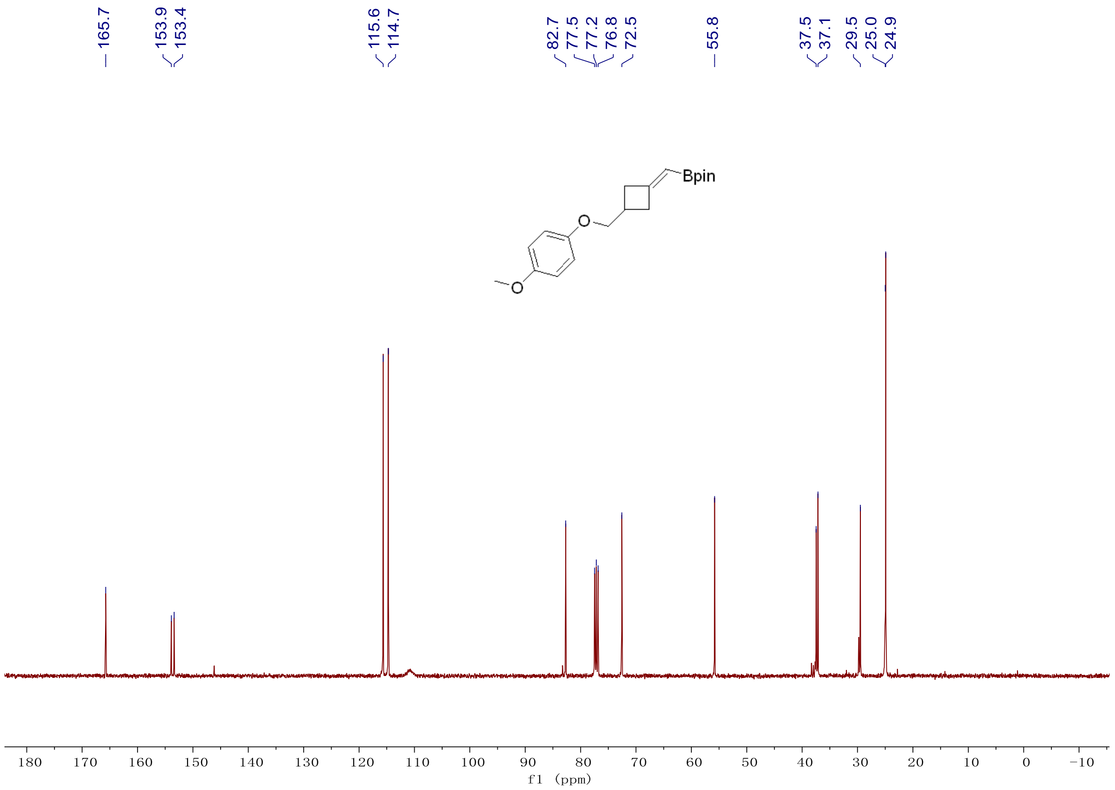

^{13}C NMR of compound 2p

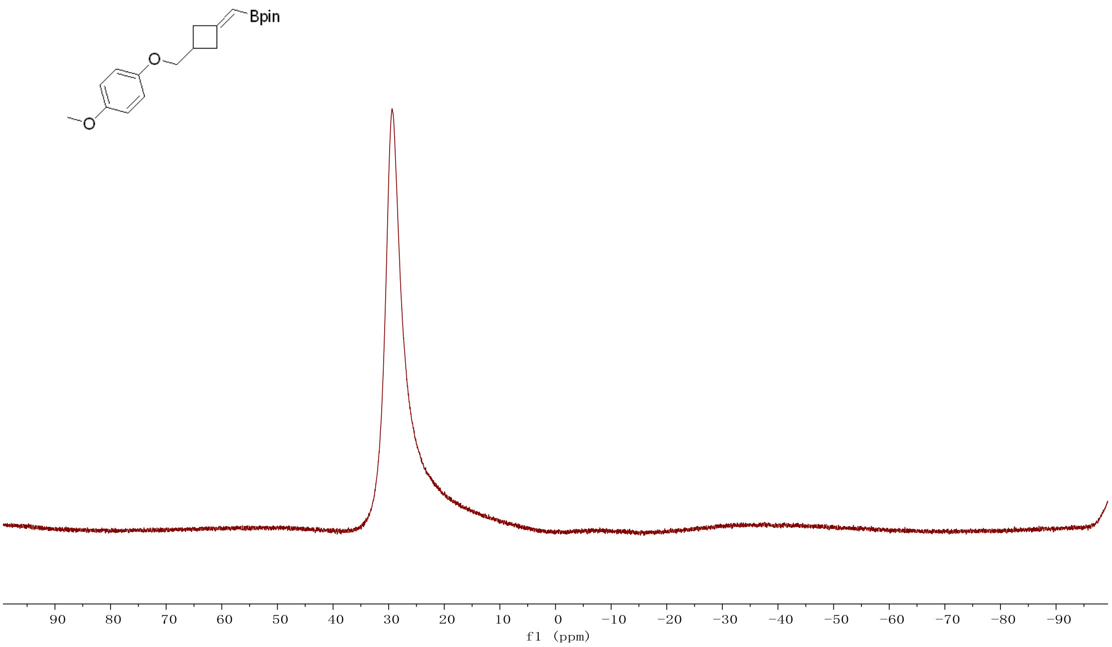

^{11}B NMR of compound 2p

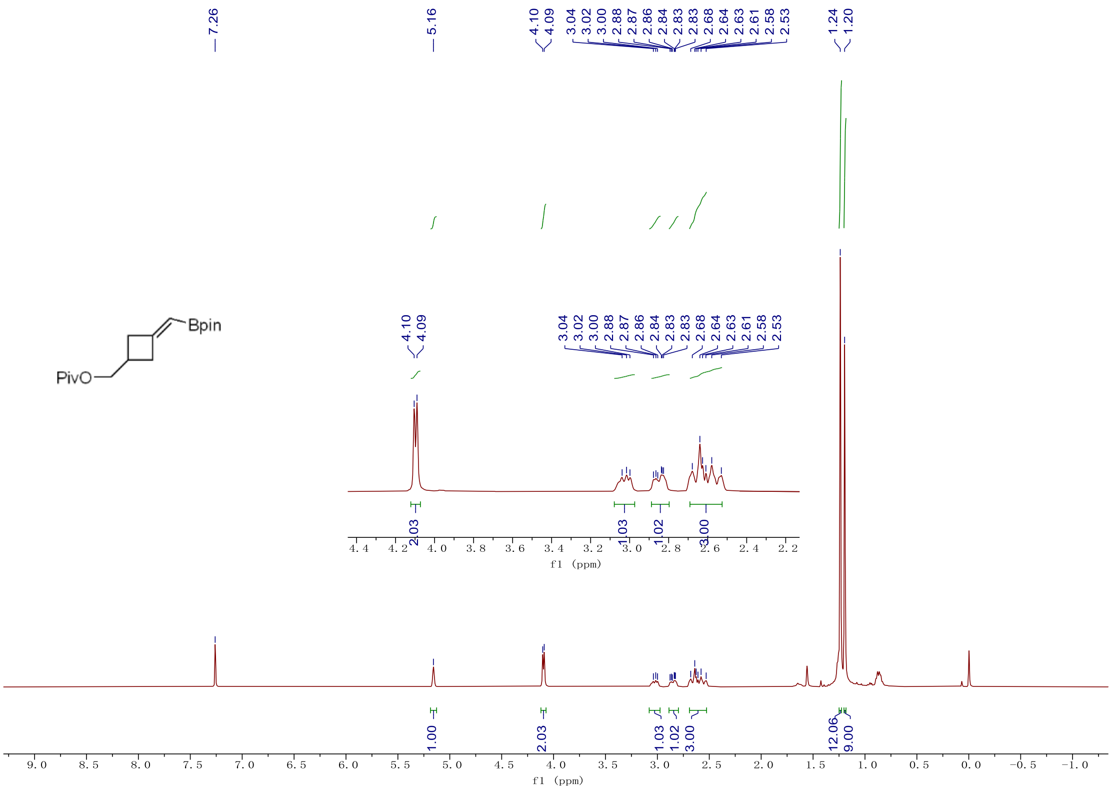

^{1}H NMR of compound 2q

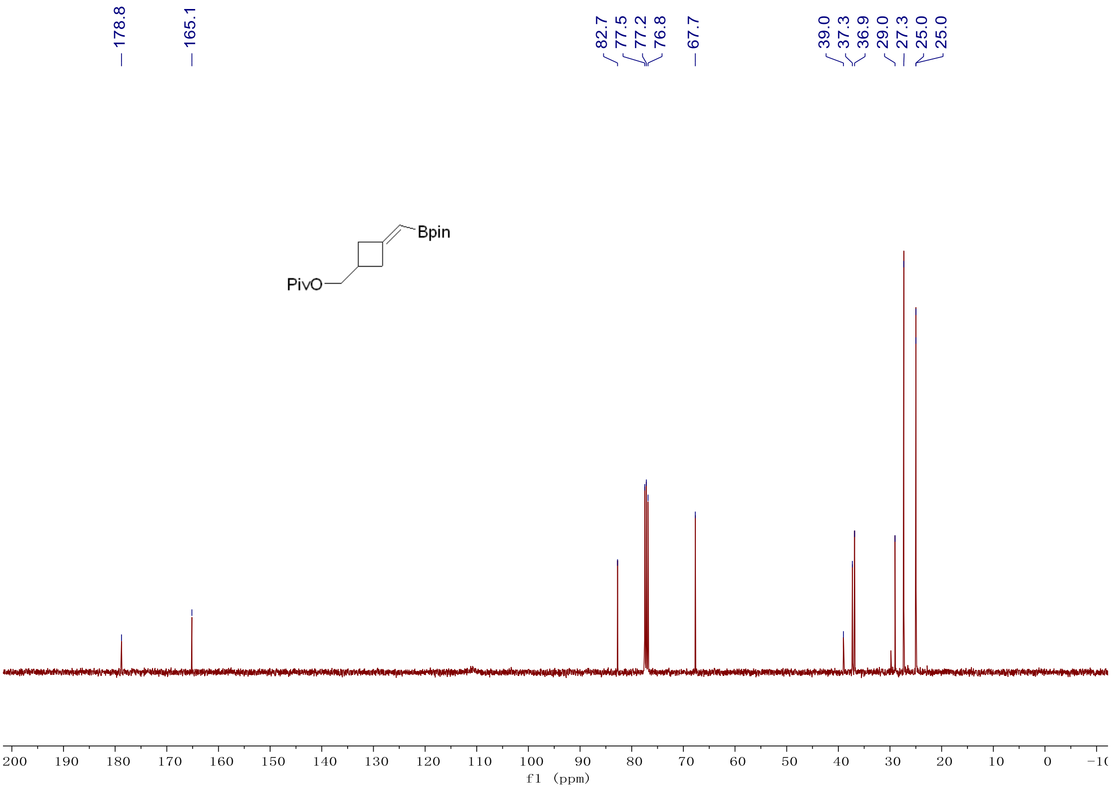

^{13}C NMR of compound 2q

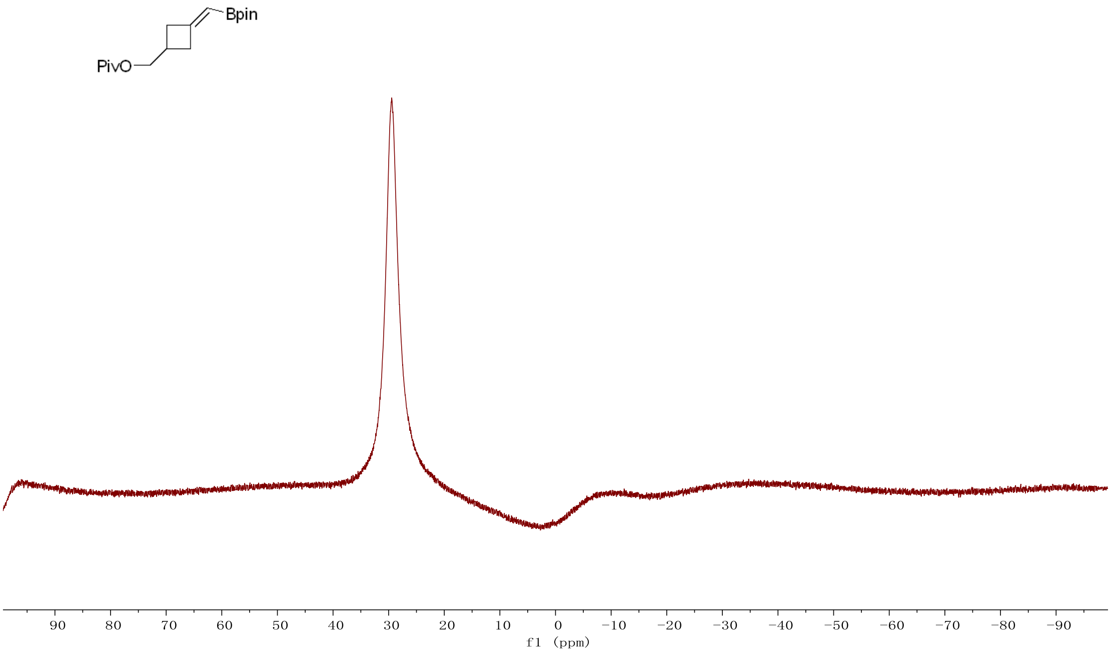

^{11}B NMR of compound 2q

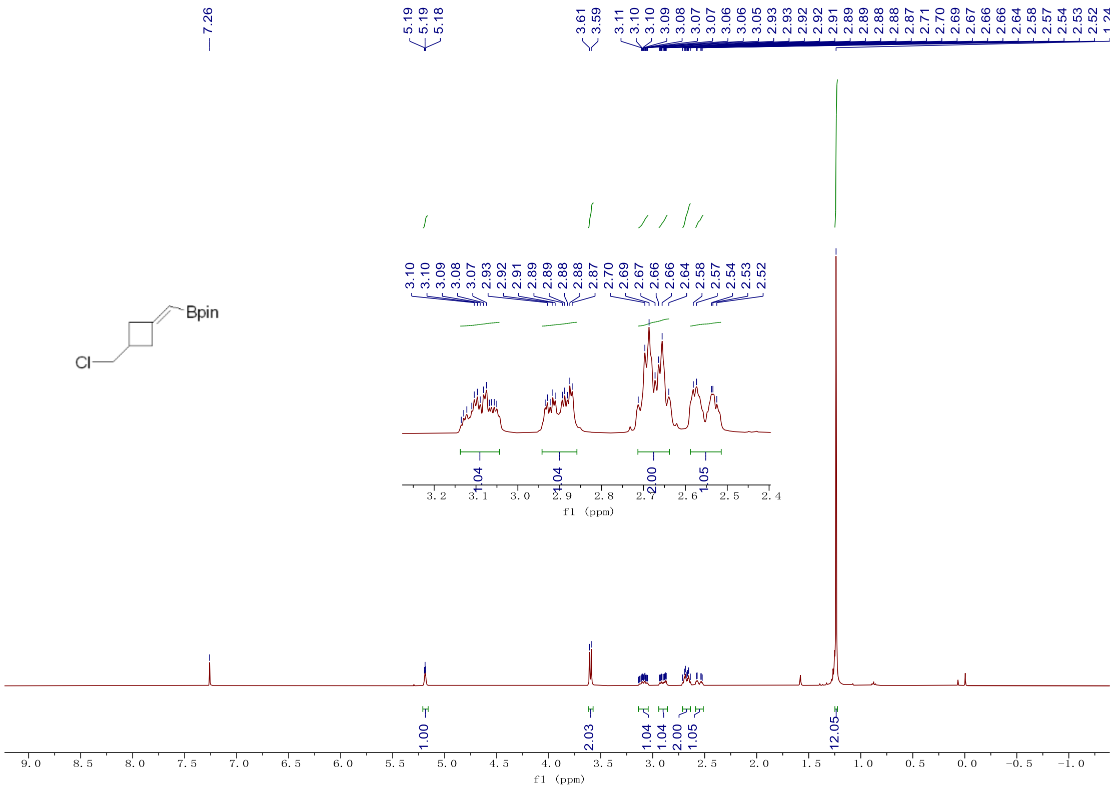

^{1}H NMR of compound 2r

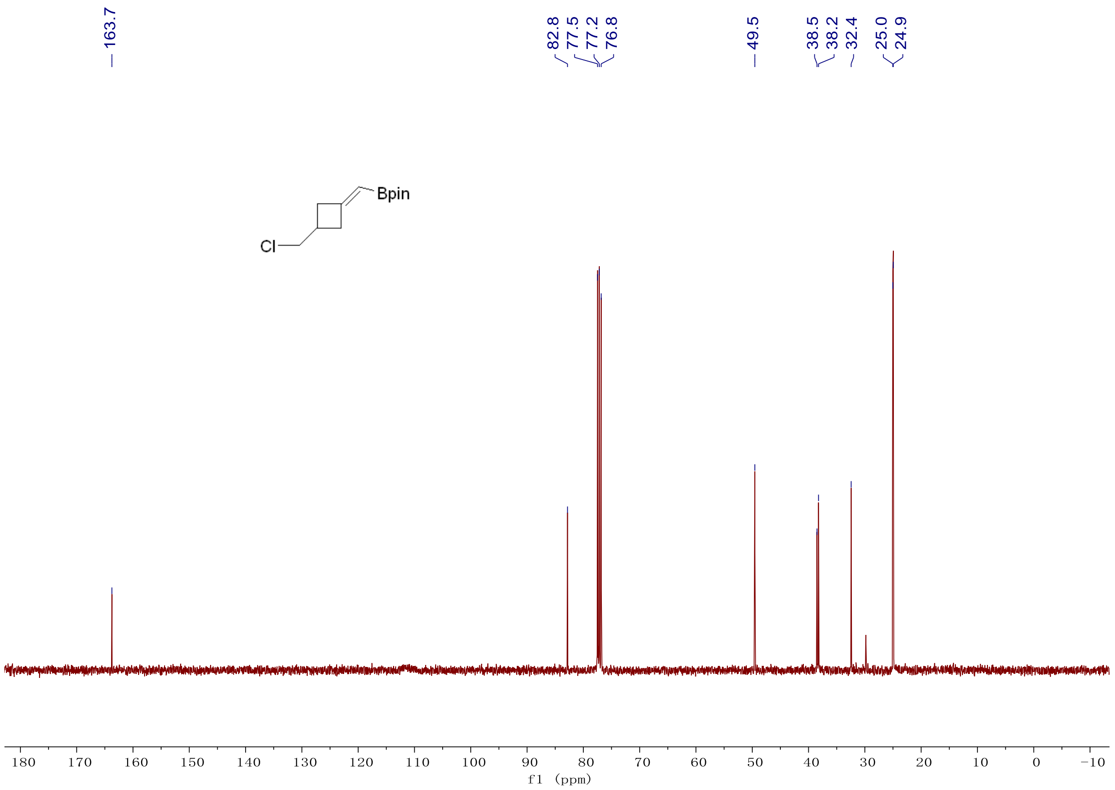

^{13}C NMR of compound 2r

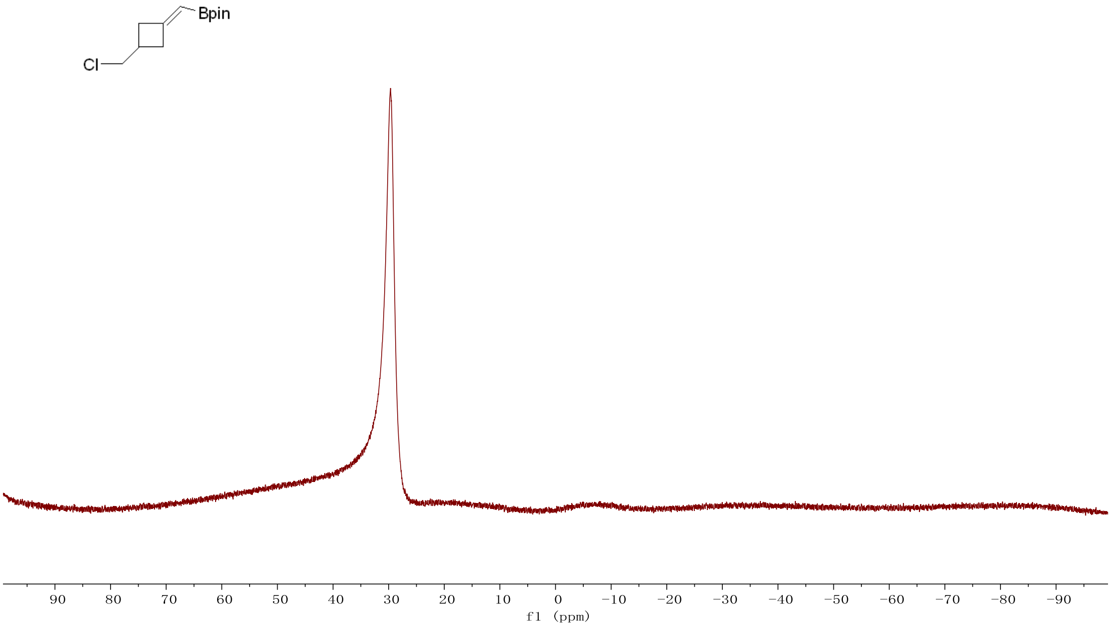

^{11}B NMR of compound 2r

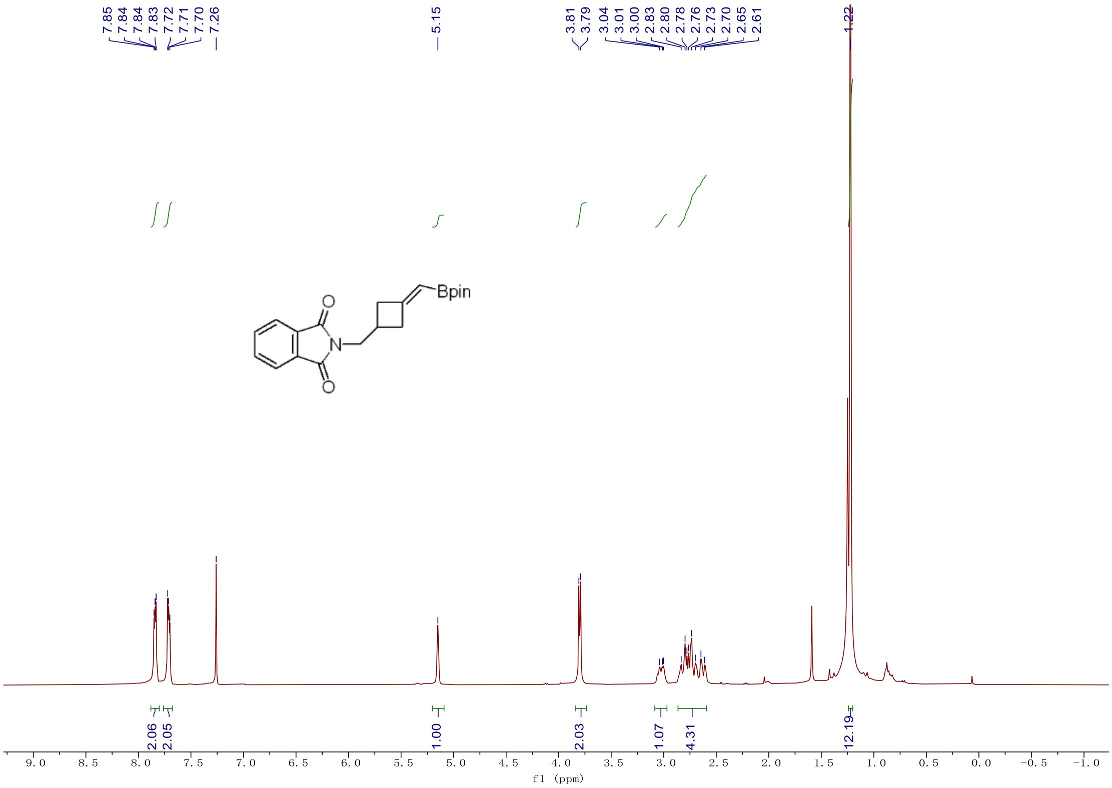

^{1}H NMR of compound 2s

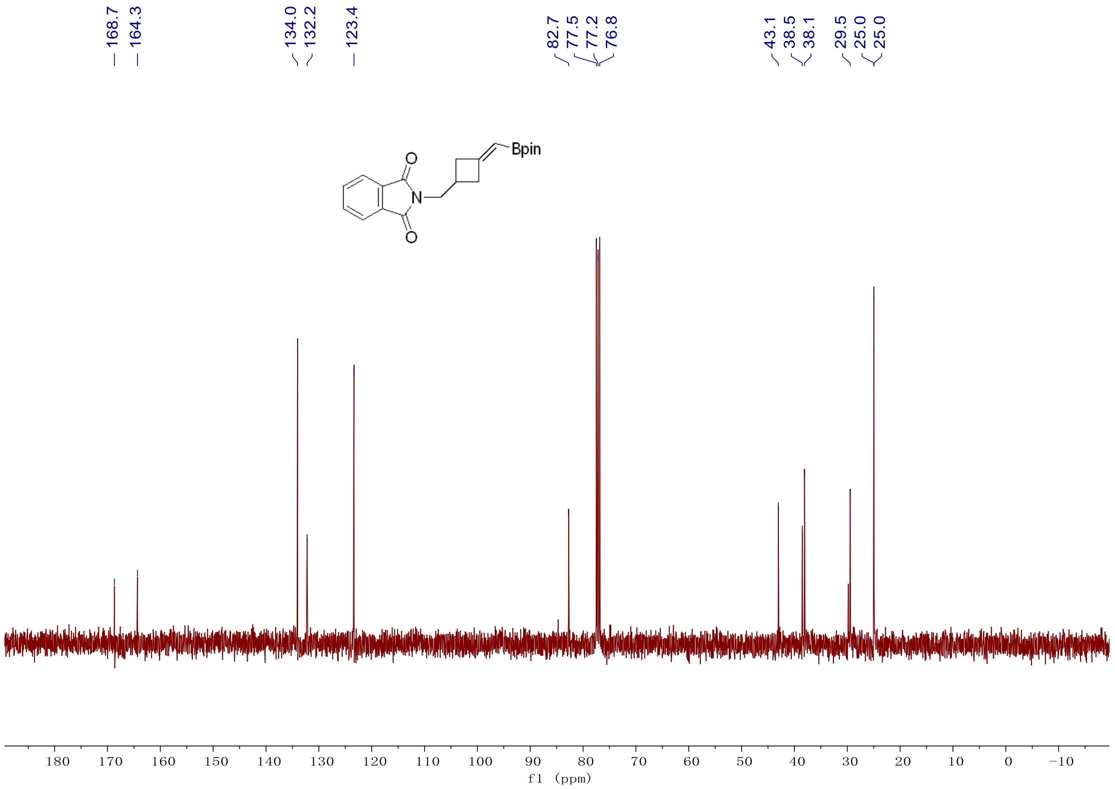

^{13}C NMR of compound 2s

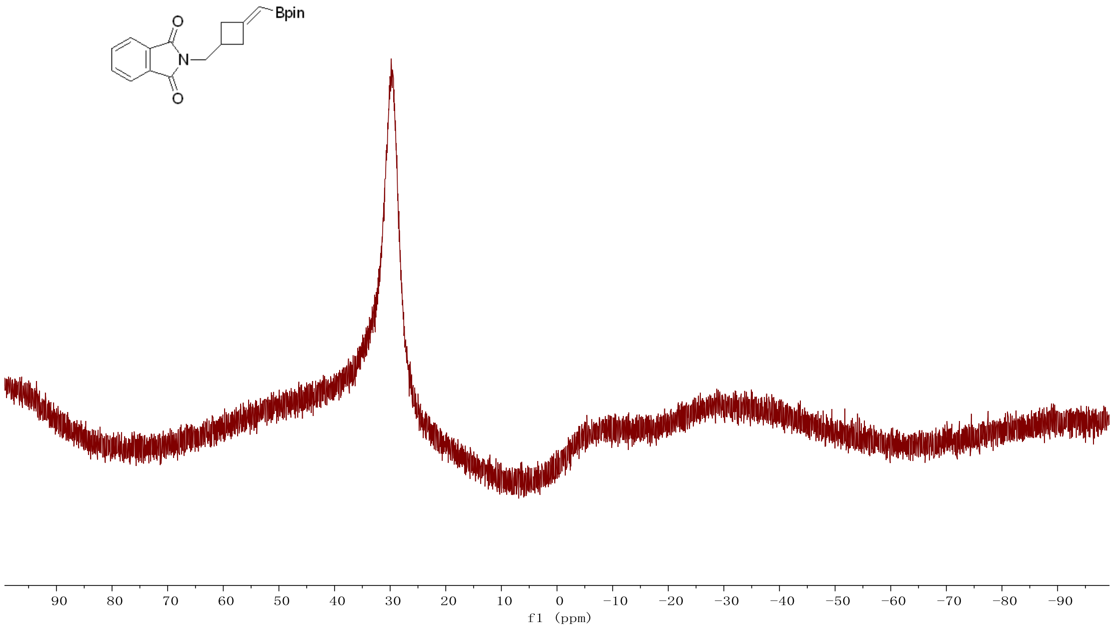

^{11}B NMR of compound 2s

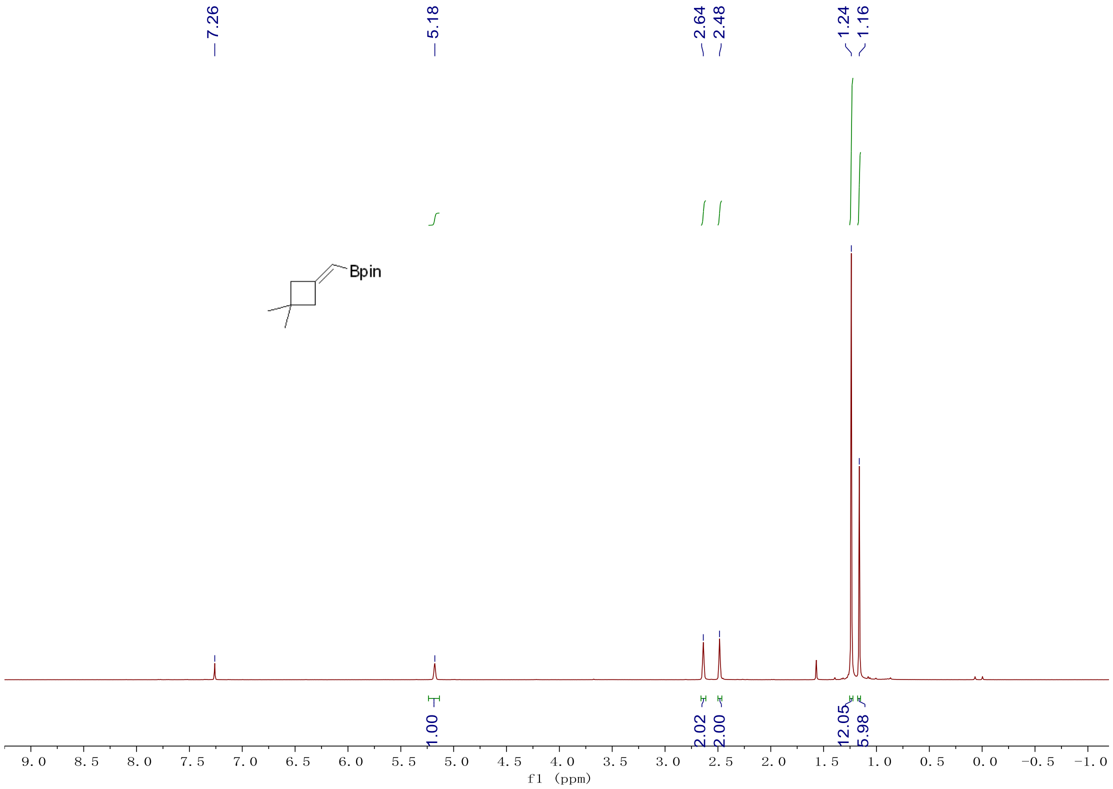

^{1}H NMR of compound 2t

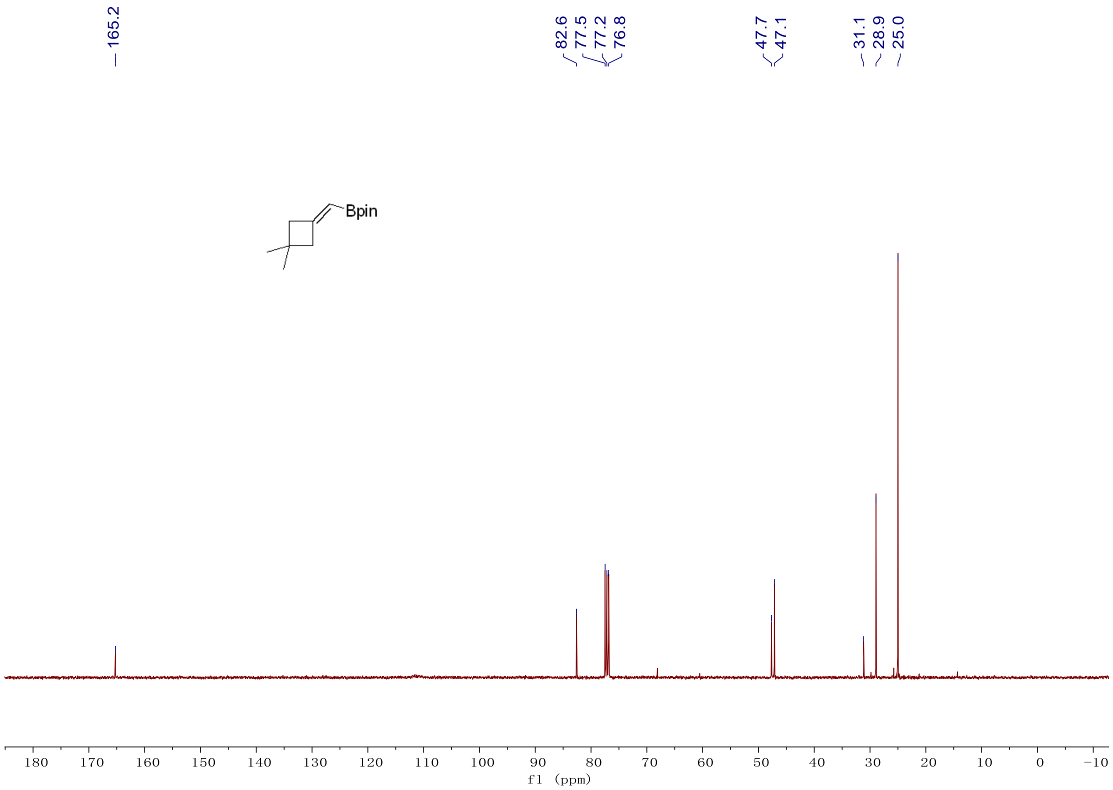

^{13}C NMR of compound 2t

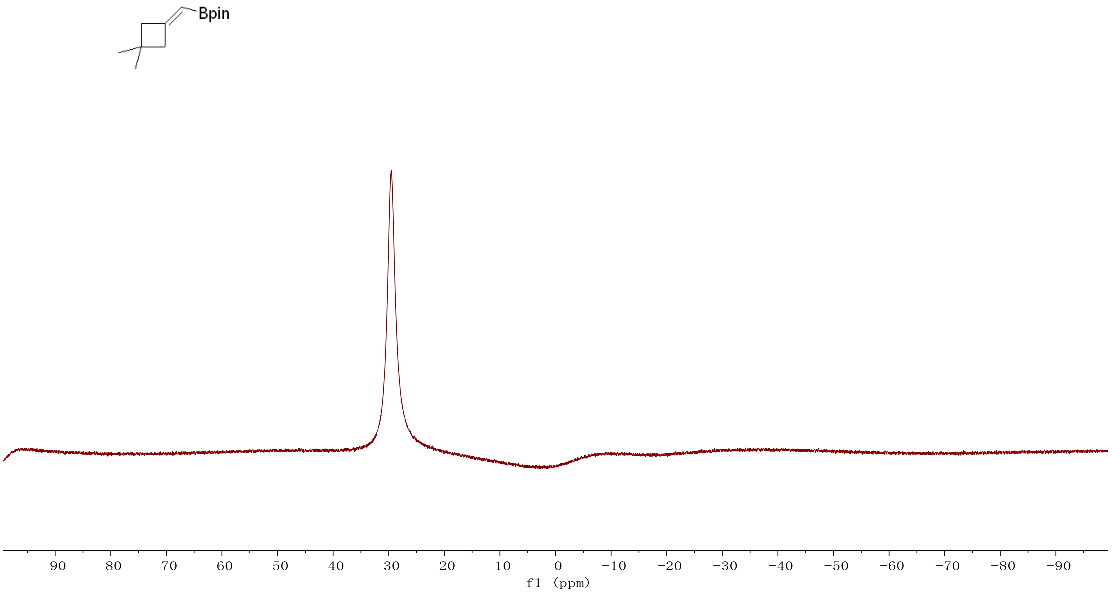

^{11}B NMR of compound 2t

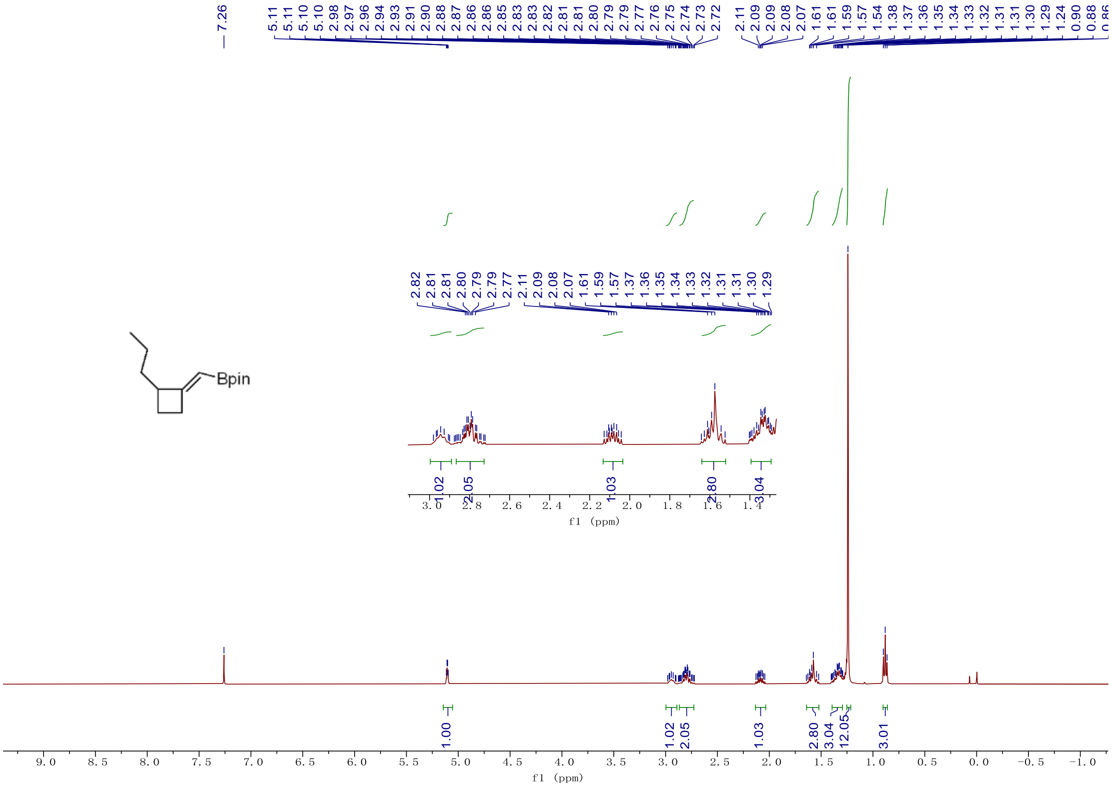

^{1}H NMR of compound 2u

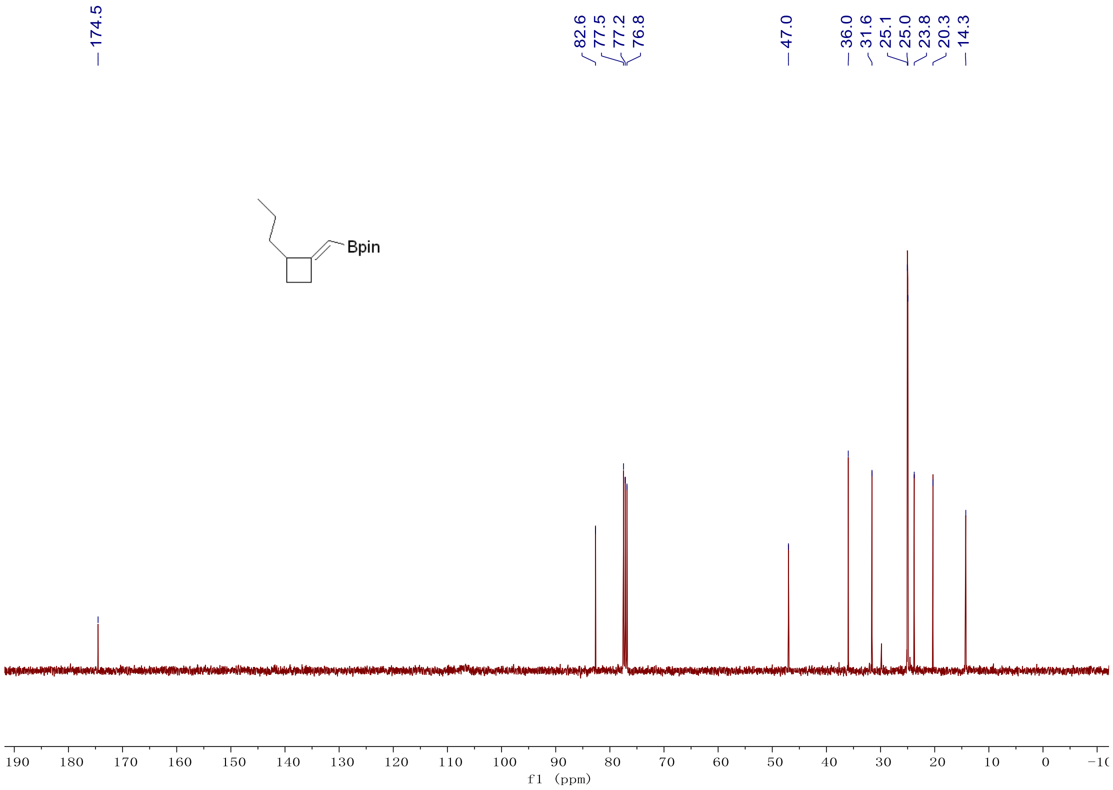

^{13}C NMR of compound 2u

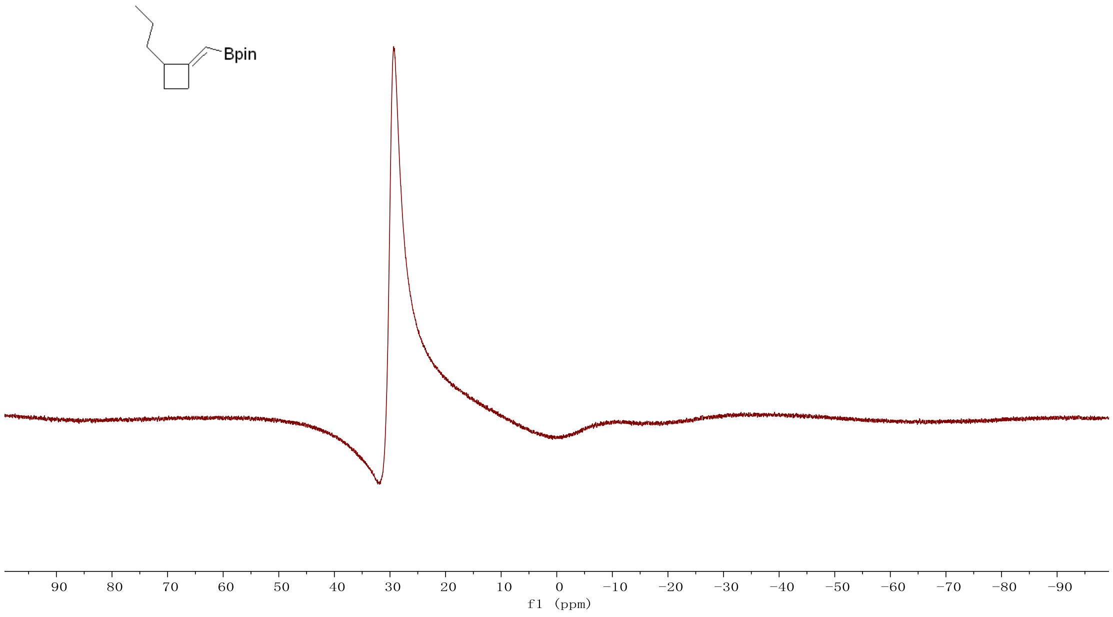

^{11}B NMR of compound 2u

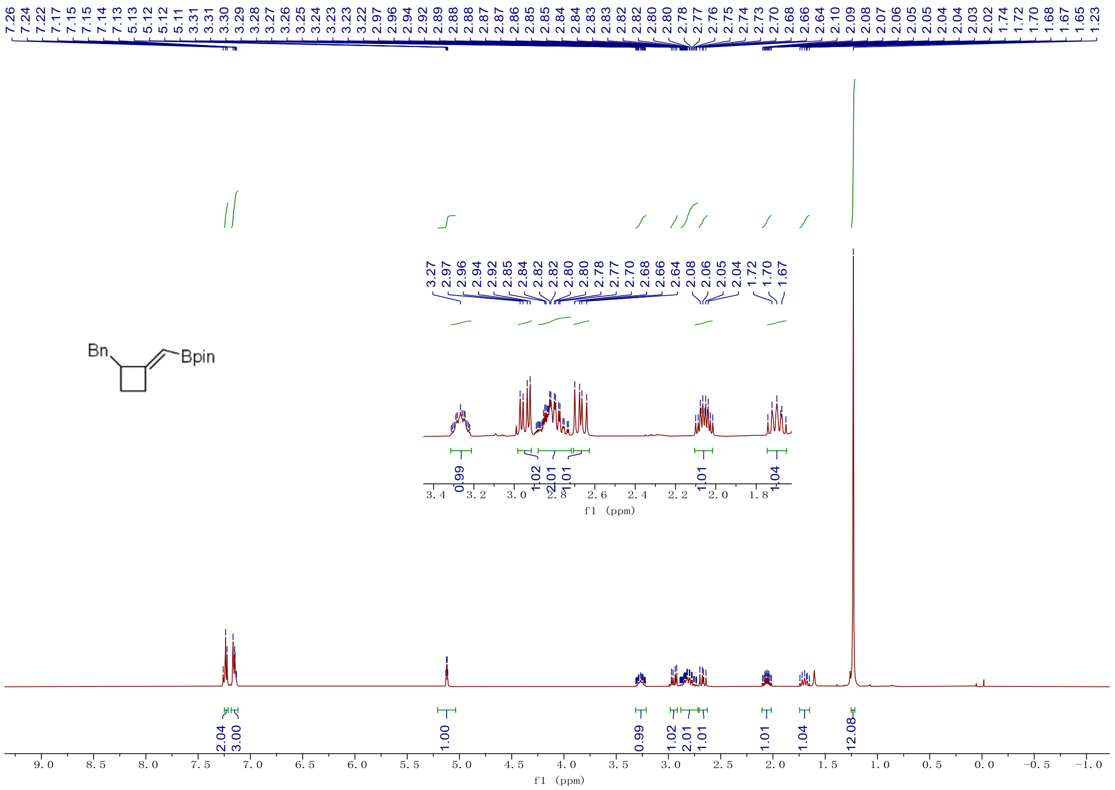

^{1}H NMR of compound 2v

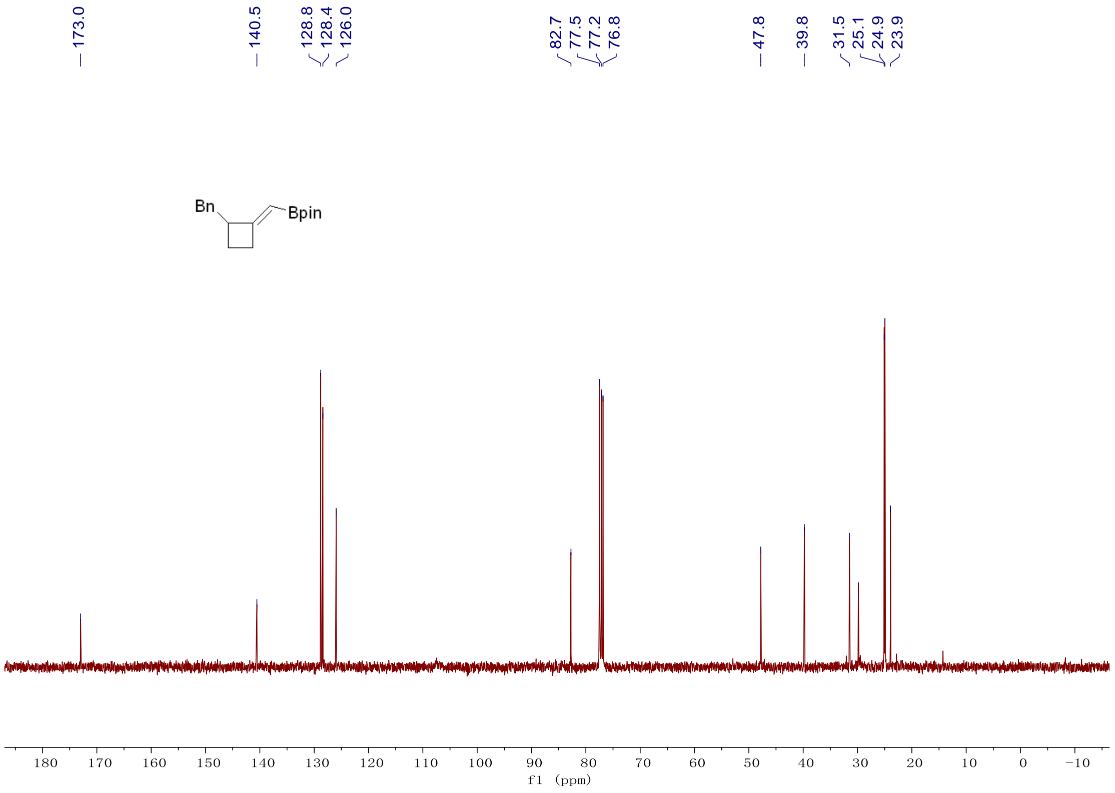

^{13}C NMR of compound 2v

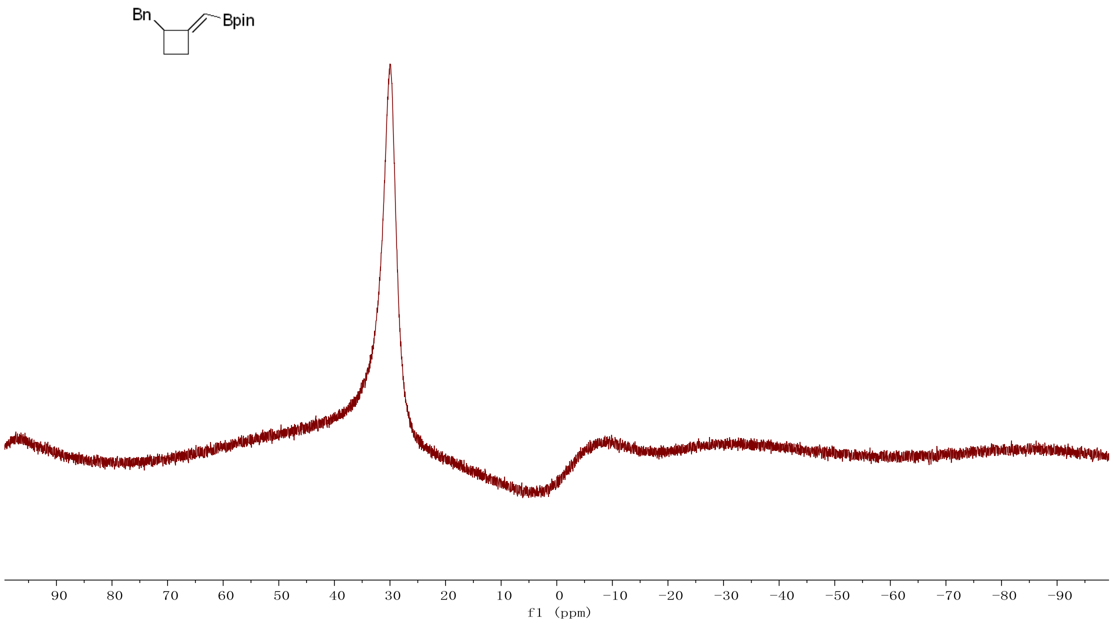

^{11}B NMR of compound 2v

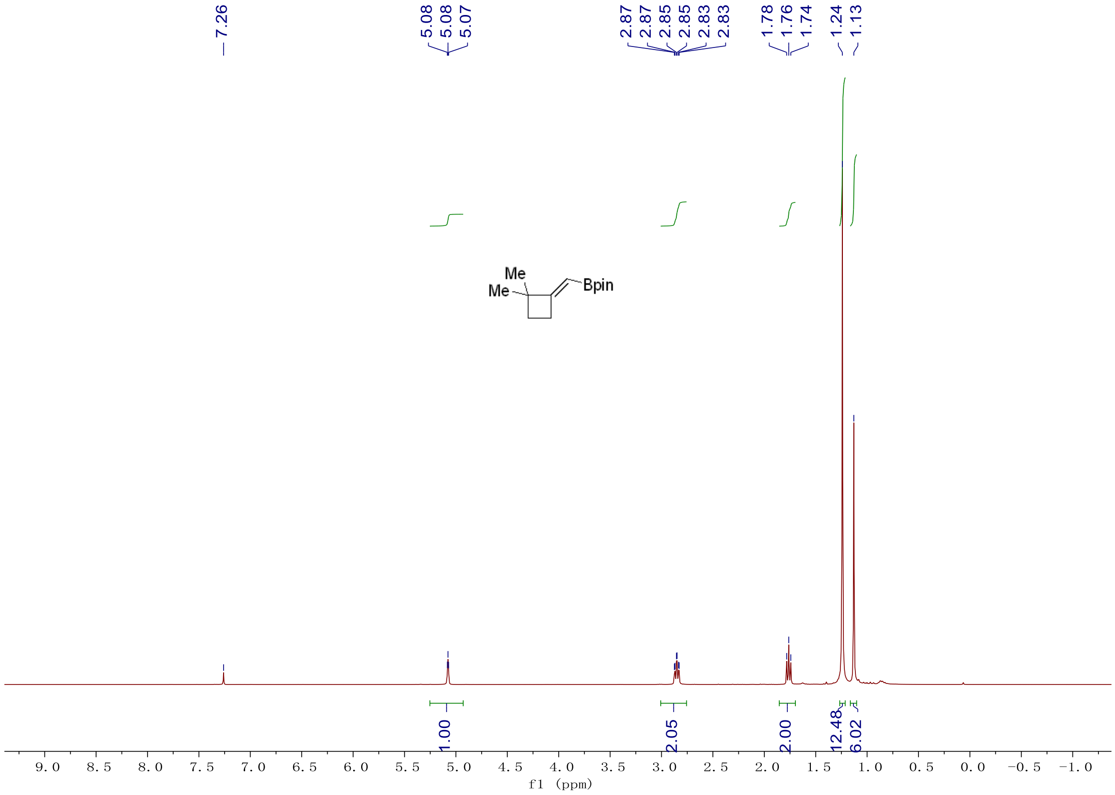

^{1}H NMR of compound 2w

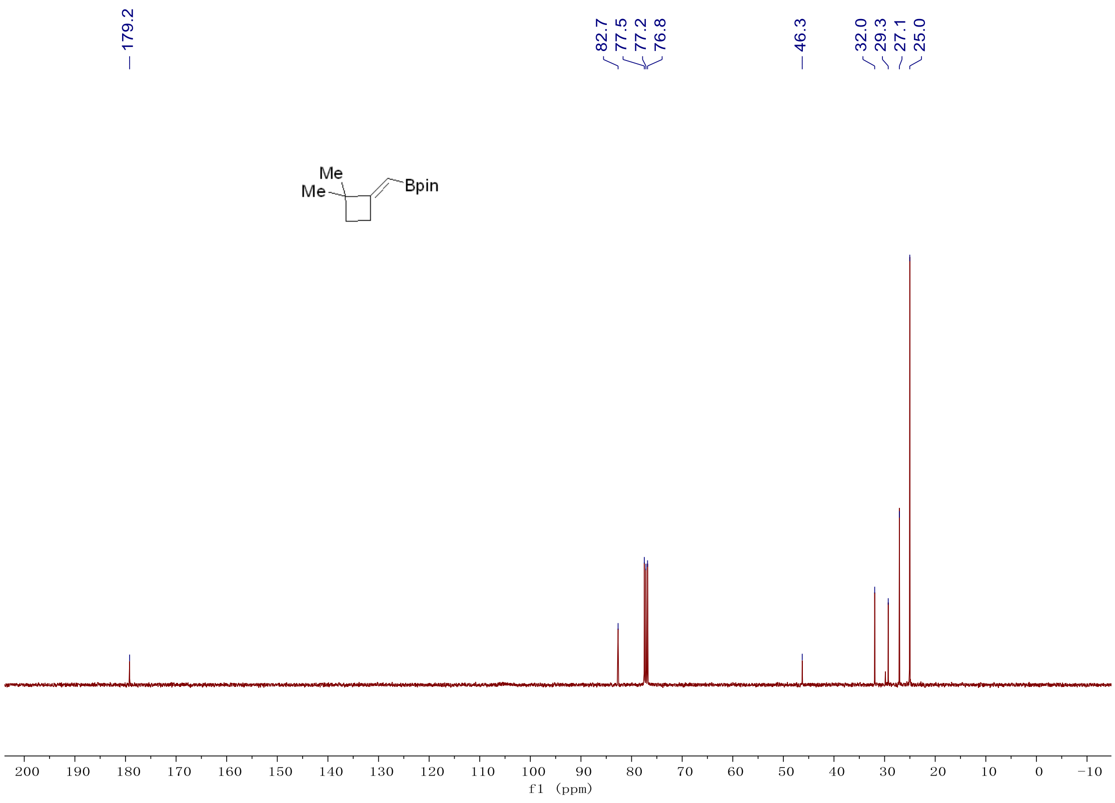

^{13}C NMR of compound 2w

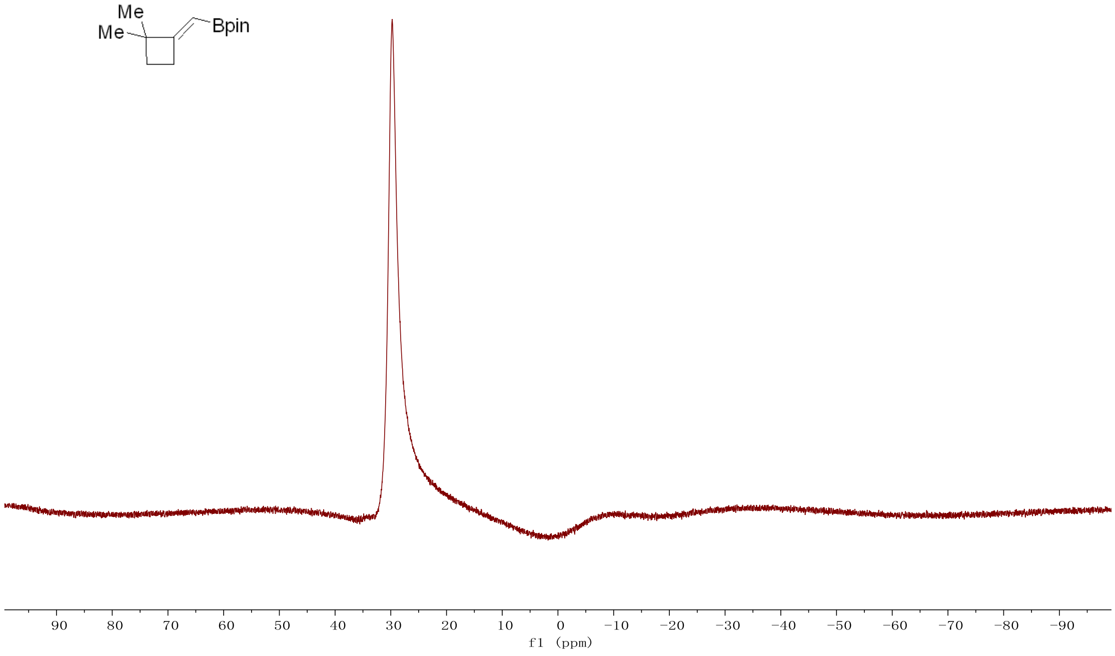

^{11}B NMR of compound 2w

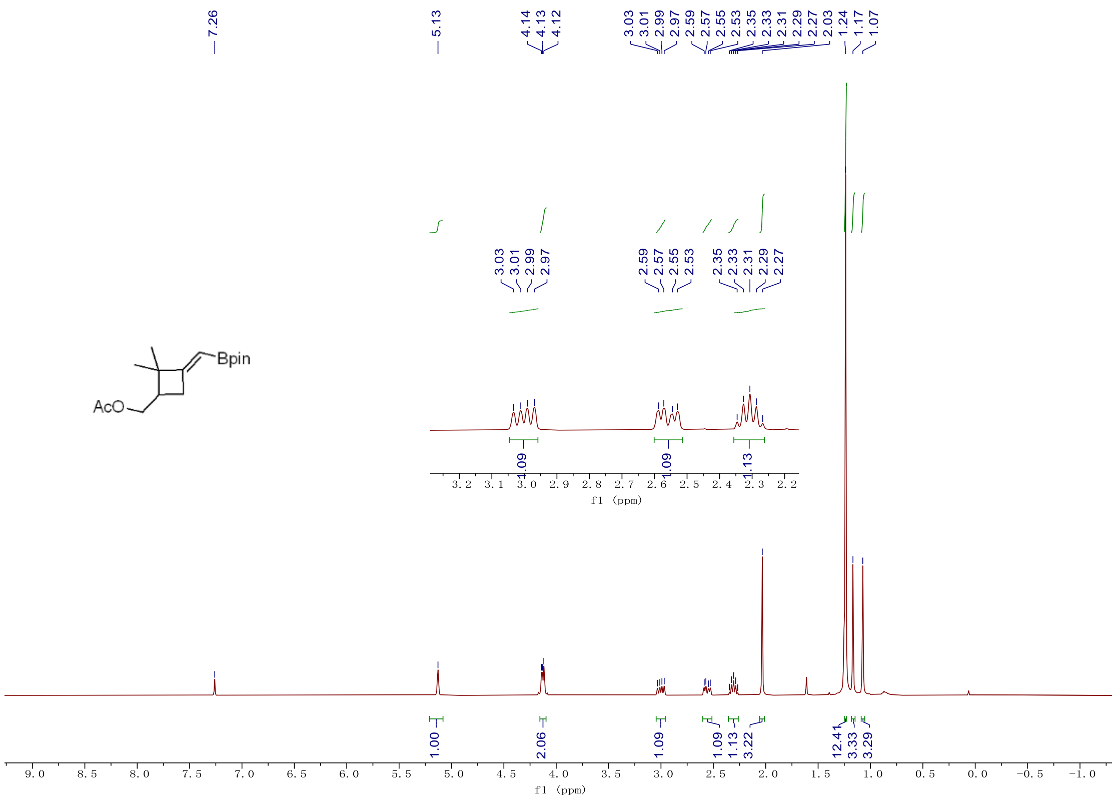

^{1}H NMR of compound 2x

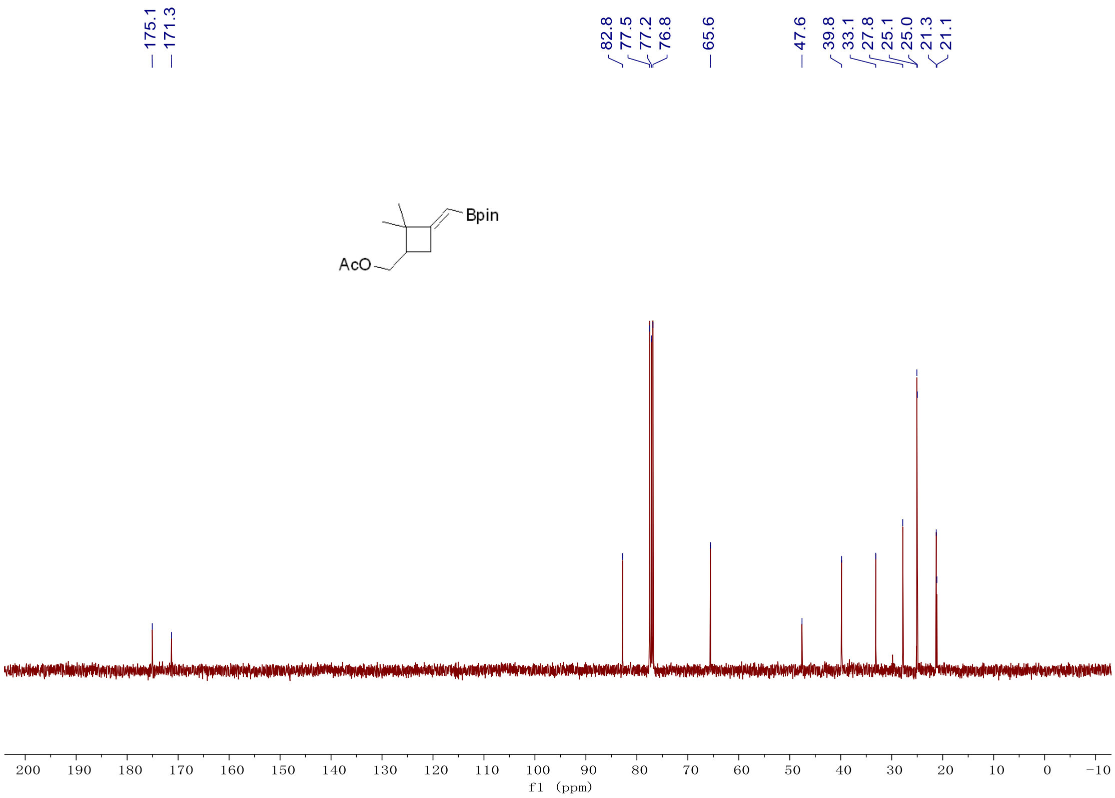

^{13}C NMR of compound 2x

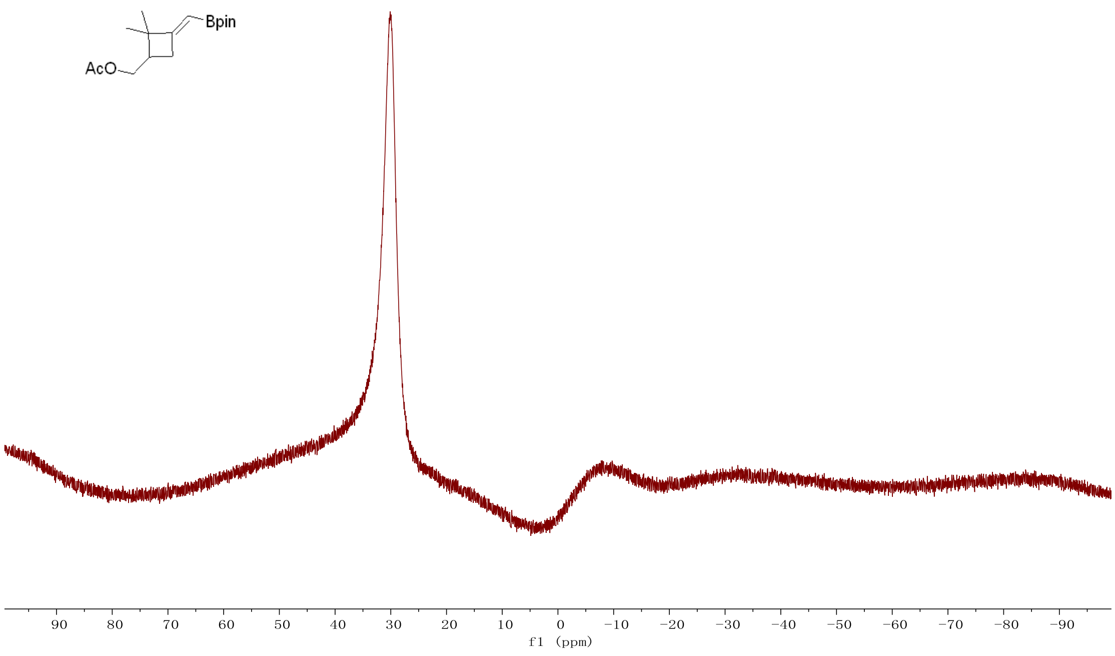

^{11}B NMR of compound 2x

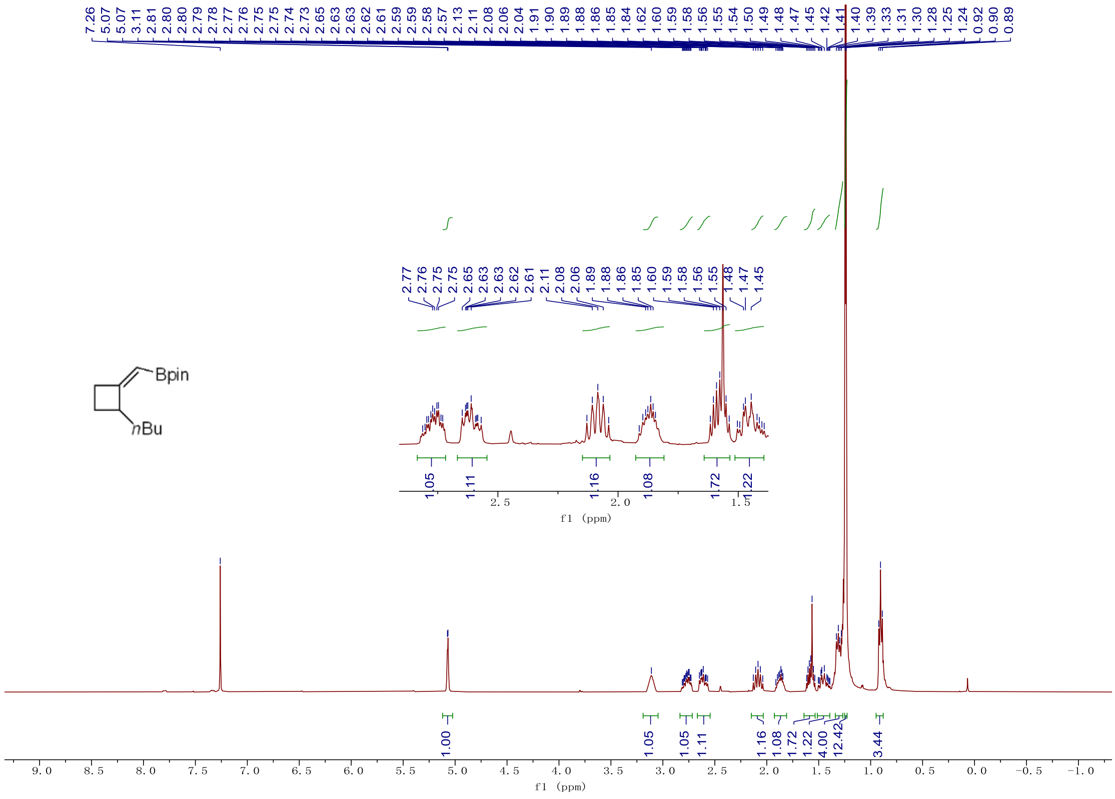

^{1}H NMR of compound 2y

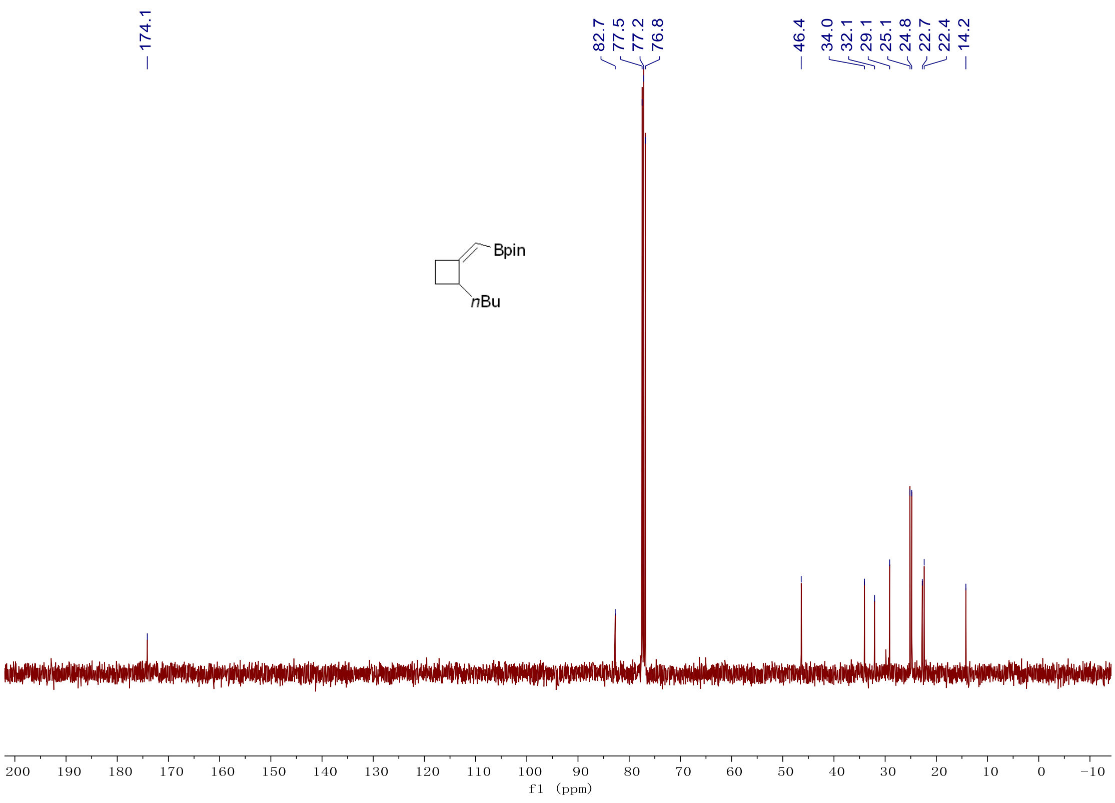

^{13}C NMR of compound 2y

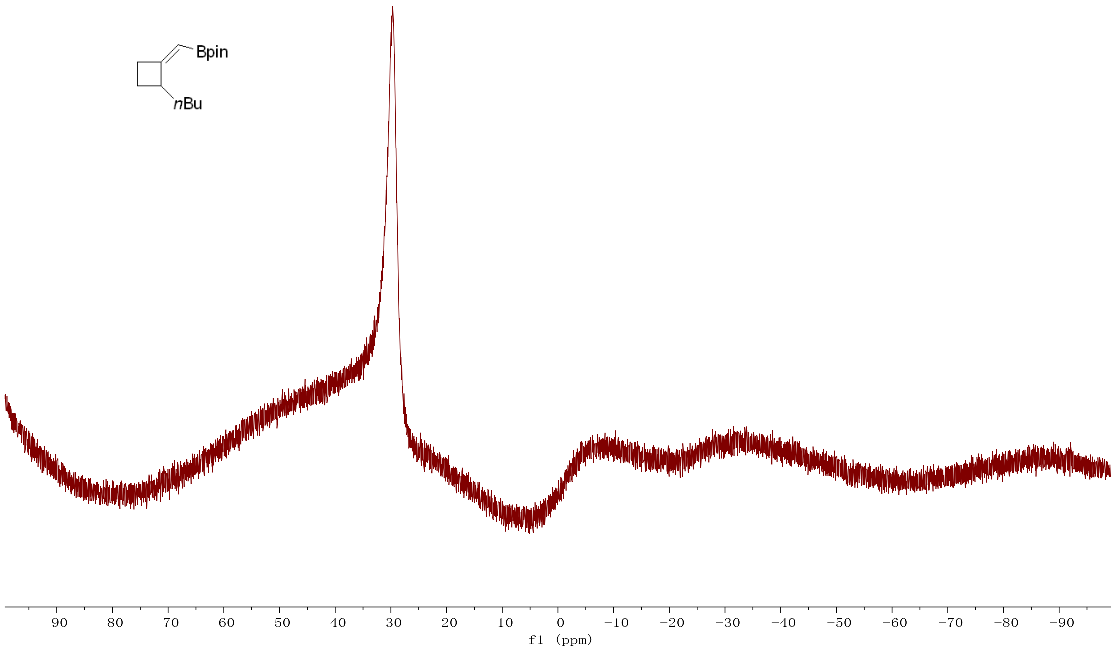

^{11}B NMR of compound 2y

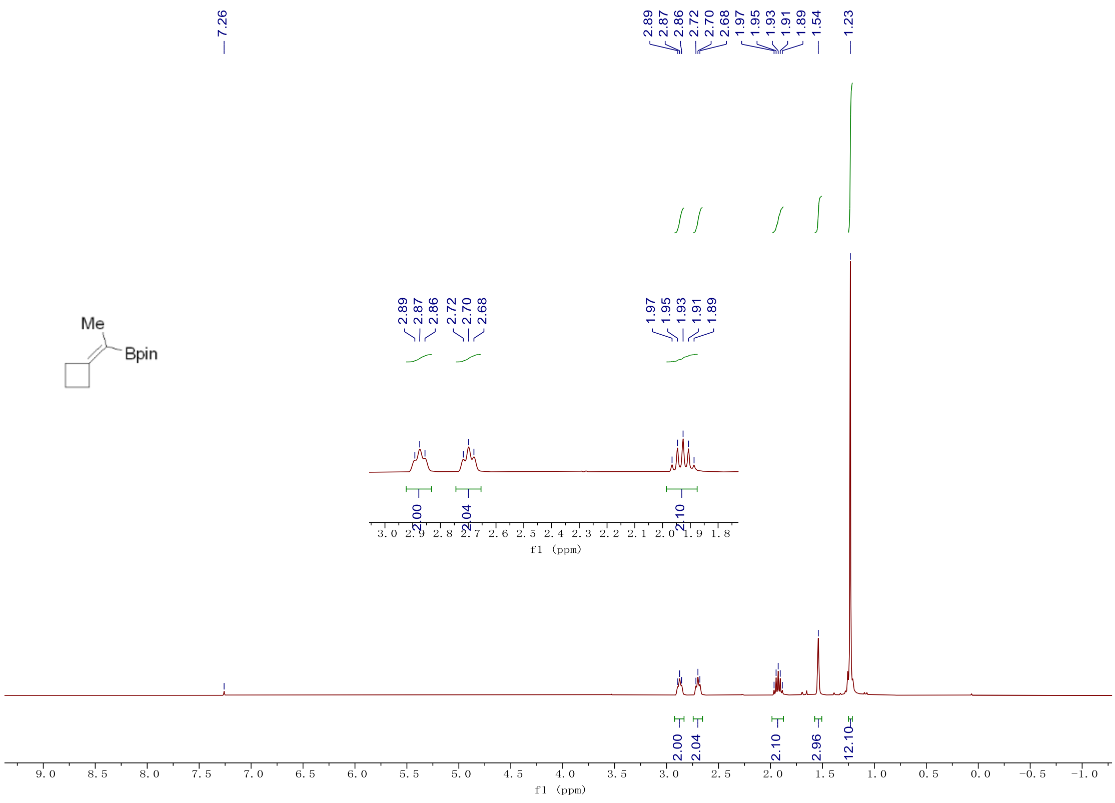

^{1}H NMR of compound 2z

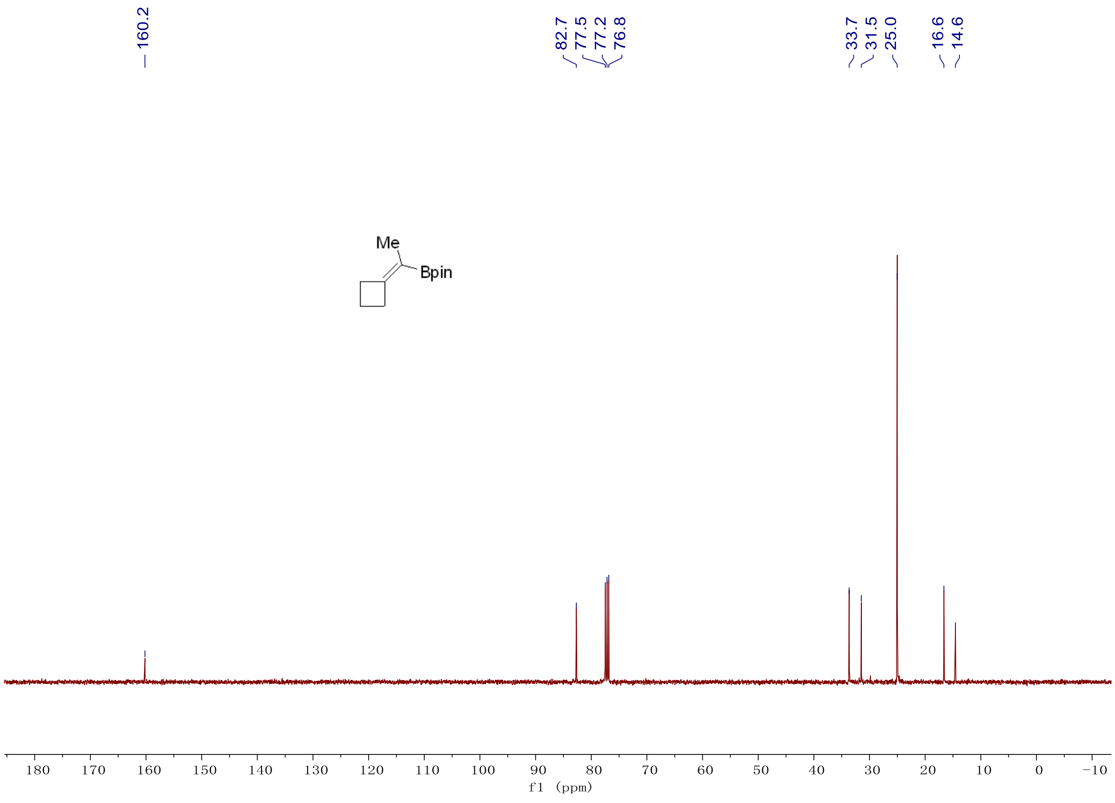

^{13}C NMR of compound 2z

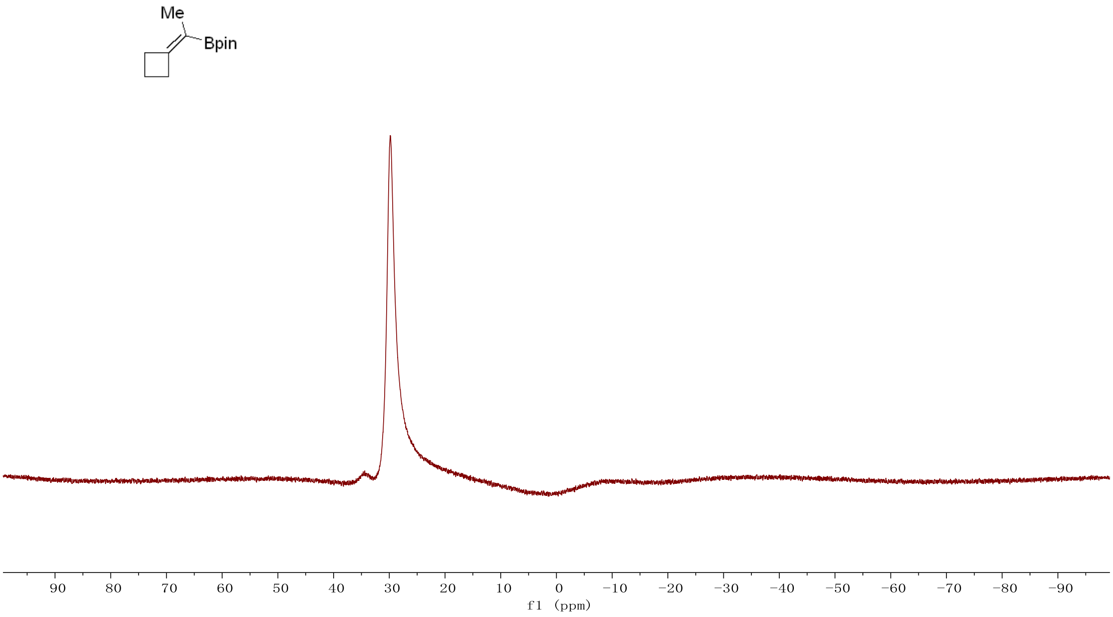

^{11}B NMR of compound 2z

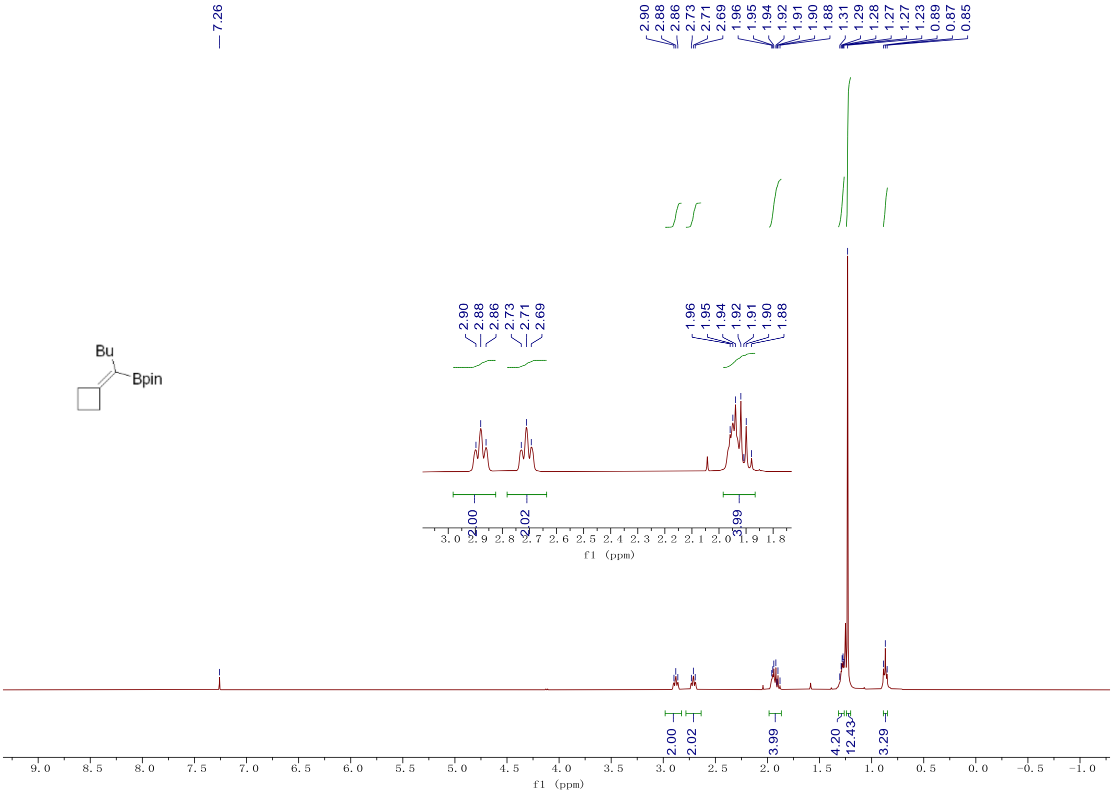

^{1}H NMR of compound 2aa

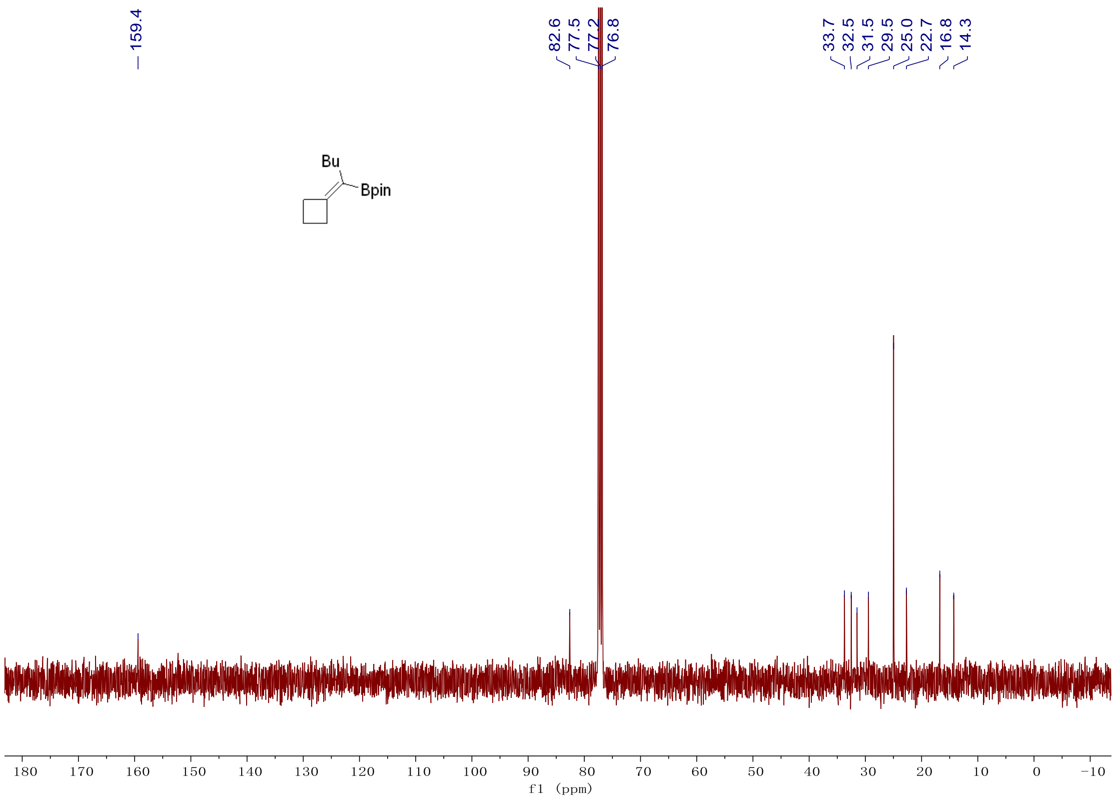

^{13}C NMR of compound 2aa

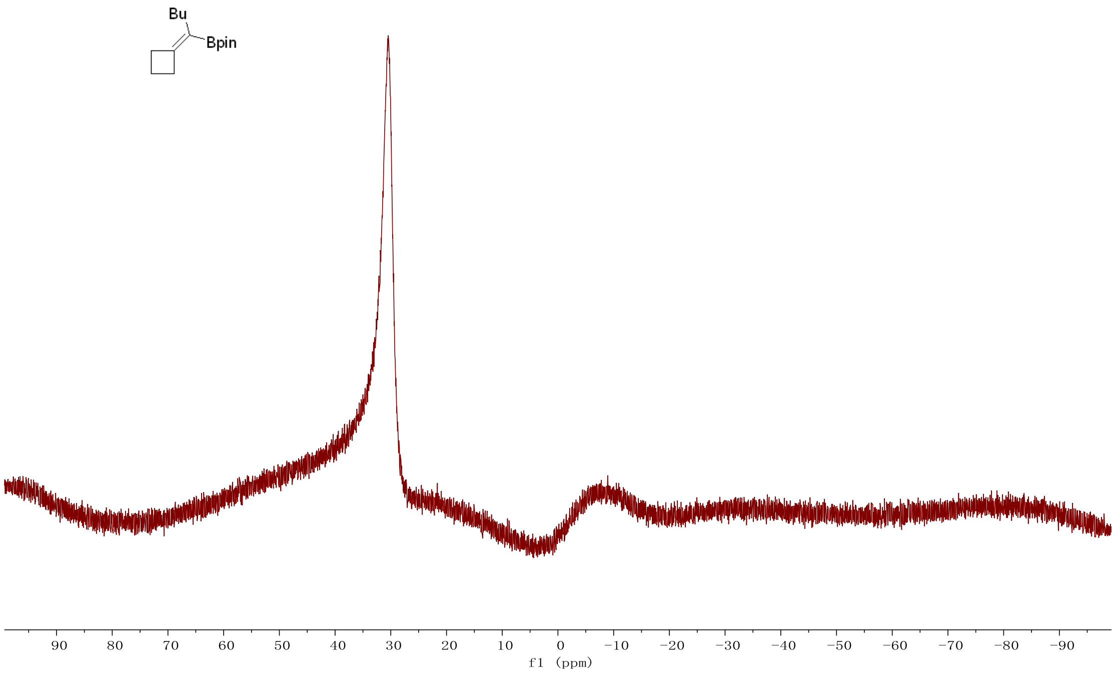

^{11}B NMR of compound 2aa

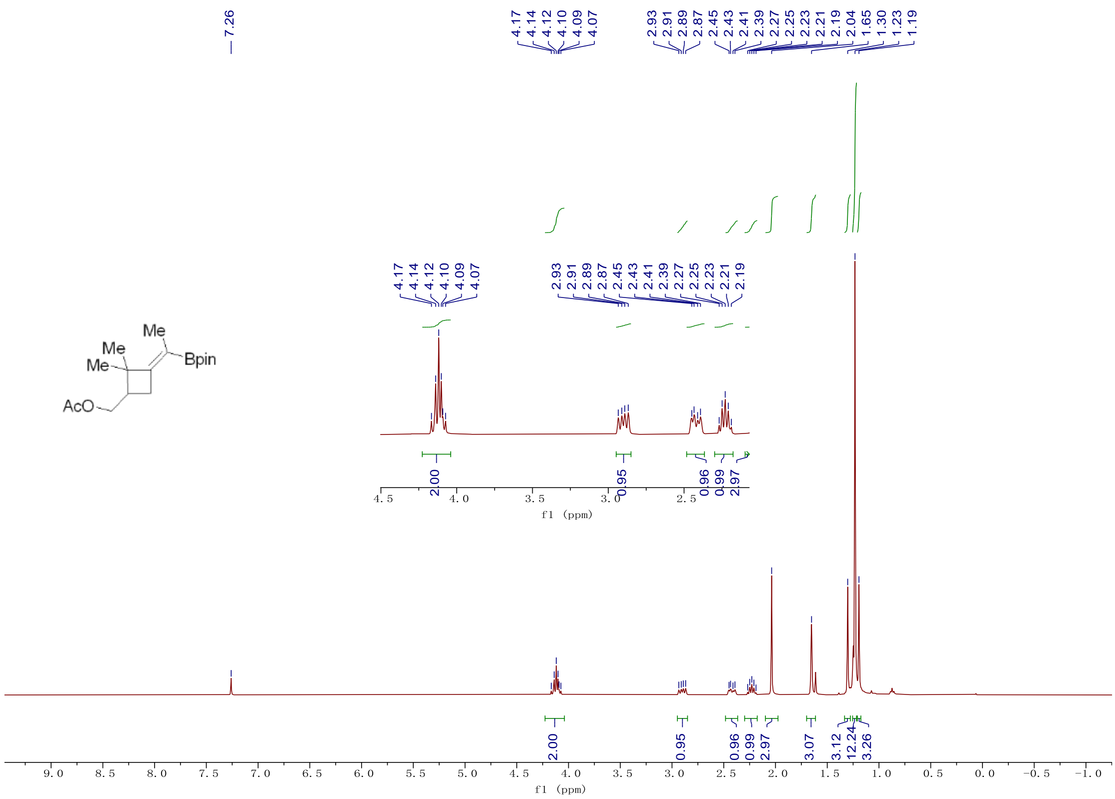

^{1}H NMR of compound 2ab

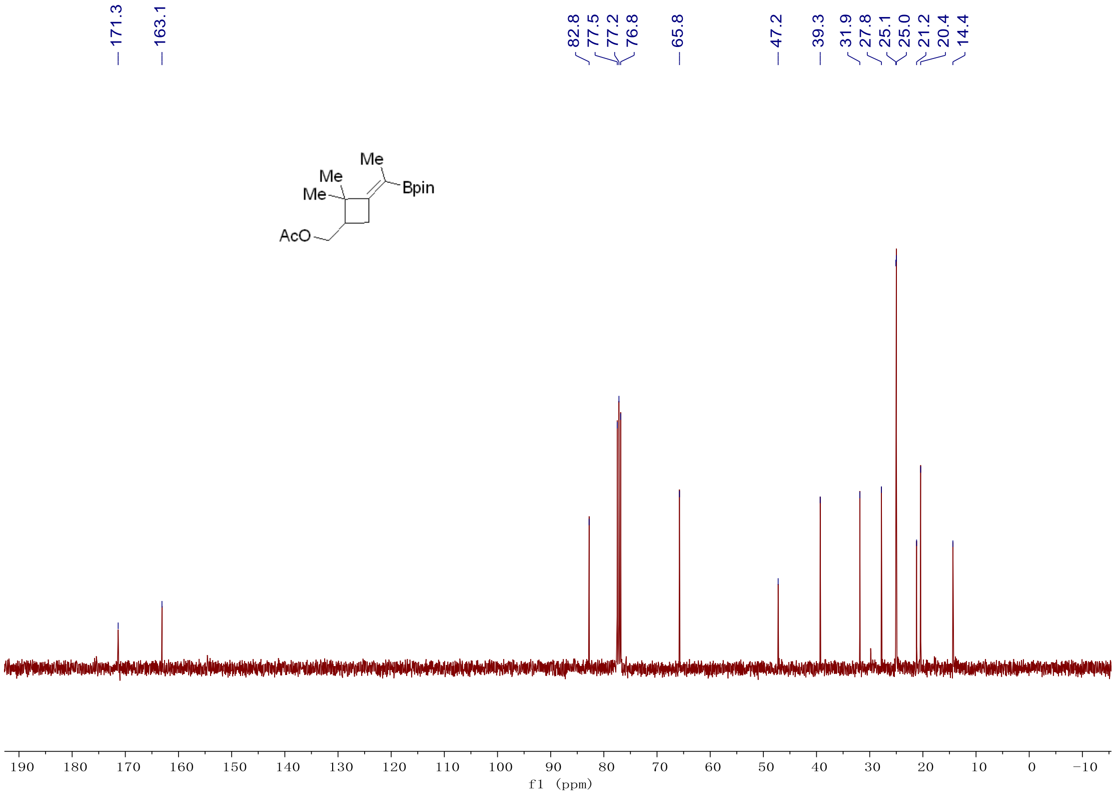

^{13}C NMR of compound 2ab

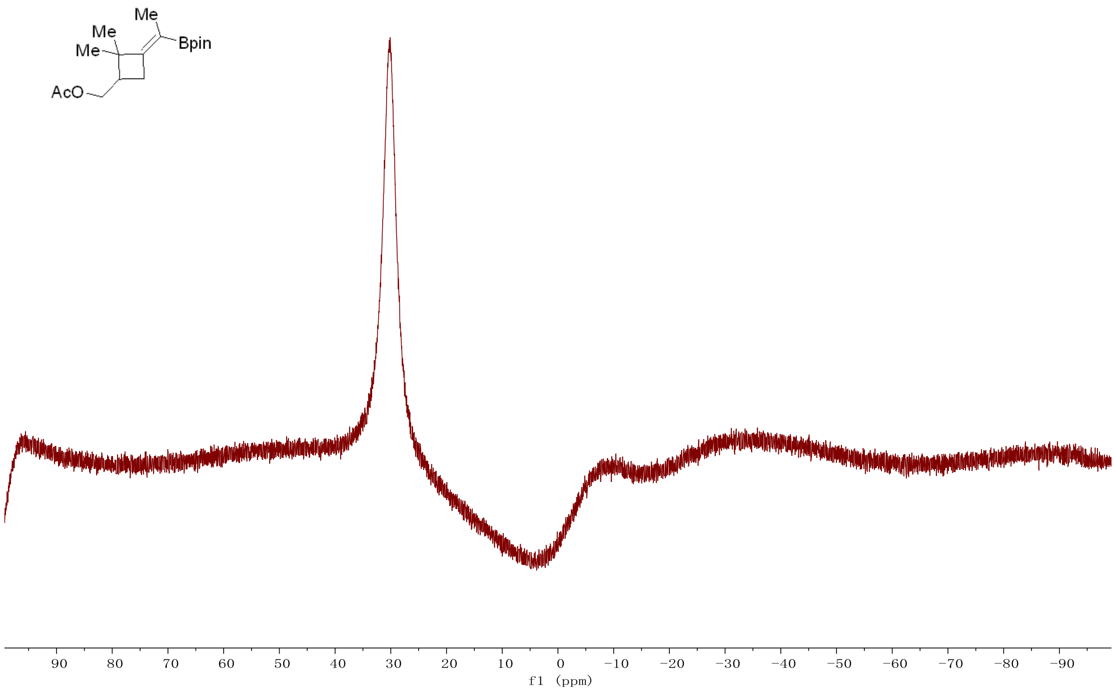

^{11}B NMR of compound 2ab

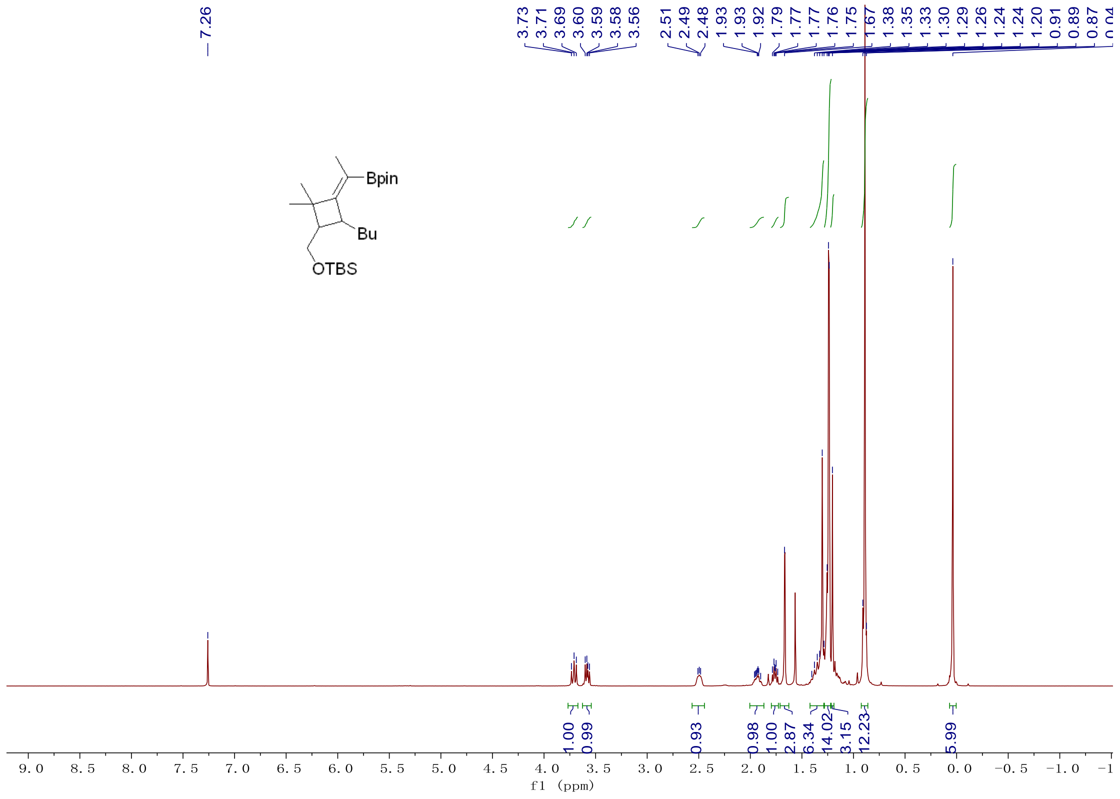

^{1}H NMR of compound 2ac

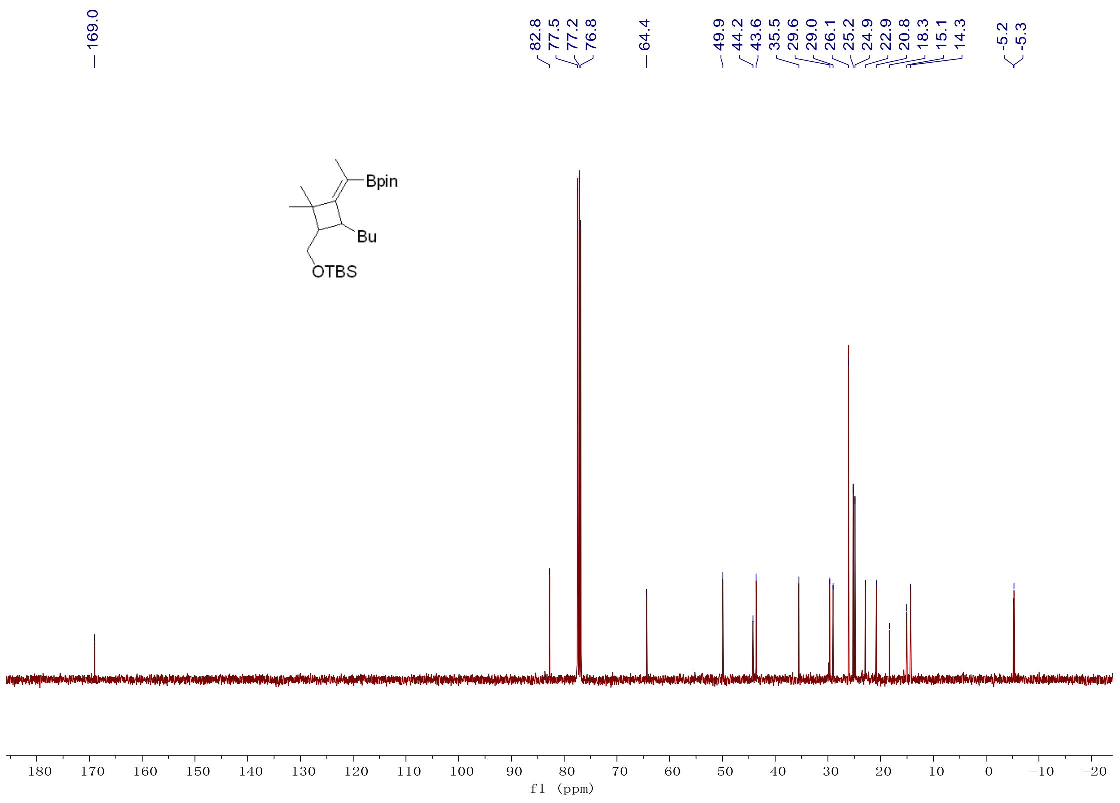

^{13}C NMR of compound 2ac

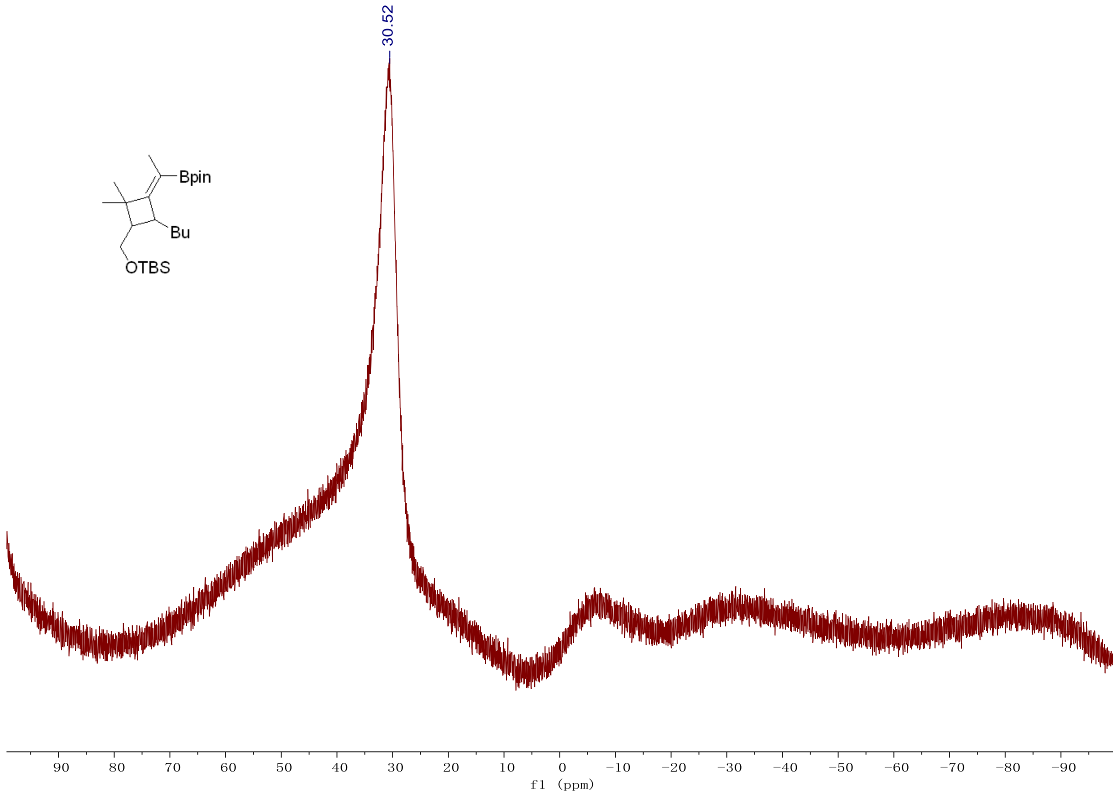

^{11}B NMR of compound 2ac

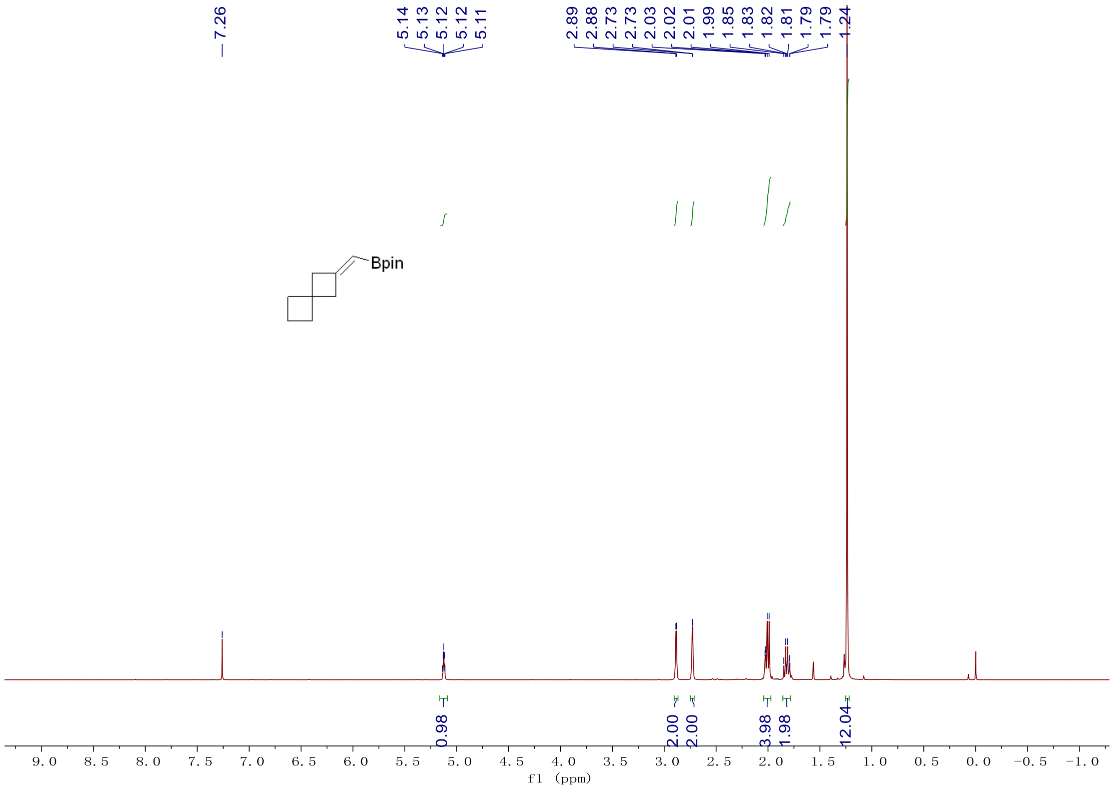

^{1}H NMR of compound 2ad

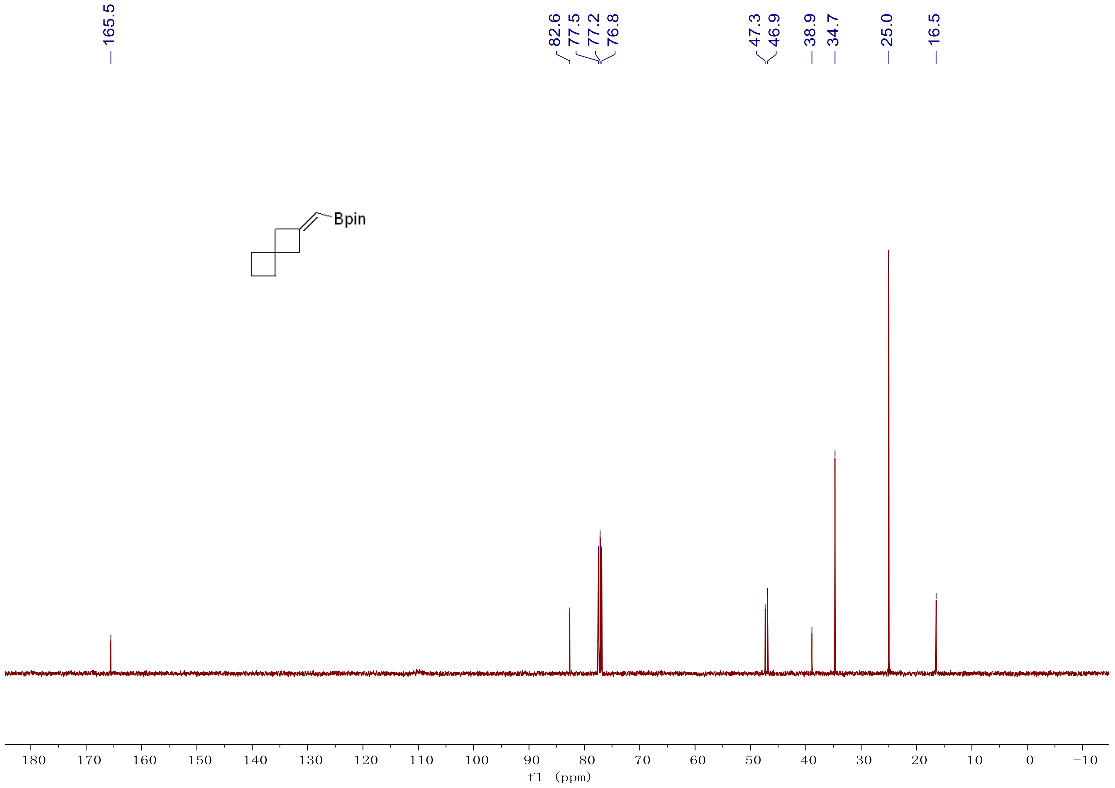

^{13}C NMR of compound 2ad

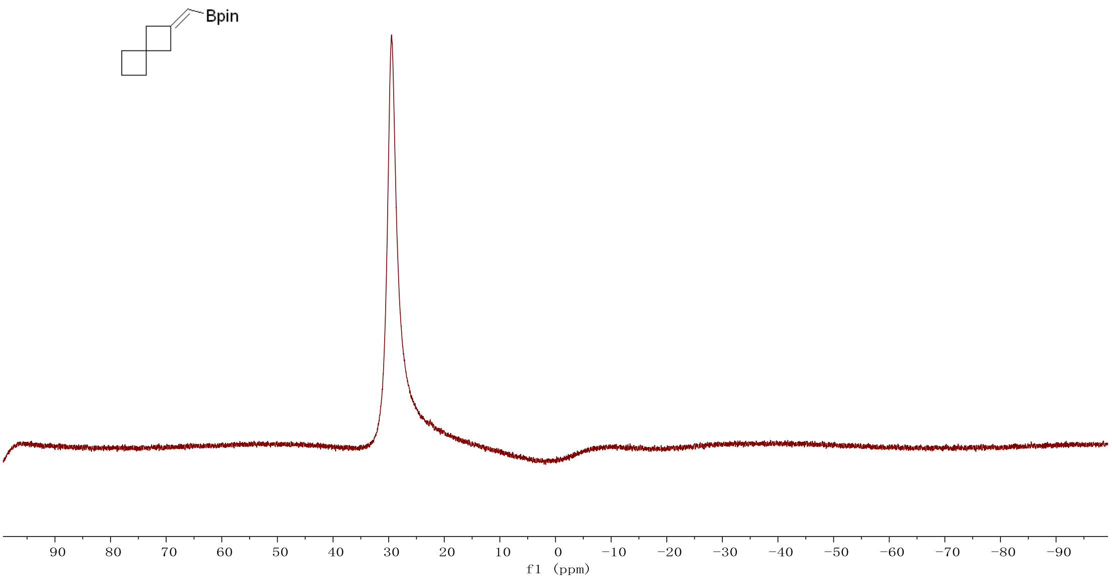

^{11}B NMR of compound 2ad

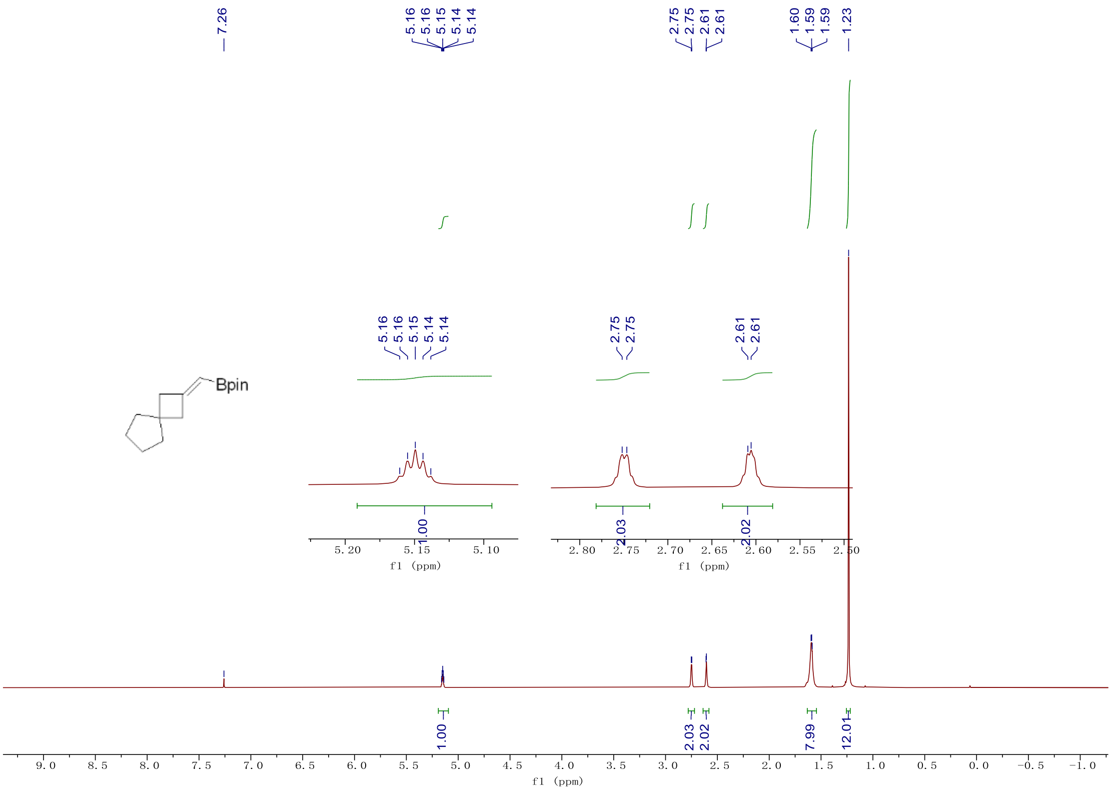

^{1}H NMR of compound 2ae

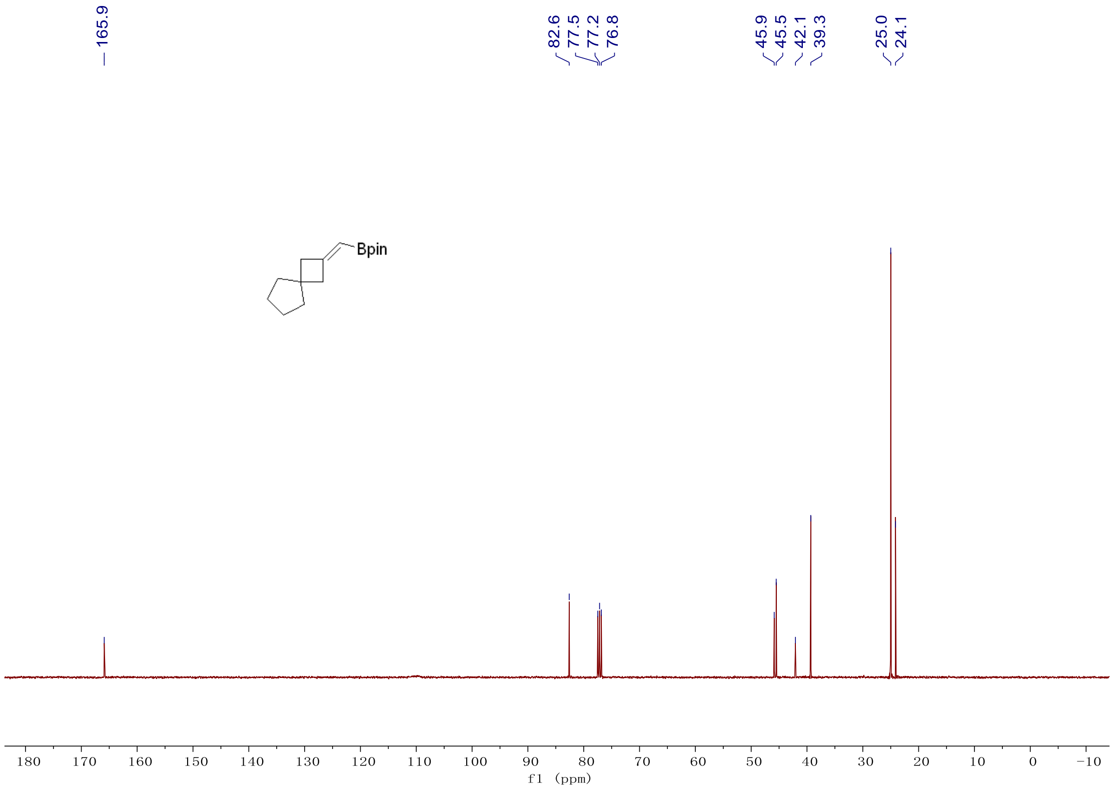

^{13}C NMR of compound 2ae

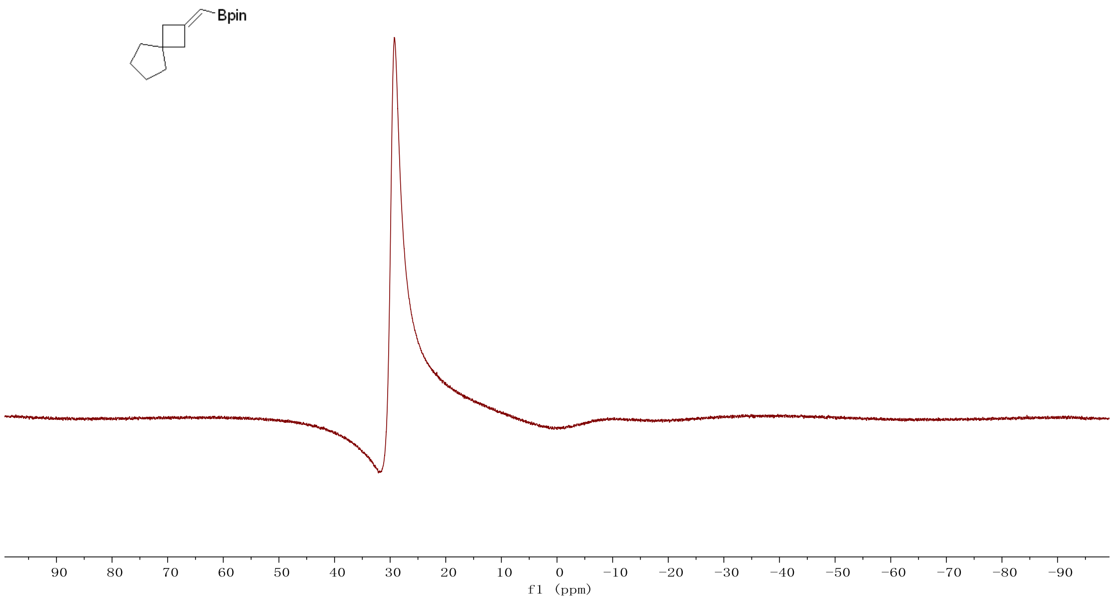

^{11}B NMR of compound 2ae

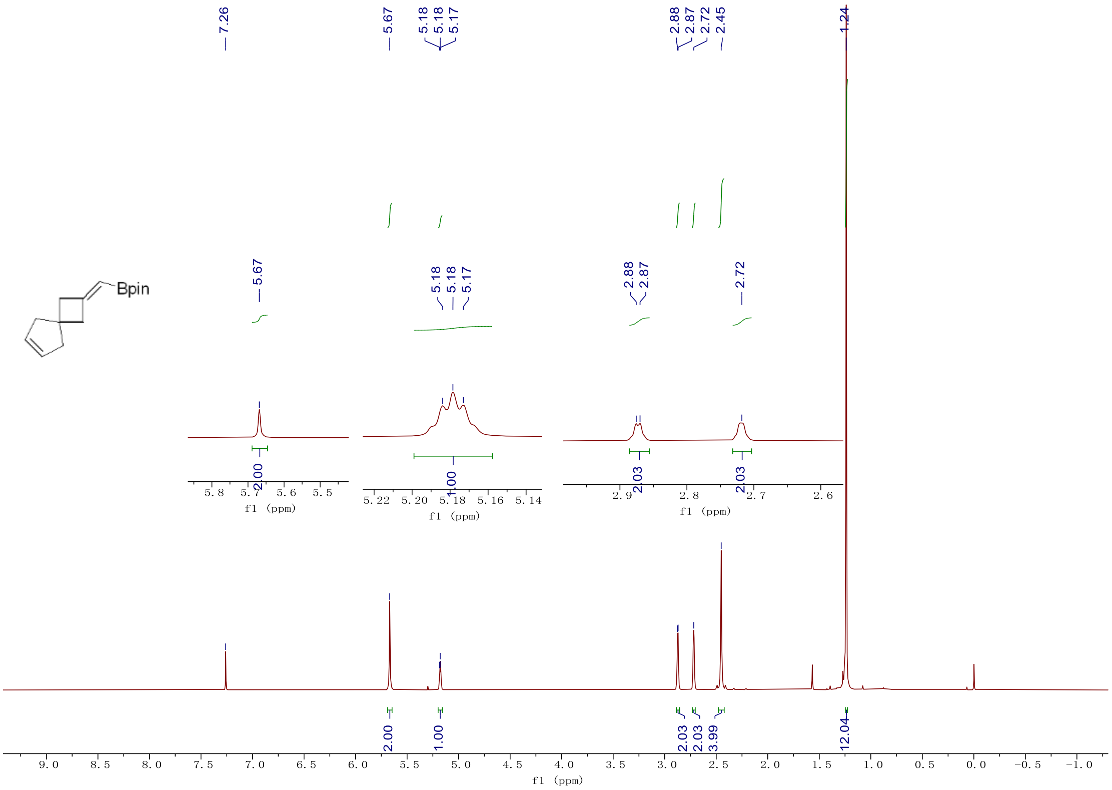

^{1}H NMR of compound 2af

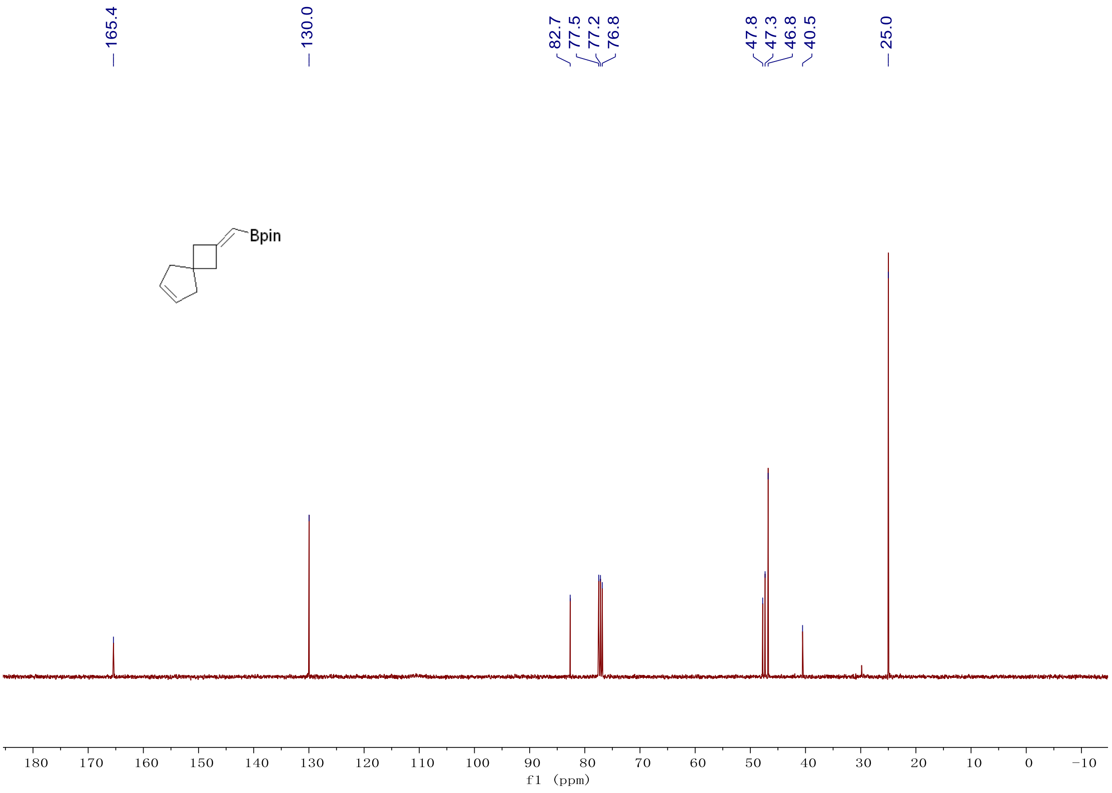

^{13}C NMR of compound 2af

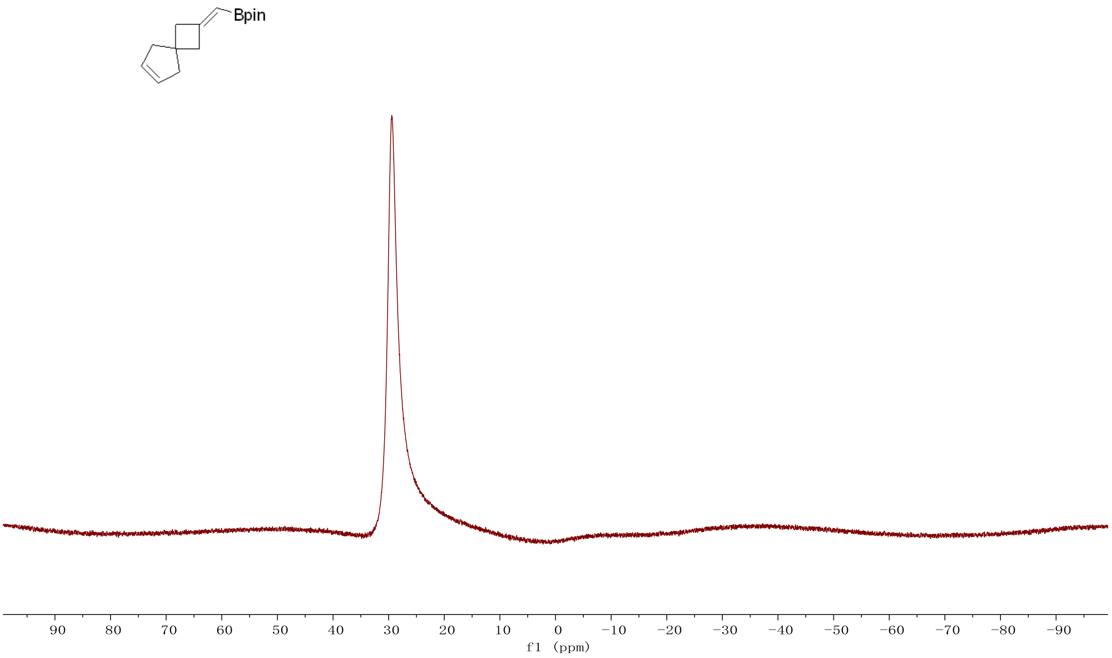

^{11}B NMR of compound 2af

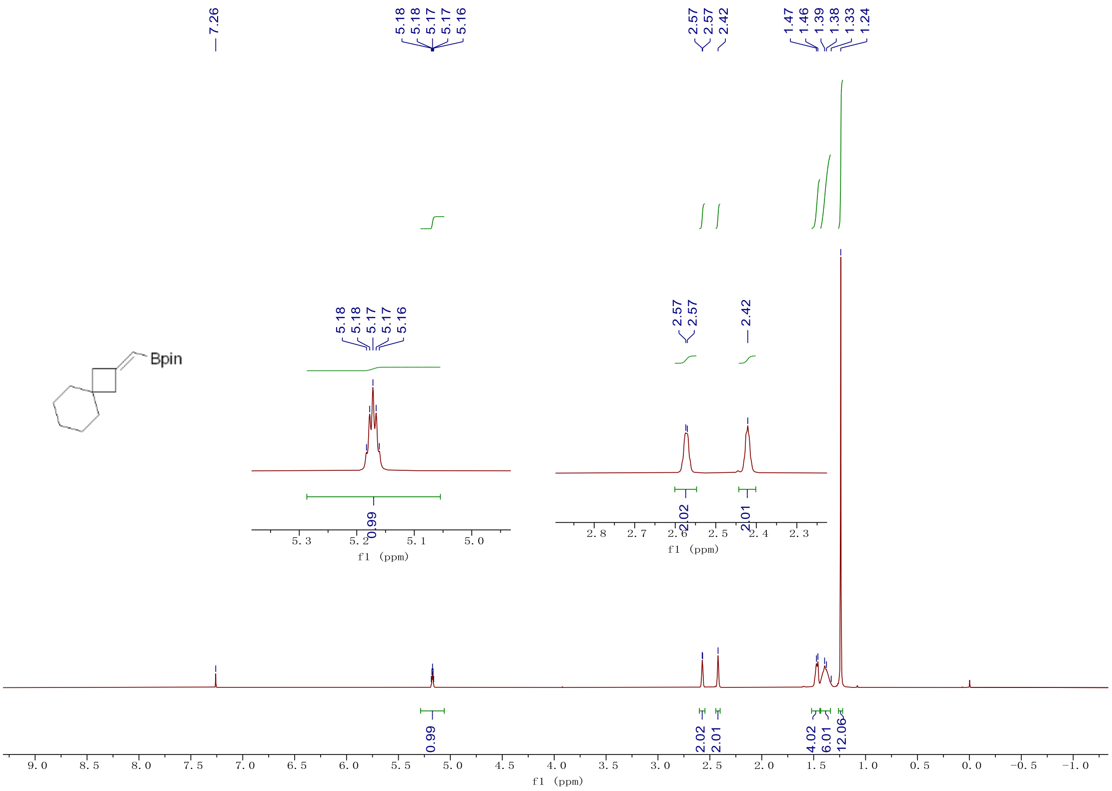

^{1}H NMR of compound 2ag

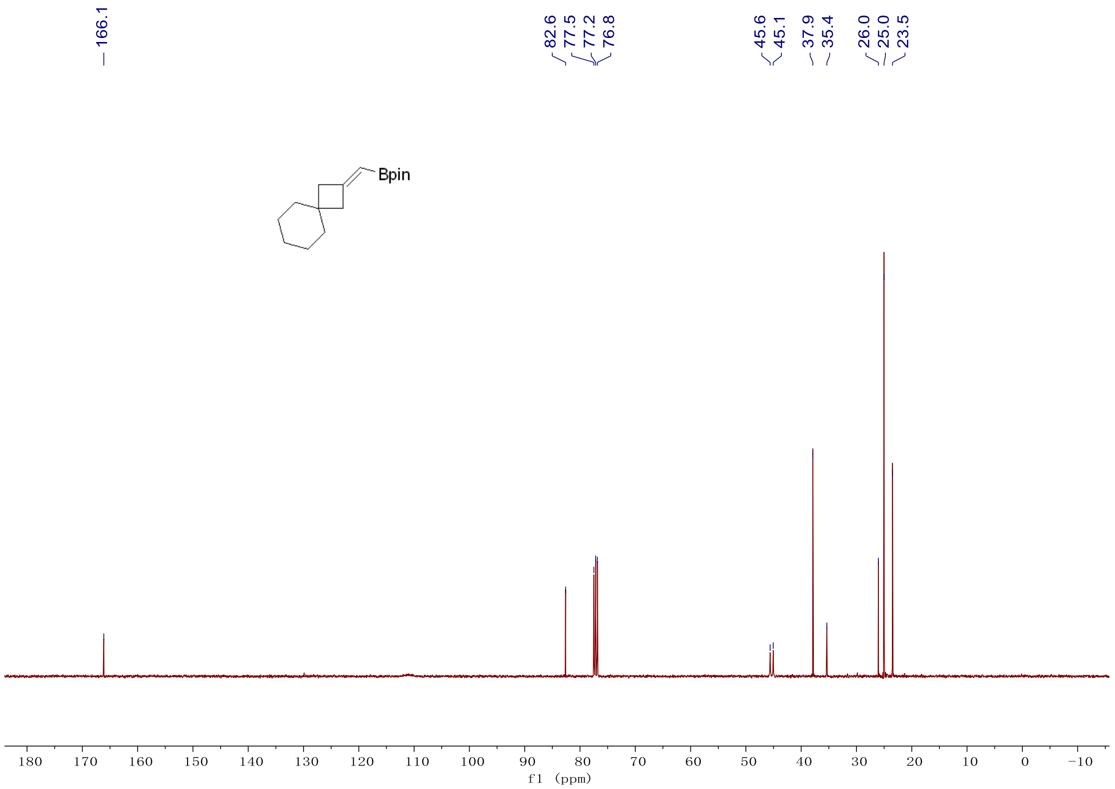

^{13}C NMR of compound 2ag

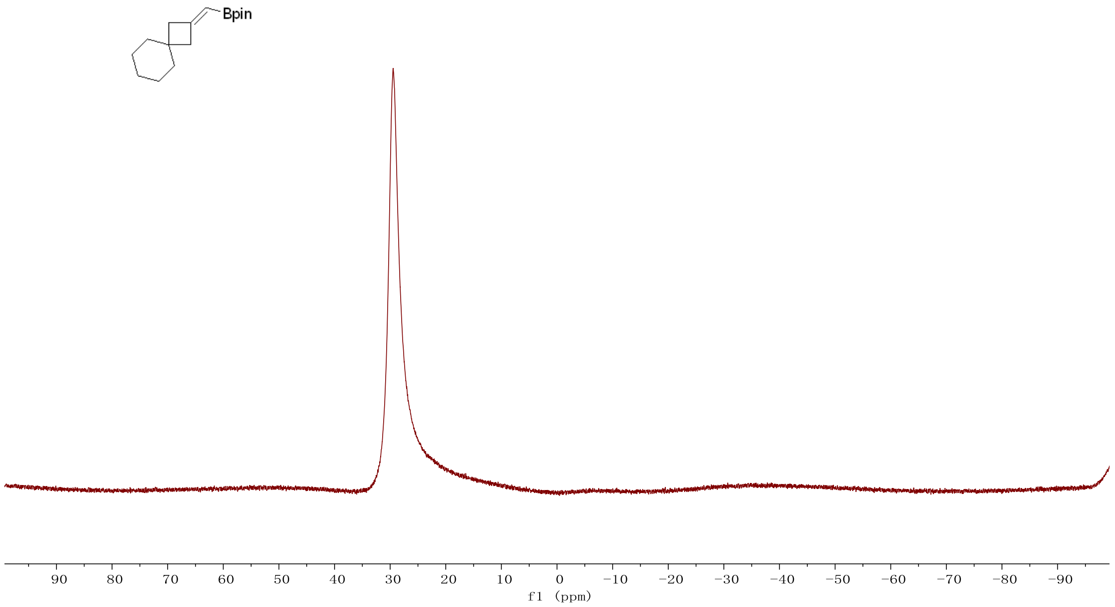

^{11}B NMR of compound 2ag

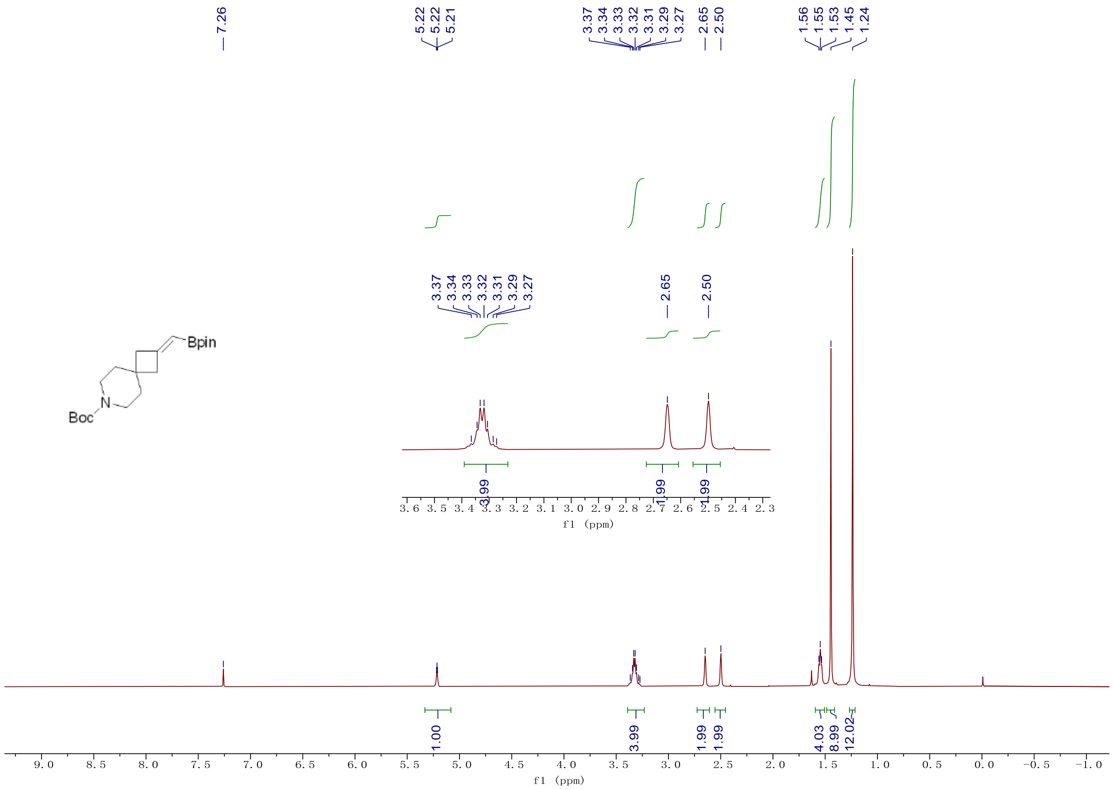

^{1}H NMR of compound 2ah

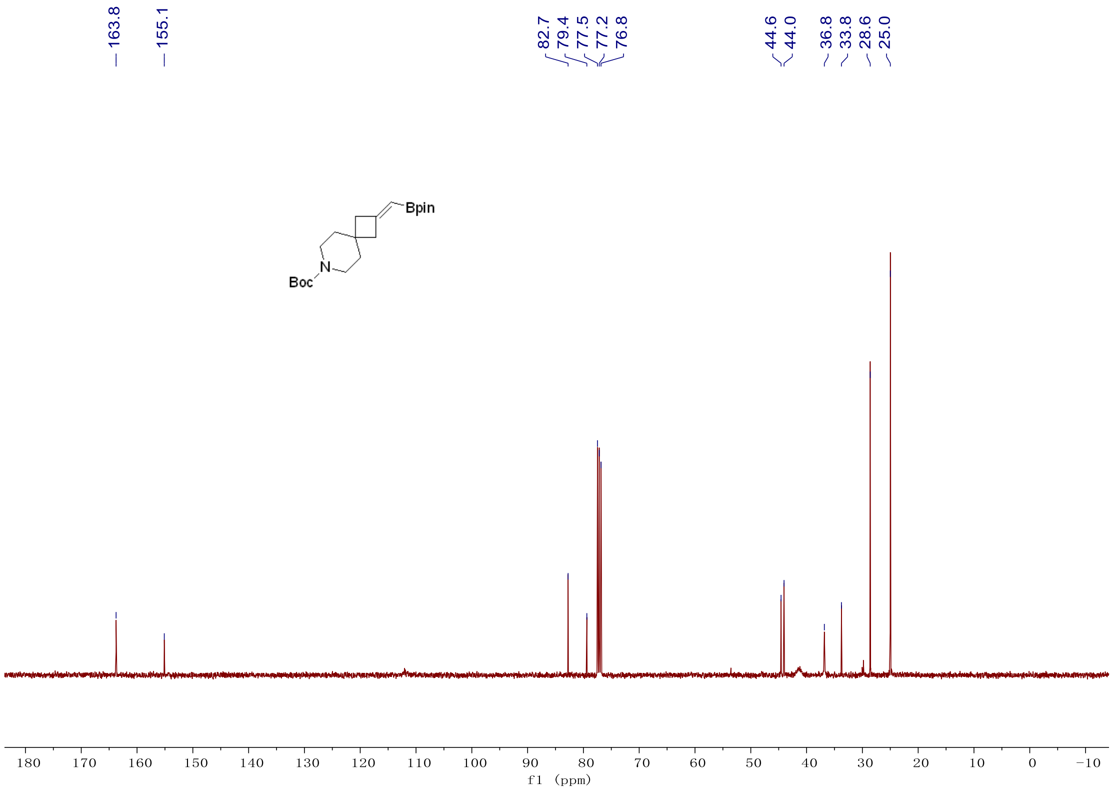

^{13}C NMR of compound 2ah

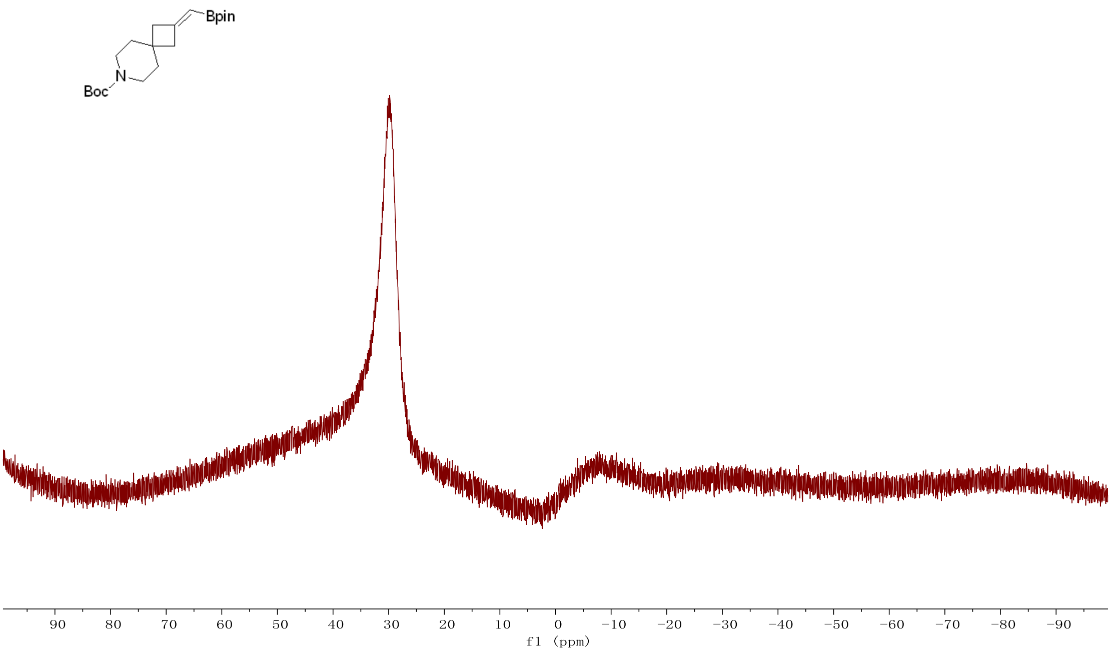

^{11}B NMR of compound 2ah

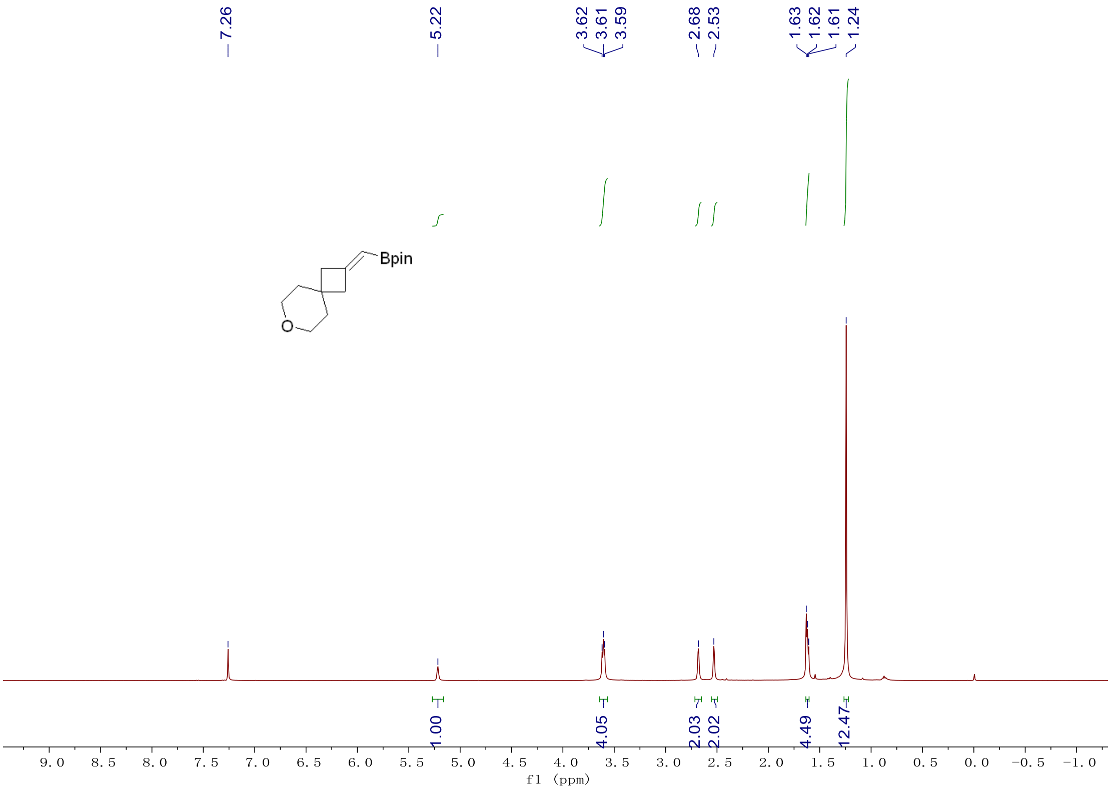

^{1}H NMR of compound 2ai

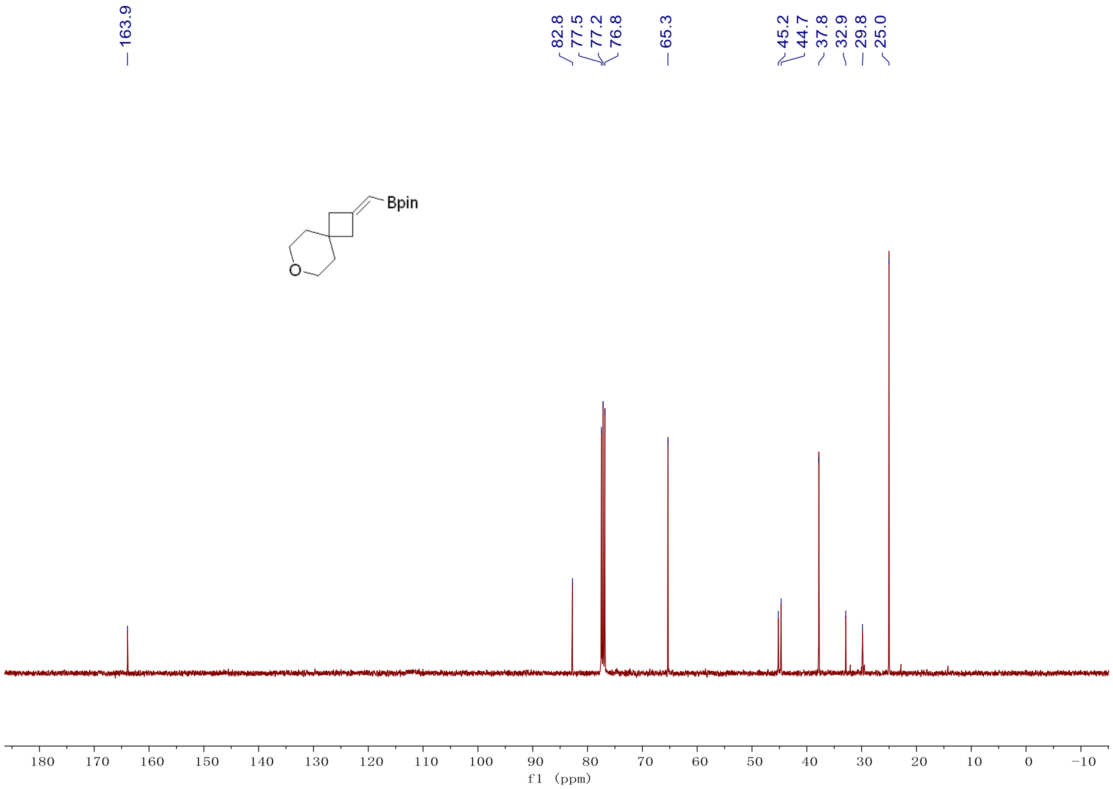

^{13}C NMR of compound 2ai

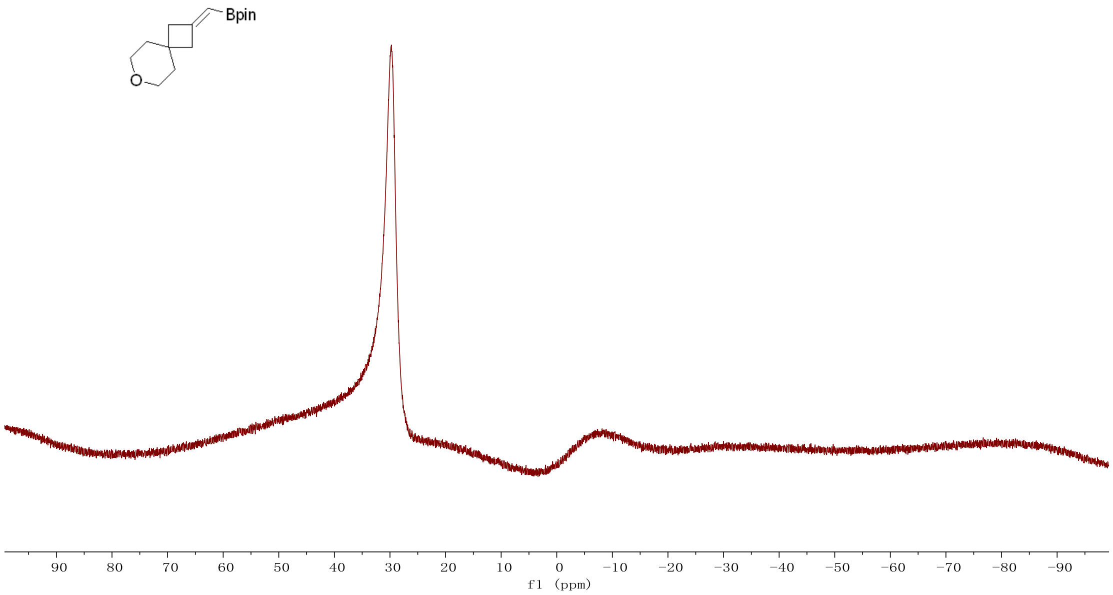

^{11}B NMR of compound 2ai

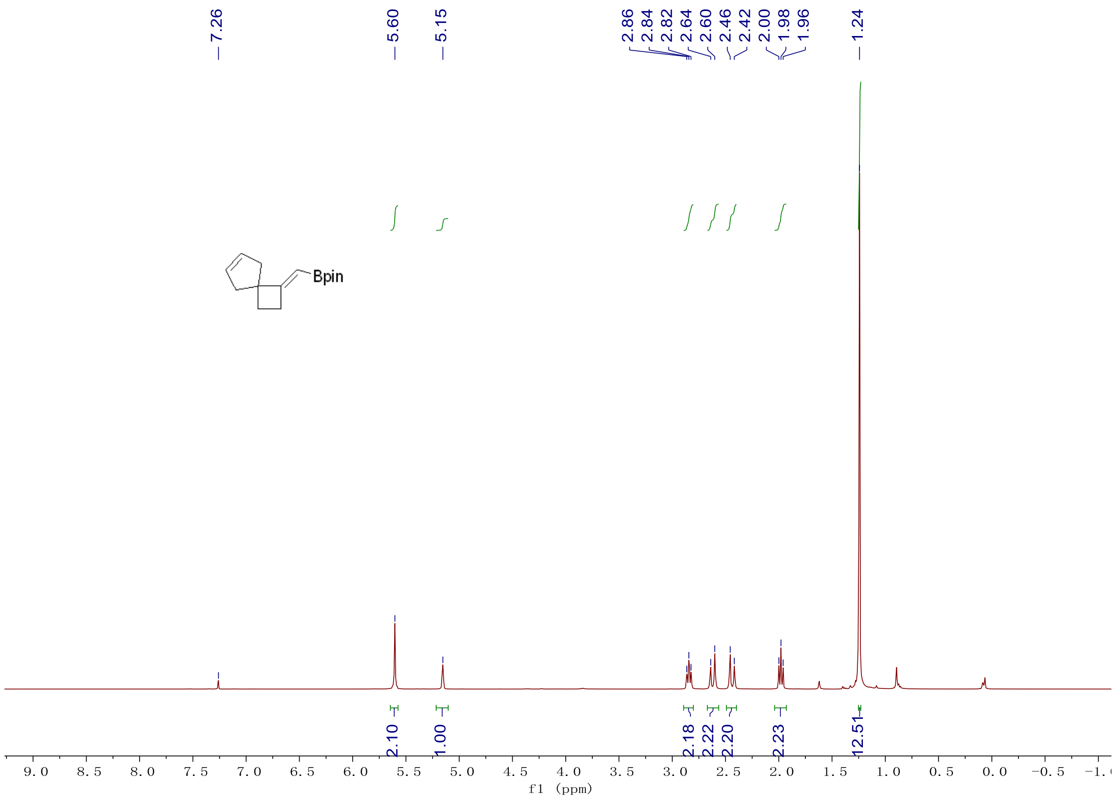

^{1}H NMR of compound 2aj

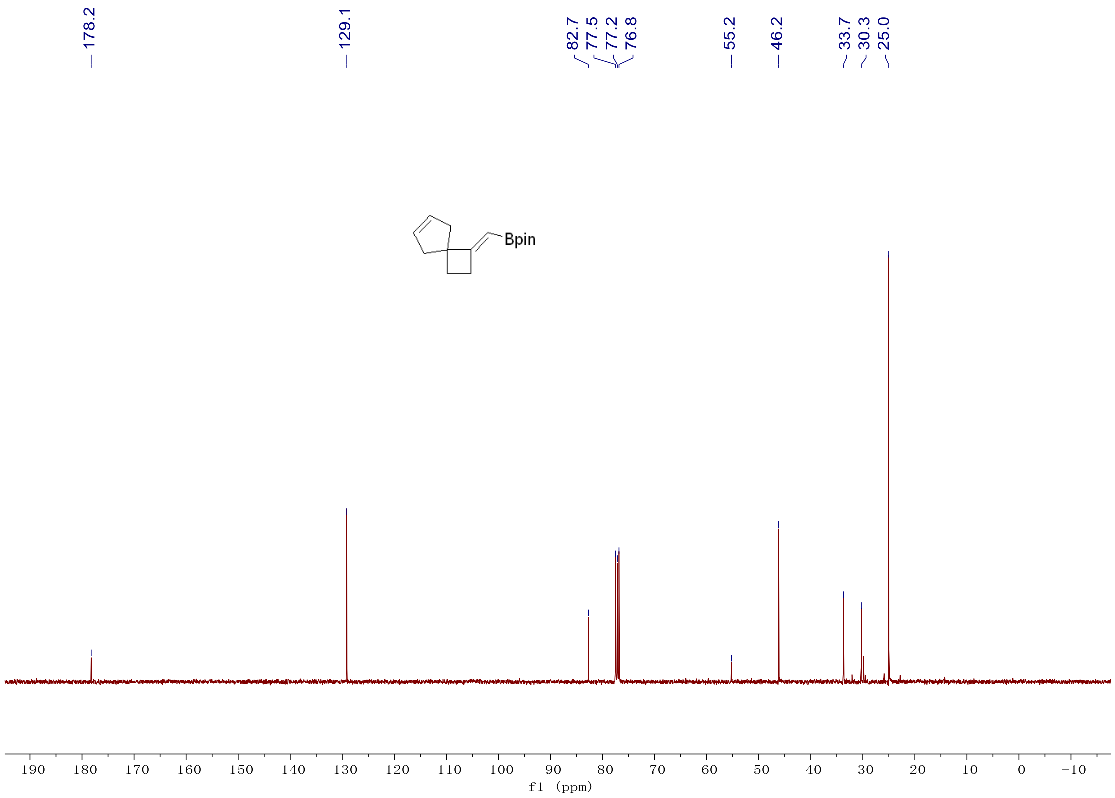

^{13}C NMR of compound 2aj

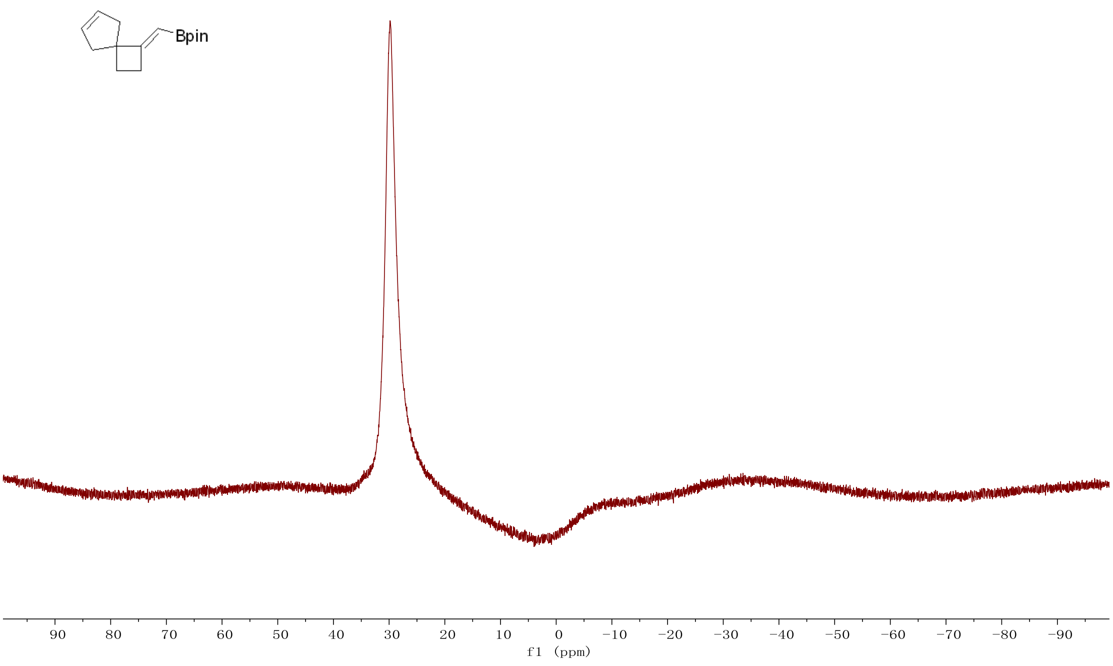

^{11}B NMR of compound 2aj

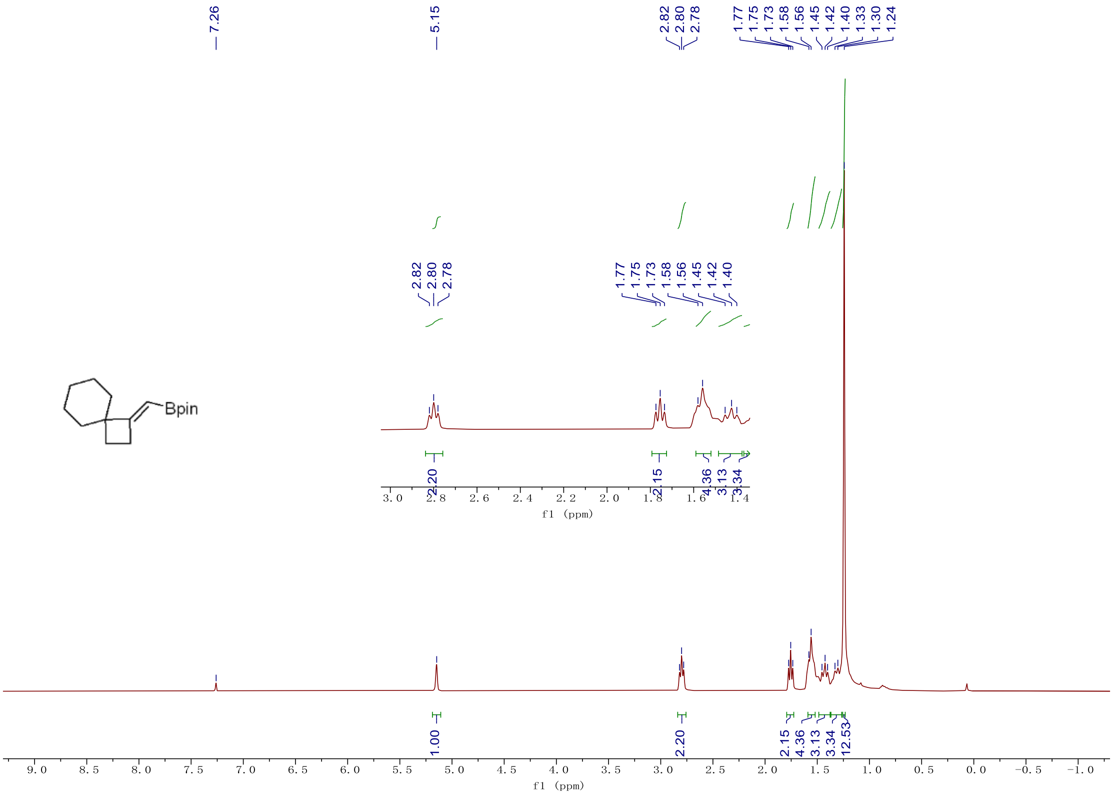

^{1}H NMR of compound 2ak

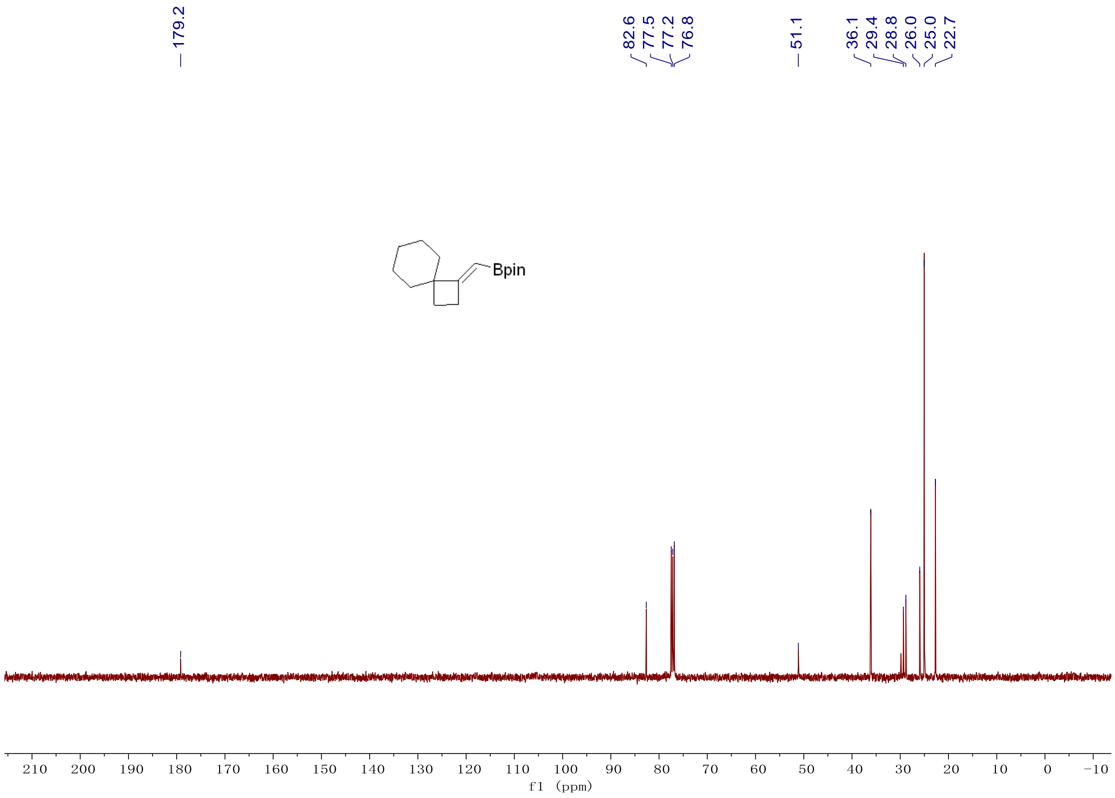

^{13}C NMR of compound 2ak

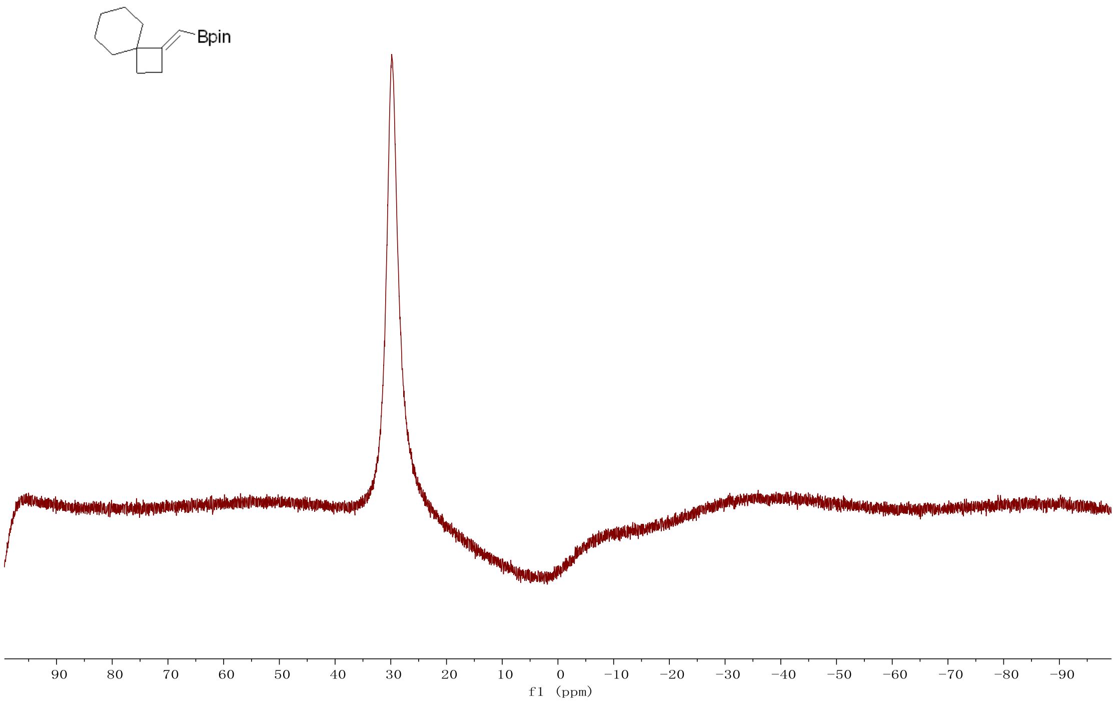

^{11}B NMR of compound 2ak

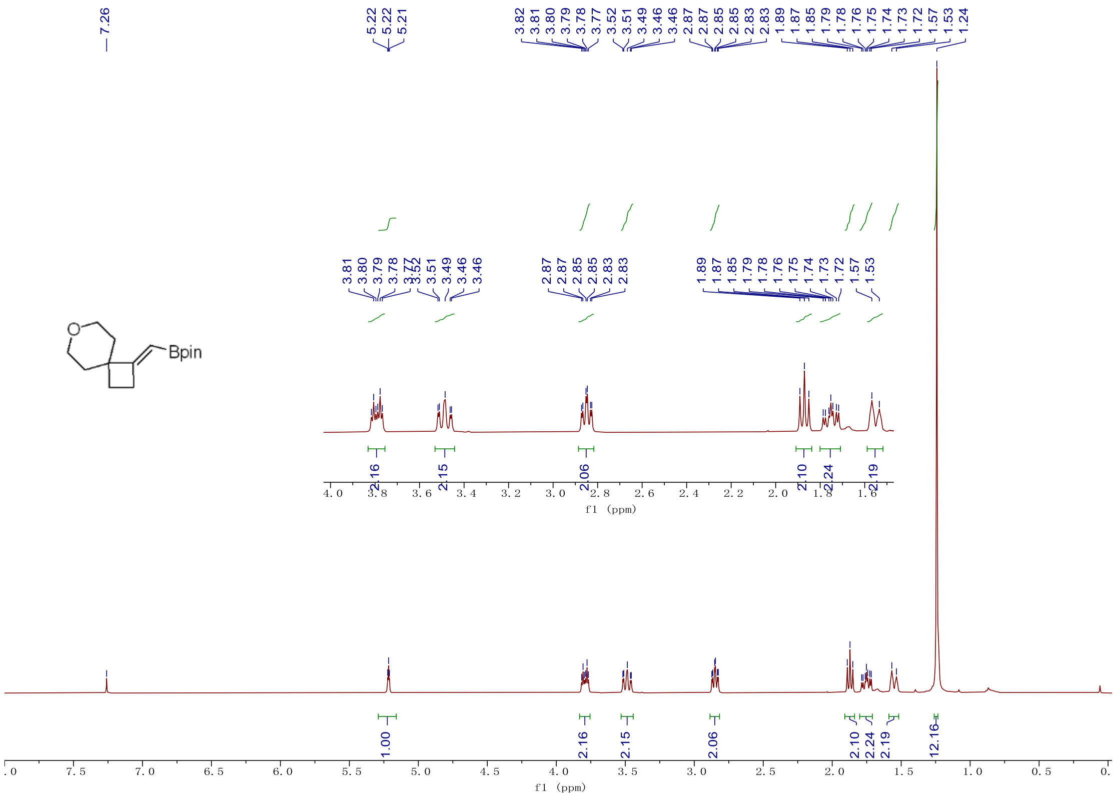

^{1}H NMR of compound 2al

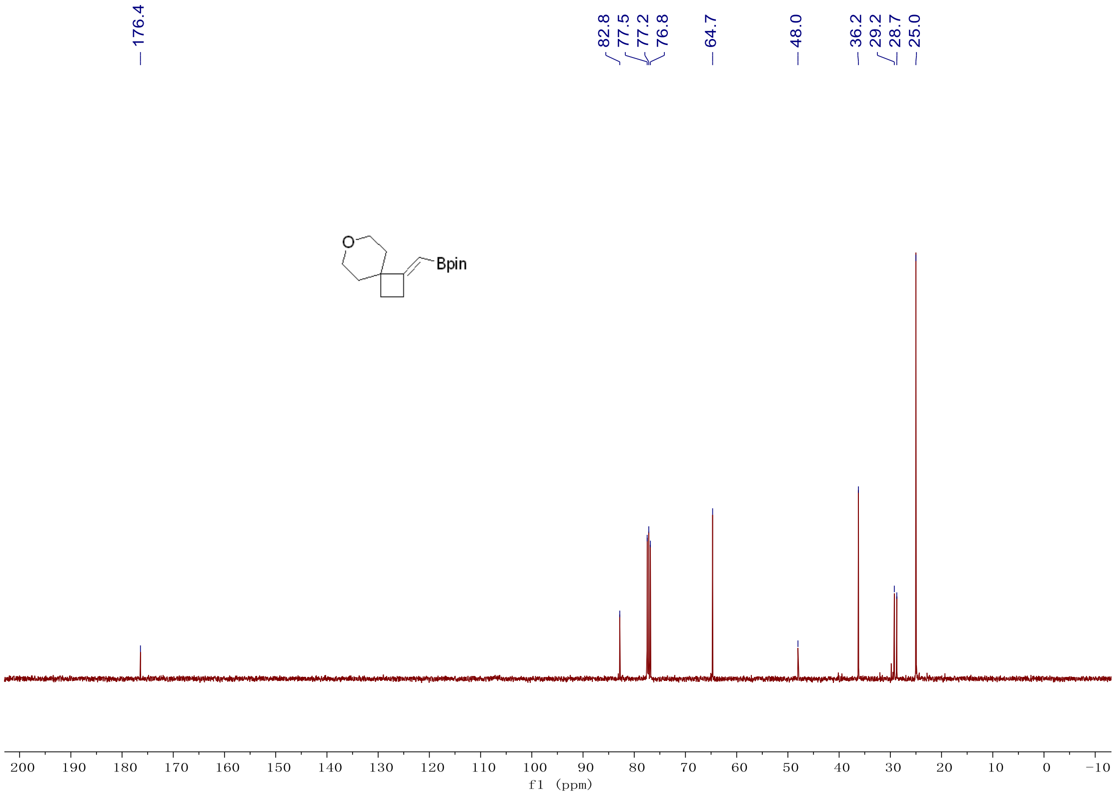

^{13}C NMR of compound 2al

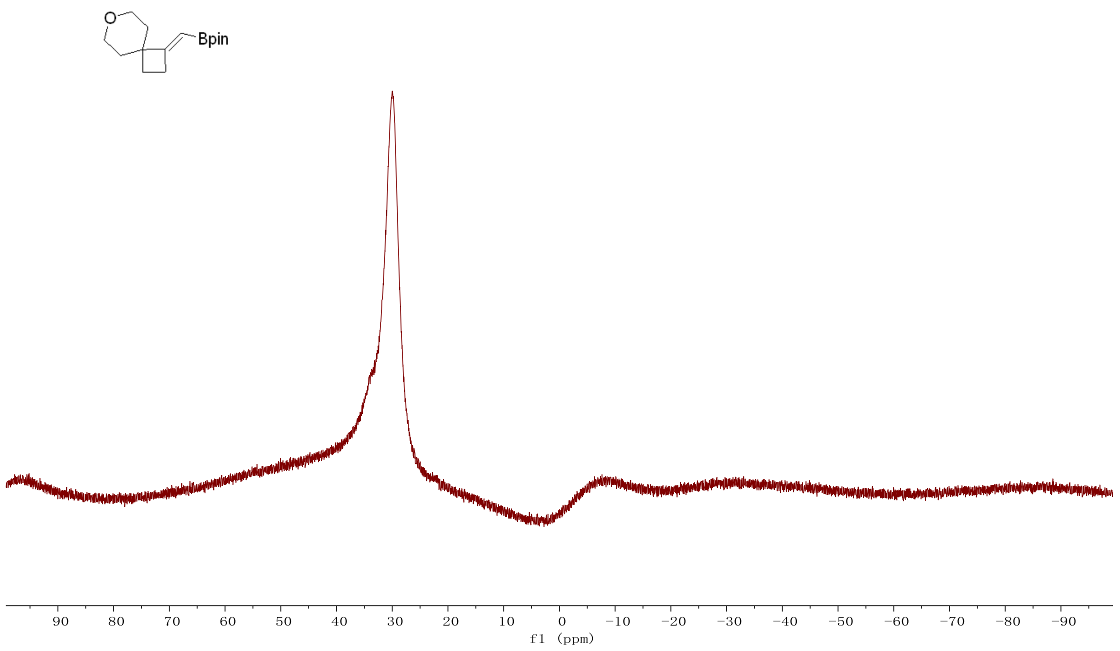

^{11}B NMR of compound 2al

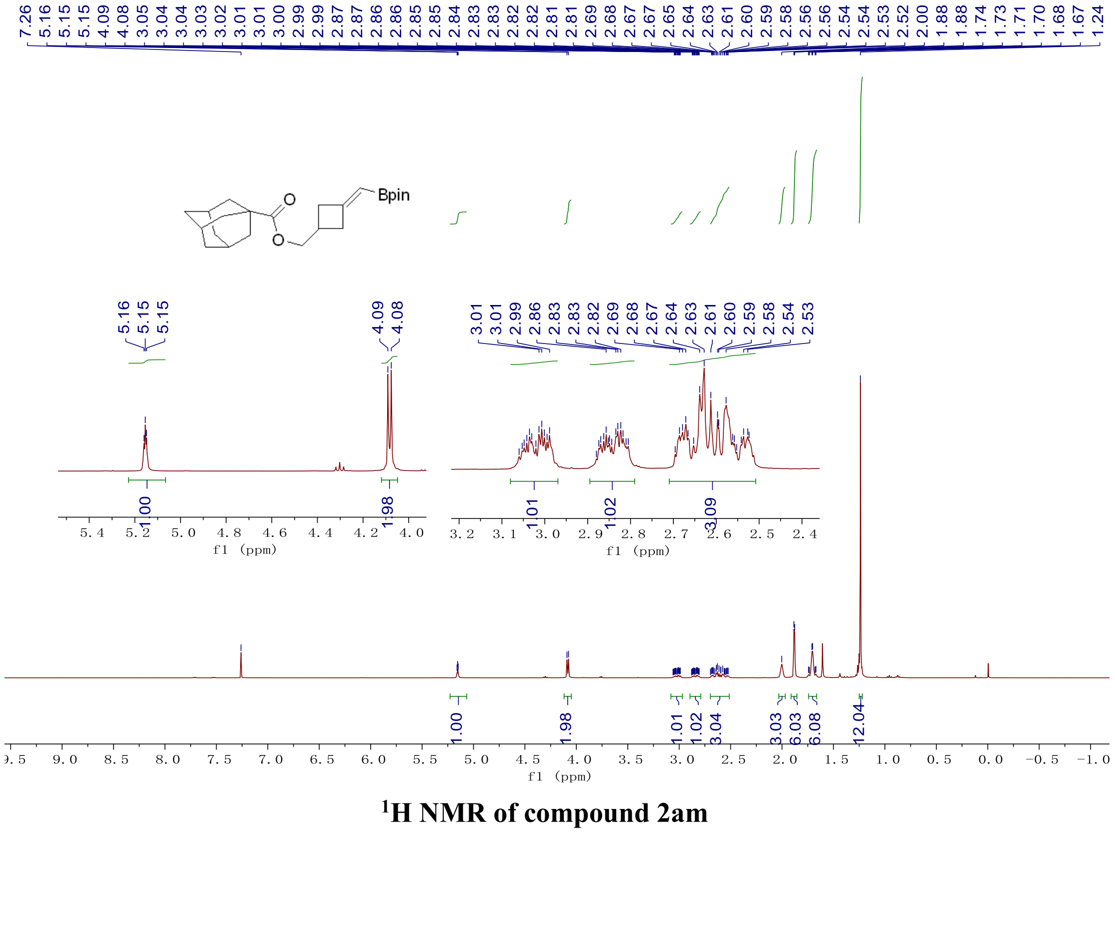

^{1}H NMR of compound 2am

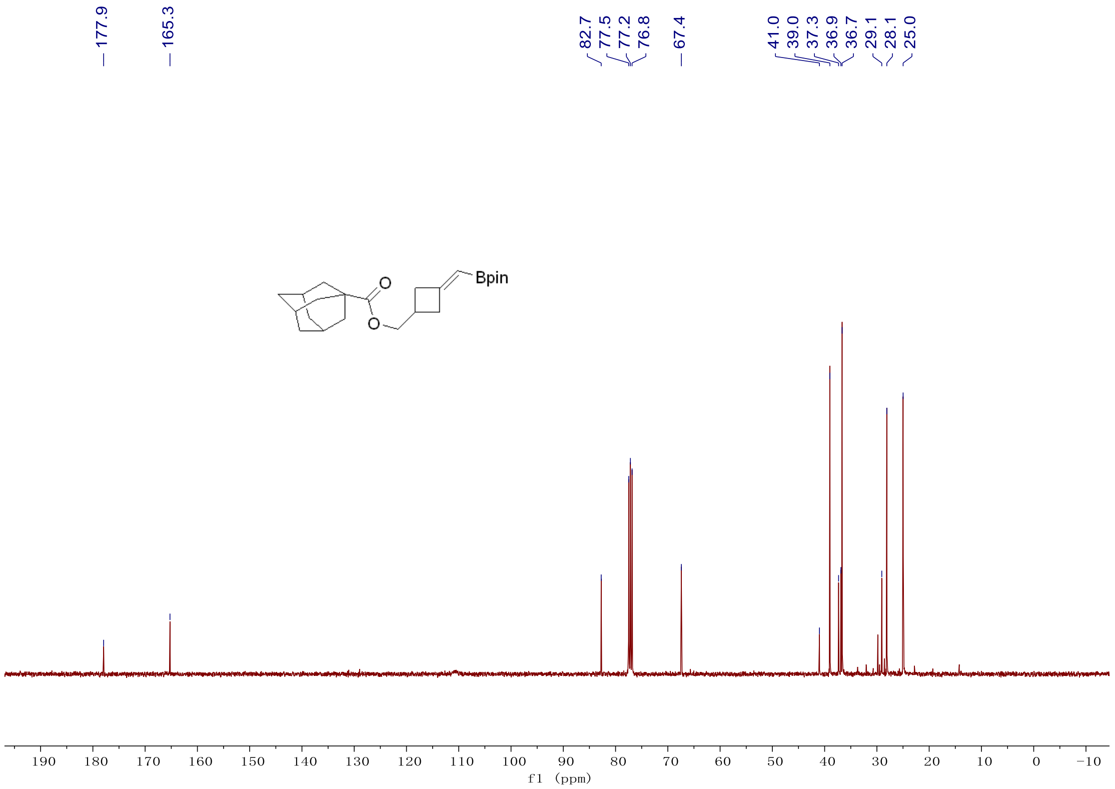

^{13}C NMR of compound 2am

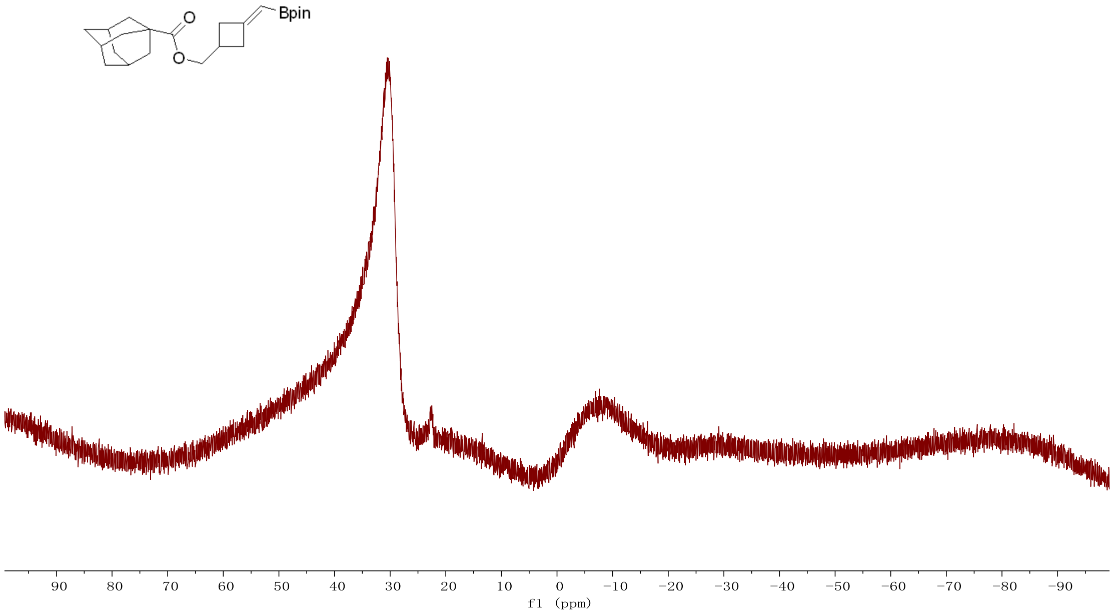

^{11}B NMR of compound 2am

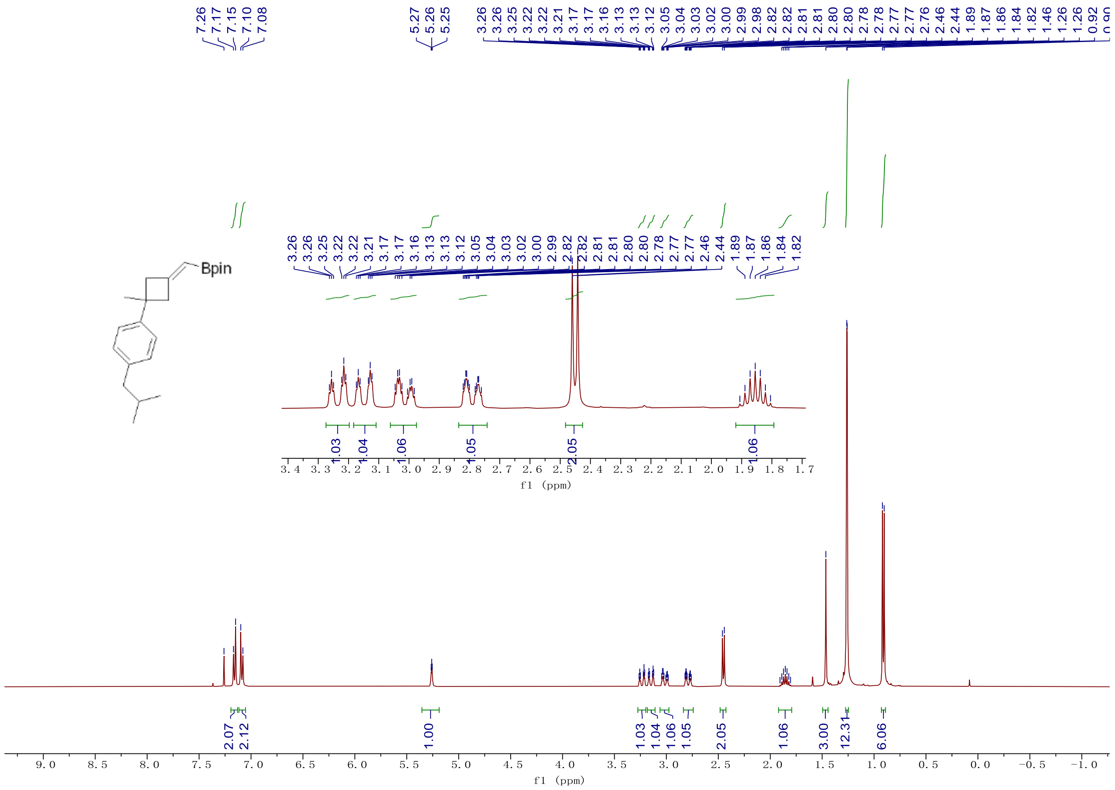

^{1}H NMR of compound 2an

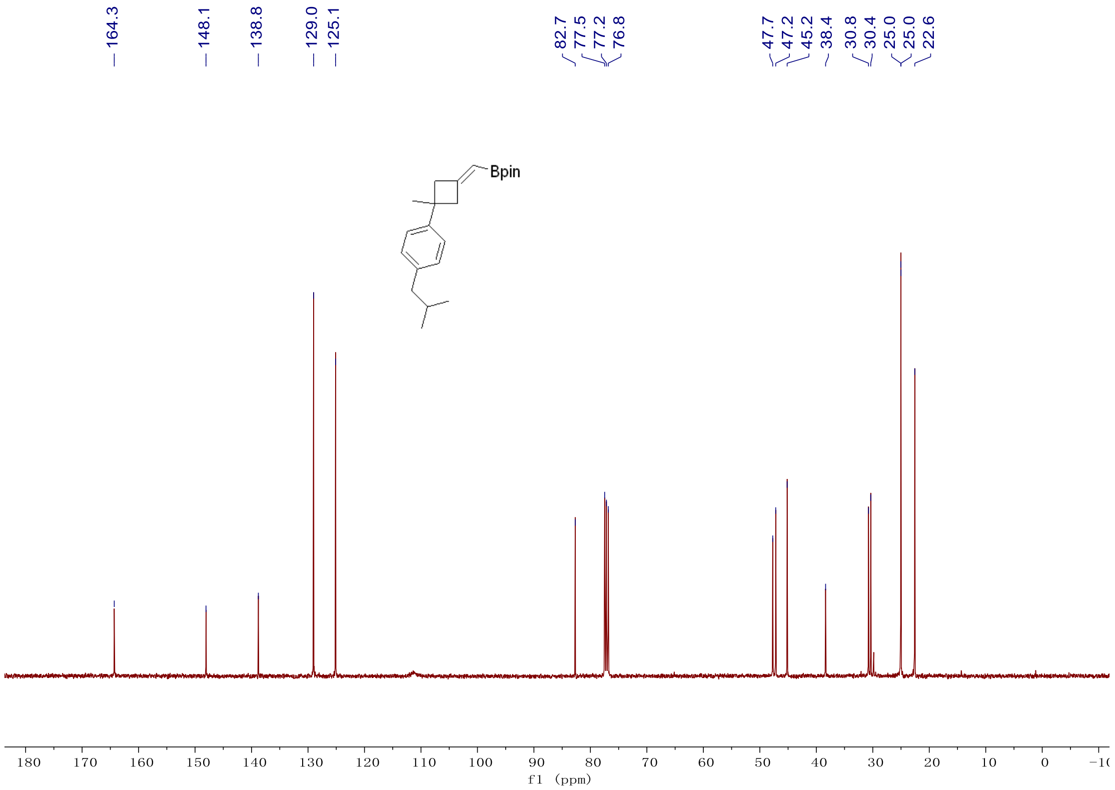

^{13}C NMR of compound 2an

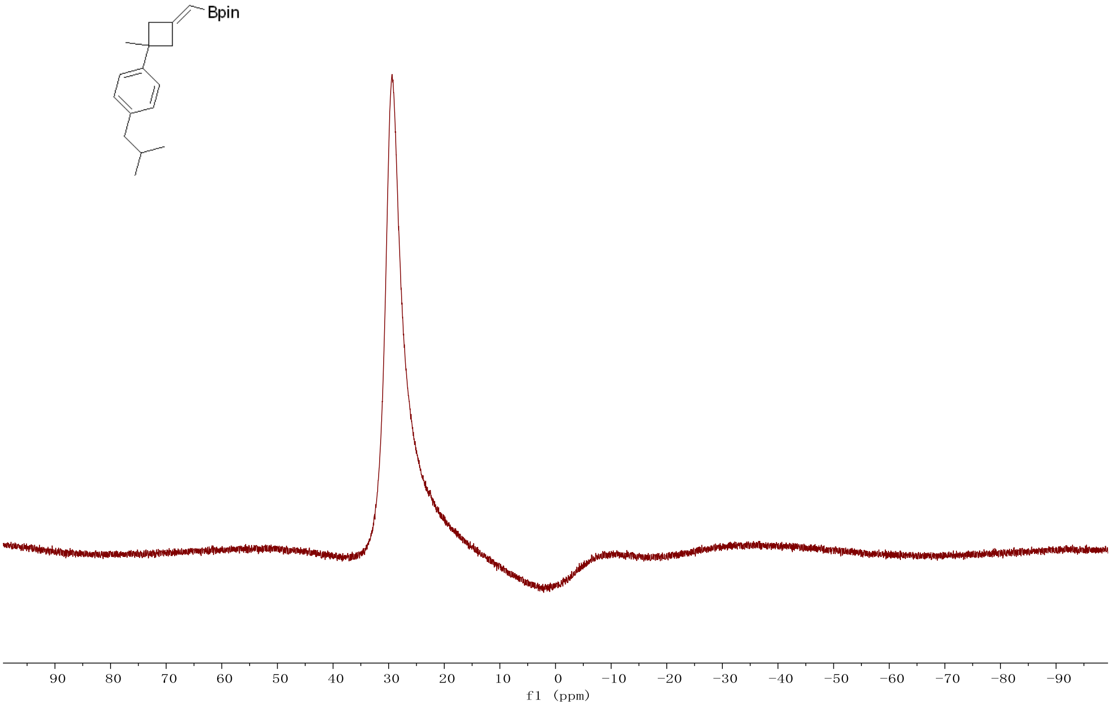

^{11}B NMR of compound 2an

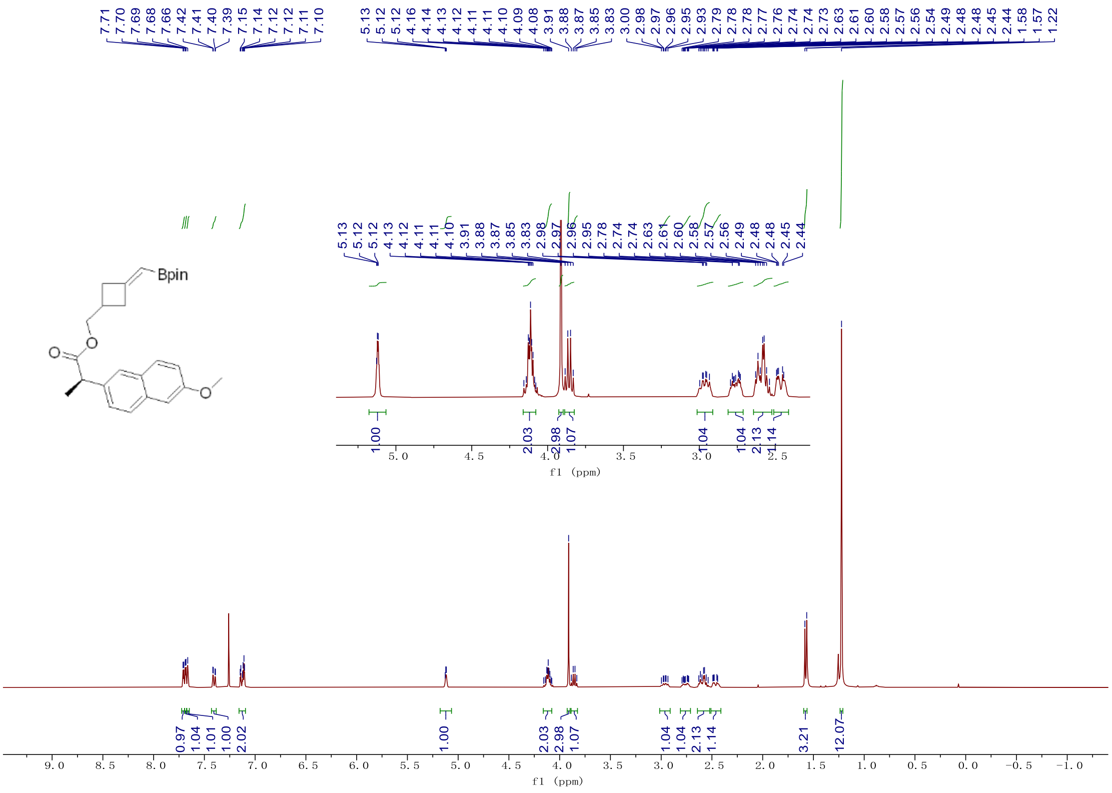

^{1}H NMR of compound 2ao

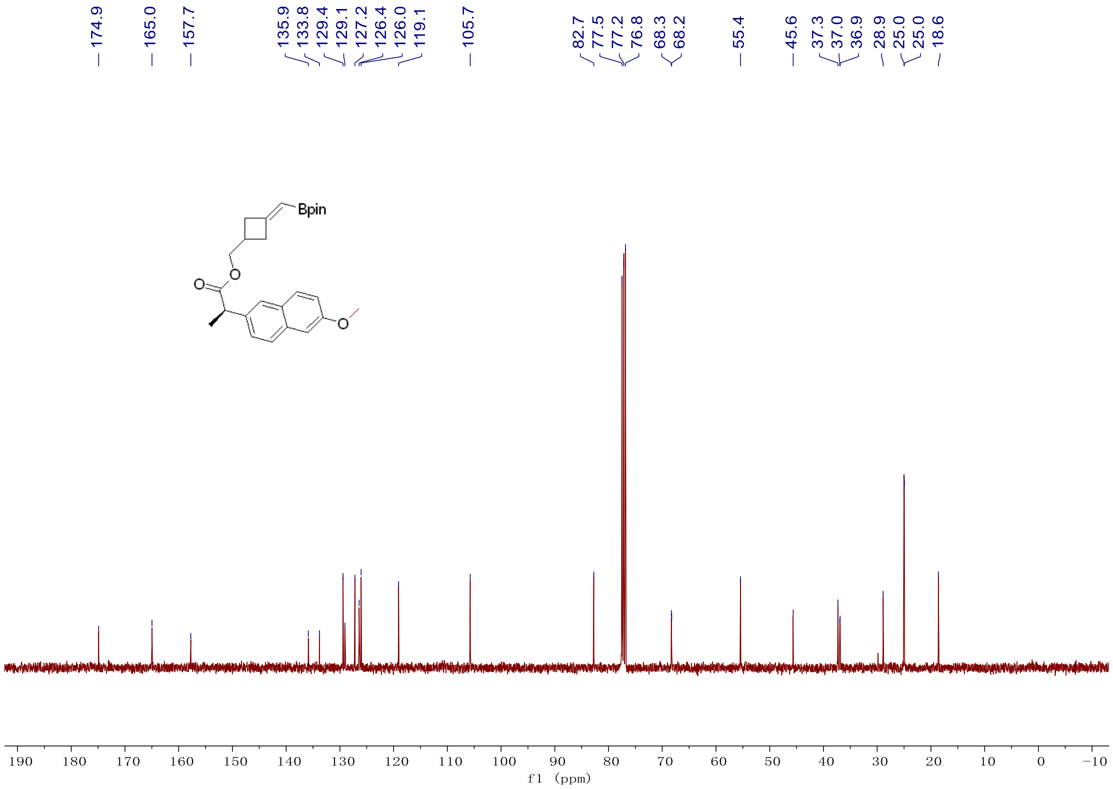

^{13}C NMR of compound 2ao

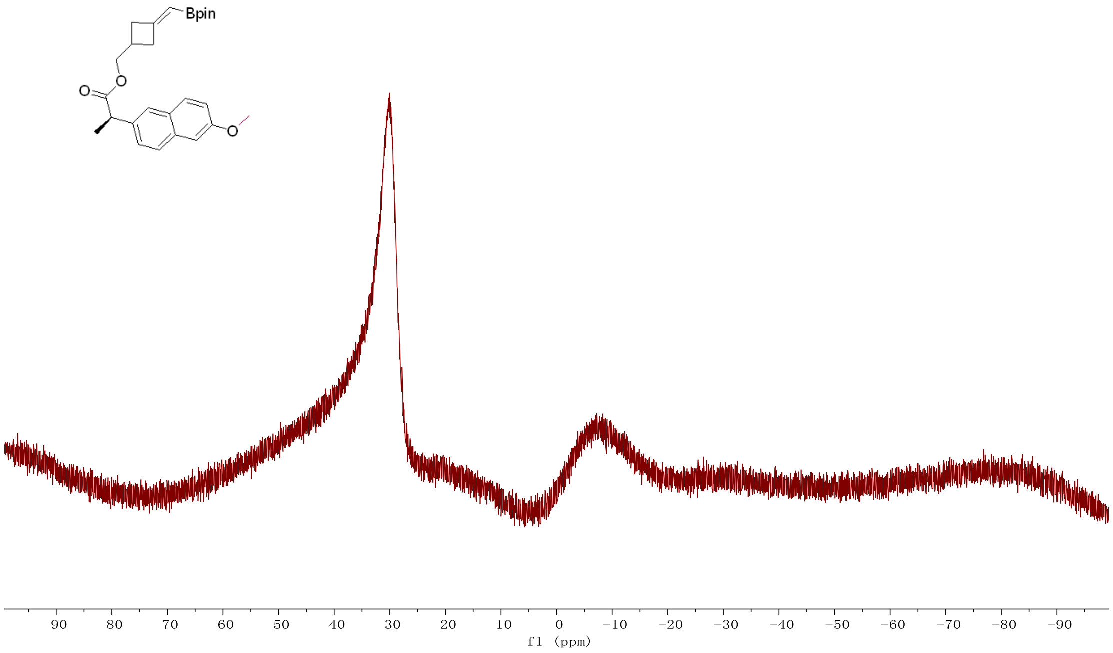

^{11}B NMR of compound 2ao

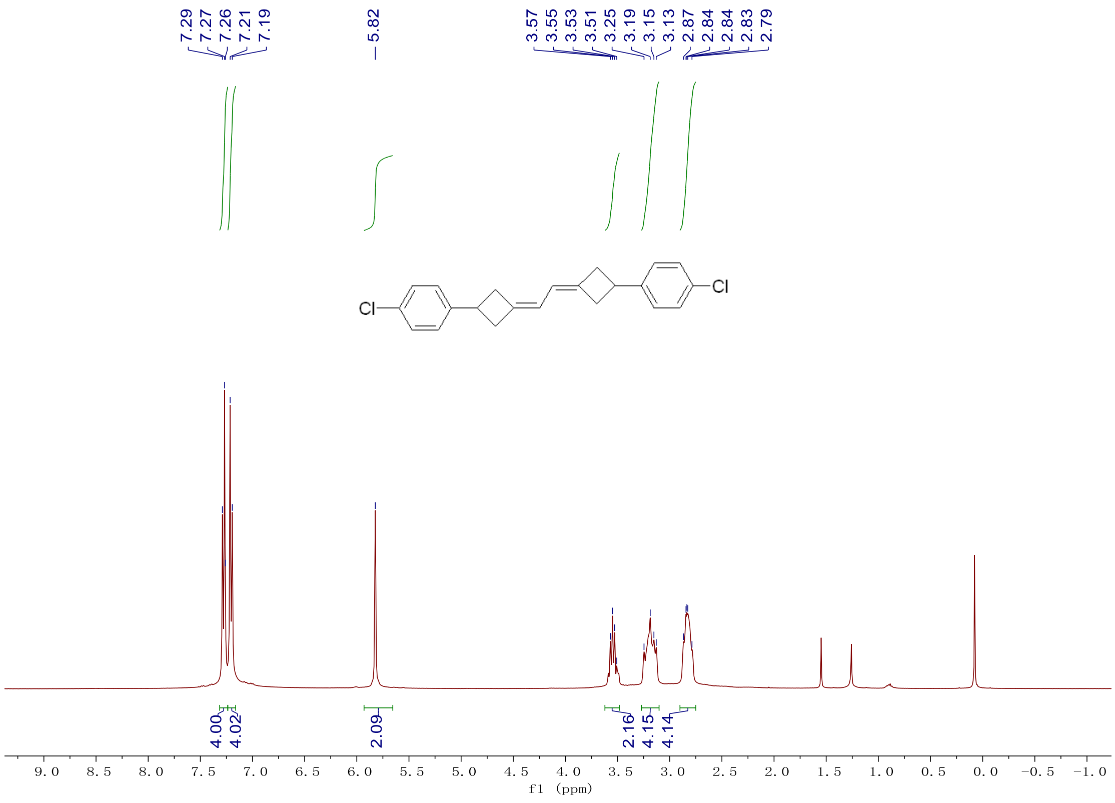

^{1}H NMR of compound 3

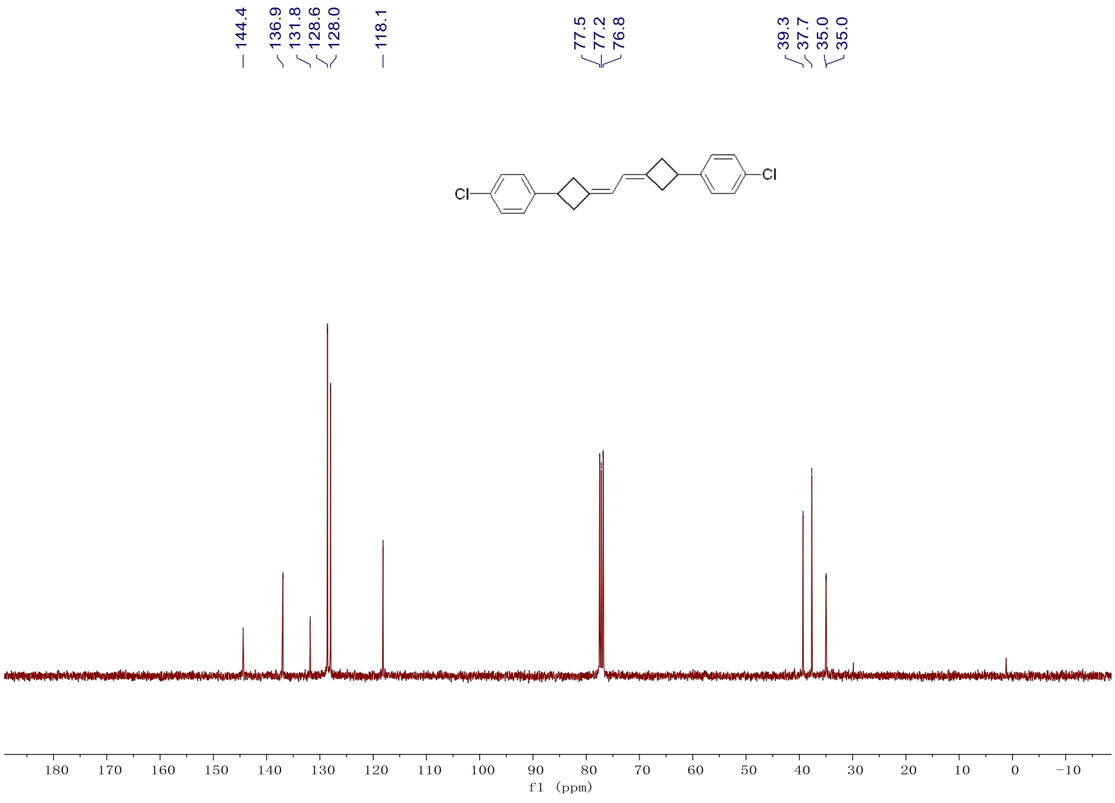

^{13}C NMR of compound 3

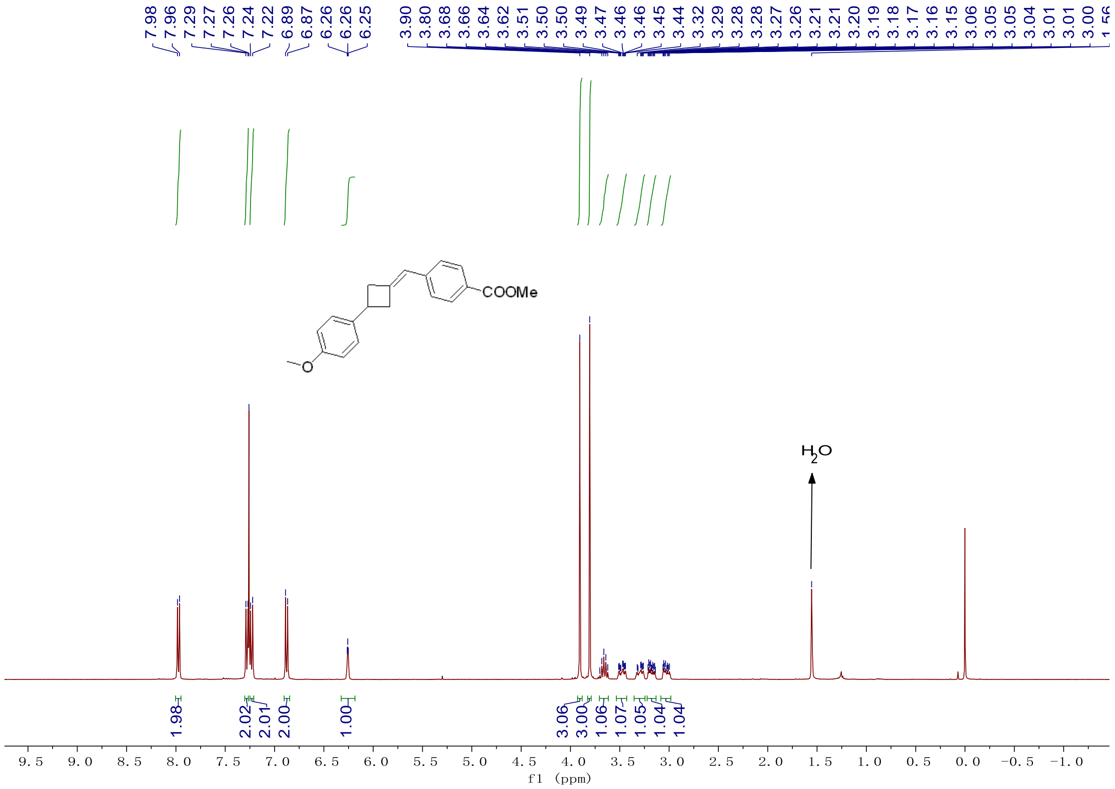

^{1}H NMR of compound 4

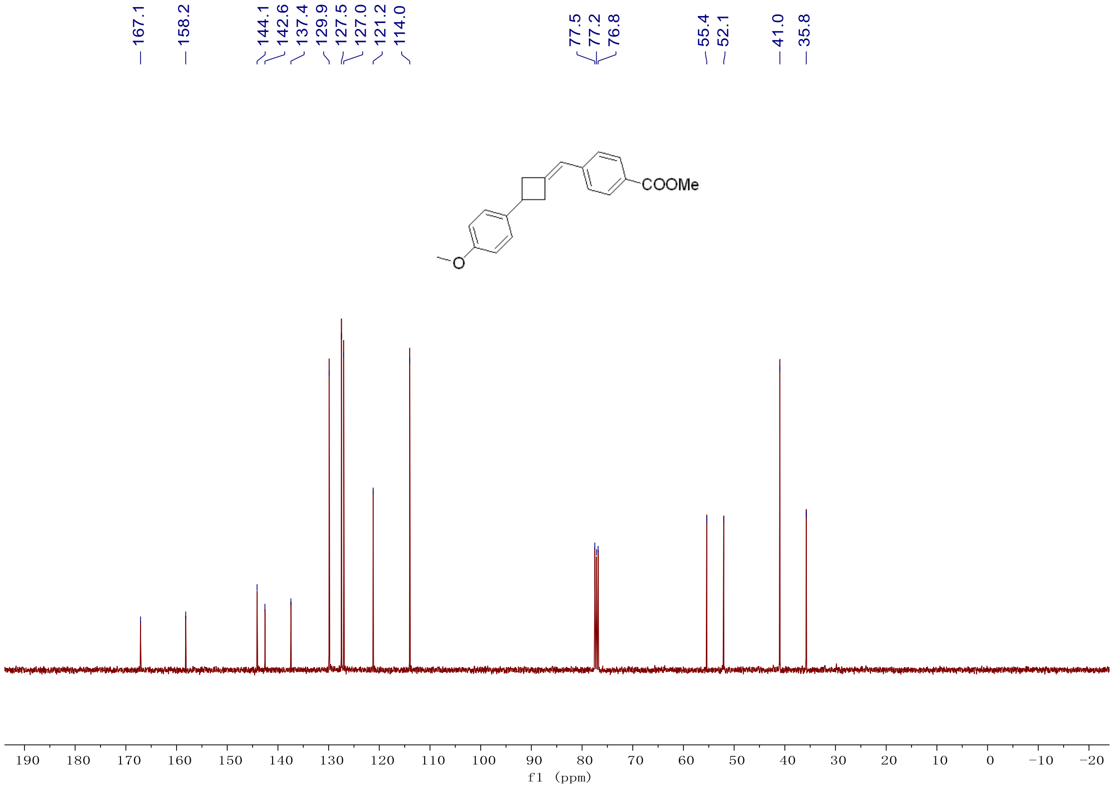

^{13}C NMR of compound 4

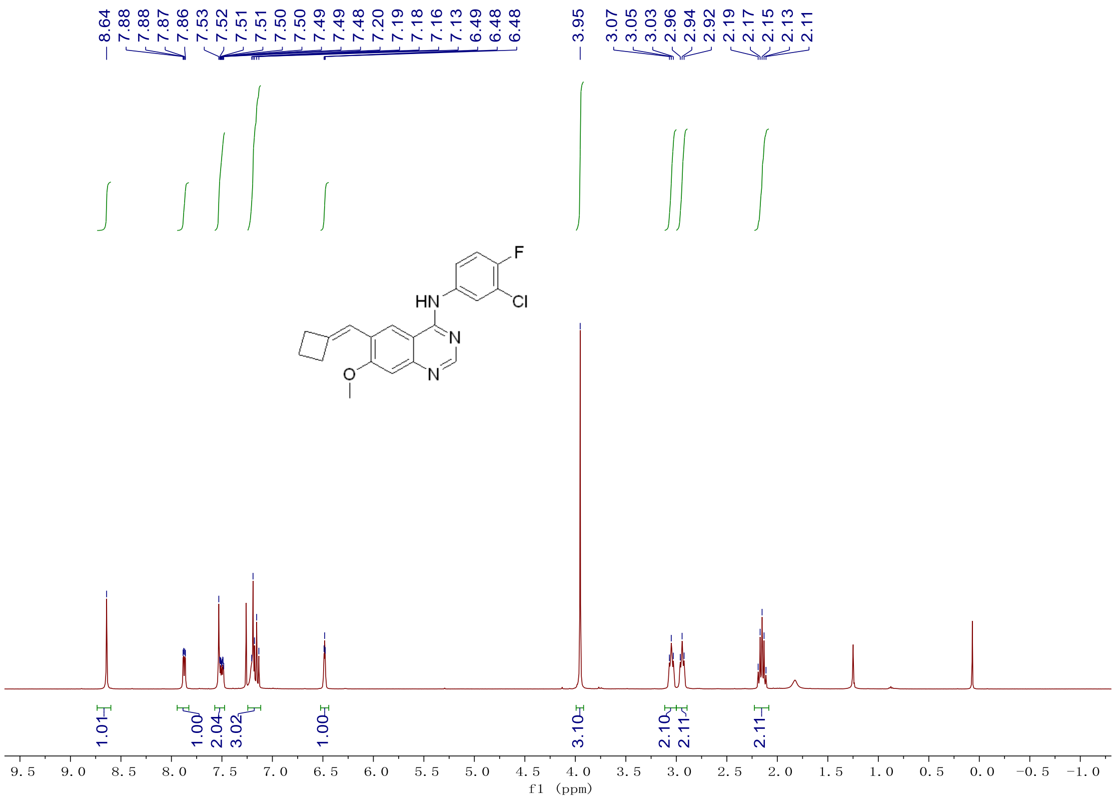

^{1}H NMR of compound 5

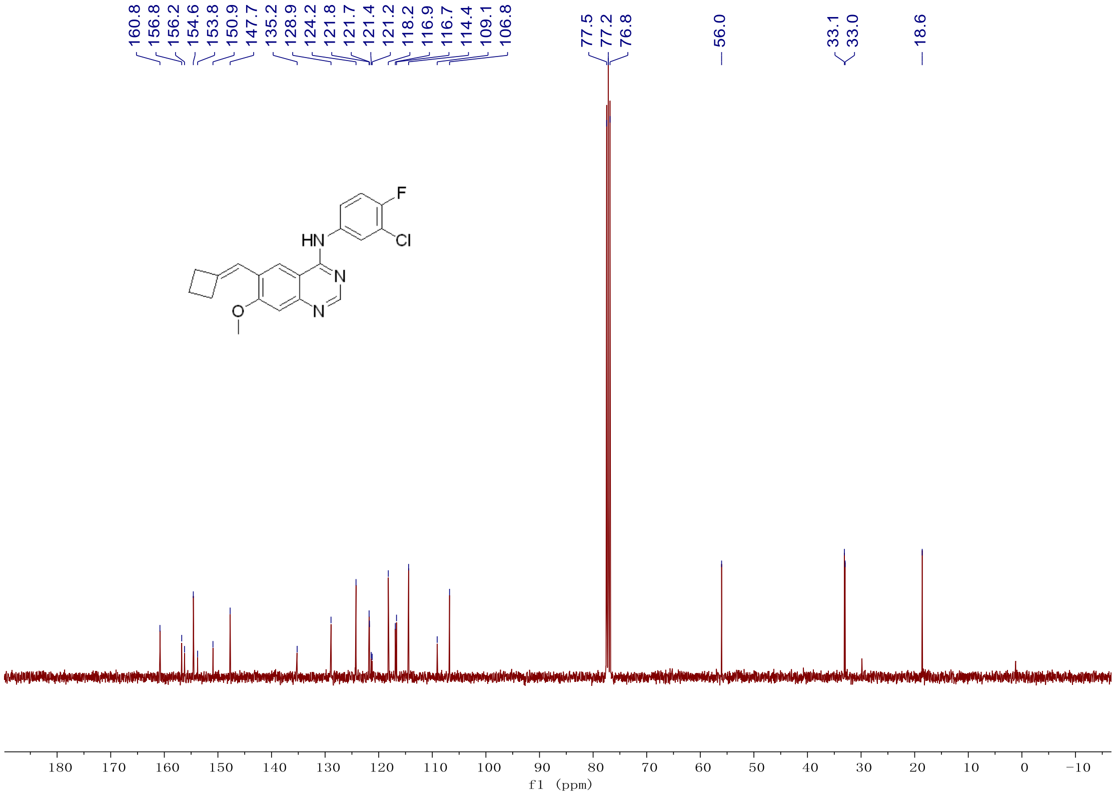

^{13}C NMR of compound 5

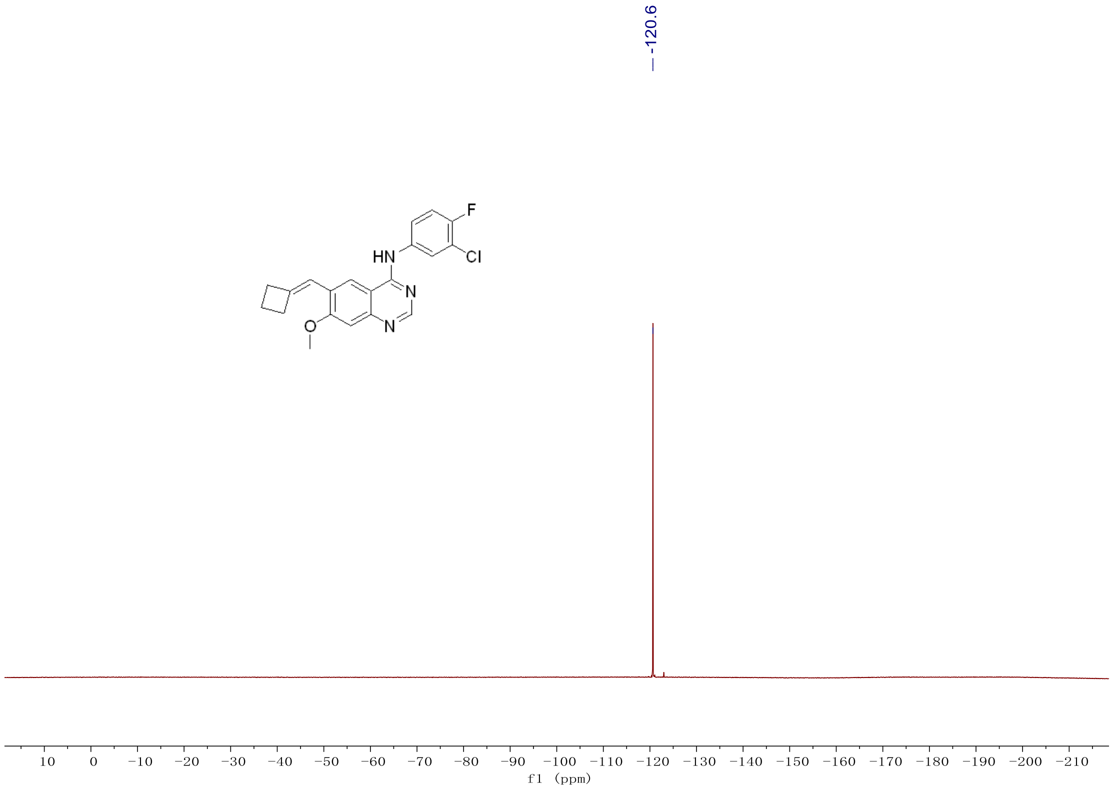

^{19}F NMR of compound 5

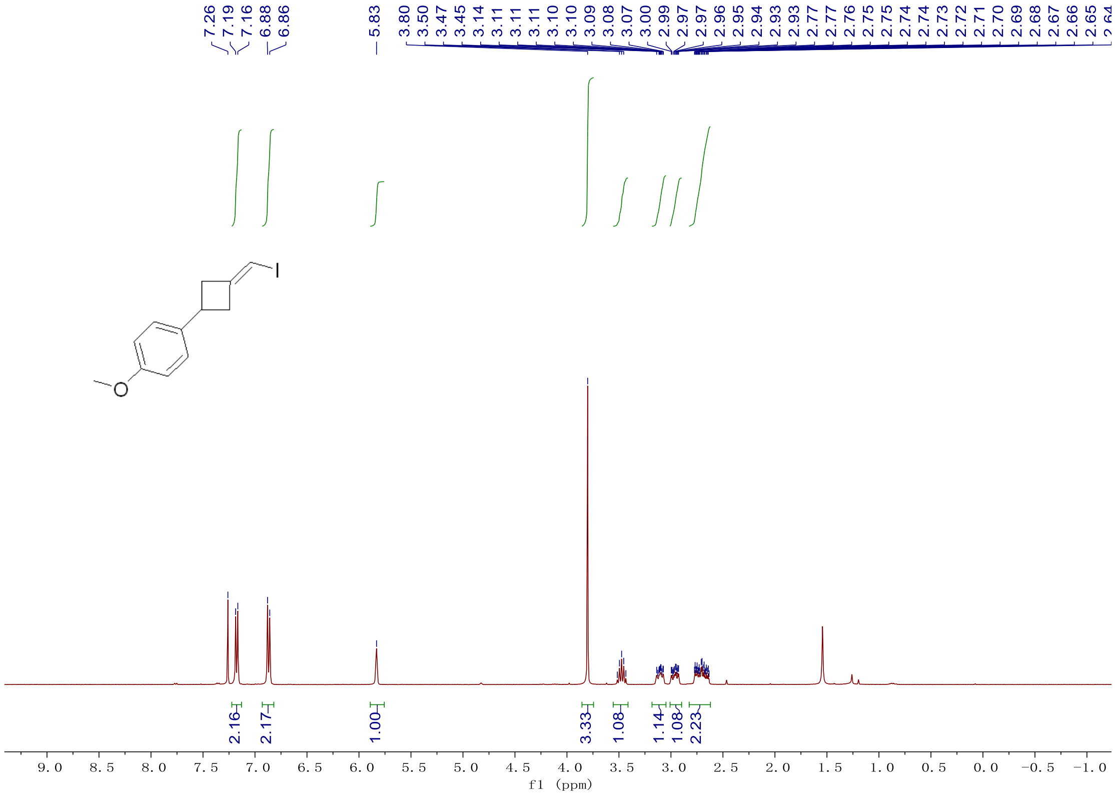

^{1}H NMR of compound 6

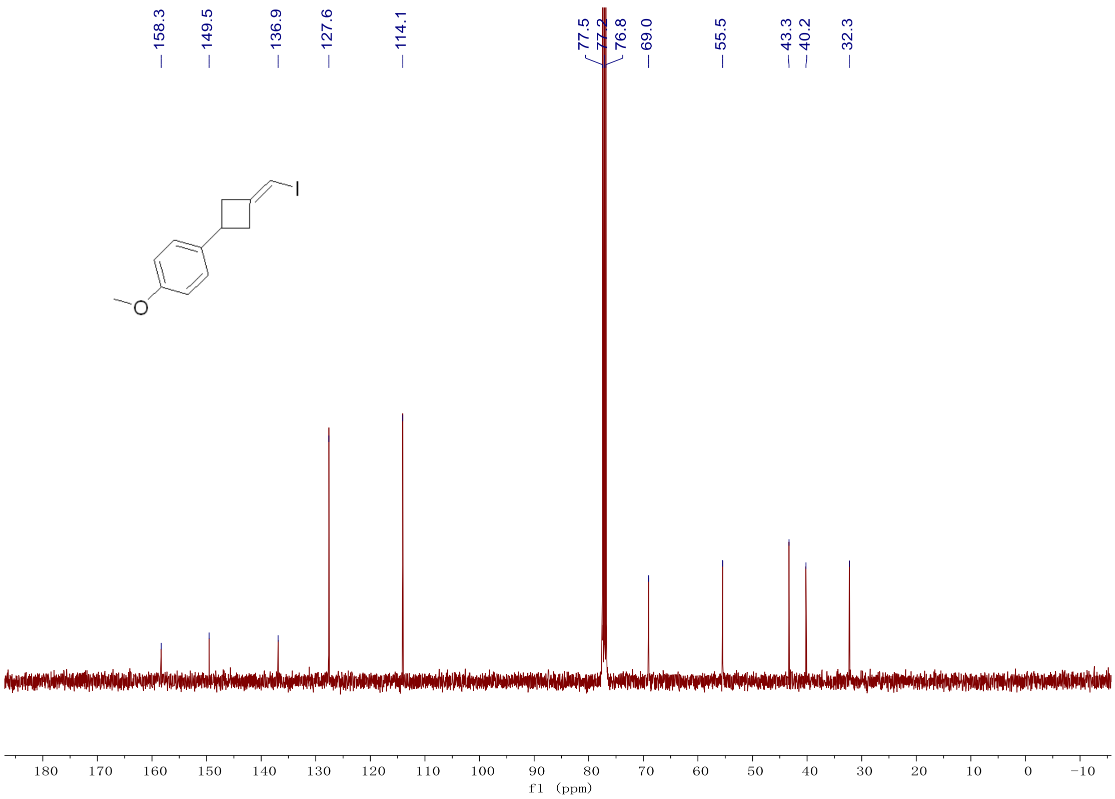

^{13}C NMR of compound 6

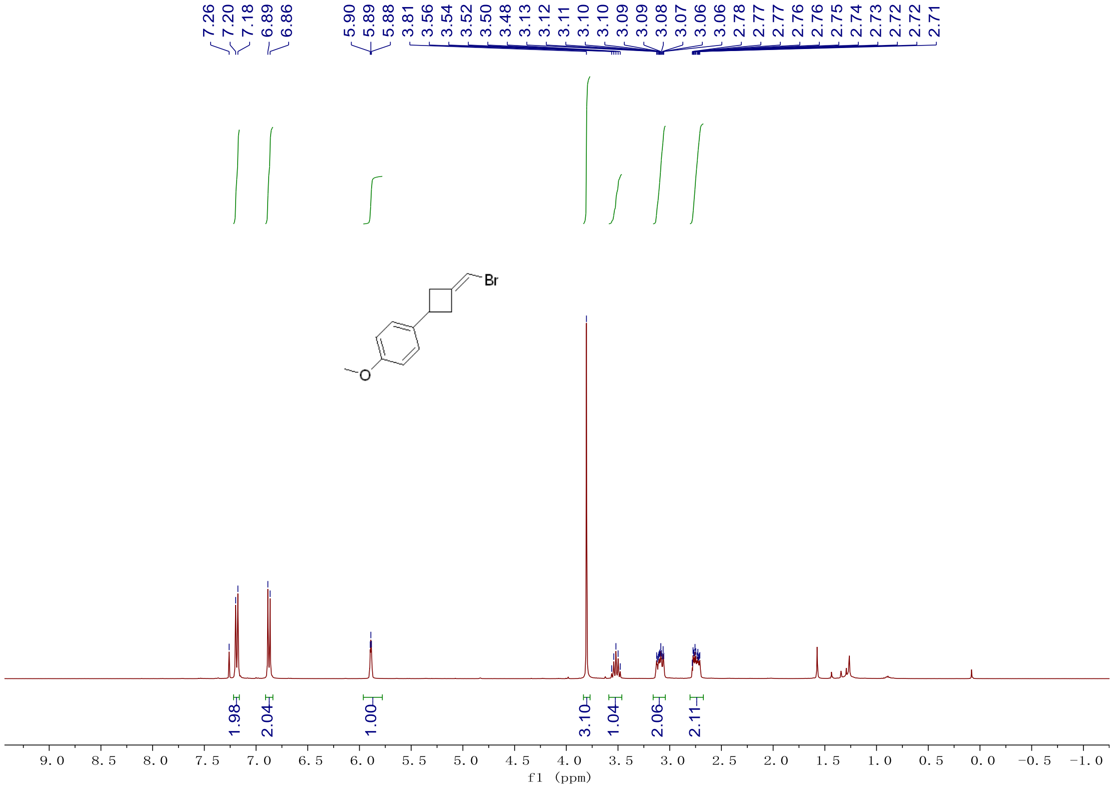

^{1}H NMR of compound 7

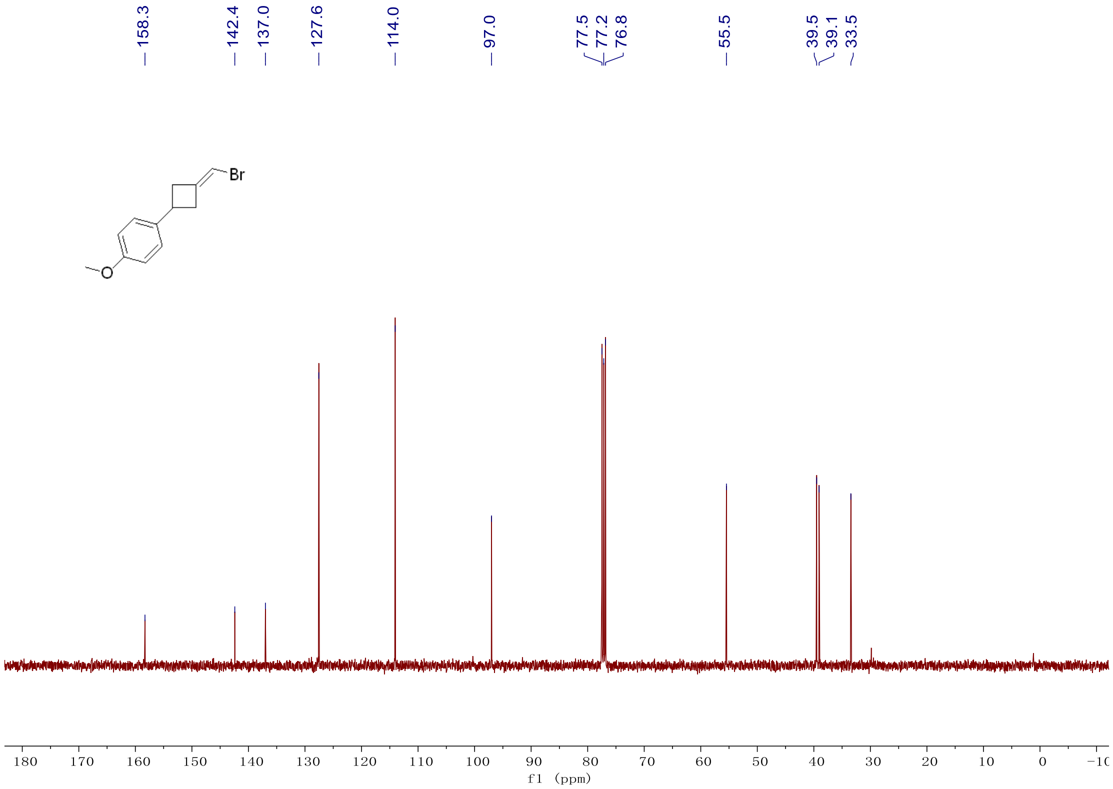

^{13}C NMR of compound 7

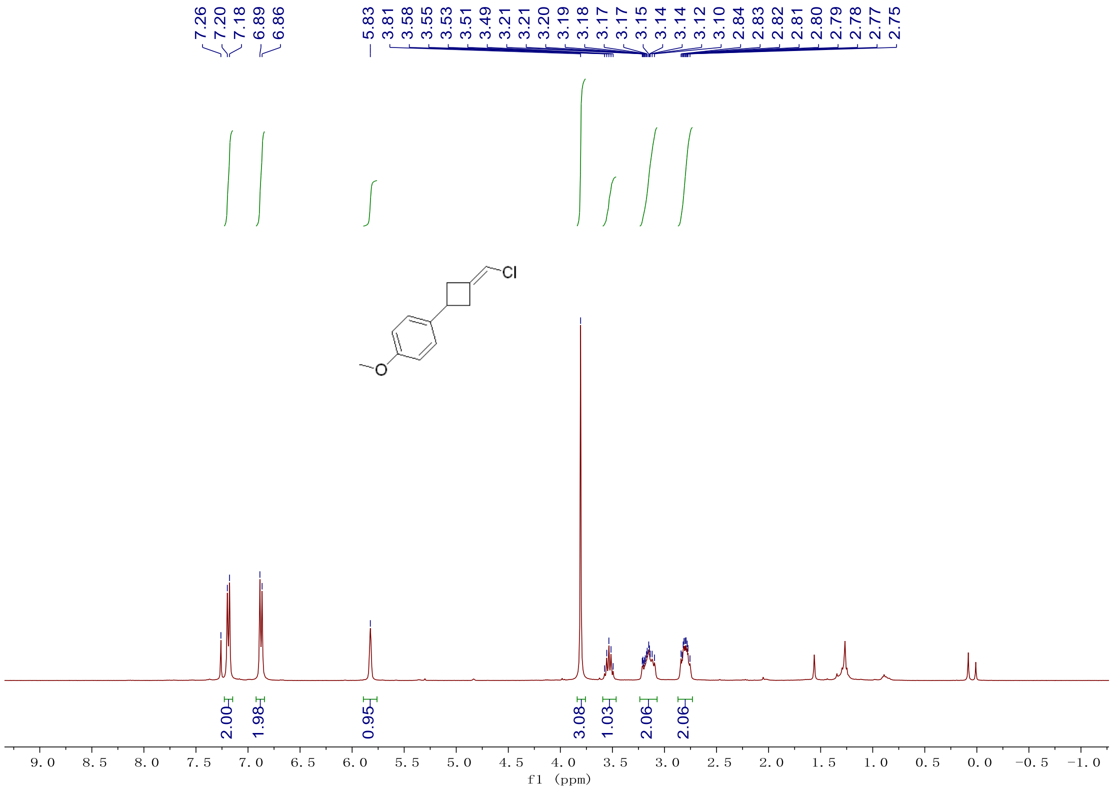

^{1}H NMR of compound 8

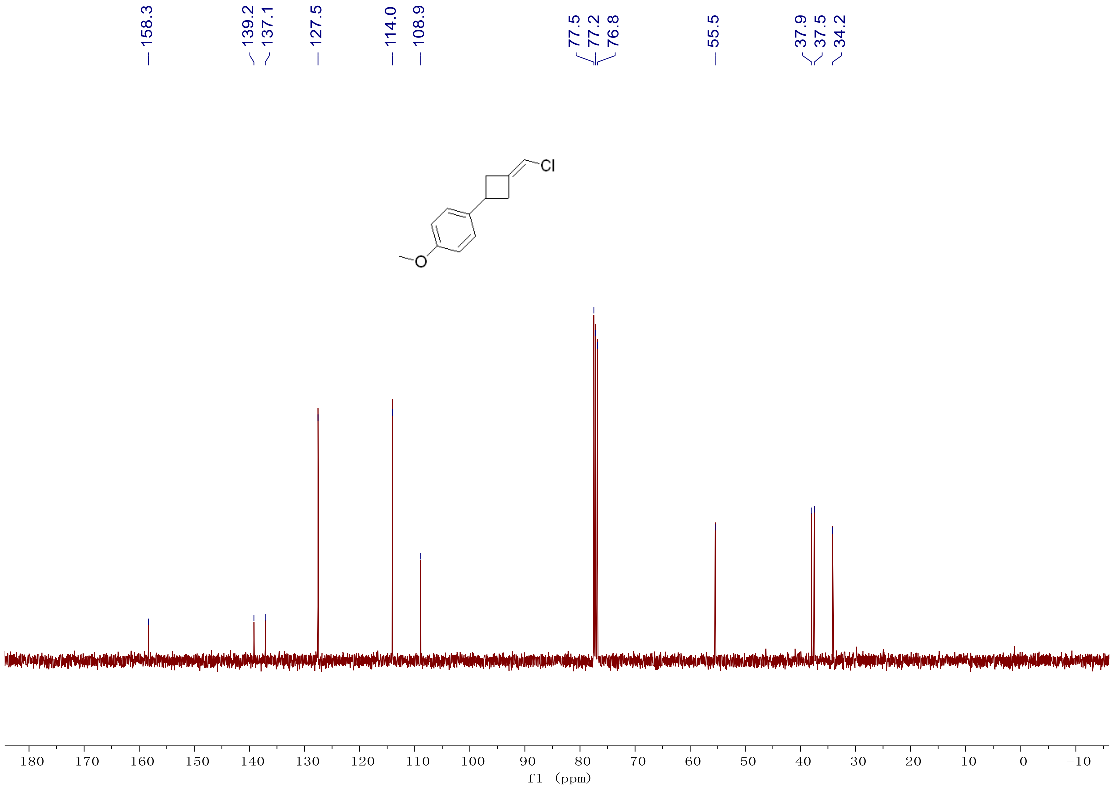

^{13}C NMR of compound 8

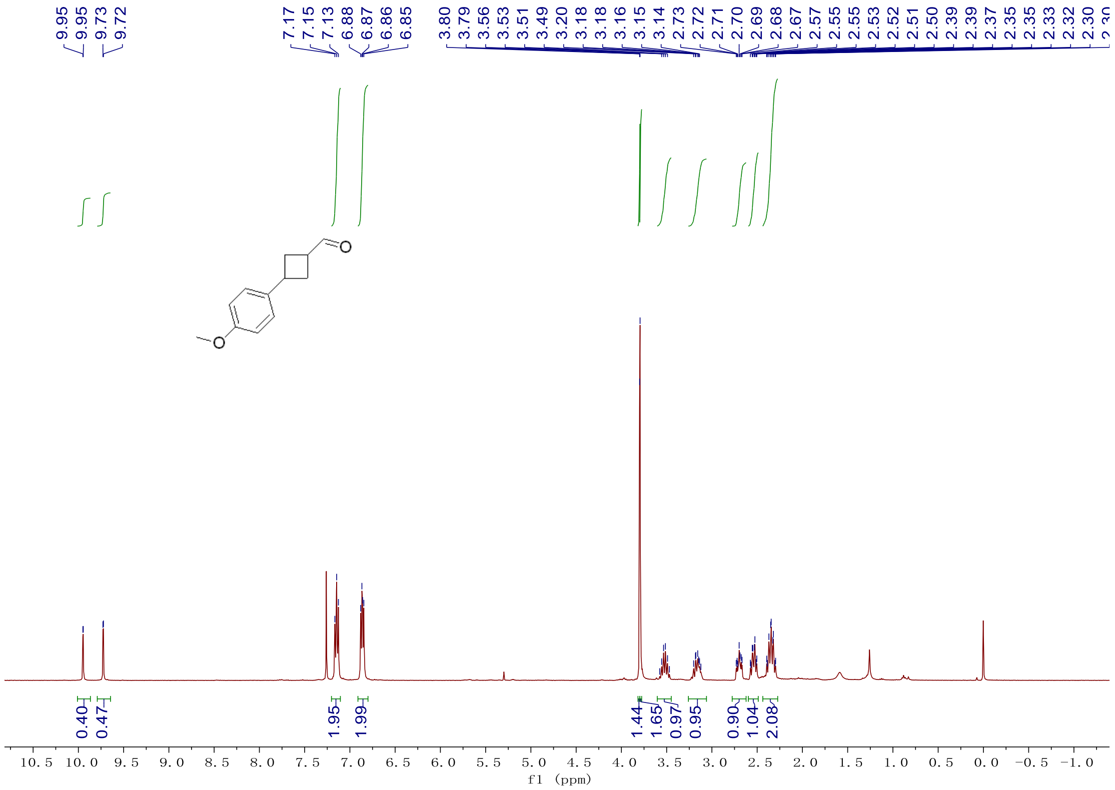

^{1}H NMR of compound 9

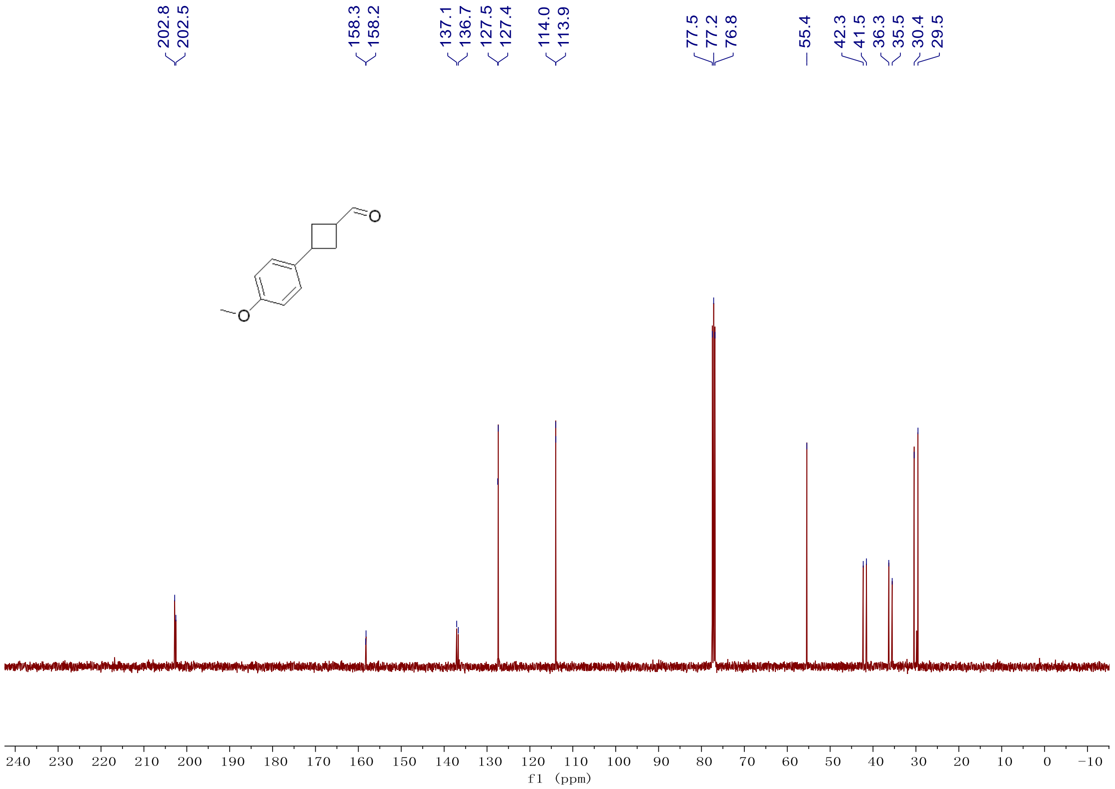

^{13}C NMR of compound 9

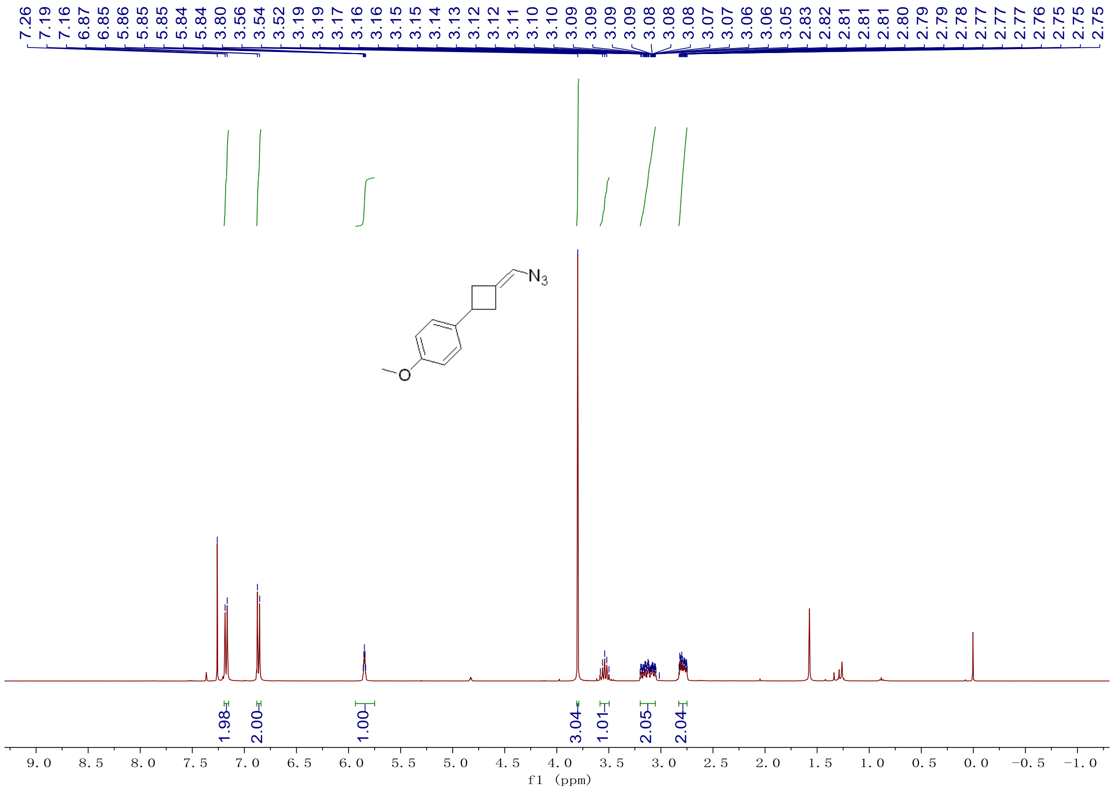

^{1}H NMR of compound 10

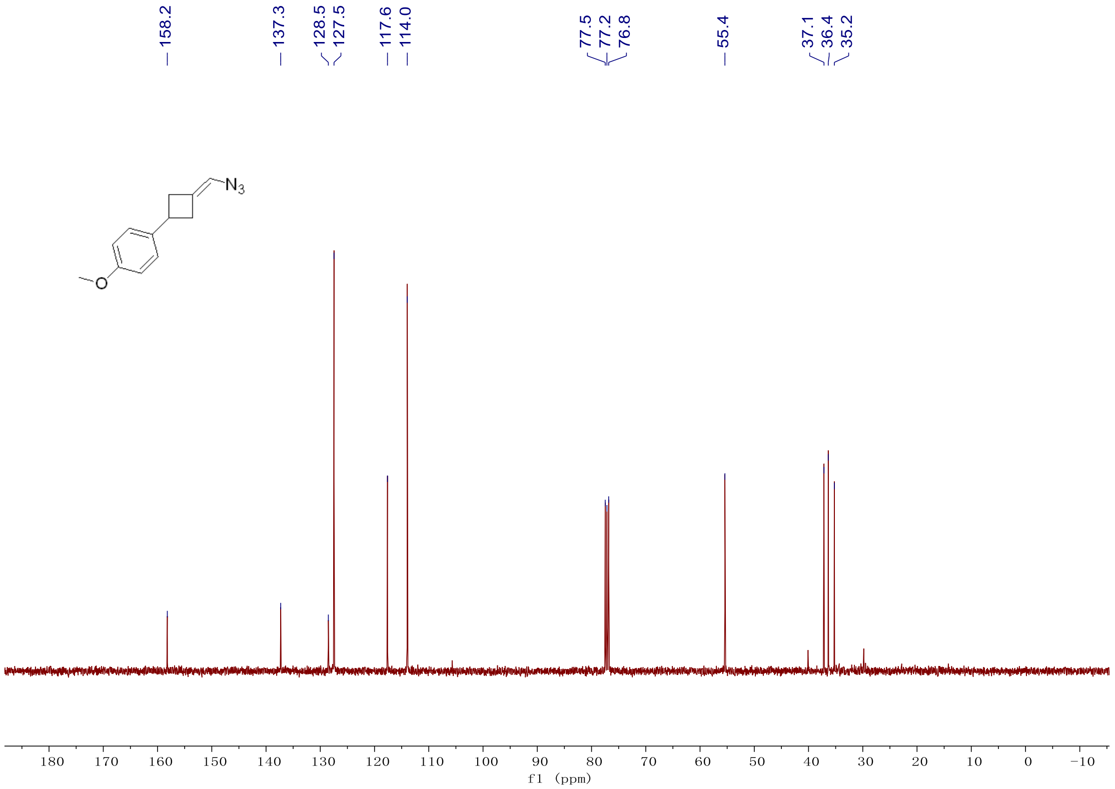

^{13}C NMR of compound 10

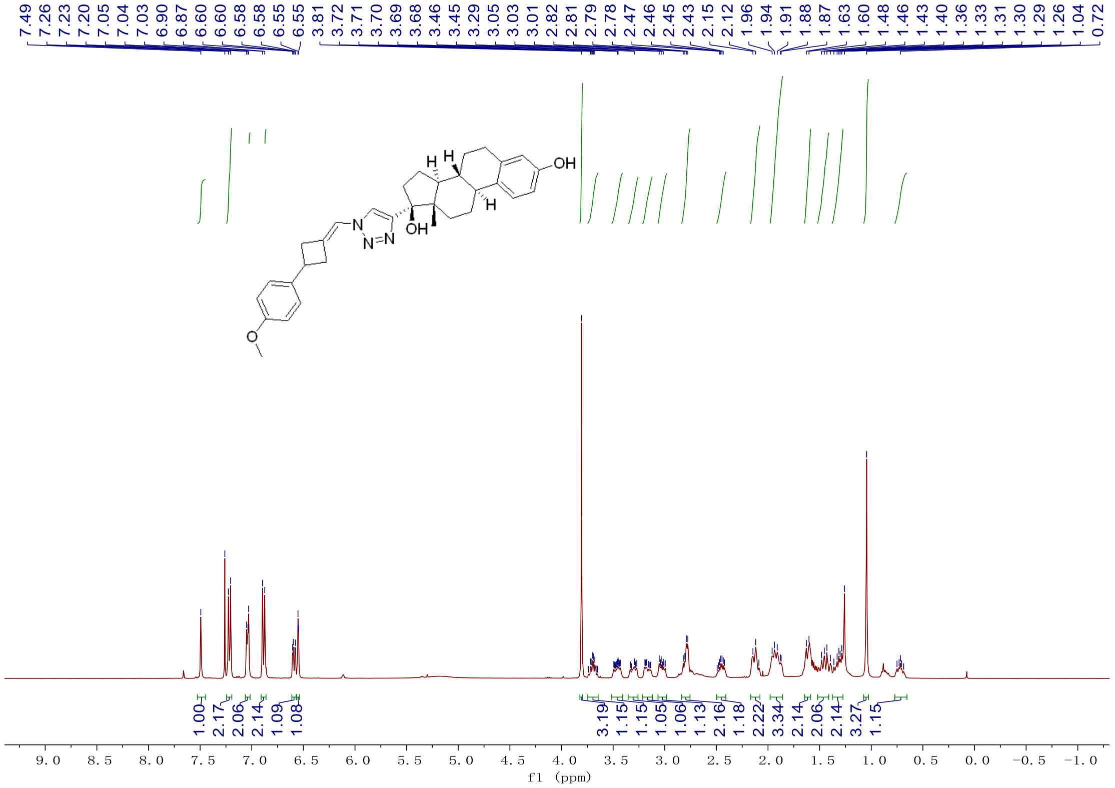

^{1}H NMR of compound 11

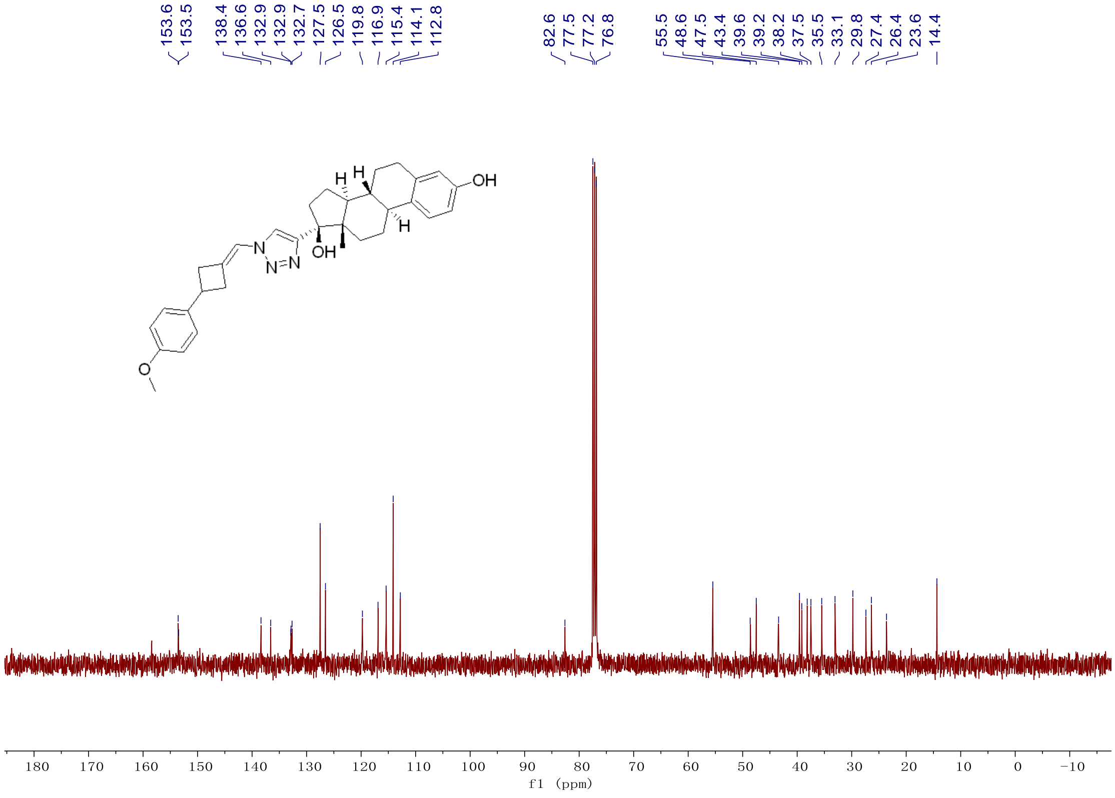

^{13}C NMR of compound 11

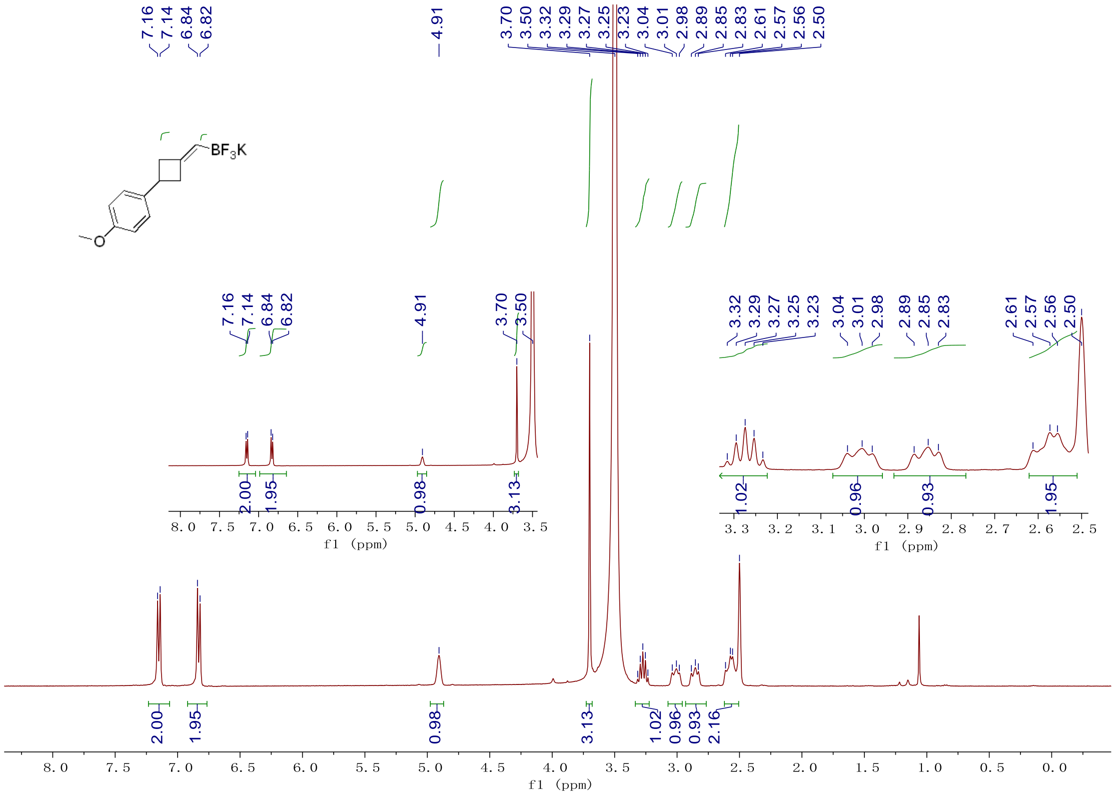

^{1}H NMR of compound 12

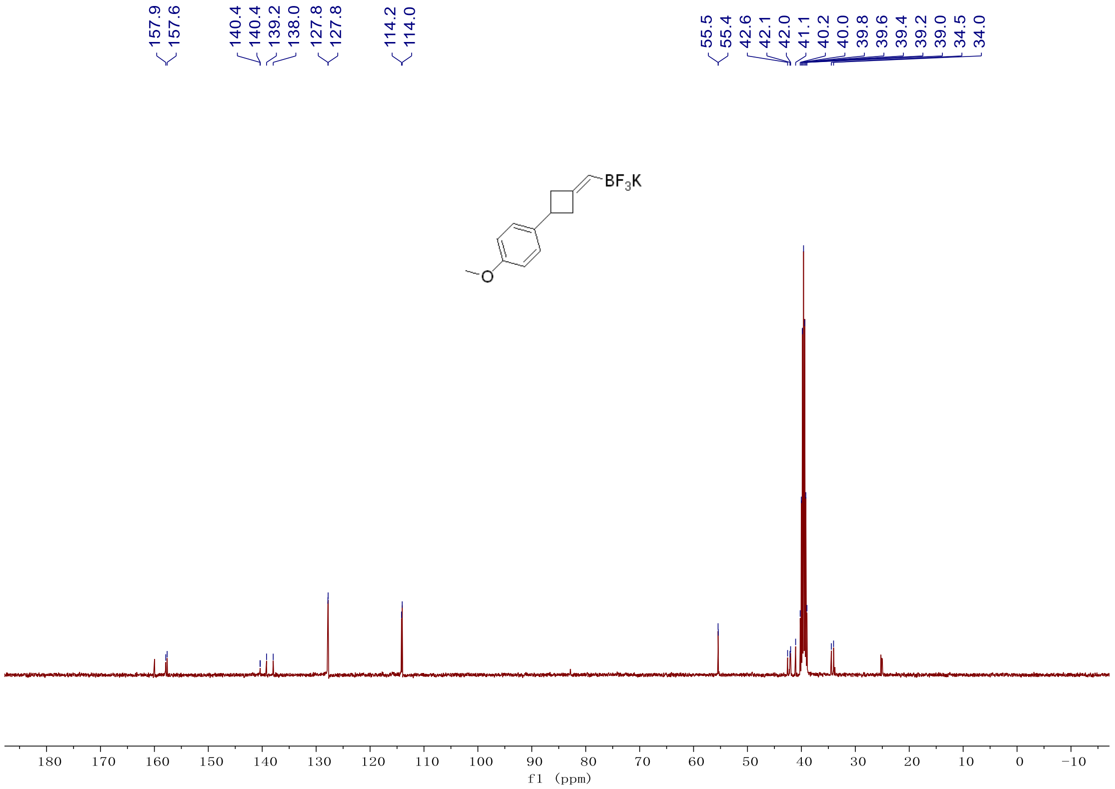

^{13}C NMR of compound 12

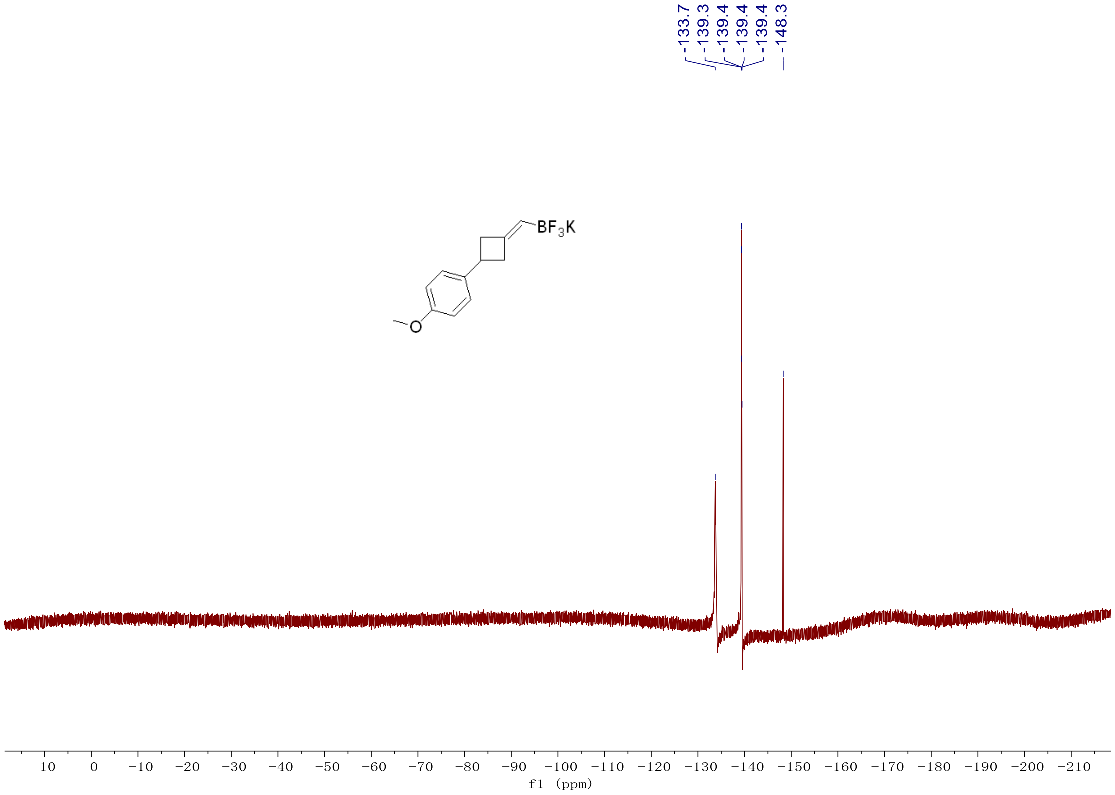

^{19}F NMR of compound 12

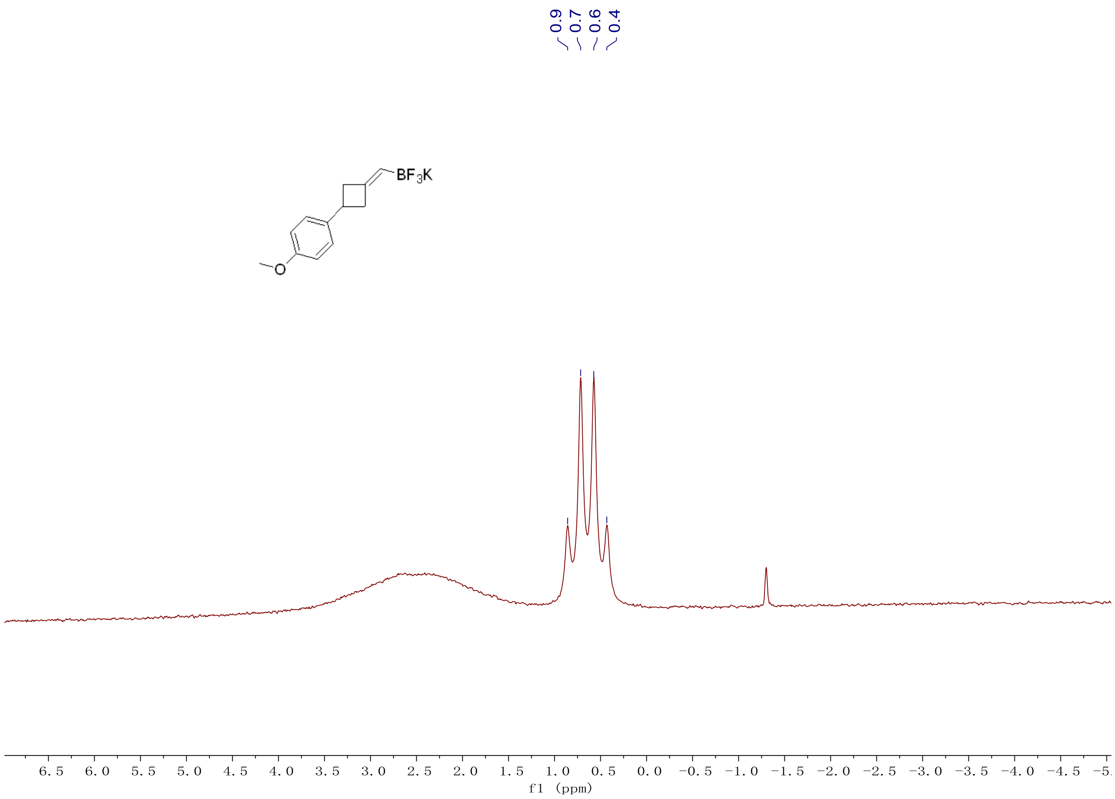

^{11}B NMR of compound 12

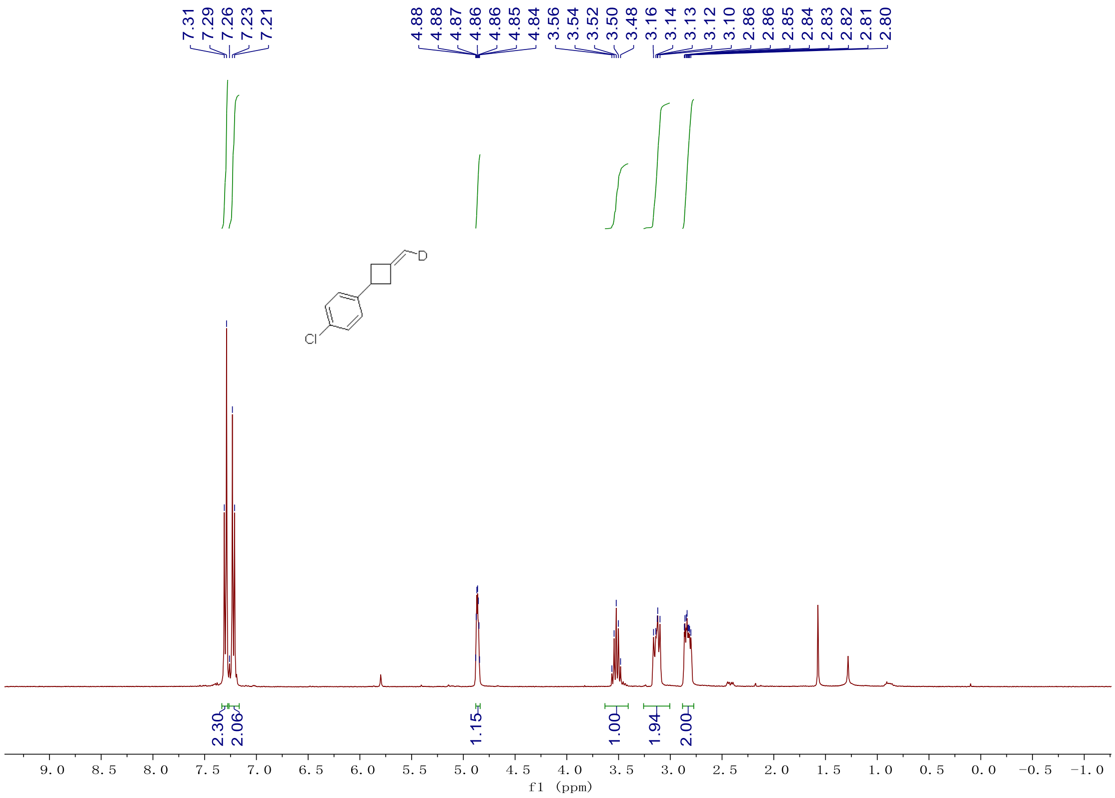

^{1}H NMR of compound 13

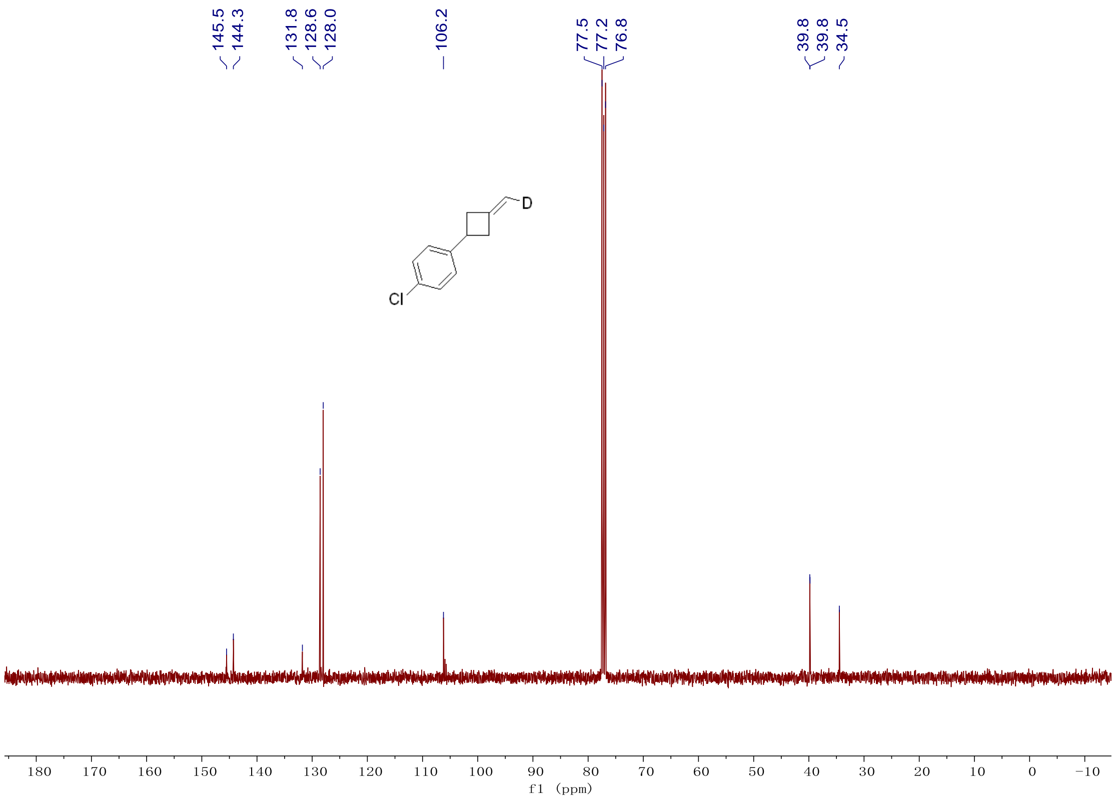

^{13}C NMR of compound 13

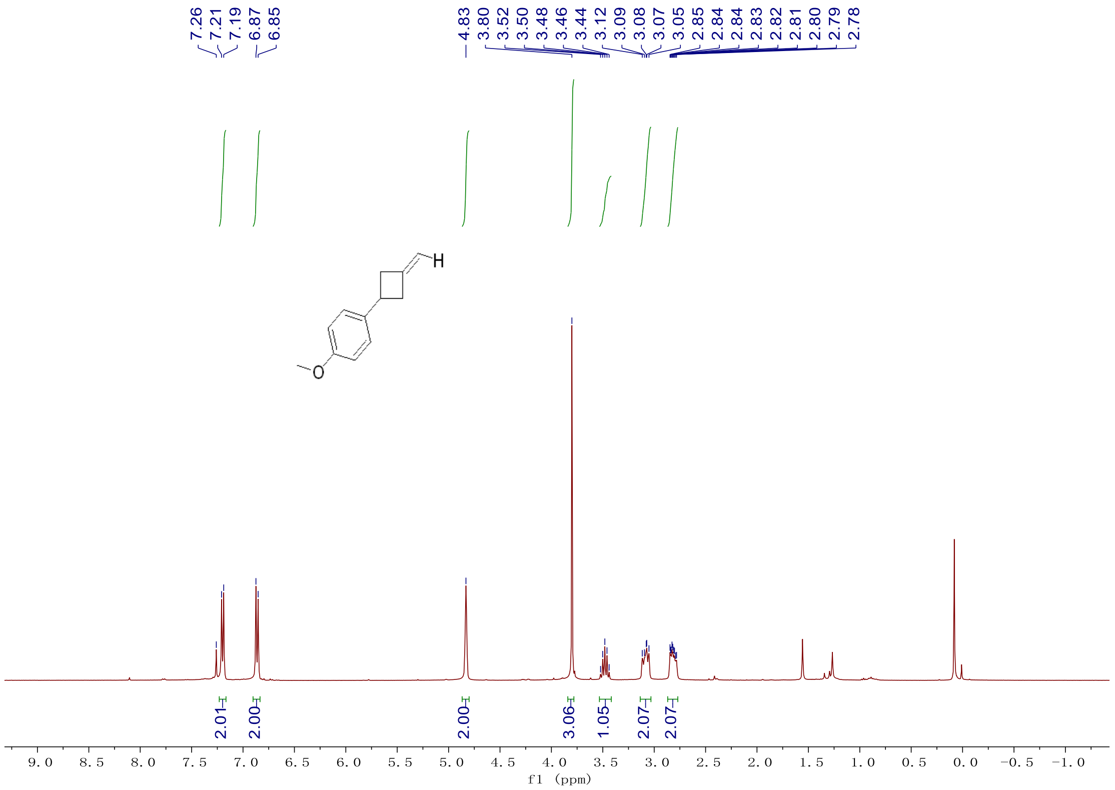

^{1}H NMR of compound 14

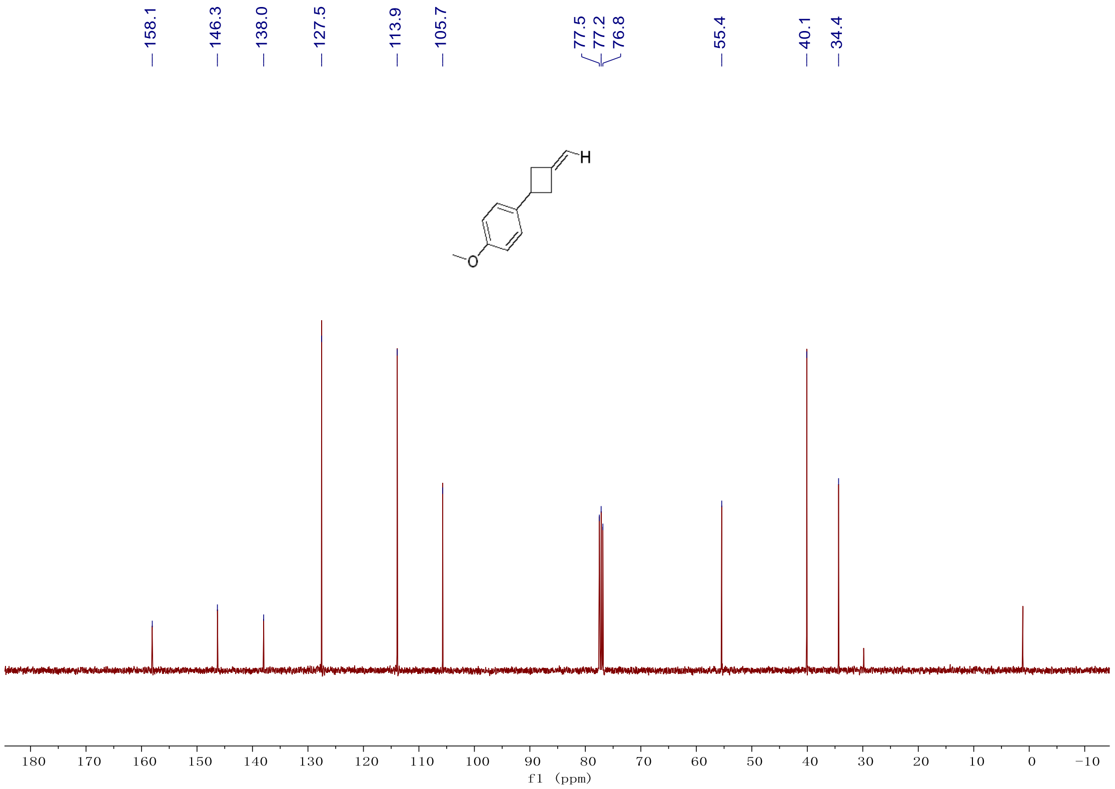

^{13}C NMR of compound 14

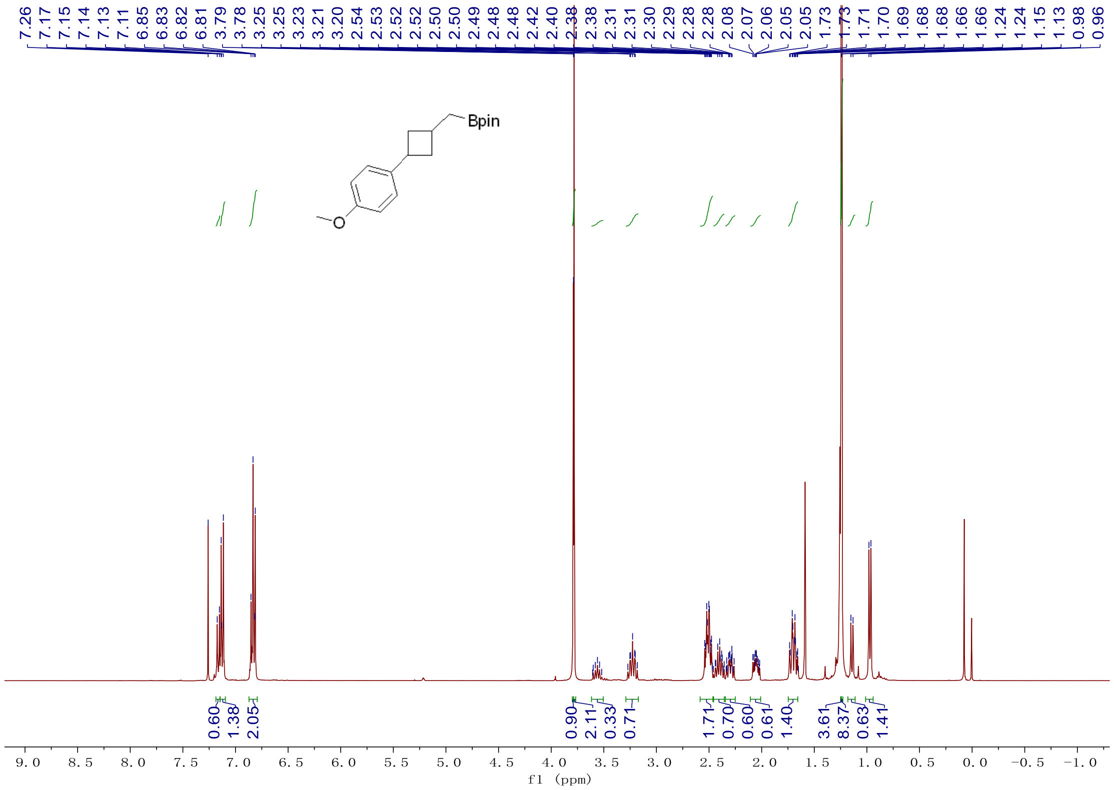

^{1}H NMR of compound 15

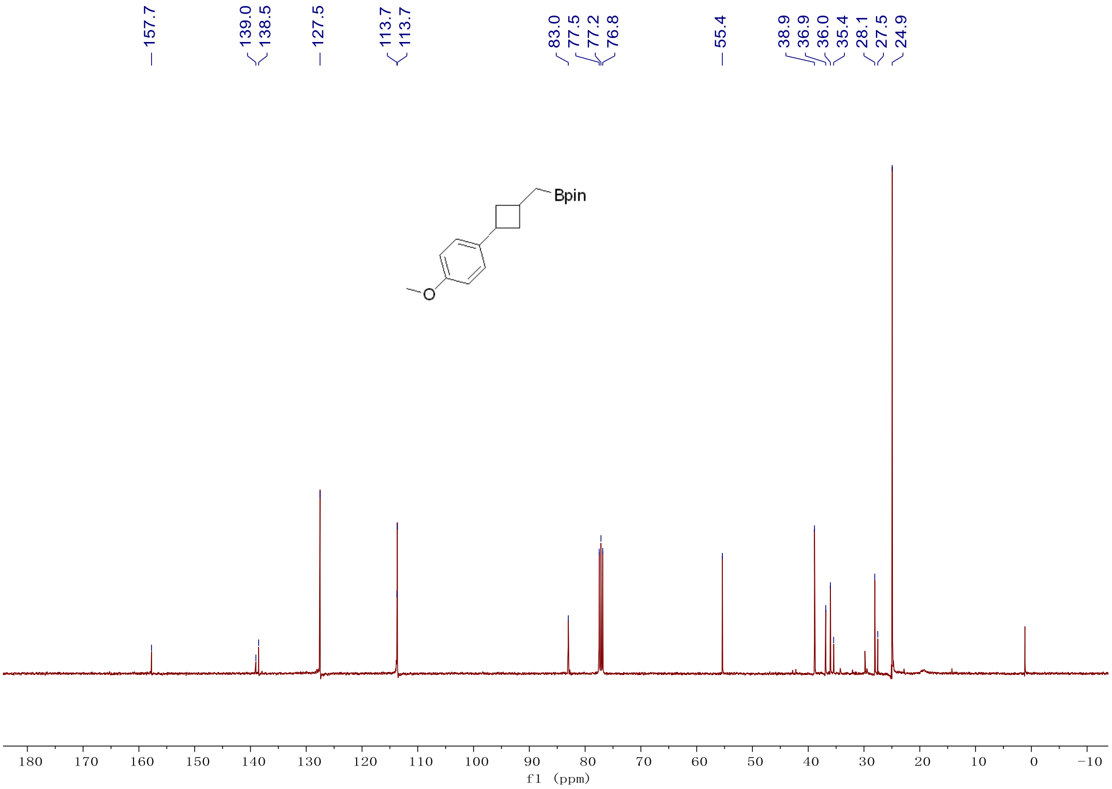

^{13}C NMR of compound 15

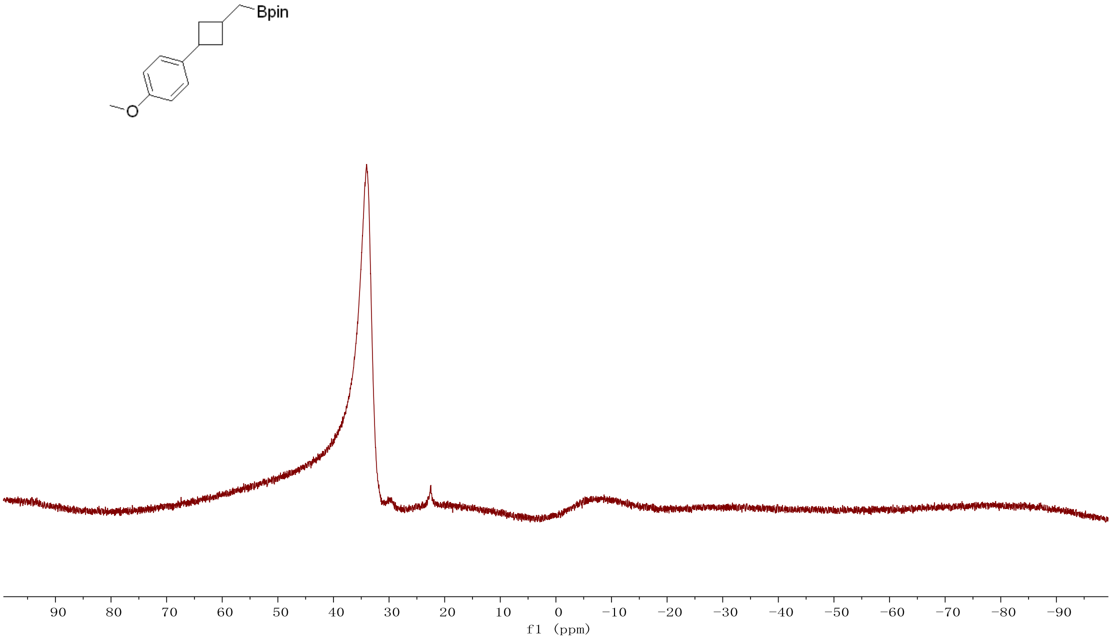

^{11}B NMR of compound 15

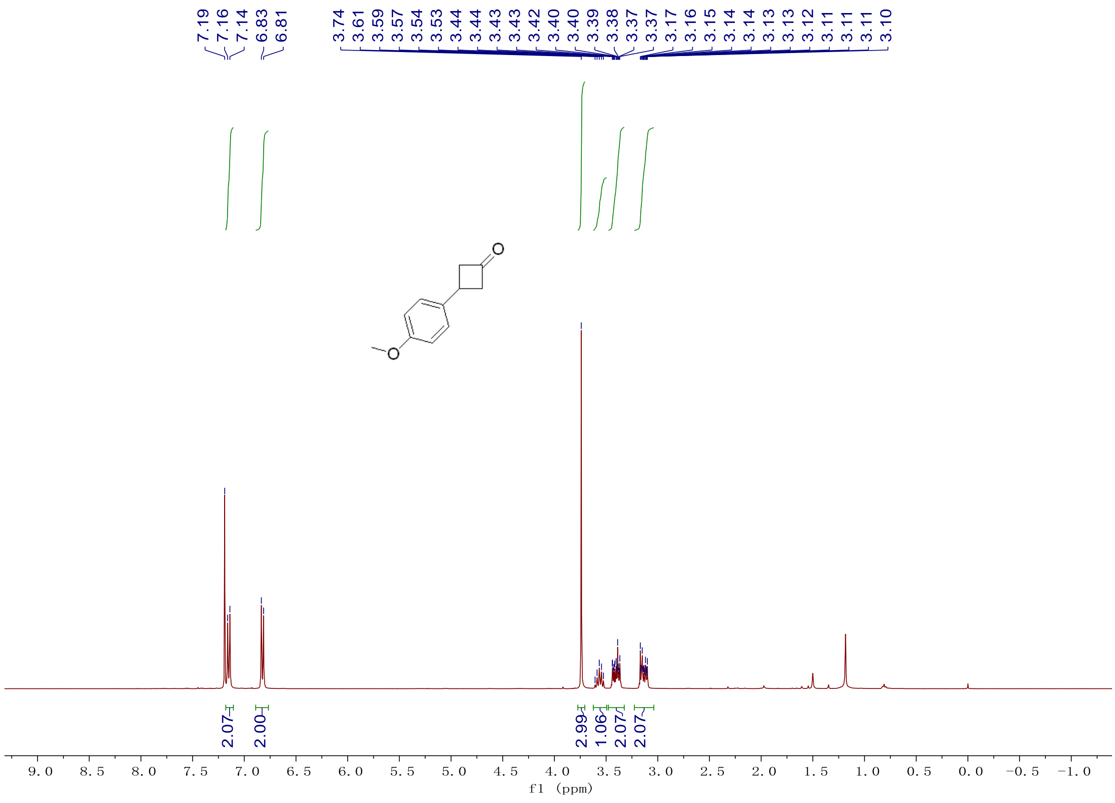

^{1}H NMR of compound 16

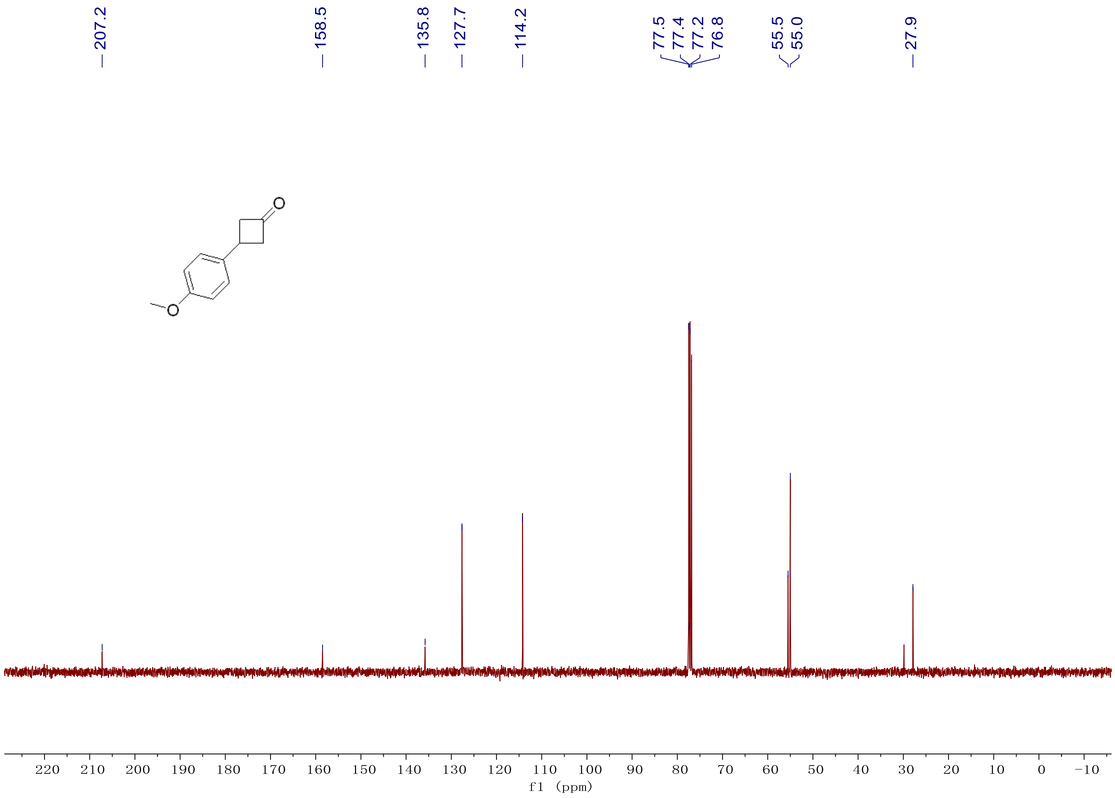

^{13}C NMR of compound 16

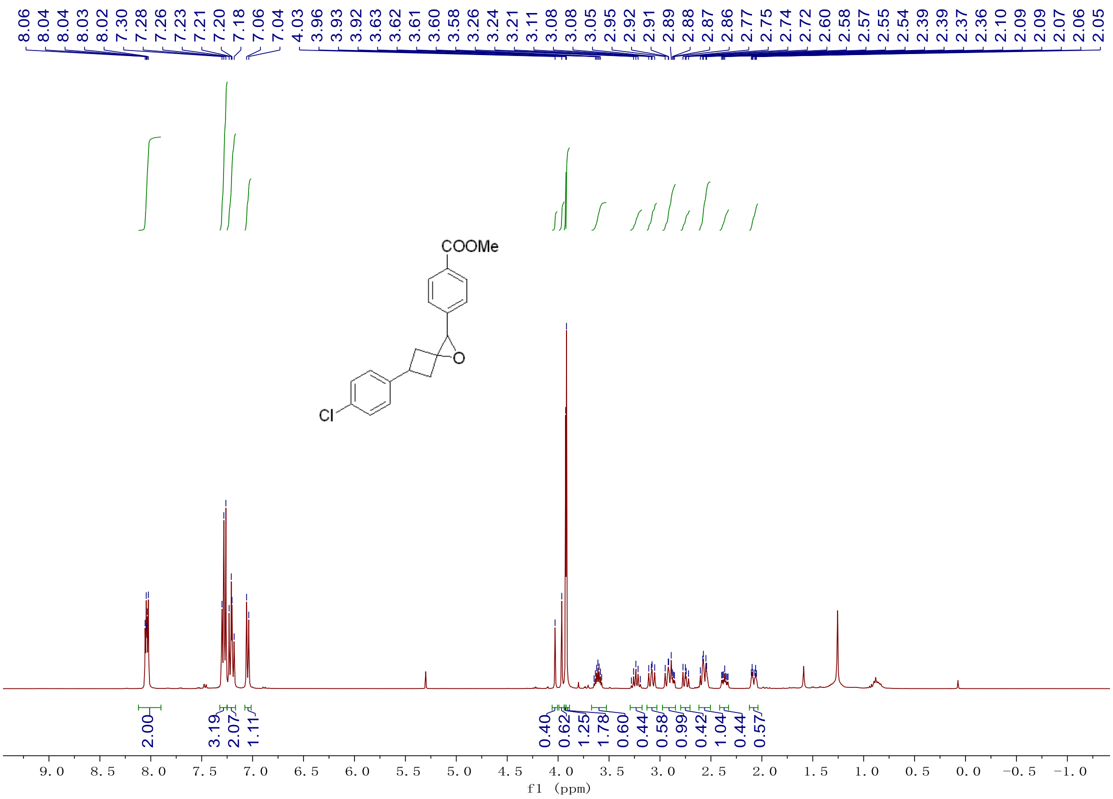

^{1}H NMR of compound 17

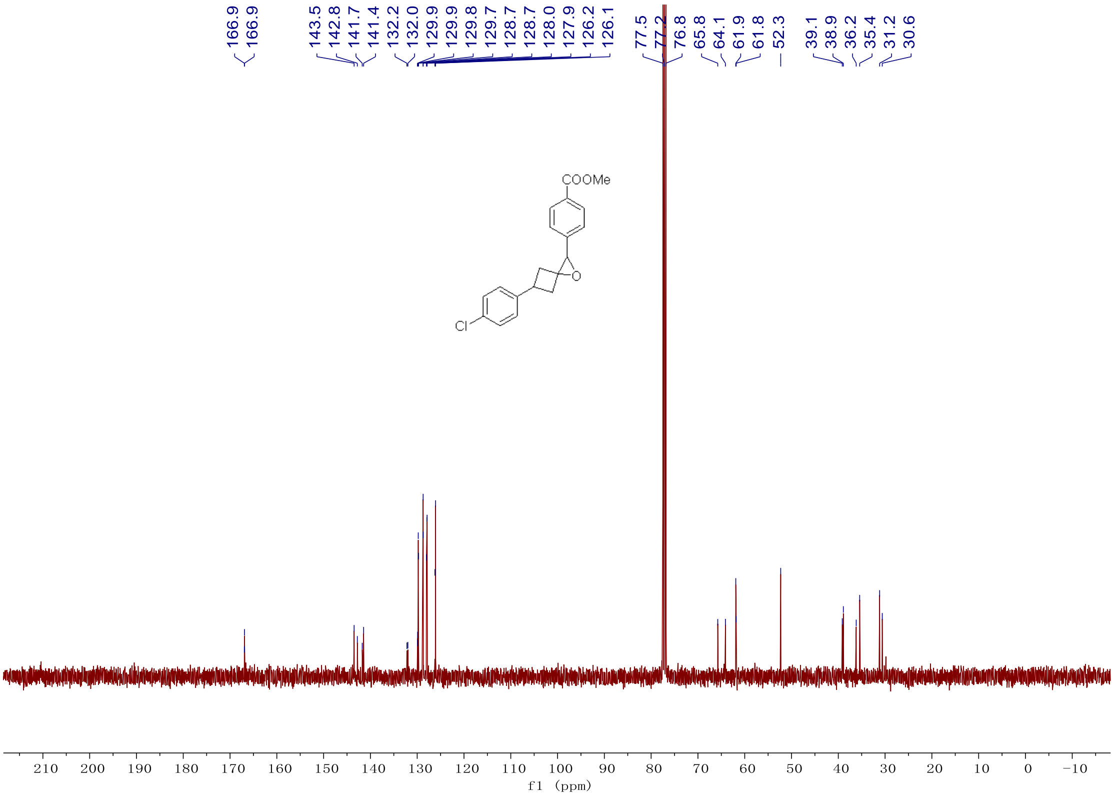

^{13}C NMR of compound 17

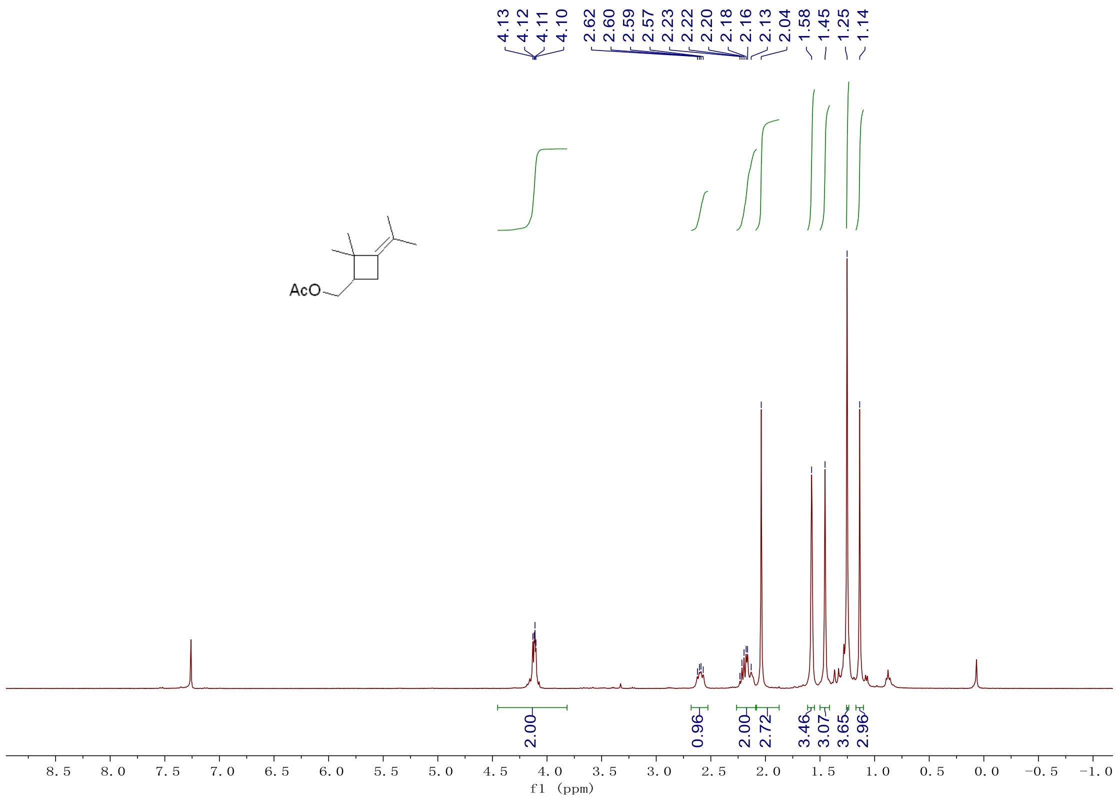

^{1}H NMR of compound 18

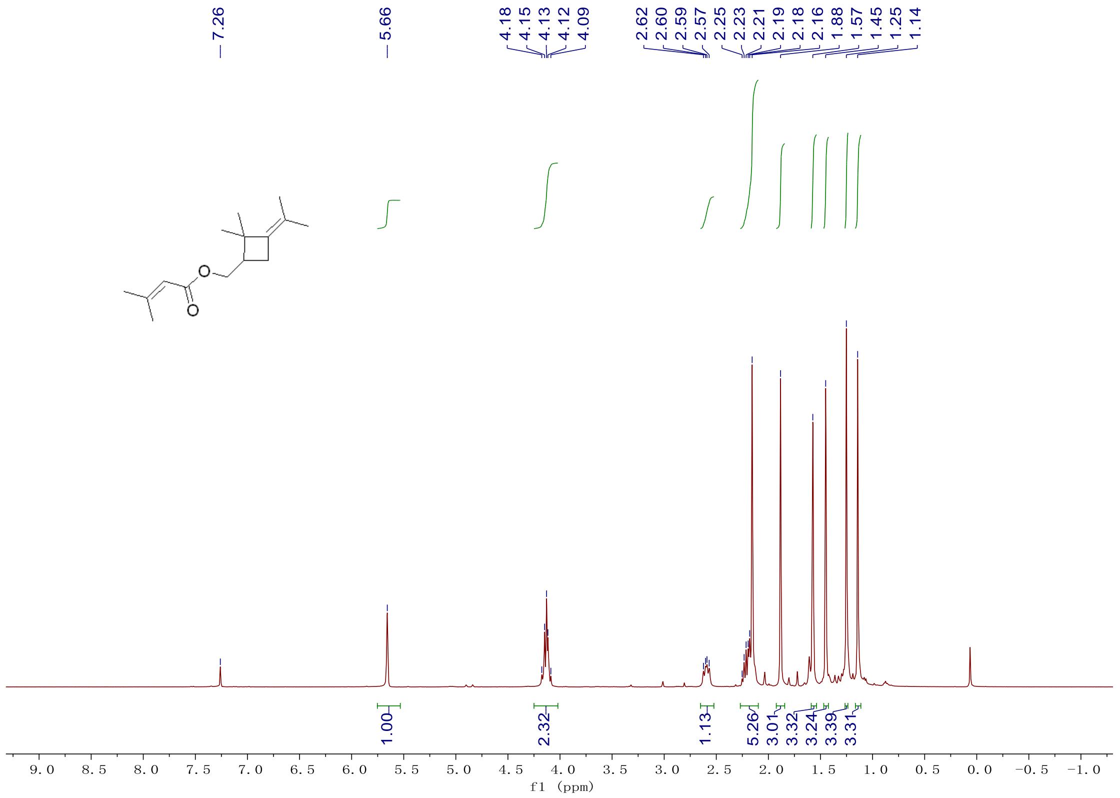

^{1}H NMR of compound 20

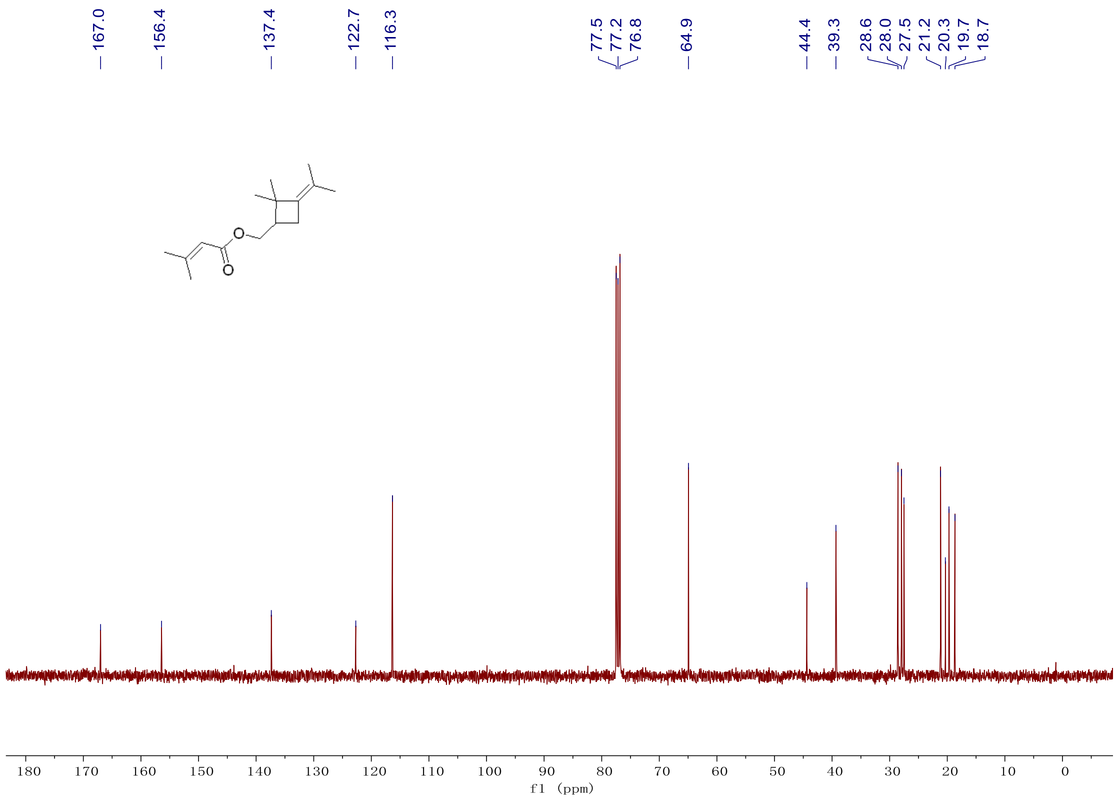

^{13}C NMR of compound 20

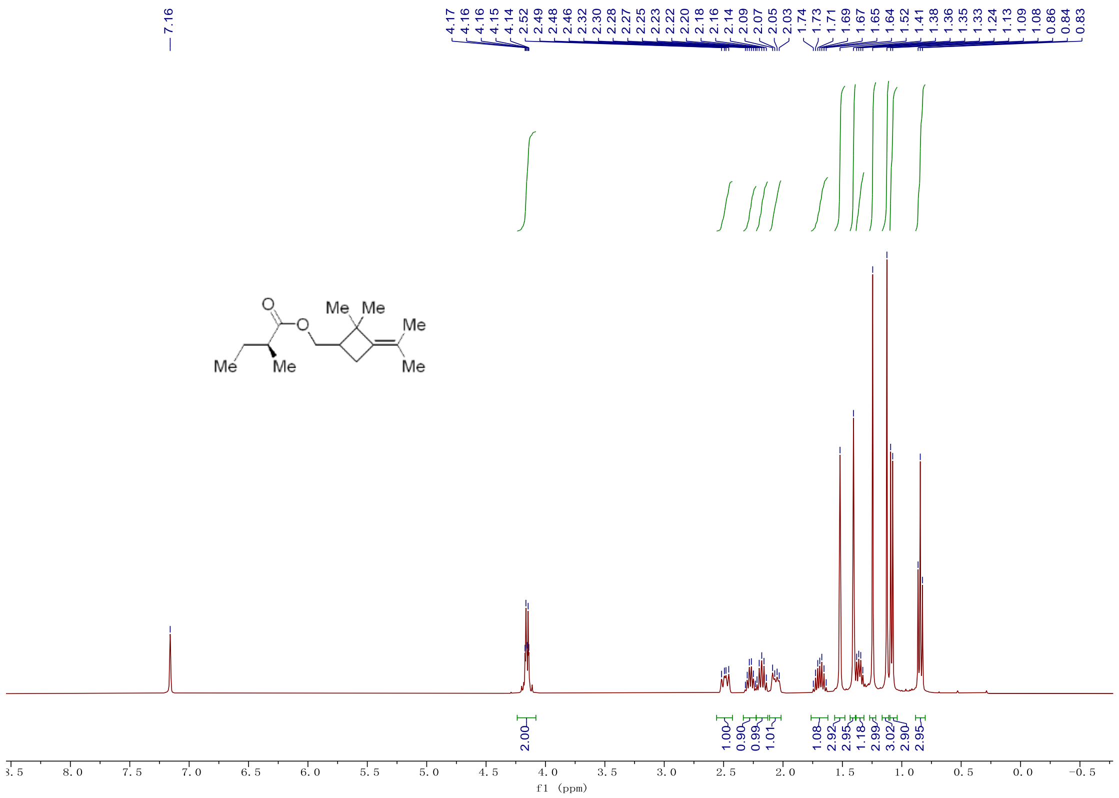

^{1}H NMR of compound 21

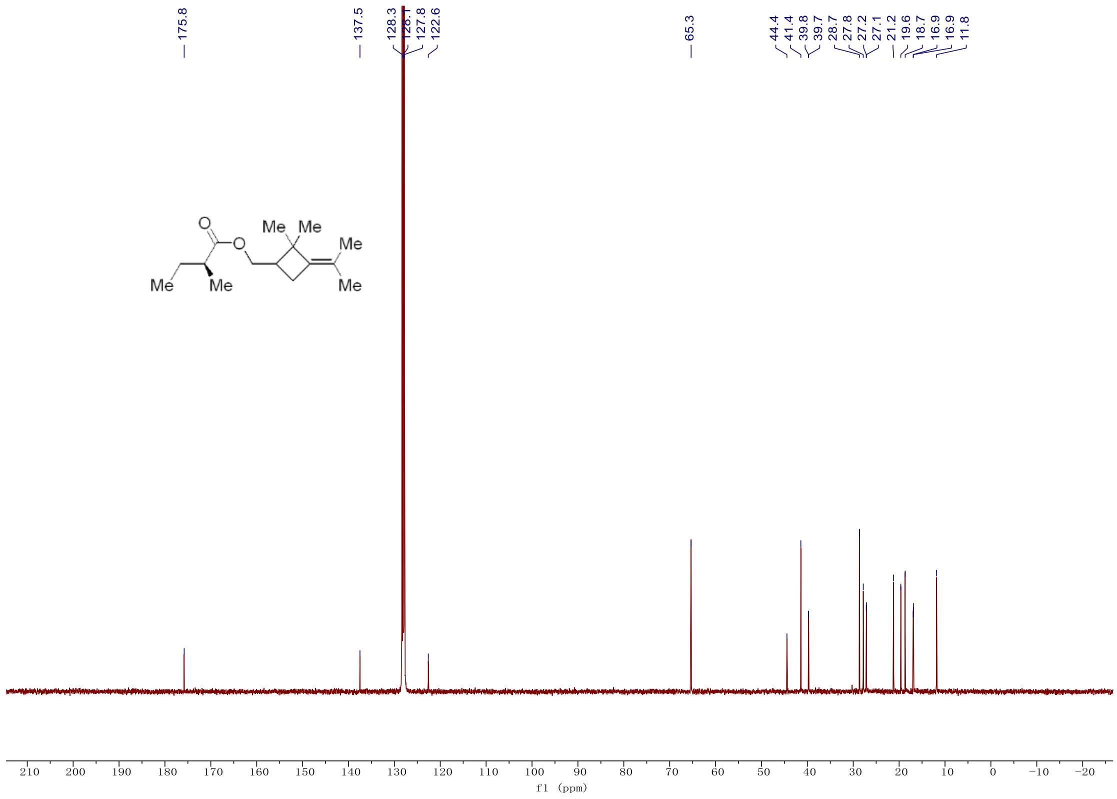

^{13}C NMR of compound 21

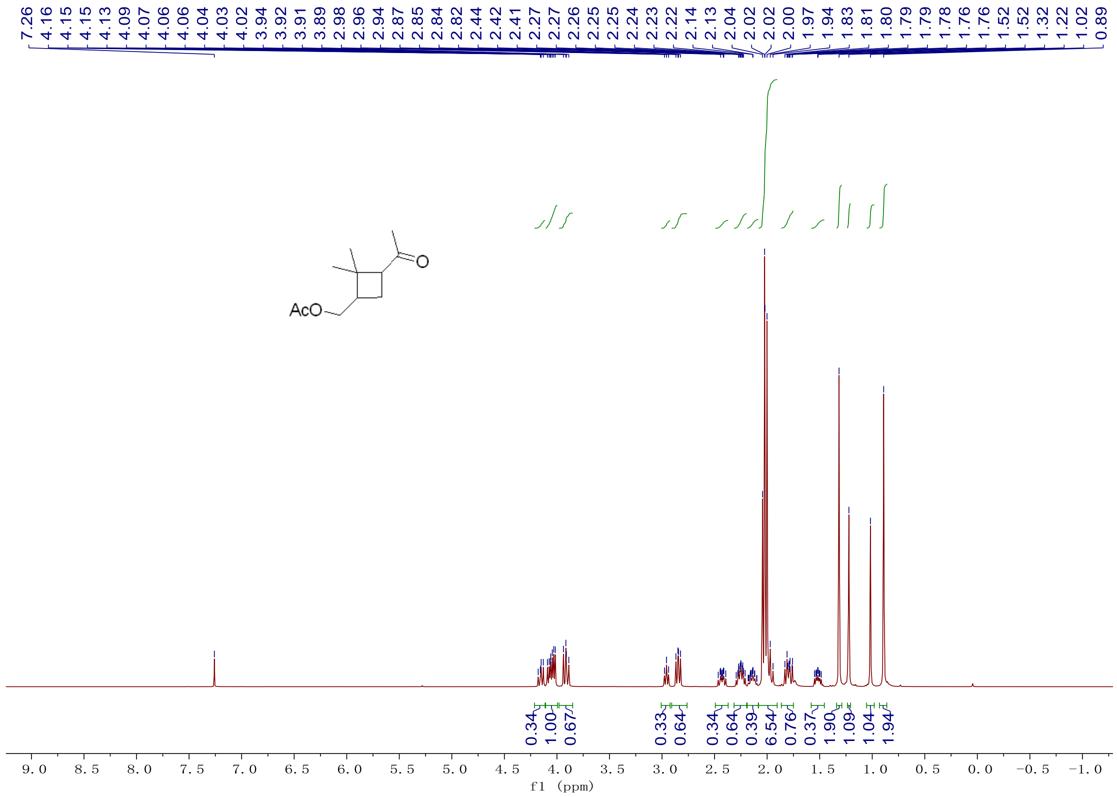

^{1}H NMR of compound 22

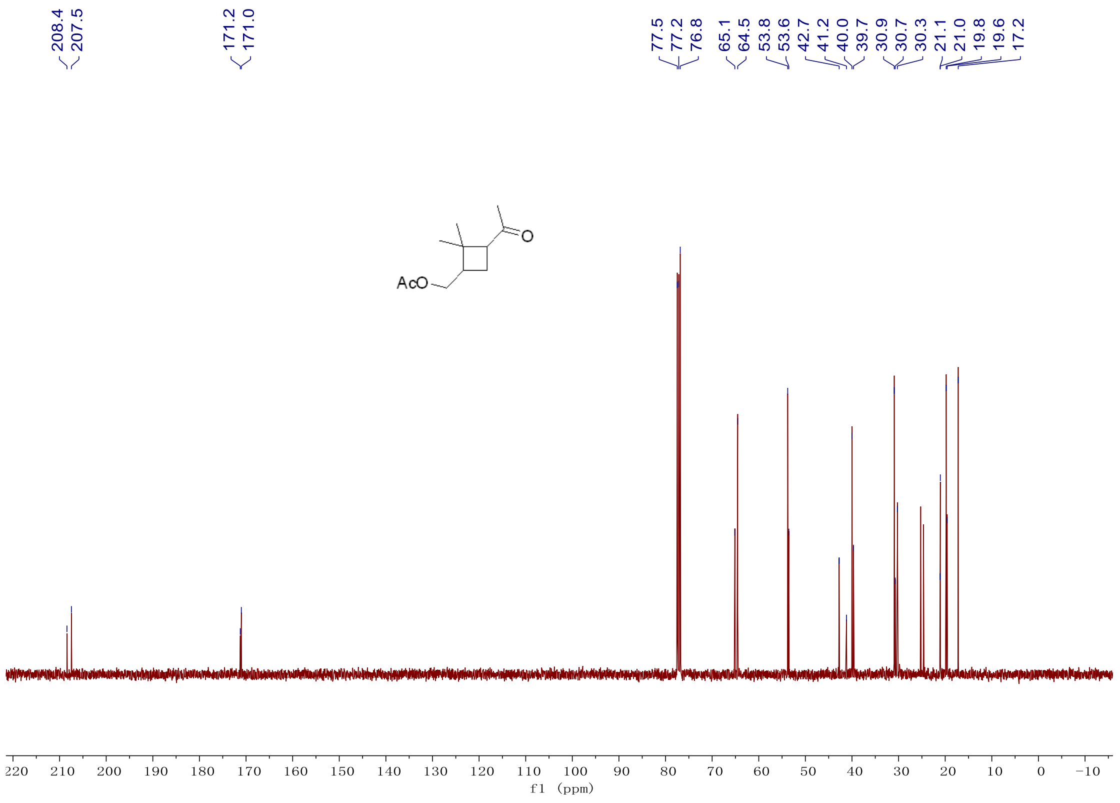

^{13}C NMR of compound 22

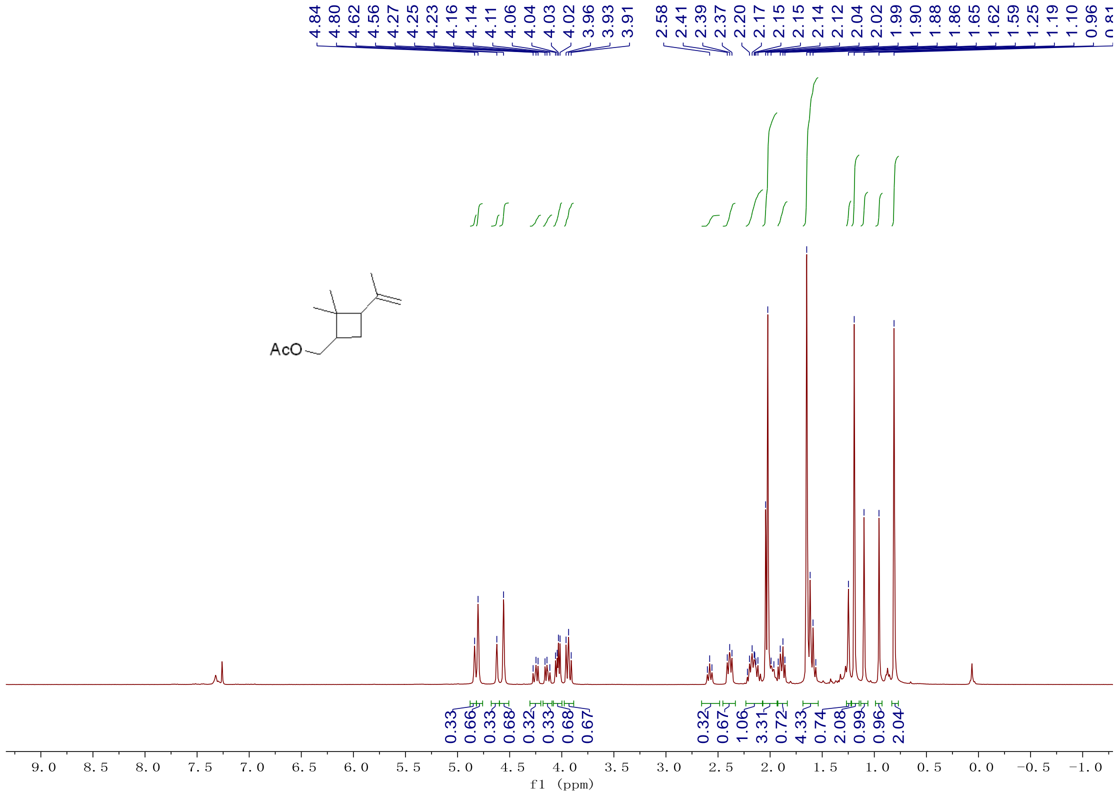

^{1}H NMR of compound 23

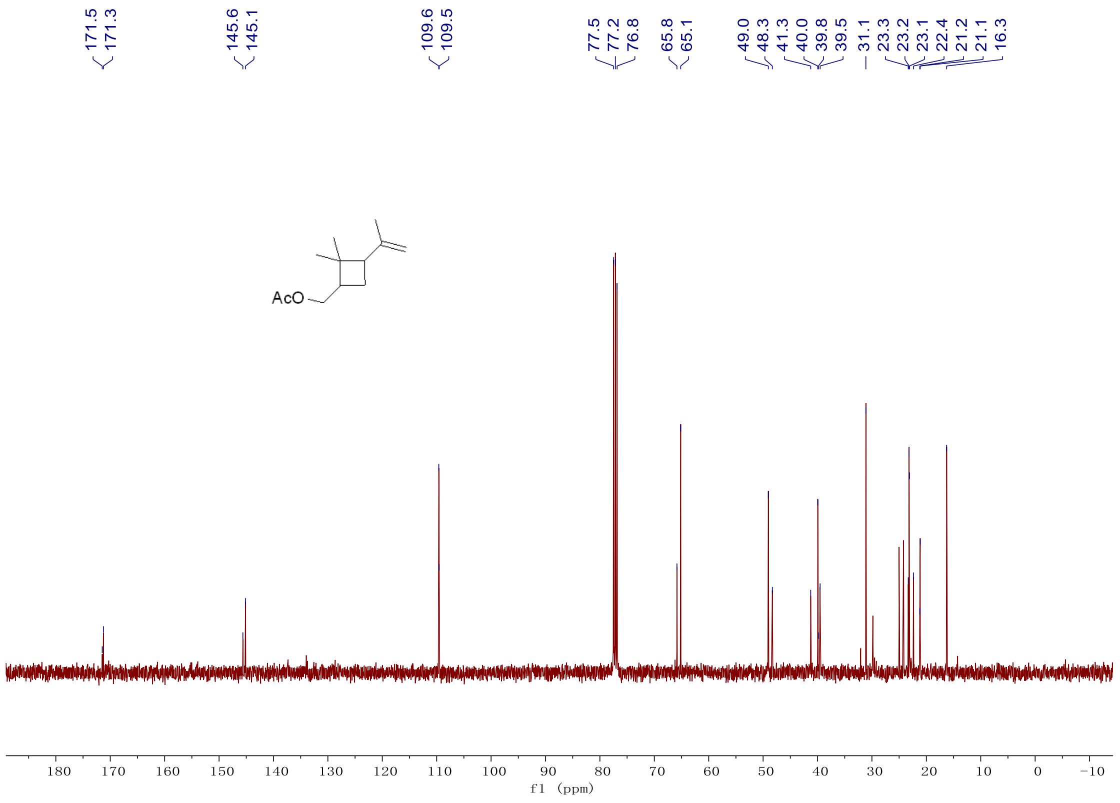

^{13}C NMR of compound 23

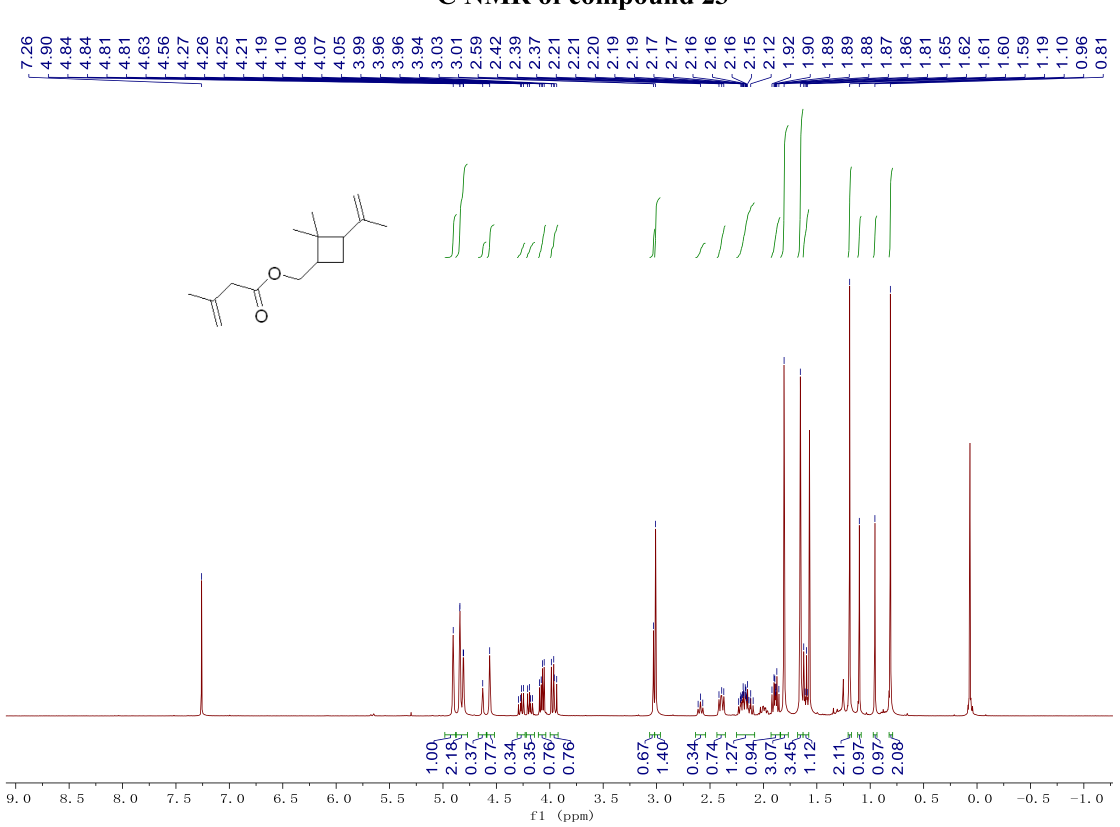

^{1}H NMR of compound 25

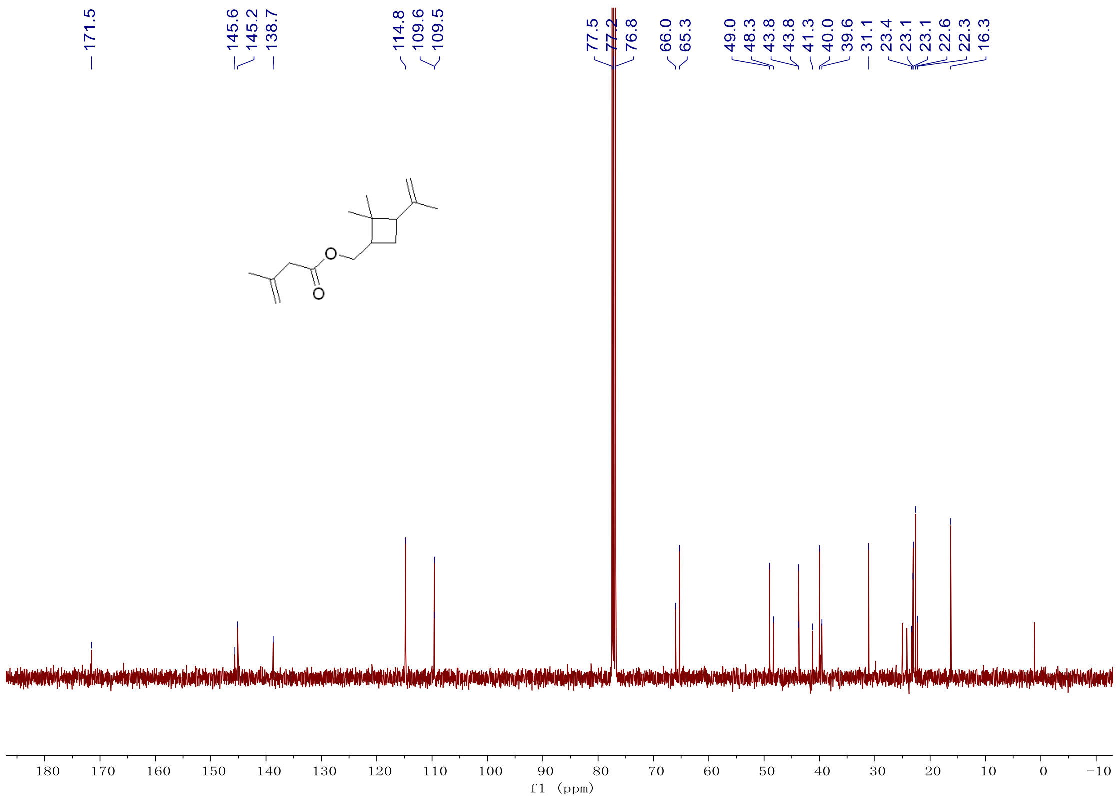

^{13}C NMR of compound 25
